# Conserved degronome features governing quality control associated proteolysis

**DOI:** 10.1038/s41467-022-35298-y

**Published:** 2022-12-08

**Authors:** Bayan Mashahreh, Shir Armony, Kristoffer Enøe Johansson, Alon Chappleboim, Nir Friedman, Richard G. Gardner, Rasmus Hartmann-Petersen, Kresten Lindorff-Larsen, Tommer Ravid

**Affiliations:** 1 Department of Biological Chemistry, The Alexander Silberman Institute of Life Sciences, The Hebrew University of Jerusalem, Jerusalem, Israel; 2 The Linderstrøm-Lang Centre for Protein Science, Department of Biology, University of Copenhagen, Copenhagen, Denmark; 3 Department of Pharmacology, University of Washington, Seattle, WA 98195 USA

**Keywords:** Protein quality control, Protein quality control

## Abstract

The eukaryotic proteome undergoes constant surveillance by quality control systems that either sequester, refold, or eliminate aberrant proteins by ubiquitin-dependent mechanisms. Ubiquitin-conjugation necessitates the recognition of degradation determinants, termed degrons, by their cognate E3 ubiquitin-protein ligases. To learn about the distinctive properties of quality control degrons, we performed an unbiased peptidome stability screen in yeast. The search identify a large cohort of proteome-derived degrons, some of which exhibited broad E3 ligase specificity. Consequent application of a machine-learning algorithm establishes constraints governing degron potency, including the amino acid composition and secondary structure propensities. According to the set criteria, degrons with transmembrane domain-like characteristics are the most probable sequences to act as degrons. Similar quality control degrons are present in viral and human proteins, suggesting conserved degradation mechanisms. Altogether, the emerging data indicate that transmembrane domain-like degron features have been preserved in evolution as key quality control determinants of protein half-life.

## Abstract (web summary)

How misfolded proteins are selected by the ubiquitin-conjugating system for elimination is largely unknown. Here, the authors identify conserved features of proteome-derived degradation signals, including amino acid and structural preferences, that trigger quality-control-associated proteolysis.

## Introduction

Intracellular protein quality control (PQC) is a principal regulatory mechanism for the maintenance of protein homeostasis^1^. PQC systems continuously survey the proteome and execute a triage of unfolded protein states, the result of which is either refolding or, if beyond repair, sequestration or degradation of aberrant proteins^2,3^. Protein refolding and sequestration are primarily mediated by molecular chaperones^4–6^, while the Ubiquitin-Proteasome System (UPS) executes quality control-associated proteolysis (QCAP)^7,8^.

A key to understanding protein homeostasis is deciphering the mode by which QCAP pathways discern the folding state of proteins. It has been established that the ubiquitin conjugation system, via E3 ubiquitin-protein ligases and auxiliary chaperones, recognizes degradation determinants termed degrons^9^ that constitute inherent sequences and structural features, as well as acquired post-translational modifications^10^. To date, degrons have been mostly identified through studies of regulated protein degradation mechanisms, such as those involved in cell division and cancer-related diseases^11,12^. These studies identified inherent degrons as short motifs, such as the destruction box of cyclins, as well as acquired degrons that are activated by phosphorylation or other post-translational modifications. However, the repertoire of known degrons cannot explain the large diversity in half-lives exhibited by the proteome^13,14^.

Our earlier work exposed the large sequence heterogenicity of the cellular degron landscape (degronome) in yeast^15^, which led to the proposition that the majority of the eukaryotic proteome contains cryptic QCAP degrons that may become exposed naturally or under misfolding conditions, such as cellular and environmental stresses^16^. These degrons target protein ubiquitination via the activity of a relatively small number of designated QCAP E3 ligases, suggesting that each recognizes a large and possibly diverse set of substrates^17^. Furthermore, QCAP E3 enzymes can act redundantly in the ubiquitination of their substrates, seemingly exhibiting overlapping recognition mechanisms^18–20^. However, the significance of this functional redundancy is not yet fully understood.

Here we describe a yeast-adapted Global Protein Stability (GPS)-peptidome technology (yGPS-P) originally established for the discovery of degrons in mammalian cell lines^21,22^. By employing a peptide library fused to yGPS-P, we have identified multiple degron sequences that were subsequently analyzed using a machine learning algorithm. The resulting computer program termed Quality Control Degron Prediction (QCDPred)^23^ revealed amino acid preferences in QCAP degrons. The determined degron features were highly dependent on the overall hydrophobicity, and consistently transmembrane domains (TMDs) exhibit extreme degron potency, signifying their critical role in the degradation of integral membrane proteins prevented from entering the secretory pathway.

## Results

### Yeast-based GPS-peptidome technology

**Fig. 1:**
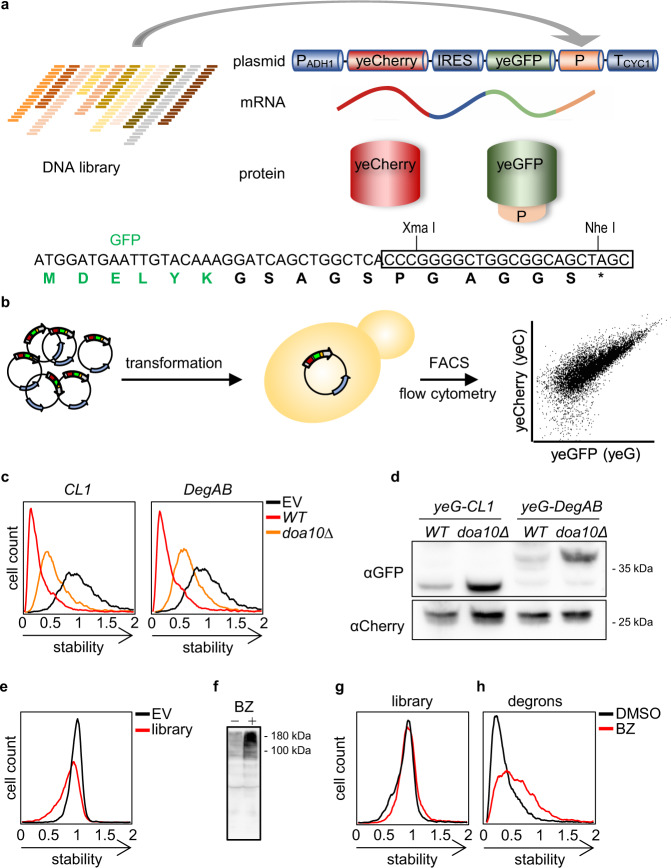
Principles and validation of the yGPS-peptidome method. **a** Schematic representation of yGPS-peptide library (yGPS-P_lib_) where a DNA library comprised of 51-mer tiled DNA fragments from 326 proteins, represented by different colors, are cloned in-frame downstream to GFP in the yGPS-P vector. The single mRNA product consists of yeCherry-IRES-yeGFP-peptide. DNA and protein sequences illustrate the cloning site that pursues GFP and the pentameric linker. **b** Flowchart of the degron screen. yGPS-P_lib_ is transformed into yeast, followed by flow cytometry or FACS. **c** Flow cytometry histograms of *CL1* and *DegAB* degrons appended to yGPS-P, in *wild type* and *doa10∆* cells. Fluorescence emissions of 10,000 cells were determined for each condition. Stability scale: Median value of the yeG/yeC ratio in empty vector (EV) control was set as one. All other histograms were distributed accordingly. **d** Immunoblot analysis of the levels of *CL1-* and *DegAB*- appended GFP compared to Cherry loading control. This analysis was repeated three times. yeG: yeGFP. **e** Flow cytometry histogram of normalized yeG/yeC in yGPS-P_lib_ compared to empty vector control. Stability scale was set as explicated in Fig. 1c. **f**, **g** Proteasome dependence of GFP-appended peptides. Cells expressing yGPS-P_lib_ were treated with 10 µM Bortezomib (BZ) for 4 h or with DMSO vehicle control. Cells were subjected to immunoblotting with anti-ubiquitin Abs (Fig. 1f), or to flow cytometry analysis (Fig. 1g). The immunoblot was repeated two times. **h** Flow cytometry analysis of a pre-selected degron library composed of top10% degrons, with or without BZ. Stability scale was set as explicated in Fig. 1c. Source data for panels c-h are provided as a Source Data file.

To set up a comprehensive degron discovery platform in the yeast *S. cerevisiae* proteome, we applied a fluorescence-based GPS technology^21^, previously developed in cultured human cells^22^. yGPS-P utilizes a bicistronic gene expression system in which codon-optimized versions of yeast-enhanced Cherry (yeC) and yeast-enhanced GFP (yeG) are expressed from a single transcript. The two proteins are, however, translated separately due to the presence of an Internal Ribosome Entry Site (IRES) upstream to GFP that allows translation initiation in a cap-independent manner^24^ (Fig. 1a). A yeast GPS peptidome library (yGPS-P_lib_) is generated by subsequent in-frame insertion of proteome-derived DNA fragments downstream to GFP in a yGPS-P vector (Fig. 1a). The plasmid library is transformed into yeast, followed by quantitative flow cytometry or Fluorescence-Activated Cell Sorting (FACS) (Fig. 1b). As both Cherry and GFP are expressed from a single transcript yet translated independently, Cherry levels reflect the basal expression of the reporters while the ratio between GFP to Cherry (shown henceforth as yeG/yeC) determines the relative GFP protein level that is governed by the fused peptide.

To validate the use of yGPS-P as a degron discovery platform, we examined the competence of two well-characterized QCAP degrons of the Doa10 E3 ligase, *CL1* and *DegAB*^25–27^ to trigger degradation of the otherwise stable GFP. Doa10 is an endoplasmic reticulum (ER)-embedded enzyme that operates in ER-associated degradation (ERAD)^28^. When the yeG/yeC ratio in cells expressing the fused degrons was compared to empty vector control, a more than 5-fold decrease was observed, presumably due to UPS-mediated proteolysis (Fig. 1c). An increase in yeG/yeC in *doa10∆* cells confirmed this assertion. That the increase in yeG/yeC is indeed a result of elevated GFP protein levels was demonstrated by immunoblot analysis of the corresponding fusion proteins (Fig. 1d).

As a source for a peptide library, we chose a subset of proteins, all components of multimeric protein complexes that potentially undergo QCAP triage^29,30^. In total, 326 yeast proteins that are part of 23 different complexes were selected (Supplementary Data 1). These proteins operate in distinct cell compartments and the composition of amino acids and secondary structure elements of the selected proteins are similar to those of the entire yeast proteome (Supplementary Fig. 1a, b). Consequently, 51-mer DNA fragments with 36-mer DNA overlaps (corresponding to 17 amino acid-length tiled peptides with 12 amino acid overlaps) were synthesized to give rise to a yGPS-P_lib_ containing approximately 29,500 DNA fragments. yGPS-P_lib_ was transformed into yeast, followed by flow cytometry determination of the yeG/yeC ratio. The observed decrease in yeG/yeC in yGPS-P_lib_ (Fig. 1e) indicates the presence of a destabilizing peptide population within the tested peptidome. To assess the contribution of degrons of the UPS to GFP destabilization, the effect of the reversible proteasome inhibitor Bortezomib (BZ) on yeG/yeC was determined. To this end, yGPS-P_lib_ was transformed into cells lacking the multidrug transporter *PDR5 (pdr5∆*) to increase drug sensitivity^31^. Comparing mock- and drug-treated cells, we observed an increase in the overall cellular levels of high molecular weight ubiquitin conjugates (confirming proteasome inhibition) (Fig. 1f), as well as a mild increase in yeG/yeC (Fig. 1g). A larger increase of yeG/yeC was obtained when a pre-sorted top 10% degron-enriched population was tested (Fig. 1h, and Supplementary Fig. 1c), confirming that changes in yeG/yeC accurately reflect susceptibility to UPS-mediated degradation.

### Mapping QCAP degrons using a machine-learning-based approach

**Fig. 2:**
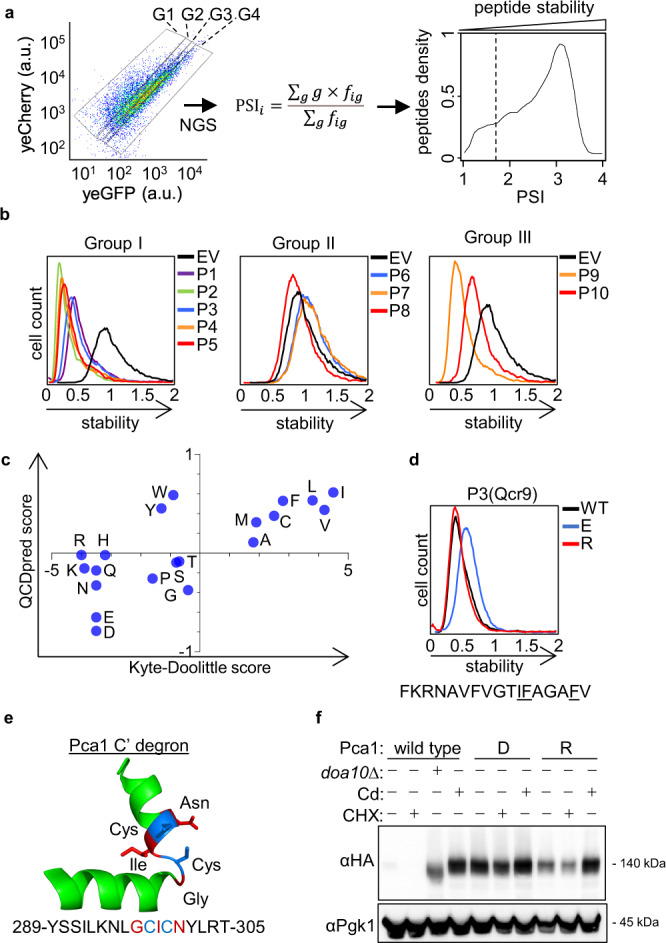
QCAP degrons functionality is largely defined by their amino acid composition. **a** Fluorescence-activated cell sorting of yGPS-P_lib_ into four gates (G1-4), each composed of 2.5 million cells, was followed by NGS and protein stability index (PSI) scoring of each peptide, leading to the formation of a peptide density map. Shown is a FACS illustration and gating of 10,000 cells. Degron cutoff at PSI < 1.7 is marked by a dashed line. a.u.: arbitrary units. **b** Validation of QCDPred-based degron predictions. Ten DNA fragments from the library were re-cloned into yGPS-P, followed by flow cytometry analysis. The corresponding peptides were divided into three different groups: Group I- PSI ≤ 1.62, *P* ≥ 0.85; Group II- PSI > 1.62, *P* < 0.85; Group III- PSI ≤ 1.62, *P* < 0.85. Stability scale: Median value of the yeG/yeC ratio in empty vector (EV) control was set as one. All other histograms were distributed accordingly. **c** Scatter plot comparing Kyte-Doolittle hydrophobicity amino acid scores and QCDPred probabilities. **d** Flow cytometry histogram of intact and mutant P3 peptide in which three amino acids were replaced with either glutamate (E) or arginine (R) residues. Stability scale was set as shown in Fig. 2B. **e** 3D structure of QCDPred-calculated cytosolic Pca1 degron (amino acids 289-305), based on AlphaFold Pca1 structural model #AF-p38360-F1 (Conf. (90 > pLDDT > 70). Marked by a blue color are the cysteine residues. Marked by a red color are the three amino acids that have been replaced with aspartate or arginine. pLDDT: per-residue confidence score on a scale of 0-100. **f** Immunoblot analysis of Pca1 protein levels. *Wild type* or *doa10∆* (*d∆*) cells, expressing the indicated HA-tagged Pca1 proteins were left intact or treated with 50 μM CdCl_2_ for 1 h. To determine degradation, where indicated, the translation inhibitor cycloheximide (CHX) was added to cells for 15 min before cell harvesting. Pca1 levels were measured by immunoblotting using anti-HA Abs while Pgk1 levels served as a loading control. This analysis was repeated two times. D: replacement of residues marked in 2E by red color with aspartate; R: replacement of residues marked in 2E by red color with Arginine. Source data for panels a-d and f are provided as a Source Data file.

To classify degron sequences within yGPS-P_lib_, mid-log-phase cells expressing the appended peptidome were separated by FACS into four gates according to yeG/yeC, each containing an equal cell number, and the identity and amounts of the peptides’ DNA in the different gates were determined by Next-Generation Sequencing (NGS) (Fig. 2a). After filtering ambiguous peptides DNA from the NGS data, the contribution of 23,600 peptides to GFP stability was calculated based on their abundance in the different gates and each was assigned a Protein Stability Index (PSI) score^21^ (Fig. 2a and Supplementary Data 2). Overall, 9.5% of the analyzed peptidome had PSI values < 1.7 (on a scale of 1 – 4), suggesting a degron function (Fig. 2a).

To identify sequence patterns within the examined degrons, we next opted to employ a machine-learning algorithm, termed QCDPred, described in detail in a companion paper^23^. Briefly, a logistic regression model was trained on the amino acid composition of each tile, together with a binary stable/unstable label derived from the PSIs. Peptides were labeled as unstable if their PSI < 2.2 and stable if their PSI > 2.8. The model achieves a good fit to the data only when using a single parameter for each of the 20 natural amino acids. The 20 parameters of QCDPred may in addition be interpreted as the contribution of each amino acid type to degron potency^23^. Consequently, protein stability profiles were formed for all proteins with at least five tiles screened (*N* = 306), each including the experimental PSI values for each peptide, average PSI values for each amino acid, and the QCDPred probability scores (Supplementary Data 3).

To experimentally validate the high-throughput procedure and test the QCDPred model, we selected ten peptide sequences that we studied one at a time, using the same reporter system and flow cytometry readout (Fig. 2b, Table 1). Five peptides, predicted by QCDPred to have degron activity (group I), and three peptides predicted not to have degron activity (group II) were confirmed in this experiment. In contrast, two peptides were found to have some degron activity even though they were not predicted as degrons by QCDPred (group III). We also tested steady-state levels of selected peptides from each group and found a good correlation: Levels of group I and group III fusion peptides was significantly lower than that of group II (Supplementary Fig. 2a). Moreover, treating the cells with BZ restored GFP fluorescence for the degron sequences, demonstrating that the lowered fluorescence is due to proteasomal degradation, lending further support that QCDPred predicts proteasomal QCAP substrates (Supplementary Fig. 2b).

**Table 1:** List of selected peptides, their origin, sequence, PSI, and GRAVY scores

name	Origin	PSI score	QCDPred score	K&D GRAVY	Sequence
Test set					
P1	Cob1 184–200	1.39	0.99	2.19	YLVPFIIAAMVIMHLMA
P2	Pci8 304–320	1.44	0.98	0.41	IMRCKIYFFYLRISKKL
P3	Qcr9 11–27	1.44	0.89	1.19	FKRNAVFVGTIFAGAFV
P4	Rps26A 69–85	1.57	0.93	0.29	NKLHYCVSCAIHARIVR
P5	Cyt1 279–295	1.6	0.97	0.73	SLYLLSIWVKKFKWAGI
P6	Cyt1 136–152	3.25	0	−2.34	DEPDEQGNPKKRPGKLS
P7	Itc1 301–317	3.27	0.01	−2.14	SGKSNTSNDASNKKETK
P8	Pci8 383–399	3.04	0.13	−0.68	VIDKLKNENTDLKDIIQ
P9	Rpc19 101–117	1.55	0.48	0.12	LNIRIQTYGETTAVDAL
P10	Rpc53 359–375	1.58	0.62	−0.06	KVGSIRVHKSGKLSVKI
Scrambled peptides					
SC1	Cob1 184–200		0.99	2.19	ILFMVAIPVHAIAYLMM
SC2	Cob1 184–200		0.99	2.19	HAAIMYMAVIVLIFLMP
Modified peptide P3					
3E	Qcr9 11–27		0.18	−0.02	FKRNAVFVGTEEAGAEV
3R	Qcr9 11–27		0.60	−0.19	FKRNAVFVGTRRAGARV

*PSI* Protein Stability Index.

*GRAVY* Grand average of hydropathy (Based on Kyte & Doolittle scale).

As projected from previous studies of QCAP degrons, QCDPred scores of most amino acid types are correlated with their Kyte-Doolittle hydrophobicity scores, and indeed, all hydrophobic amino acids contributed positively to QCAP degrons’ probability (Fig. 2c). Conversely, QCDPred scores of the two negatively charged amino acids, glutamate and aspartate, were significantly lower than others, suggesting that the presence of negatively charged amino acids specifically interferes with a QCAP degron’s function. According to the prediction, inserting negatively charged amino acids into a peptide degron significantly reduces its QCDPred score (As an example, see Supplementary Table 1). This was confirmed experimentally by replacing three amino acids in a re-evaluated proteasome-dependent degron peptide P3 from the yeast protein Qcr9 with either glutamate or arginine (Fig. 2d). We concluded that the classified QCAP degronome prefers hydrophobic residues while negatively charged amino acids are disfavored.

We next examined these assertions on a native QCAP degron from the P-type Cation-transporting ATPase Pca1, that was not in our tested degron cohort. Under standard growth conditions, Pca1 is constitutively degraded via Doa10, which recognizes a cysteine-enriched degron localized within amino acids 271–320 of the cytosolic and solvent-exposed N-terminal region of Pca1^32^. However, cadmium sensing by the degron enables Pca1 to circumvent ERAD^32^. As this region is too long for precise analysis, we used QCDPred to locate a shorter sequence that defines the operational degron between amino acids 289–305, which was predicted by AlphaFold^33,34^ to form an exposed helix-turn-helix structure (Fig. 2e). Examining Pca1 steady-state levels, we observed that replacing three amino acids in the degron core (Fig. 2e) with aspartate residues greatly stabilized the protein while replacing the same amino acids with arginine residues only showed moderate stabilization (Fig. 2f). Importantly, mutant Pca1 still retained cadmium sensitivity (Fig. 2f), indicating overall structural preservation. These data confirmed our assertion that negatively charged amino acids greatly interfere with native QCAP degron’s function. The data also demonstrated the capability of QCDPred to identify functional degrons within the proteome while in their physiological context.

### Transmembrane domains function as QCAP degrons

**Fig. 3:**
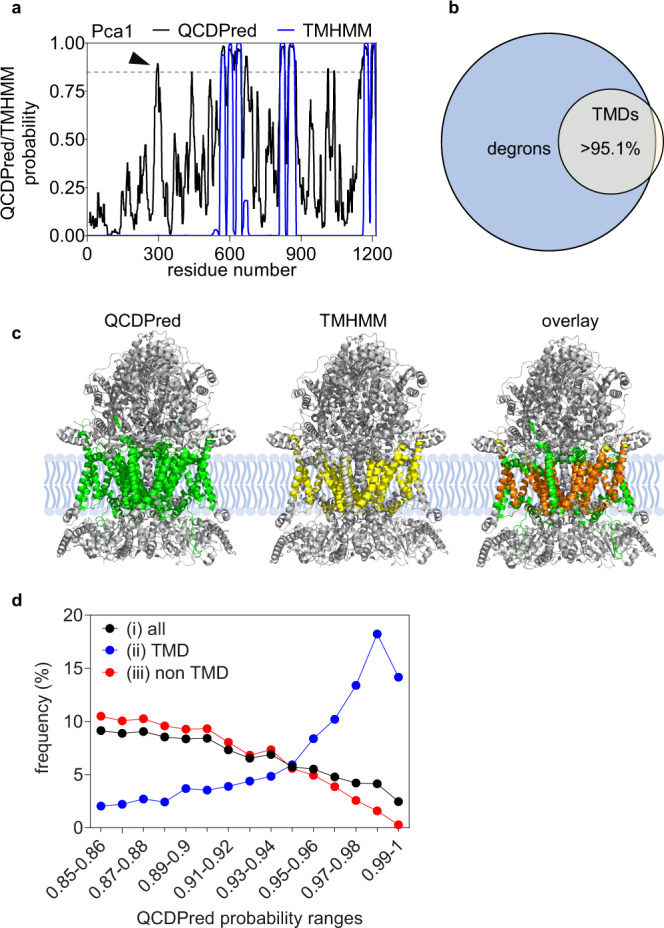
QCDPred assignment of TMDs as QCAP degrons. **a** Map of degrons prediction by QCDPred and TMDs prediction by TMHMM within Pca1. Marked by an arrowhead is the Pca1 cytosolic degron. A degron cutoff probability is marked at P = 0.85 by a dashed gray line. **b** Venn diagram of the relationship between yeast TMDs, extracted from the TM Helix Hidden Markov Model (TMHMM) algorithm, and QCDPred degron probabilities of the entire yeast proteome (*P* ≥ 0.85). **c** Assignment of QCDPred calculated degrons (in green) and TMHMM predicted TMDs (in yellow) to the cytochrome b-c1 complex (PDB #6T0B^70^). overlay regions are colored in orange. **d** Frequency distribution of (i) all QCDPred assigned degrons within the yeast proteome (*P* ≥ 0.85), (ii) QCDPred assigned degrons within the yeast proteome composed of TMDs, and (iii) QCDPred assigned degrons within the yeast proteome that are not part of a TMD, sorted into probability ranges between 0.85 −1.

Intriguingly, besides the aforementioned cytosolic degron, QCDPred also assigned remarkably high degron probabilities (P ≥ 0.93) to Pca1 TMDs (Fig. 3a). This was unexpected, not only because QCDPred was not programmed to consider protein topology, but also because TM proteins comprised only a minute proportion of the proteins included in the screen (1.79%) and hence their small contribution to the algorithm. The observation is, however, in line with QCAP degrons being hydrophobic (Fig. 2c). Thus, when applying QCDPred to the entire yeast proteome, the vast majority of TMDs were assigned as degrons (Fig. 3b). Our experimental peptidome data agree with this prediction, demonstrating that 12 out of 13 peptides localized to TMDs of the inner mitochondrial cytochrome b-c1 respiratory complex^35^, the only TM-embedded complex in our peptidome cohort (Supplementary Data 1), function as degrons (Fig. 3c and Supplementary Fig. 3). In line with these findings, most predicted TMD degrons in the yeast proteome were enriched in the highest QCDPred score range of 0.95–1.0 while the rest of the degrons were underrepresented in this range (Fig. 3d). Thus, according to QCDPred, TMDs comprise the most potent QCAP degron sequences. Hence, the hydrophobic sequence and possibly structural resemblance to TMDs is likely a significant feature of QCAP degrons. TMDs themselves could be relevant as QCAP degrons in cases when TM proteins fail to insert correctly in membranes (see next).

**Fig. 4:**
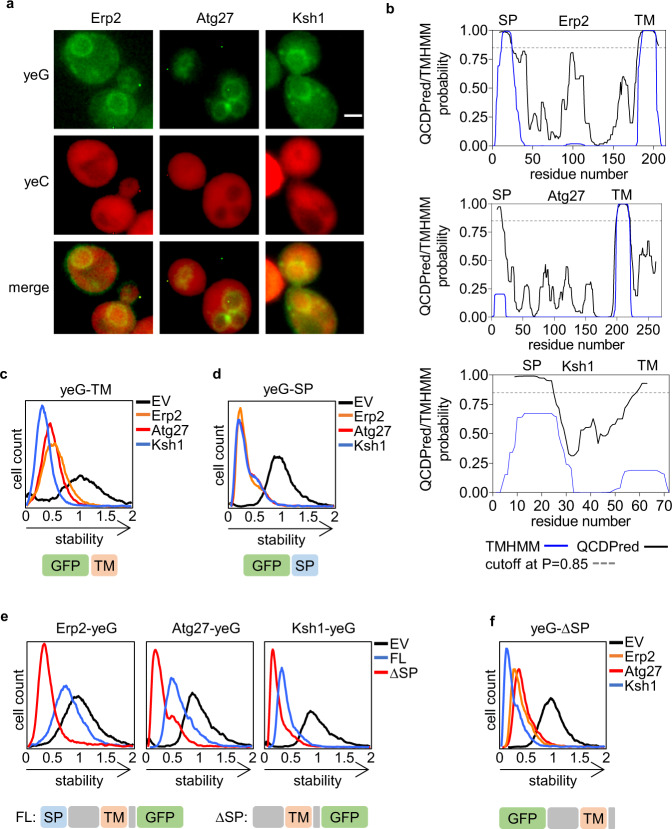
Transmembrane domains function as QCAP degrons. **a** Represantitive fluorescence images demonstrating membrane localization of the single-pass type-1 TM proteins, Erp2, Atg27, and Ksh1. Scale bar- 2 μm. **b** Map of QCDPred and TMHMM predictions of Erp2, Atg27, and Ksh1 degron and TMD probabilities, respectively. A degron cutoff probability is marked at P = 0.85 by a dashed gray line. **c**–**f** Flow cytometry histograms of the normalized yeG/yeC ratio in cells expressing (**c**) C-terminal appended TMDs, (**d**) C-terminal appended SPs, (**e**) N-terminal appended, full length and ΔSP proteins, and (**f)** C-terminal appended ΔSP proteins. Illustrated below each panel are the composition and domain order of the GFP-appended proteins. TM: transmembrane, SP: signal peptide. Stability scale: Median value of the yeG/yeC ratio in empty vector (EV) control was set as one. All other histograms were distributed accordingly. yeG: yeGFP, yeC: yeCherry. Source data for panels **a** and **c–f** are provided as a Source Data file.

Since the QCDPred algorithm is based on data from short peptides and thereby devoid of the cellular context, we wished to empirically assess the putative function of TMDs as potential degrons in a more physiological setting. To this end, three single-pass type-I proteins were arbitrarily selected to examine our hypothesis. These include the COPII-coated vesicles protein Erp2^36^, Atg27, which is involved in autophagy and coated-vesicle transport^37^, and Ksh1, which functions in the early steps of the secretory pathway^38^. When expressed as GFP fusions, all three proteins exhibited membrane localization (Fig. 4a). QCDPred assigned degron function to both the N-terminal signal peptides (SPs) that mediate ER insertion^39^ and the TMDs of the three proteins (Fig. 4b). This was confirmed experimentally by fusing the TM or SP regions of the three proteins C-terminally to GFP (a position that is unlikely to support ER translocation), followed by flow cytometry analysis (Fig. 4c, d). Both TMs and SPs are proteasome substrates, as demonstrated in cells expressing the corresponding plasmids that were treated with BZ (Supplementary Fig. 4a). Thus, besides ER targeting, SPs may additionally function as QCAP degrons if translocation fails.

As the removal of the SP would likely disrupt ER targeting, we hypothesized that this would in turn lead to a rapid turnover of TMD-containing proteins via recognition of TMDs as degrons. This was tested by determining the steady-state levels, with or without the SP, of Erp2, Atg27, and Ksh1 (Fig. 4e, Supplementary Fig. 4b). Appending the full-length proteins N-terminally to GFP, thereby enabeling SP-dependent membrane insertion, resulted in moderate destabilization, assumingly by QCAP, that indeed, was greatly enhanced by the removal of the SP. Curiously, _∆SP_Atg27 did not respond to proteasome inhibition while levels of _∆SP_Erp2 and _∆SP_Ksh1 were significantly increased (Supplementary Fig. 4b), suggesting a proteasome-independent degradation mechanism for the mislocalized autophagy-associated protein. Notably, positioning of the cytosolic exposed TMD degrons is immaterial to their function as both N-terminally- and C-terminally-appended SP-excluded proteins were substantially unstable (Fig. 4e, f).

### QCAP degron characteristics

**Fig. 5:**
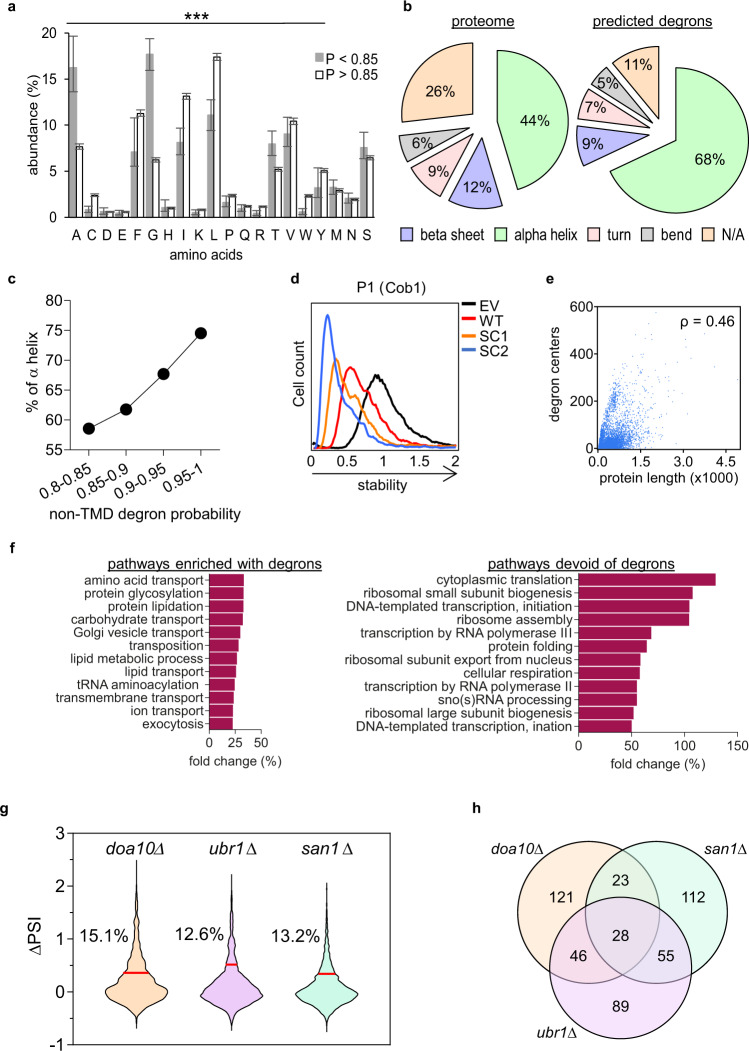
Characterization of QCAP degrons. **a** Comparison of mean amino acid distribution between TMDs in the yeast proteome with high degron probability (P ≥ 0.85; White color; *n* = 1204) to those with lower probability (*P* < 0.85; Gray color; *n* = 107). Data are presented as mean values + /- low and high confidence intervals. Statistical tests were (**a**) Chi-square (*p* = 0.018; DF = 19) that was followed by (**b**) Two sided Mann Whitney U test (***: *p* < 0.001). Amino acid residues serine, methionine and asparagine did not show significant differences between the two groups. **b** Pie chart of the classification and relative proportions of protein secondary structure versus that of QCDPred-predicted degrons within the entire yeast proteome, based on the AlphaFold Protein Structure Database. **c** Plot of the percentile of α helix structures versus QCDPred assigned non-TMD degron probabilities, divided into four equal bins. **d** Flow cytometry histograms of the normalized yeG/yeC ratio in cells expressing P1 peptide emerged from the yeast protein Cob1 and two randomly scrambled variants SC1 and SC2. Stability scale: Median value of the yeG/yeC ratio in empty vector (EV) control was set as one. All other histograms were distributed accordingly. **e** Plot of QCDPred calculated degron centers within the entire yeast proteome, as a function of protein length. ρ: Spearman’s correlation coefficient. *p* < 0.0001. **f** Gene ontology (GO) process annotations of the fold change of top twelve significant pathways (*p* < 0.05) enriched or devoid of degrons, compared to a reference yeast proteome (strain S288C). **g** Violin plot displaying changes in the PSI of 2175 degrons (PSI ≤ 1.7) upon knocking out ORFs of the tested QCAP E3 enzymes. ∆PSI values between degrons in *pdr5∆* strain *and E3∆* strains were calculated. The red line marks two standard errors from the mean for each strain. Degrons above this threshold were considered stabilized by the knockout. The Percentile of stabilized degrons is indicated for each tested QCAP E3. **h** Venn diagram displaying overlapping functions of the tested QCAP E3 enzymes. Sequences of the top 10% ∆PSI values for each knockout strain (218 peptides) were compared. Source data for panels **a, d** and **e** are provided as a Source Data file.

The data so far indicate that in the yeast proteome QCAP degrons are widespread and that these regions are prevalent in hydrophobic residues while negatively charged residues are depleted. Nevertheless, we found that within hydrophobic degrons there is a bias toward specific residues (Fig. 5a): Comparing the distribution of amino acids in hydrophobic TMDs with high degron probability (*P* ≥ 0.85) to a small set of TMDs with low degron probability (*P* < 0.85), we found that the former is greatly enriched in bulky and branched hydrophobic amino acids, while the latter express small, non-polar, amino acids instead. In addition, we identified a prevalence of alpha-helical configurations in QCAP degrons in their native protein context (Fig. 5b). This not only agrees with the established helical structure of TMDs but also correlates strongly with an increase in the probability of non-TMD degrons (Fig. 5c). Interestingly, QCAP degrons were hardly found in N/A (not assigned) regions that are likely intrinsically disordered (Fig. 5b). This implies that for most QCAP degrons to become active, the protein must be structurally perturbed so that a degron is exposed. Indeed, we have found that for disordered proteins and regions there is a correlation between the presence of predicted degrons and the abundance and half-lives of the proteins^23^. Altogether, our data indicate that QCAP degrons are enriched in bulky hydrophobic TMD-like entities.

We next examined the role of the linear order of amino acids within a degron on its function. To this end, the amino acid sequence of a peptide P1 from the yeast protein Cob1 of the b-c1 complex was scrambled to form two random peptides having identical amino acid content, hence also identical degron probability when predicted by QCDPred (Table 1). When fused to the C-terminus of GFP, all three peptides were predicted to form helices, however, to various degrees (Supplementary Fig. 5). Both scrambled peptides not only conferred GFP degradation but were even more potent than the original P1 peptide, indicating that the general chemical properties of the degron, rather than its exact linear sequence is a principal QCAP degron determinant (Fig. 5d). The results, however, also show that at least in this case, the patterning and sequence can play a modulatory role in the degron strength.

### Correlation between proteins function and the presence of QCAP degrons

We next searched for roles governing the distribution of degrons in the proteome. Specifically, whether it is simply dependent on the probability of finding a defined sequence within the entire proteome or associated with specific protein property or function. As shown in Fig.5e, a weak monotonic relation was observed between protein length and degron presence (Spearman rank correlation coefficient (ρ) of 0.46), thus excluding a random distribution of the QCAP degronome. We then analyzed degrons through Gene Ontology (GO) annotation (Fig. 5f). To this end, the yeast proteome was classified, based on QCDPred score into two groups: with or without degrons at high significance (*P* ≥ or <0.85). Each group was then used as input for determining GO processes using the Saccharomyces Genome Database (SGD) Gene Ontology Slim Term Mapper (https://www.yeastgenome.org/goSlimMapper). Data in Fig. 5f and Supplementary Fig. 6 show that proteins engaged in transport and lipid metabolism are enriched in QCAP degrons. Many of these protein classes are integrated into membranes, an observation that agrees with our finding that TMDs can also act as QCAP degrons when exposed to the PQC system. Conversely, proteins, where QCAP degrons are underrepresented, are mostly involved in transcription, translation, ribosome assembly, as well as protein folding. The latter group is of particular interest as it implies that the exclusion of QCAP degrons in chaperones involved in proteolysis renders them resistant to degradation themselves. QCDPred analysis of a collection of cytosolic/nuclear yeast chaperones predicted one or more degrons in Hsp90, Hsp104, and Hsp110 family members, while Hsp40 and Hsp70 family members, that are directly involved in proteolysis^2^, were mostly devoid of QCAP degrons (Supplementary Fig. 7).

### Partial selectivity of QCAP E3 ligases

To learn about UPS-dependent QCAP functionality, we next examined degron specificity of ubiquitin-protein E3 ligases. To this end, the aforementioned peptide library was inserted into yeast strains lacking one of three well-defined QCAP E3 ligases: Doa10, which is a multi-TMDs E3 ligase that localizes to the outer leaflet of the ER membrane and the nuclear envelope^40^, Ubr1, which is a soluble protein residing in the cytoplasm and the nucleoplasm^41^, and San1, which is exclusively nuclear E3^42^. yGPS-P_lib_ transformation into E3 deleted strains (*E3∆*) was followed by FACS and NGS, and PSI scores of degrons having *P* < 1.7 were determined and compared to that of *pdr5∆* cells that served as a control strain (Supplementary Data 2). All knockout groups displayed a significant increase in degrons PSI scores (Kruskal-Wallis test *p* < 0.001). A violin plot of the change in PSI score (ΔPSI = PSI_E3∆_-PSI_WT_) indicates that Doa10 substrates are the largest group of degrons in the tested peptidome (Fig. 5g). We note, however, that because the PSI scale is effectively determined by the distribution of degron potential within an individual experiment, it is difficult to interpret ΔPSI scores on an absolute scale. A Venn diagram determining E3 functional overlaps indicates that approximately one-third of degrons were recognized by two or more E3 enzymes (Fig. 5h). This finding is consistent with that of Hickey and colleagues who showed distinct yet overlapping QCAP E3 ligase substrate specificity governed by E3 subcellular localization^18^.

### Constraints governing degron potency are evolutionarily conserved

**Fig. 6:**
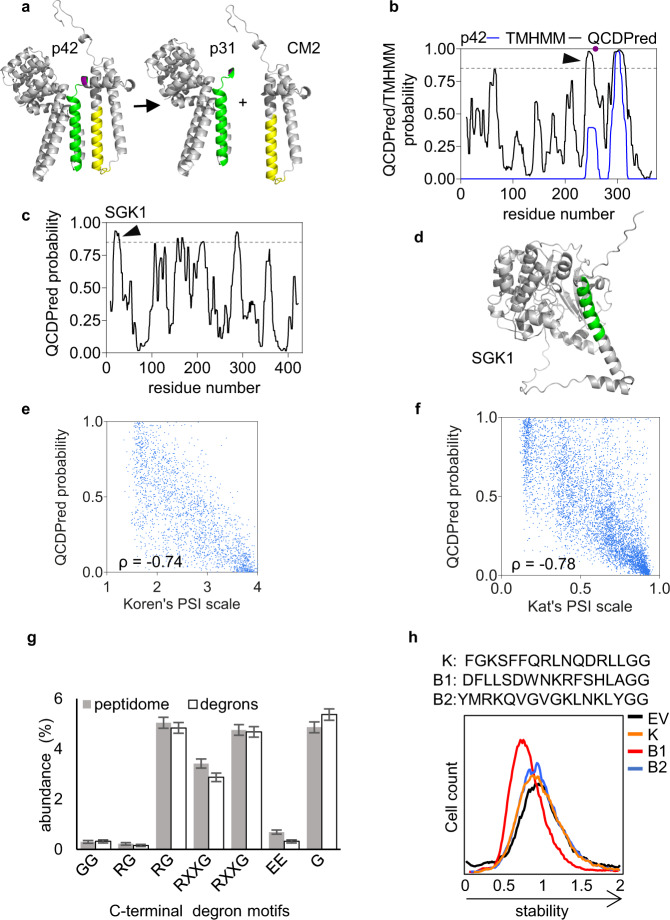
Degron projections in non-yeast organisms. **a** Assignment of QCDPred projected degrons into the influenza C virus Polyprotein p42 trRosetta-predicted 3D structure (Template modeling score: 0.642). Shown are the schematics of p42 cleavage at an internal SP site (marked in purple) into p31 and CM2. **b** QCDPred and TMHMM probabilities of p42 degrons. Arrowhead indicates the position of the postulated p31 degron. A degron cutoff probability is marked at P = 0.85 by a dashed gray line. **c** QCDPred-calculated degron probabilities within SGK1. Arrowhead indicates the position of the postulated SGK1 degron. A degron cutoff probability is marked at P = 0.85 by a dashed gray line. **d** Assignment of a QCDPred-identified QCAP degron (colored green) into the AlphFold-predicted SGK1 structure (#AF-O00141-F1). **e** Scatter plot comparing PSI values of 23-mer C-terminal peptides of the human proteome^22^ and averaged QCDPred assessment of the same fragments. Prediction scores were averaged between 7 different amino acid centers within the 23 amino acids length of the original peptides. Shown are values of random 25% peptides from Koren’s screen. ρ: Spearman’s correlation coefficient. *p* < 0.0001. *n* = 22,564. **f** Scatter plot comparing PSI values of 15-mer N-terminal peptides of the yeast *S. cerevisiae* proteome^45^ and QCDPred assessment of the same fragments. ρ: Spearman’s correlation coefficient. *p* < 0.0001. *n* = 6,370. **g** Comparison of the mean distribution of the six principal C-terminal degradation motifs identified by Koren et al.^22^, between the entire tested peptidome (White color; *N* = 23,600) and calculated degrons within the tested peptidome (PSI ≤ 1.62; Gray color; *N* = 1882). Data are presented as mean values + /- SD. Statistical test Chi-square did no show significant differences in motif abundance between the two groups (*p* = 0.99; DF = 6). **h** Flow cytometry histograms of the normalized yeG/yeC ratio in cells expressing fused peptides with a GG end: K- a C-terminal degron identified by Koren et al.^22^, and B1-2 from the current screen. Stability scale: Median value of the yeG/yeC ratio in empty vector (EV) control was set as one. Source data for panels **e-h** are provided as a Source Data file.

Considering the high conservation of QCAP pathways in the evolution of all eukaryotes, we hypothesized that QCAP degron properties are similarly well preserved. To test this paradigm, we next investigated how well the yeast-based QCDPred algorithm predicts the presence of QCAP degrons in other organisms and selected the influenza C virus p42 protein and human serum and glucocorticoid-inducible kinase 1 (SGK1) as test cases. The influenza p42 contains a signal peptidase site at residue 259 that upon cleavage yields the p31 and CM2 proteins (Fig. 6a). CM2 integrates into the ER membrane through a single TMD, while p31 is rapidly degraded by the UPS via a degron at the C-terminal region^43^. As anticipated, both the C-terminal region of p31 and the TMD of p42 were predicted by QCDPred to function as degrons (Fig. 6b), however, during viral infection only the C-terminal region of p31 likely functions as a degron because it is accessible to the degradation system. Furthermore, Arteaga and co-workers have previously demonstrated that an amphipathic helix at the N-terminus of SGK1 targets the protein for proteolysis^44^. Indeed, QCDPred analysis of SGK1 revealed three potential degrons, the strongest of which is placed between amino acids 17 and 29, the same region that was previously identified as a QCAP degron^44^ (Fig. 6c, d).

Having demonstrated that the tested non-yeast QCAP degrons can be predicted by QCDPred, we next wanted to investigate the universality of QCAP degrons by testing whether the principles established for yeast degrons generally apply to the human degronome. To this end, we assessed the correlation between PSI values, previously assigned by Koren et al. for C-terminal regions of the entire human proteome^22^, and their average QCDPred probabilities. The comparison yielded a high correlation (ρ = −0.74) (Fig. 6e), similar to that observed by Kats et al., for the yeast N-terminome (ρ = −0.78)^45^ (Fig. 6f), indicating that indeed principles of QCAP degron features are evolutionarily conserved. Consequently, average QCDPred scores were assigned to each amino acid in both the yeast and human proteomes (Supplementary Data 4, 5) and we also provided QCDPred as a web-server tool to predict degrons^23^.

Koren et al. have shown that C-terminal glycine residues are underrepresented in the eukaryotic proteome and proposed that the depletion of glycine at the C termini of eukaryotic proteins is a result of avoidance of E3s targeting glycine-end degrons^22^. Glycine residues are equally underrepresented in *S. cerevisiae*, which implies a similar role in degradation (Supplementary Fig. 8). To examine the possible role of glycines and other C-terminal degradation motifs described by Koren et al. in *S. cerevisiae*, we analyzed their abundance at the C-terminus of high confident degrons within the tested degronome (PSI ≤ 1.62) and compare it to that of the entire peptidome. Surprisingly, no significant difference between the two groups was observed (Fig. 6g), suggesting that, unlike in humans, C-terminal degrons do not play a substantial role in determining the half-lives of *S. cerevisiae* proteins. In agreement with these findings, neither a degron peptide from the Koren screen (K) nor re-cloned peptides from the peptidome, having two glycine residues at their C-termini, conferred GFP destabilization in yGPS-P vector (Fig. 6h).

## Discussion

A major barrier to unearthing QCAP degrons has been their unconformity. Therefore, authentic degron discovery largely relied on the screening of peptide libraries in search of sequences that induce the degradation of otherwise stable proteins and then, trying to deduce consensus sequence requirements^16^. Indeed, these efforts led to the discovery of many artificial and physiological degrons but were still far from distinguishing common degradation motifs in QCAP. A breakthrough in degron discovery has been provided by the development of the pioneering GPS-peptidome methods to study protein degradation and identify degron motifs^22^. By implementing GPS-P in yeast we have here identified a large cohort of authentic degrons that was subsequently used to train a degron prediction algorithm^23^. This large group of peptides that determine a variety of half-lives, provide consensus degron features that are compatible with TMD properties. Further functional validation established TM-like degron features as key determinants of QCAP and possibly of the proteome half-life.

The discovery that E3 ligases selectively bind substrates through recognition of distinct determinants (degrons) established their role as substrate recognition modules of the UPS^46^. Consequently, the identification of degrons has become one of the focal points of UPS research. Initially, the hunt for degrons identified mostly acquired determinants that are, for example, the result of transient post-translational modifications such as phosphorylation that induce timely, regulated degradation via dedicated E3 ligases^47^. Obviously, acquired degrons do not account for the majority of QCAP, carried out by specialized E3 ligase systems that monitor protein folding state, presumably recognizing internal sequences that may become exposed following conformational aberrations. This assertion was confirmed during the characterization of *Deg1* degron of the Doa10 E3 ligase^48^. *Deg1* is masked in the Mata1/Matα2 mating-type dimer and exposed upon complex dissociation^49^. Critical for *Deg1* degron function is an amphipathic helix^49^, also present in other synthetic and authentic Doa10 substrates^25,26,44,50^. Despite hydrophobicity constituting a primary Doa10-recognition determinant^18^, not all QCAP degrons conform to the same consensus^19^ (see a comparison in Supplementary Data 6). Overall, the initial degron discoveries, while gaining important insights into degron complexities, could not account for the entire QCAP.

Here we show that a large portion of QCAP degrons shares similar characteristics with *Deg1*. These degradation elements are conserved in evolution, likely playing distinct roles during a protein’s lifetime. QCAP degrons probably play a pivotal role in determining complications in cotranslational protein folding. They may also dictate problems in the early steps of protein complexes formation by monitoring the cotranslational assembly of nascent chains with fully synthesized co-partners^51^. Indeed, a large fraction of the newly synthesized proteome was shown to undergo co-translational proteolysis^52^. Once proteins progress into their mature structure, they can populate ensembles of conformations, some of which are unfunctional. The exposure of QCAP degrons may further support the enrichment of advanced conformations by continuously eliminating unwanted protein populations. This triage mechanism is predicted to intensify under stress. QCAP degrons also contribute to the elimination of protein subunits during the disassembly of temporary protein complexes^29,30^. In all these scenarios, QCAP counteracts unfolded protein’s propensity for aggregation, signifying its importance to the viability and survival of all eukaryotes.

Hydrophobic stretches are considered key determinants of QCAP in all eukaryotes, from yeast to mammals^17,18^. Our observations that QCAP degrons are to a large extent determined by amino acid composition implies a broad degron recognition mechanism. The prevalence of QCAP degrons function in yeast was previously demonstrated by overlapping degron recognition by E3 ligases (Fig. 5g and^17,18^). As protein structure is dynamic, QCAP sensing of protein folding state via the exposure of hydrophobic, TM-like regions and their nonselective E3 ligase recognition can now provide a plausible mechanism for regulation of the proteome stability. As E3 ligases are rate-limiting for ubiquitin-dependent degradation, recognition of exposed hydrophobic stretches, either directly or indirectly, by multiple E3 ligases may increase cellular degradation capacity in response to diverse stress conditions where aberrant protein overload might lead to proteotoxicity^53^.

That TMDs can operate as degrons is not surprising as membrane-embedded sequences within integral ER-membrane proteins have already been shown to display a degron function. These include the C-terminal TMD of the T-cell receptor α subunit (TCR α)^54^ as well as other lone proteins that are normally part of TM protein complexes^55,56^. Single and homomeric ER-embedded proteins, such as the E2 enzyme Ubc6 and the C-terminal TMDs of yeast and human HMG CoA reductase can similarly undergo QCAP via their TMDs that act as degrons^57–59^. By demonstrating that single-pass TM proteins devoid of their SPs were rapidly degraded by the UPS (Fig. 4e, f), we have expanded this view by establishing TMDs as conserved QCAP degrons of non-integrated TM proteins.

Our findings that TMDs can act as degrons are compatible with a pre-insertion degradation mechanism, operating at the cytosolic side of the ER membrane, that subjects Signal Recognition Particle (SRP)-independent substrates of the glycosylphosphatidylinositol (GPI) anchored proteins to QCAP^60^. It is also compatible with SRP-independent insertion mechanisms of Atg27 and Ksh1^61^ that eliminate subpopulations that may evade the secretory pathway (Fig. 4f). Moreover, in mammals, BAG6 and its associated protein UBQLN4 were shown to recognize the exposed hydrophobicity of TMDs of proteins that evade the secretory pathway and trigger their proteasomal degradation^62,63^. Thus, exposure of both the SP and TMDs may ensure that suboptimal or complete failure of ER membrane integration would result in QCAP. Failure to degrade mislocalized membrane proteins may result in cytotoxicity, due to enhanced formation of insoluble intracellular bodies or aberrant insertion in the mitochondria membrane^61^. Our observation that SP-devoid integral membrane proteins are subjected to rapid degradation (Fig. 4e, f) is fully compatible with this assumption.

Molecular chaperones that discern misfolded proteins also participate in misfolded protein degradation^2,64^. Auxiliary PQC chaperone functions include substrate solubilization, mediating E3 binding, as well as delivery of ubiquitinated proteins to the proteasome. The prevailing view of chaperone function in QCAP asserts that Hsp70s initially recognize misfolded substrates and deliver them to an E3 ligase, a function facilitated by Hsp40s and nuclear exchange factors that catalyze ATP hydrolysis and ADP exchange, respectively^64^. While a role for Hsp70s/Hsp40s in misfolded substrates targeting the human E3 ligase, the carboxy terminus of Hsc70 interacting protein (CHIP), is well established^65^, whether Hsp70s/Hsp40s similarly mediate the recognition of QCAP degrons by their cognate E3s remains to be determined.

Surprisingly, despite our observation that glycine residues are underrepresented at the C-termini of the *S. cerevisiae* proteome, sequence-specific C-terminal degrons do not seem to play a principal role in yeast protein turnover determination. One explanation for the discrepancy between the human and yeast proteomes is that yeast does not encompass Cullin Ring Ligases (CRLs) that recognize C-terminal degrons. Indeed, the principal CRLs that take part in C-terminal degron recognition, namely Cul2 and Cul4 family members, are absent in *S. cerevisiae* and the relevant F-boxes are also missing. Alternatively, C-terminal yeast degrons may be recognized by other E3 ligases with more complex specificity. Perhaps the role of C-terminal glycines in fungal protein degradation is more context-dependent, e.g., that it requires other, more distant degron elements that are not present in the 17-mer peptides. However, further studies of the stability of yeast proteome are required in order to identify and characterize these speculative distal elements.

## Methods

### Plasmids, yeast strains, and antibodies

Antibodies, yeast strains, and plasmids used in this study are listed in Supplementary Tables 2, 3, and 4, respectively.

### Parental plasmid for yGPS-P screen

Plasmid pGADT7-ADH700-yeCherry-p150-yeGFP-DHFR was obtained from Addgene (#24378)^24^ and was used as a template for PCR cloning of ADH700-yeCherry-P150-yeGFP into pTR1412^15^ at NotI and XmaI restriction sites. A 5-mer linker was added downstream to GFP to create the parental plasmid pTR1861 for yGPS-P screen (Fig. 1a). pTR2089, the parental plasmid for yGPS-P N-terminal cloning, was constructed by overlap extension PCR^66^, producing a fragment containing PacI and BamHI restriction sites, that was placed upstream to GFP in pTR1861 by ligation.

### Cloning and mutagenesis

Plasmids used in this study are listed in Supplementary Table 4. DNA fragments encoding the full-length, ∆SP, TMD-only, and SP-only versions of Erp2, Atg27, and Ksh1 were amplified from the genomic DNA of *wild-type* yeast strain BY4741. The PCR fragments were subjected to digestion with restriction enzymes XmaI and NheI (for C-terminal cloning) or with PacI and BamHI (for N-terminal cloning). The resulting fragments were subcloned into pTR1861 or pTR2089, respectively.

Oligos insertions into pTR1861 were done by heating a single DNA pair containing the wanted insertion (see “Supplementary Table 5” for details) and flanking sequences responsible for overhangs compatible with XmaI and NheI restriction sites and cooling down slowly to create double-stranded fragments. The double-strand DNA was ligated directly into pTR1861 and digested by the same restriction enzymes. Mutagenesis of peptide P3 and Pca1 was conducted using QuikChange Lightning Site-Directed Mutagenesis Kit, according to the manufacturer’s instructions (Agilent). All products were verified by sequencing.

### Generation of a peptide library (yGPS-P_lib_)

Three hundred twenty six proteins, corresponding to 23 yeast complexes were first encoded as DNA bases using the Saccharomyces Genome Database (SGD) website. Then, DNA corresponding to the open reading frame of each protein was divided into 51 bp (17-mer) fragments with 36 bp (12-mer) overlaps between neighboring oligonucleotides (tiling). The fragments also contained two flanking 12 bp primers that match the vector sequence to enable Gibson assembly. The corresponding oligonucleotides were synthesized by LC Sciences (Houston, TX), amplified by PCR, and cloned by Gibson assembly master mix kit (New England Biolabs) into pTR1861 at XmaI and NheI restriction sites, followed by transformation into electro-competent DH10B bacterial cells. Approximately two million colonies were scraped from plates and pooled and plasmid DNA was purified using PureLink® HiPure plasmid filter Midiprep kit (Invitrogen). The resulting plasmid library was transformed into TRy1392 (*pdr5∆*) yeast strain, followed by selection on leucine-deficient media (SD-Leu). Surviving cells were scraped, pooled, and frozen immediately in 25% glycerol at −80 °C.

### Generation of a degron library (yGPS-Pdeg)

Yeast cells expressing yGPS-P_lib_ were grown O/N on SD-Leu media to a mid-log phase. Cells were subjected to BD FACS Aria III instrument using 488 nm and 561 nm lasers for capturing the fluorescence emission of GFP and Cherry, respectively. One million cells having the lowest 10% yeG/yeC ratio, representing cells harboring unstable GFP, were separated (Supplementary Fig. 1c). Sorted cells were incubated O/N in SD-Leu media, then divided into aliquots and frozen in 25% glycerol at −80 °C.

### Degron screen

Cells expressing yGPS-P_lib_ were grown to mid-log-phase and sorted by FACS BD-Facsaria III into four equal gates, each containing 2.5 million yeast cells, based on their yeG/yeC ratio (Fig. 2a). Plasmids from each gate were purified using Zymoprep yeast Plasmid Miniprep II (Zymo Research).

### Preparation of plasmid DNA for NGS

DNA sequencing by NGS consisted of two PCR amplification steps with KAPA HiFi HotStart ReadyMix PCR Kit (Roche). The first step (18 cycles) was performed using primers flanking the peptides, with overhangs complementary to Illumina adapters (primers NGS-F, NGS-R). The second step was performed using standard N-series Illumina barcoded adapters (12 cycles). Sequences were size-selected using SPRI beads for NGS. Samples were subsequently pooled, purified on an agarose gel, and sequenced on an Illumina NextSeq 500 machine.

### Data preprocessing

Sequencing data were processed using a custom pipeline written for the R project for statistical computing (https://www.R-project.org). Reads were aligned to the expected oligo database with bowtie2^67^. Sequences corresponding to the tiled peptides were counted and assigned to the different strains based on forward and reverse barcodes.

Protein stability indexes (PSIs) were calculated according to Yen et al.^21^ Briefly, the frequency, *f*_*ig*_ of peptide *i* in gate *g*, was multiplied by gate number (1–4) and summed up, yielding a stability score between 1 (maximally unstable) and 4 (maximally stable)

1 $${{{{{{\rm{PSI}}}}}}}_{{{{{{\rm{i}}}}}}}=\frac{{\sum }_{g}g{\times f}_{{ig}}}{{\sum }_{g}{f}_{{ig}}}$$

### Flow cytometry

Yeast cells were grown to mid-log-phase on SD-Leu media and analyzed on a CellStream analyzer instrument (Merck) using 488 nm and 561 nm lasers for capturing the fluorescence emission of GFP and Cherry, respectively. For each condition, 10,000 events were analyzed and presented on a histogram using FlowJo software V10.8.1 (BD Biosciences). Experiments were repeated two or more times.

### Machine learning

A detailed explanation of the QCDPred model is described by Johansson et al.^23^ Briefly, a machine-learning model based on logistic regression was trained using 18,599 peptides (peptides with more than 50 sequencing reads across the bins). These peptides were classified as degrons if their PSI < 2.2 and as non-degrons if their PSI > 2.8. Peptides’ amino acid composition was fed into QCDPred, which outputs a probability score for each amino acid.

### Immunoblotting

Cells were grown to mid-log-phase with or without the following reagents: Bortezomib (BZ; A2S Cat # 2614), cycloheximide (CHX, Sigma, Cat # C6255) or cadmium (Cd; Sigma Cat # 265365). Cells were pelleted by centrifugation (3,500 × g, 5 min) and incubated with 0.1 N NaOH for 5 min, followed by centrifugation (17,000 × g, 3 min). SDS-PAGE sample buffer containing 50 mg/ml Dithiothreitol was next added, followed by boiling for 5 min. Proteins were separated on SDS-PAGE, transferred to a PVDF membrane, blocked in 10% Dry Milk in TBS + 0.1% Tween-20 (TBS-T), and then probed with primary antibodies for 1 h at room temperature. Following three washes with TBS-T, the membrane was incubated with a secondary antibody for 0.5 h at room temperature, then washed three times with TBS-T. Membranes were incubated with ECL mix (Thermo Fisher Scientific) for 2 min and reactive bands were visualized using Fusion Pulse (Vilber Lourmat).

### Fluorescence microscopy

Imaging was performed with Olympus IX71 inverted microscope with an x 60 oil objective lens. Fluorescence was excited with 576 nm for Cherry and with 488 nm for GFP. Imaging data were handled using ImageJ V1.53t.

### Proteome databases

All proteome databases were downloaded from the UniProt database server (https://www.uniprot.org) as FASTA files. These files contain the full-length protein sequence.

### PDB/CIF data

AlphaFold-2-based PDB and CIF files for single proteins were extracted from the European Bioinformatics Institute website (https://alphafold.ebi.ac.uk/). These files were used for creating a 3D protein database. Most PDB and CIF files contain information on secondary structure patterns. Each amino acid in these models was assigned a secondary structure indicator and proteome statistics were inferred. PDB files were visualized using PyMOL software V2.5.4.

### Transmembrane protein data

Transmembrane protein data was extracted from the TM Helix Hidden Markov Model (TMHMM) algorithm^68^, implemented with the python package tmhmm.py. To compute the intersection between TMDs and degrons, each amino acid within the entire yeast proteome was evaluated for QCDPred value and TMD classification. Amino acids with QCDPred probability ≥ 0.85 that were classified as TMDs according to TMHMM were added to the intersection group.

### Reporting summary

Further information on research design is available in the Nature Portfolio Reporting Summary linked to this article.

## Supplementary information

Supplementary Information

Description of Additional Supplementary Files

Supplementary Data 1

Supplementary Data 2

Supplementary Data 3

Supplementary Data 4

Supplementary Data 5

Supplementary Data 6

Reporting Summary

## Data Availability

The data supporting the findings of this study, including experimental procedures and compound characterization, are available within the article and its Supplementary Information files, or from the corresponding author upon request. Plasmid pTR2089 has been deposited with the corresponding sequence at Addgene. QCDPred analyses of yeast and human proteomes are available in Supplementary Data 4, 5, respectively. QCDPred analyses of other proteins of interest are available on a web server described by Johansson et al^23^. PDB entry 6T0B was used in the course of this study. Source data are provided with this paper.

## Source data

Source Data

## Code availability

Custom code used to process and analyze peptide library data is available at: https://github.com/KULL-Centre/_2022_Mashahreh_degronome^69^.
