# Supplementary material for: Conserved degronome features governing quality control associated proteolysis
Source: Nat Commun. 2022 Dec 8;13:7588. doi: 10.1038/s41467-022-35298-y (PMC9732359; doi:10.1038/s41467-022-35298-y)
Supplement: Supplementary file 1 — Supplementary Information [file 41467_2022_35298_MOESM1_ESM.pdf]

Supplementary Information

Conserved degronome features governing quality control associated proteolysis

Bayan Mashahreh[1], Shir Armony[1], Kristoffer Enøe Johansson[2], Alon Chappleboim[1], Nir Friedman[1], Richard G. Gardner[3], Rasmus Hartmann-Petersen[2], Kresten Lindorff-Larsen[2], Tommer Ravid[1*]

[1]Department of Biological Chemistry, The Alexander Silberman Institute of Life Sciences, The Hebrew University of Jerusalem, Jerusalem, Israel

[2]The Linderstrøm-Lang Centre for Protein Science, Department of Biology, University of Copenhagen, Copenhagen, Denmark

[3]Department of Pharmacology, University of Washington, Seattle, WA 98195, USA

*Correspondence: tommer.ravid@mail.huji.ac.il

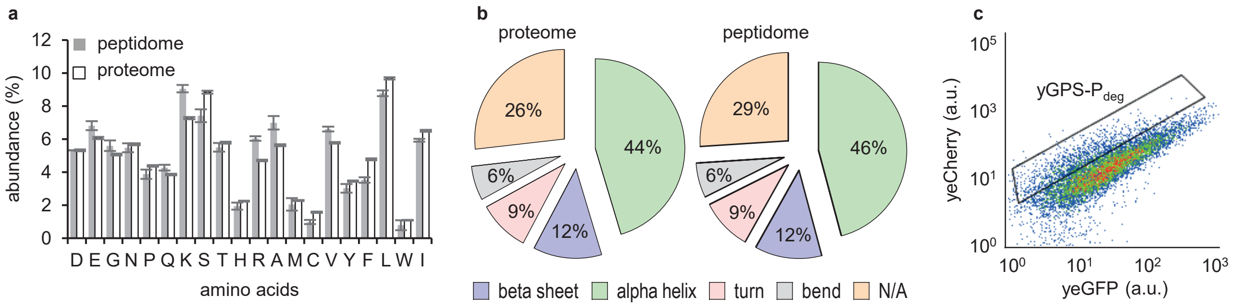

Supplementary Figure 1. Characteristics of the tested peptidome and isolation of degrons. **a** Amino acid composition of the tested peptidome (Gray color; N=323) compared to that of the proteome (White color; N = 6616). Data are presented as mean values +/- low and high confidence intervals. Statistical test Chi-square goodness of fit did no show significant differences in amino acid distribution between the two groups (p =0.99; DF = 19) **b** Pie chart of the classification and relative proportions of protein secondary structure within the yeast proteome versus that of the peptidome, based on the AlphaFold Protein Structure Database. **c** Isolation of top 10% degrons. Yeast cells expressing yGPS-P$_{lib}$ Were grown to mid-log-phase, followed by FACS of the top 10% degron population. One million cells comprising the degron library (yGPS-P$_{deg}$) were collected, then grown O/N and frozen in liquid nitrogen or taken for further analysis. Rectangle: gating of the top 10% degron population out of a total of 10,000 cells. a.u.: arbitrary units. Source data for panels a and c are provided as a Source Data file.

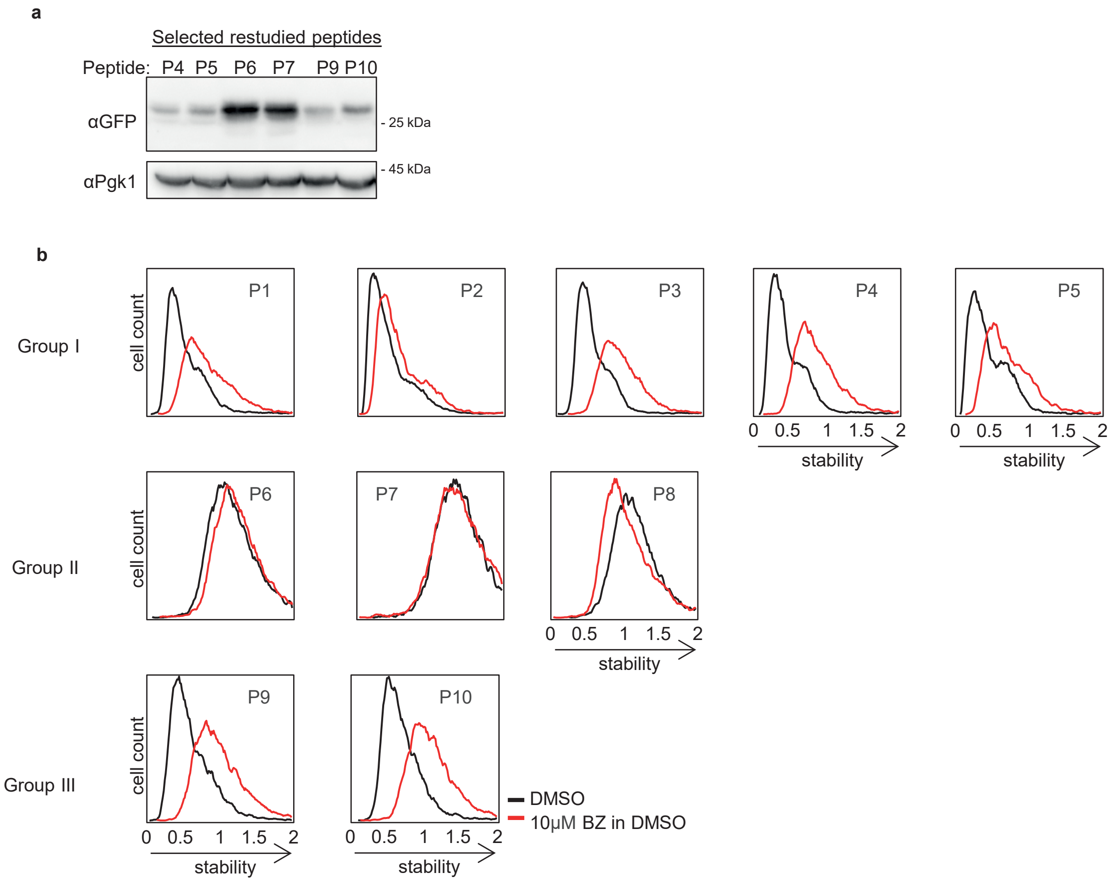

Supplementary Figure 2. Determination of the steady-state levels of selected peptides. a Two peptides from each QCDPred probability group were selected for immunoblot analysis. Cells expressing the corresponding peptides were grown to log phase and lysed, followed by separation on 10% SDS-PAGE and transfer into PVDF membrane. Levels of the GFP-fused peptides were determined by immunoblotting with anti-GFP Abs. This analysis was repeated three times. **b** Cells expressing the related fusion peptides were incubated with DMSO (vehicle) or with 10μM Bortezomib (BZ) for four hours. The increase in relative stability in group-I and group-III peptides indicates proteasome-dependent degradation. No significant effect of BZ on Group-II peptides was observed. Scale: The median value of the yeG/yeC ratio in empty vector (EV) control was set as one. All other histograms were distributed accordingly. Source data are provided as a Source Data file.

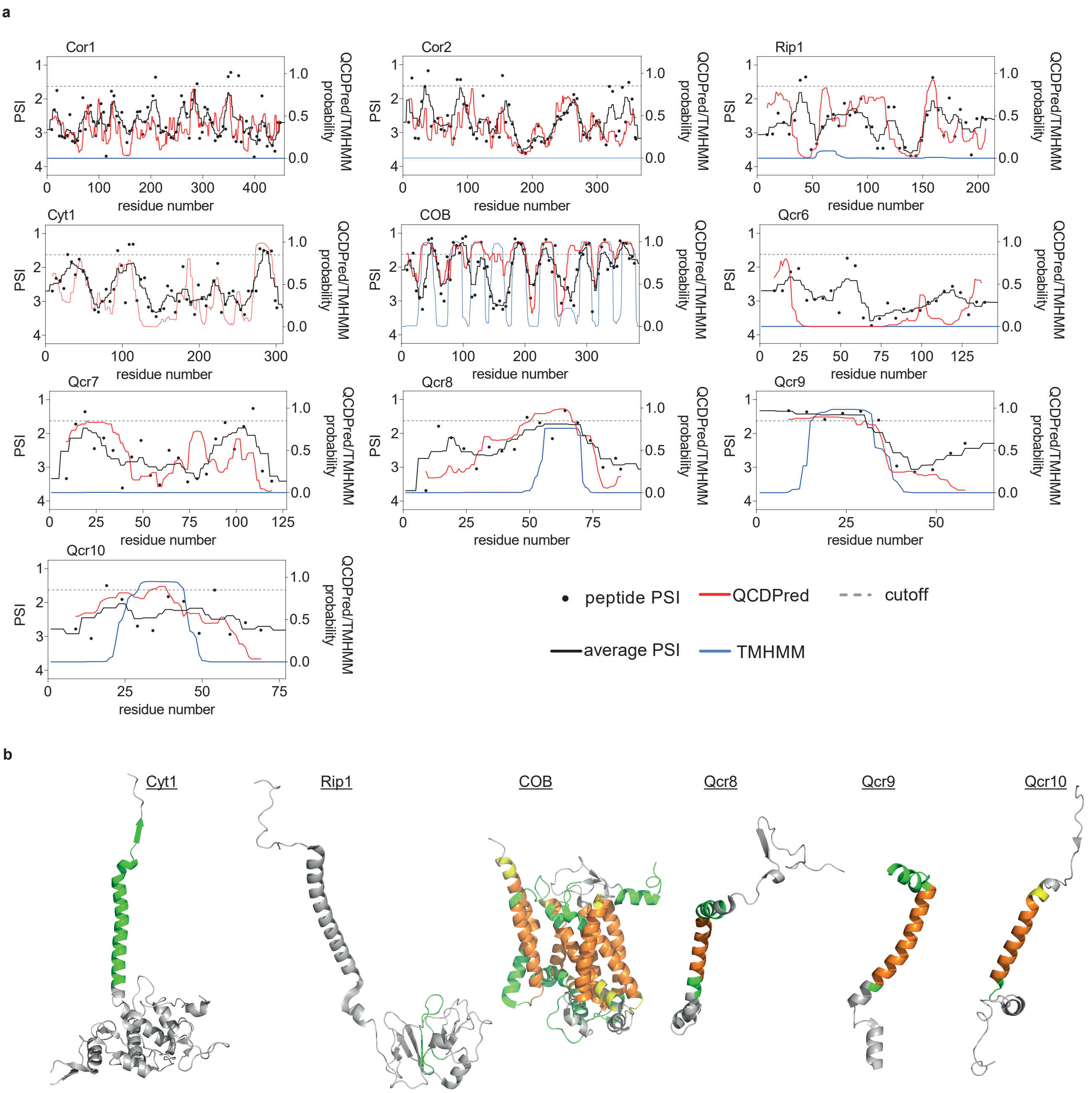

Supplementary Figure 3. Characterization of degrons in the b-c1 complex. a PSI values emerged from experimental data (black dots), average PSI values for each amino acid (colored in black), QCDPred probabilities (colored in red), and TMHMM prediction (colored in light blue) were plotted for each subunit of the b-c1 complex. A degron cutoff probability is marked at P = 0.85 by a dashed grey line. **b** Assignment of QCDPred projected Degrons (green) and TMHMM predicted TMDs (yellow) to TM proteins in the b-c1 3D structure (PDB #6t0b).[1] Orange color signifies overlying regions.

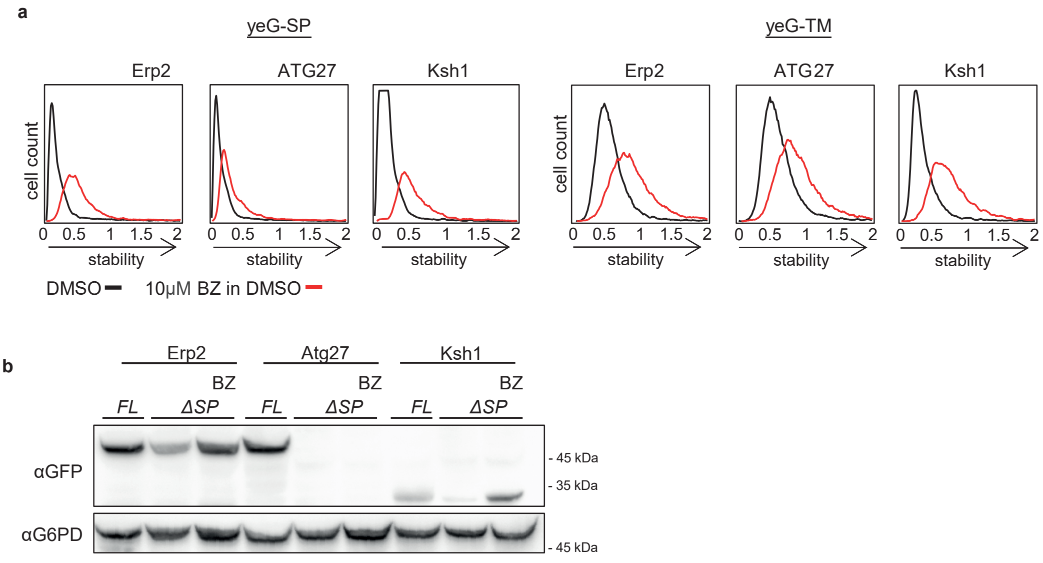

Supplementary Figure 4. Effect of proteasome inhibition on TM proteins steady-state levels. a Cells expressing the indicated fusion peptides were incubated with DMSO (vehicle) or with 10µM Bortezomib (BZ) for four hours. Scale: The median value of the yeG/yeC ratio in empty vector (EV) control was set as one. All other histograms were distributed accordingly. The increase in normalized yeG/yeC indicates proteasome-dependent degradation. **b** Immunoblot analysis of Erp2, Atg27, and Ksh1, with or without their SPs. Where indicated, 10 µM BZ was added to cells for 4 hrs. before cell harvesting. This analysis was repeated two times FL- full-length proteins. ∆SP- proteins devoid of the SP. G6PD: Glucose-6-phosphate dehydrogenase. Source data are provided as a Source Data file.

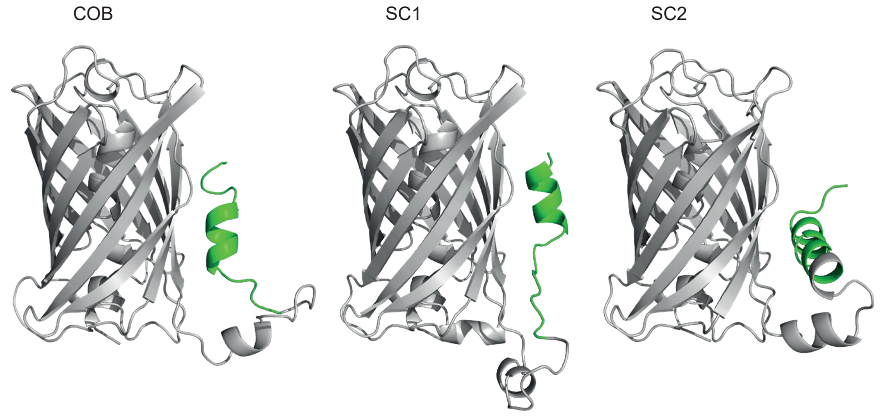

Supplementary Figure 5. Structure prediction of GFP-fused peptides using trRosseta. Estimated Template Modeling scores: COB -0.856, SC1 -0.865, SC2 -0.828. GFP is colored gray and peptides are colored green.

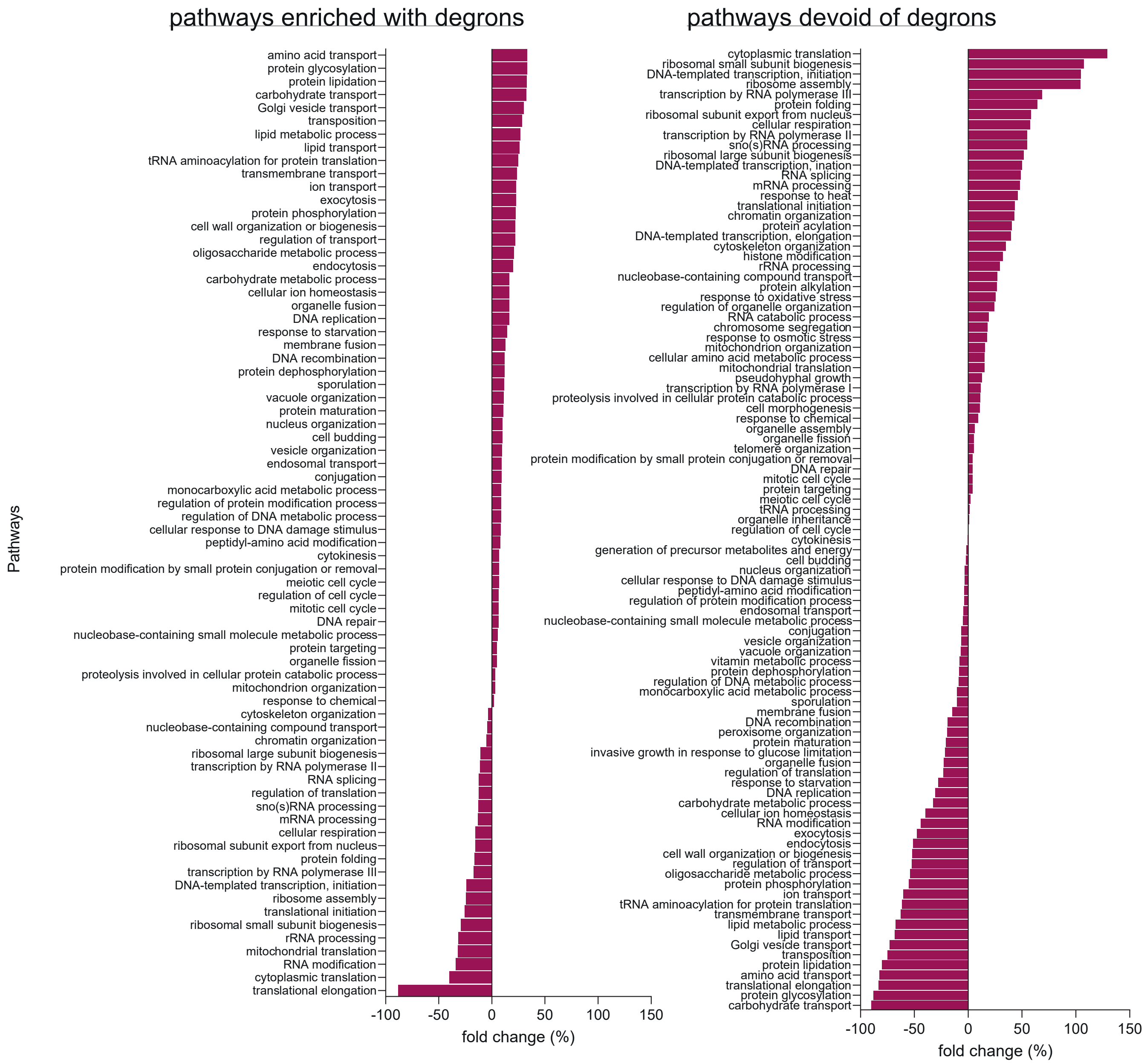

Supplementary Figure 6. Large-scale GO annotations of all significant processes (p-value < 0.05) that are either enriched or devoid of predicted degrons at P ≥ 0.85.

Cytosolic/nuclear Hsp40/70 chaperones

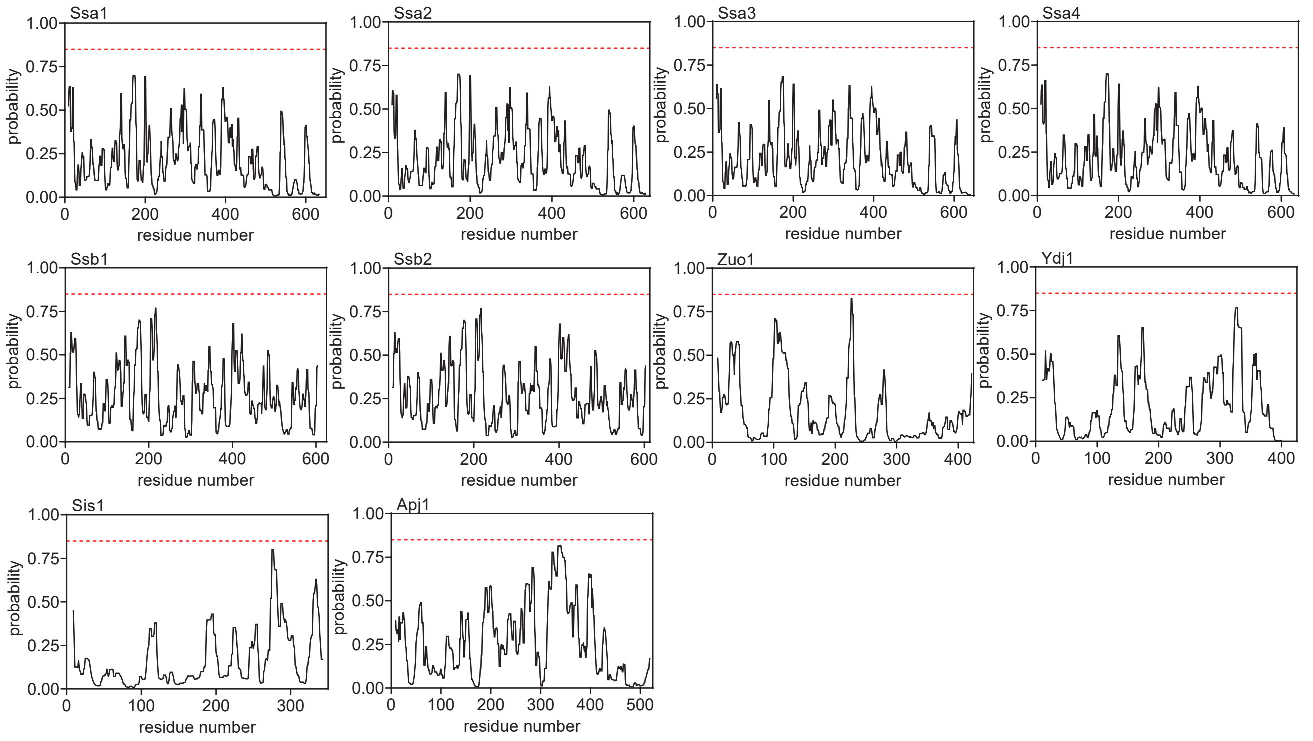

Cytosolic/nuclear Hsp90/100/110 chaperones

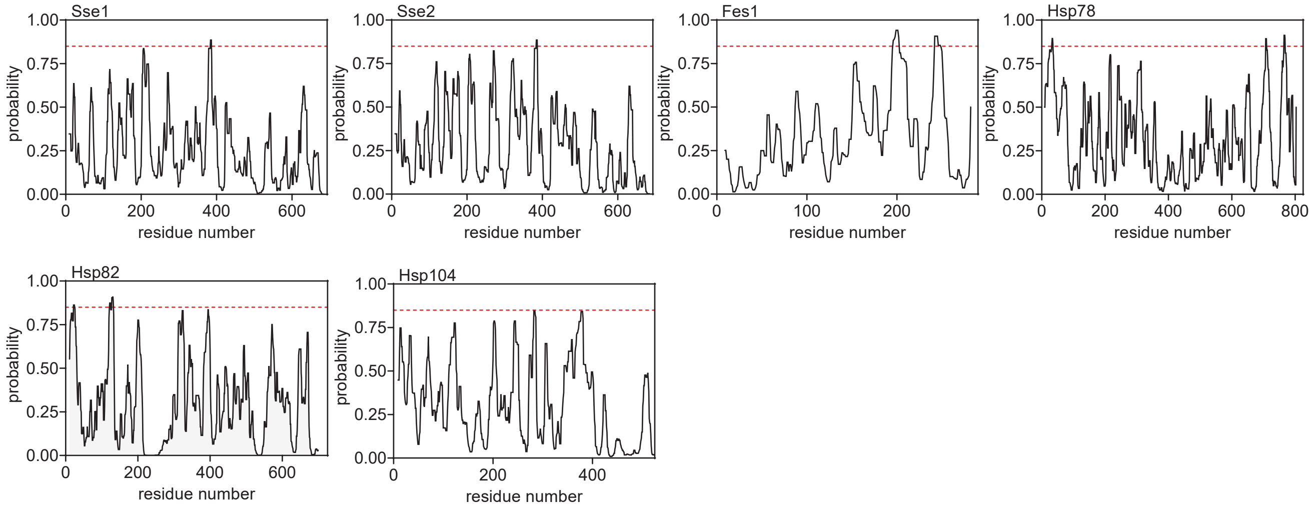

Supplementary Figure 7. QCDPred analysis of key cytosolic/nuclear yeast chaperones. A degron cutoff probability is marked at P = 0.85 by a dashed red line.

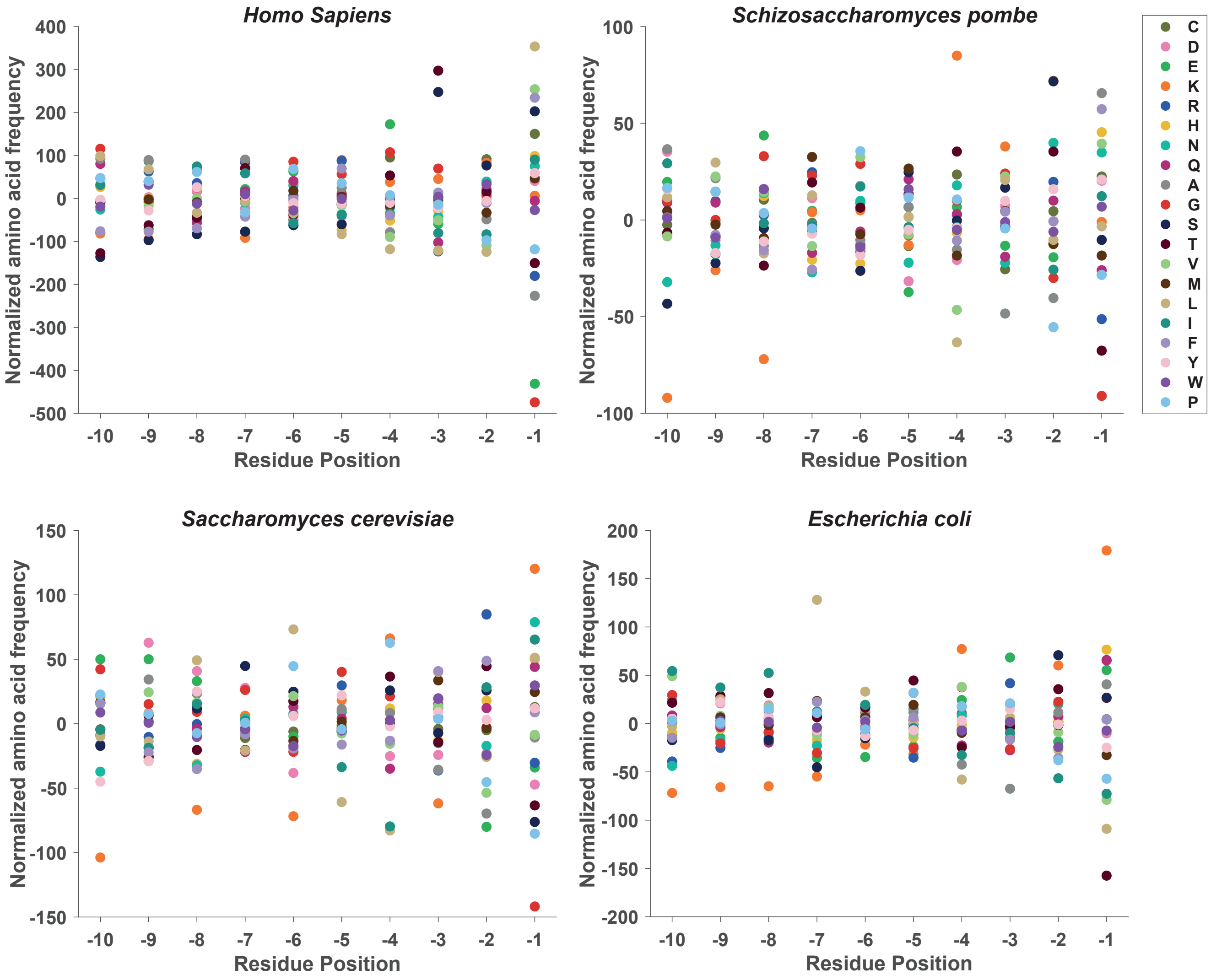

Supplementary Figure 8. C-Terminal Glycine is depleted from the proteome of Saccharomyces cerevisiae. Amino acid proportions across the last ten positions of each proteome are shown. The data for each residue are centered to have a mean zero. Protein number for each panel: *H. Sapiens,* n = 20,176; *S. Pombe,* n = 5,121*; S. Cerevisiae,* n = 6,616*; E. Coli,* n = 4,525.

Supplementary Table 1. Mutant P3 peptides and their QCDPred scores.

degron_name	seq	Probability
P3	FKRNAVFVGTIFAGAFV	0.89
P3_1E	FKRNAVFVGTI**E**AGAFV	0.72
P3_1R	FKRNAVFVGTI**R**AGAFV	0.83
P3_2E	FKRNAVFVGTI**E**AGA**E**V	0.44
P3_2R	FKRNAVFVGTI**R**AGA**R**V	0.74
P3_3E	FKRNAVFVGT**EE**AGA**E**V	0.18
P3_3R	FKRNAVFVGT**RR**AGA**R**V	0.60

Supplementary Table 2. Antibodies used in this study.

Polyclonal Rabbit anti-GFP	Abcam	ab290
Monoclonal Rabbit anti-mCherry	Abcam	ab213511
Monoclonal Rat anti-HA	Roche	11867423001
Monoclonal Mouse anti-PGK1	Abcam	ab113687
Polyclonal Rabbit anti-G6PD	Sigma Israel	a9521
Polyclonal Rabbit anti-Ubiquitin	Dako	Z0458
Goat anti-Rabbit	ImmunoResearch	111035144
Goat anti-Mouse	ImmunoResearch	115035062
Goat anti-Rat	ImmunoResearch	112035062

Supplementary Table 3. Yeast strains used in this study.

Strain name	Genotype
BY4741	*MATa met15Δ0, ura3Δ0, leu2Δ0, his3Δ1*
TRy1121	*MATa met15Δ0, ura3Δ0, leu2Δ0, his3Δ1, doa10::KanMX*
TRy1373	*MATa met15Δ0, ura3Δ0, leu2Δ0, his3Δ1, ubr1::KanMX*
TRy1388	*MATa met15Δ0, ura3Δ0, leu2Δ0, his3Δ1, san1::KanMX*
TRy1392	*MATa met15Δ0, ura3Δ0, leu2Δ0, his3Δ1, pdr5::KanMX*

Supplementary Table 4. List of plasmids used in this study.

Unless indicated otherwise, clones are C-terminal fusions to yeGFP.

Name	Description	Additional information
pTR1412	pRS315-CuP-URA3-HA-GFP-3xstop	From Geffen et al. [2]
pTR1494	pRS416-GPDp-HA-Pca1	From Adle et al.[3]
pTR1859	pRS315-ADH700p-yeCherry-p150-yeGFP-XmaI,NheI-CYCt	
pTR1861	pRS315-ADH700p-yeCherry-p150-yeGFP-linker-XmaI,NheI-CYCt	C-terminal fusion to yeG
pTR1882	pRS315-ADH700p-yeCherry-p150-yeGFP-linker-XmaI-*CL1*-NheI-CYCt	
pTR1884	pRS315-ADH700p-yeCherry-p150-yeGFP-linker-XmaI-*DegAB*-NheI-CYCt	
pTR2036	pRS315-ADH700p-yeCherry-p150-yeGFP-linker-XmaI-P1-NheI-CYCt	
pTR2037	pRS315-ADH700p-yeCherry-p150-yeGFP-linker-XmaI-P2-NheI-CYCt	
pTR2038	pRS315-ADH700p-yeCherry-p150-yeGFP-linker-XmaI-P3-NheI-CYCt	
pTR2058	pRS315-ADH700p-yeCherry-p150-yeGFP-linker-XmaI-P3$_{EE}$-NheI-CYCt	TIFAGAFV → TEEAGAEV
pTR2060	pRS315-ADH700p-yeCherry-p150-yeGFP-linker-XmaI-P3$_{RR}$-NheI-CYCt	TIFAGAFV → TRRAGARV
pTR2039	pRS315-ADH700p-yeCherry-p150-yeGFP-linker-XmaI-P4-NheI-CYCt	
pTR2040	pRS315-ADH700p-yeCherry-p150-yeGFP-linker-XmaI-P5-NheI-CYCt	
pTR2041	pRS315-ADH700p-yeCherry-p150-yeGFP-linker-XmaI-P6-NheI-CYCt	
pTR2042	pRS315-ADH700p-yeCherry-p150-yeGFP-linker-XmaI-P7-NheI-CYCt	
pTR2043	pRS315-ADH700p-yeCherry-p150-yeGFP-linker-XmaI-P8-NheI-CYCt	
pTR2044	pRS315-ADH700p-yeCherry-p150-yeGFP-linker-XmaI-P9-NheI-CYCt	
pTR2045	pRS315-ADH700p-yeCherry-p150-yeGFP-linker-XmaI-P10-NheI-CYCt	
pTR2089	pRS315-ADH700p-yeCherry-p150-PacI,BamHI-yeGFP-linker-XmaI,NheI-CYCt	N or C-terminal fusion
pTR2124	pRS416-GPDp-HA-Pca1$_{DD}$	LGCICNY→ LDCDCDY
pTR2127	pRS315-ADH700p-yeCherry-p150-yeGFP-linker-XmaI-$_{\Delta sp}$Erp2-NheI-CYCt	
pTR2128	pRS315-ADH700p-yeCherry-p150-yeGFP-linker-XmaI-$_{\Delta sp}$Atg27-NheI-CYCt	
pTR2129	pRS315-ADH700p-yeCherry-p150-yeGFP-linker-XmaI-$_{\Delta sp}$Ksh1-NheI-CYCt	
pTR2149	pRS315-ADH700p-yeCherry-p150-PacI-Erp2-BamHI-yeGFP-linker-CYCt	N-terminal fusion
pTR2151	pRS315-ADH700p-yeCherry-p150-PacI-Atg27-BamHI-yeGFP-linker-CYCt	N-terminal fusion
pTR2152	pRS315-ADH700p-yeCherry-p150-PacI-Ksh1-BamHI-yeGFP-linker-CYCt	N-terminal fusion
pTR2153	pRS315-ADH700p-yeCherry-p150-PacI-$_{\Delta sp}$Erp2-BamHI-yeGFP-linker-CYCt	N-terminal fusion
pTR2155	pRS315-ADH700p-yeCherry-p150-PacI-$_{\Delta sp}$Atg27-BamHI-yeGFP-linker-CYCt	N-terminal fusion
pTR2156	pRS315-ADH700p-yeCherry-p150-PacI-$_{\Delta sp}$Ksh1-BamHI-yeGFP-linker-CYCt	N-terminal fusion
pTR2160	pRS315-ADH700p-yeCherry-p150-yeGFP-linker-XmaI-SP(Ksh1)-NheI-CYCt	SP of Ksh1 (aa 1-26)
pTR2162	pRS315-ADH700-yeCherry-p150-yeGFP-linker-XmaI-SP(Erp2)-NheI-CYCt	SP of Erp2 (aa 1-26)
pTR2164	pRS315-ADH700p-yeCherry-p150-yeGFP-linker-XmaI-SP(Atg27)-NheI-CYCt	SP of Atg27 (aa 1-19)
pTR2167	pRS416 GPD HA-Pca1$_{RR}$	LGCICNY → LRCRCRY
pTR2171	pRS315-ADH700p-yeCherry-p150-yeGFP-linker-XmaI-SC1-NheI-CYCt	Scrambled P1, variant #1
pTR2172	pRS315-ADH700p-yeCherry-p150-yeGFP-linker-XmaI-SC2-NheI-CYCt	Scrambled P1, variant #2
pTR2213	pRS315-ADH700p-yeCherry-p150-yeGFP-linker-XmaI-TMD(Atg27)-NheI-CYCt	TMD of Atg27 (aa 200-220)
pTR2214	pRS315-ADH700p-yeCherry-p150-yeGFP-linker-XmaI-TMD(Erp2)-NheI-CYCt	TMD of Erp2 (aa 183-203)
pTR2215	pRS315-ADH700p-yeCherry-p150-yeGFP-linker-XmaI-TMD(Ksh1)-NheI-CYCt	TMD of Ksh1 (aa 54-72)
pTR2279	pRS315-ADH700p-yeCherry-p150-yeGFP-linker-XmaI-K-NheI-CYCt	
pTR2280	pRS315-ADH700p-yeCherry-p150-yeGFP-linker-XmaI-B1-NheI-CYCt	
pTR2281	pRS315-ADH700p-yeCherry-p150-yeGFP-linker-XmaI-B2-NheI-CYCt	

Supplementary Table 5. Oligos used in this study.

Name	Sequence
NGS-F	TCGTCGGCAGCGTCAGATGTGTATAAGAGACANNNGGATCAGCTGGCTCACCC
NGS-R	GTCTCGTGGGCTCGGAGATGTGTATAAGAGACAGANNNACATAACTAATTACATGATCAGTCAG
P1_F	CCGGGTATTTAGTACCTTTTATCATTGCTGCAATGGTTATTATGCATTTAATGGCATGAG
P1_R	CTAGCTCATGCCATTAAATGCATAATAACCATTGCAGCAATGATAAAAGGTACTAAATAC
P2_F	CCGGGATCATGAGGTGCAAAATATATTTCTTTTACTTAAGGATATCAAAAAAGCTATGAG
P2_R	CTAGCTCATAGCTTTTTTGATATCCTTAAGTAAAAGAAATATATTTTGCACCTCATGATC
P3_F	CCGGGTTCAAACGAAACGCTGTTTTTGTTGGTACTATCTTTGCAGGTGCCTTTGTTTGAG
P3_R	CTAGCTCAAACAAAGGCACCTGCAAAGATAGTACCAACAAAAACAGCGTTTCGTTTGAAC
P4_F	CCGGGAACAAGTTACACTACTGTGTTTCTTGTGCTATTCACGCCAGAATTGTCAGATGAG
P4_R	CTAGCTCATCTGACAATTCTGGCGTGAATAGCACAAGAAACACAGTAGTGTAACTTGTTC
P5_F	CCGGGTCTTTGTATTTGCTATCTATCTGGGTGAAGAAGTTCAAATGGGCCGGTATCTGAG
P5_R	CTAGCTCAGATACCGGCCCATTTGAACTTCTTCACCCAGATAGATAGCAAATACAAAGAC
P6_F	CCGGGGACGAACCTGATGAACAAGGTAACCCTAAAAAGAGACCAGGTAAGTTGTCCTGAG
P6_R	CTAGCTCAGGACAACTTACCTGGTCTCTTTTTAGGGTTACCTTGTTCATCAGGTTCGTCC
P7_F	CCGGGTCGGGTAAAAGTAACACCTCAAACGATGCTTCTAACAAAAAAGAAACCAAGTGAG
P7_R	CTAGCTCACTTGGTTTCTTTTTTGTTAGAAGCATCGTTTGAGGTGTTACTTTTACCCGAC
P8_F	CCGGGGTTATAGATAAACTAAAAAATGAAAACACGGATTTGAAAGACATTATCCAATGAG
P8_R	CTAGCTCATTGGATAATGTCTTTCAAATCCGTGTTTTCATTTTTTAGTTTATCTATAACC
P9_F	CCGGGTTAAATATCAGAATTCAAACCTACGGTGAAACGACCGCGGTGGATGCTCTTTGAG
P9_R	CTAGCTCAAAGAGCATCCACCGCGGTCGTTTCACCGTAGGTTTGAATTCTGATATTTAAC
P10_F	CCGGGAAGGTTGGGTCTATACGGGTTCACAAATCTGGAAAACTTTCCGTGAAAATTTGAG
P10_R	CTAGCTCAAATTTTCACGGAAAGTTTTCCAGATTTGTGAACCCGTATAGACCCAACCTTC
P3 E_{mut}_F	CCGGGTTCAAACGAAACGCTGTTTTTGTTGGTACTGAGGAGGCAGGTGCCGAGGTTTGAG
P3 E_{mut}_R	CTAGCTCAAACCTCGGCACCTGCCTCCTCAGTACCAACAAAAACAGCGTTTCGTTTGAAC
P3 R_{mut}_F	CCGGGTTCAAACGAAACGCTGTTTTTGTTGGTACTCGTCGTGCAGGTGCCCGTGTTTGAG
P3 R_{mut}_R	CTAGCTCAAACACGGGCACCTGCACGACGAGTACCAACAAAAACAGCGTTTCGTTTGAAC
Scr1_F	CCGGGATCTTATTTATGGTAGCTATTCCTGTTCATGCAATTGCATATTTAATGATGTGAG
Scr1_R	CTAGCTCACATCATTAAATATGCAATTGCATGAACAGGAATAGCTACCATAAATAAGATC
Scr2_F	CCGGGCATGCTGCAATCATGTATATGGCAGTTATTGTATTAATTTTTTTAATGCCTTGAG
Scr2_R	CTAGCTCAAGGCATTAAAAAAATTAATACAATAACTGCCATATACATGATTGCAGCATGC
K_F	CCGGGTTCGGCAAAAGCTTCTTCCAGAGGCTGAACCAGGACCGGCTTCTGGGCGGCTGAG
K_R	CTAGCTCAGCCGCCCAGAAGCCGGTCCTGGTTCAGCCTCTGGAAGAAGCTTTTGCCGAAC
B1_F	CCGGGGATTTTTTGCTATCTGATTGGAATAAAAGGTTCTCTCATCTTGCTGGTGGTTGAG
B1_R	CTAGCTCAACCACCAGCAAGATGAGAGAACCTTTTATTCCAATCAGATAGCAAAAAATCC
B2_F	CCGGGTACATGAGAAAACAAGTTGGTGTTGGTAAATTGAACAAATTATACGGTGGTTGAG
B2_R	CTAGCTCAACCACCGTATAATTTGTTCAATTTACCAACACCAACTTGTTTTCTCATGTAC

Supplementary References:

1. Hartley, A. M., Meunier, B., Pinotsis, N. & Maréchal, A. Rcf2 revealed in cryo-EM structures of hypoxic isoforms of mature mitochondrial III-IV supercomplexes. *Proc Natl Acad Sci U S A* **117**, 9329–9337 (2020).

2. Geffen, Y. et al. Mapping the landscape of a eukaryotic degronome. Mol Cell 63, 1055–1065 (2016).

3. Adle, D. J., Wei, W., Smith, N., Bies, J. J. & Lee, J. Cadmium-mediated rescue from ER-associated degradation induces expression of its exporter. Proc Natl Acad Sci U S A 106, 10189–10194 (2009).
