# Supplementary material for: Conserved degronome features governing quality control associated proteolysis
Source: Nat Commun. 2022 Dec 8;13:7588. doi: 10.1038/s41467-022-35298-y (PMC9732359; doi:10.1038/s41467-022-35298-y)
Supplement: Supplementary file 2 — Description of Additional Supplementary Files [file 41467_2022_35298_MOESM2_ESM.docx]

File Name: Supplementary Data 1

Description: related to Figure 1. proteins in the screen and the complex they belong to.

File Name: Supplementary Data 2

Description: related to Figure 2. PSI values for peptides in the screen in different strains.

File Name: Supplementary Data 3

Description: related to Figure 2. Degron profiles per protein. The PSI of each 17 amino acid tile was assigned to the central amino acid (black dots). An average PSI was calculated for all positions from the tiles that cover the position (typically three tiles). This profile was window-smoothed by a five-residue (plus/minus two) running median (black line). QCDPred scores were calculated for all possible 17-tiles of the proteins and assigned to the central amino acid (except for the first and last 8 positions which are assigned 0.5) and the profile was smoothed using the same five-residue running median. The gray area marks the intermediate PSI region considered to be inconclusive. Profiles are made for 306 proteins that have PSI values for 5 tiles or more.

File Name: Supplementary Data 4

Description: related to Figure 6. QCDPred analysis of the yeast proteome.

File Name: Supplementary Data 5

Description: related to Figure 6. QCDPred analysis of the human proteome.

File Name: Supplementary Data 6

Description: list of intact and mutated Doa10 degrons and their QCDPred probability score and their GRAVY scores.
