# Supplementary material for: Conserved degronome features governing quality control associated proteolysis
Source: Nat Commun. 2022 Dec 8;13:7588. doi: 10.1038/s41467-022-35298-y (PMC9732359; doi:10.1038/s41467-022-35298-y)
Supplement: Supplementary file 9 — Reporting Summary [file 41467_2022_35298_MOESM9_ESM.pdf]

nature portfolio

Corresponding author(s): Tommer Ravid

Last updated by author(s): Jun 20, 2022

Reporting Summary

Nature Portfolio wishes to improve the reproducibility of the work that we publish. This form provides structure for consistency and transparency in reporting. For further information on Nature Portfolio policies, see our Editorial Policies and the Editorial Policy Checklist.

Statistics

For all statistical analyses, confirm that the following items are present in the figure legend, table legend, main text, or Methods section.

n/a	Confirmed	
☐	☒	The exact sample size (*n*) for each experimental group/condition, given as a discrete number and unit of measurement
☐	☒	A statement on whether measurements were taken from distinct samples or whether the same sample was measured repeatedly
☐	☒	The statistical test(s) used AND whether they are one- or two-sided<br>*Only common tests should be described solely by name; describe more complex techniques in the Methods section.*
☒	☐	A description of all covariates tested
☐	☒	A description of any assumptions or corrections, such as tests of normality and adjustment for multiple comparisons
☐	☒	A full description of the statistical parameters including central tendency (e.g. means) or other basic estimates (e.g. regression coefficient) AND variation (e.g. standard deviation) or associated estimates of uncertainty (e.g. confidence intervals)
☐	☒	For null hypothesis testing, the test statistic (e.g. *F*, *t*, *r*) with confidence intervals, effect sizes, degrees of freedom and *P* value noted<br>*Give P values as exact values whenever suitable.*
☒	☐	For Bayesian analysis, information on the choice of priors and Markov chain Monte Carlo settings
☒	☐	For hierarchical and complex designs, identification of the appropriate level for tests and full reporting of outcomes
☒	☐	Estimates of effect sizes (e.g. Cohen's *d*, Pearson's *r*), indicating how they were calculated

Our web collection on statistics for biologists contains articles on many of the points above.

Software and code

Policy information about availability of computer code

Data collection	Sequencing data was collected via NextSeq.
Data analysis	The code for PSI calculation is available at https://github.com/KULL-Centre/_2022_Mashahreh_degronome.<br>The code for QCDPred, is available at:https://github.com/KULL-Centre/papers/blob/main/2022/degron-predict-Johansson-et-al.<br>A webserver to test QCDPred on any protein of interest is available at: https://colab.research.google.com/github/KULL-Centre/papers/blob/main/2022/degron-predict-Johansson-et-al/QCDpred.ipynb<br><br>Additional details about QCDPred are available in a related manuscript, currently under consideration (BioRxiv 2022.04.06.487301. https://doi.org/10.1101/2022.04.06.487301)<br><br>PDB files were visualized using PyMOL software, version 2.5.4.<br>FlowJo software, version 10.8.1. was used for flow cytometry analysis and visualization.<br>Imaging data were handled using Image, version 1.53t<br>MatLab, version R2021a, was used for data processing and analysis.<br>Structure prediction for chimera proteins was done using trRosetta web server https://yanglab.nankai.edu.cn/trRosetta/

For manuscripts utilizing custom algorithms or software that are central to the research but not yet described in published literature, software must be made available to editors and reviewers. We strongly encourage code deposition in a community repository (e.g. GitHub). See the Nature Portfolio guidelines for submitting code & software for further information.

Data

Policy information about availability of data

All manuscripts must include a data availability statement. This statement should provide the following information, where applicable:

- Accession codes, unique identifiers, or web links for publicly available datasets
- A description of any restrictions on data availability
- For clinical datasets or third party data, please ensure that the statement adheres to our policy

A data availability statement will be added to the manuscript as follows:
The authors declare that the main data supporting the findings of this study, including experimental procedures and compound characterization, are available within the article and its Supplementary Information files, or from the corresponding author upon request. Plasmid pTR2089 has been deposited with the corresponding sequence at Addgene. QCDPred analyses of yeast and human proteomes are available in Supplementary Data 4, 5, respectively. QCDPred analyses of other proteins of interest are available on a web server described by Johansson et al22. Source data are provided with this paper. PDB availability: 6T0B [http://doi.org/10.2210/pdb6T0B/pdb].

Human research participants

Policy information about studies involving human research participants and Sex and Gender in Research.

Reporting on sex and gender	N/A
Population characteristics	N/A
Recruitment	N/A
Ethics oversight	N/A

Note that full information on the approval of the study protocol must also be provided in the manuscript.

Field-specific reporting

Please select the one below that is the best fit for your research. If you are not sure, read the appropriate sections before making your selection.

☒ Life sciences ☐ Behavioural & social sciences ☐ Ecological, evolutionary & environmental sciences

For a reference copy of the document with all sections, see nature.com/documents/nr-reporting-summary-flat.pdf

Life sciences study design

All studies must disclose on these points even when the disclosure is negative.

Sample size	1.The cut-off at 10,000 events is considered as the gold standard in flow cytometry and therefore we collected 10,000 cells in all experiments.<br>2. 10 million cells were sorted by FACS for downstream applications to maintain ~ 300-fold coverage of the library.<br>3. for immunobloting, protein lysates equivalent to 0.25 OD of live cells were loaded on each well. This amount is sufficient for protein visualization without overloading acrylamide gel.<br>4. Peptides were considered as degons if their PSI < 1.7. Overall 2243 peptides out of 23600 were considered as degrons. For analyses that were based on QCDPred a cut of 0.85 was chosen arbitrarily to define high confident degrons this is correlated with a PSI value of 1.62
Data exclusions	For flow cytometry presentation a sub-population of extremely strong fluorescence signal, likely representing dead cells, was excluded from the plots
Replication	All flow cytometry and immunoblot analyses were repeated reproducibly at least two times.<br>Immunoblots were repeated reproducibly 2-3 times. The best image was chosen as representative<br>Microscopy analysis was performed twice. Representative examples were chosen from fields of 50-100 cells.
Randomization	Visualization and processing of flow cytometry data was done using randomly chosen 10,000 events out of 0.5-1x10^7 cells<br>Visualization of data in Figures 6e and 6f was done using randomly chosen 10% and 50% events, respectively, out of the total PSI scores.<br>Peptide scrambling was done randomly using an online server peptidenexus.com
Blinding	Blinding experiments are not commonly done in biochemical and molecular biology studies and were not performed in this study.

Reporting for specific materials, systems and methods

We require information from authors about some types of materials, experimental systems and methods used in many studies. Here, indicate whether each material, system or method listed is relevant to your study. If you are not sure if a list item applies to your research, read the appropriate section before selecting a response.

Materials & experimental systems

n/a	Involved in the study
☐	☒ Antibodies
☒	☐ Eukaryotic cell lines
☒	☐ Palaeontology and archaeology
☒	☐ Animals and other organisms
☒	☐ Clinical data
☒	☐ Dual use research of concern

Methods

n/a	Involved in the study
☒	☐ ChIP-seq
☐	☒ Flow cytometry
☒	☐ MRI-based neuroimaging

Antibodies

Antibodies used	Polyclonal Rabbit anti-GFP Abcam ab290<br>Monoclonal Rabbit anti-mCherry Abcam ab213511<br>Monoclonal Rat anti-HA Roche 11867423001<br>Monoclonal Mouse anti-PGK1 Abcam ab113687<br>Polyclonal Rabbit anti-G6PD Sigma Israel a9521<br>Polyclonal Rabbit anti-Ubiquitin Dako Z0458
Validation	https://www.abcam.com/gfp-antibody-ab290.html, https://www.abcam.com/mcherry-antibody-epr20579-ab213511.html, https://www.fishersci.com/shop/products/anti-ha-high-affinity-50-ug/501003325, https://www.abcam.com/pgk1-antibody-22c5d8-ab113687.html, https://www.sigmaaldrich.com/IL/en/search/a9521?focus=products&page=1&perpage=30&sort=relevance&term=a9521&type=product_name, https://www.citeab.com/antibodies/3382935-z0458-ubiquitin

Flow Cytometry

Plots

Confirm that:

☒ The axis labels state the marker and fluorochrome used (e.g. CD4-FITC).

☒ The axis scales are clearly visible. Include numbers along axes only for bottom left plot of group (a 'group' is an analysis of identical markers).

☒ All plots are contour plots with outliers or pseudocolor plots.

☒ A numerical value for number of cells or percentage (with statistics) is provided.

Methodology

Sample preparation	yeast strains were grown to mid-log phase. sample was applied to the flow cytometry/ FACS machine.
Instrument	CellStream analyzer instrument (Merck)
Software	FlowJo V10.8.1 software (BD Biosciences)
Cell population abundance	Samples post FACS were reloaded on the machine to determine no overlaps between gates.
Gating strategy	A forward scatter (FSC) vs. forward scatter (FSC) was plotted to exclude doublets. Then, mCherry vs. GFP of the singlets population was plotted. Yeast strain lacking both florescent proteins was used to define the gate of the positive population. The positive population was then divided into equal four gates.

☒ Tick this box to confirm that a figure exemplifying the gating strategy is provided in the Supplementary Information.
