# Supplementary material for: Conserved degronome features governing quality control associated proteolysis
Source: Nat Commun. 2022 Dec 8;13:7588. doi: 10.1038/s41467-022-35298-y (PMC9732359; doi:10.1038/s41467-022-35298-y)
Supplement: Supplementary file 5 — Supplementary Data 3 [file 41467_2022_35298_MOESM5_ESM.pdf]

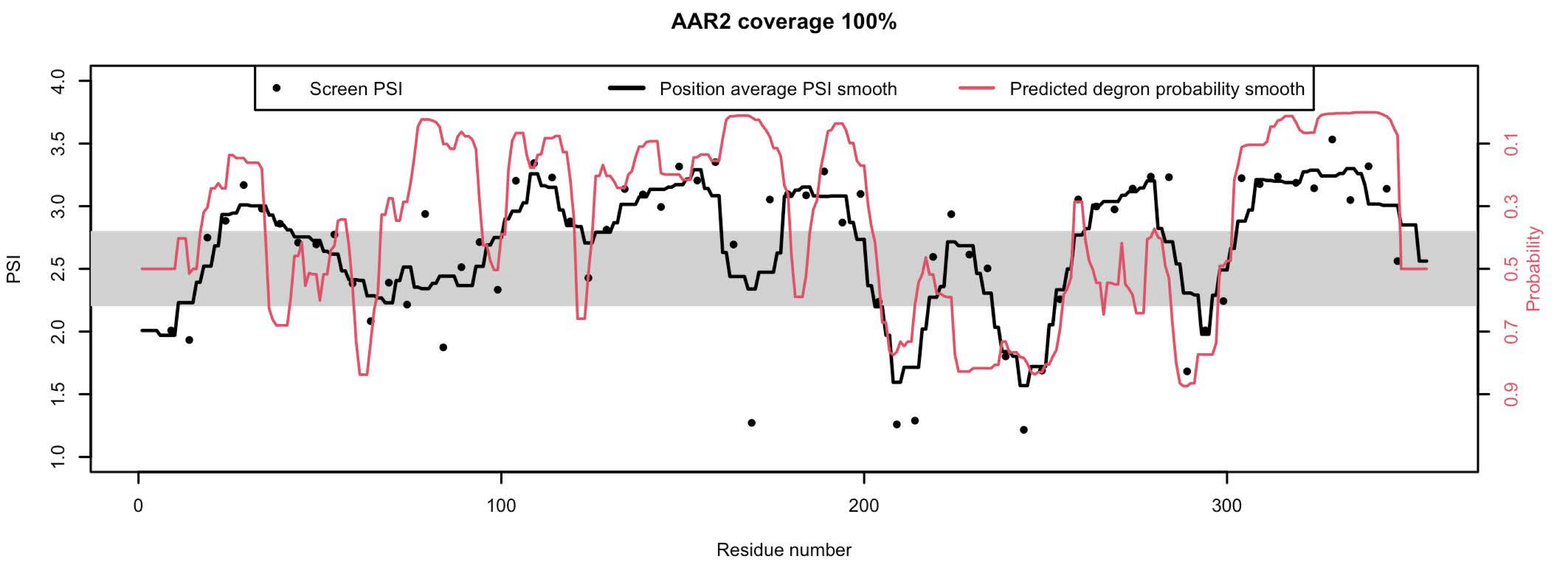

AAR2 coverage 100%
Screen PSI
Position average PSI smooth
Predicted degron probability smooth
PSI
1.0
1.5
2.0
2.5
3.0
3.5
4.0
Probability
0.1
0.3
0.5
0.7
0.9
0
100
200
300
Residue number

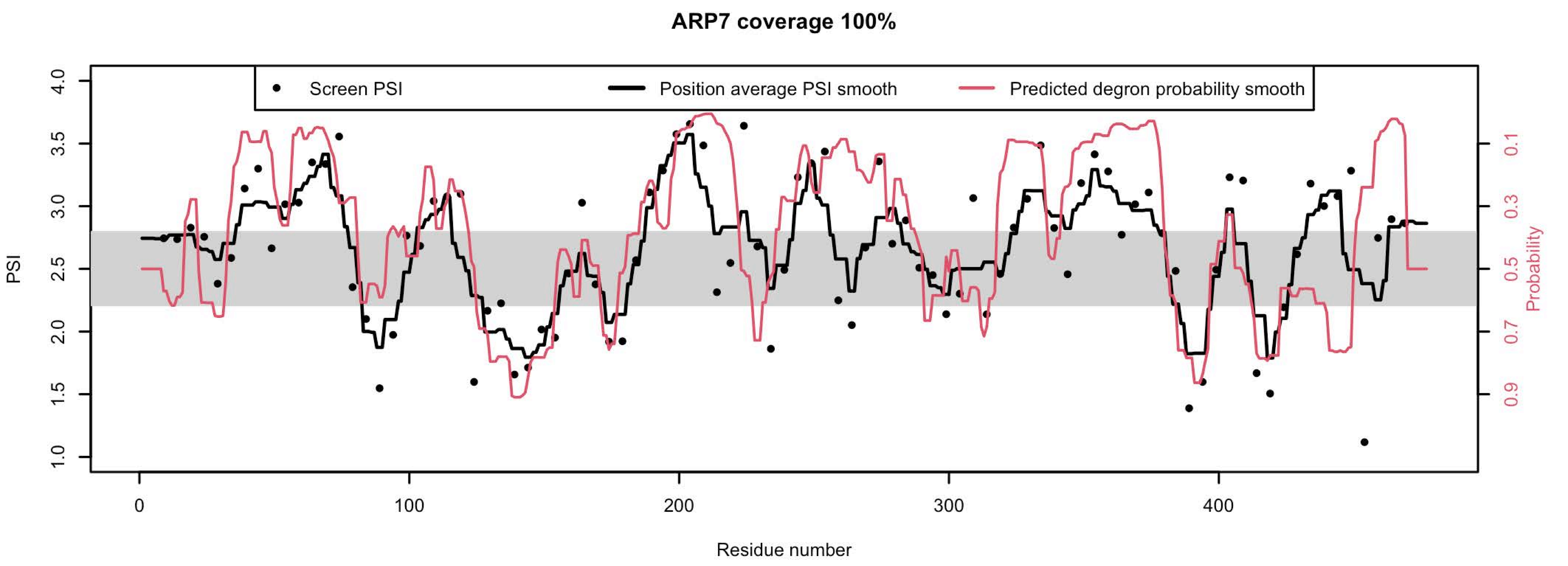
ARP7 coverage 100%
Screen PSI
Position average PSI smooth
Predicted degron probability smooth
PSI
1.0
1.5
2.0
2.5
3.0
3.5
4.0
Probability
0.1
0.3
0.5
0.7
0.9
0
100
200
300
400
Residue number

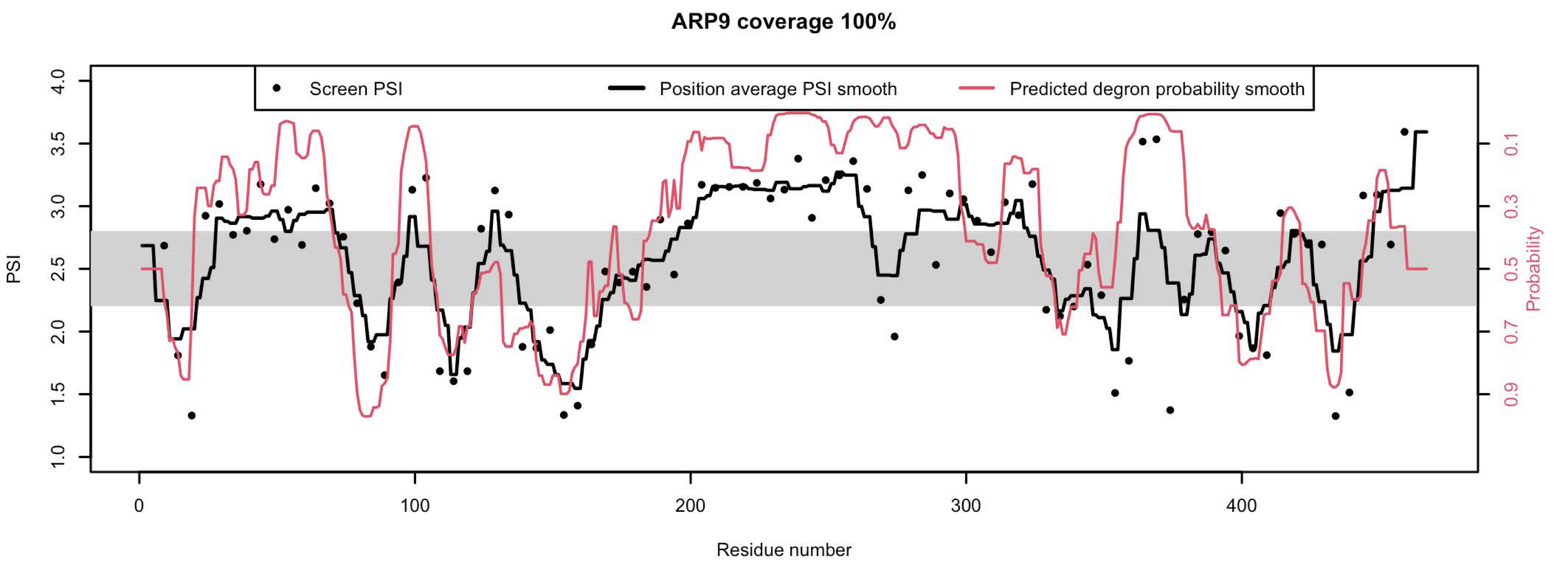

ARP9 coverage 100%
Screen PSI
Position average PSI smooth
Predicted degron probability smooth
PSI
1.0
1.5
2.0
2.5
3.0
3.5
4.0
Probability
0.1
0.3
0.5
0.7
0.9
0
100
200
300
400
Residue number

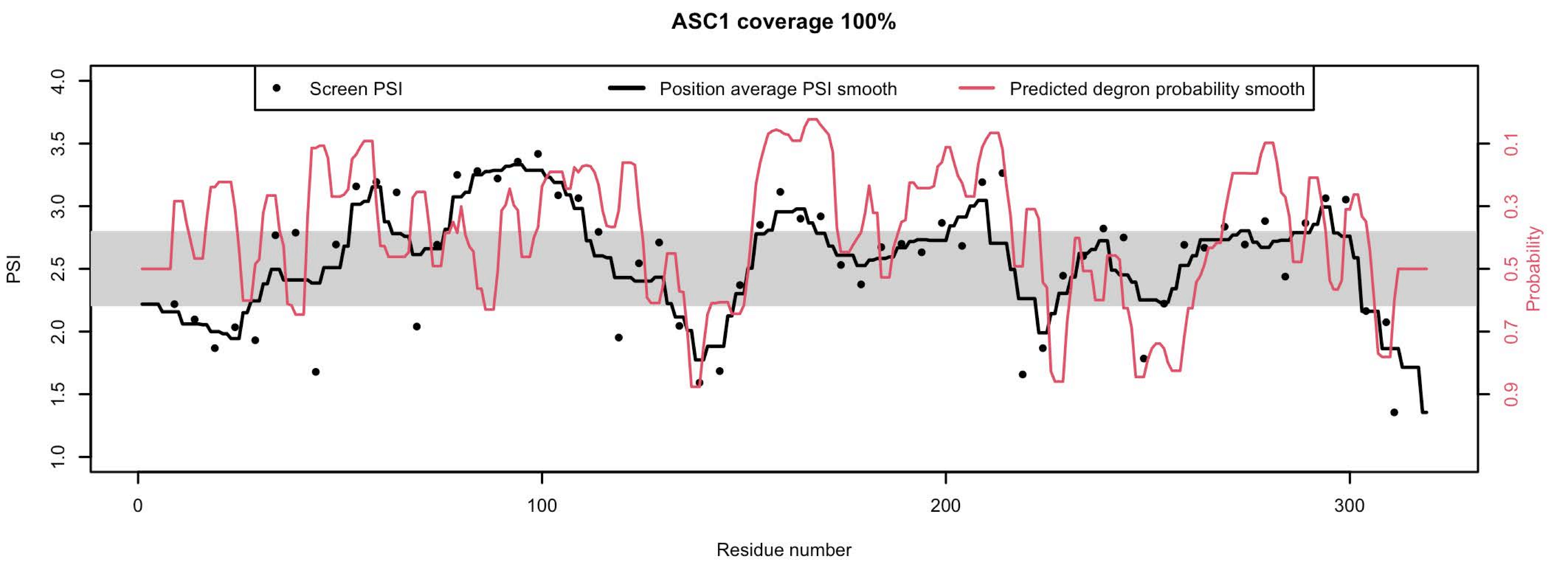
ASC1 coverage 100%
Screen PSI
Position average PSI smooth
Predicted degron probability smooth
PSI
4.0
3.5
3.0
2.5
2.0
1.5
1.0
Probability
0.1
0.3
0.5
0.7
0.9
0
100
200
300
Residue number

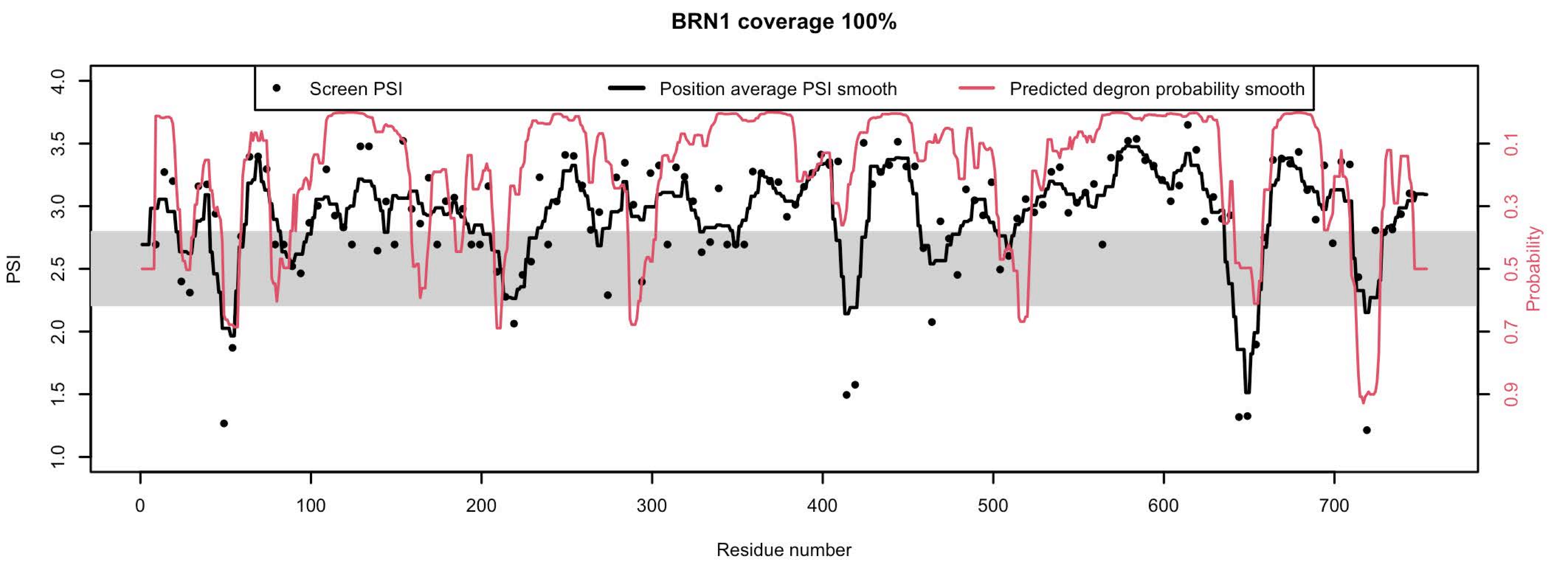
BRN1 coverage 100%
Screen PSI
Position average PSI smooth
Predicted degron probability smooth
PSI
Probability
Residue number
1.0
1.5
2.0
2.5
3.0
3.5
4.0
0.1
0.3
0.5
0.7
0.9
0
100
200
300
400
500
600
700

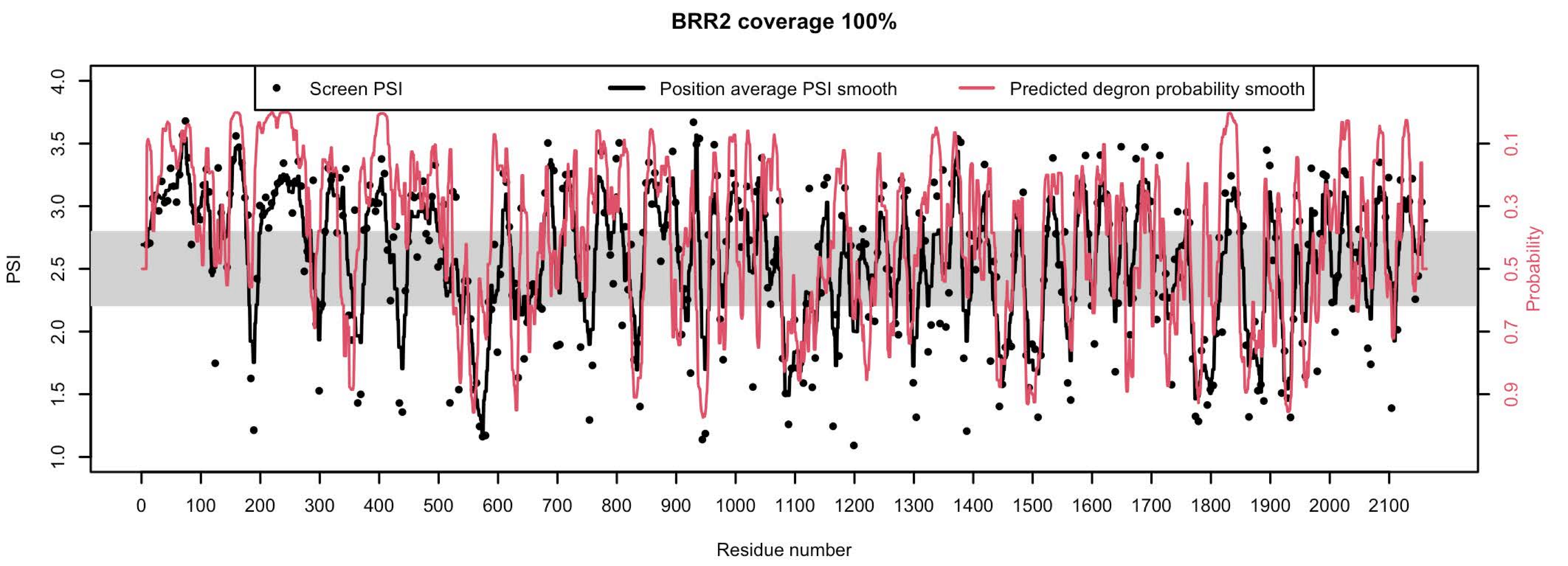

BRR2 coverage 100%
Screen PSI
Position average PSI smooth
Predicted degron probability smooth
PSI
Probability
Residue number
1.0
1.5
2.0
2.5
3.0
3.5
4.0
0.1
0.3
0.5
0.7
0.9
0
100
200
300
400
500
600
700
800
900
1000
1100
1200
1300
1400
1500
1600
1700
1800
1900
2000
2100

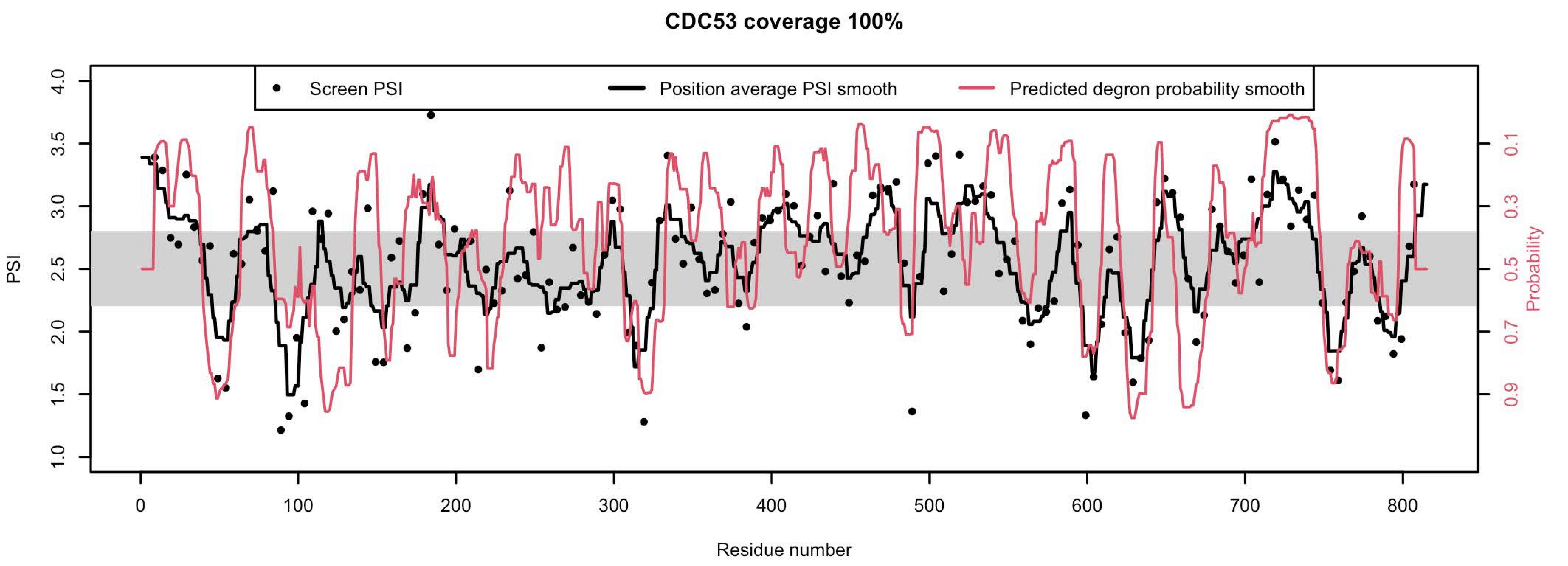
CDC53 coverage 100%
Screen PSI
Position average PSI smooth
Predicted degron probability smooth
PSI
Probability
Residue number
1.0
1.5
2.0
2.5
3.0
3.5
4.0
0.1
0.3
0.5
0.7
0.9
0
100
200
300
400
500
600
700
800

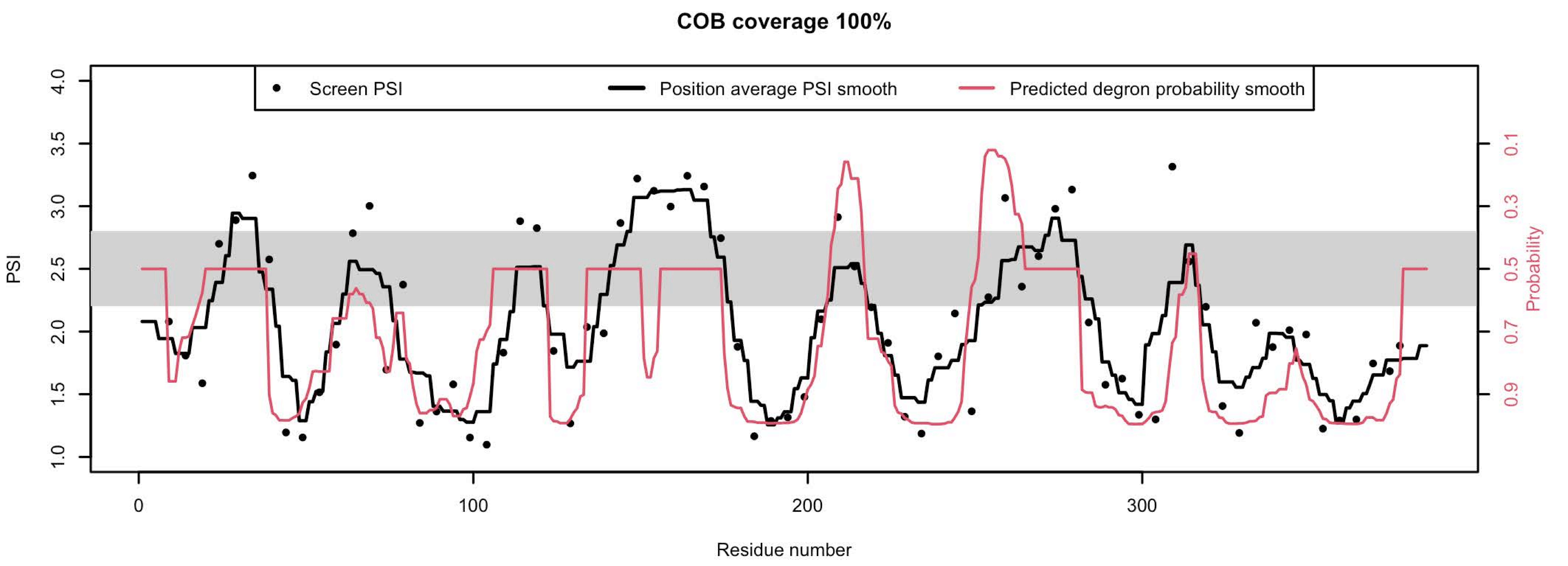

COB coverage 100%
Screen PSI
Position average PSI smooth
Predicted degron probability smooth
PSI
1.0
1.5
2.0
2.5
3.0
3.5
4.0
Probability
0.1
0.3
0.5
0.7
0.9
0
100
200
300
Residue number

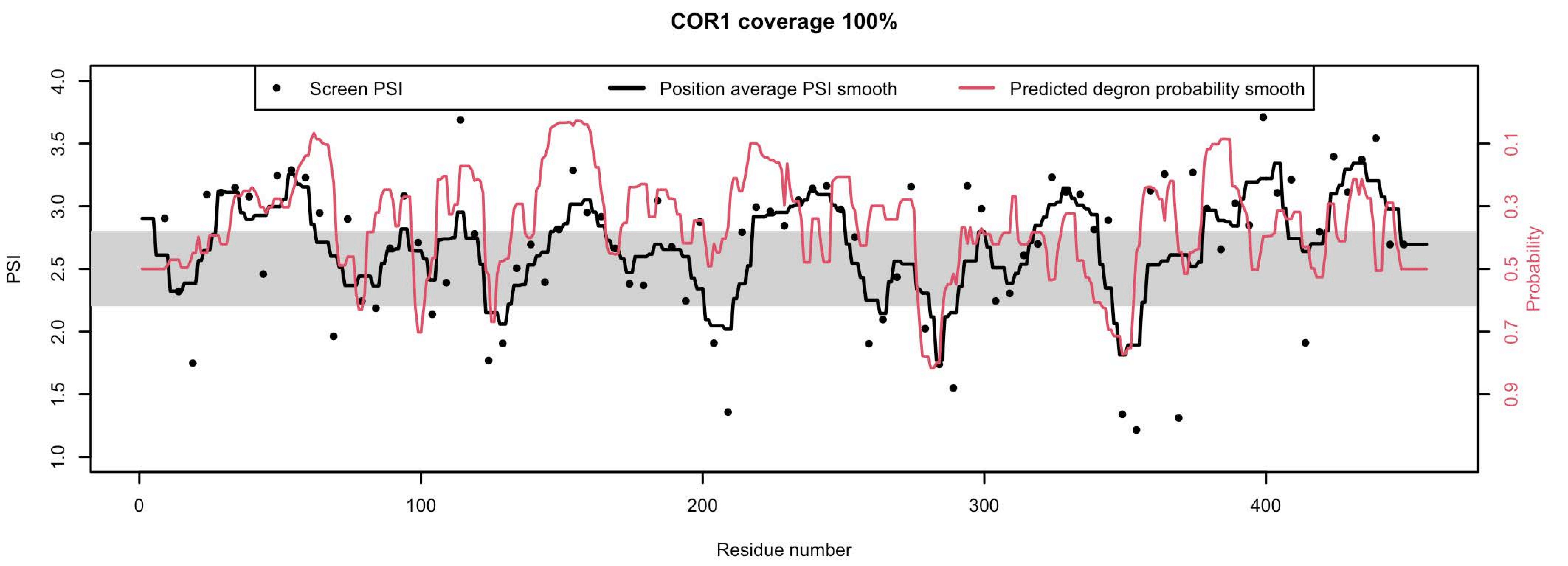

COR1 coverage 100%
Screen PSI
Position average PSI smooth
Predicted degron probability smooth
PSI
1.0
1.5
2.0
2.5
3.0
3.5
4.0
Probability
0.1
0.3
0.5
0.7
0.9
0
100
200
300
400
Residue number

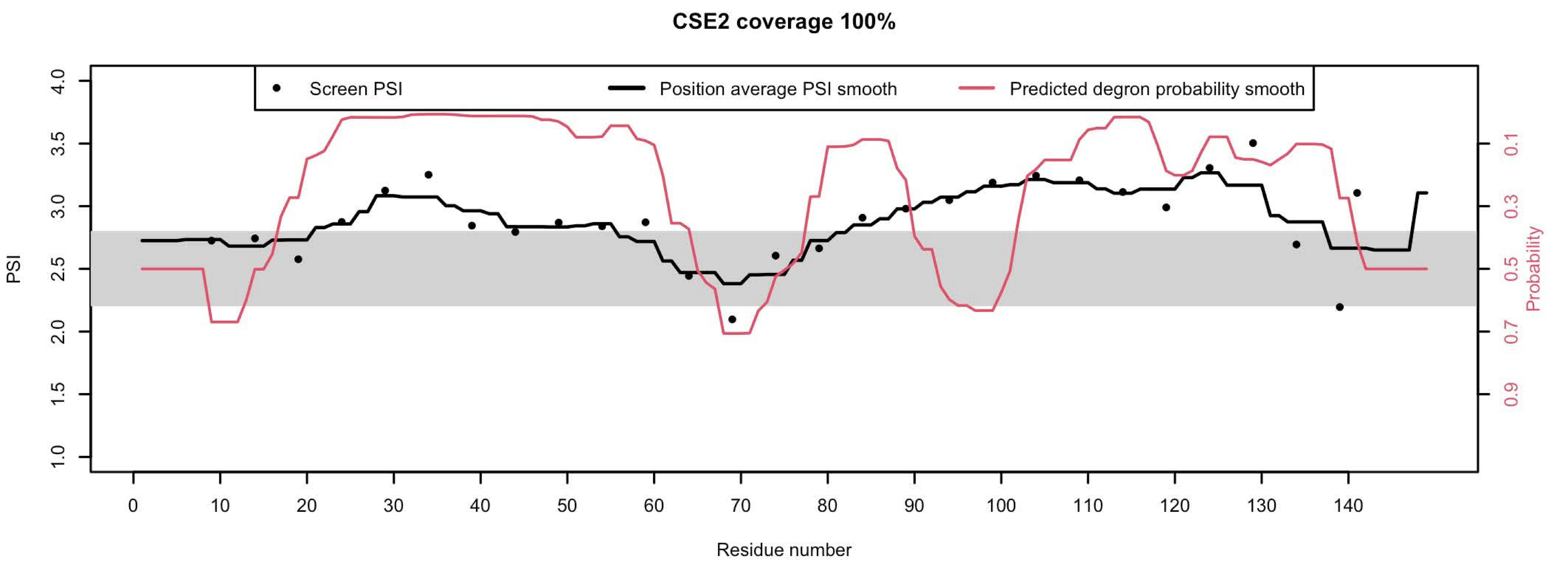

CSE2 coverage 100%
Screen PSI
Position average PSI smooth
Predicted degron probability smooth
PSI
Probability
Residue number
1.0
1.5
2.0
2.5
3.0
3.5
4.0
0.1
0.3
0.5
0.7
0.9
0
10
20
30
40
50
60
70
80
90
100
110
120
130
140

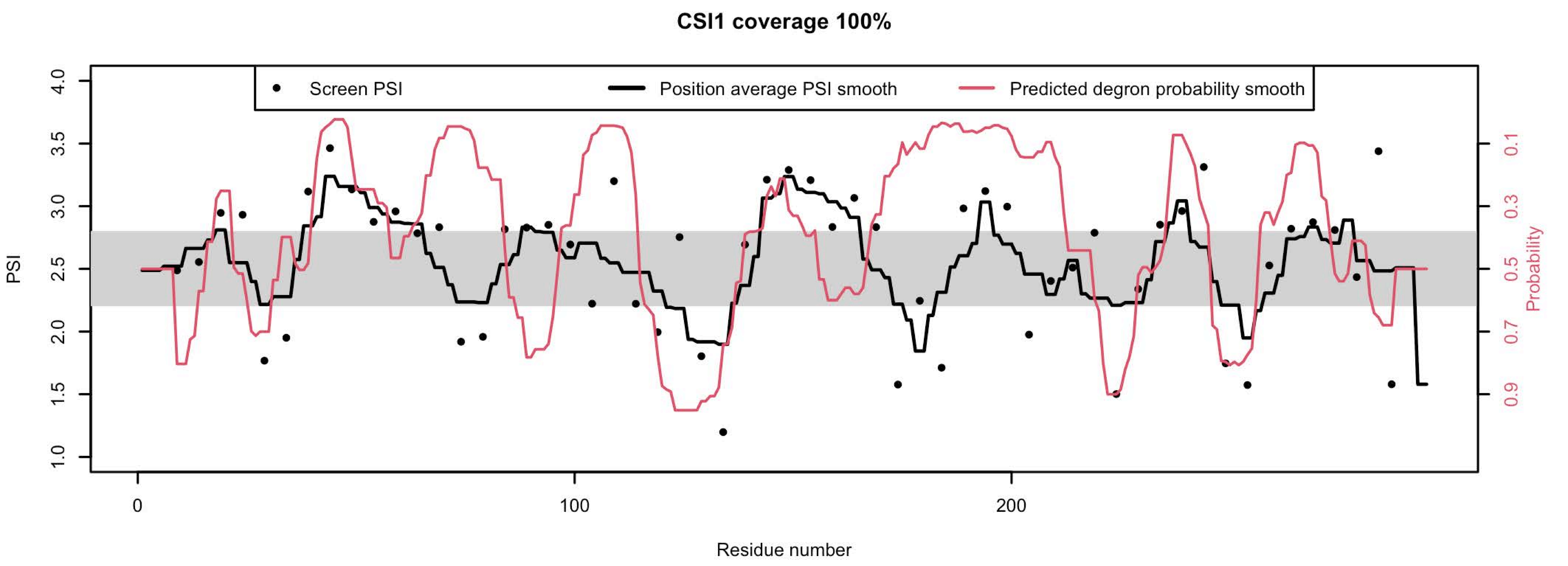
CSI1 coverage 100%
Screen PSI
Position average PSI smooth
Predicted degron probability smooth
PSI
1.0
1.5
2.0
2.5
3.0
3.5
4.0
0
100
200
Residue number
Probability
0.1
0.3
0.5
0.7
0.9

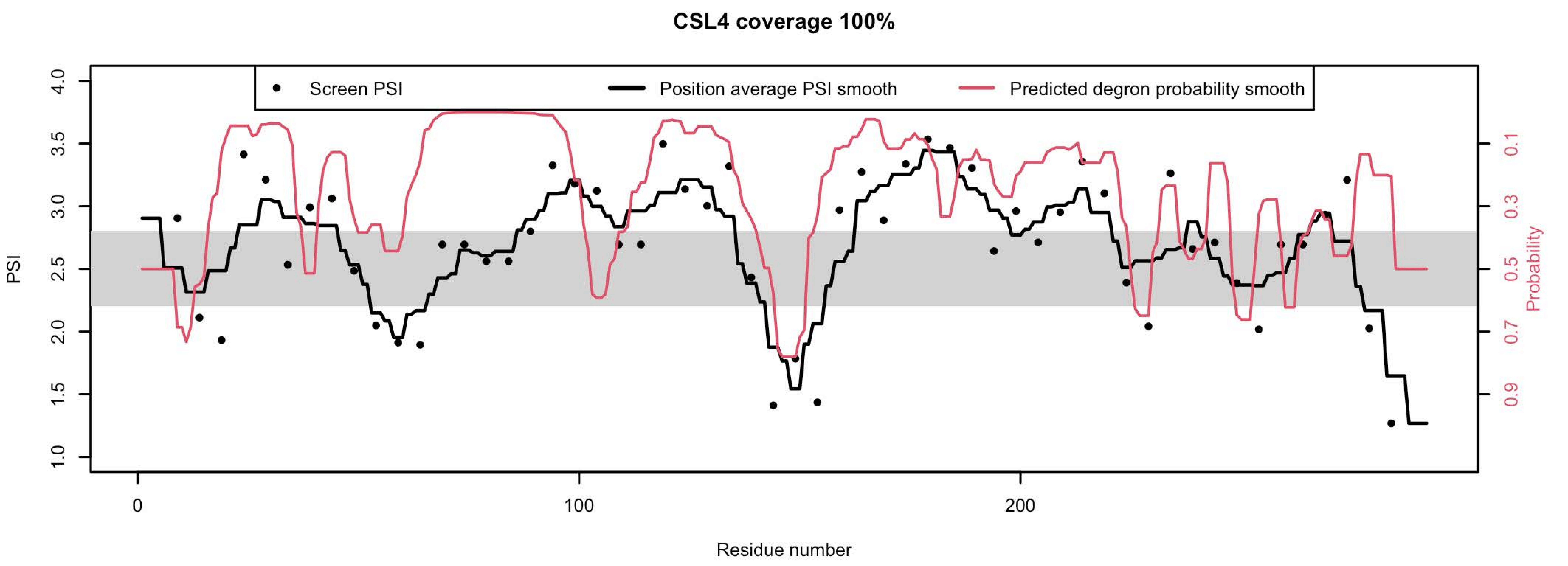

CSL4 coverage 100%
Screen PSI
Position average PSI smooth
Predicted degron probability smooth
PSI
Probability
Residue number
1.0
1.5
2.0
2.5
3.0
3.5
4.0
0.1
0.3
0.5
0.7
0.9
0
100
200

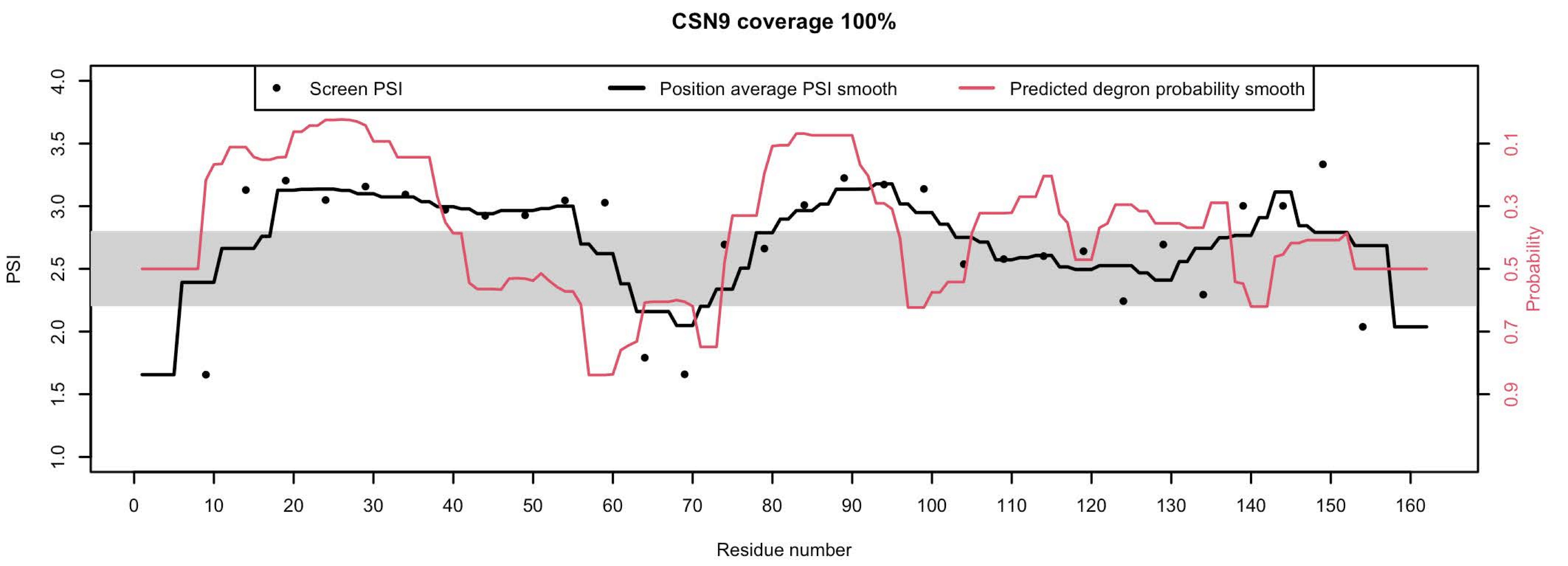
CSN9 coverage 100%
Screen PSI
Position average PSI smooth
Predicted degron probability smooth
PSI
1.0
1.5
2.0
2.5
3.0
3.5
4.0
Probability
0.1
0.3
0.5
0.7
0.9
Residue number
0
10
20
30
40
50
60
70
80
90
100
110
120
130
140
150
160

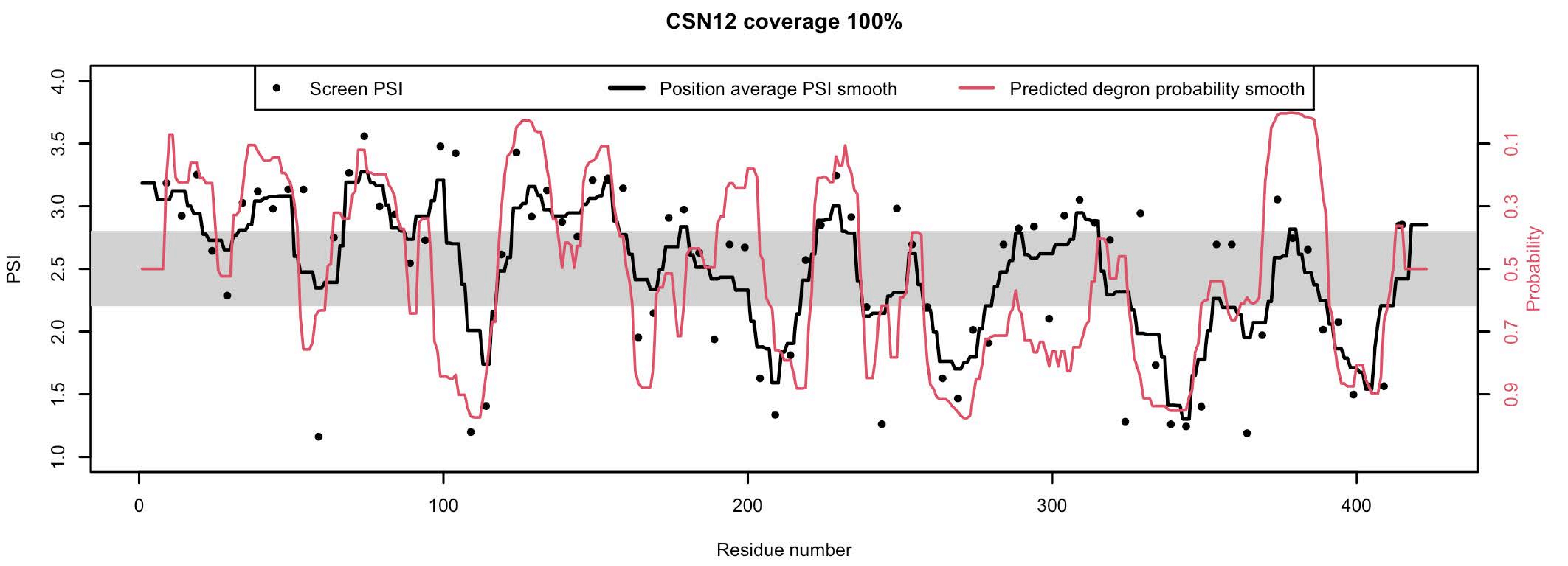

CSN12 coverage 100%
Screen PSI
Position average PSI smooth
Predicted degron probability smooth
PSI
Probability
Residue number
1.0
1.5
2.0
2.5
3.0
3.5
4.0
0.1
0.3
0.5
0.7
0.9
0
100
200
300
400

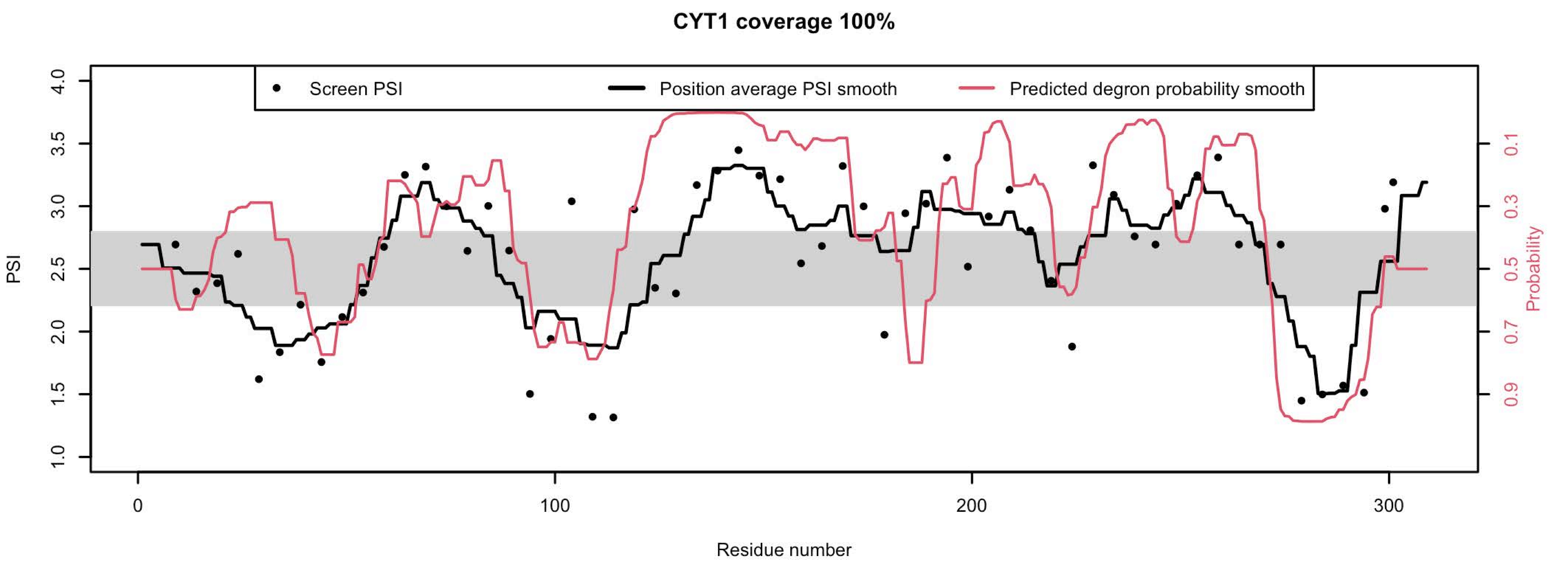

CYT1 coverage 100%
Screen PSI
Position average PSI smooth
Predicted degron probability smooth
PSI
1.0
1.5
2.0
2.5
3.0
3.5
4.0
Probability
0.1
0.3
0.5
0.7
0.9
0
100
200
300
Residue number

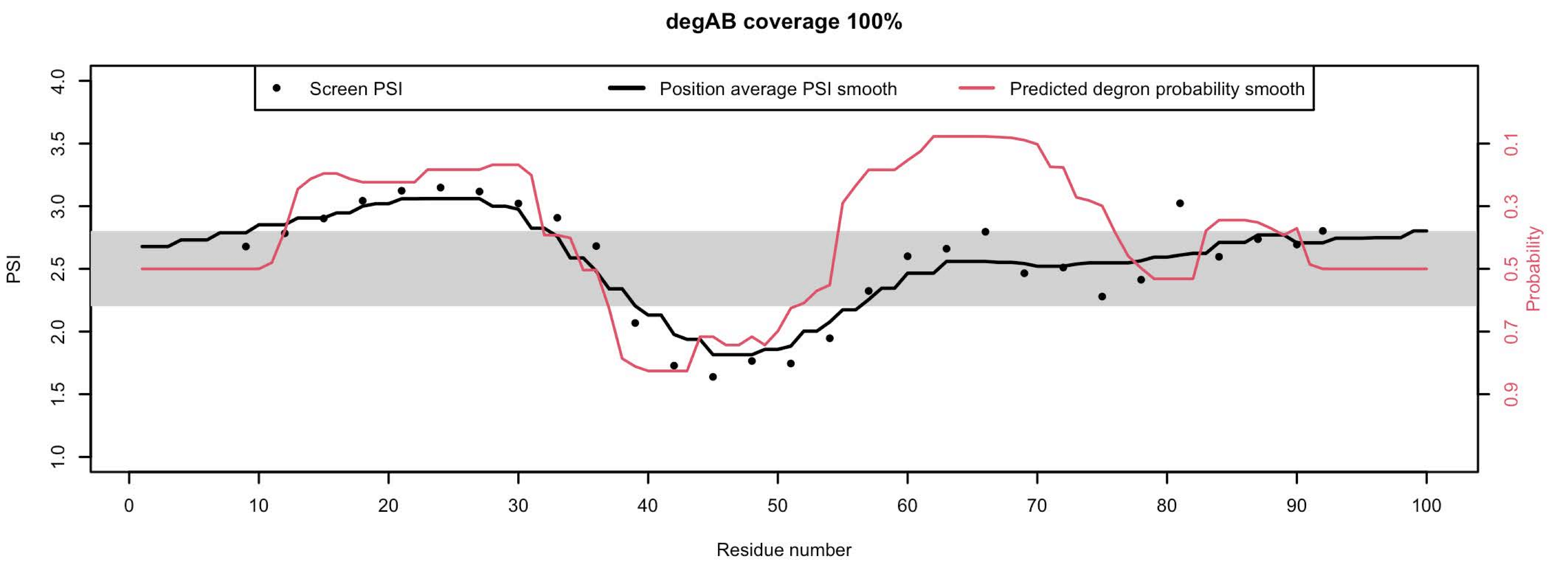
degAB coverage 100%
Screen PSI
Position average PSI smooth
Predicted degron probability smooth
PSI
4.0
3.5
3.0
2.5
2.0
1.5
1.0
Probability
0.1
0.3
0.5
0.7
0.9
Residue number
0
10
20
30
40
50
60
70
80
90
100

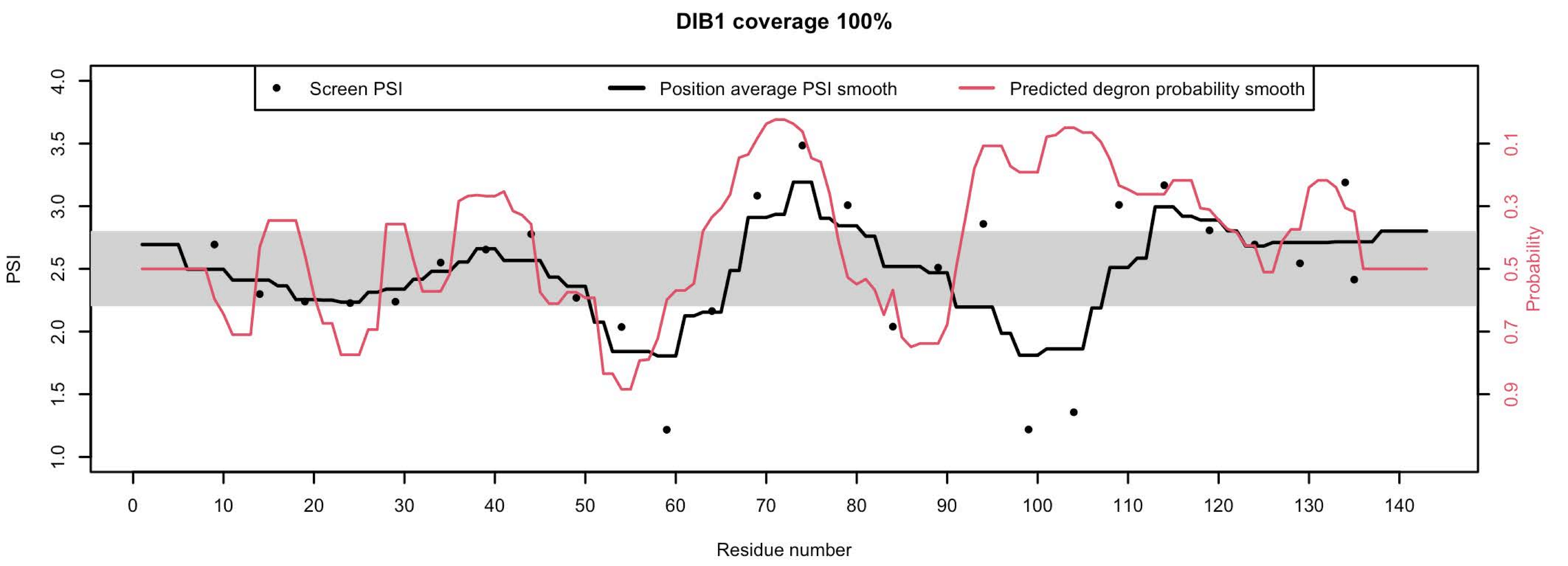
DIB1 coverage 100%
Screen PSI
Position average PSI smooth
Predicted degron probability smooth
PSI
Probability
Residue number
1.0
1.5
2.0
2.5
3.0
3.5
4.0
0.1
0.3
0.5
0.7
0.9
0
10
20
30
40
50
60
70
80
90
100
110
120
130
140

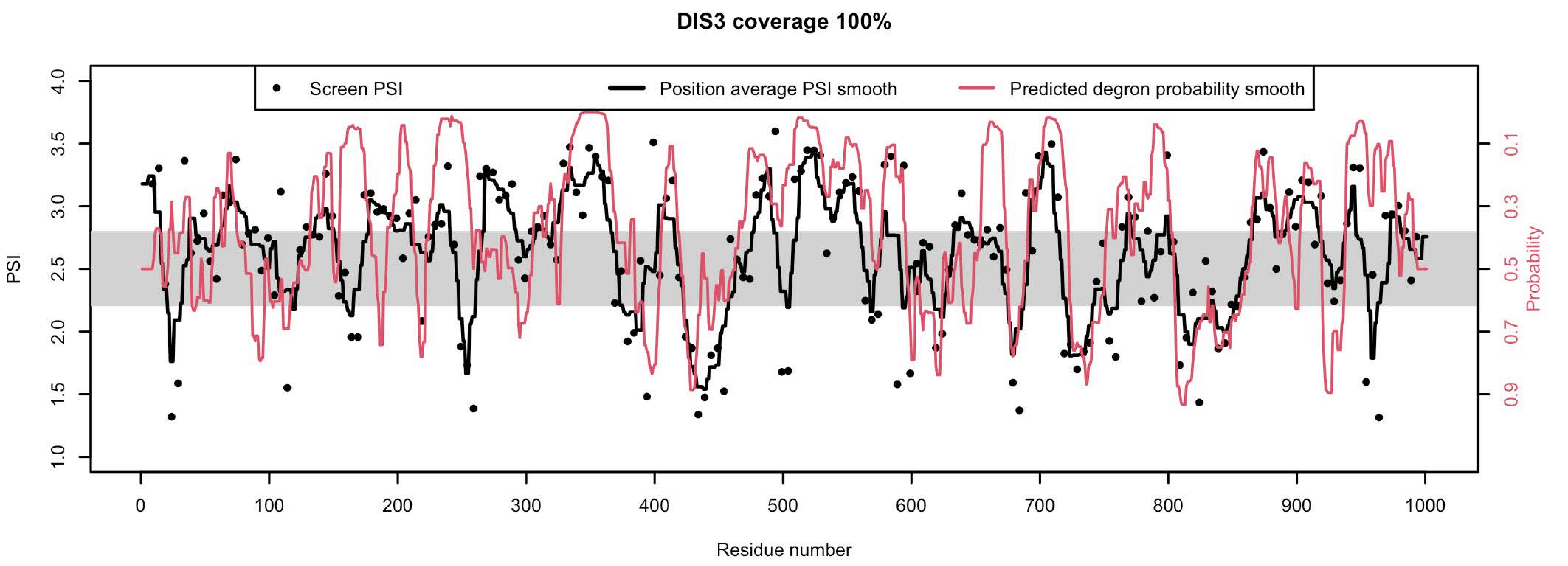

DIS3 coverage 100%
Screen PSI
Position average PSI smooth
Predicted degron probability smooth
PSI
Probability
Residue number
1.0
1.5
2.0
2.5
3.0
3.5
4.0
0.1
0.3
0.5
0.7
0.9
0
100
200
300
400
500
600
700
800
900
1000

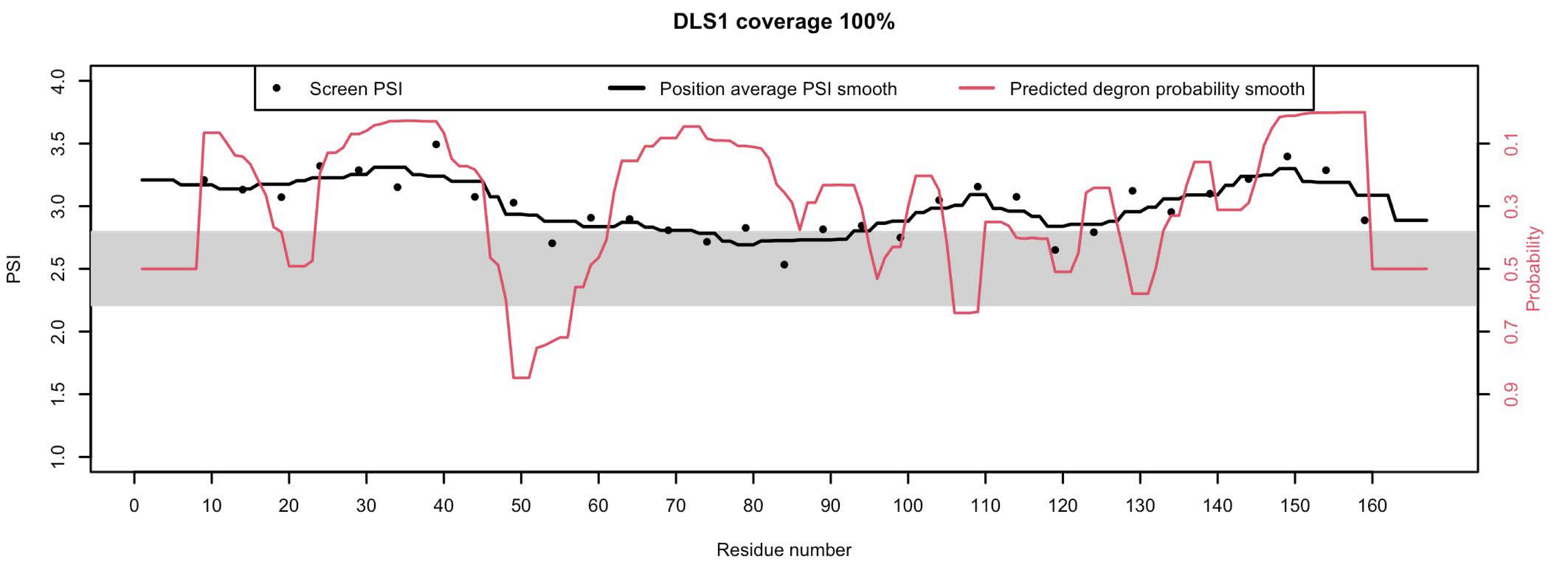
DLS1 coverage 100%
Screen PSI
Position average PSI smooth
Predicted degron probability smooth
PSI
1.0
1.5
2.0
2.5
3.0
3.5
4.0
Probability
0.1
0.3
0.5
0.7
0.9
0
10
20
30
40
50
60
70
80
90
100
110
120
130
140
150
160
Residue number

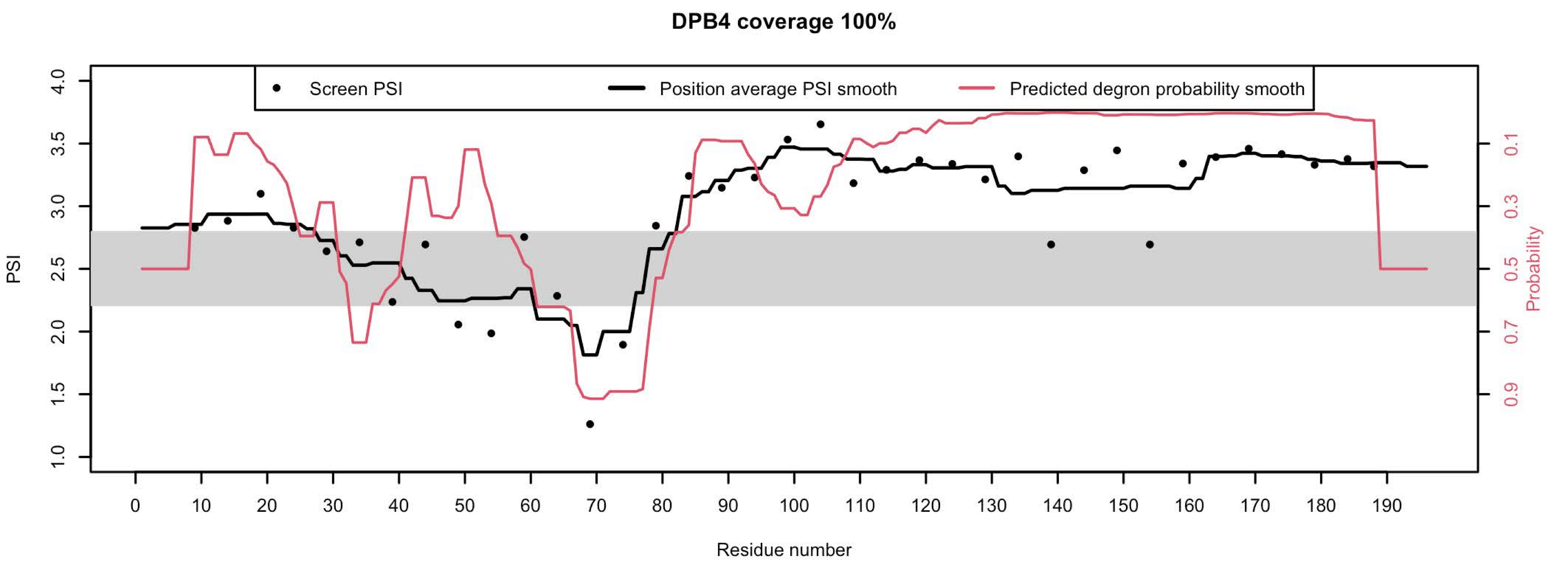

DPB4 coverage 100%
Screen PSI
Position average PSI smooth
Predicted degron probability smooth
PSI
1.0
1.5
2.0
2.5
3.0
3.5
4.0
Probability
0.1
0.3
0.5
0.7
0.9
Residue number
0
10
20
30
40
50
60
70
80
90
100
110
120
130
140
150
160
170
180
190

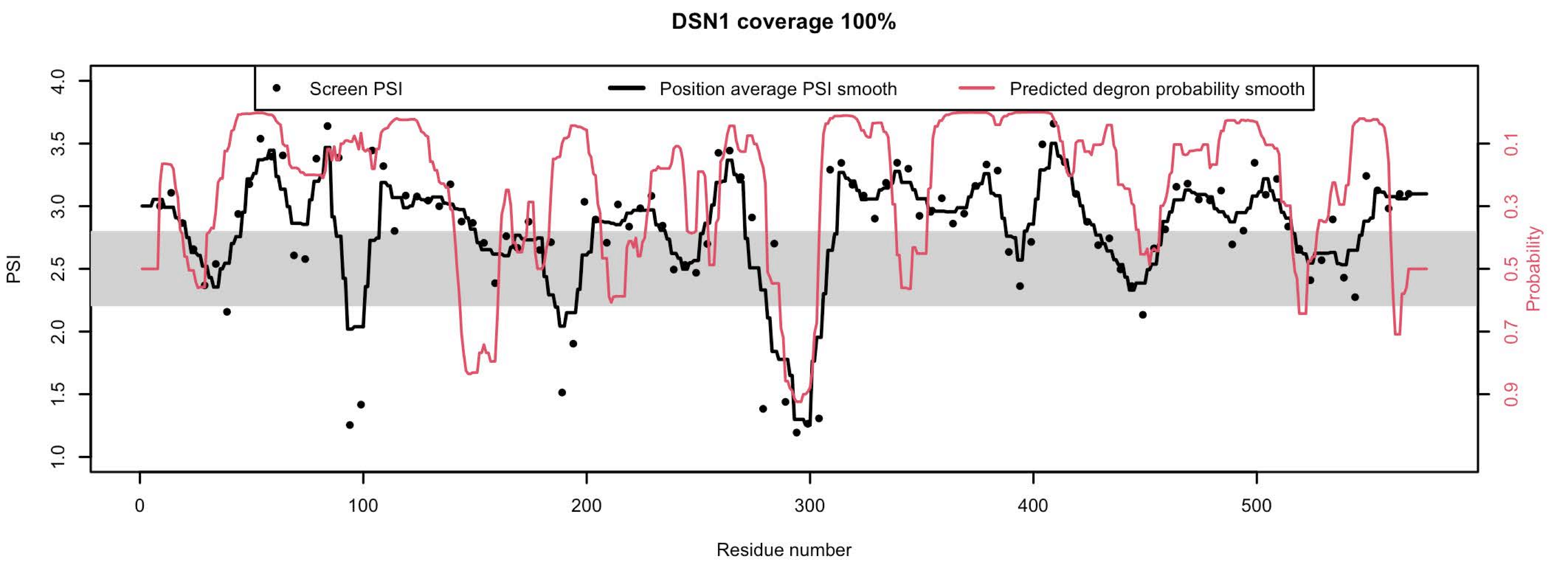
DSN1 coverage 100%
Screen PSI
Position average PSI smooth
Predicted degron probability smooth
PSI
Probability
Residue number
1.0
1.5
2.0
2.5
3.0
3.5
4.0
0.1
0.3
0.5
0.7
0.9
0
100
200
300
400
500

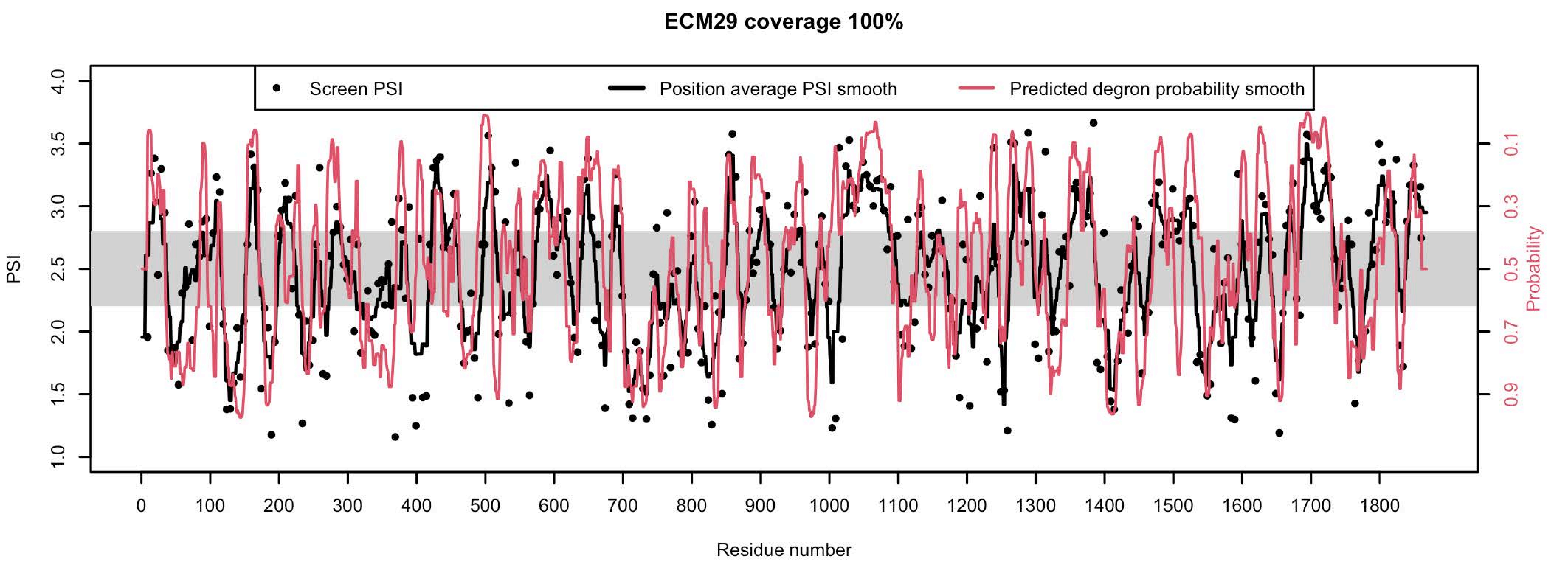

ECM29 coverage 100%
Screen PSI
Position average PSI smooth
Predicted degron probability smooth
PSI
Probability
Residue number
1.0
1.5
2.0
2.5
3.0
3.5
4.0
0.1
0.3
0.5
0.7
0.9
0
100
200
300
400
500
600
700
800
900
1000
1100
1200
1300
1400
1500
1600
1700
1800

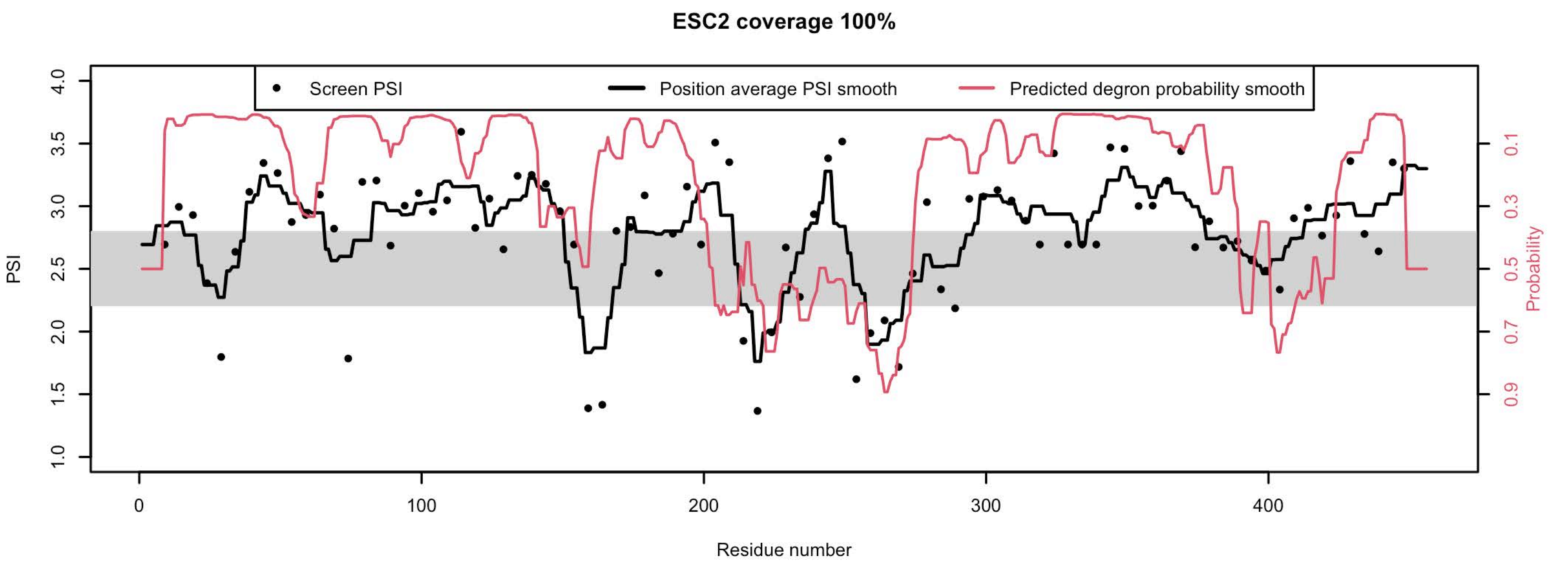

ESC2 coverage 100%
Screen PSI
Position average PSI smooth
Predicted degron probability smooth
PSI
1.0
1.5
2.0
2.5
3.0
3.5
4.0
Probability
0.1
0.3
0.5
0.7
0.9
Residue number
0
100
200
300
400

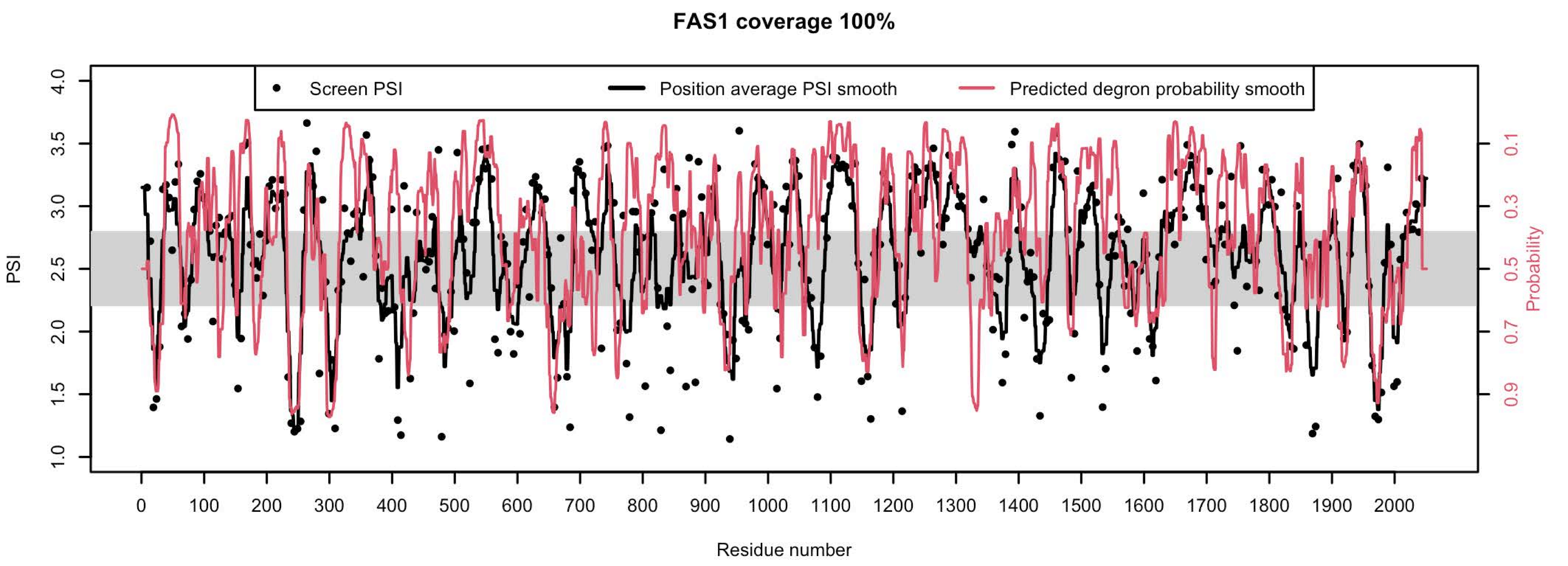
FAS1 coverage 100%
Screen PSI
Position average PSI smooth
Predicted degron probability smooth
PSI
Probability
Residue number
1.0
1.5
2.0
2.5
3.0
3.5
4.0
0.1
0.3
0.5
0.7
0.9
0
100
200
300
400
500
600
700
800
900
1000
1100
1200
1300
1400
1500
1600
1700
1800
1900
2000

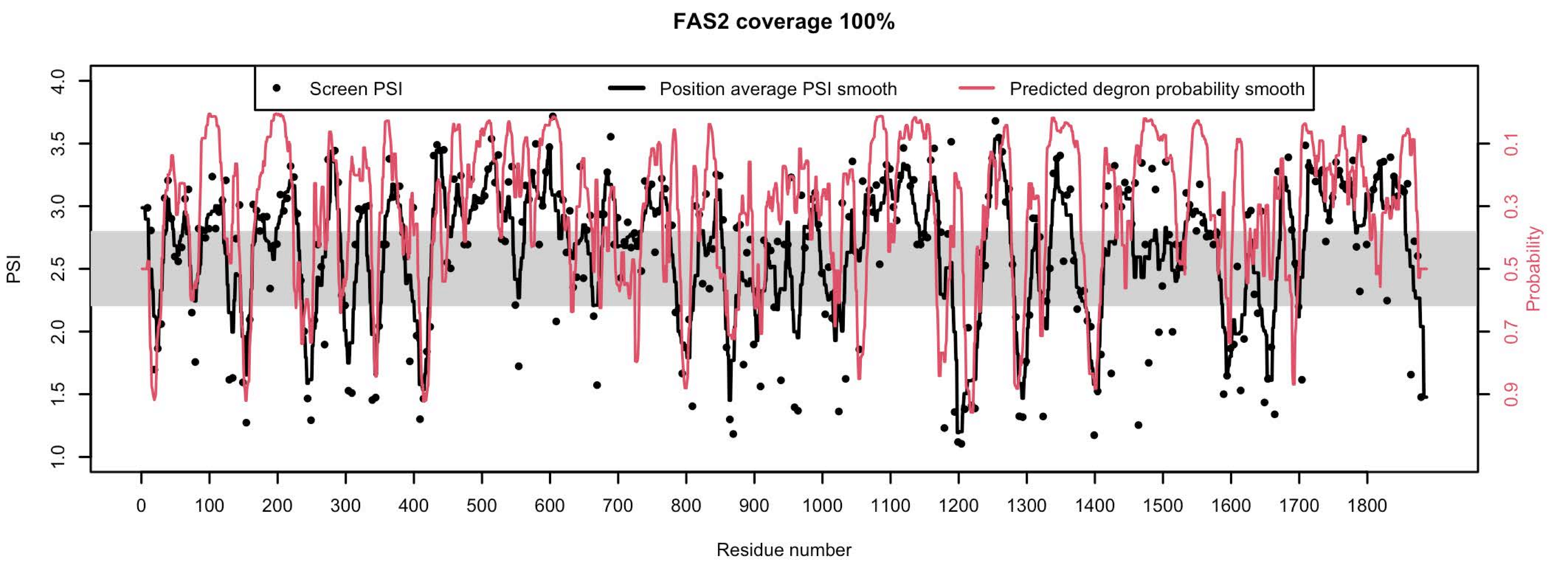

FAS2 coverage 100%
Screen PSI
Position average PSI smooth
Predicted degron probability smooth
PSI
1.0
1.5
2.0
2.5
3.0
3.5
4.0
Probability
0.9
0.7
0.5
0.3
0.1
Residue number
0
100
200
300
400
500
600
700
800
900
1000
1100
1200
1300
1400
1500
1600
1700
1800

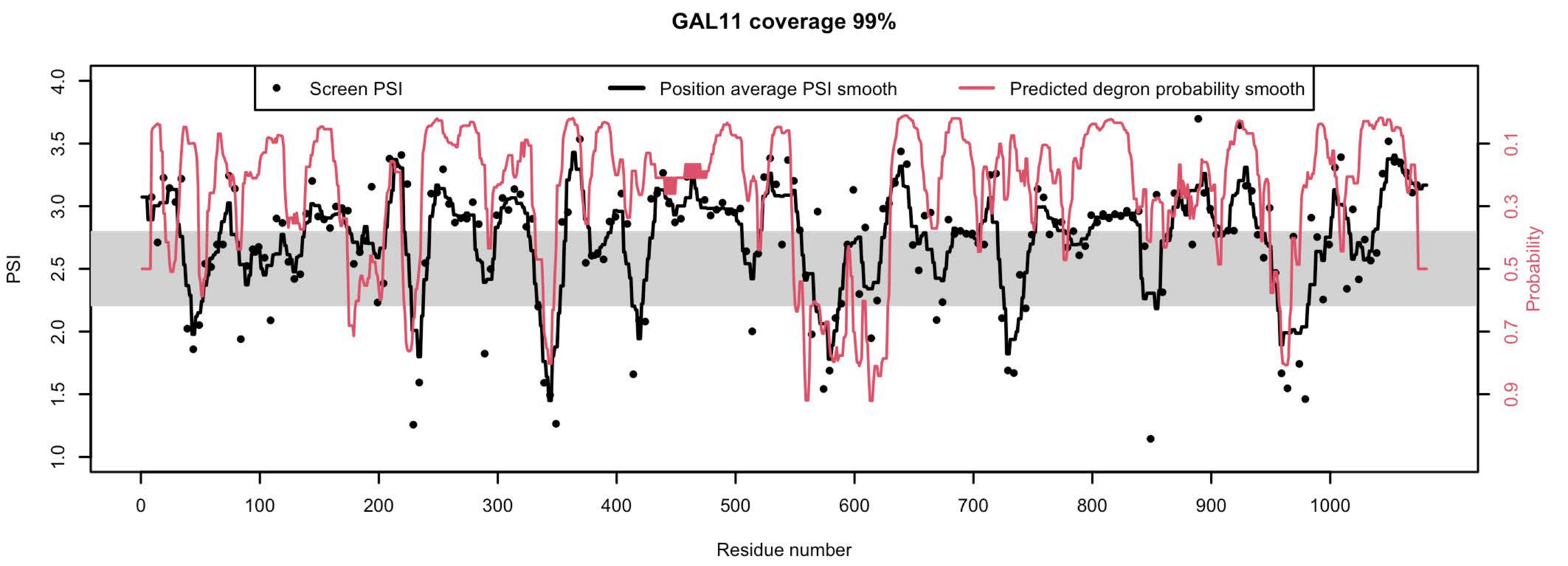
GAL11 coverage 99%
Screen PSI
Position average PSI smooth
Predicted degron probability smooth
PSI
1.0
1.5
2.0
2.5
3.0
3.5
4.0
Probability
0.1
0.3
0.5
0.7
0.9
Residue number
0
100
200
300
400
500
600
700
800
900
1000

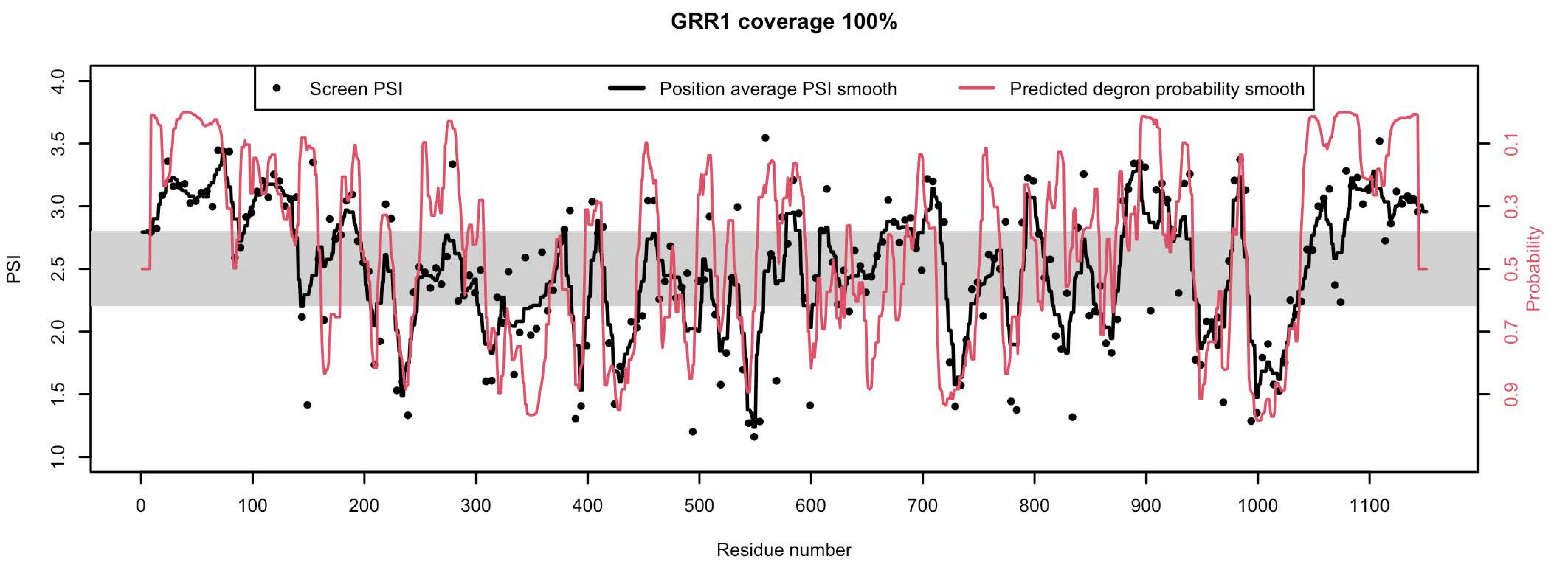
GRR1 coverage 100%
Screen PSI
Position average PSI smooth
Predicted degron probability smooth
PSI
Probability
Residue number
1.0
1.5
2.0
2.5
3.0
3.5
4.0
0.1
0.3
0.5
0.7
0.9
0
100
200
300
400
500
600
700
800
900
1000
1100

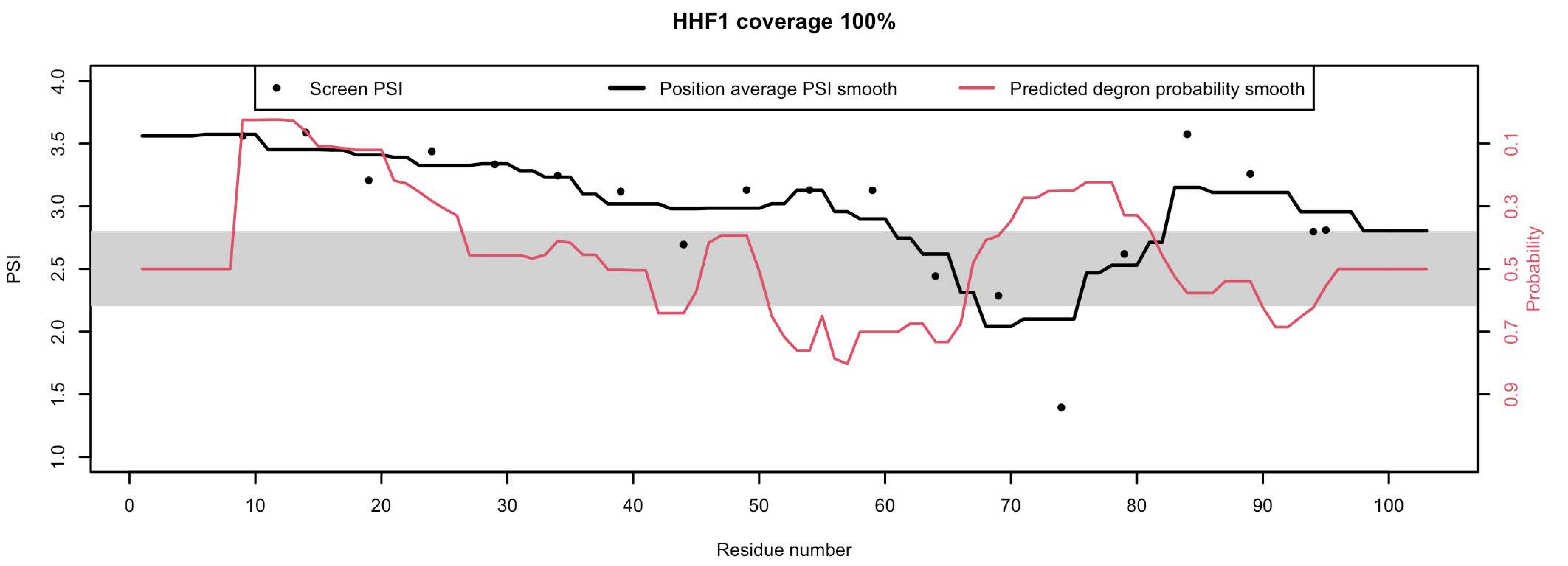

HHF1 coverage 100%
Screen PSI
Position average PSI smooth
Predicted degron probability smooth
PSI
1.0
1.5
2.0
2.5
3.0
3.5
4.0
Probability
0.1
0.3
0.5
0.7
0.9
Residue number
0
10
20
30
40
50
60
70
80
90
100

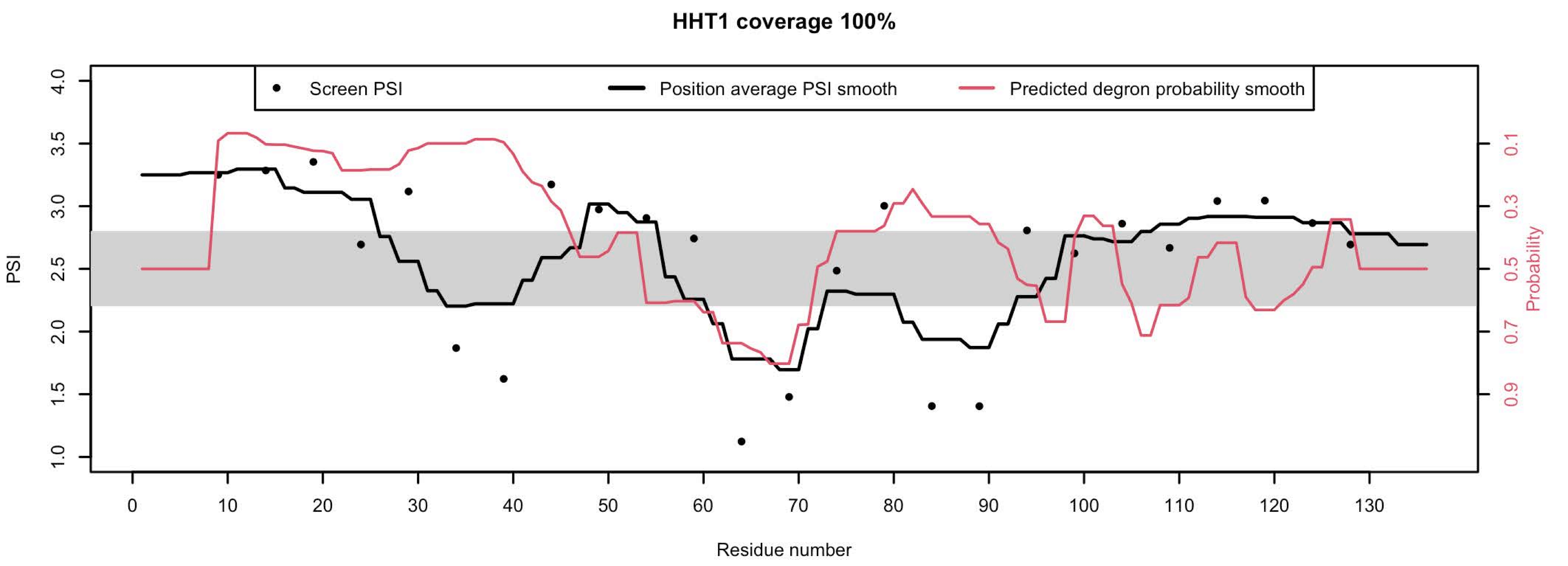

HHT1 coverage 100%
Screen PSI
Position average PSI smooth
Predicted degron probability smooth
PSI
Probability
Residue number

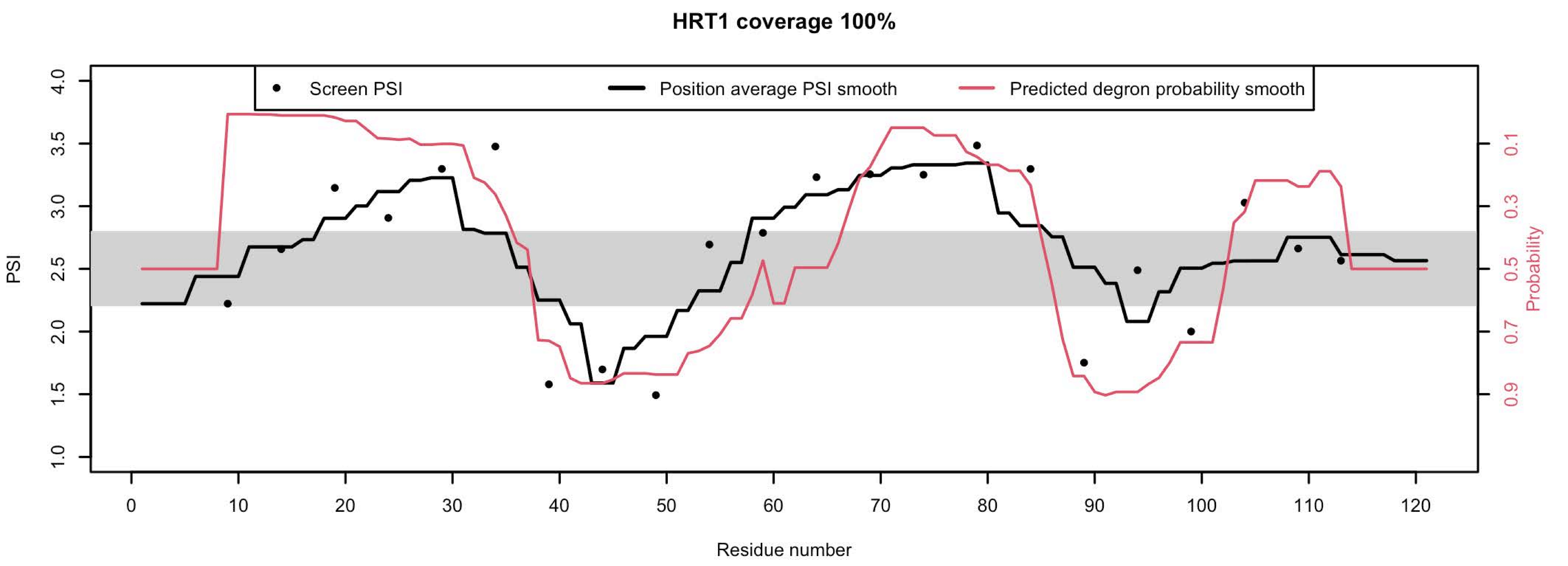

HRT1 coverage 100%
Screen PSI
Position average PSI smooth
Predicted degron probability smooth
PSI
Probability
Residue number
4.0
3.5
3.0
2.5
2.0
1.5
1.0
0.1
0.3
0.5
0.7
0.9
0
10
20
30
40
50
60
70
80
90
100
110
120

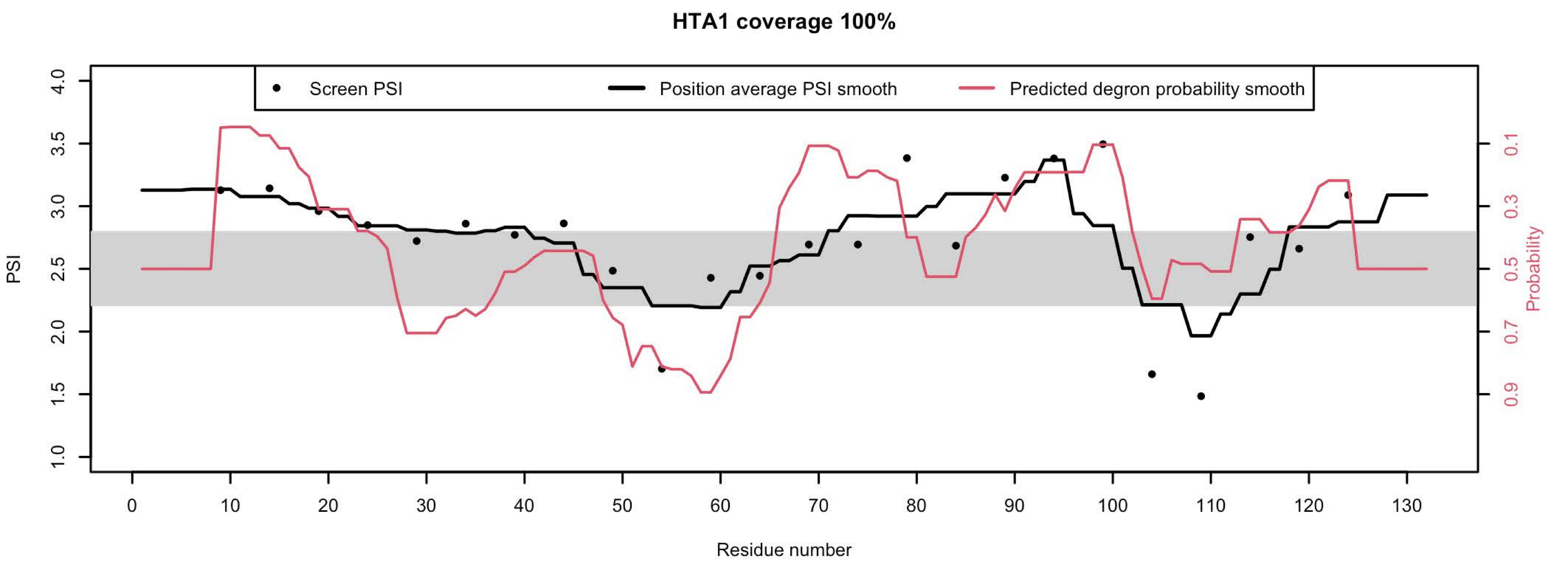

HTA1 coverage 100%
Screen PSI
Position average PSI smooth
Predicted degron probability smooth
PSI
1.0
1.5
2.0
2.5
3.0
3.5
4.0
Probability
0.1
0.3
0.5
0.7
0.9
0
10
20
30
40
50
60
70
80
90
100
110
120
130
Residue number

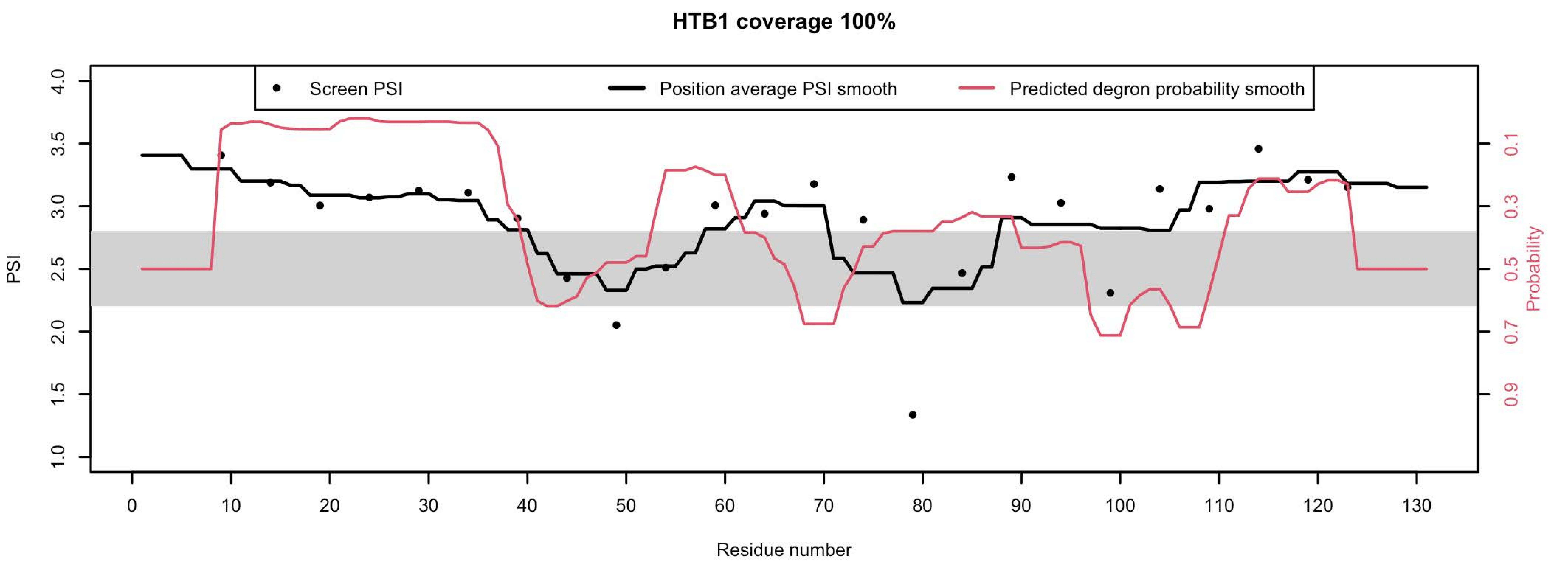
HTB1 coverage 100%
Screen PSI
Position average PSI smooth
Predicted degron probability smooth
PSI
Probability
Residue number
4.0
3.5
3.0
2.5
2.0
1.5
1.0
0.1
0.3
0.5
0.7
0.9
0
10
20
30
40
50
60
70
80
90
100
110
120
130

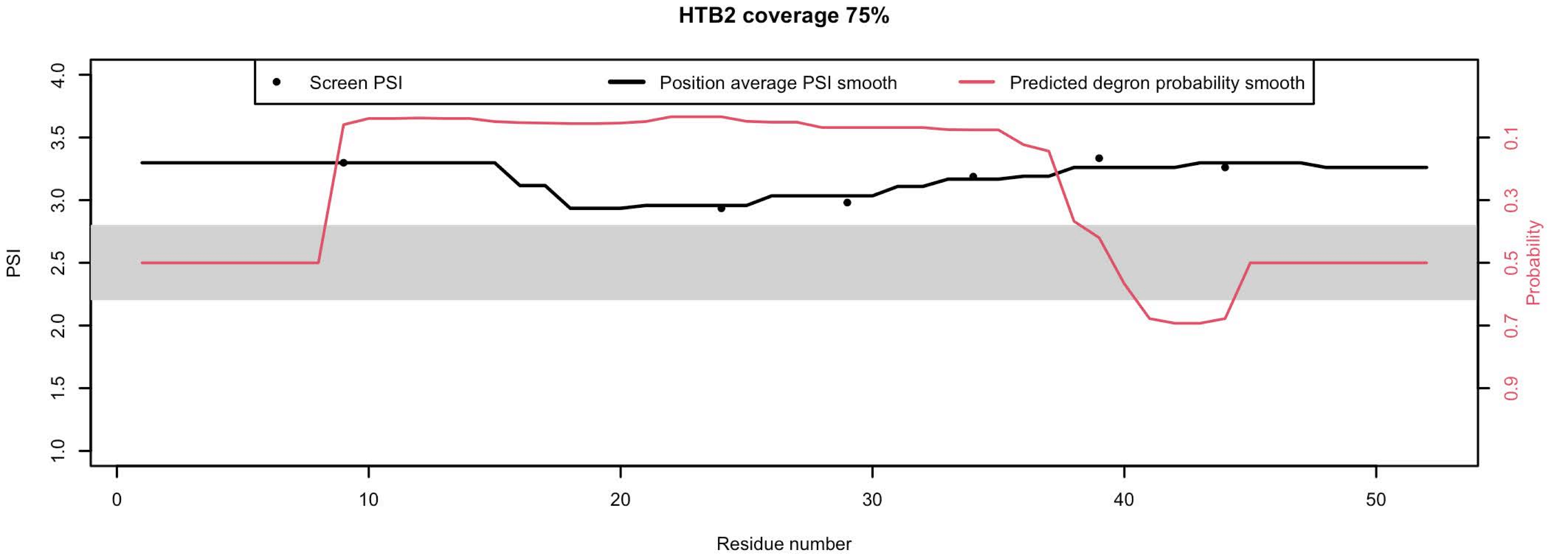
HTB2 coverage 75%
Screen PSI
Position average PSI smooth
Predicted degron probability smooth
PSI
4.0
3.5
3.0
2.5
2.0
1.5
1.0
Probability
0.1
0.3
0.5
0.7
0.9
0
10
20
30
40
50
Residue number

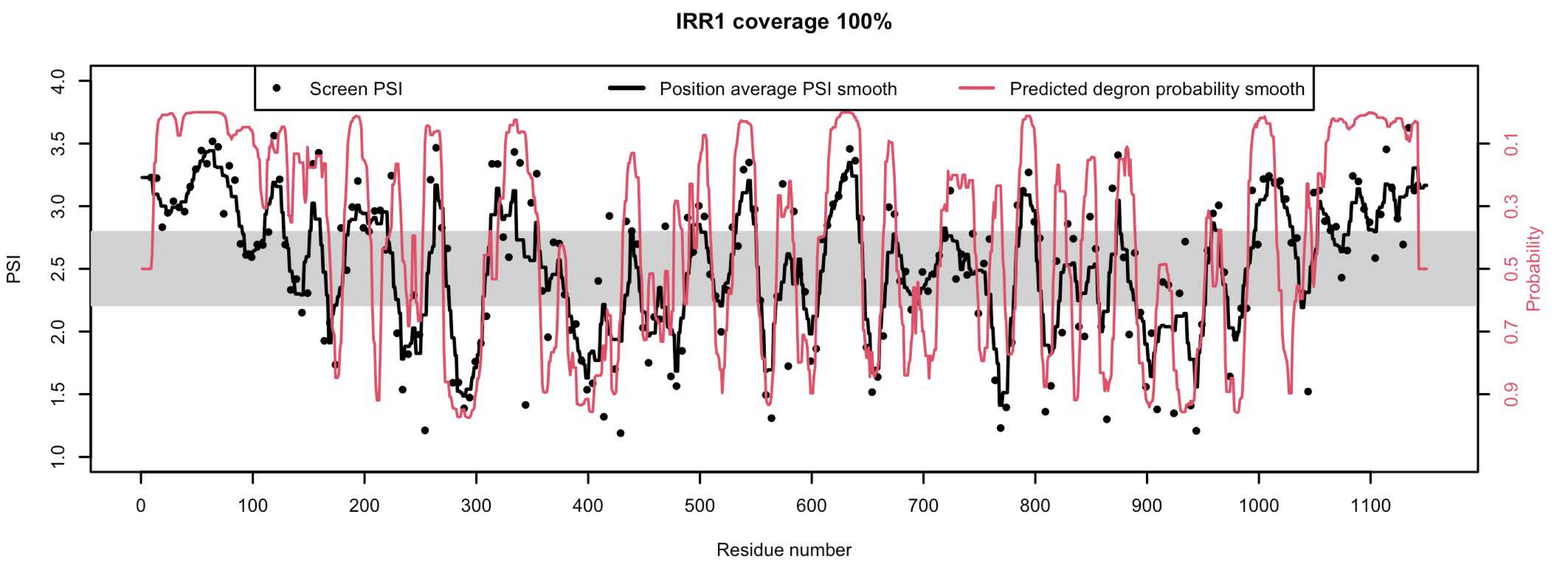
IRR1 coverage 100%
Screen PSI
Position average PSI smooth
Predicted degron probability smooth
PSI
Probability
Residue number
1.0
1.5
2.0
2.5
3.0
3.5
4.0
0.1
0.3
0.5
0.7
0.9
0
100
200
300
400
500
600
700
800
900
1000
1100

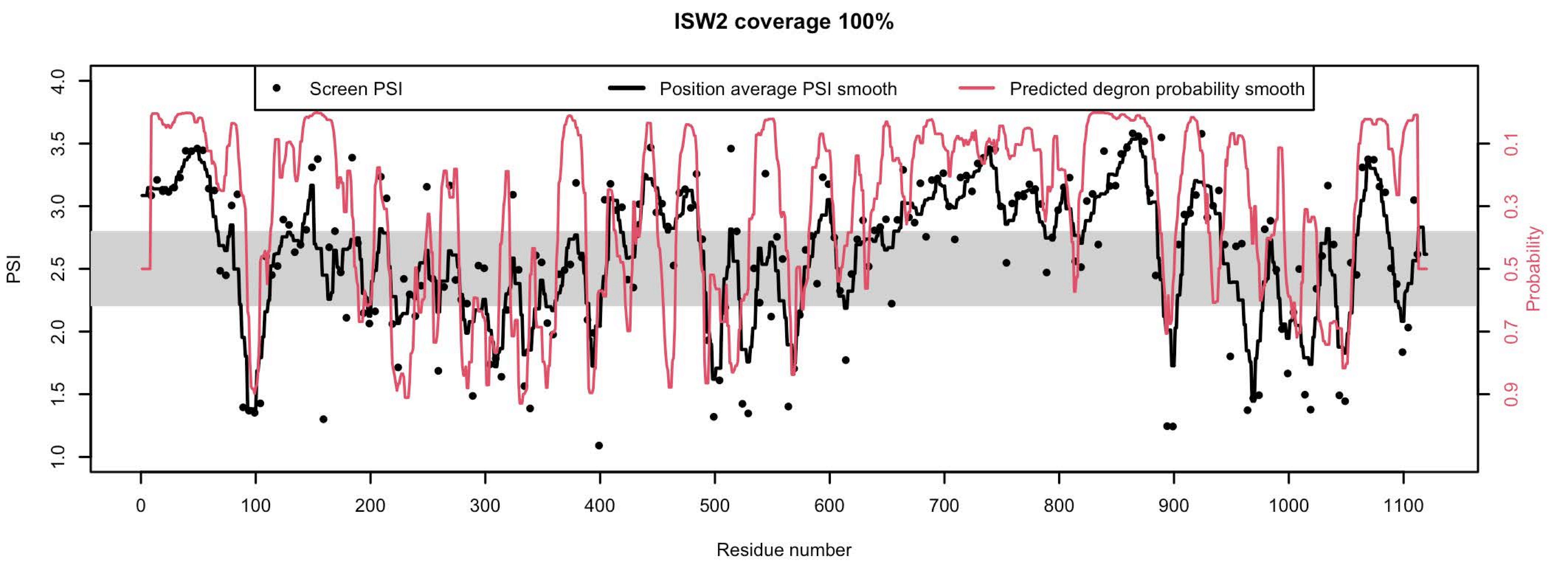
ISW2 coverage 100%
Screen PSI
Position average PSI smooth
Predicted degron probability smooth
PSI
1.0
1.5
2.0
2.5
3.0
3.5
4.0
Probability
0.1
0.3
0.5
0.7
0.9
Residue number
0
100
200
300
400
500
600
700
800
900
1000
1100

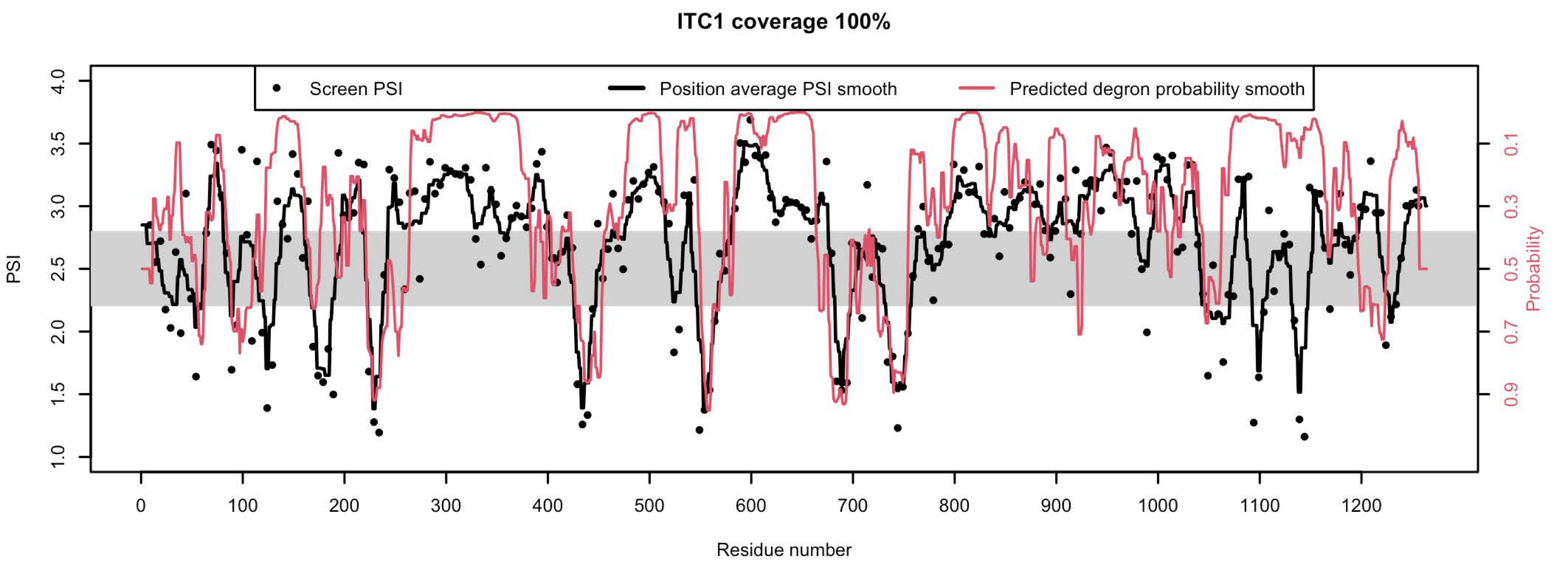

ITC1 coverage 100%
Screen PSI
Position average PSI smooth
Predicted degron probability smooth
PSI
1.0
1.5
2.0
2.5
3.0
3.5
4.0
Probability
0.1
0.3
0.5
0.7
0.9
Residue number
0
100
200
300
400
500
600
700
800
900
1000
1100
1200

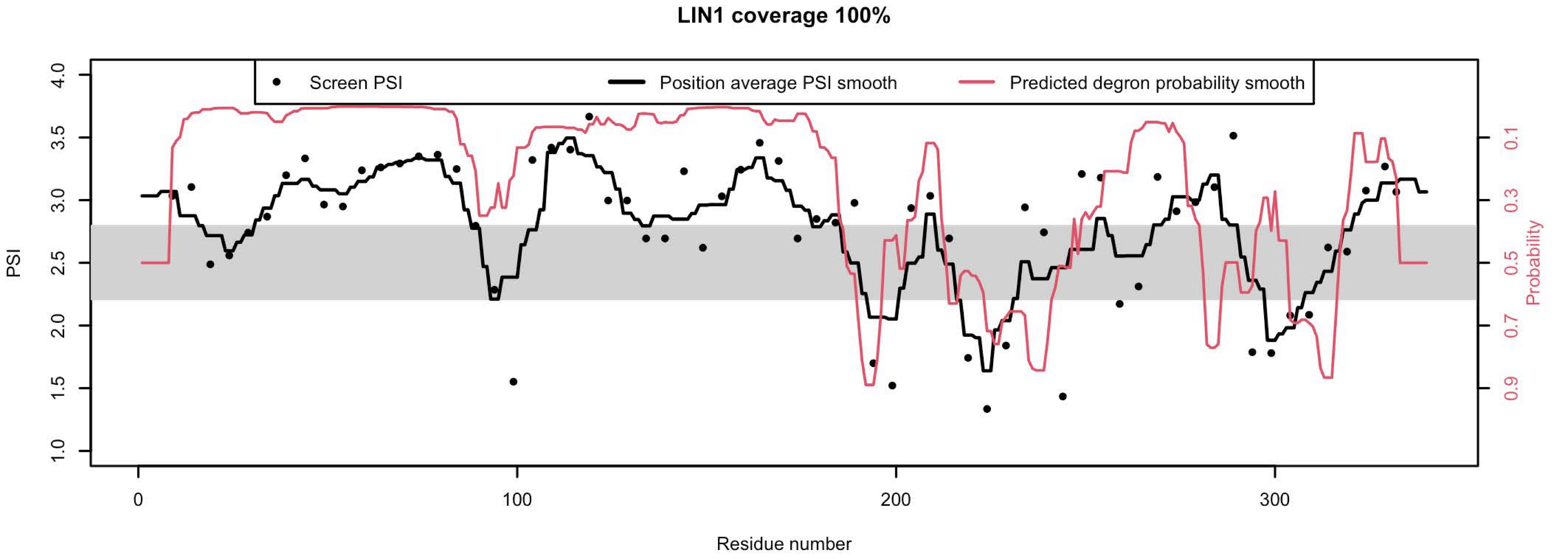

LIN1 coverage 100%
Screen PSI
Position average PSI smooth
Predicted degron probability smooth
PSI
Probability
Residue number
1.0
1.5
2.0
2.5
3.0
3.5
4.0
0.1
0.3
0.5
0.7
0.9
0
100
200
300

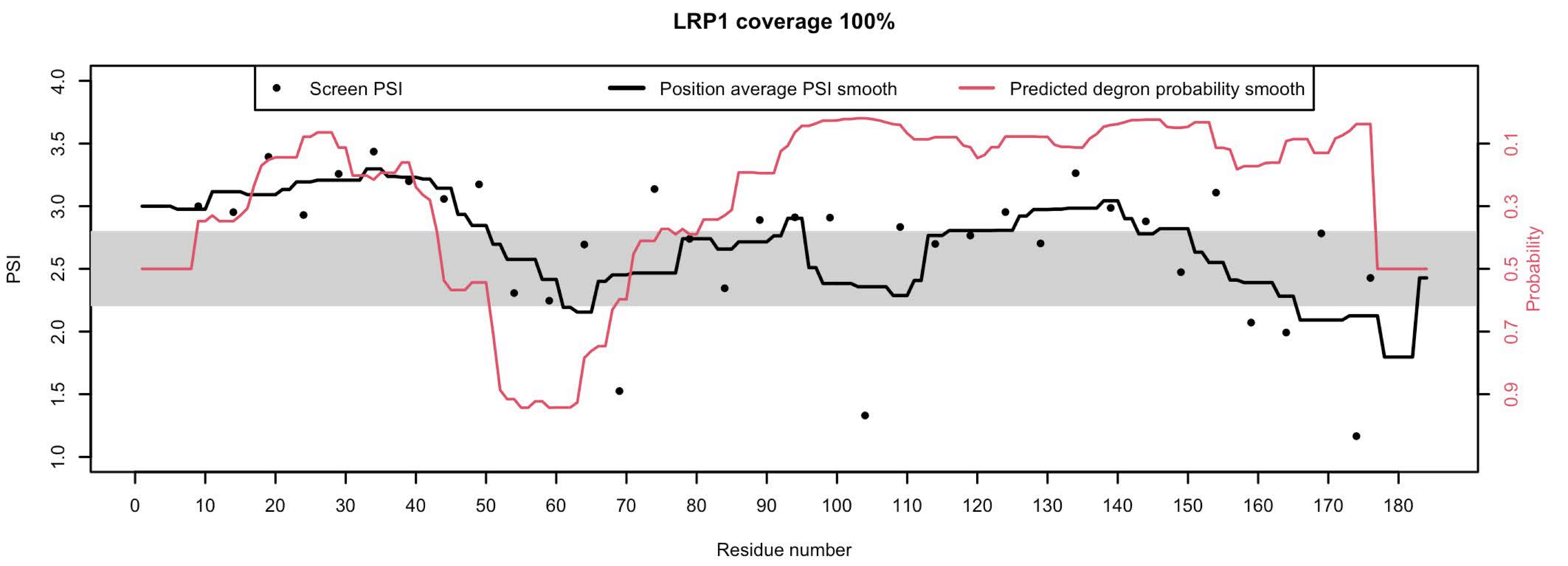
LRP1 coverage 100%
Screen PSI
Position average PSI smooth
Predicted degron probability smooth
PSI
1.0
1.5
2.0
2.5
3.0
3.5
4.0
Probability
0.9
0.7
0.5
0.3
0.1
Residue number
0
10
20
30
40
50
60
70
80
90
100
110
120
130
140
150
160
170
180

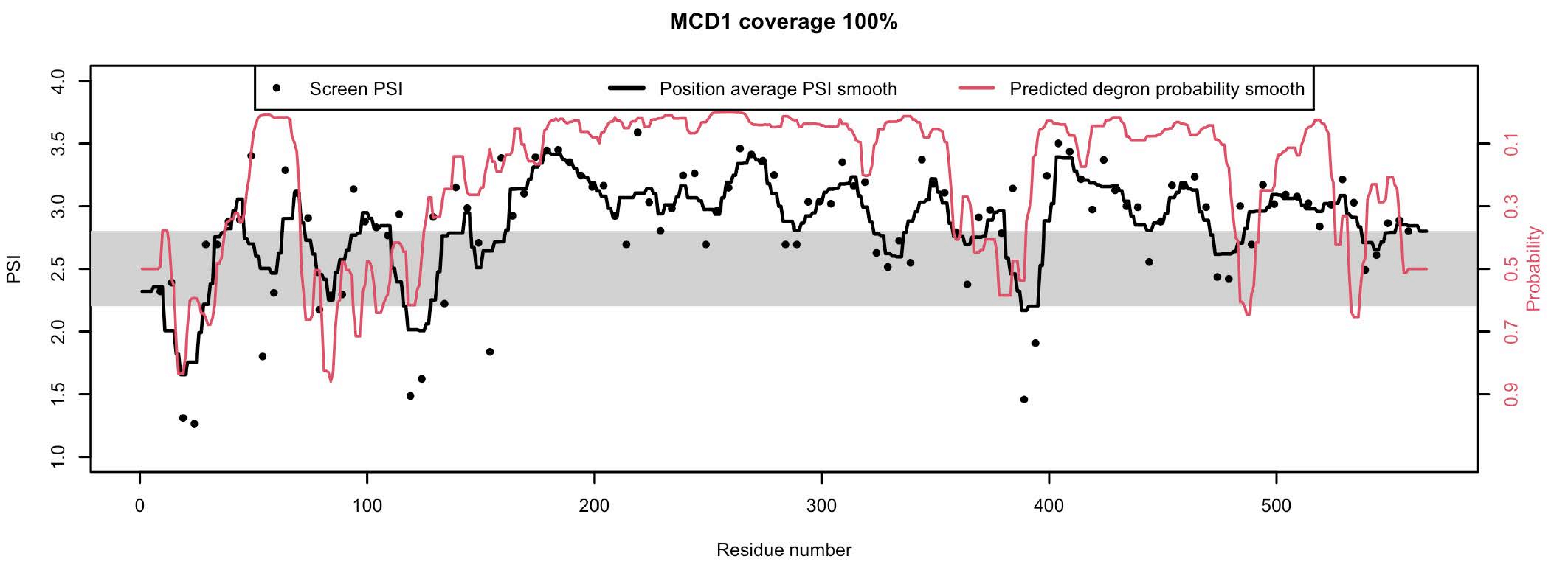

MCD1 coverage 100%
Screen PSI
Position average PSI smooth
Predicted degron probability smooth
PSI
Probability
Residue number
1.0
1.5
2.0
2.5
3.0
3.5
4.0
0.1
0.3
0.5
0.7
0.9
0
100
200
300
400
500

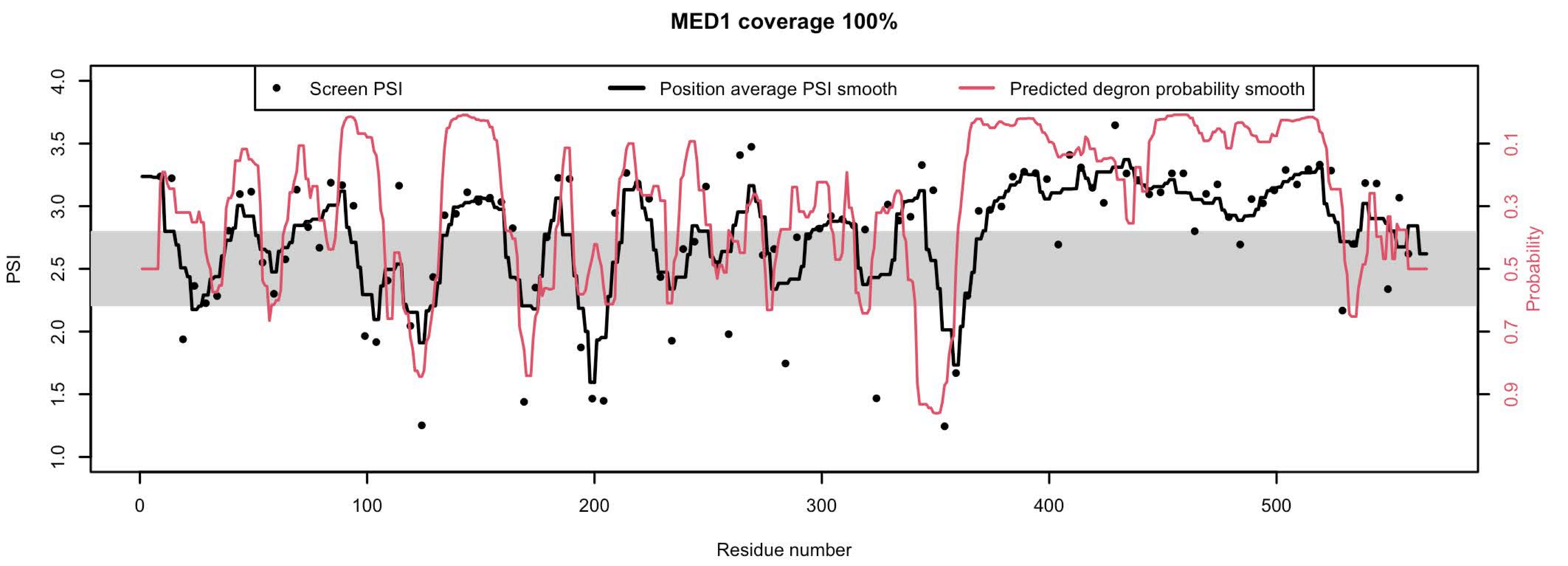
MED1 coverage 100%
Screen PSI
Position average PSI smooth
Predicted degron probability smooth
PSI
Probability
Residue number
1.0
1.5
2.0
2.5
3.0
3.5
4.0
0.1
0.3
0.5
0.7
0.9
0
100
200
300
400
500

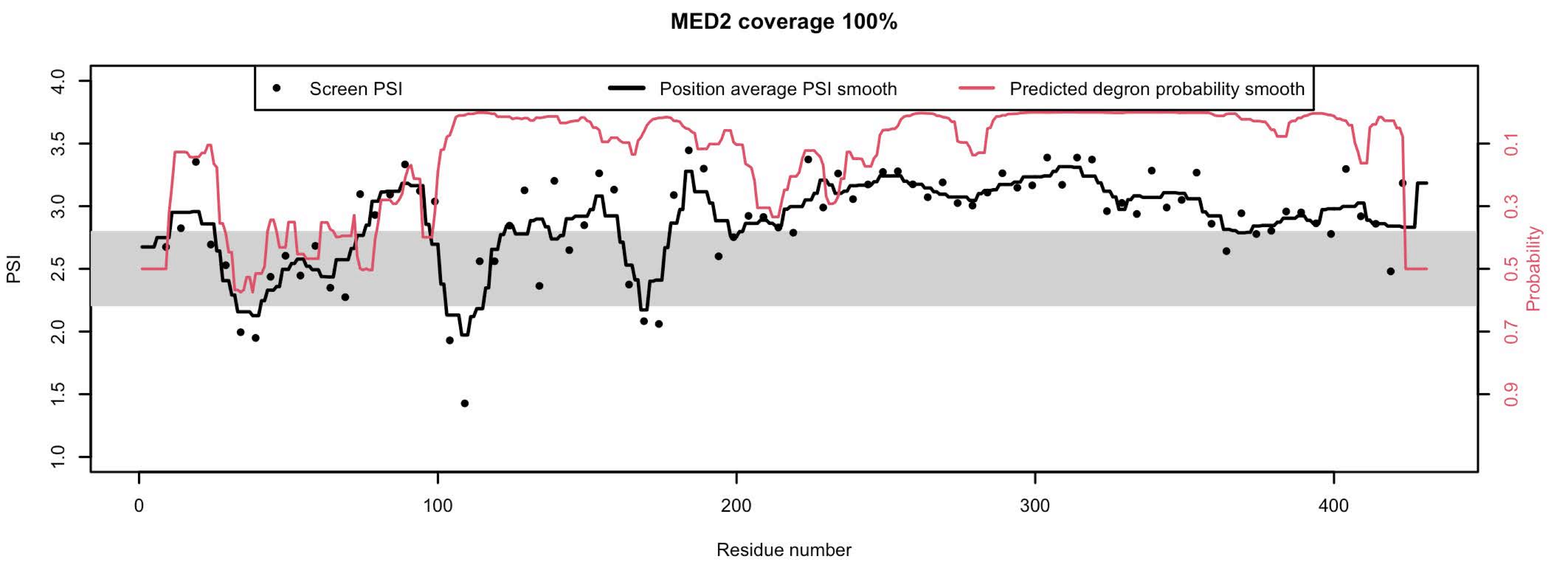

MED2 coverage 100%
Screen PSI
Position average PSI smooth
Predicted degron probability smooth
PSI
Probability
Residue number
1.0
1.5
2.0
2.5
3.0
3.5
4.0
0.1
0.3
0.5
0.7
0.9
0
100
200
300
400

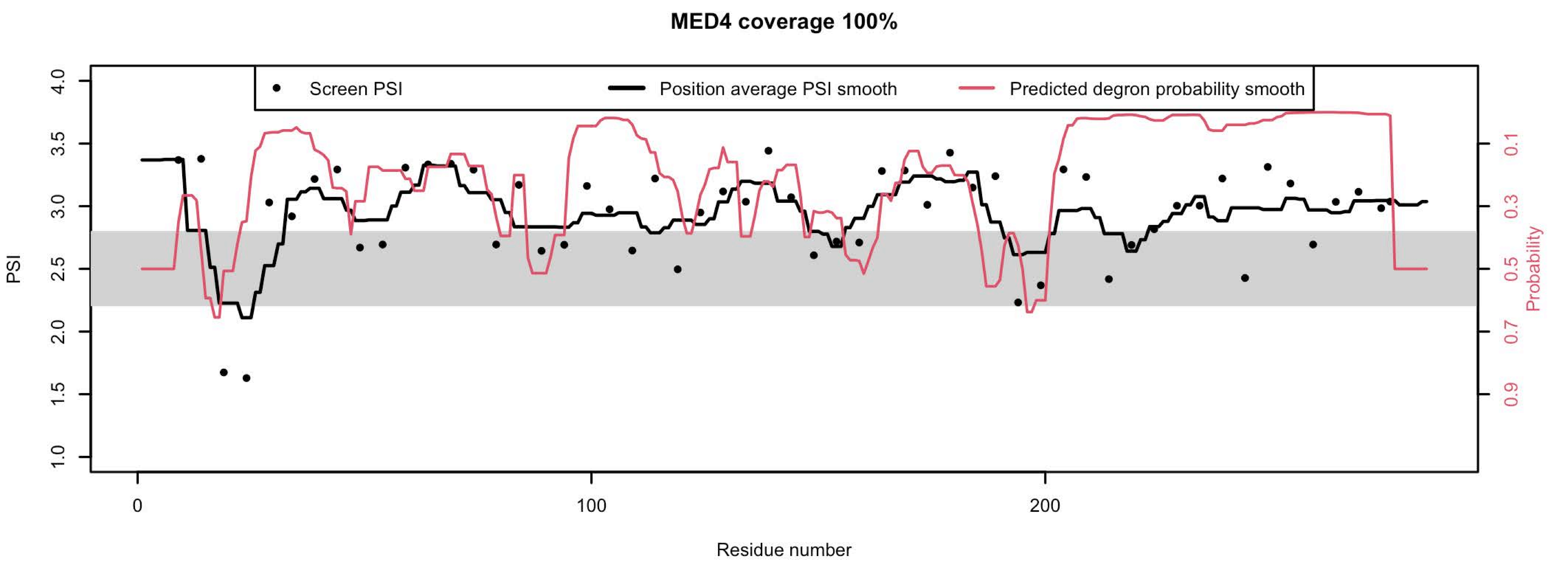

MED4 coverage 100%
Screen PSI
Position average PSI smooth
Predicted degron probability smooth
PSI
1.0
1.5
2.0
2.5
3.0
3.5
4.0
Probability
0.1
0.3
0.5
0.7
0.9
0
100
200
Residue number

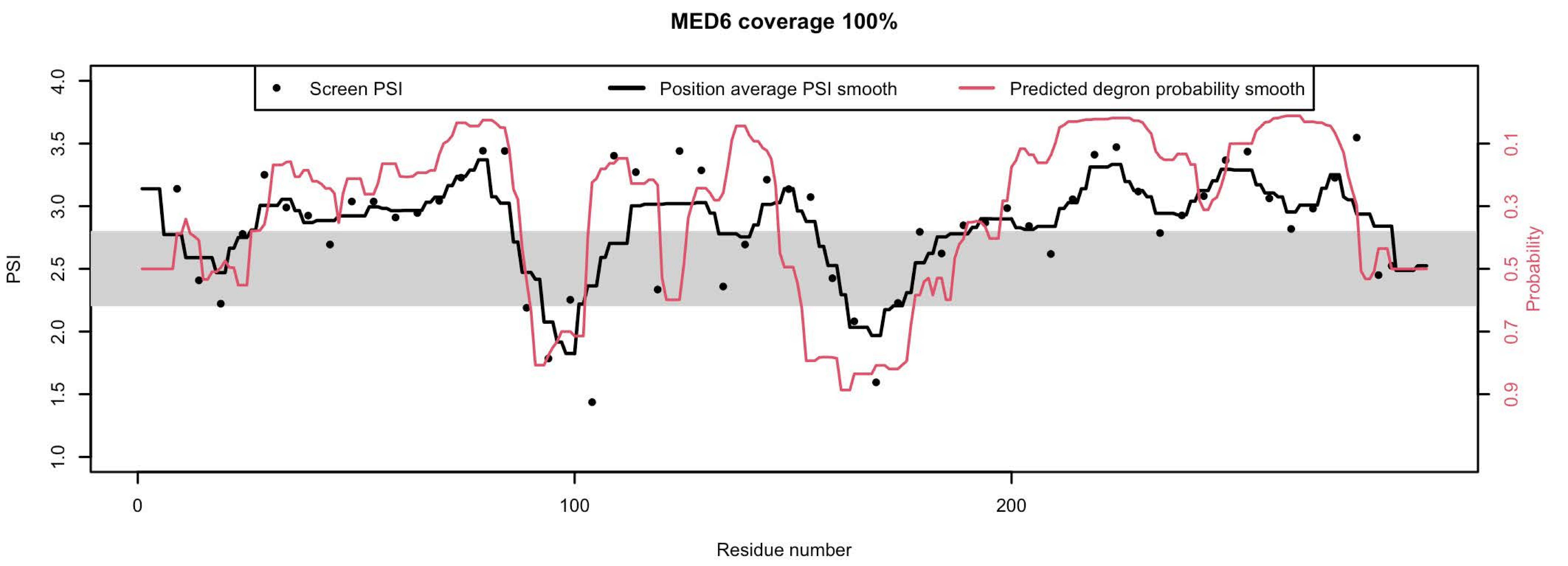

MED6 coverage 100%
Screen PSI
Position average PSI smooth
Predicted degron probability smooth
PSI
Probability
Residue number
1.0
1.5
2.0
2.5
3.0
3.5
4.0
0.1
0.3
0.5
0.7
0.9
0
100
200

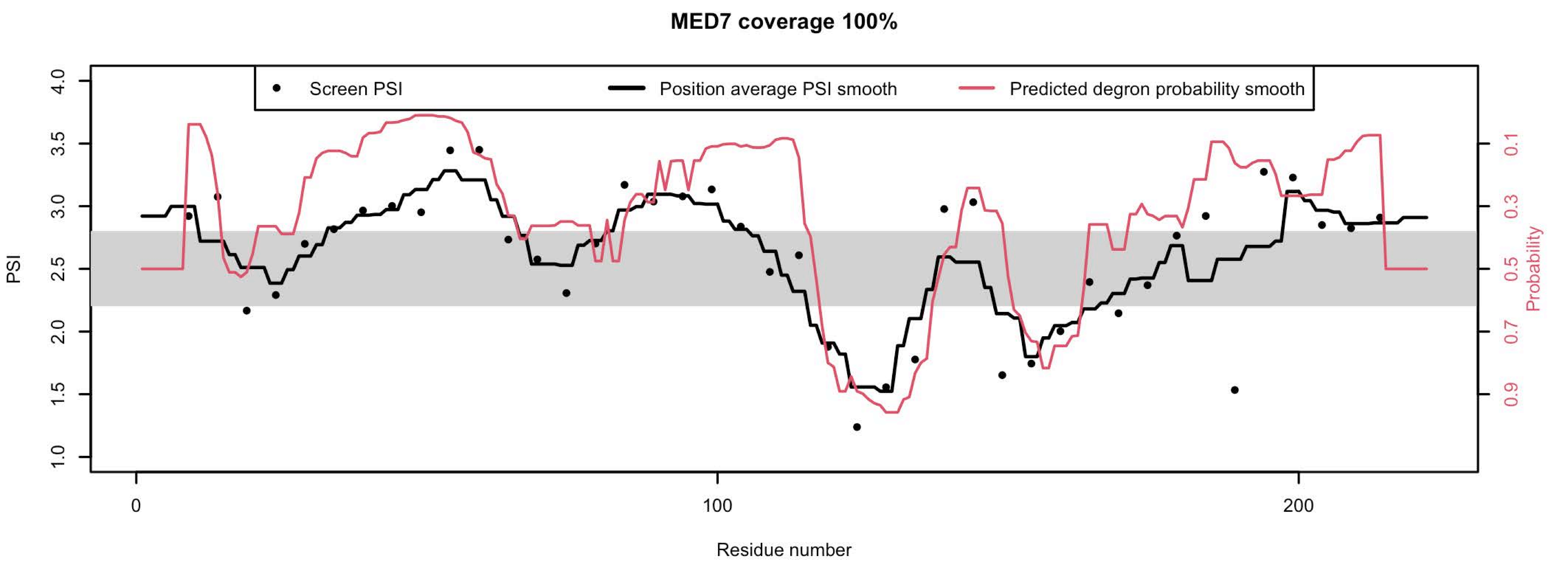

MED7 coverage 100%
Screen PSI
Position average PSI smooth
Predicted degron probability smooth
PSI
Probability
Residue number
1.0
1.5
2.0
2.5
3.0
3.5
4.0
0.1
0.3
0.5
0.7
0.9
0
100
200

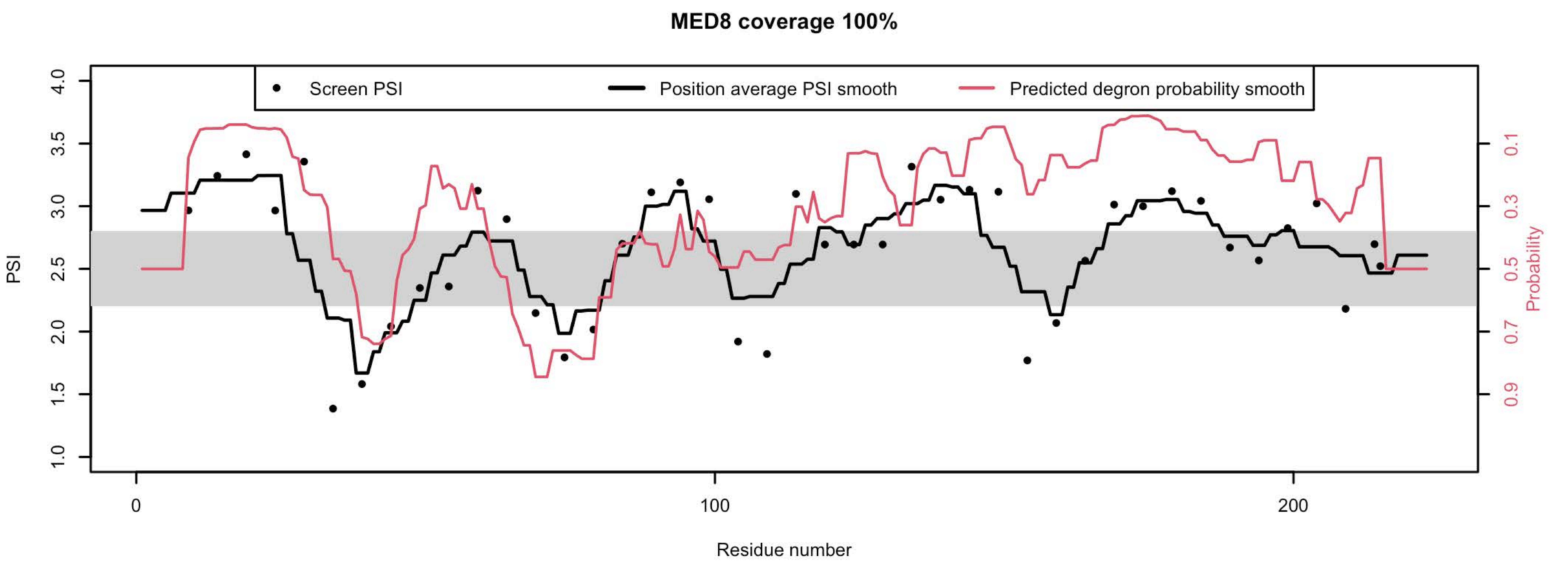
MED8 coverage 100%
Screen PSI
Position average PSI smooth
Predicted degron probability smooth
PSI
Probability
Residue number
1.0
1.5
2.0
2.5
3.0
3.5
4.0
0.1
0.3
0.5
0.7
0.9
0
100
200

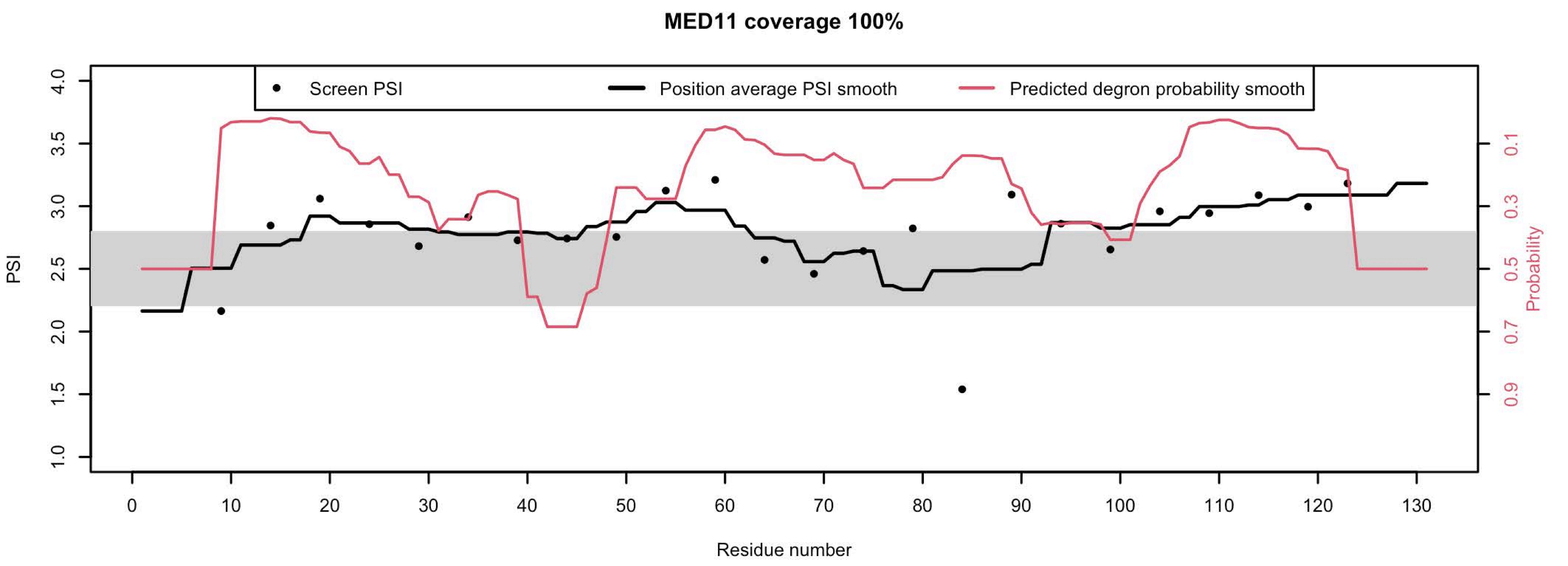
MED11 coverage 100%
Screen PSI
Position average PSI smooth
Predicted degron probability smooth
PSI
1.0
1.5
2.0
2.5
3.0
3.5
4.0
Probability
0.1
0.3
0.5
0.7
0.9
0
10
20
30
40
50
60
70
80
90
100
110
120
130
Residue number

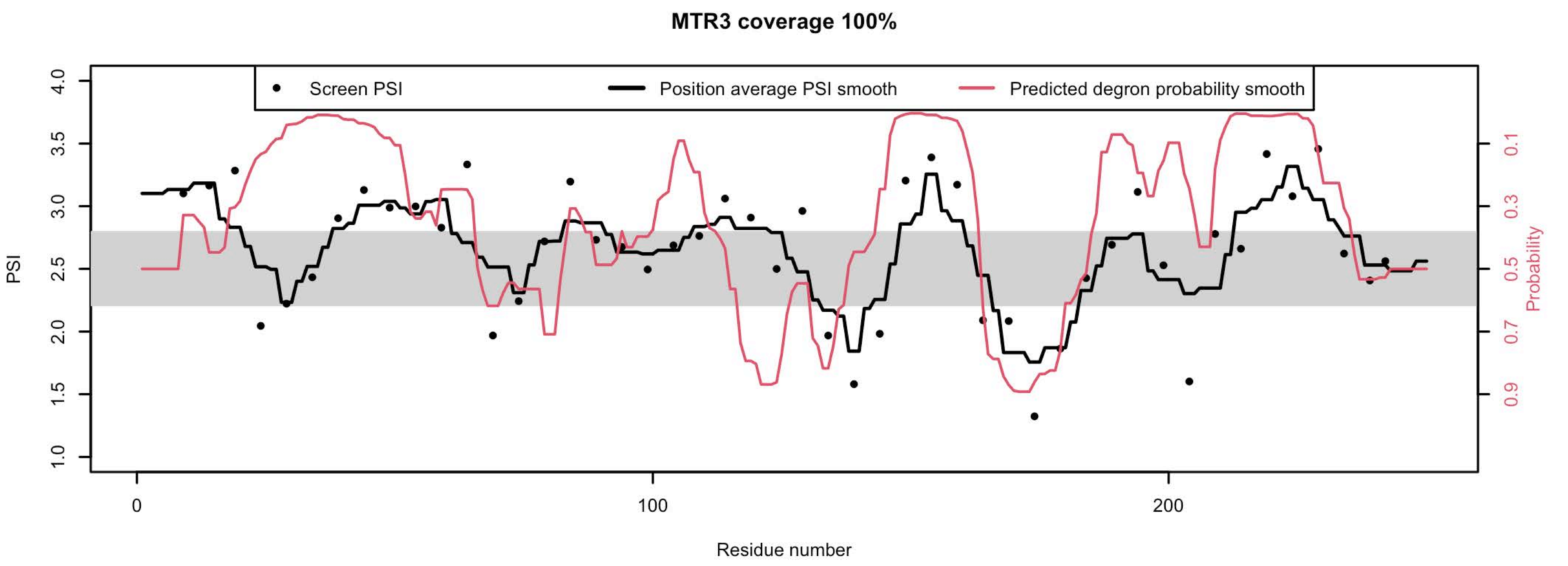

MTR3 coverage 100%
Screen PSI
Position average PSI smooth
Predicted degron probability smooth
PSI
1.0
1.5
2.0
2.5
3.0
3.5
4.0
0.1
0.3
0.5
0.7
0.9
Probability
0
100
200
Residue number

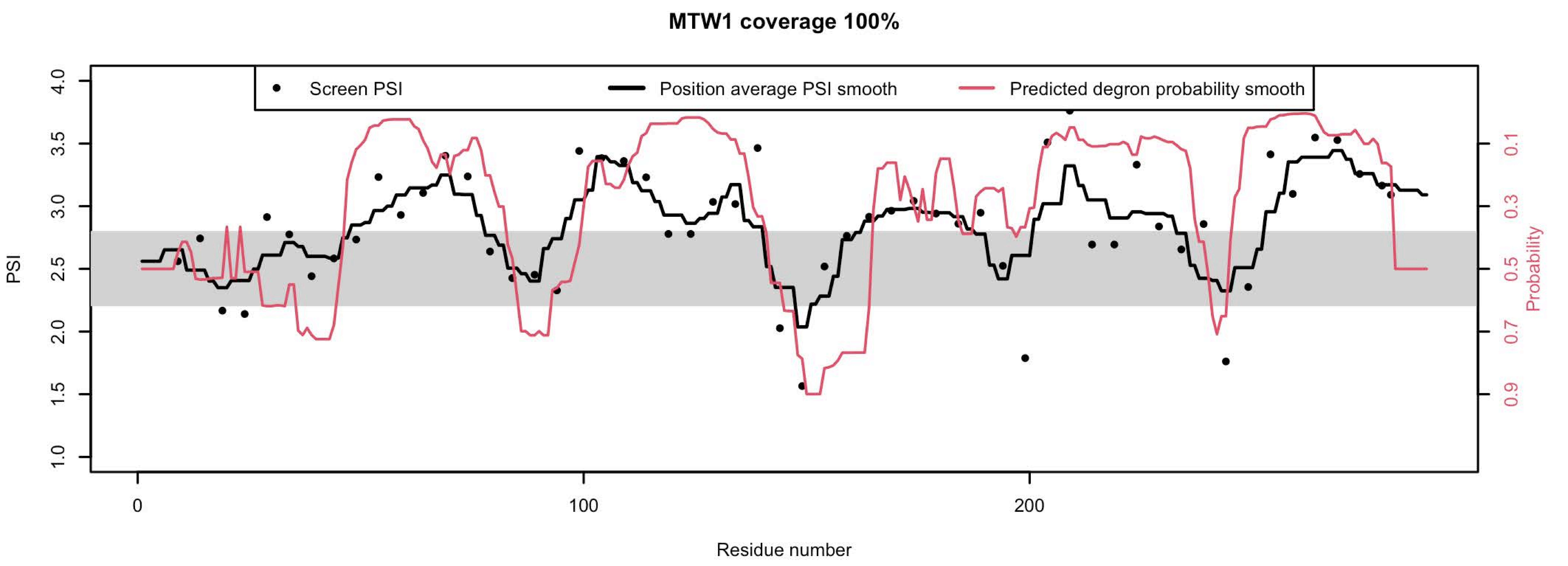
MTW1 coverage 100%
Screen PSI
Position average PSI smooth
Predicted degron probability smooth
PSI
Probability
Residue number
1.0
1.5
2.0
2.5
3.0
3.5
4.0
0.1
0.3
0.5
0.7
0.9
0
100
200

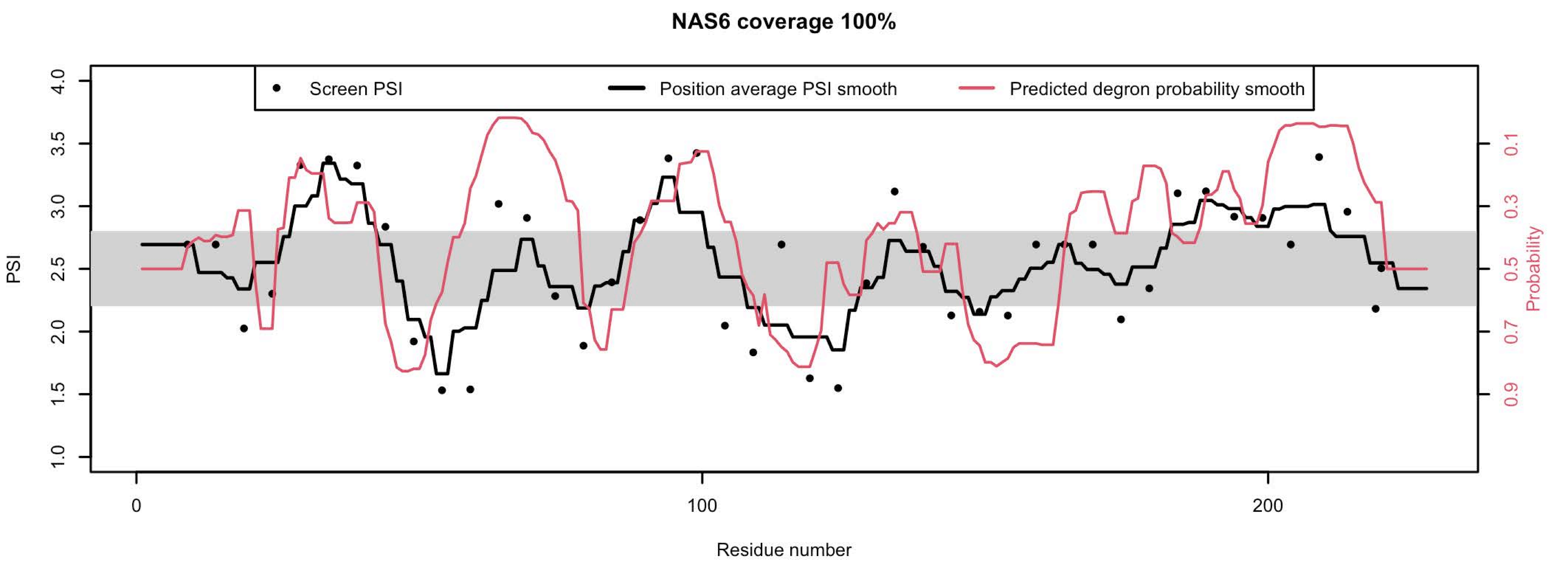

NAS6 coverage 100%
Screen PSI
Position average PSI smooth
Predicted degron probability smooth
PSI
Probability
Residue number
4.0
3.5
3.0
2.5
2.0
1.5
1.0
0.1
0.3
0.5
0.7
0.9
0
100
200

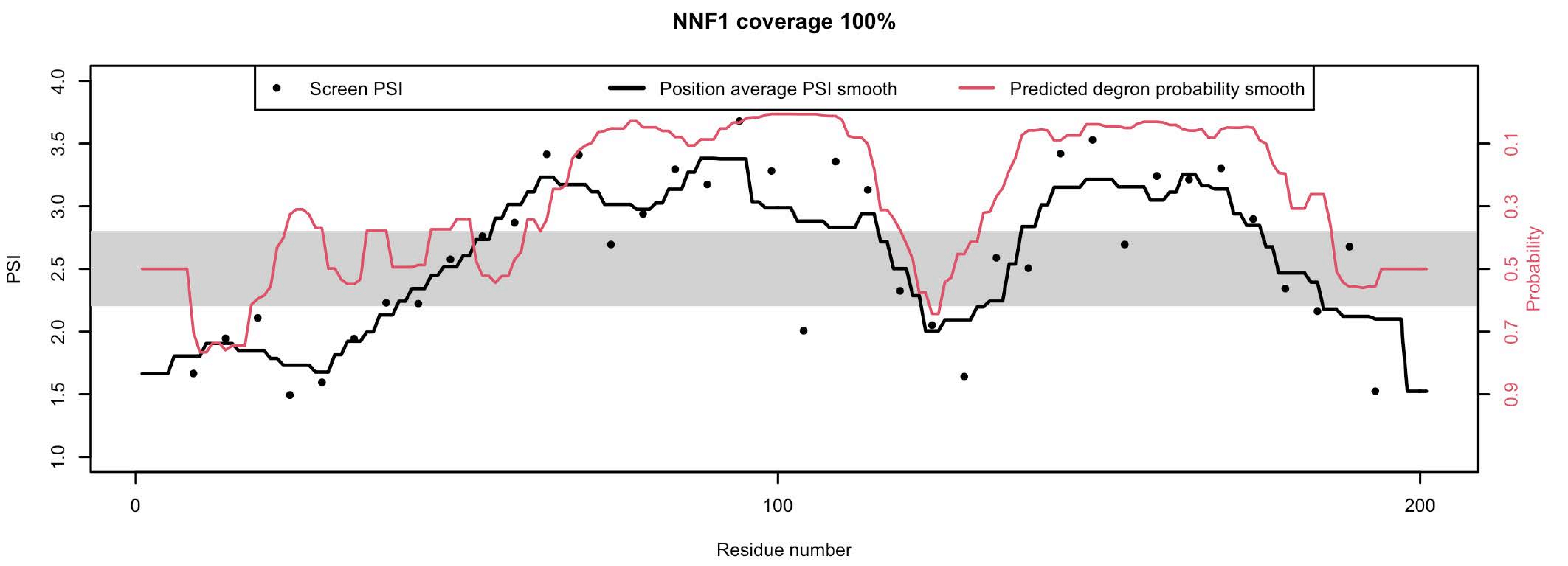
NNF1 coverage 100%
Screen PSI
Position average PSI smooth
Predicted degron probability smooth
PSI
1.0
1.5
2.0
2.5
3.0
3.5
4.0
Probability
0.1
0.3
0.5
0.7
0.9
0
100
200
Residue number

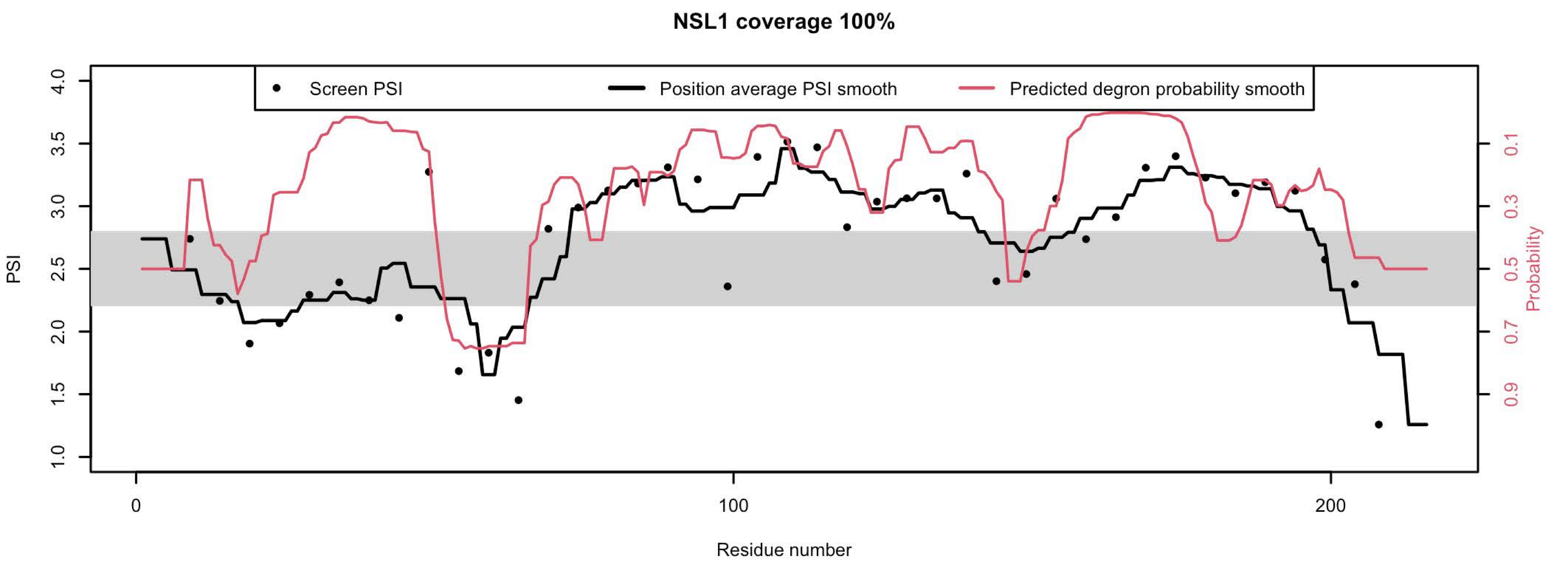

NSL1 coverage 100%
Screen PSI
Position average PSI smooth
Predicted degron probability smooth
PSI
1.0
1.5
2.0
2.5
3.0
3.5
4.0
Probability
0.1
0.3
0.5
0.7
0.9
0
100
200
Residue number

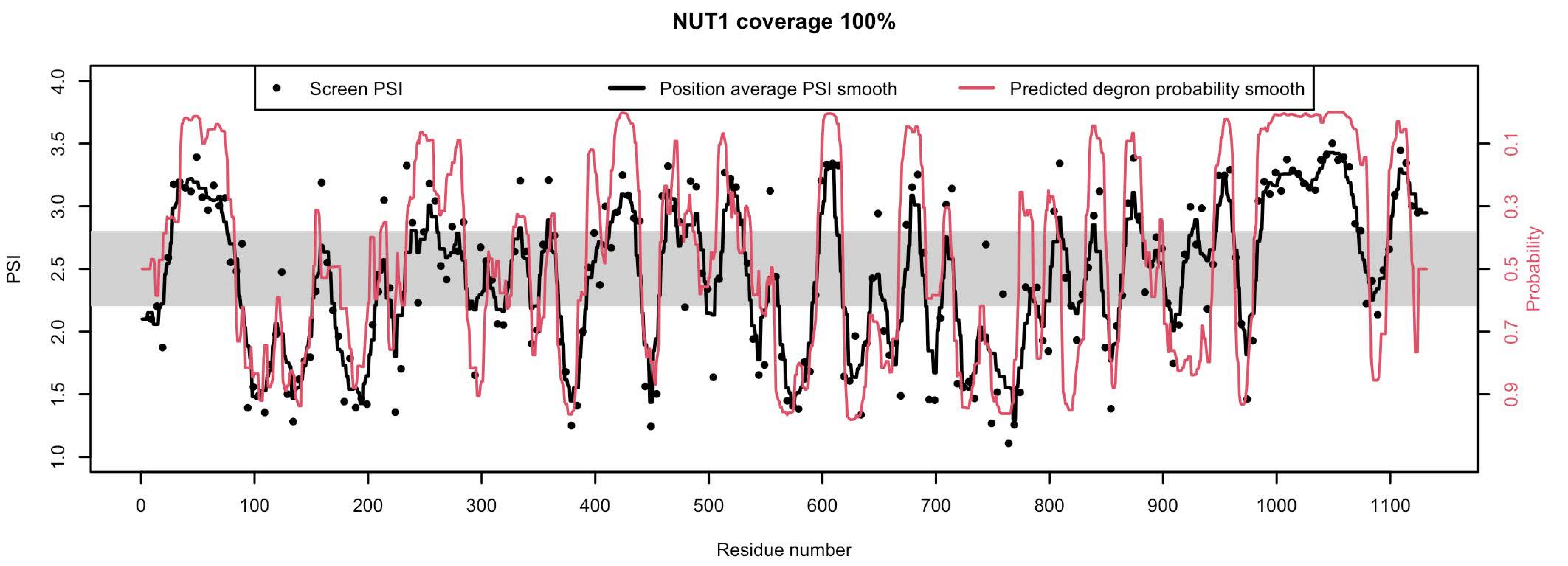
NUT1 coverage 100%
Screen PSI
Position average PSI smooth
Predicted degron probability smooth
PSI
Probability
Residue number
1.0
1.5
2.0
2.5
3.0
3.5
4.0
0.1
0.3
0.5
0.7
0.9
0
100
200
300
400
500
600
700
800
900
1000
1100

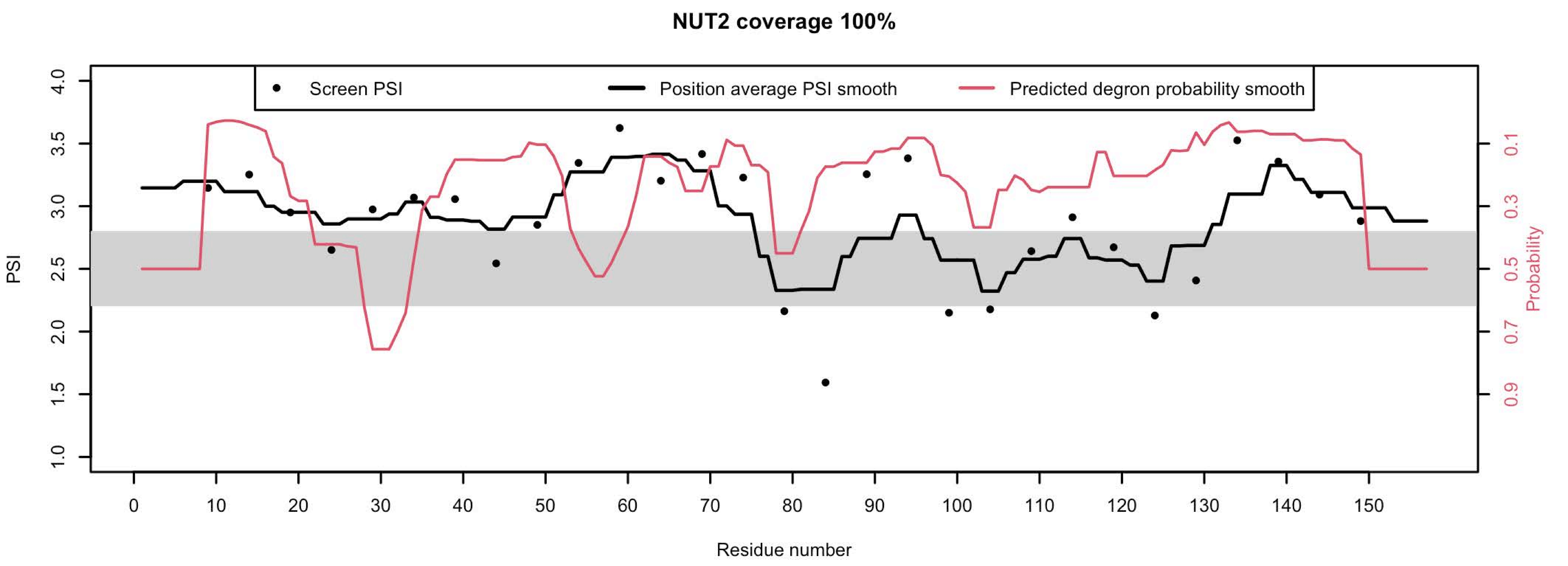
NUT2 coverage 100%
Screen PSI
Position average PSI smooth
Predicted degron probability smooth
PSI
1.0
1.5
2.0
2.5
3.0
3.5
4.0
Probability
0.1
0.3
0.5
0.7
0.9
0
10
20
30
40
50
60
70
80
90
100
110
120
130
140
150
Residue number

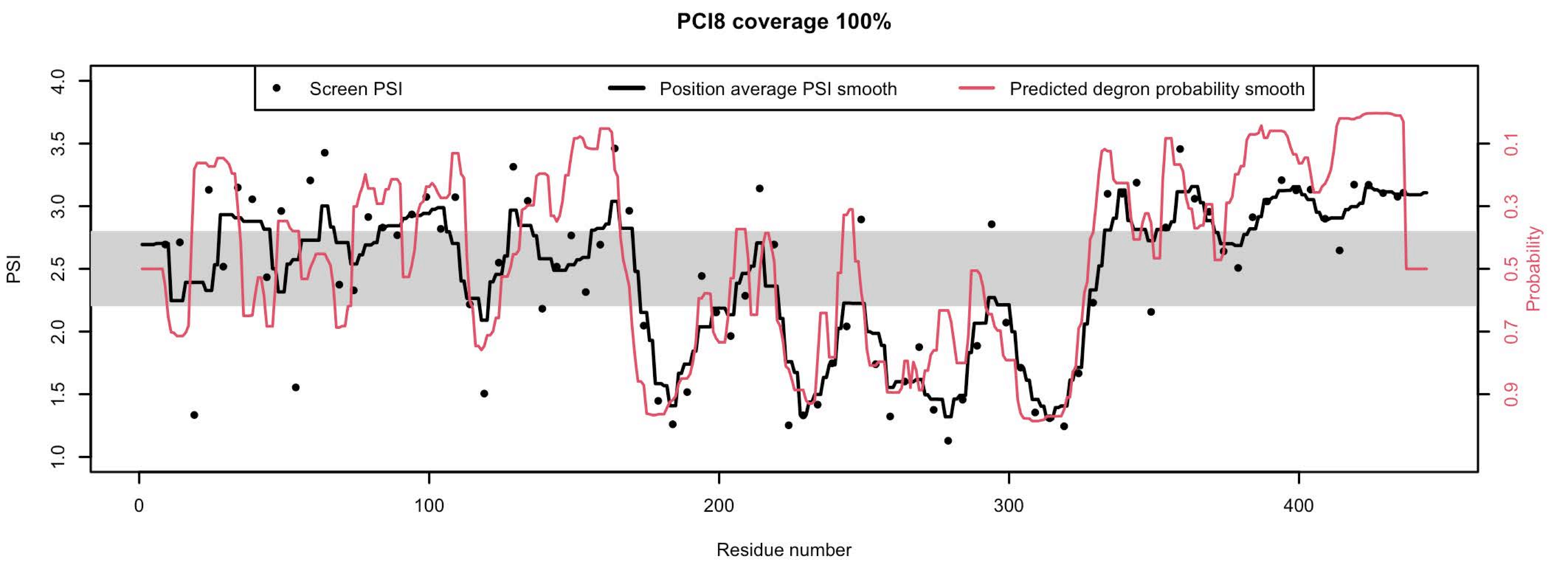
PCI8 coverage 100%
Screen PSI
Position average PSI smooth
Predicted degron probability smooth
PSI
Probability
Residue number
1.0
1.5
2.0
2.5
3.0
3.5
4.0
0.1
0.3
0.5
0.7
0.9
0
100
200
300
400

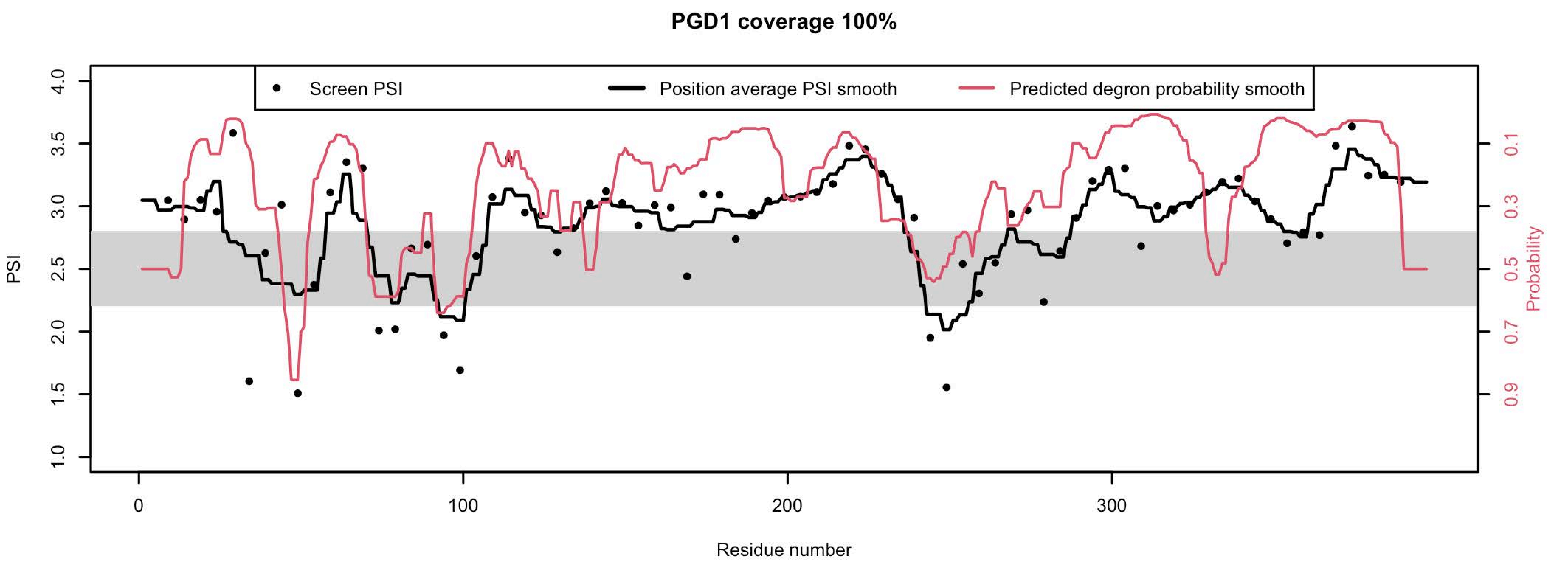

PGD1 coverage 100%
Screen PSI
Position average PSI smooth
Predicted degron probability smooth
PSI
1.0
1.5
2.0
2.5
3.0
3.5
4.0
Probability
0.1
0.3
0.5
0.7
0.9
0
100
200
300
Residue number

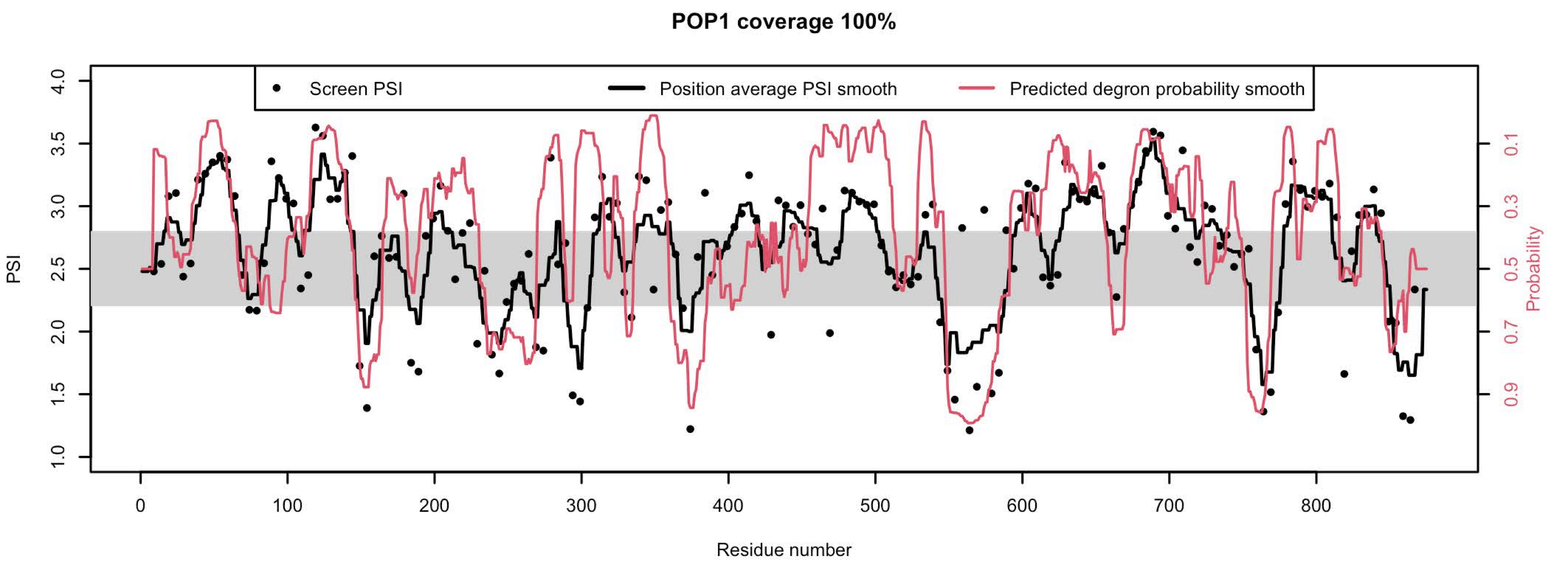
POP1 coverage 100%
Screen PSI
Position average PSI smooth
Predicted degron probability smooth
PSI
Probability
Residue number
4.0
3.5
3.0
2.5
2.0
1.5
1.0
0.1
0.3
0.5
0.7
0.9
0
100
200
300
400
500
600
700
800

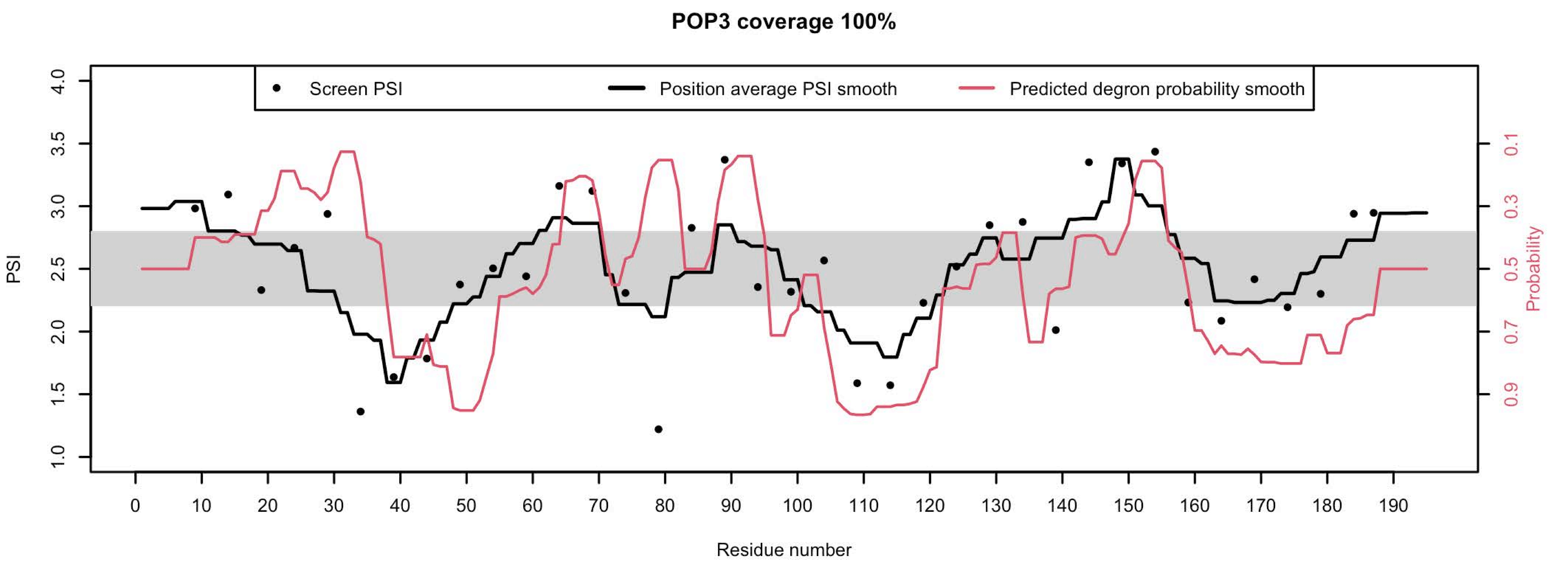
POP3 coverage 100%
Screen PSI
Position average PSI smooth
Predicted degron probability smooth
PSI
Probability
Residue number
1.0
1.5
2.0
2.5
3.0
3.5
4.0
0.1
0.3
0.5
0.7
0.9
0
10
20
30
40
50
60
70
80
90
100
110
120
130
140
150
160
170
180
190

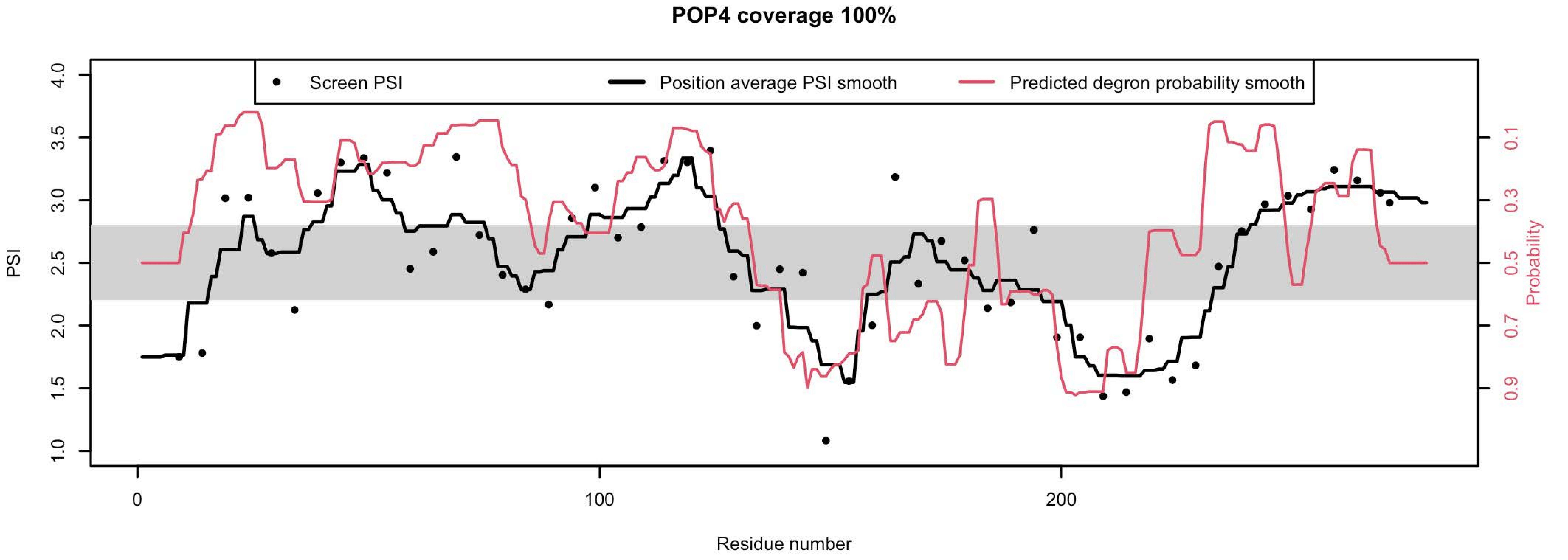

POP4 coverage 100%
Screen PSI
Position average PSI smooth
Predicted degron probability smooth
PSI
1.0
1.5
2.0
2.5
3.0
3.5
4.0
Probability
0.1
0.3
0.5
0.7
0.9
0
100
200
Residue number

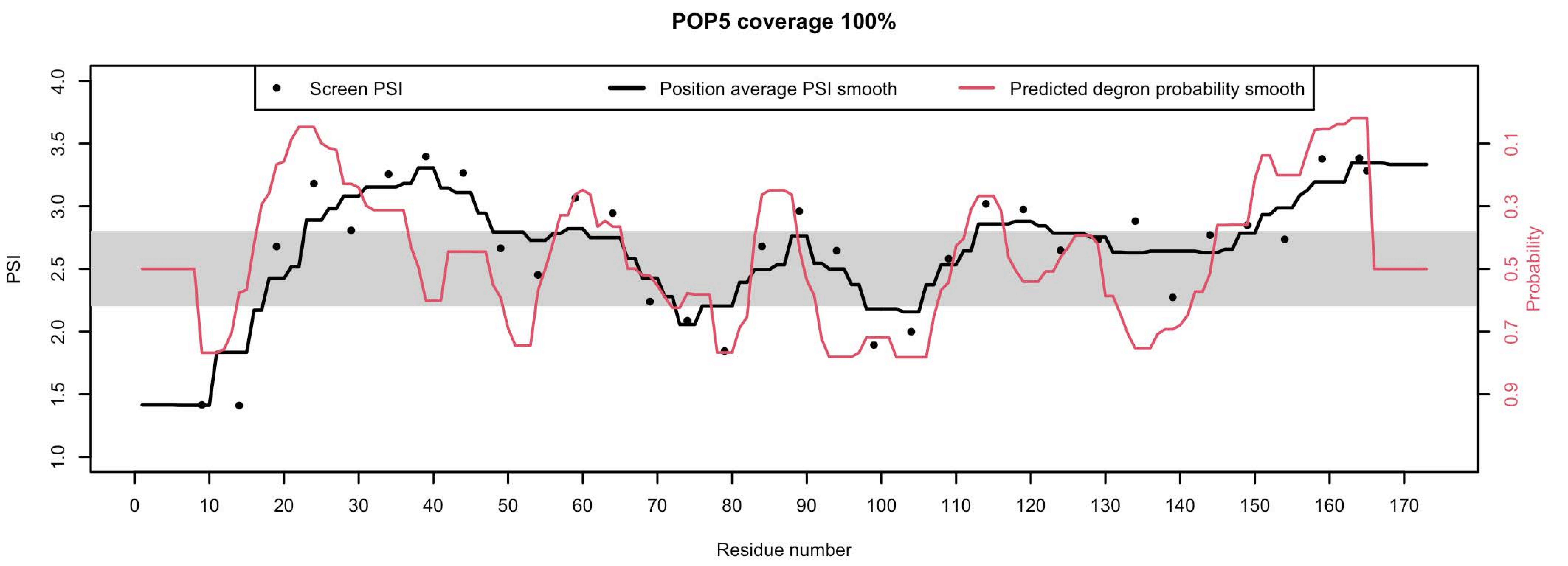
POP5 coverage 100%
Screen PSI
Position average PSI smooth
Predicted degron probability smooth
PSI
Probability
Residue number
1.0
1.5
2.0
2.5
3.0
3.5
4.0
0.1
0.3
0.5
0.7
0.9
0
10
20
30
40
50
60
70
80
90
100
110
120
130
140
150
160
170

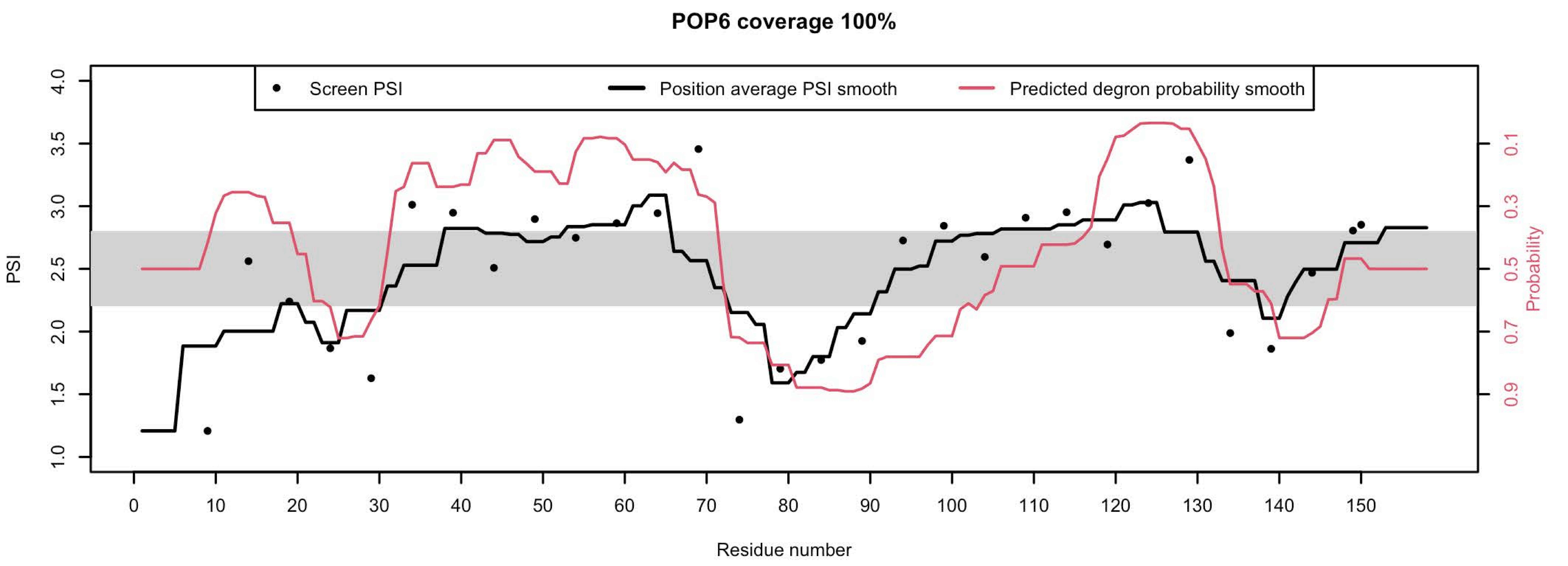
POP6 coverage 100%
Screen PSI
Position average PSI smooth
Predicted degron probability smooth
PSI
1.0
1.5
2.0
2.5
3.0
3.5
4.0
Probability
0.1
0.3
0.5
0.7
0.9
0
10
20
30
40
50
60
70
80
90
100
110
120
130
140
150
Residue number

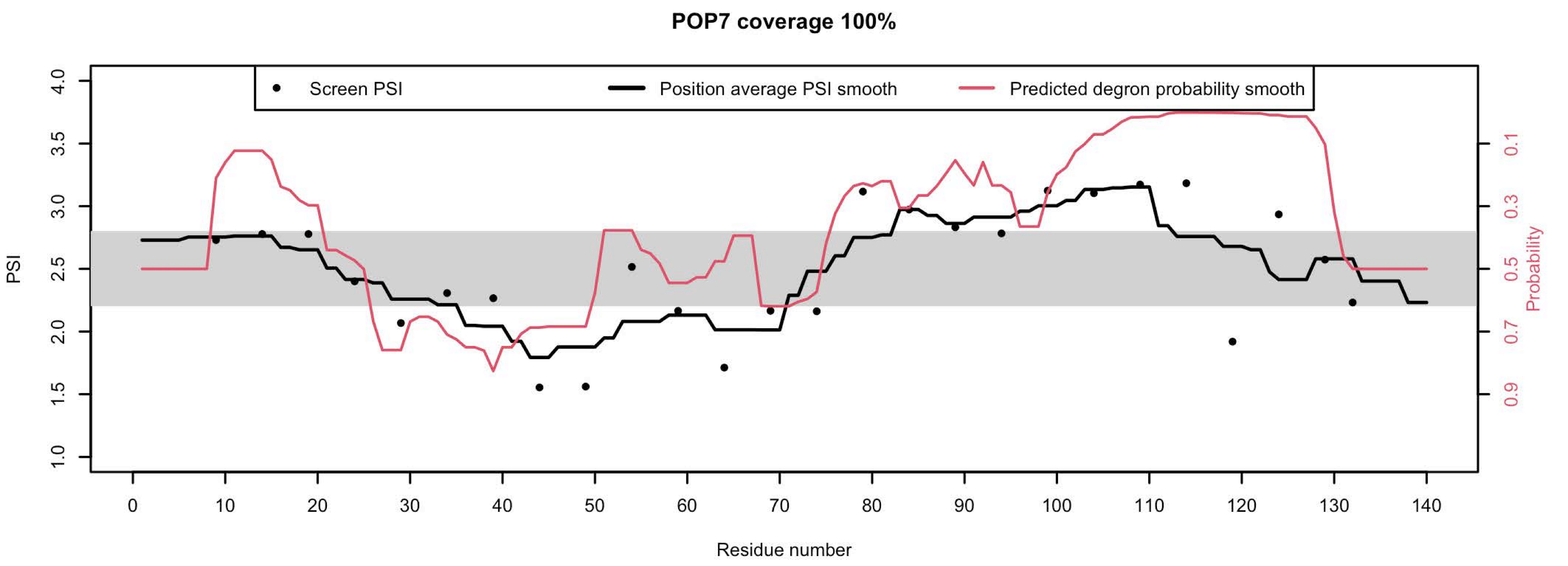
POP7 coverage 100%
Screen PSI
Position average PSI smooth
Predicted degron probability smooth
PSI
1.0
1.5
2.0
2.5
3.0
3.5
4.0
Probability
0.1
0.3
0.5
0.7
0.9
0
10
20
30
40
50
60
70
80
90
100
110
120
130
140
Residue number

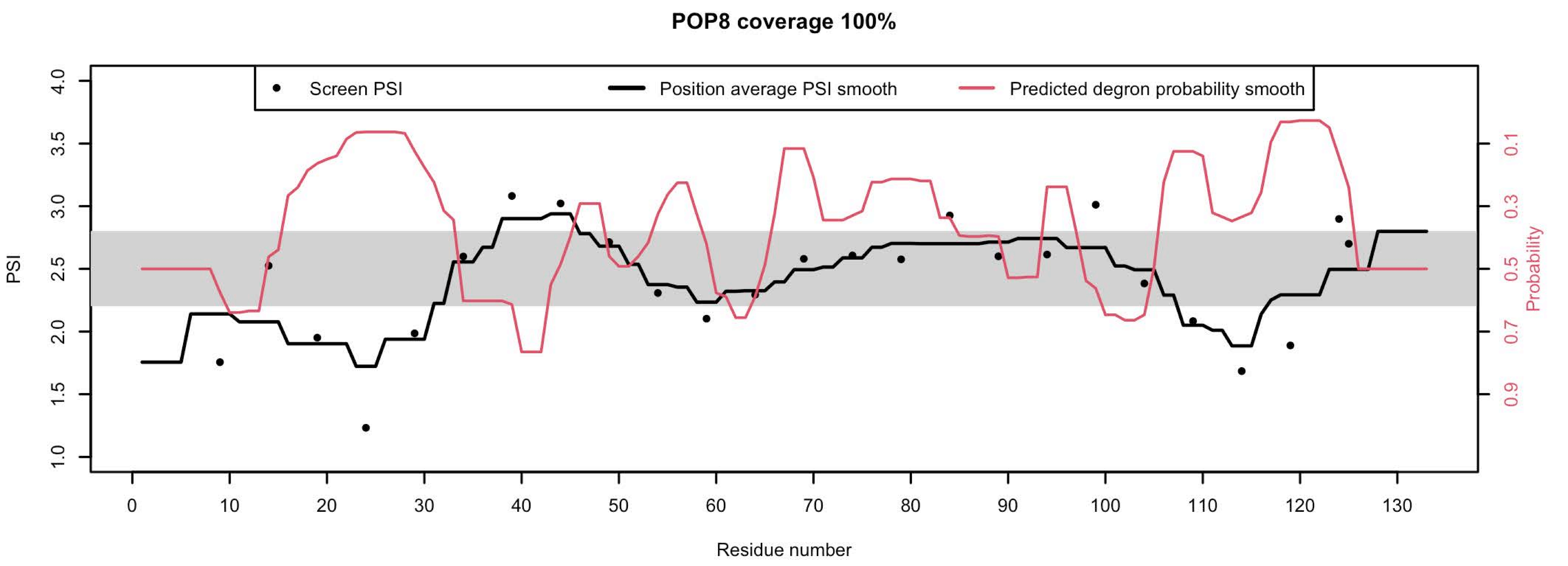
POP8 coverage 100%
Screen PSI
Position average PSI smooth
Predicted degron probability smooth
PSI
1.0
1.5
2.0
2.5
3.0
3.5
4.0
Probability
0.1
0.3
0.5
0.7
0.9
0
10
20
30
40
50
60
70
80
90
100
110
120
130
Residue number

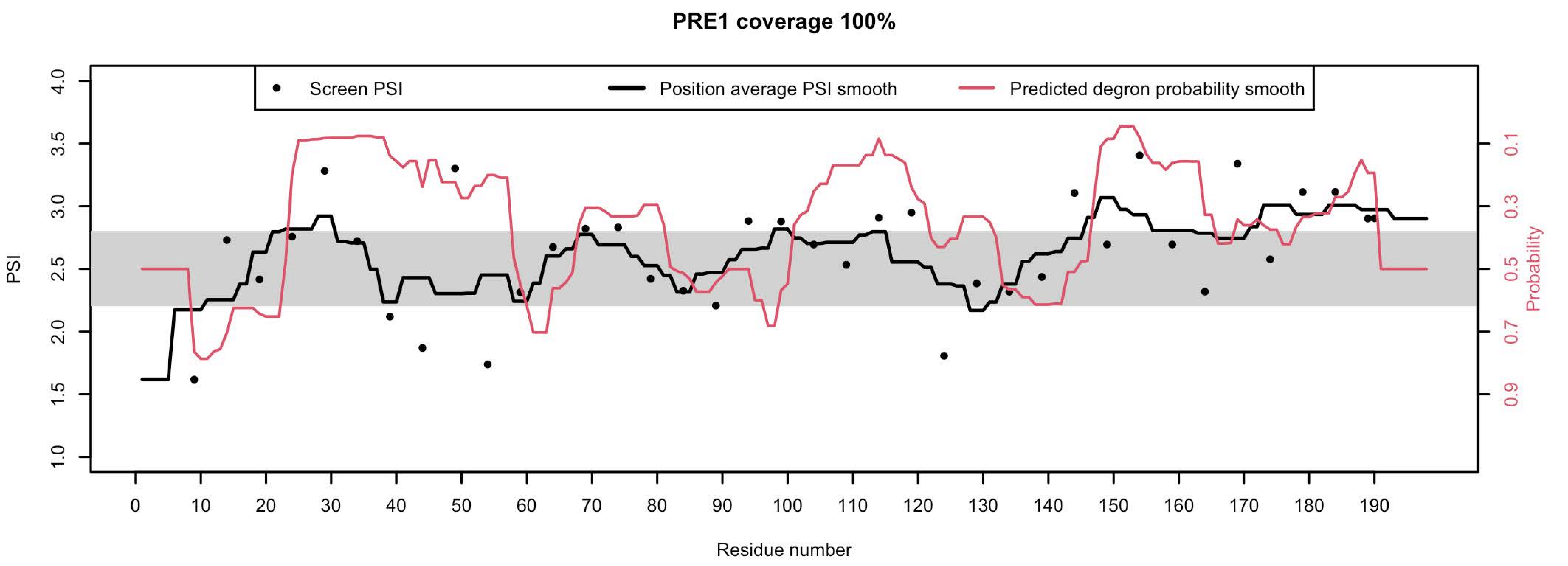
PRE1 coverage 100%
Screen PSI
Position average PSI smooth
Predicted degron probability smooth
PSI
Probability
Residue number
1.0
1.5
2.0
2.5
3.0
3.5
4.0
0.1
0.3
0.5
0.7
0.9
0
10
20
30
40
50
60
70
80
90
100
110
120
130
140
150
160
170
180
190

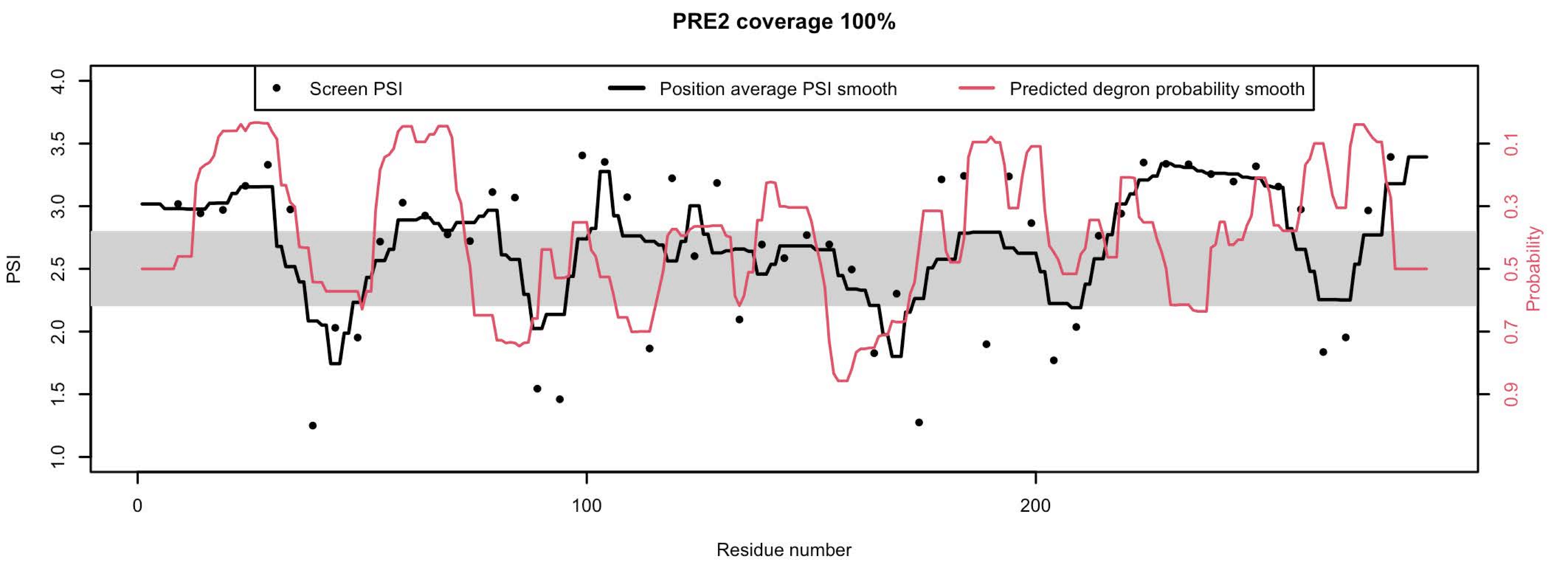
PRE2 coverage 100%
Screen PSI
Position average PSI smooth
Predicted degron probability smooth
PSI
1.0
1.5
2.0
2.5
3.0
3.5
4.0
Probability
0.1
0.3
0.5
0.7
0.9
0
100
200
Residue number

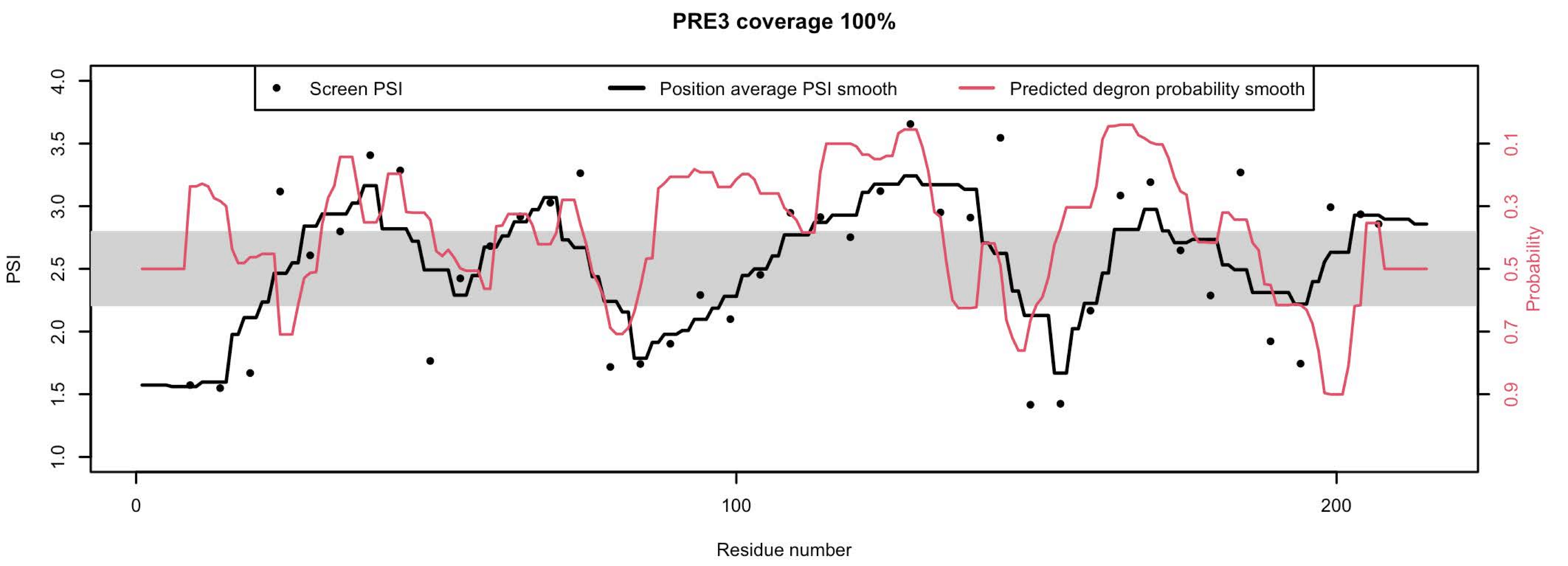

PRE3 coverage 100%
Screen PSI
Position average PSI smooth
Predicted degron probability smooth
PSI
1.0
1.5
2.0
2.5
3.0
3.5
4.0
Probability
0.1
0.3
0.5
0.7
0.9
0
100
200
Residue number

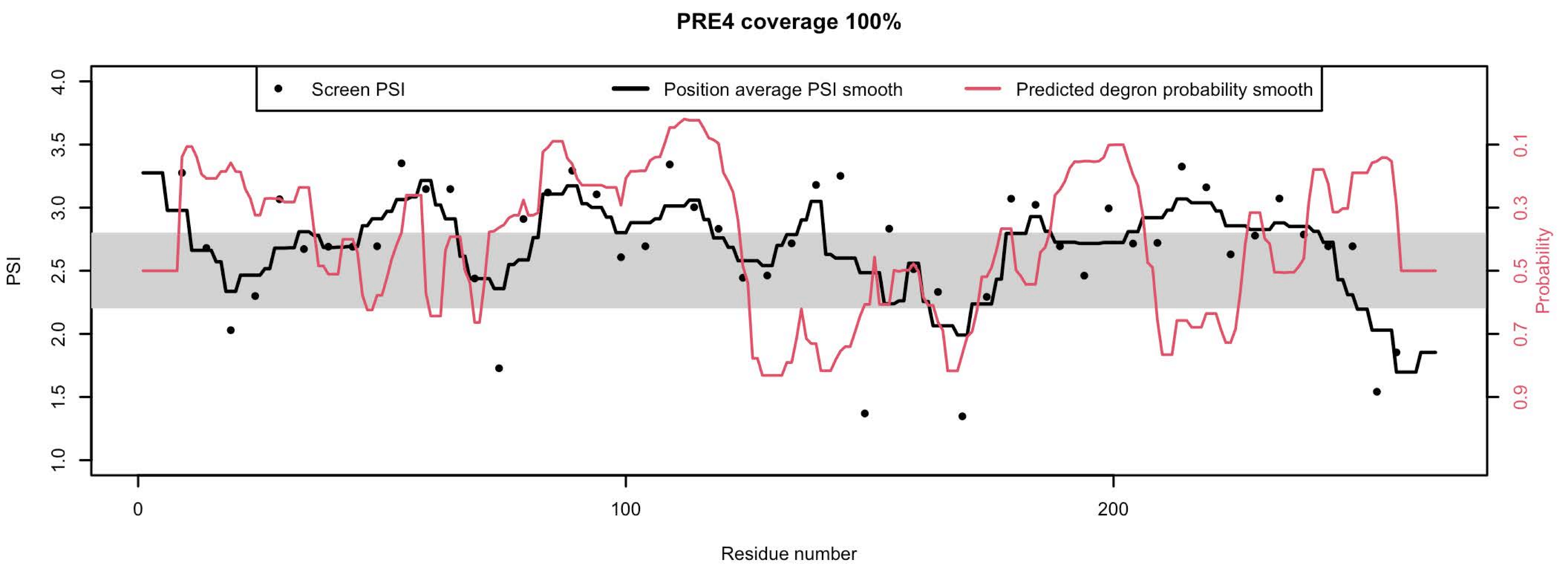

PRE4 coverage 100%
Screen PSI
Position average PSI smooth
Predicted degron probability smooth
PSI
4.0
3.5
3.0
2.5
2.0
1.5
1.0
0
100
200
Residue number
Probability
0.1
0.3
0.5
0.7
0.9

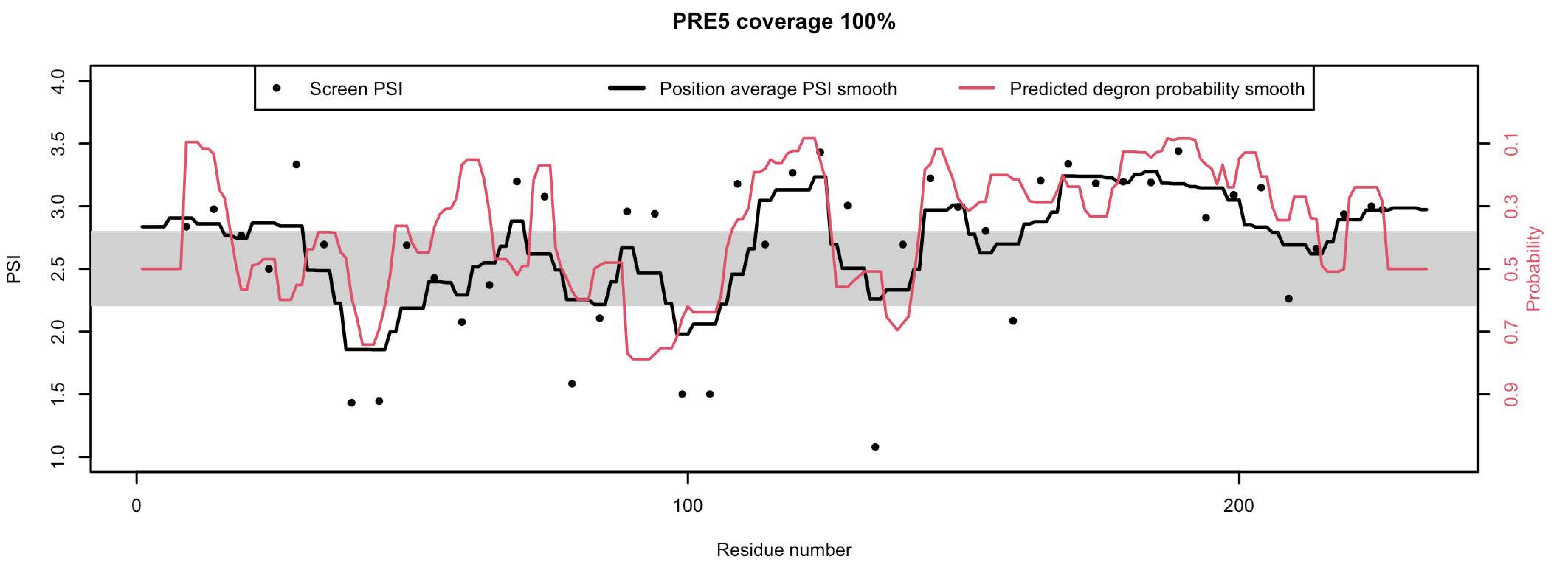
PRE5 coverage 100%
Screen PSI
Position average PSI smooth
Predicted degron probability smooth
PSI
1.0
1.5
2.0
2.5
3.0
3.5
4.0
Probability
0.1
0.3
0.5
0.7
0.9
0
100
200
Residue number

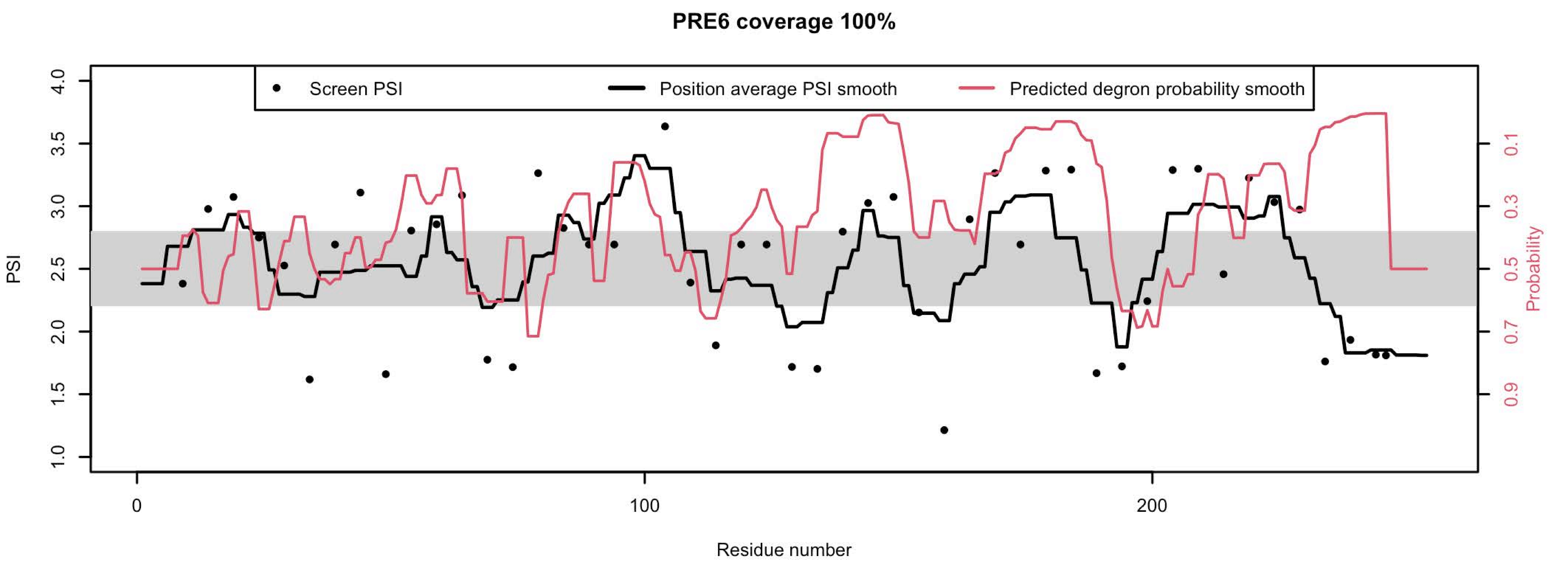

PRE6 coverage 100%
Screen PSI
Position average PSI smooth
Predicted degron probability smooth
PSI
1.0
1.5
2.0
2.5
3.0
3.5
4.0
Probability
0.1
0.3
0.5
0.7
0.9
0
100
200
Residue number

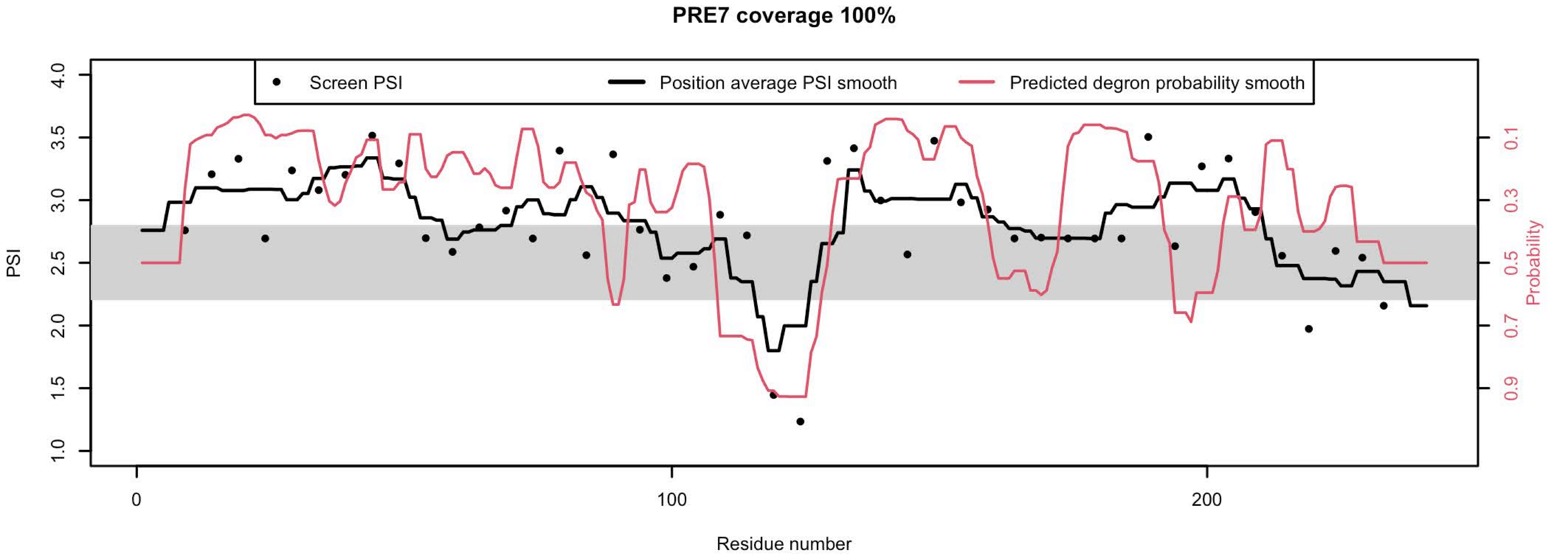
PRE7 coverage 100%
Screen PSI
Position average PSI smooth
Predicted degron probability smooth
PSI
4.0
3.5
3.0
2.5
2.0
1.5
1.0
Probability
0.1
0.3
0.5
0.7
0.9
0
100
200
Residue number

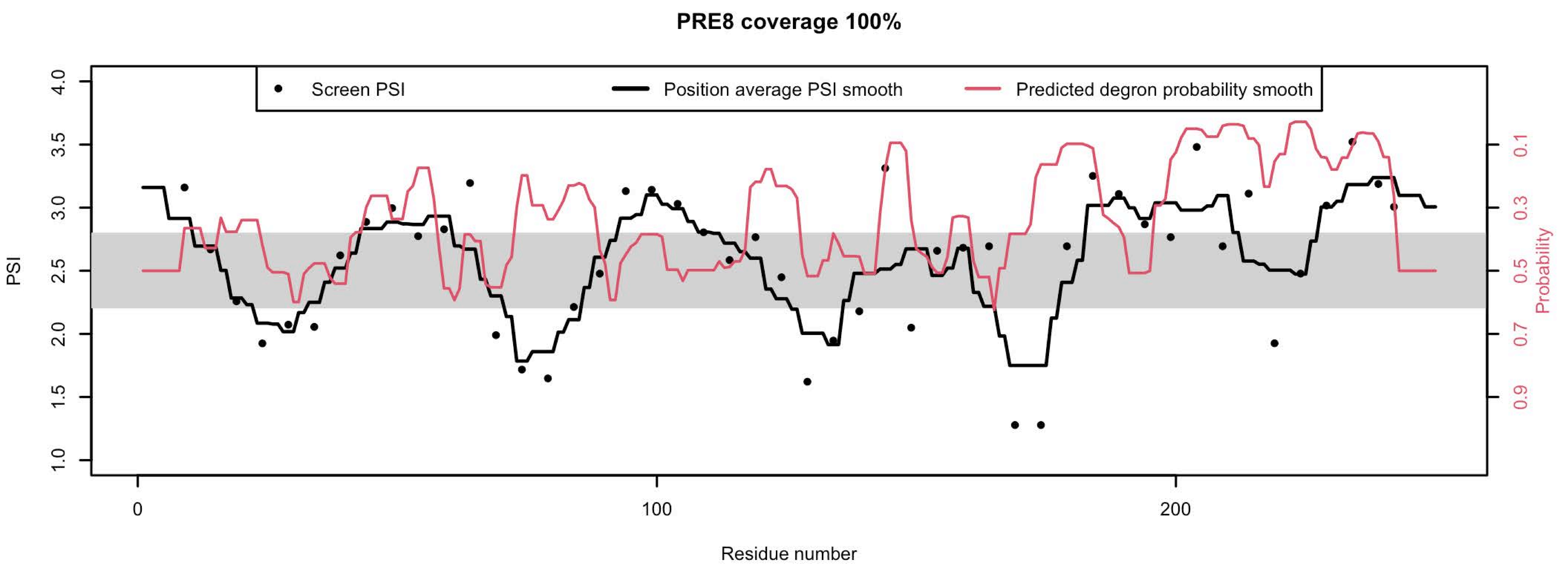

PRE8 coverage 100%
Screen PSI
Position average PSI smooth
Predicted degron probability smooth
PSI
1.0
1.5
2.0
2.5
3.0
3.5
4.0
Probability
0.1
0.3
0.5
0.7
0.9
0
100
200
Residue number

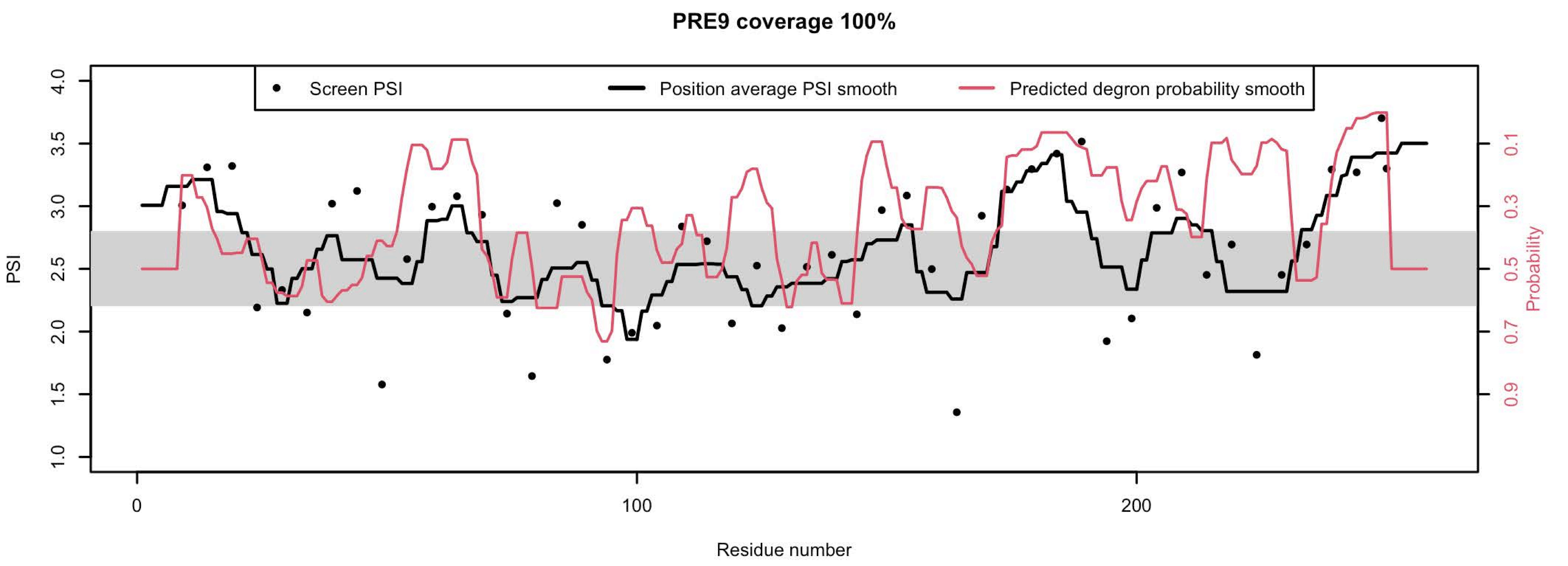
PRE9 coverage 100%
Screen PSI
Position average PSI smooth
Predicted degron probability smooth
PSI
4.0
3.5
3.0
2.5
2.0
1.5
1.0
0
100
200
Residue number
Probability
0.1
0.3
0.5
0.7
0.9

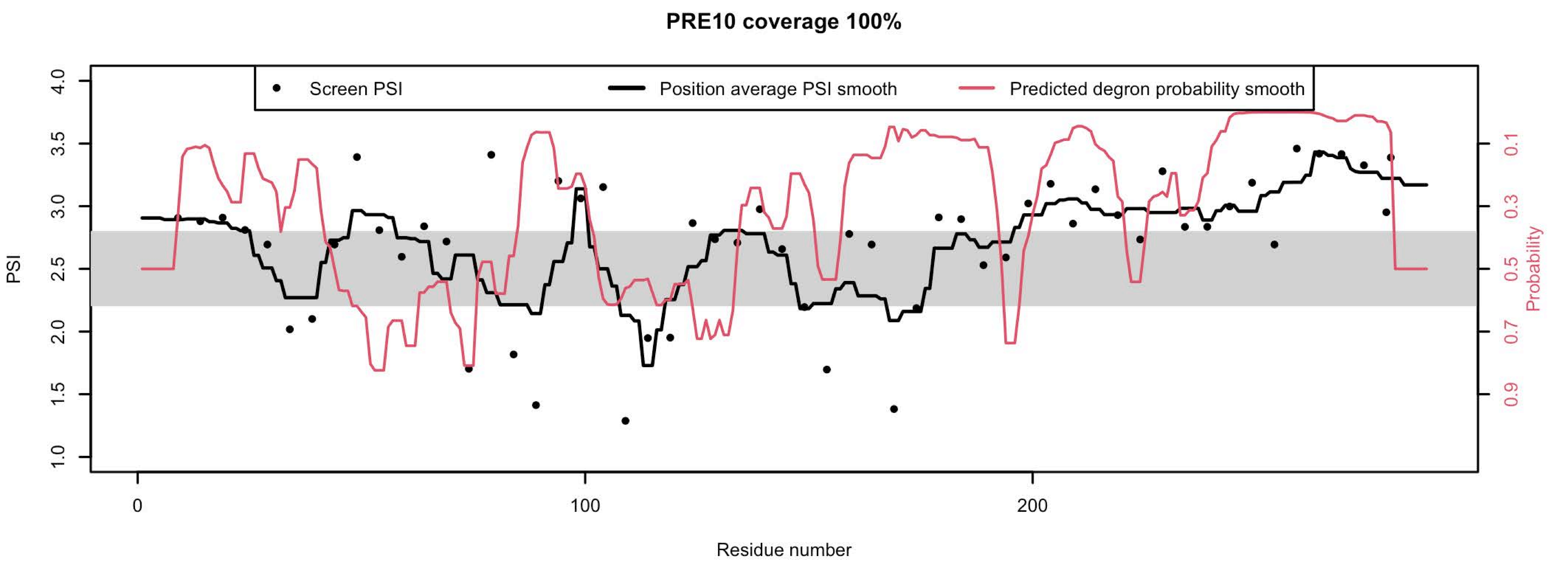

PRE10 coverage 100%
Screen PSI
Position average PSI smooth
Predicted degron probability smooth
PSI
1.0
1.5
2.0
2.5
3.0
3.5
4.0
Probability
0.1
0.3
0.5
0.7
0.9
0
100
200
Residue number

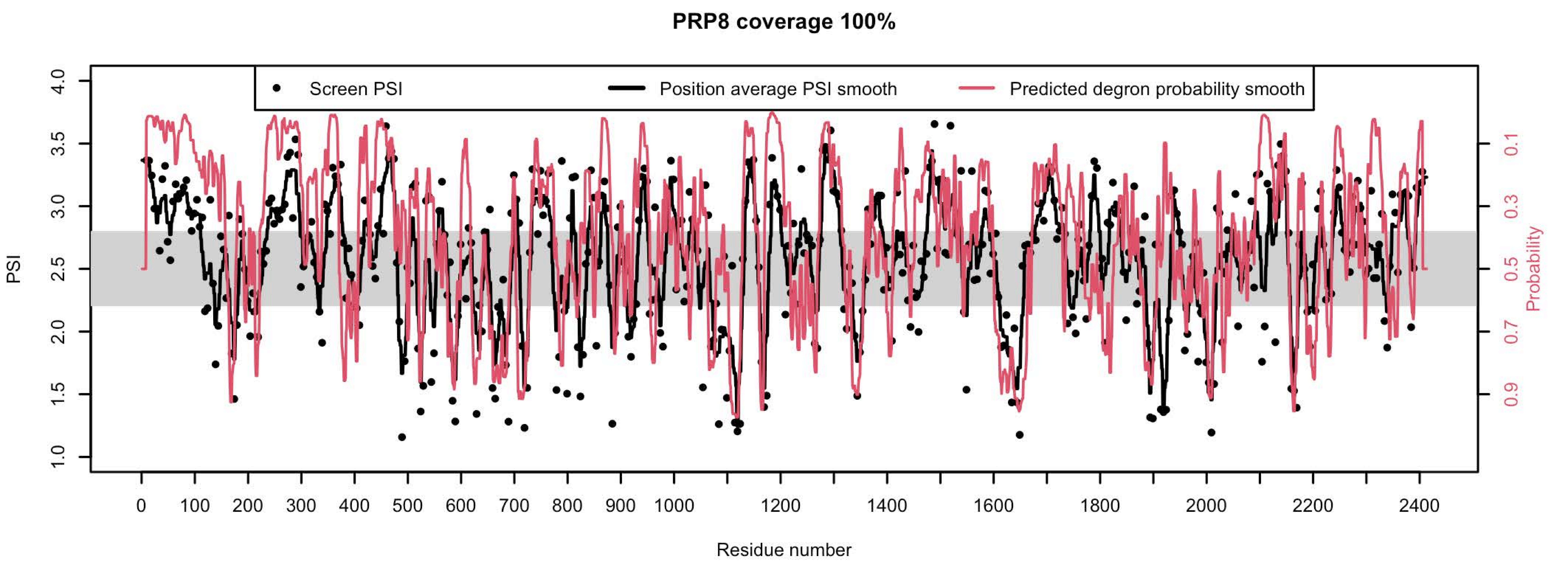
PRP8 coverage 100%
Screen PSI
Position average PSI smooth
Predicted degron probability smooth
PSI
Probability
Residue number
1.0
1.5
2.0
2.5
3.0
3.5
4.0
0.1
0.3
0.5
0.7
0.9
0
100
200
300
400
500
600
700
800
900
1000
1200
1400
1600
1800
2000
2200
2400

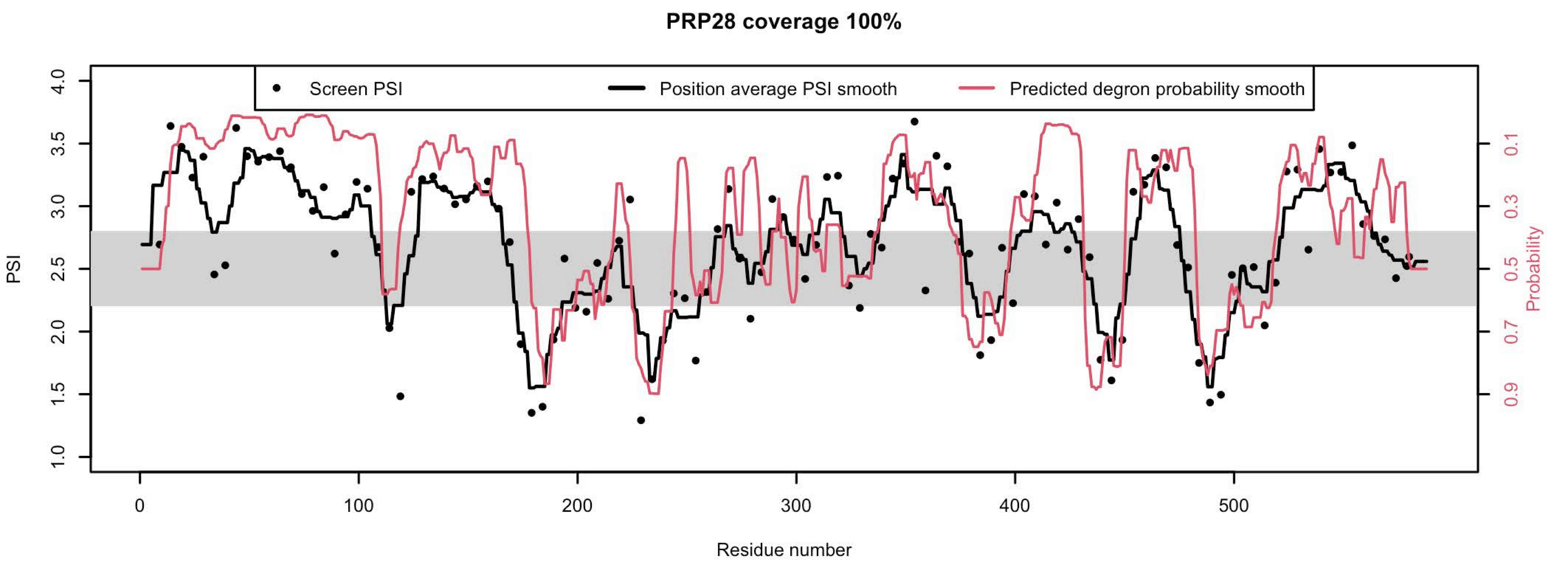

PRP28 coverage 100%
Screen PSI
Position average PSI smooth
Predicted degron probability smooth
PSI
4.0
3.5
3.0
2.5
2.0
1.5
1.0
Probability
0.1
0.3
0.5
0.7
0.9
0
100
200
300
400
500
Residue number

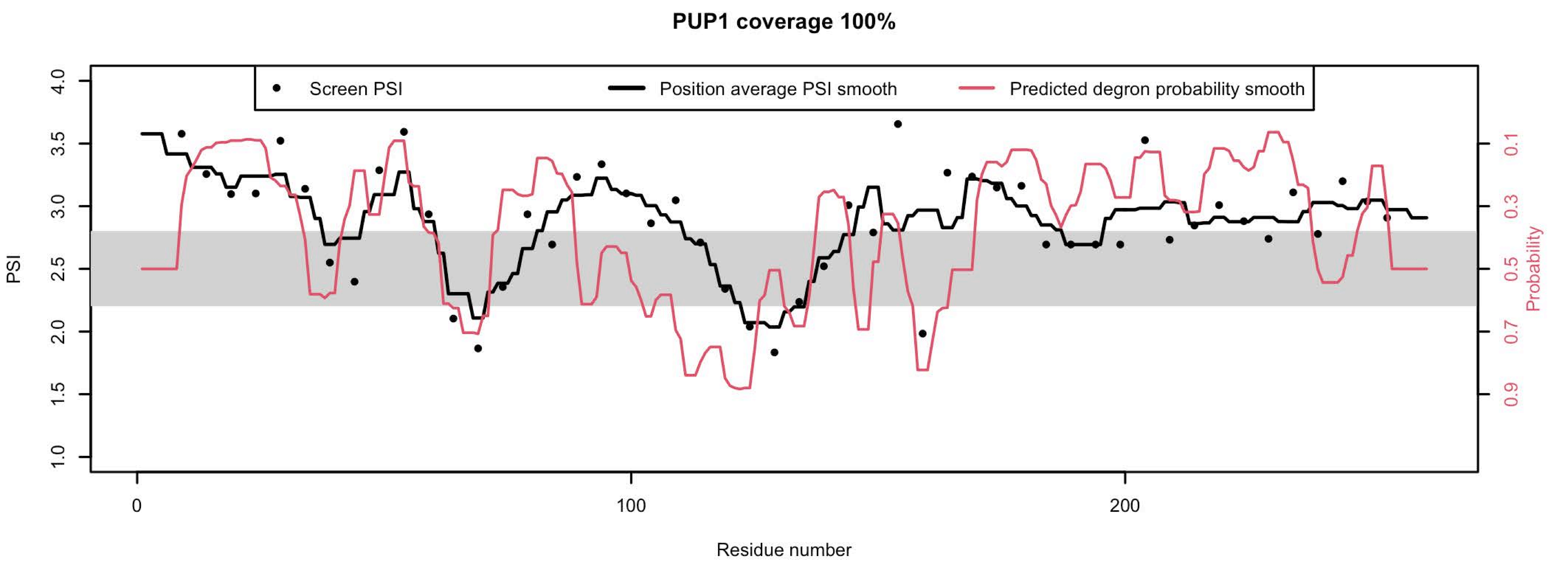
PUP1 coverage 100%
Screen PSI
Position average PSI smooth
Predicted degron probability smooth
PSI
4.0
3.5
3.0
2.5
2.0
1.5
1.0
Probability
0.1
0.3
0.5
0.7
0.9
0
100
200
Residue number

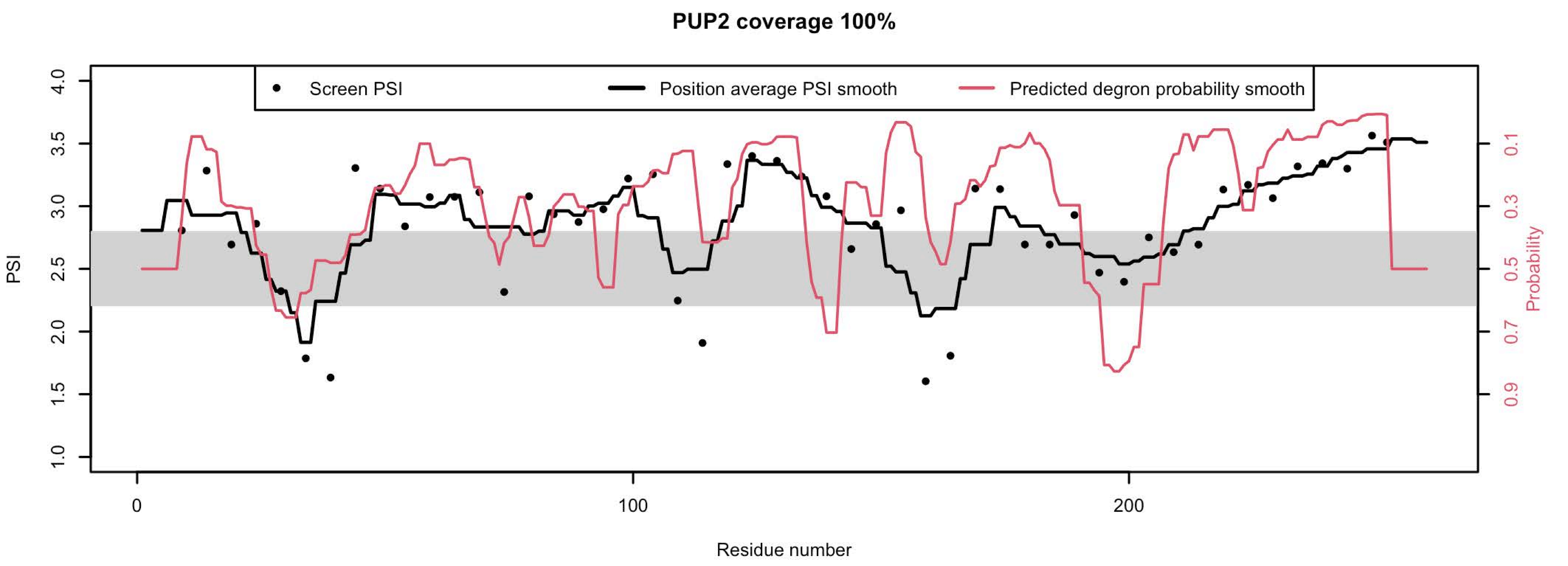
PUP2 coverage 100%
Screen PSI
Position average PSI smooth
Predicted degron probability smooth
PSI
1.0
1.5
2.0
2.5
3.0
3.5
4.0
Probability
0.9
0.7
0.5
0.3
0.1
0
100
200
Residue number

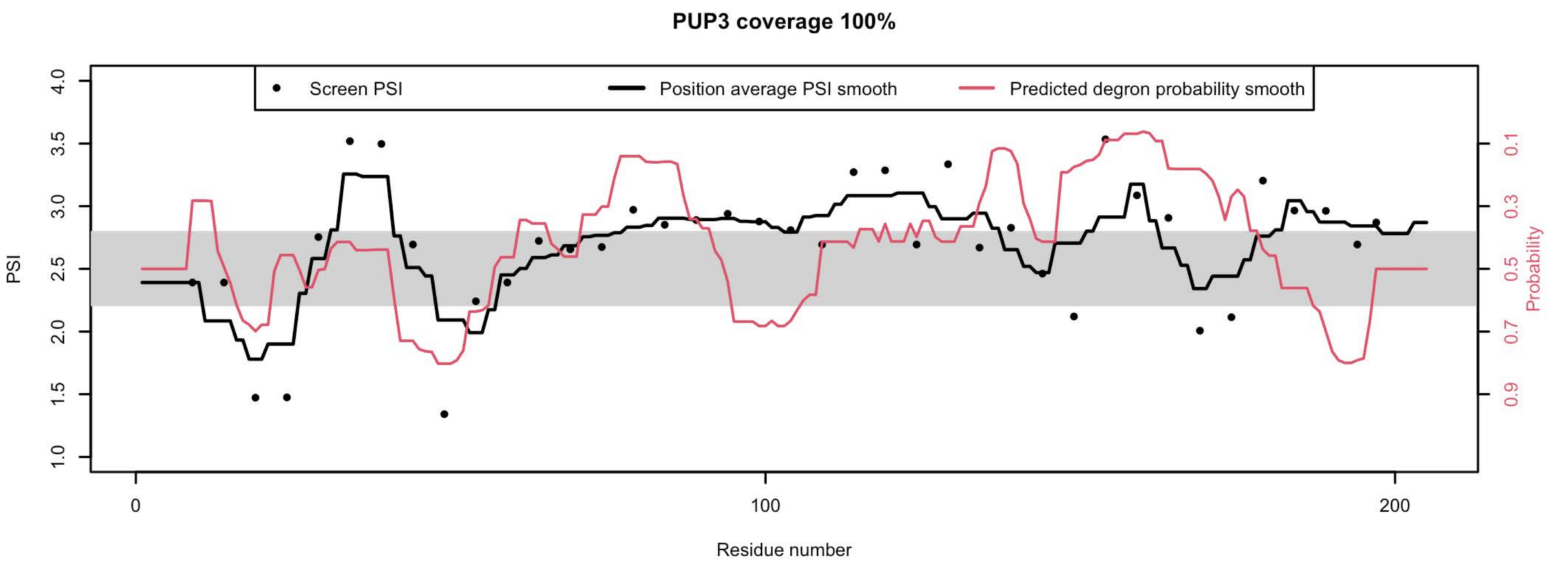

PUP3 coverage 100%
Screen PSI
Position average PSI smooth
Predicted degron probability smooth
PSI
1.0
1.5
2.0
2.5
3.0
3.5
4.0
Probability
0.1
0.3
0.5
0.7
0.9
0
100
200
Residue number

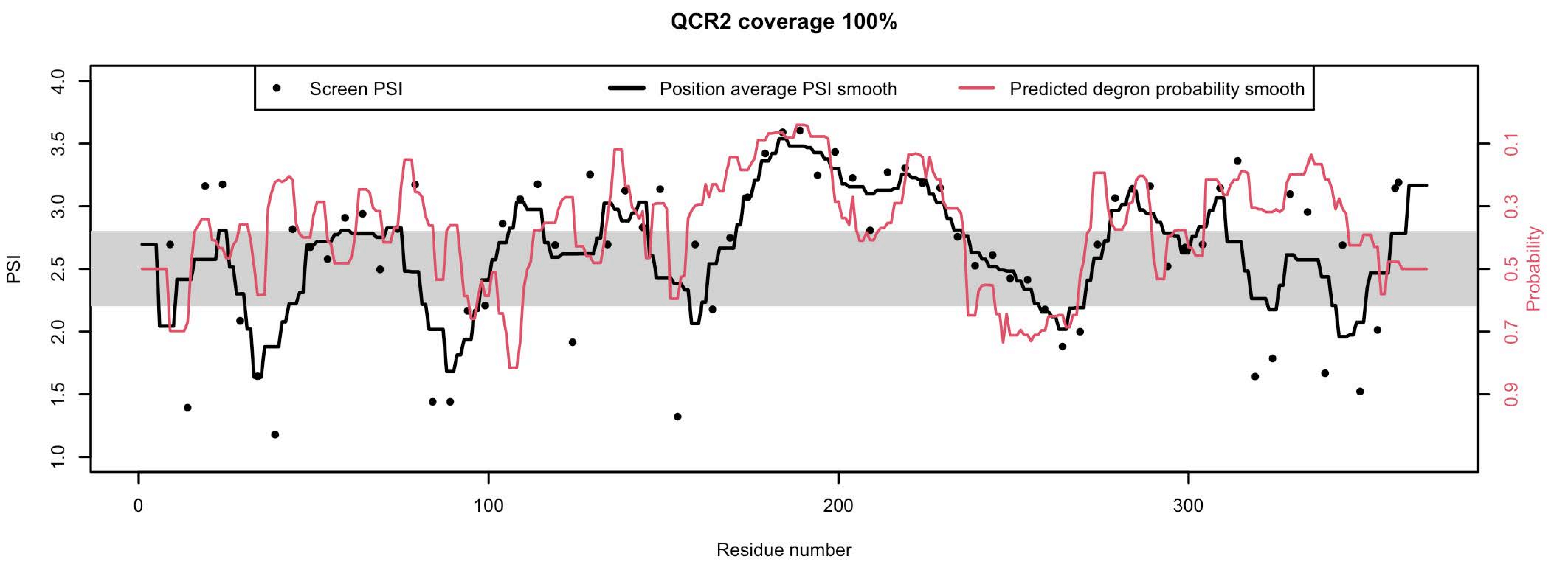

QCR2 coverage 100%
Screen PSI
Position average PSI smooth
Predicted degron probability smooth
PSI
1.0
1.5
2.0
2.5
3.0
3.5
4.0
Probability
0.1
0.3
0.5
0.7
0.9
0
100
200
300
Residue number

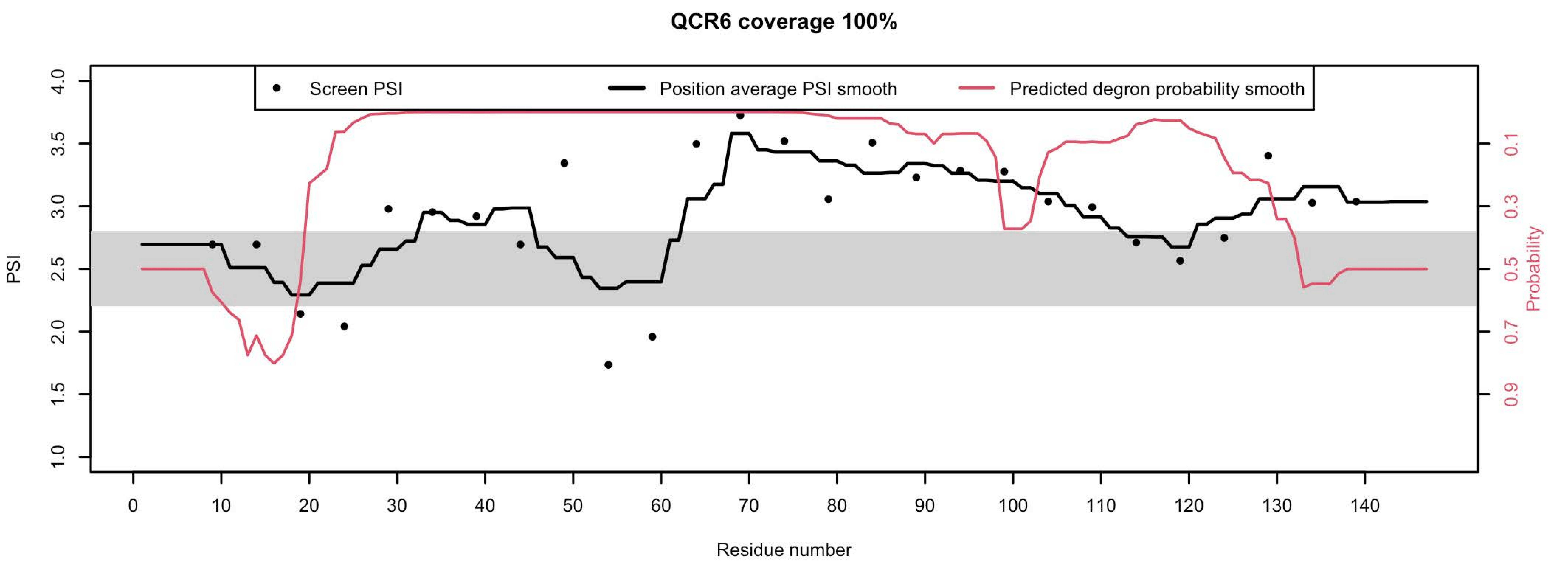

QCR6 coverage 100%
Screen PSI
Position average PSI smooth
Predicted degron probability smooth
PSI
Probability
Residue number
1.0
1.5
2.0
2.5
3.0
3.5
4.0
0.9
0.7
0.5
0.3
0.1
0
10
20
30
40
50
60
70
80
90
100
110
120
130
140

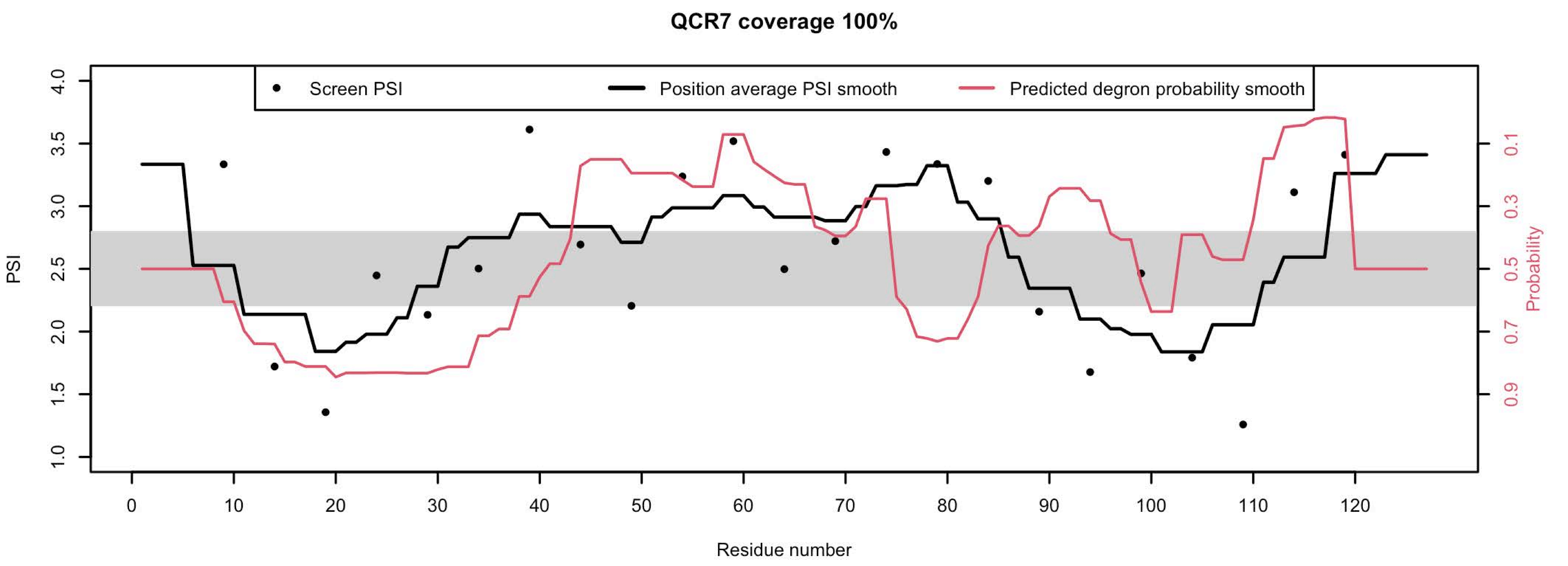
QCR7 coverage 100%
Screen PSI
Position average PSI smooth
Predicted degron probability smooth
PSI
1.0
1.5
2.0
2.5
3.0
3.5
4.0
Probability
0.1
0.3
0.5
0.7
0.9
0
10
20
30
40
50
60
70
80
90
100
110
120
Residue number

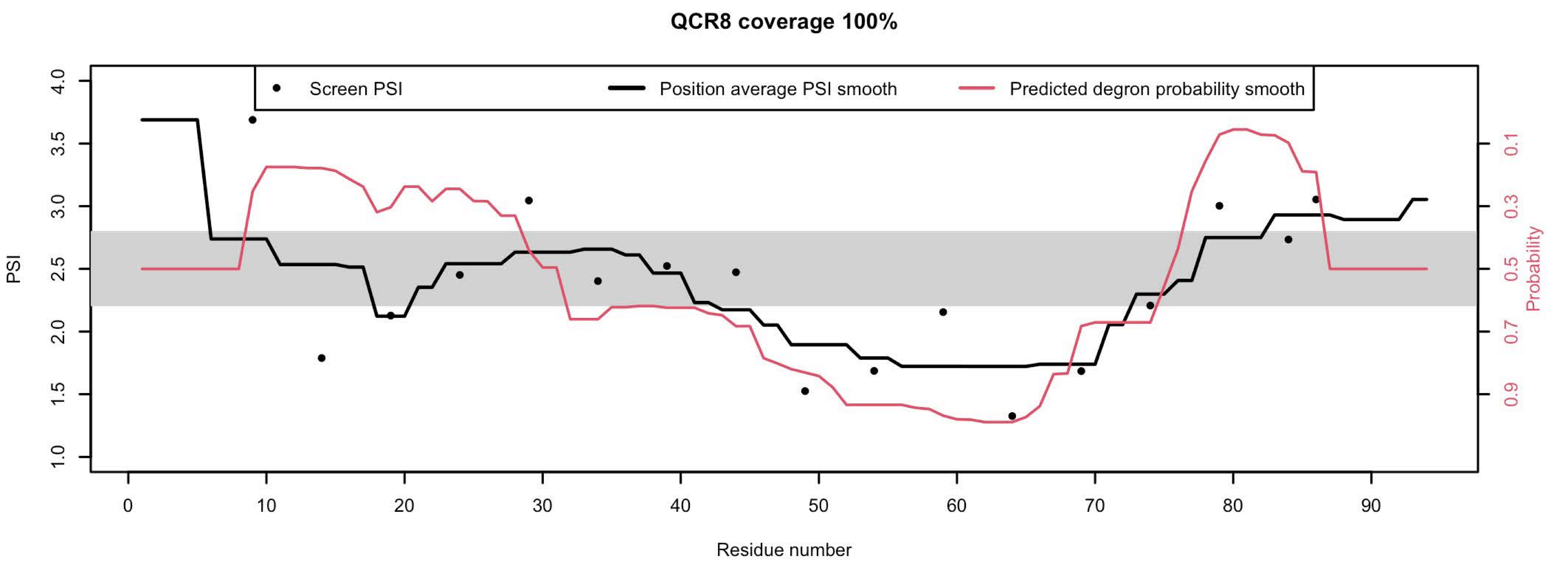

QCR8 coverage 100%
Screen PSI
Position average PSI smooth
Predicted degron probability smooth
PSI
Probability
Residue number
1.0
1.5
2.0
2.5
3.0
3.5
4.0
0.1
0.3
0.5
0.7
0.9
0
10
20
30
40
50
60
70
80
90

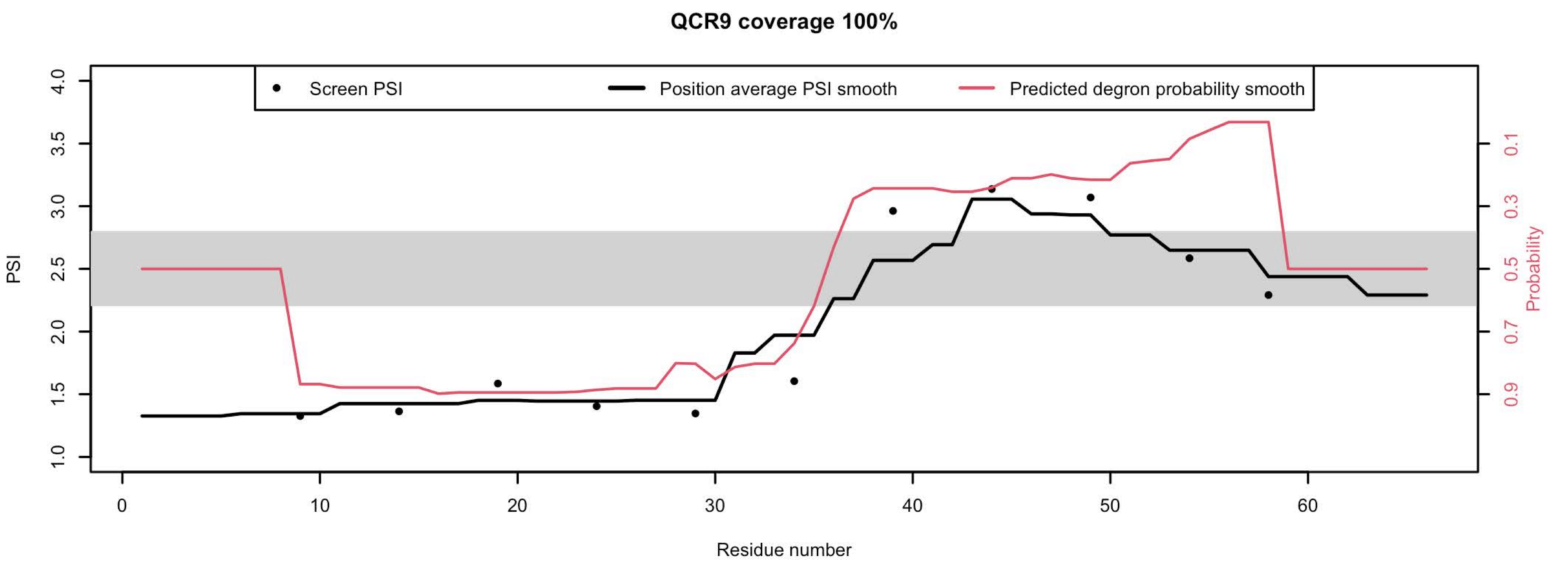

QCR9 coverage 100%
Screen PSI
Position average PSI smooth
Predicted degron probability smooth
PSI
Probability
Residue number
1.0
1.5
2.0
2.5
3.0
3.5
4.0
0.1
0.3
0.5
0.7
0.9
0
10
20
30
40
50
60

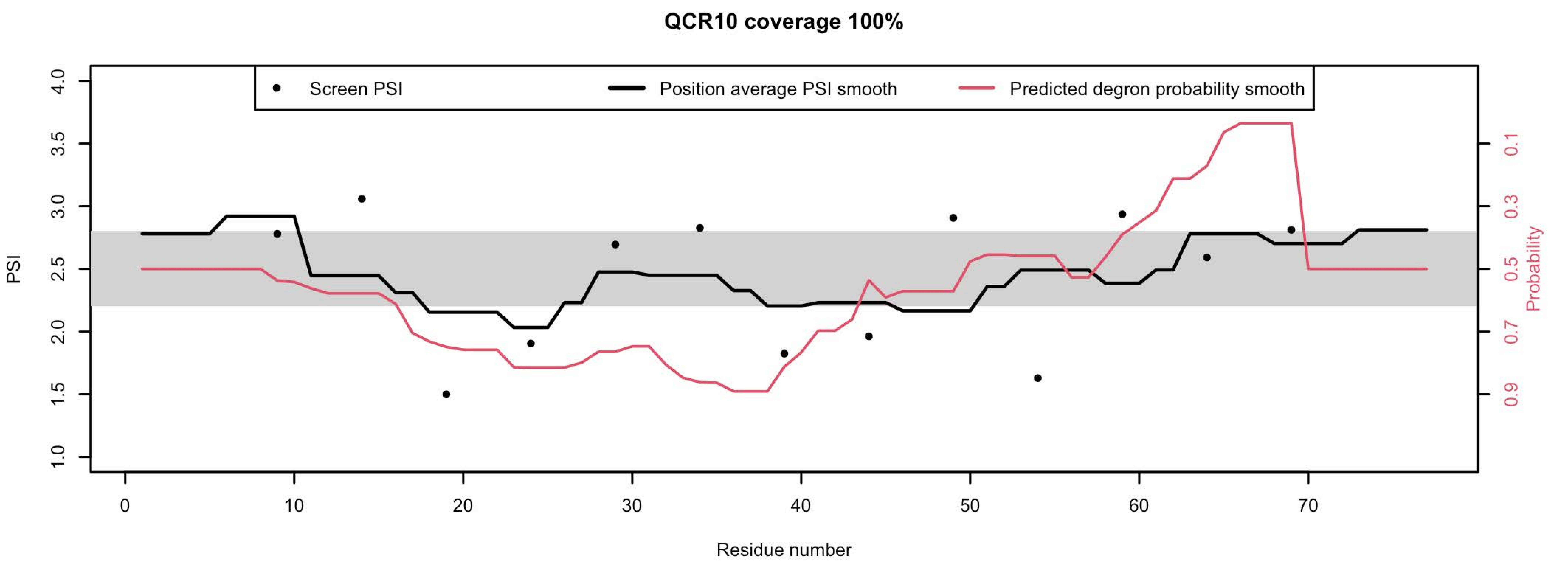

QCR10 coverage 100%
Screen PSI
Position average PSI smooth
Predicted degron probability smooth
PSI
Probability
Residue number
1.0
1.5
2.0
2.5
3.0
3.5
4.0
0.1
0.3
0.5
0.7
0.9
0
10
20
30
40
50
60
70

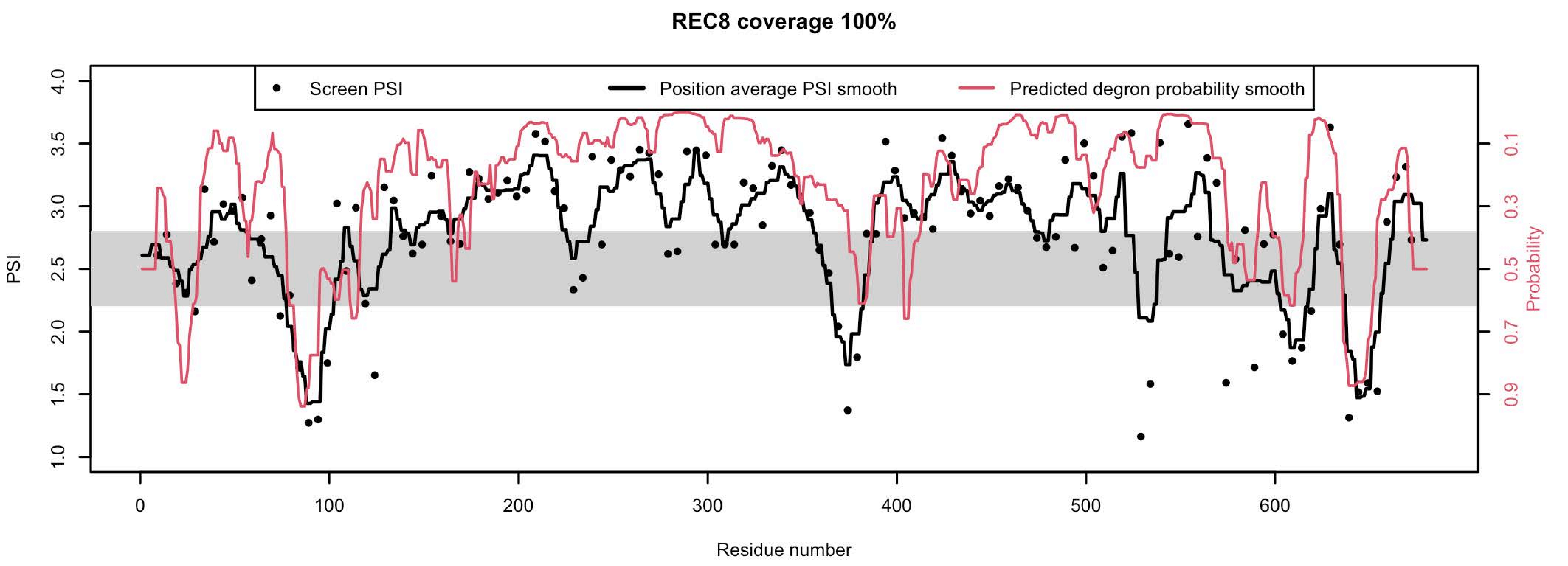

REC8 coverage 100%
Screen PSI
Position average PSI smooth
Predicted degron probability smooth
PSI
1.0
1.5
2.0
2.5
3.0
3.5
4.0
Probability
0.1
0.3
0.5
0.7
0.9
0
100
200
300
400
500
600
Residue number

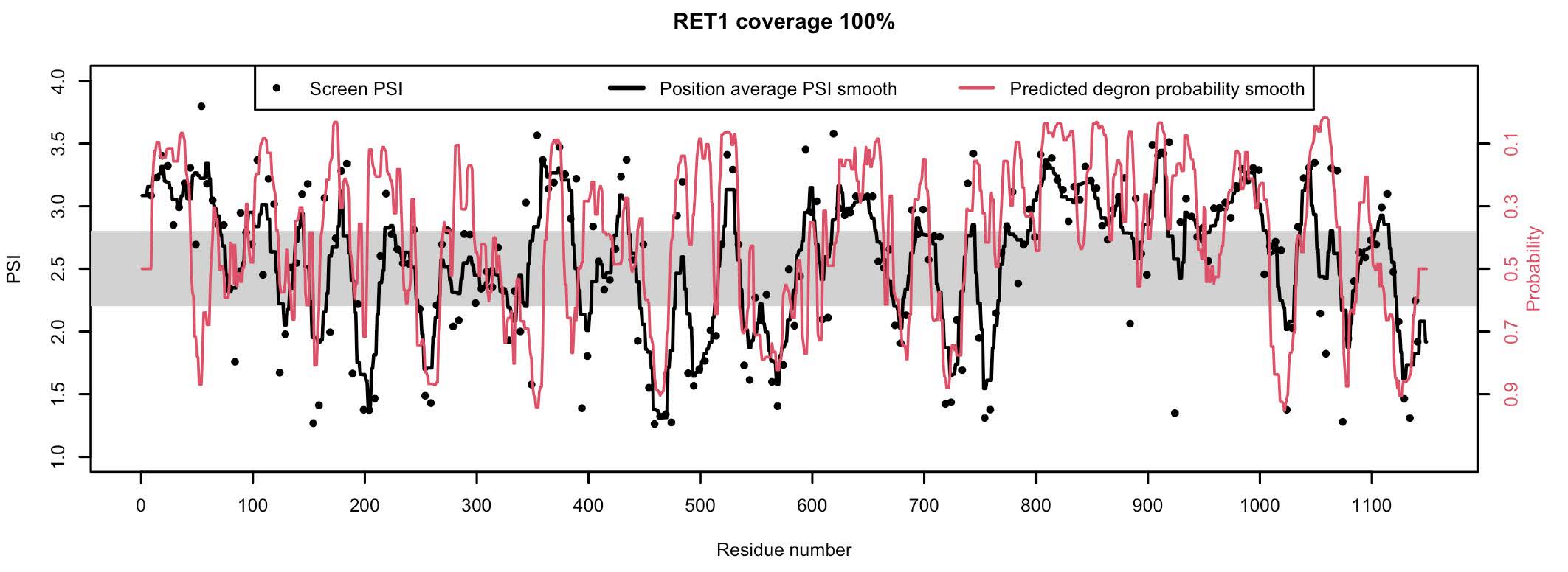
RET1 coverage 100%
Screen PSI
Position average PSI smooth
Predicted degron probability smooth
PSI
Probability
Residue number
1.0
1.5
2.0
2.5
3.0
3.5
4.0
0.1
0.3
0.5
0.7
0.9
0
100
200
300
400
500
600
700
800
900
1000
1100

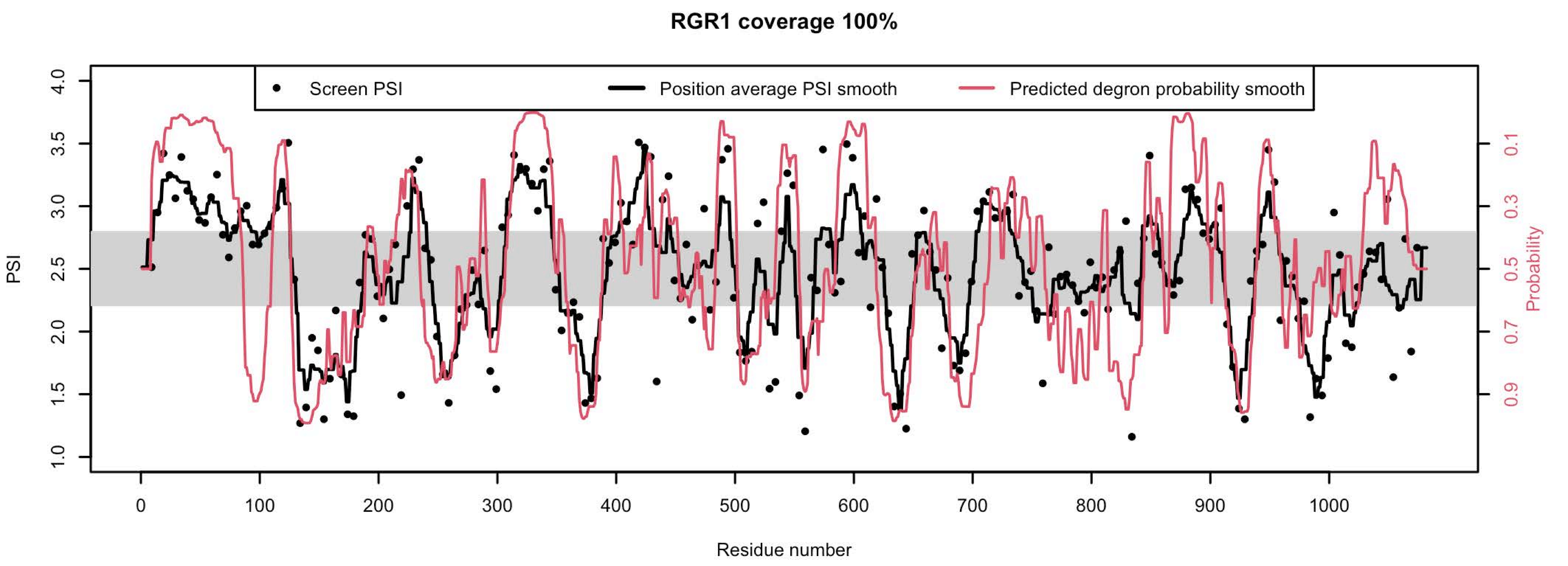

RGR1 coverage 100%
Screen PSI
Position average PSI smooth
Predicted degron probability smooth
PSI
1.0
1.5
2.0
2.5
3.0
3.5
4.0
Probability
0.1
0.3
0.5
0.7
0.9
0
100
200
300
400
500
600
700
800
900
1000
Residue number

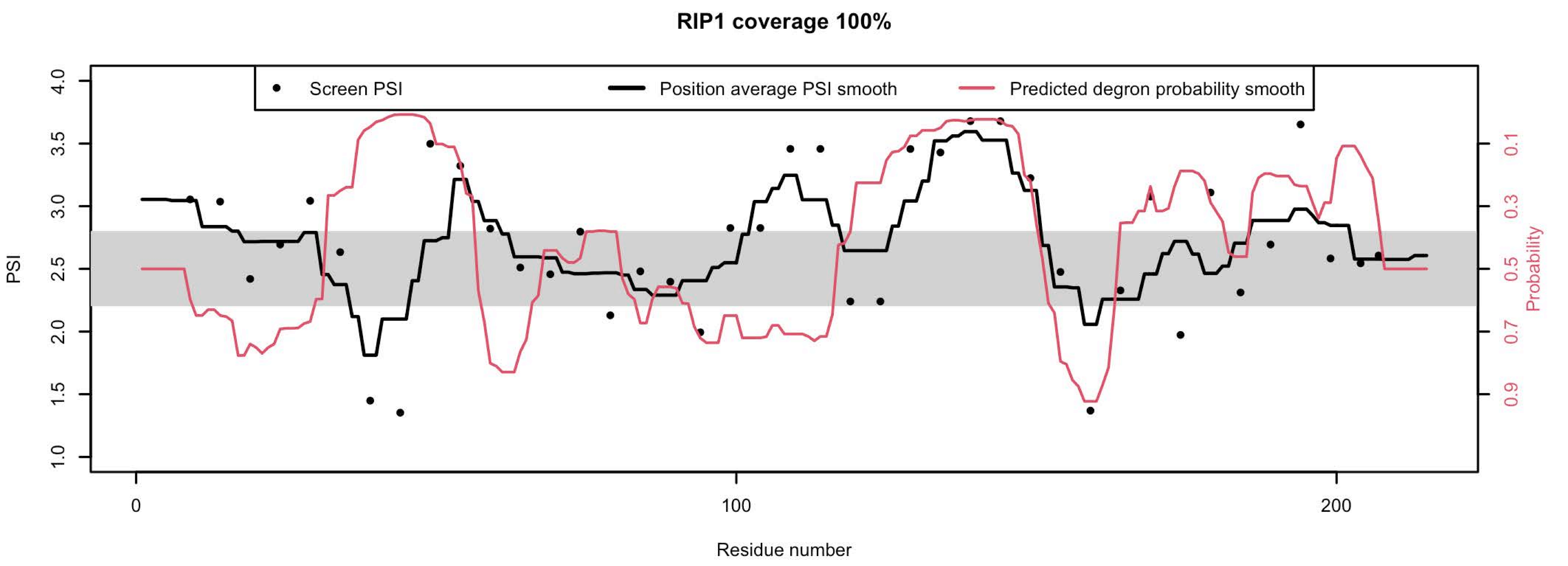

RIP1 coverage 100%
Screen PSI
Position average PSI smooth
Predicted degron probability smooth
PSI
Probability
Residue number
1.0
1.5
2.0
2.5
3.0
3.5
4.0
0.1
0.3
0.5
0.7
0.9
0
100
200

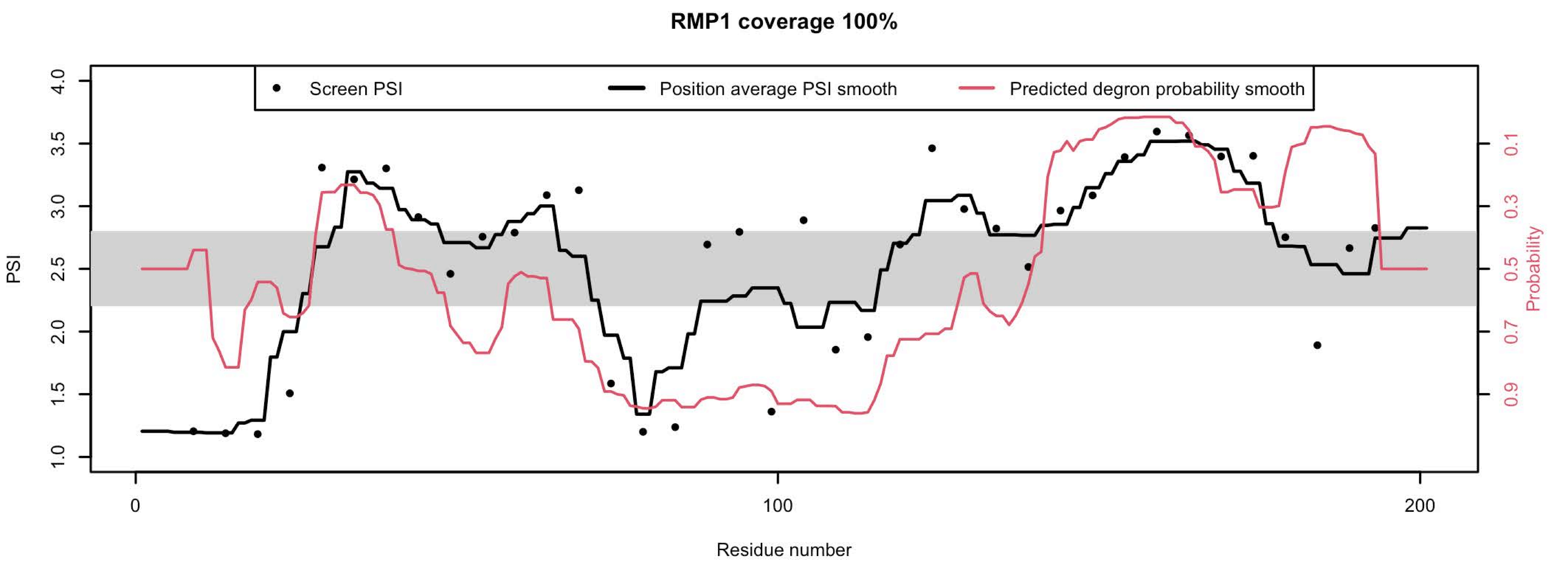

RMP1 coverage 100%
Screen PSI
Position average PSI smooth
Predicted degron probability smooth
PSI
1.0
1.5
2.0
2.5
3.0
3.5
4.0
Probability
0.1
0.3
0.5
0.7
0.9
0
100
200
Residue number

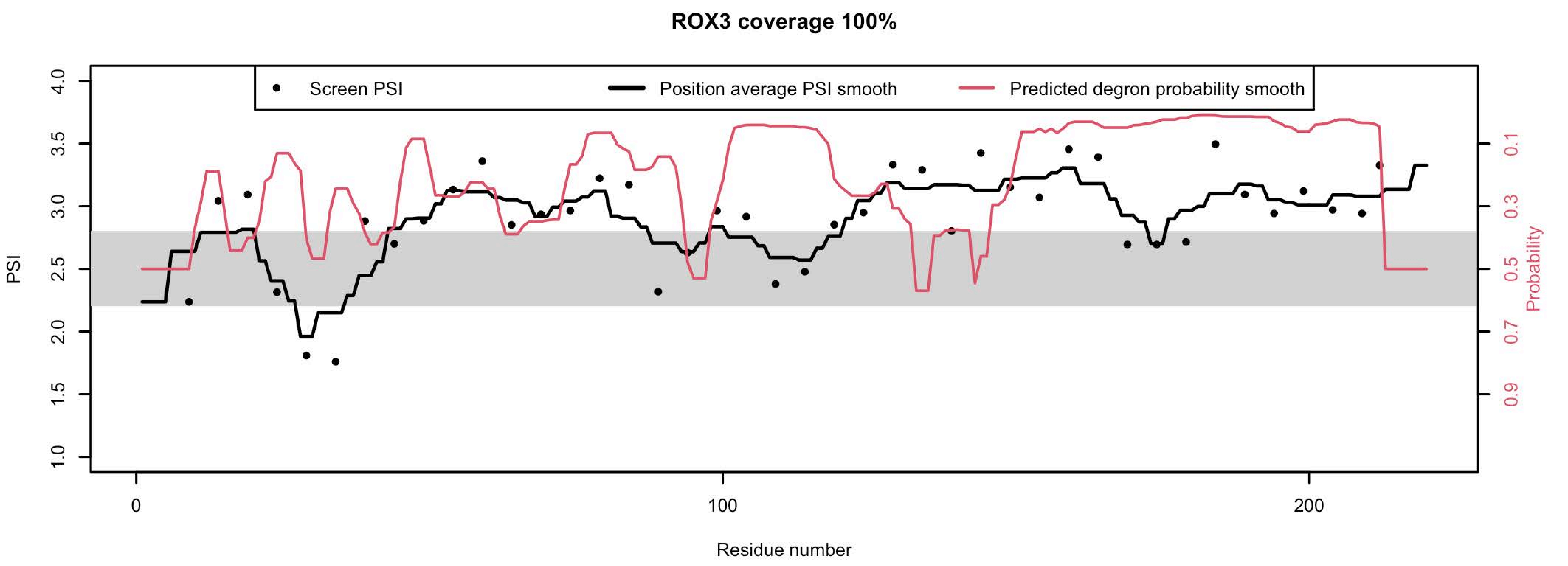
ROX3 coverage 100%
Screen PSI
Position average PSI smooth
Predicted degron probability smooth
PSI
1.0
1.5
2.0
2.5
3.0
3.5
4.0
Probability
0.1
0.3
0.5
0.7
0.9
0
100
200
Residue number

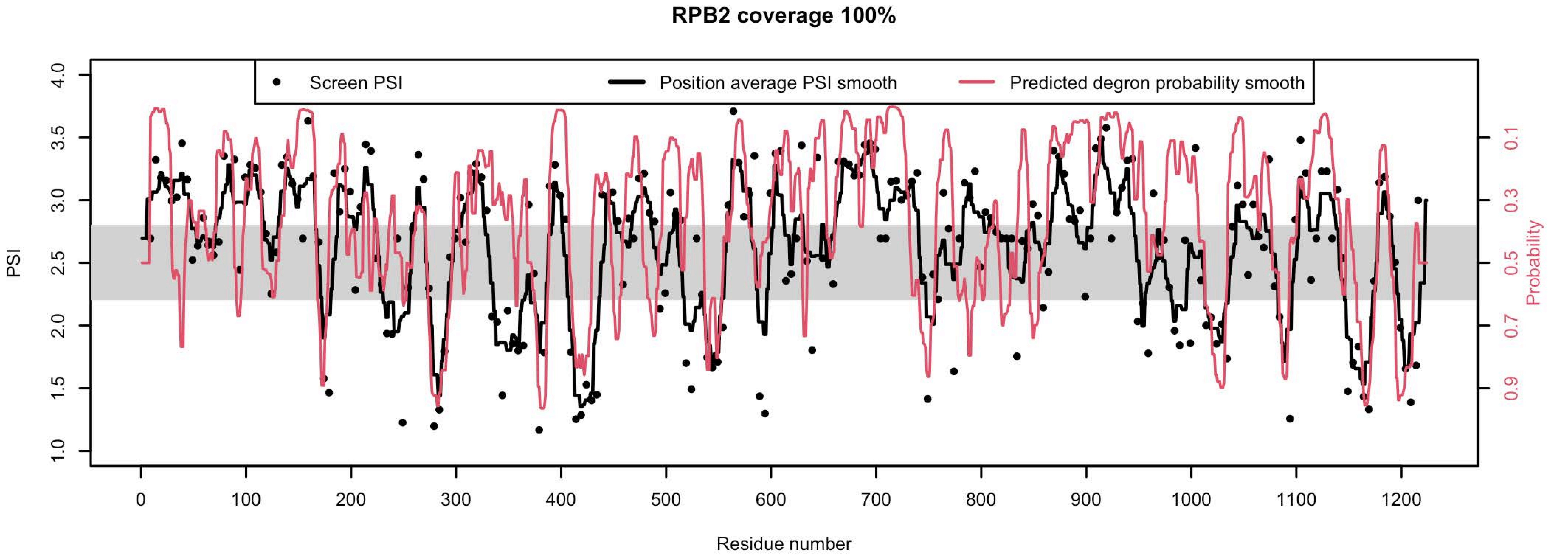

RPB2 coverage 100%
Screen PSI
Position average PSI smooth
Predicted degron probability smooth
PSI
Probability
Residue number
1.0
1.5
2.0
2.5
3.0
3.5
4.0
0.1
0.3
0.5
0.7
0.9
0
100
200
300
400
500
600
700
800
900
1000
1100
1200

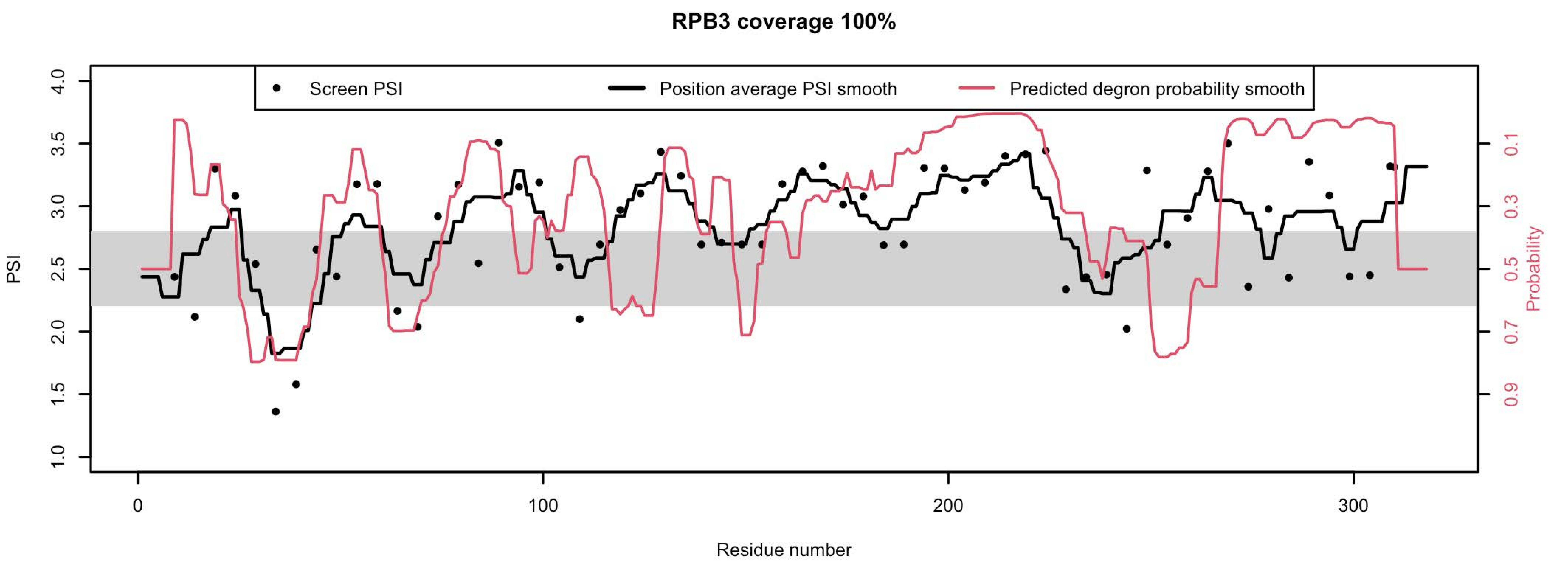

RPB3 coverage 100%
Screen PSI
Position average PSI smooth
Predicted degron probability smooth
PSI
1.0
1.5
2.0
2.5
3.0
3.5
4.0
Probability
0.1
0.3
0.5
0.7
0.9
0
100
200
300
Residue number

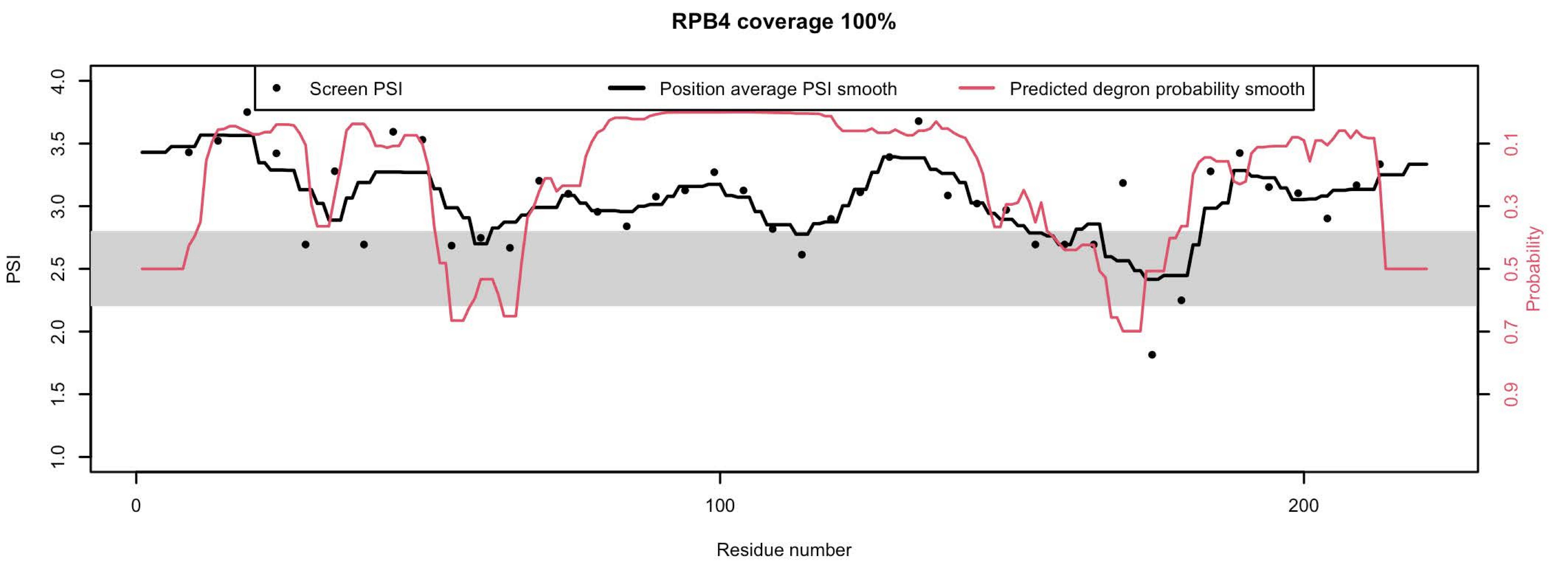

RPB4 coverage 100%
Screen PSI
Position average PSI smooth
Predicted degron probability smooth
PSI
1.0
1.5
2.0
2.5
3.0
3.5
4.0
Probability
0.1
0.3
0.5
0.7
0.9
0
100
200
Residue number

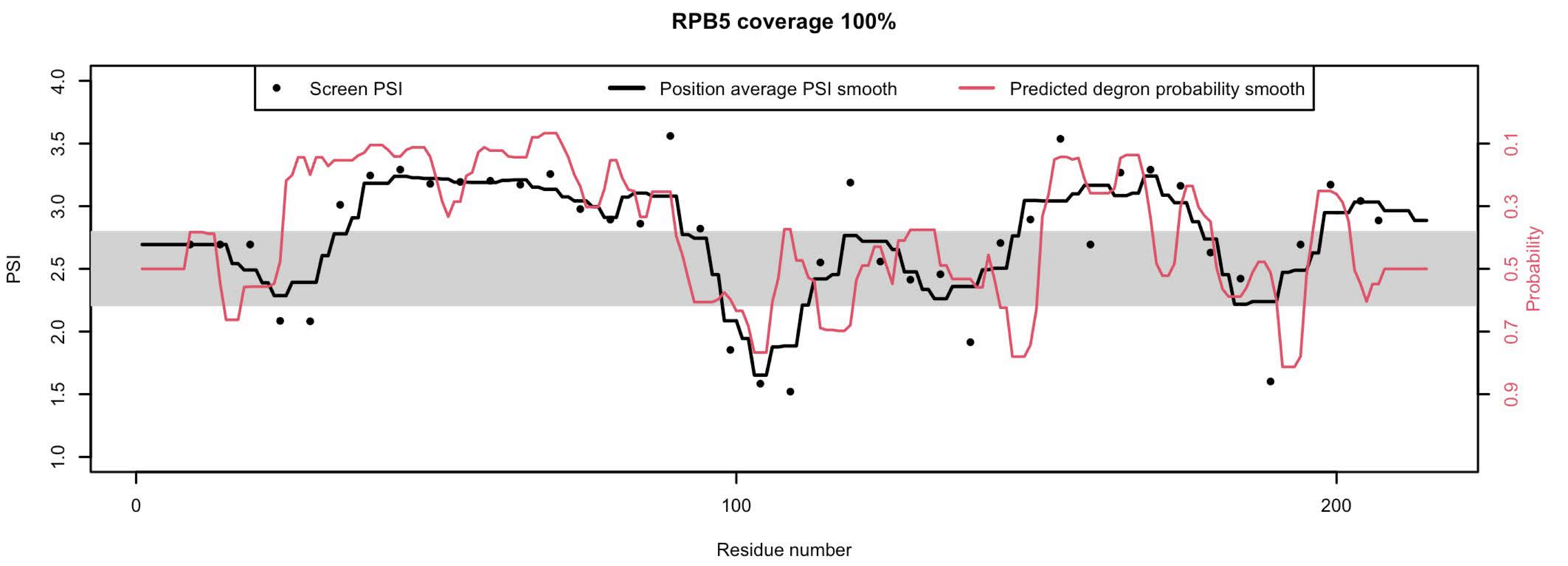
RPB5 coverage 100%
Screen PSI
Position average PSI smooth
Predicted degron probability smooth
PSI
1.0
1.5
2.0
2.5
3.0
3.5
4.0
Probability
0.1
0.3
0.5
0.7
0.9
0
100
200
Residue number

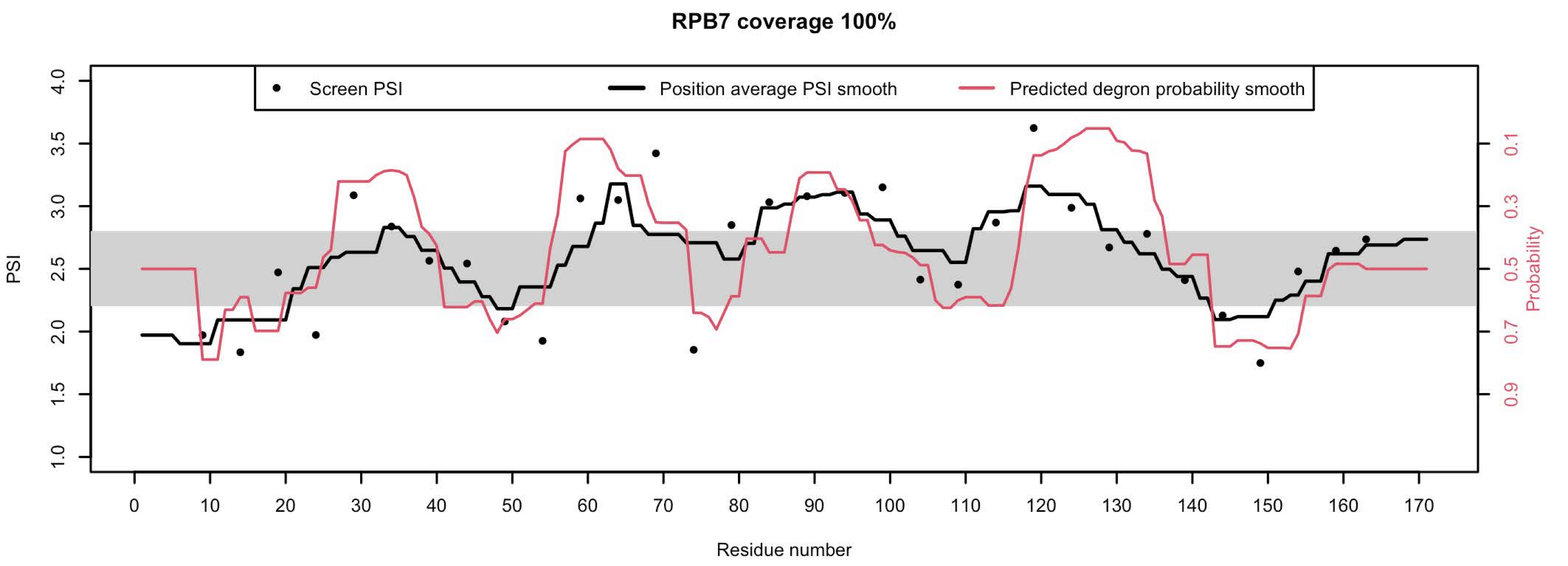

RPB7 coverage 100%
Screen PSI
Position average PSI smooth
Predicted degron probability smooth
PSI
1.0
1.5
2.0
2.5
3.0
3.5
4.0
Probability
0.1
0.3
0.5
0.7
0.9
Residue number
0
10
20
30
40
50
60
70
80
90
100
110
120
130
140
150
160
170

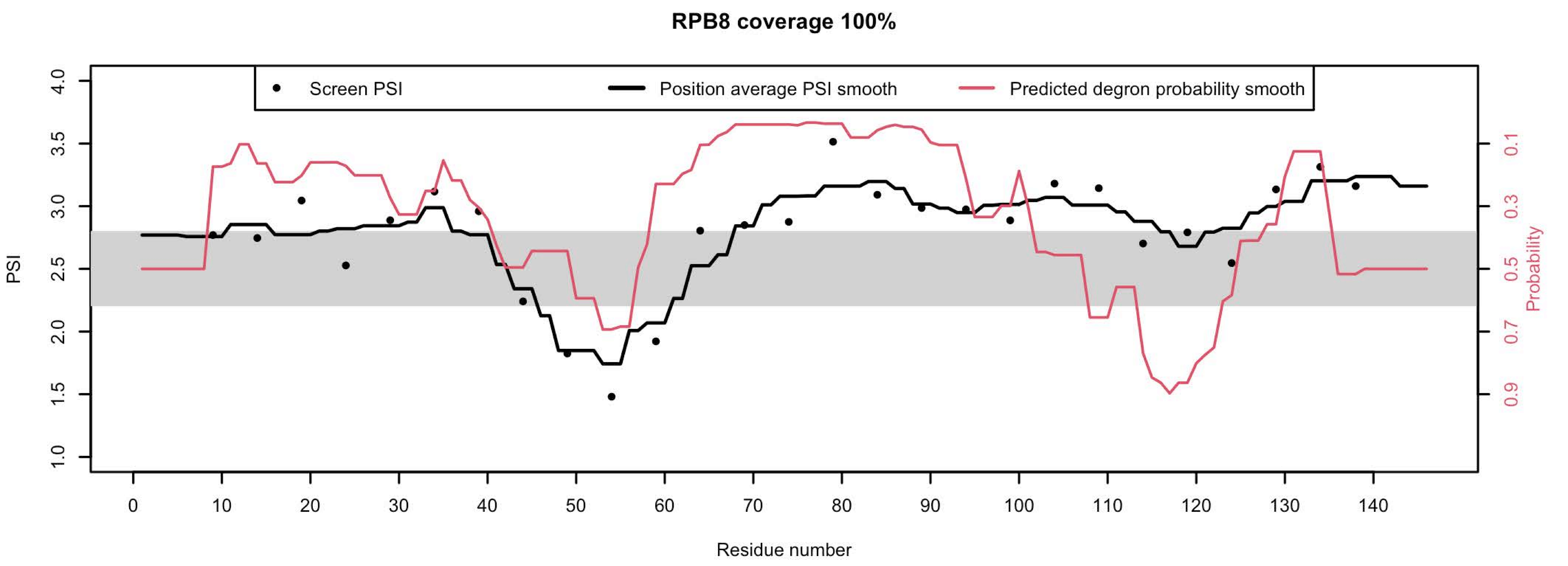

RPB8 coverage 100%
Screen PSI
Position average PSI smooth
Predicted degron probability smooth
PSI
1.0
1.5
2.0
2.5
3.0
3.5
4.0
Probability
0.1
0.3
0.5
0.7
0.9
0
10
20
30
40
50
60
70
80
90
100
110
120
130
140
Residue number

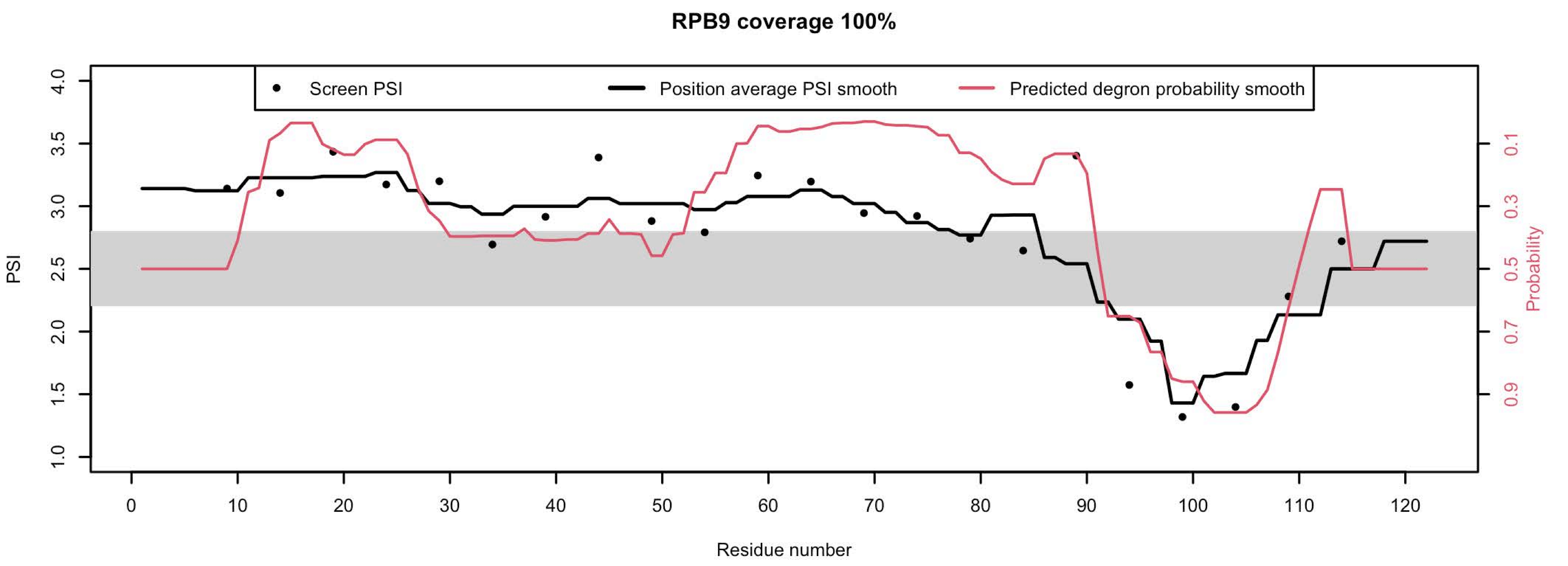
RPB9 coverage 100%
Screen PSI
Position average PSI smooth
Predicted degron probability smooth
PSI
1.0
1.5
2.0
2.5
3.0
3.5
4.0
Probability
0.1
0.3
0.5
0.7
0.9
Residue number
0
10
20
30
40
50
60
70
80
90
100
110
120

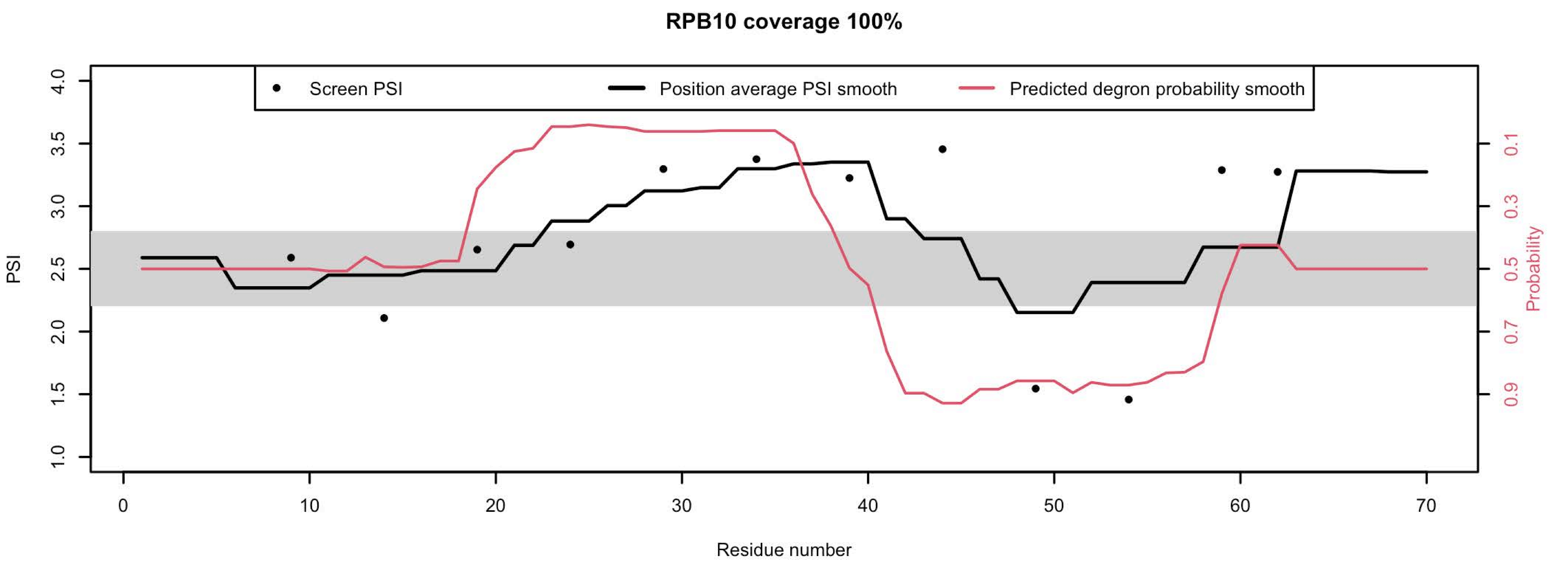
RPB10 coverage 100%
Screen PSI
Position average PSI smooth
Predicted degron probability smooth
PSI
Probability
Residue number
1.0
1.5
2.0
2.5
3.0
3.5
4.0
0.1
0.3
0.5
0.7
0.9
0
10
20
30
40
50
60
70

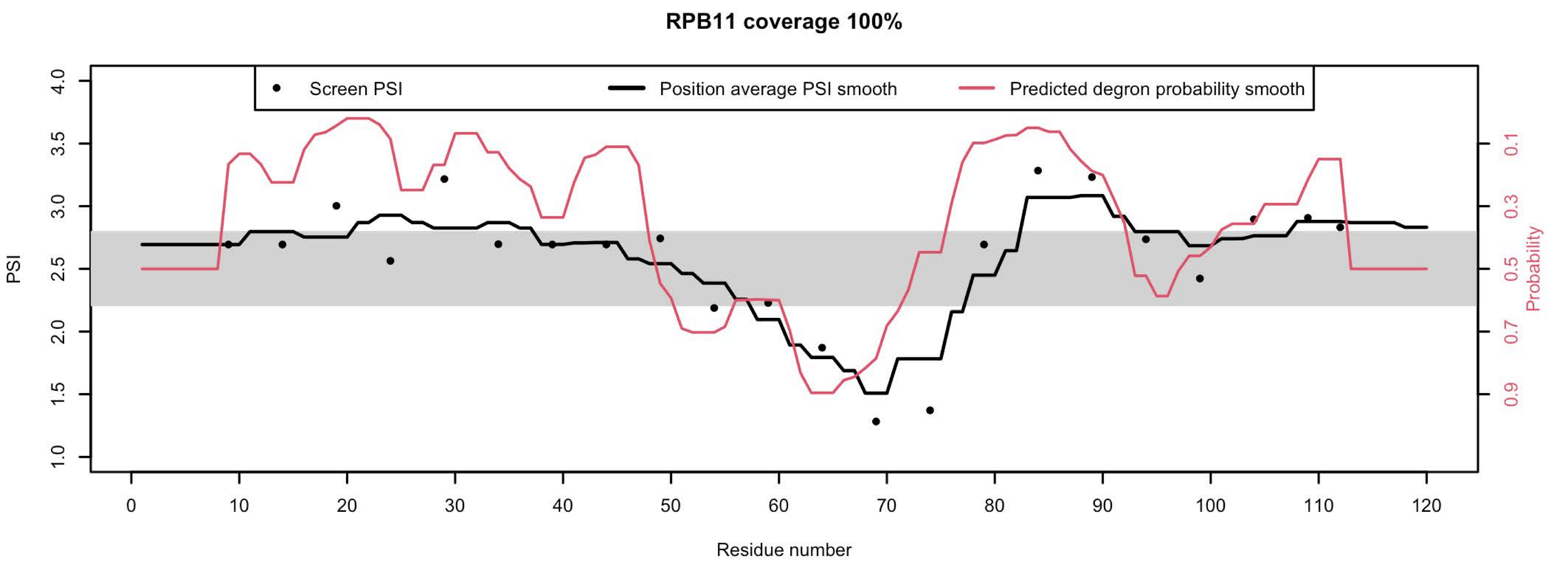

RPB11 coverage 100%
Screen PSI
Position average PSI smooth
Predicted degron probability smooth
PSI
1.0
1.5
2.0
2.5
3.0
3.5
4.0
Probability
0.1
0.3
0.5
0.7
0.9
0
10
20
30
40
50
60
70
80
90
100
110
120
Residue number

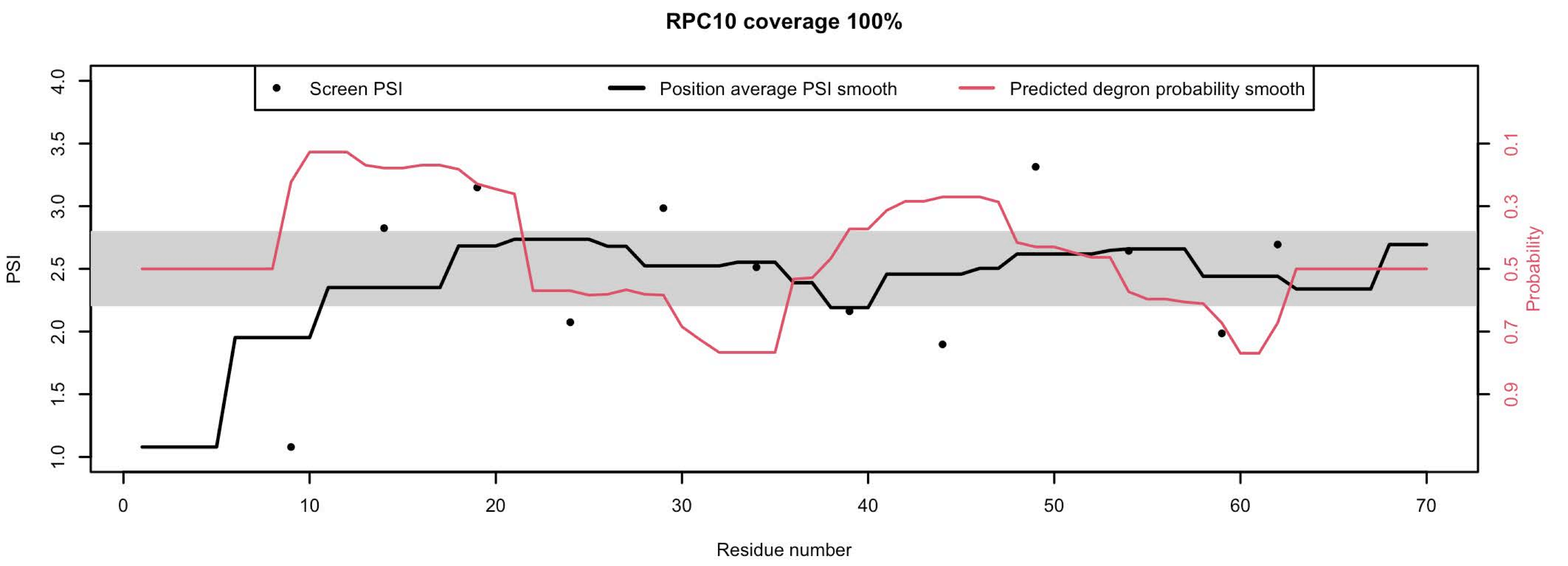

RPC10 coverage 100%
Screen PSI
Position average PSI smooth
Predicted degron probability smooth
PSI
4.0
3.5
3.0
2.5
2.0
1.5
1.0
0.1
0.3
0.5
0.7
0.9
Probability
0
10
20
30
40
50
60
70
Residue number

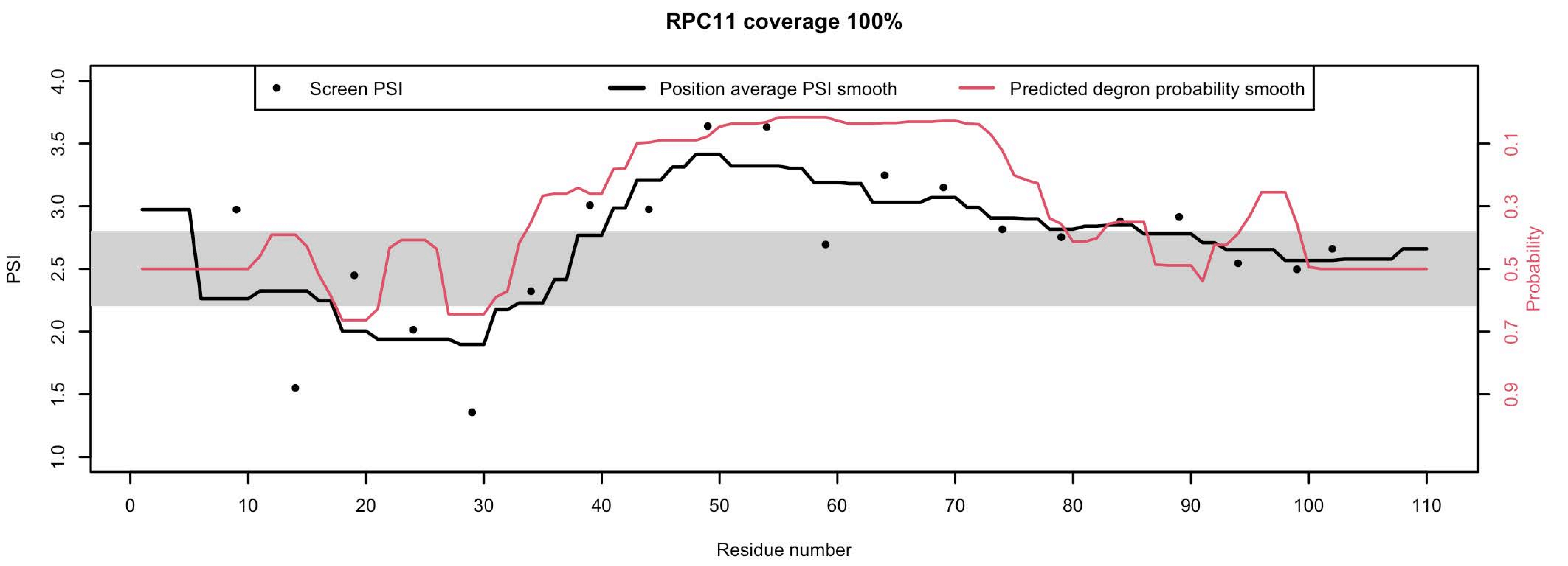
RPC11 coverage 100%
Screen PSI
Position average PSI smooth
Predicted degron probability smooth
PSI
Probability
Residue number
1.0
1.5
2.0
2.5
3.0
3.5
4.0
0.1
0.3
0.5
0.7
0.9
0
10
20
30
40
50
60
70
80
90
100
110

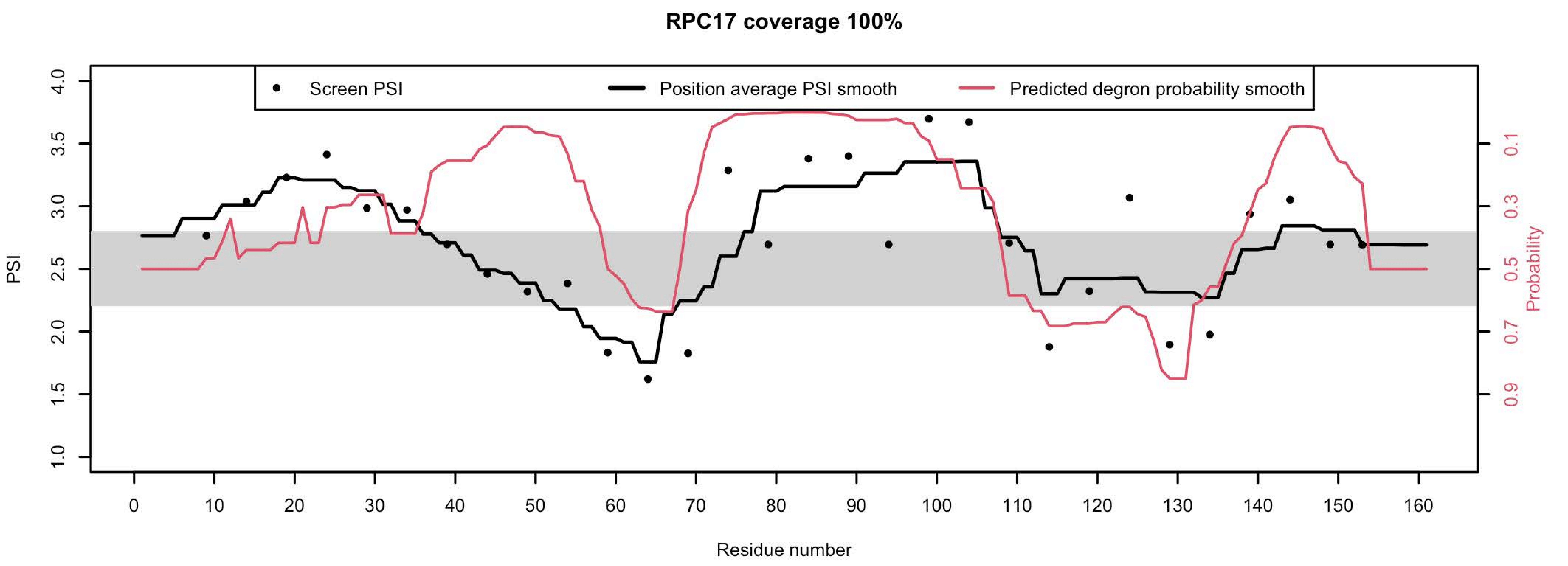

RPC17 coverage 100%
Screen PSI
Position average PSI smooth
Predicted degron probability smooth
PSI
Probability
Residue number
1.0
1.5
2.0
2.5
3.0
3.5
4.0
0.1
0.3
0.5
0.7
0.9
0
10
20
30
40
50
60
70
80
90
100
110
120
130
140
150
160

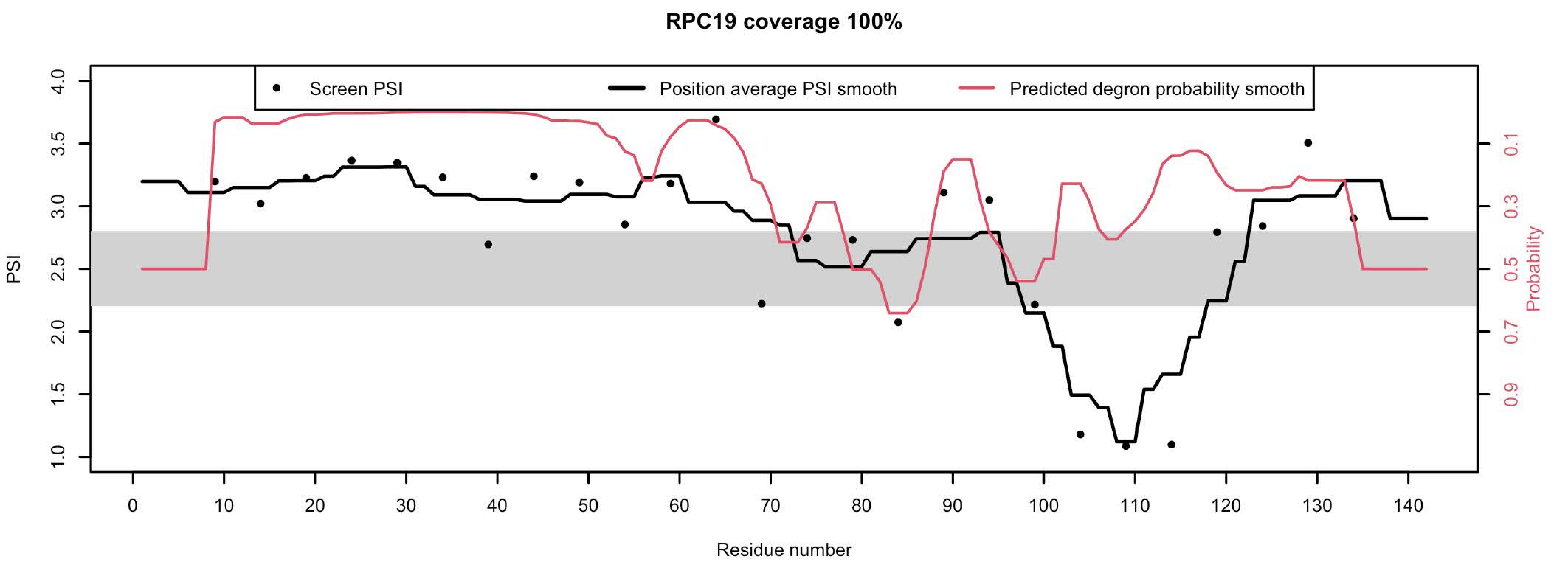

RPC19 coverage 100%
Screen PSI
Position average PSI smooth
Predicted degron probability smooth
PSI
Probability
Residue number
1.0
1.5
2.0
2.5
3.0
3.5
4.0
0.1
0.3
0.5
0.7
0.9
0
10
20
30
40
50
60
70
80
90
100
110
120
130
140

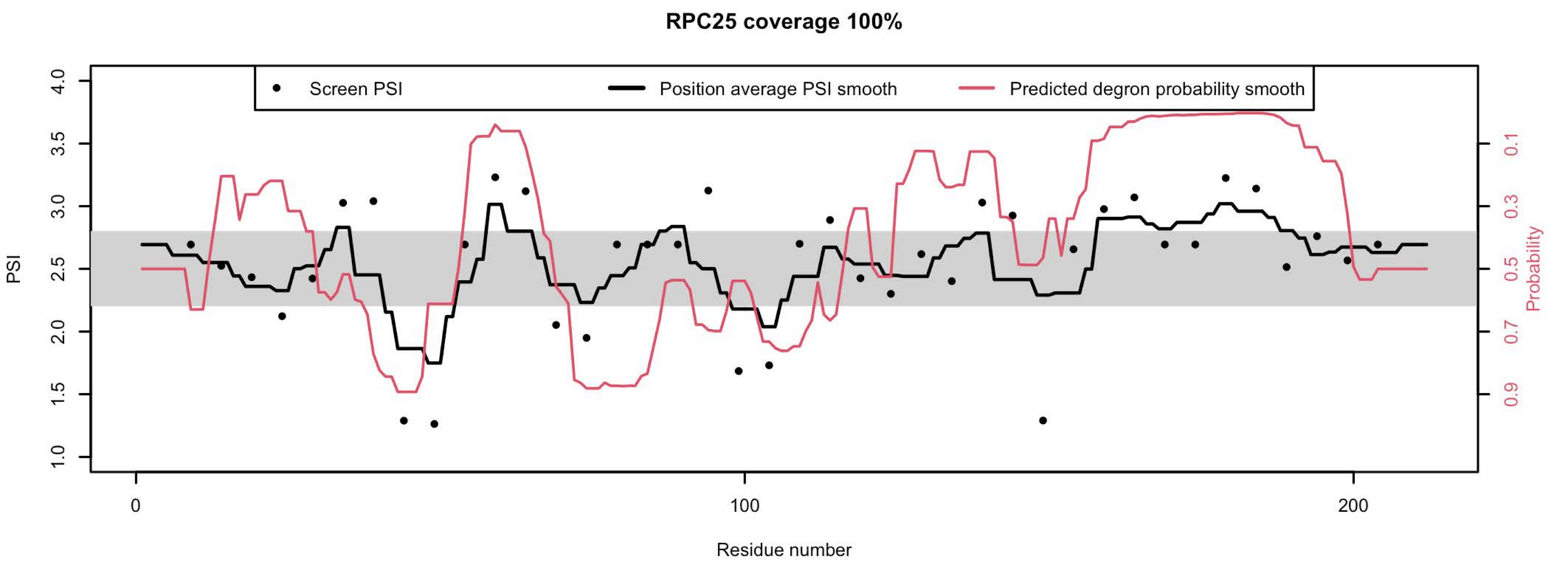

RPC25 coverage 100%
Screen PSI
Position average PSI smooth
Predicted degron probability smooth
PSI
4.0
3.5
3.0
2.5
2.0
1.5
1.0
Probability
0.1
0.3
0.5
0.7
0.9
0
100
200
Residue number

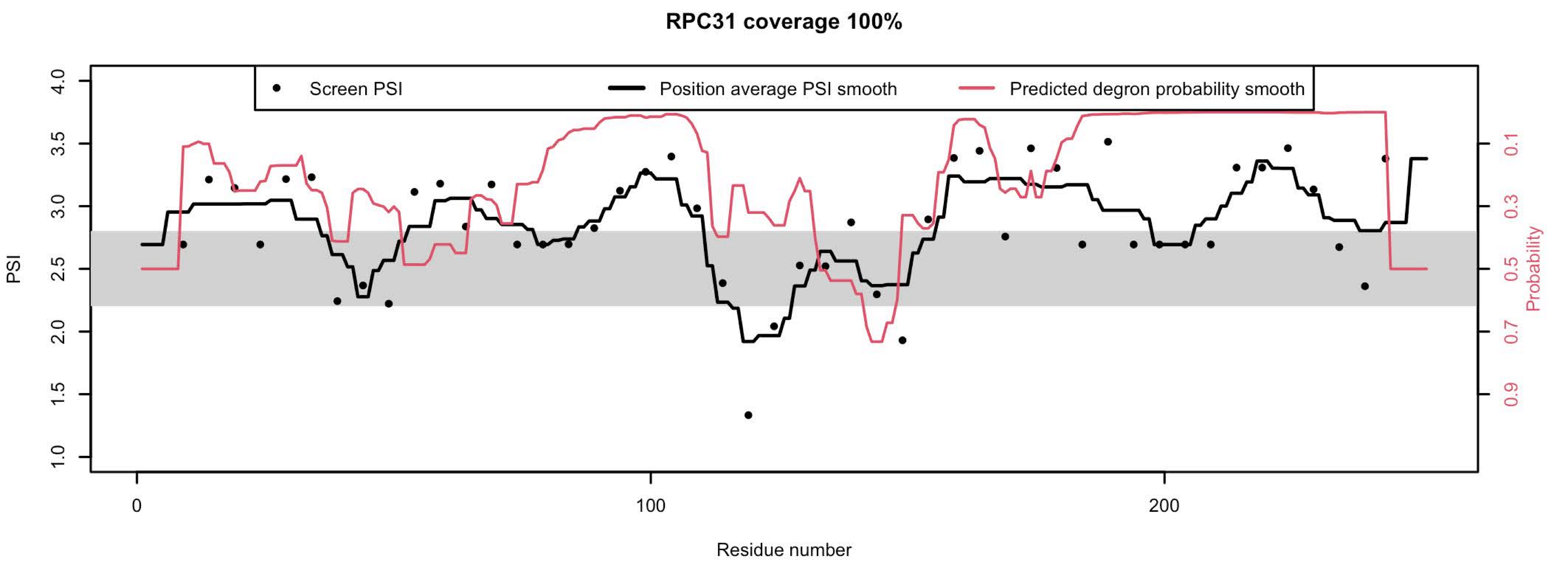
RPC31 coverage 100%
Screen PSI
Position average PSI smooth
Predicted degron probability smooth
PSI
1.0
1.5
2.0
2.5
3.0
3.5
4.0
Probability
0.1
0.3
0.5
0.7
0.9
0
100
200
Residue number

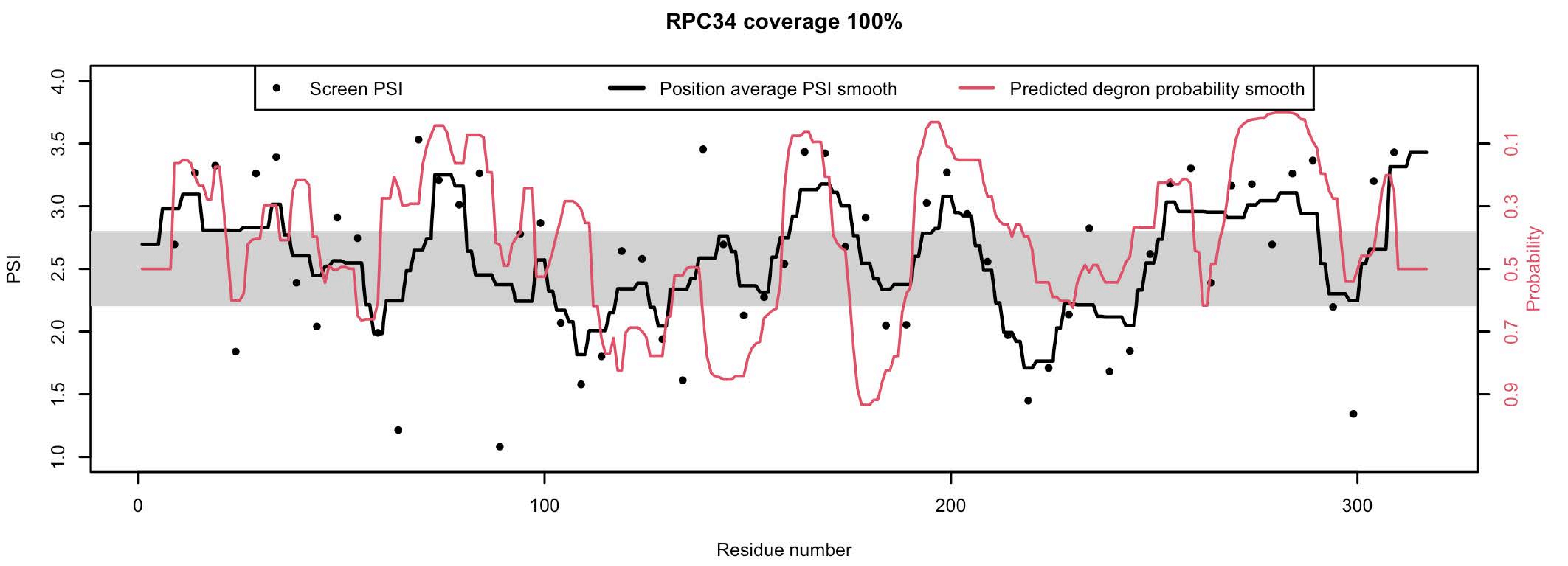
RPC34 coverage 100%
Screen PSI
Position average PSI smooth
Predicted degron probability smooth
PSI
1.0
1.5
2.0
2.5
3.0
3.5
4.0
Probability
0.1
0.3
0.5
0.7
0.9
0
100
200
300
Residue number

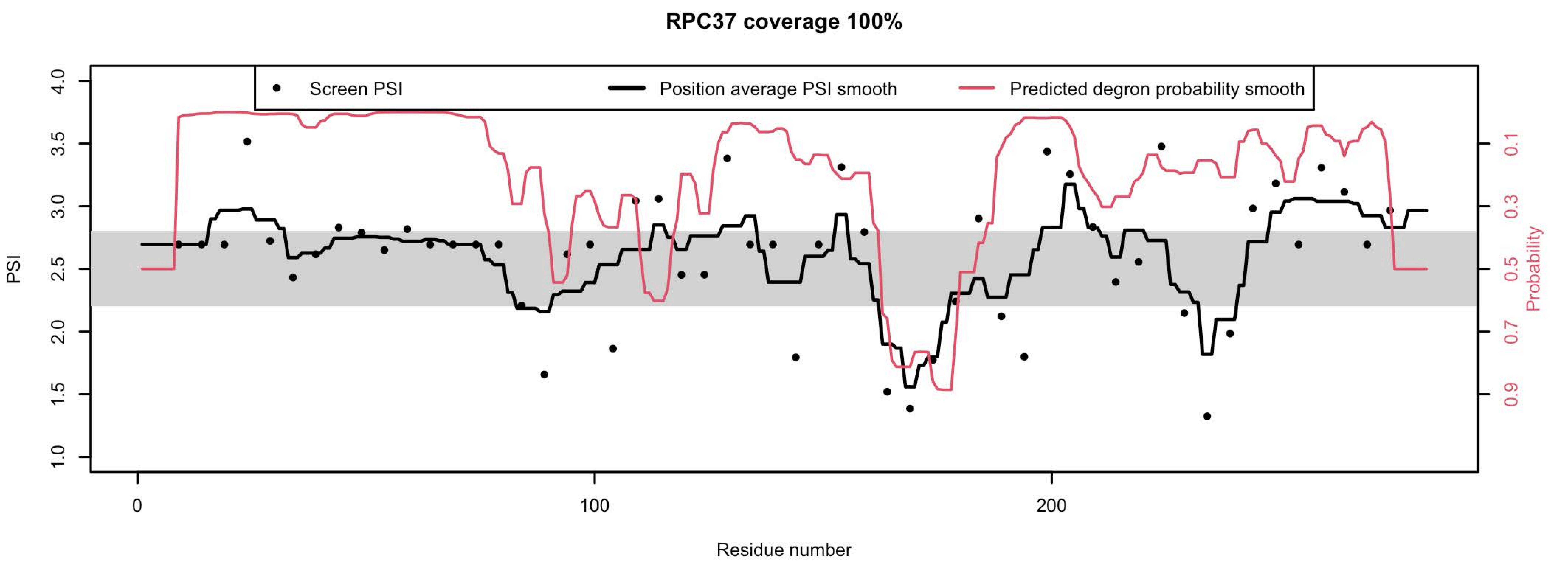

RPC37 coverage 100%
Screen PSI
Position average PSI smooth
Predicted degron probability smooth
PSI
1.0
1.5
2.0
2.5
3.0
3.5
4.0
Probability
0.1
0.3
0.5
0.7
0.9
0
100
200
Residue number

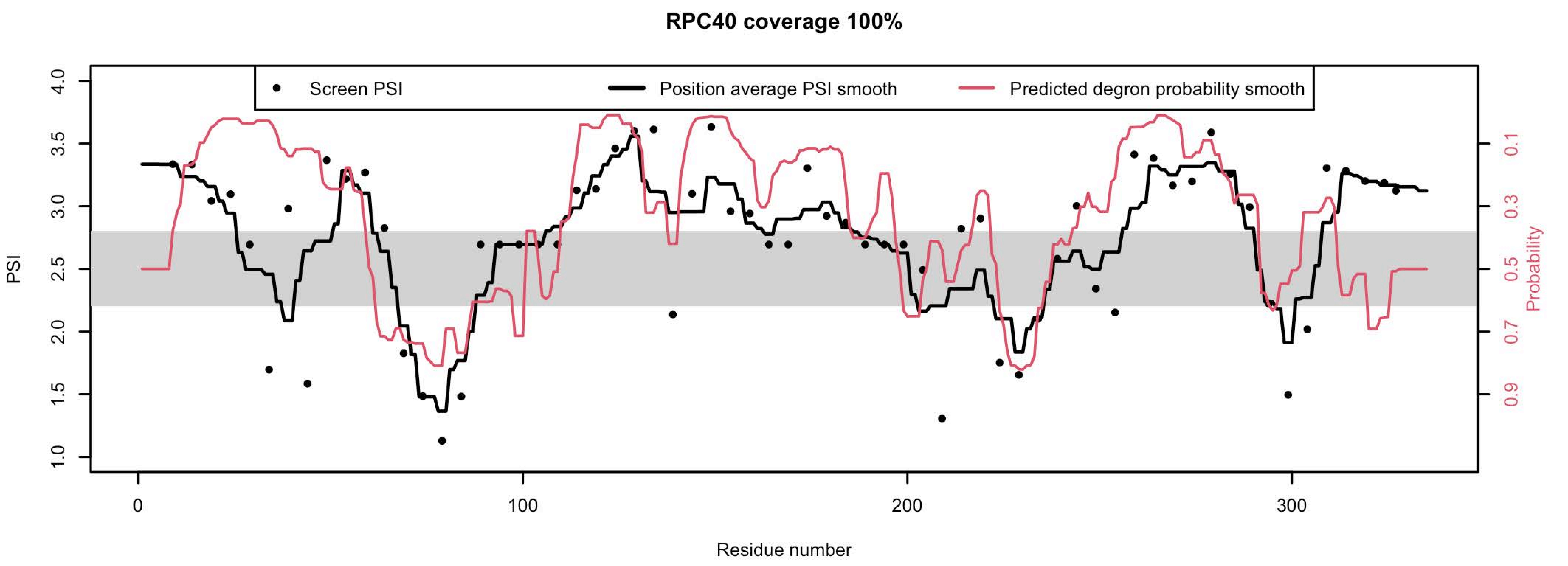

RPC40 coverage 100%
Screen PSI
Position average PSI smooth
Predicted degron probability smooth
PSI
1.0
1.5
2.0
2.5
3.0
3.5
4.0
Probability
0.1
0.3
0.5
0.7
0.9
0
100
200
300
Residue number

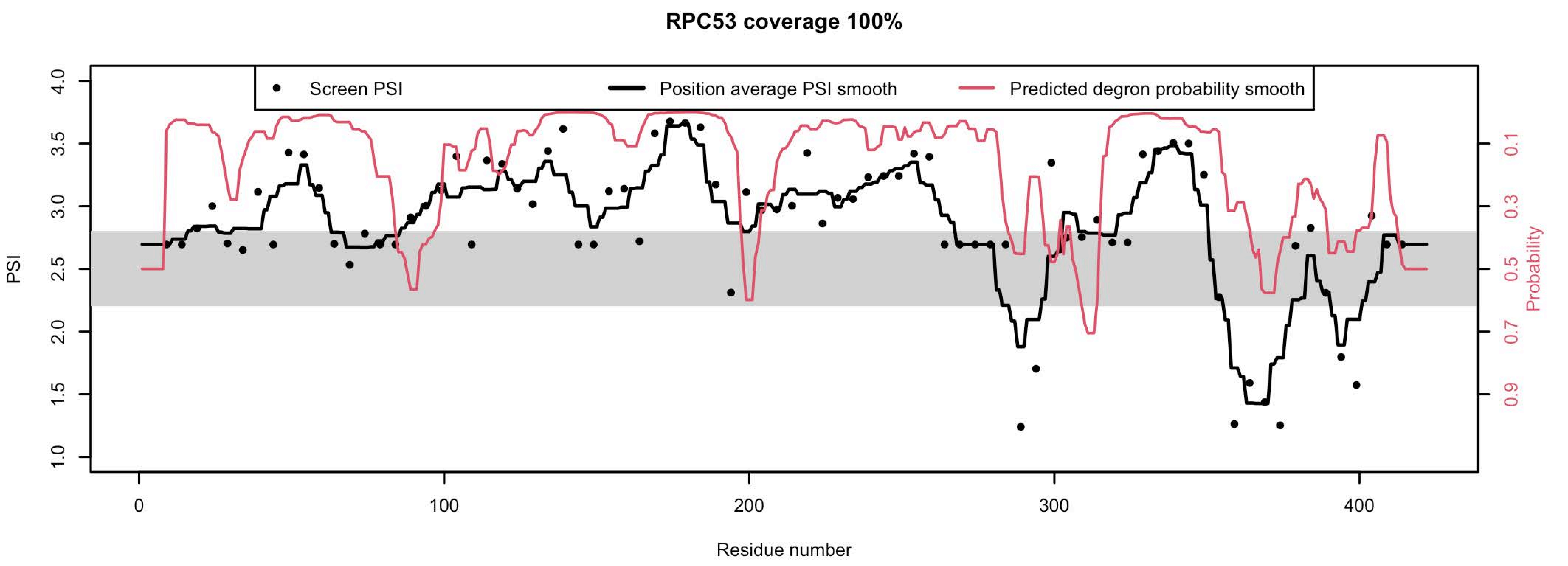

RPC53 coverage 100%
Screen PSI
Position average PSI smooth
Predicted degron probability smooth
PSI
Probability
Residue number
1.0
1.5
2.0
2.5
3.0
3.5
4.0
0.1
0.3
0.5
0.7
0.9
0
100
200
300
400

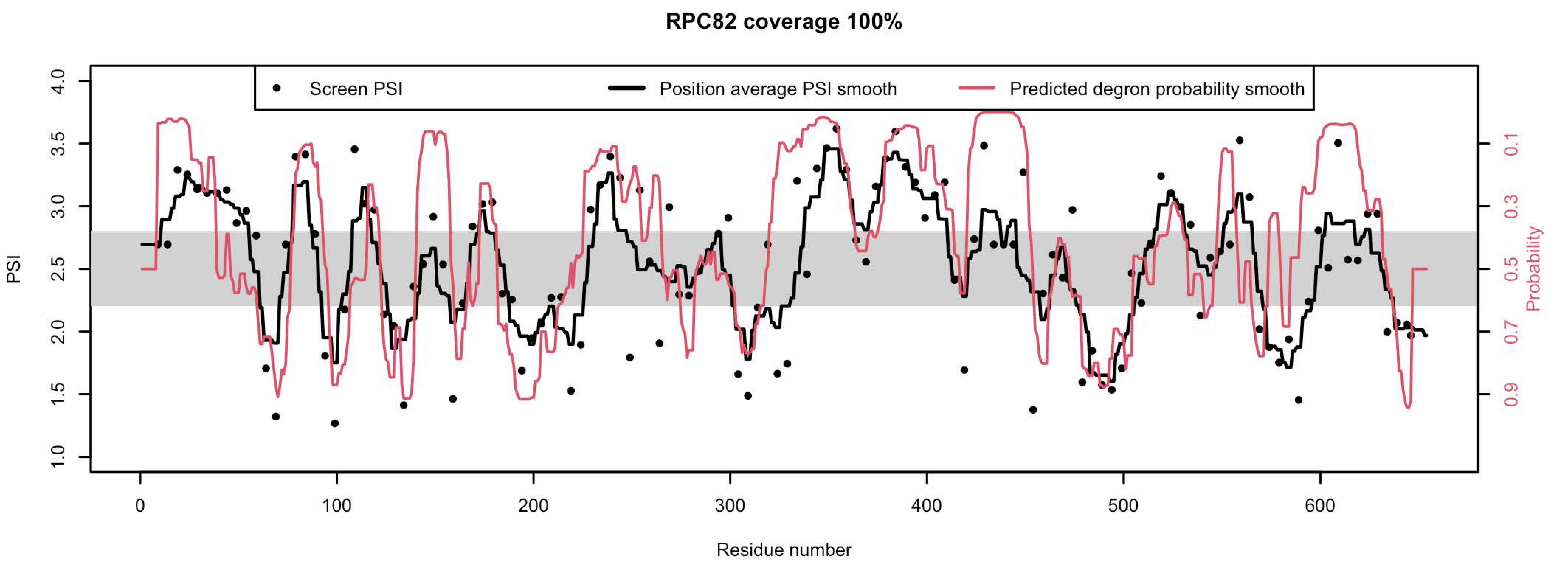

RPC82 coverage 100%
Screen PSI
Position average PSI smooth
Predicted degron probability smooth
PSI
Probability
Residue number

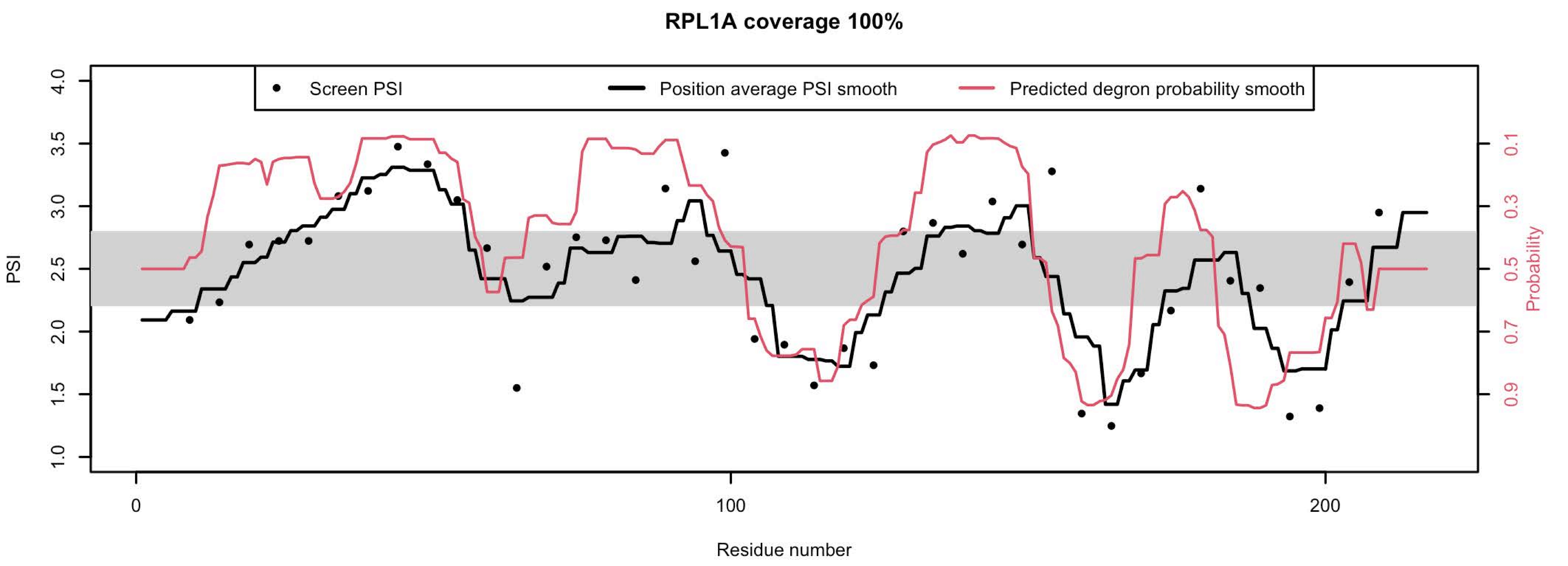

RPL1A coverage 100%
Screen PSI
Position average PSI smooth
Predicted degron probability smooth
PSI
Probability
Residue number
1.0
1.5
2.0
2.5
3.0
3.5
4.0
0.1
0.3
0.5
0.7
0.9
0
100
200

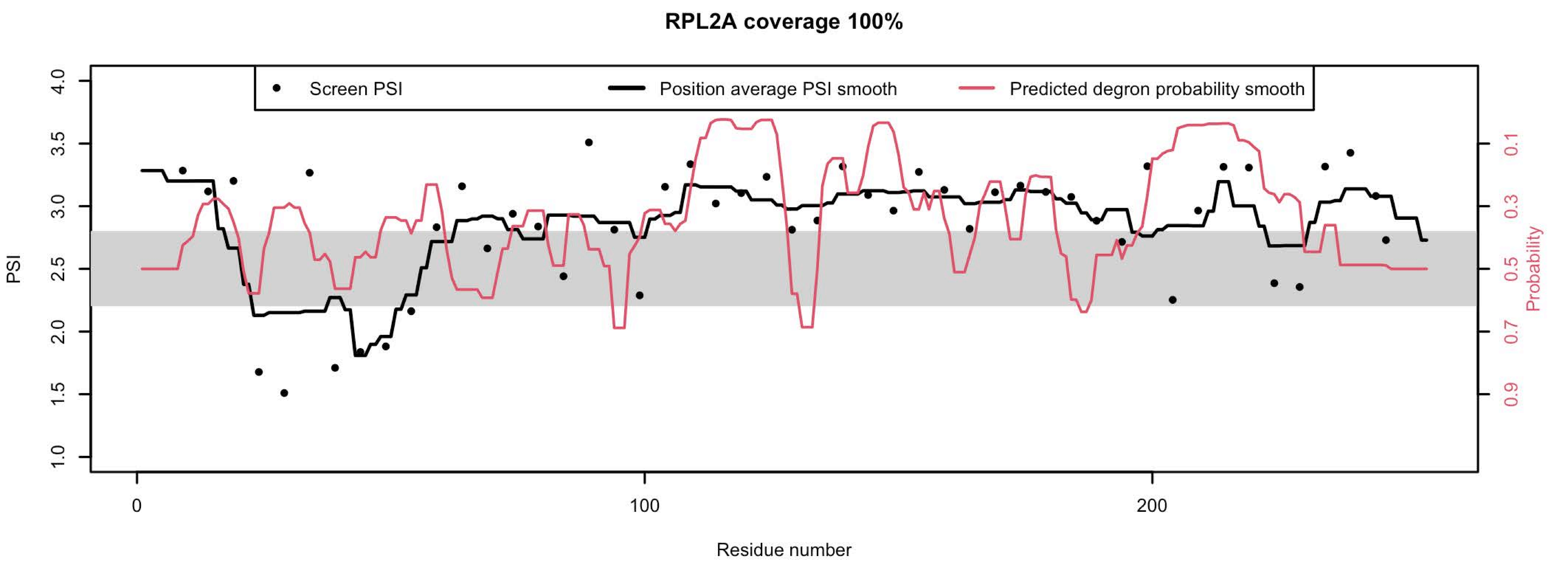

RPL2A coverage 100%
Screen PSI
Position average PSI smooth
Predicted degron probability smooth
PSI
4.0
3.5
3.0
2.5
2.0
1.5
1.0
Probability
0.1
0.3
0.5
0.7
0.9
0
100
200
Residue number

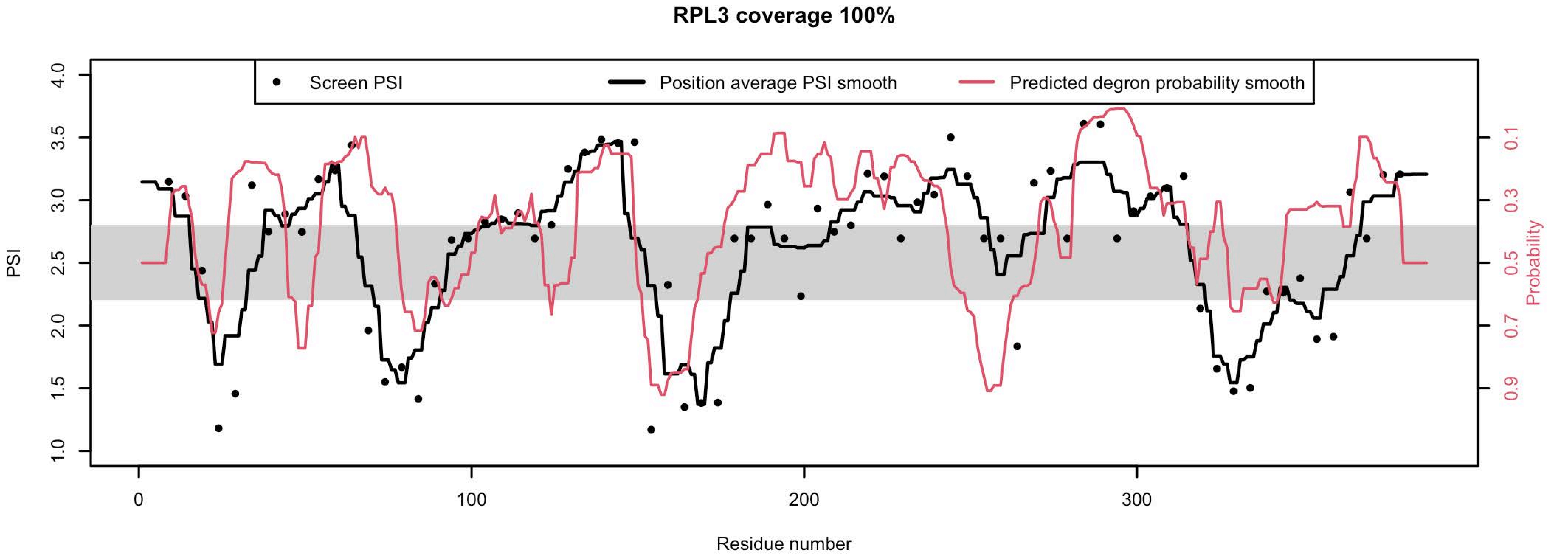

RPL3 coverage 100%
Screen PSI
Position average PSI smooth
Predicted degron probability smooth
PSI
1.0
1.5
2.0
2.5
3.0
3.5
4.0
Probability
0.1
0.3
0.5
0.7
0.9
0
100
200
300
Residue number

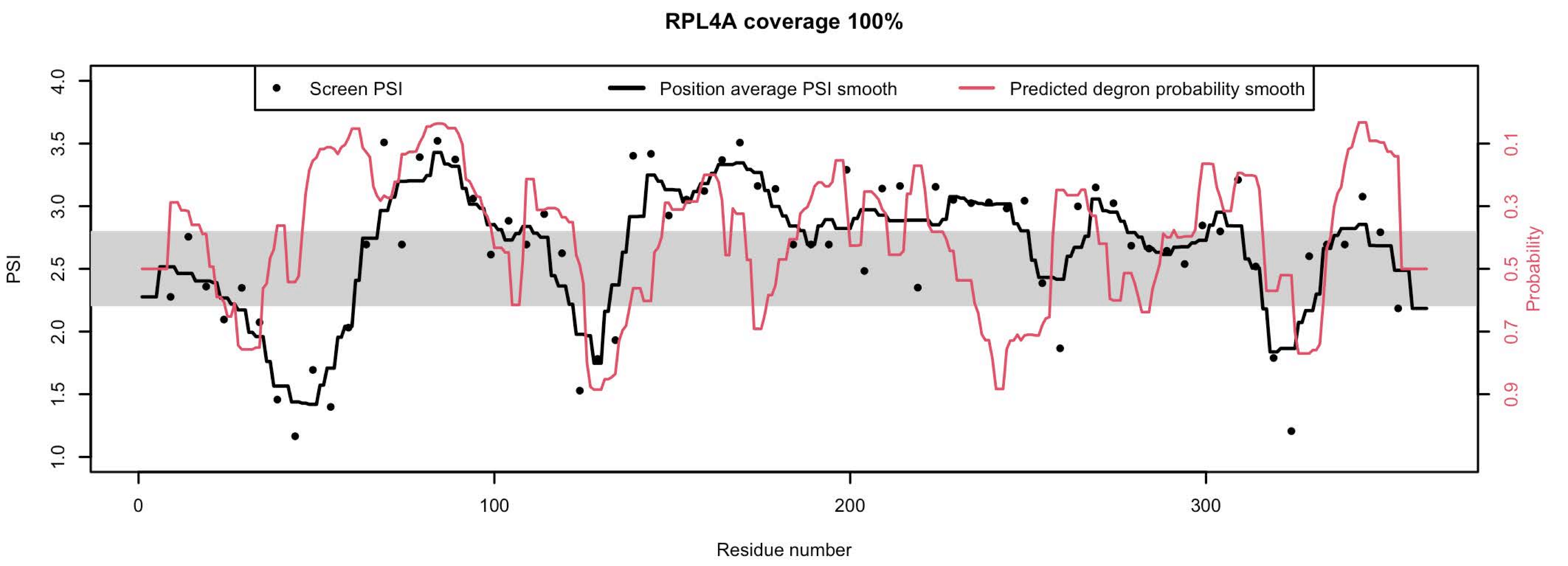
RPL4A coverage 100%
Screen PSI
Position average PSI smooth
Predicted degron probability smooth
PSI
4.0
3.5
3.0
2.5
2.0
1.5
1.0
Probability
0.1
0.3
0.5
0.7
0.9
0
100
200
300
Residue number

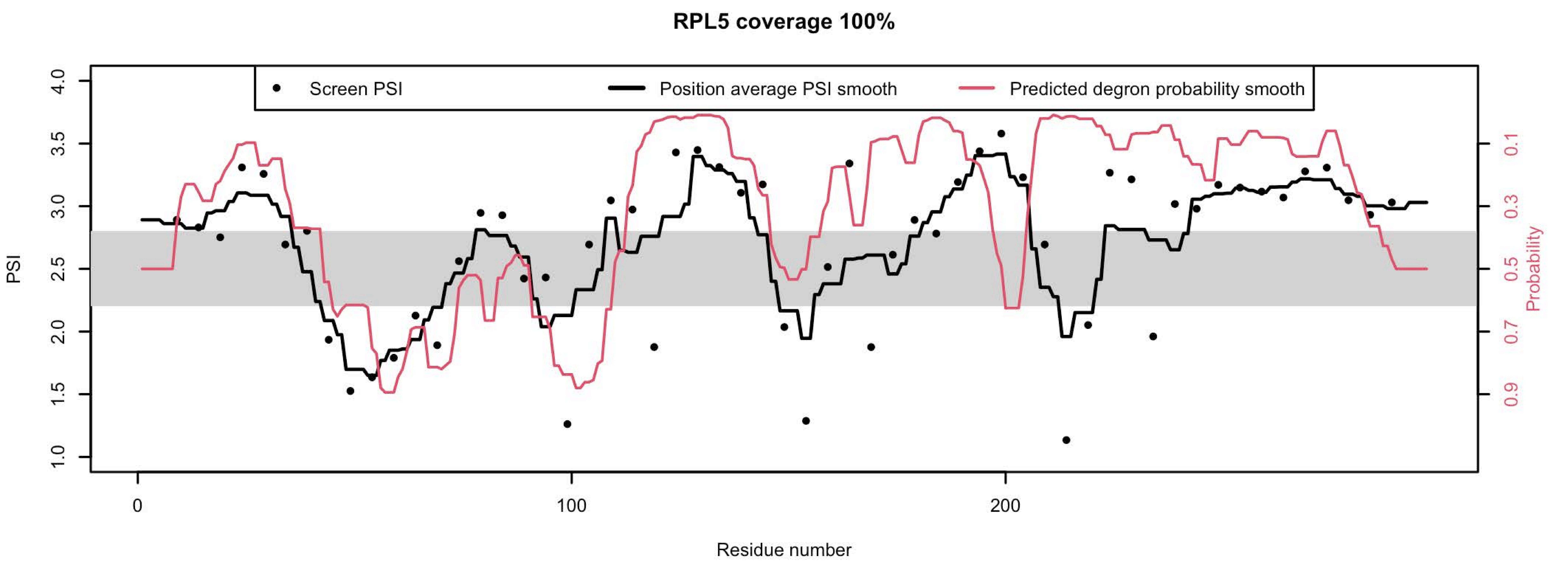
RPL5 coverage 100%
Screen PSI
Position average PSI smooth
Predicted degron probability smooth
PSI
1.0
1.5
2.0
2.5
3.0
3.5
4.0
Probability
0.1
0.3
0.5
0.7
0.9
0
100
200
Residue number

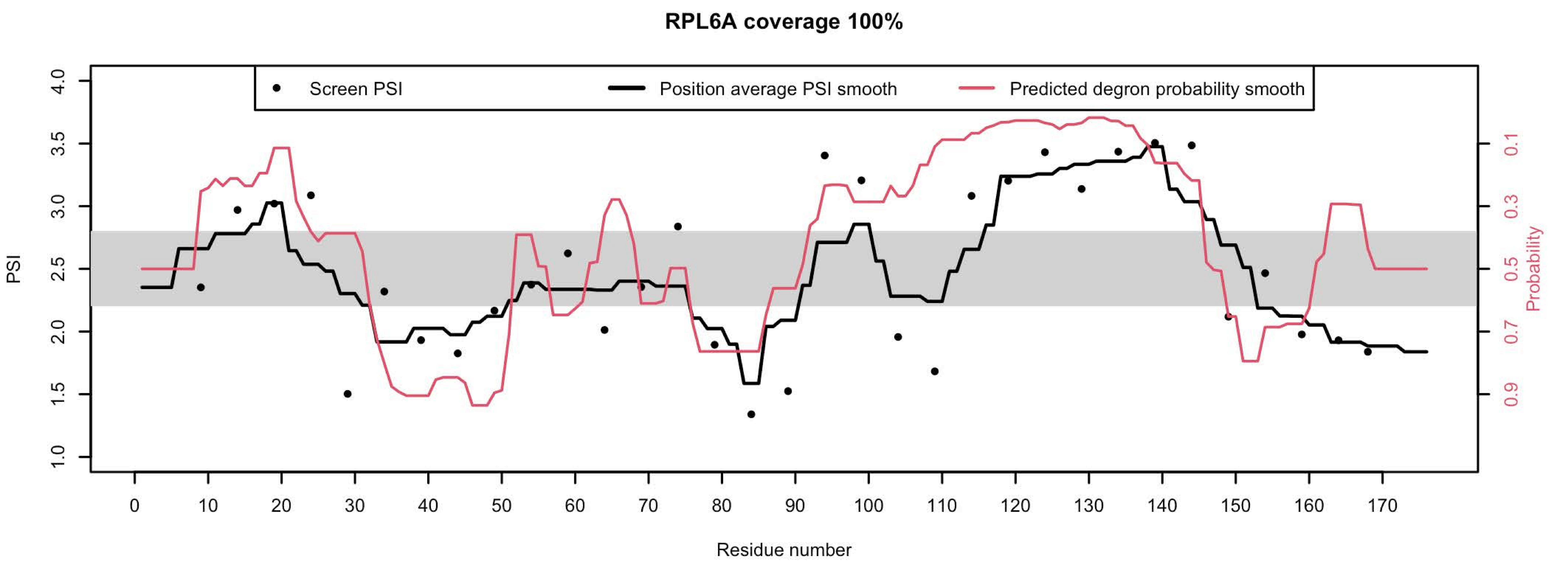

RPL6A coverage 100%
Screen PSI
Position average PSI smooth
Predicted degron probability smooth
PSI
4.0
3.5
3.0
2.5
2.0
1.5
1.0
Probability
0.1
0.3
0.5
0.7
0.9
0
10
20
30
40
50
60
70
80
90
100
110
120
130
140
150
160
170
Residue number

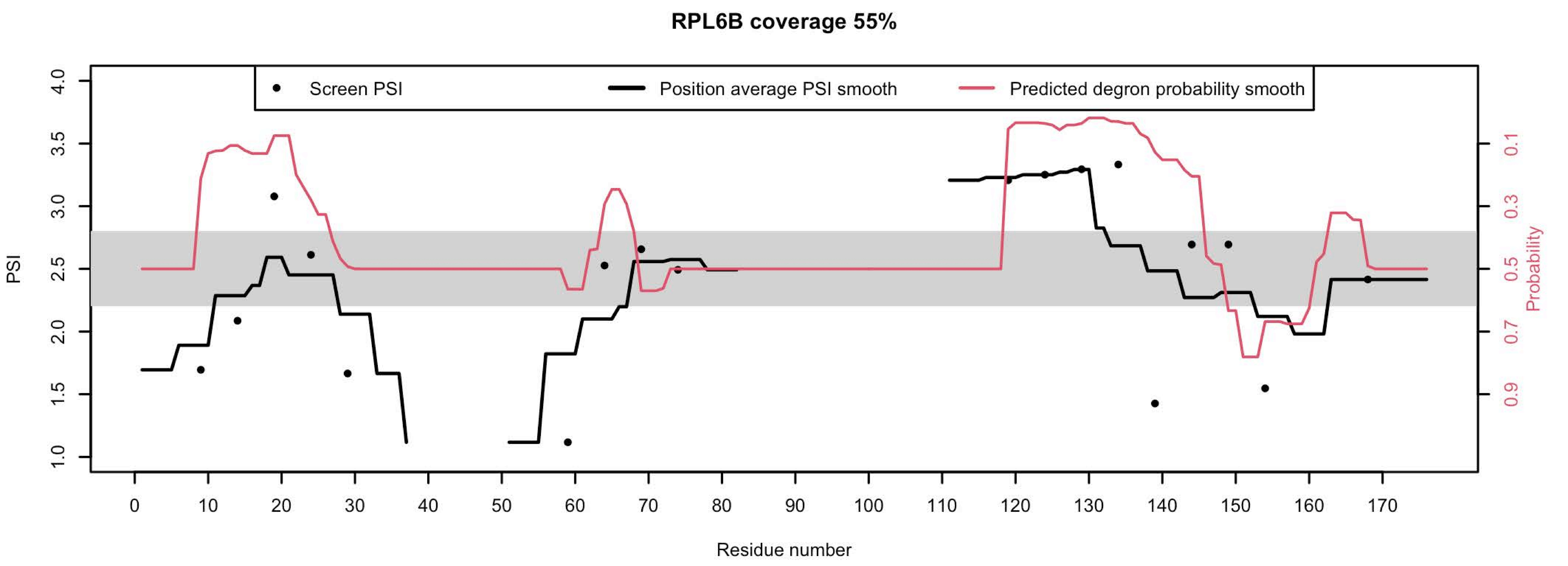

RPL6B coverage 55%
Screen PSI
Position average PSI smooth
Predicted degron probability smooth
PSI
1.0
1.5
2.0
2.5
3.0
3.5
4.0
Probability
0.1
0.3
0.5
0.7
0.9
Residue number
0
10
20
30
40
50
60
70
80
90
100
110
120
130
140
150
160
170

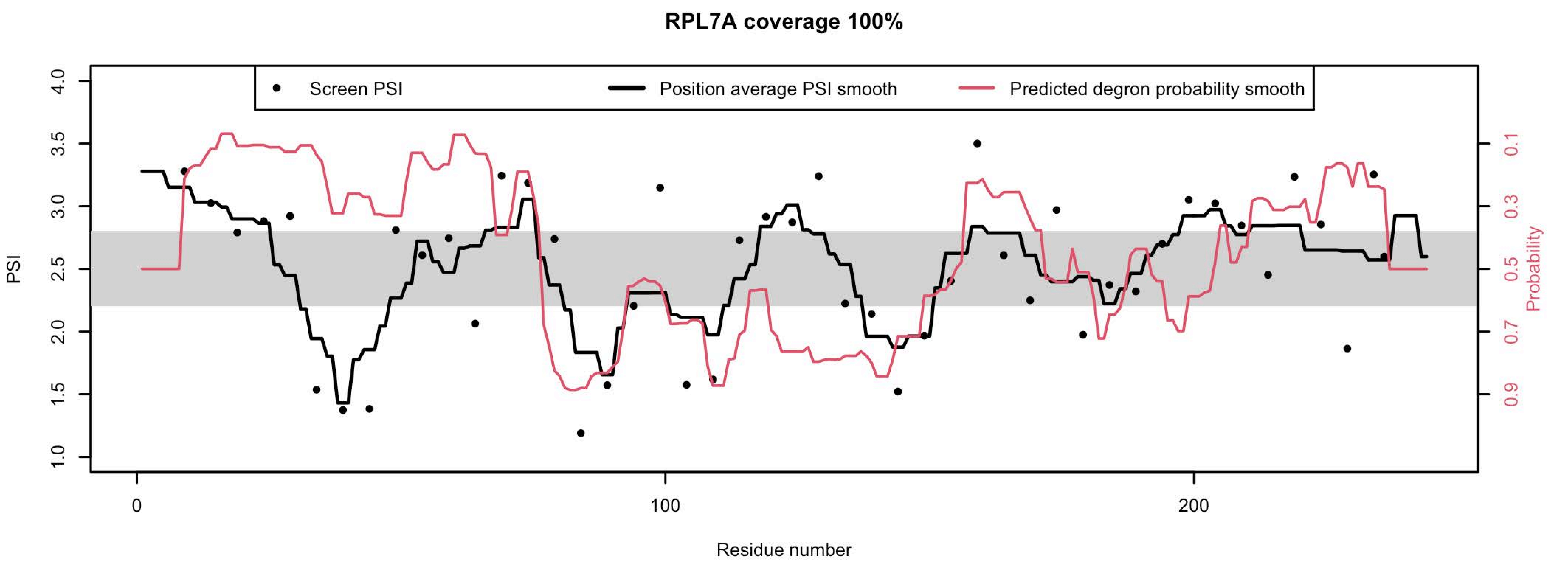
RPL7A coverage 100%
Screen PSI
Position average PSI smooth
Predicted degron probability smooth
PSI
1.0
1.5
2.0
2.5
3.0
3.5
4.0
Probability
0.1
0.3
0.5
0.7
0.9
Residue number
0
100
200

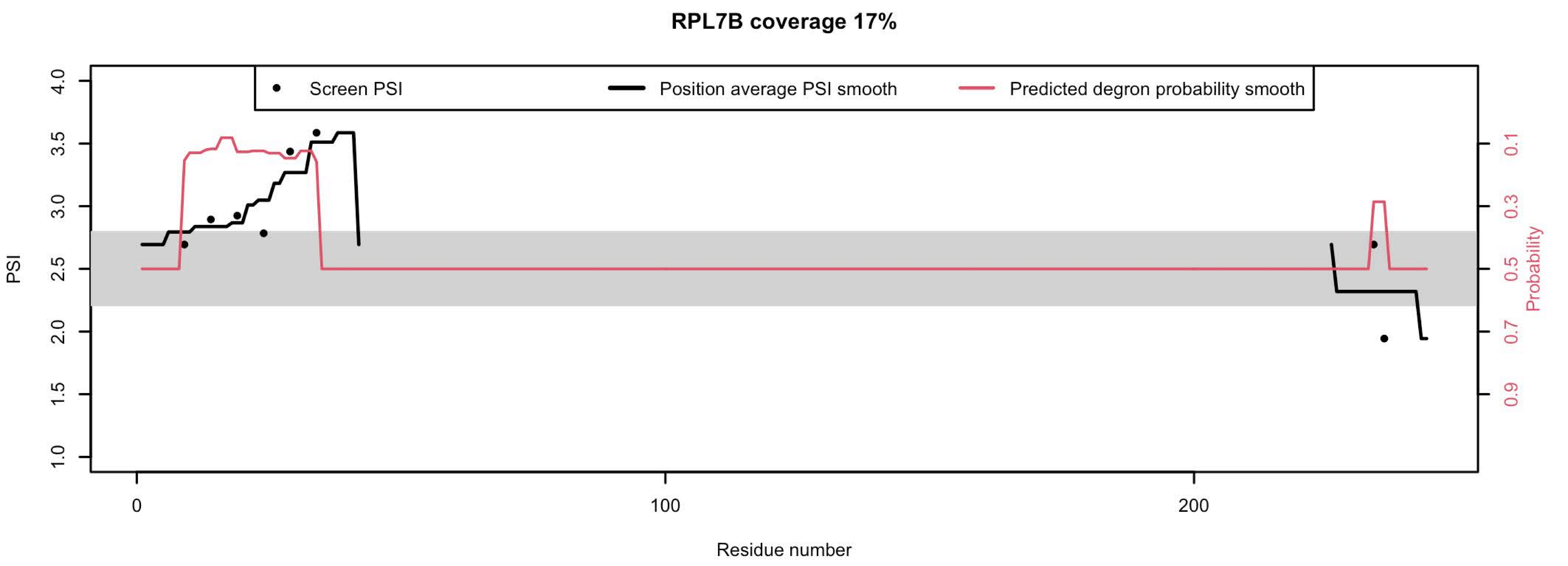

RPL7B coverage 17%
Screen PSI
Position average PSI smooth
Predicted degron probability smooth
PSI
1.0
1.5
2.0
2.5
3.0
3.5
4.0
Probability
0.1
0.3
0.5
0.7
0.9
0
100
200
Residue number

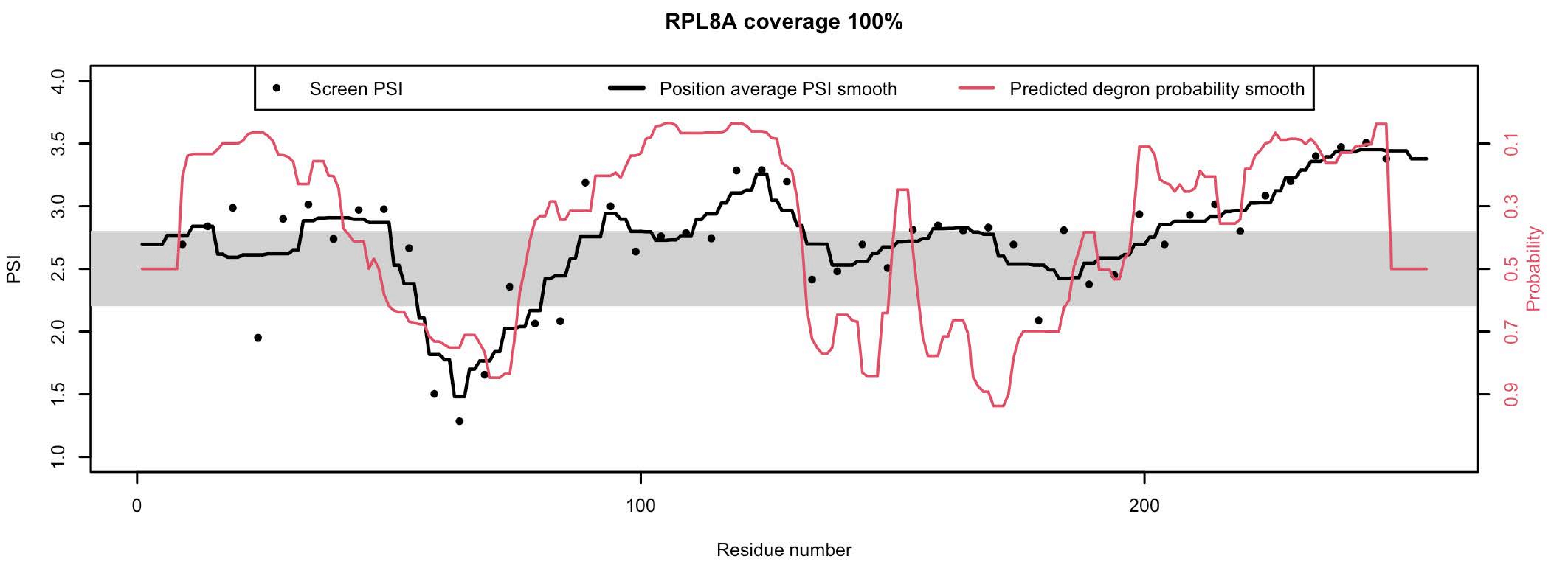
RPL8A coverage 100%
Screen PSI
Position average PSI smooth
Predicted degron probability smooth
PSI
4.0
3.5
3.0
2.5
2.0
1.5
1.0
Probability
0.1
0.3
0.5
0.7
0.9
0
100
200
Residue number

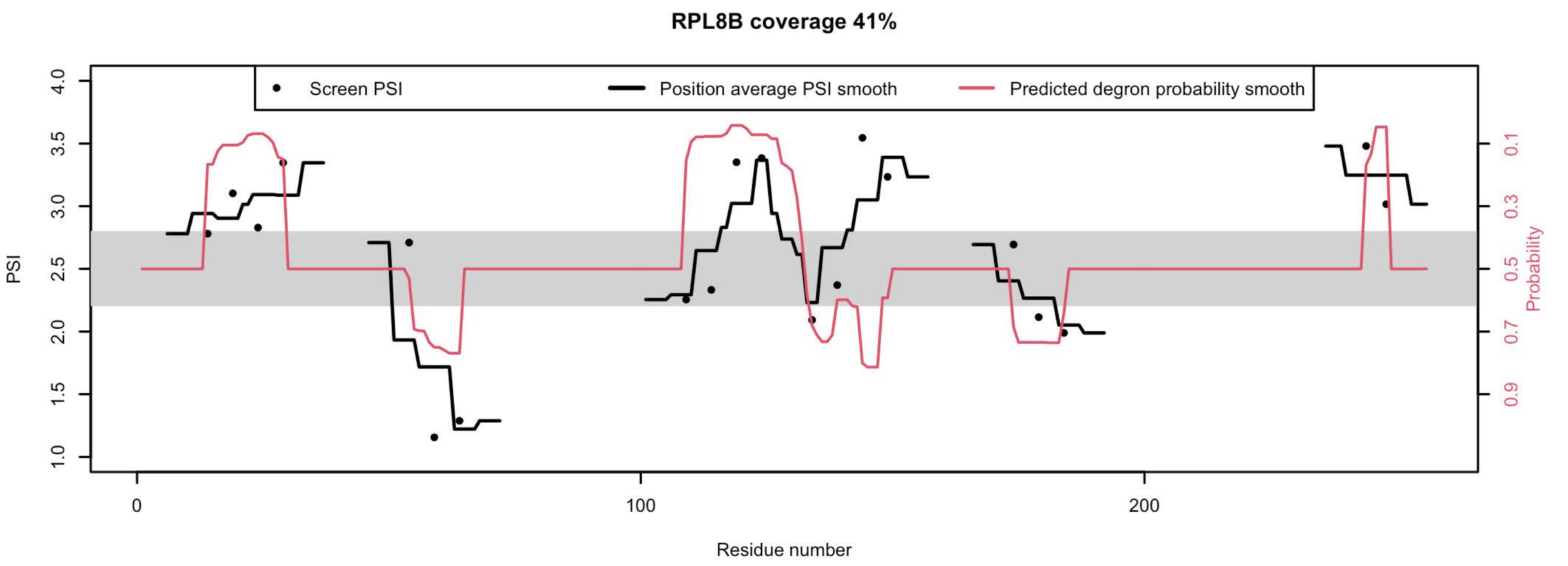

RPL8B coverage 41%
Screen PSI
Position average PSI smooth
Predicted degron probability smooth
PSI
4.0
3.5
3.0
2.5
2.0
1.5
1.0
Probability
0.1
0.3
0.5
0.7
0.9
0
100
200
Residue number

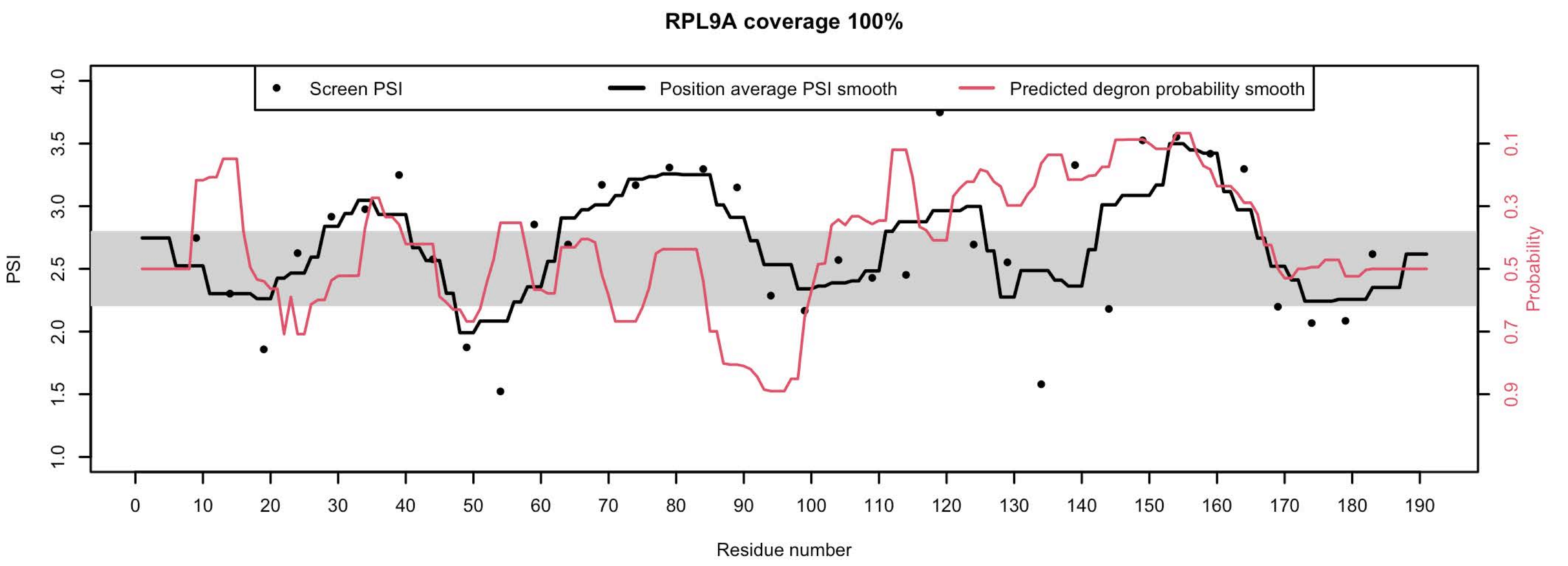

RPL9A coverage 100%
Screen PSI
Position average PSI smooth
Predicted degron probability smooth
PSI
Probability
Residue number
1.0
1.5
2.0
2.5
3.0
3.5
4.0
0.1
0.3
0.5
0.7
0.9
0
10
20
30
40
50
60
70
80
90
100
110
120
130
140
150
160
170
180
190

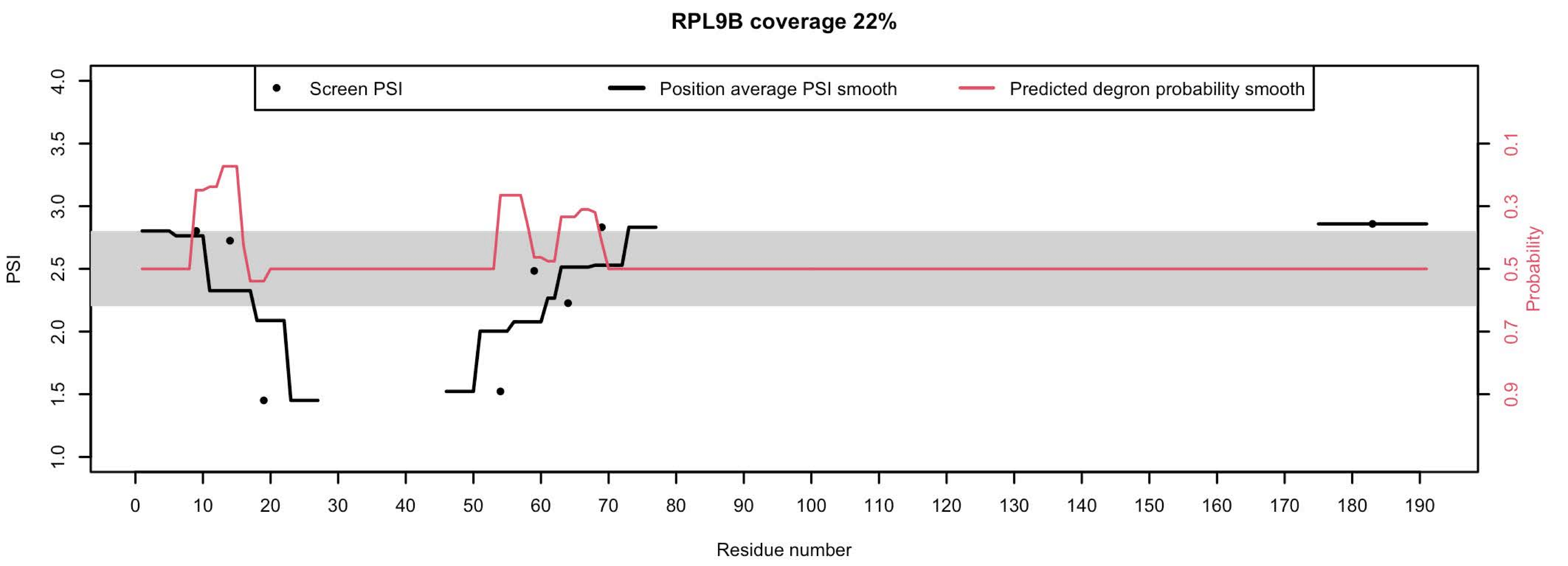
RPL9B coverage 22%
Screen PSI
Position average PSI smooth
Predicted degron probability smooth
PSI
4.0
3.5
3.0
2.5
2.0
1.5
1.0
Probability
0.1
0.3
0.5
0.7
0.9
0
10
20
30
40
50
60
70
80
90
100
110
120
130
140
150
160
170
180
190
Residue number

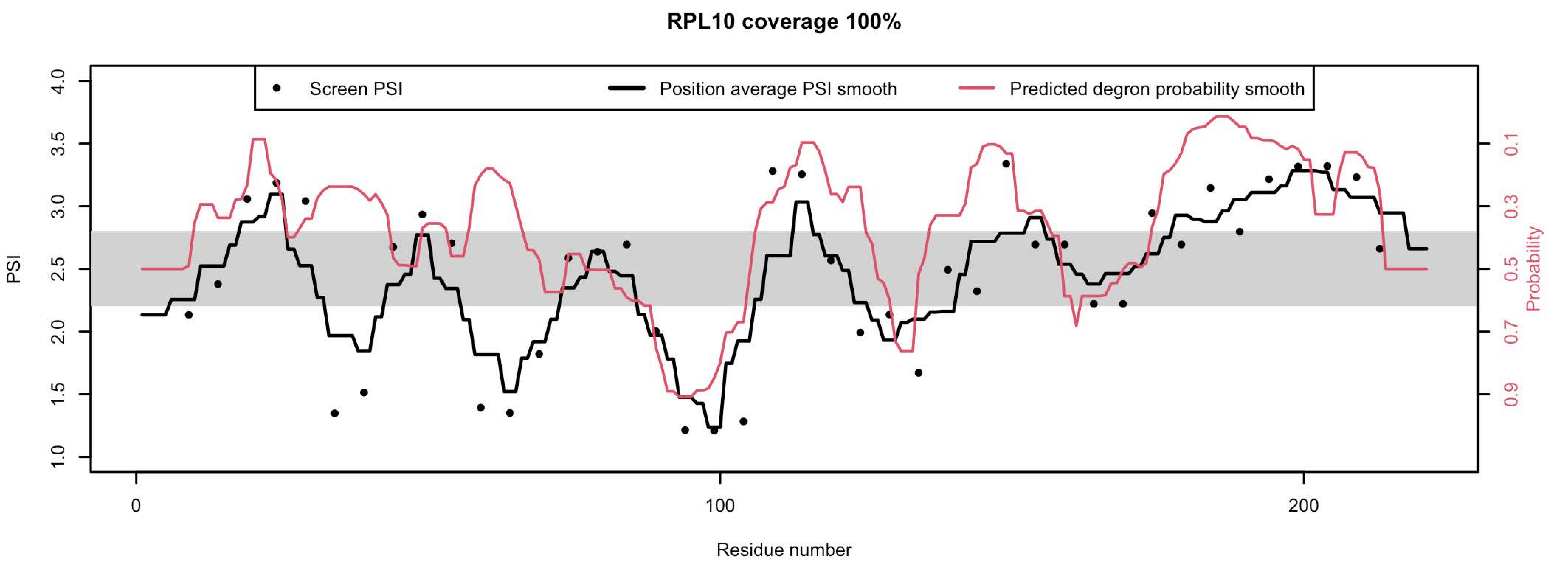

RPL10 coverage 100%
Screen PSI
Position average PSI smooth
Predicted degron probability smooth
PSI
4.0
3.5
3.0
2.5
2.0
1.5
1.0
Probability
0.1
0.3
0.5
0.7
0.9
0
100
200
Residue number

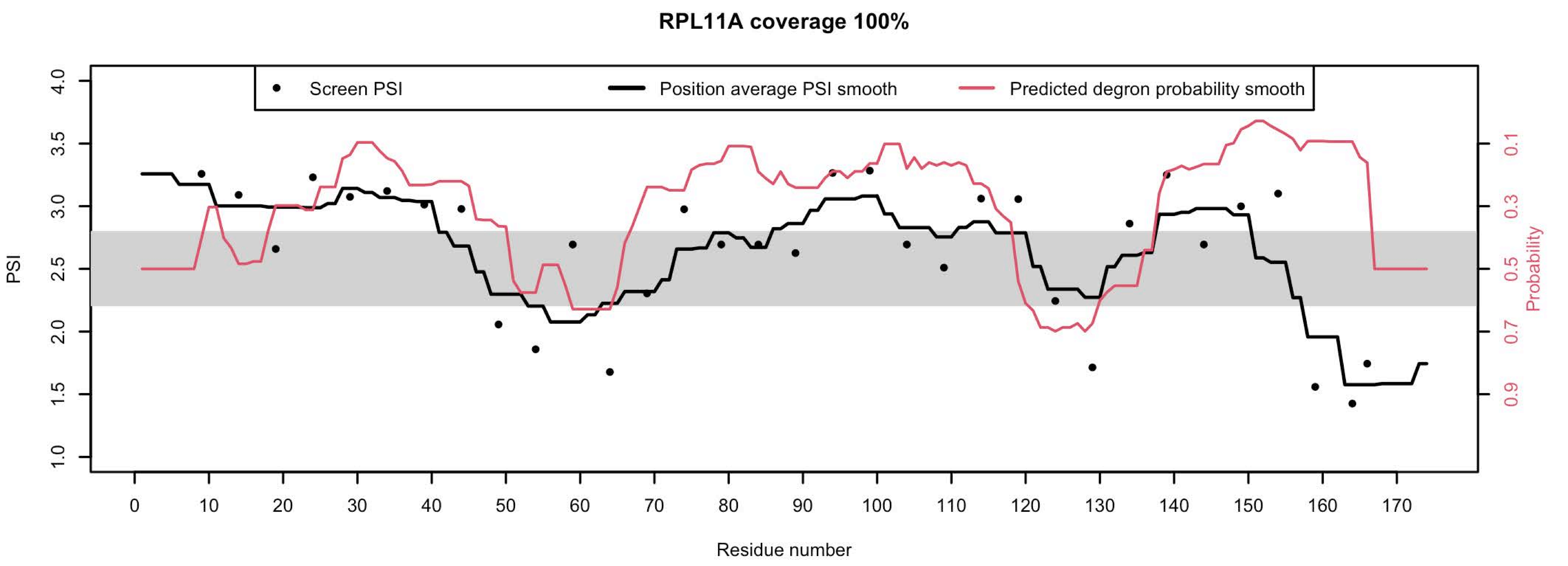

RPL11A coverage 100%
Screen PSI
Position average PSI smooth
Predicted degron probability smooth
PSI
1.0
1.5
2.0
2.5
3.0
3.5
4.0
Probability
0.1
0.3
0.5
0.7
0.9
0
10
20
30
40
50
60
70
80
90
100
110
120
130
140
150
160
170
Residue number

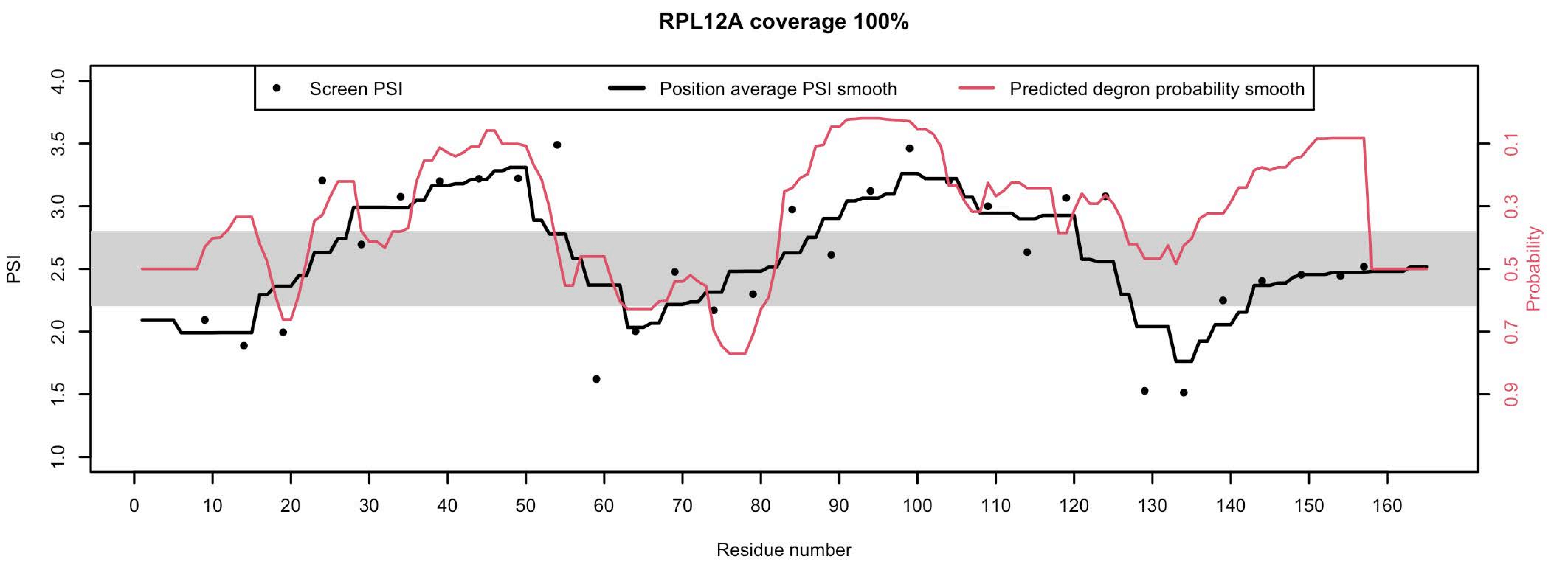
RPL12A coverage 100%
Screen PSI
Position average PSI smooth
Predicted degron probability smooth
PSI
Probability
Residue number
1.0
1.5
2.0
2.5
3.0
3.5
4.0
0.1
0.3
0.5
0.7
0.9
0
10
20
30
40
50
60
70
80
90
100
110
120
130
140
150
160

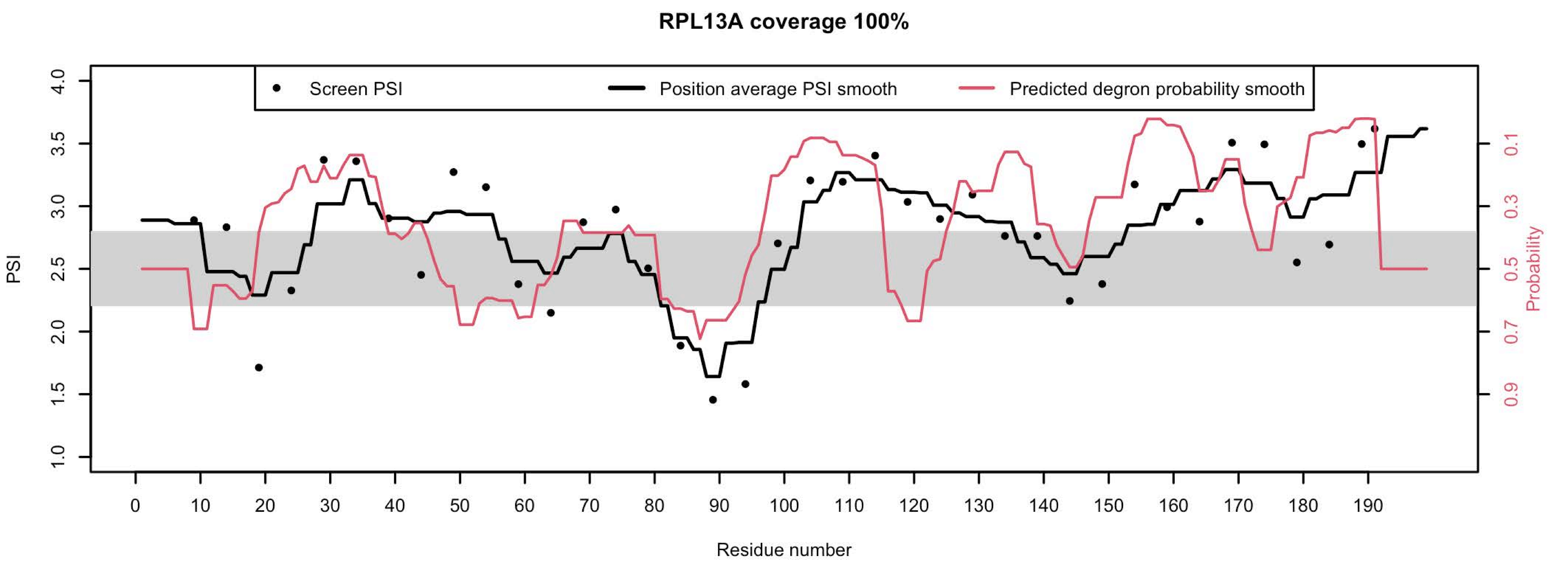
RPL13A coverage 100%
Screen PSI
Position average PSI smooth
Predicted degron probability smooth
PSI
Probability
Residue number
1.0
1.5
2.0
2.5
3.0
3.5
4.0
0.1
0.3
0.5
0.7
0.9
0
10
20
30
40
50
60
70
80
90
100
110
120
130
140
150
160
170
180
190

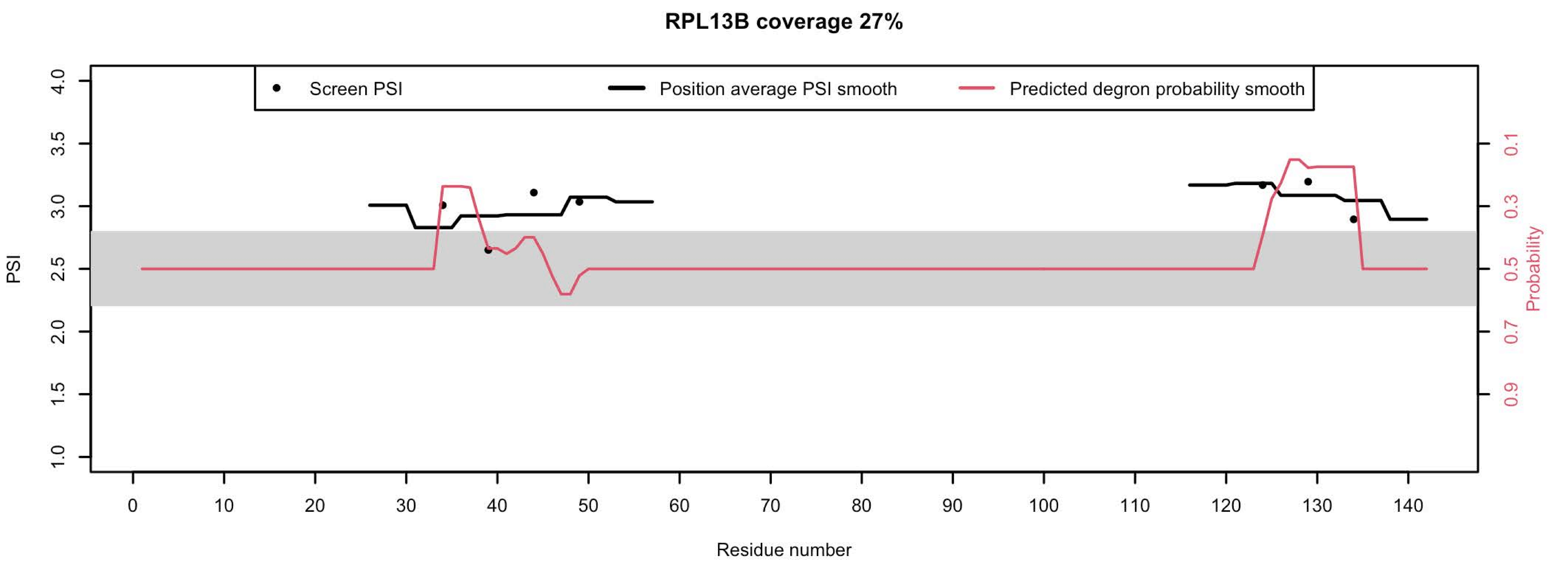

RPL13B coverage 27%
Screen PSI
Position average PSI smooth
Predicted degron probability smooth
PSI
1.0
1.5
2.0
2.5
3.0
3.5
4.0
Probability
0.1
0.3
0.5
0.7
0.9
0
10
20
30
40
50
60
70
80
90
100
110
120
130
140
Residue number

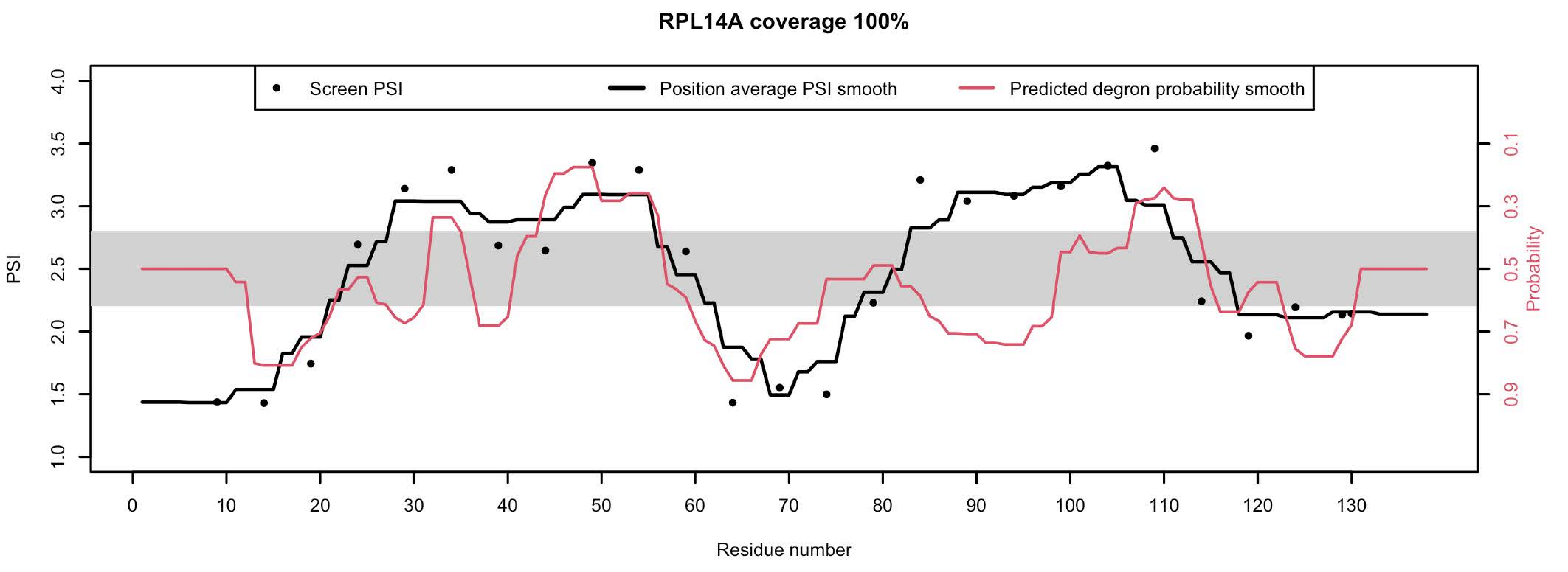
RPL14A coverage 100%
Screen PSI
Position average PSI smooth
Predicted degron probability smooth
PSI
Probability
Residue number
1.0
1.5
2.0
2.5
3.0
3.5
4.0
0.1
0.3
0.5
0.7
0.9
0
10
20
30
40
50
60
70
80
90
100
110
120
130

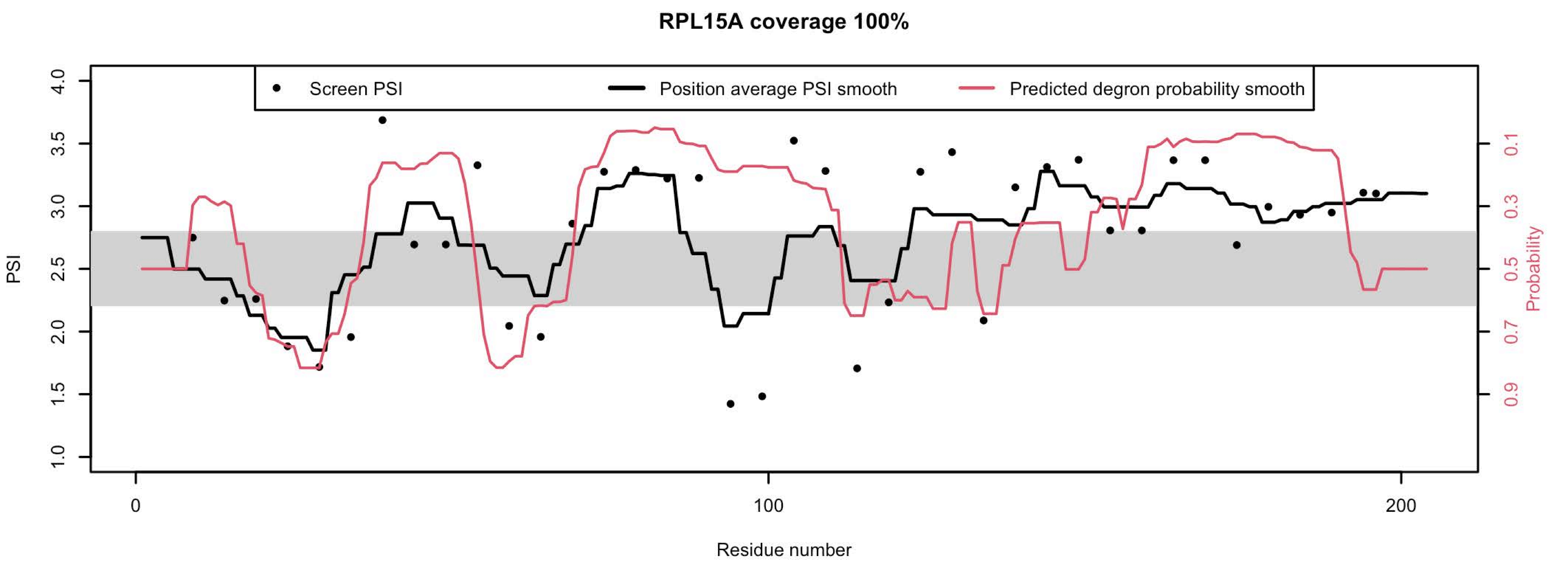

RPL15A coverage 100%
Screen PSI
Position average PSI smooth
Predicted degron probability smooth
PSI
Probability
Residue number
1.0
1.5
2.0
2.5
3.0
3.5
4.0
0.1
0.3
0.5
0.7
0.9
0
100
200

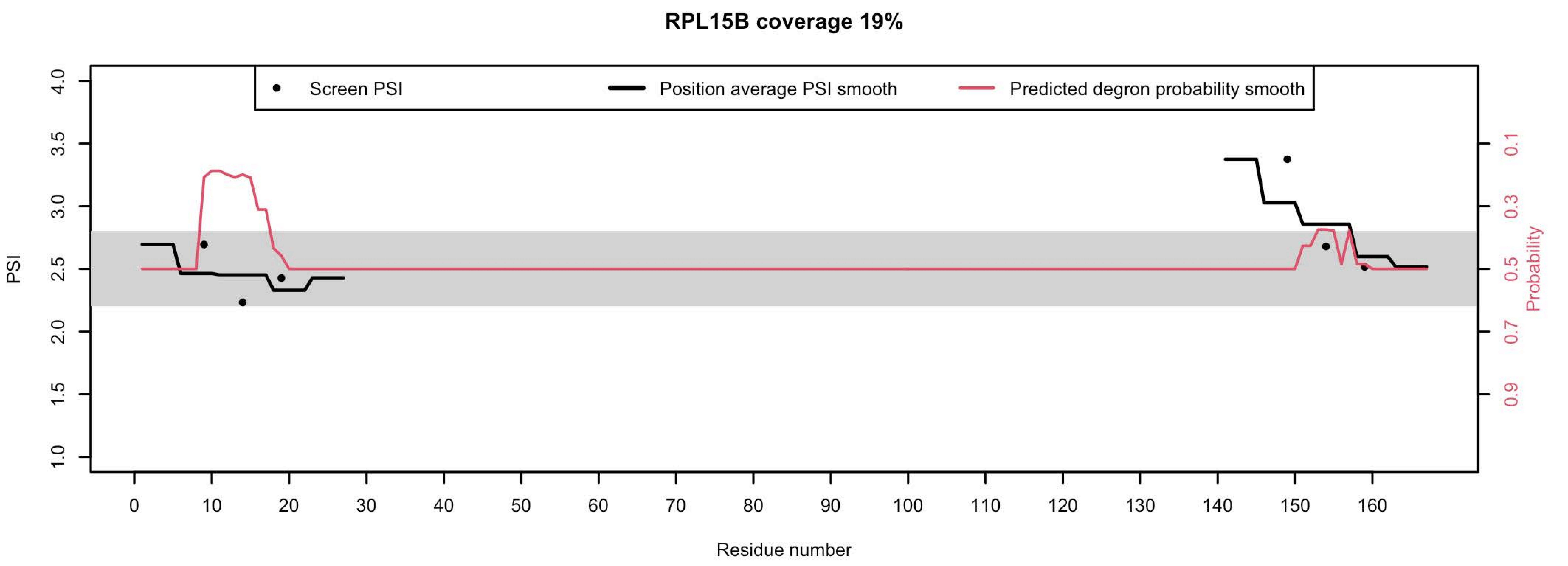

RPL15B coverage 19%
Screen PSI
Position average PSI smooth
Predicted degron probability smooth
PSI
Probability
Residue number

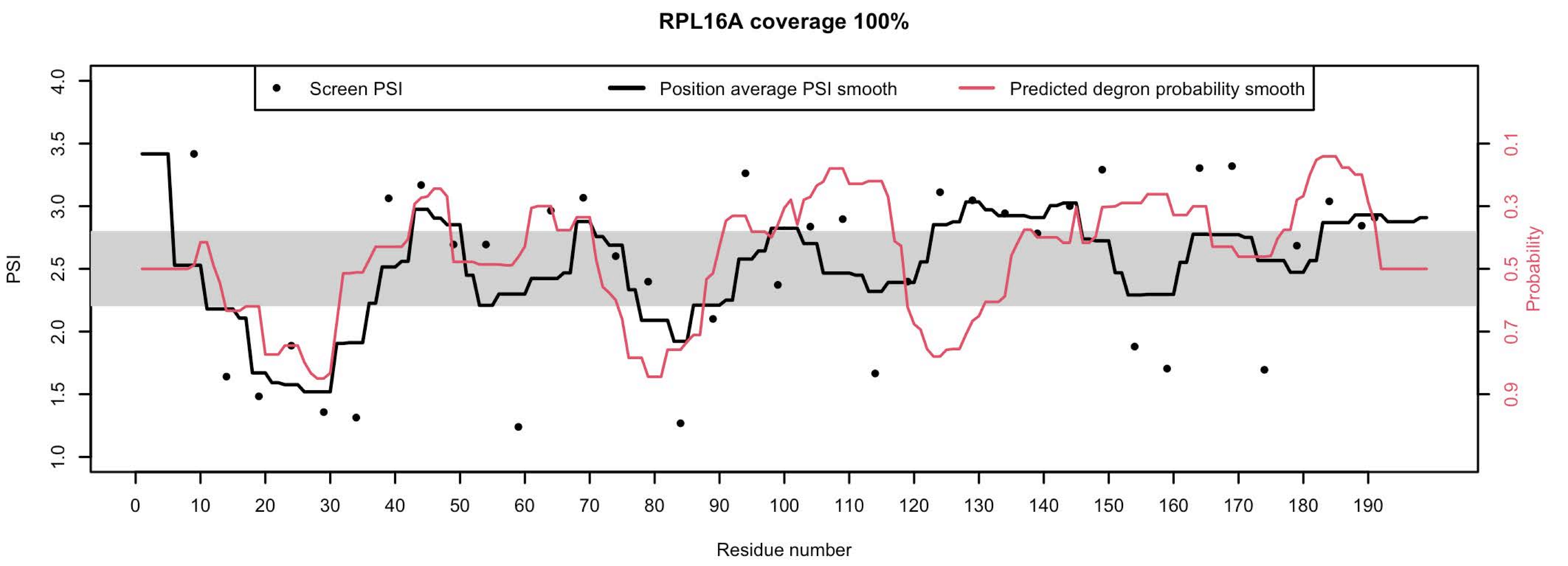

RPL16A coverage 100%
Screen PSI
Position average PSI smooth
Predicted degron probability smooth
PSI
Probability
Residue number
1.0
1.5
2.0
2.5
3.0
3.5
4.0
0.1
0.3
0.5
0.7
0.9
0
10
20
30
40
50
60
70
80
90
100
110
120
130
140
150
160
170
180
190

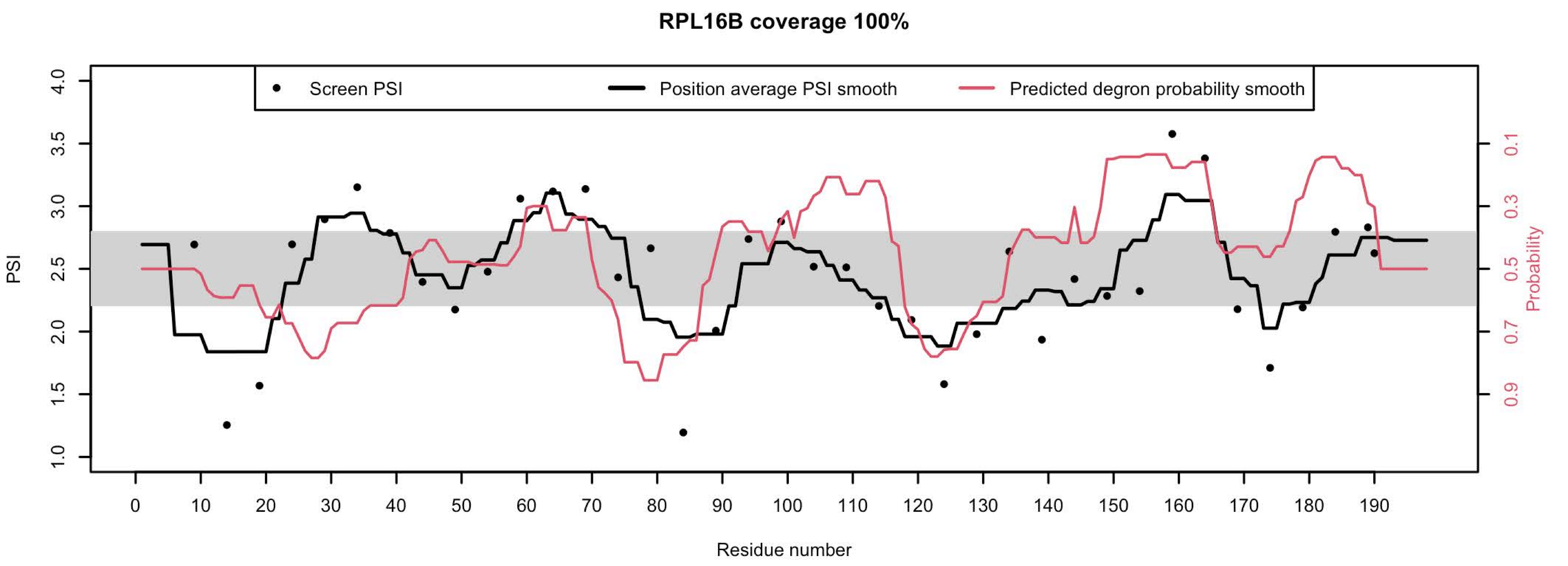
RPL16B coverage 100%
Screen PSI
Position average PSI smooth
Predicted degron probability smooth
PSI
1.0
1.5
2.0
2.5
3.0
3.5
4.0
Probability
0.1
0.3
0.5
0.7
0.9
0 10 20 30 40 50 60 70 80 90 100 110 120 130 140 150 160 170 180 190
Residue number

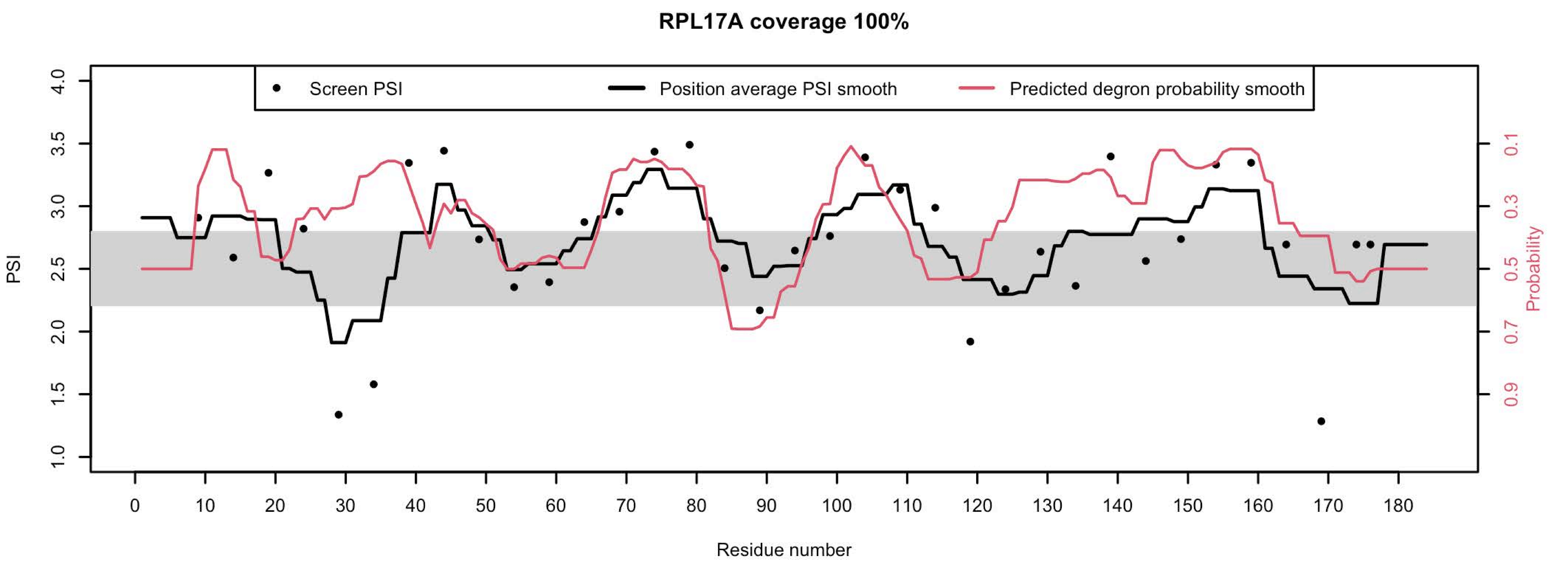
RPL17A coverage 100%
Screen PSI
Position average PSI smooth
Predicted degron probability smooth
PSI
Probability
Residue number
1.0
1.5
2.0
2.5
3.0
3.5
4.0
0.1
0.3
0.5
0.7
0.9
0
10
20
30
40
50
60
70
80
90
100
110
120
130
140
150
160
170
180

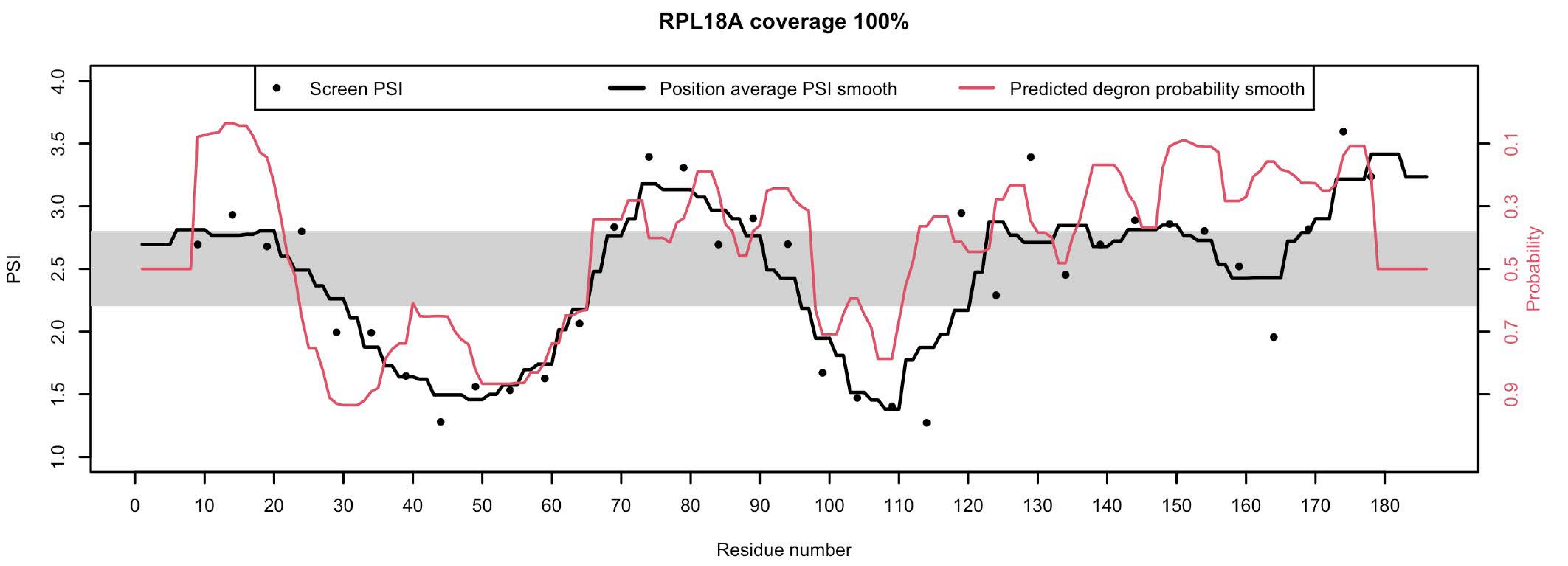

RPL18A coverage 100%
Screen PSI
Position average PSI smooth
Predicted degron probability smooth
PSI
1.0
1.5
2.0
2.5
3.0
3.5
4.0
Probability
0.1
0.3
0.5
0.7
0.9
Residue number
0
10
20
30
40
50
60
70
80
90
100
110
120
130
140
150
160
170
180

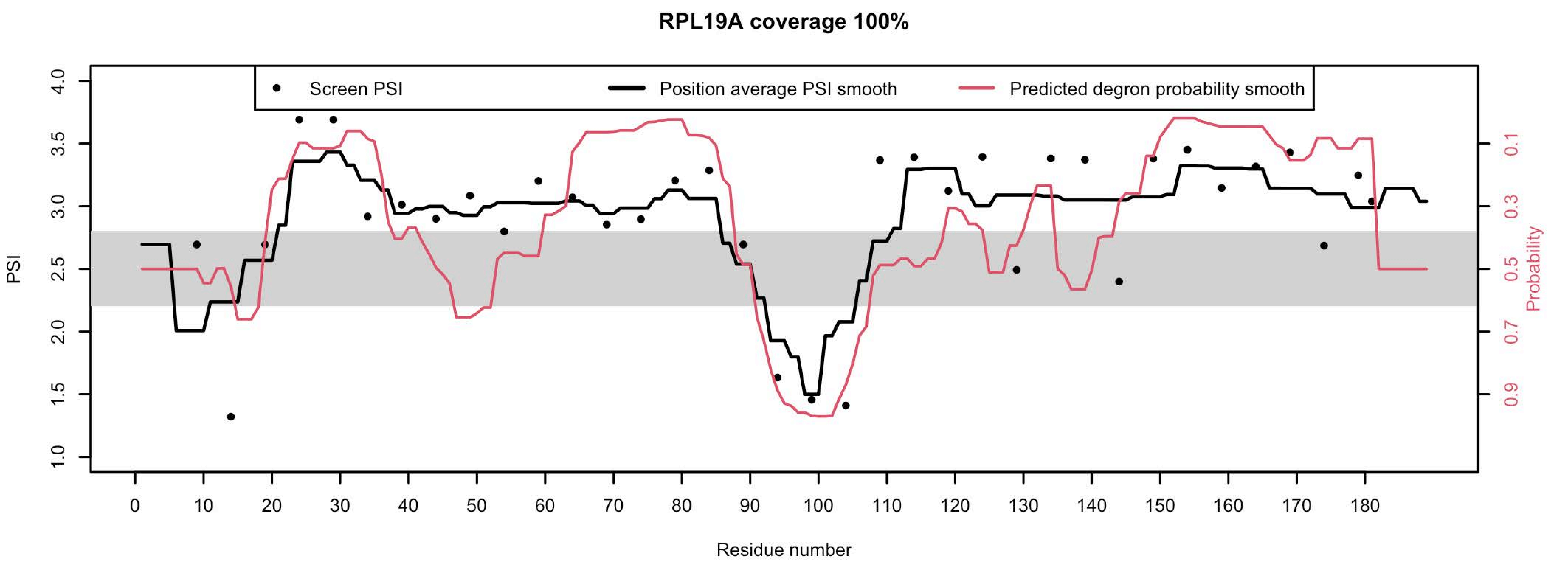
RPL19A coverage 100%
Screen PSI
Position average PSI smooth
Predicted degron probability smooth
PSI
Probability
Residue number
1.0
1.5
2.0
2.5
3.0
3.5
4.0
0.1
0.3
0.5
0.7
0.9
0
10
20
30
40
50
60
70
80
90
100
110
120
130
140
150
160
170
180

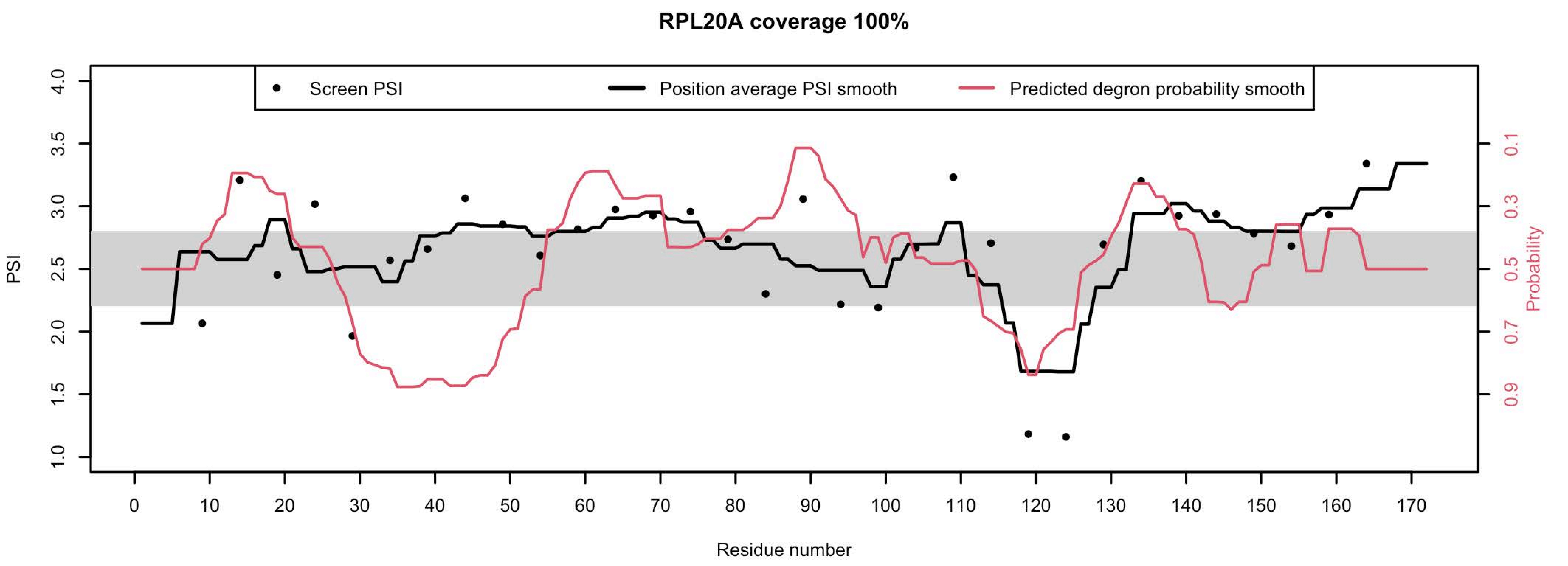
RPL20A coverage 100%
Screen PSI
Position average PSI smooth
Predicted degron probability smooth
PSI
1.0
1.5
2.0
2.5
3.0
3.5
4.0
Probability
0.1
0.3
0.5
0.7
0.9
Residue number
0
10
20
30
40
50
60
70
80
90
100
110
120
130
140
150
160
170

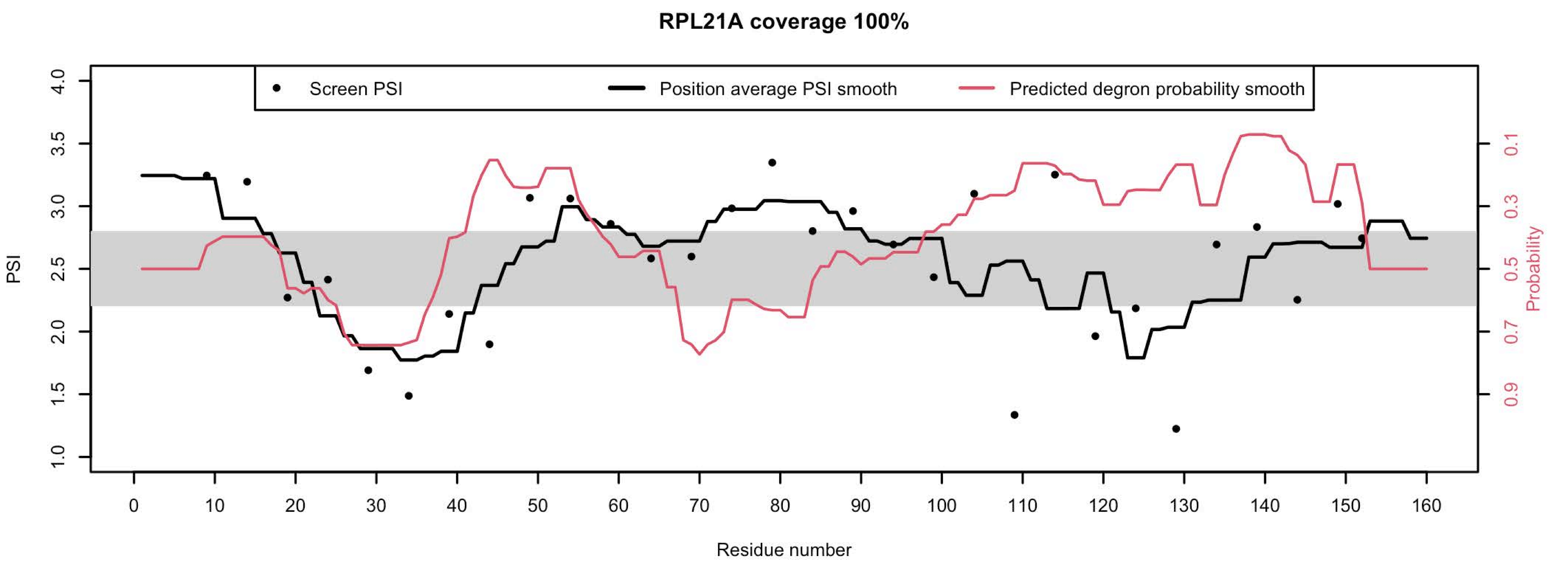
RPL21A coverage 100%
Screen PSI
Position average PSI smooth
Predicted degron probability smooth
PSI
Probability
Residue number
1.0
1.5
2.0
2.5
3.0
3.5
4.0
0.1
0.3
0.5
0.7
0.9
0
10
20
30
40
50
60
70
80
90
100
110
120
130
140
150
160

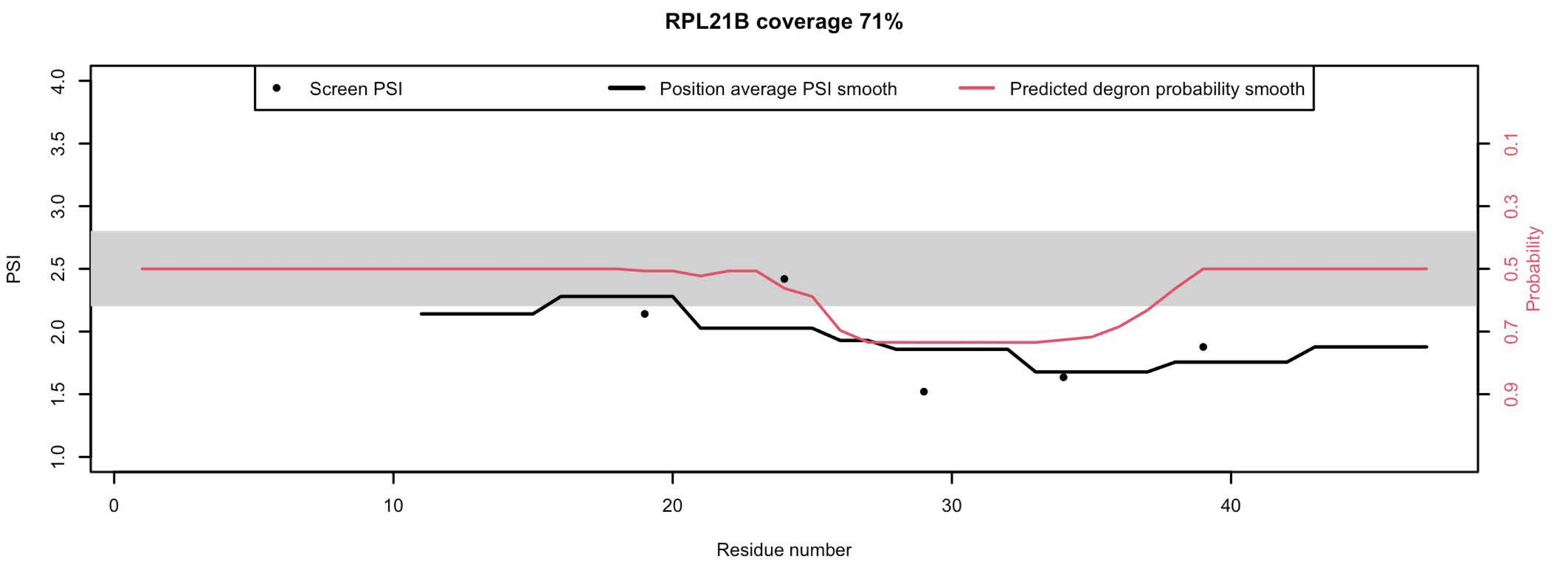

RPL21B coverage 71%
Screen PSI
Position average PSI smooth
Predicted degron probability smooth
PSI
1.0
1.5
2.0
2.5
3.0
3.5
4.0
Probability
0.1
0.3
0.5
0.7
0.9
0
10
20
30
40
Residue number

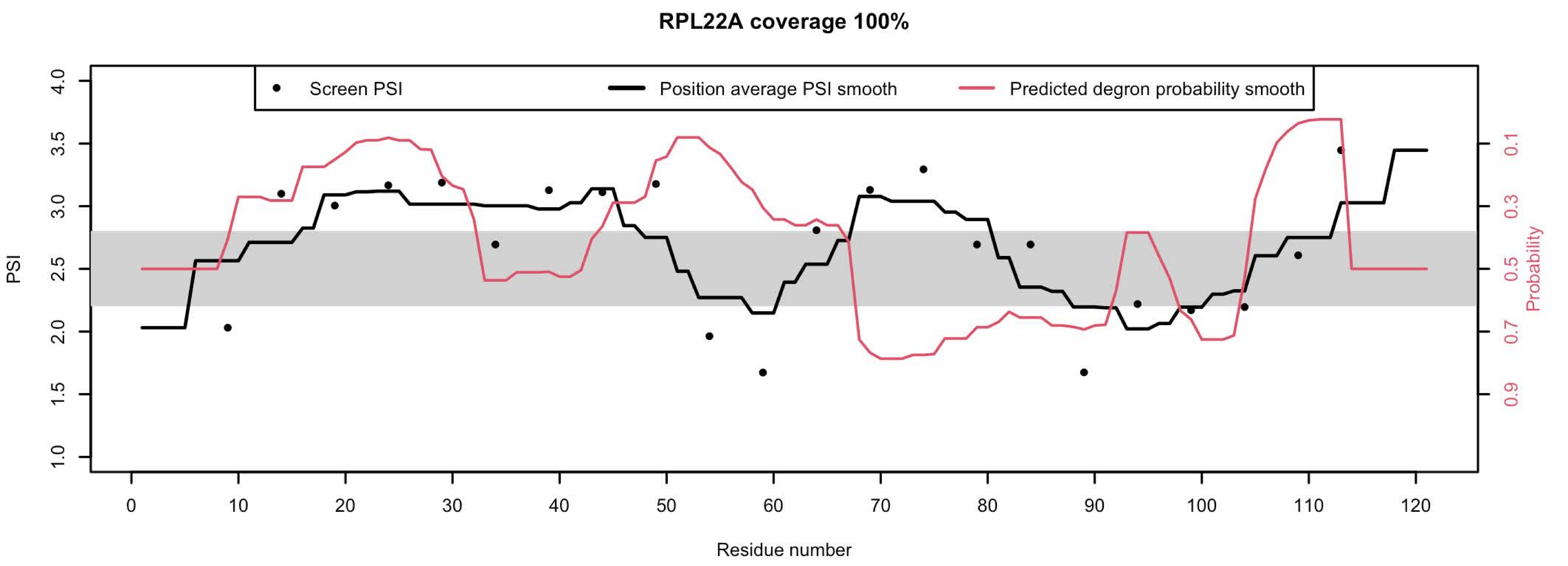

RPL22A coverage 100%
Screen PSI
Position average PSI smooth
Predicted degron probability smooth
PSI
Probability
Residue number
1.0
1.5
2.0
2.5
3.0
3.5
4.0
0.1
0.3
0.5
0.7
0.9
0
10
20
30
40
50
60
70
80
90
100
110
120

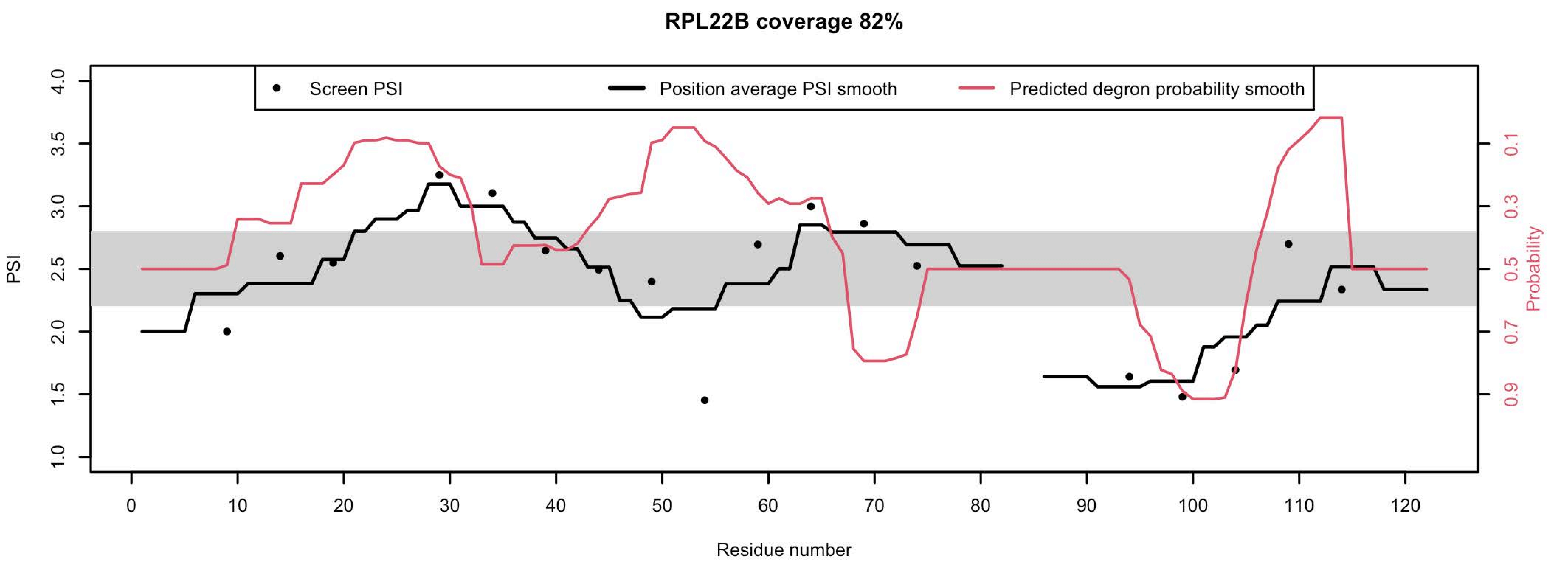

RPL22B coverage 82%
Screen PSI
Position average PSI smooth
Predicted degron probability smooth
PSI
Probability
Residue number
1.0
1.5
2.0
2.5
3.0
3.5
4.0
0.1
0.3
0.5
0.7
0.9
0
10
20
30
40
50
60
70
80
90
100
110
120

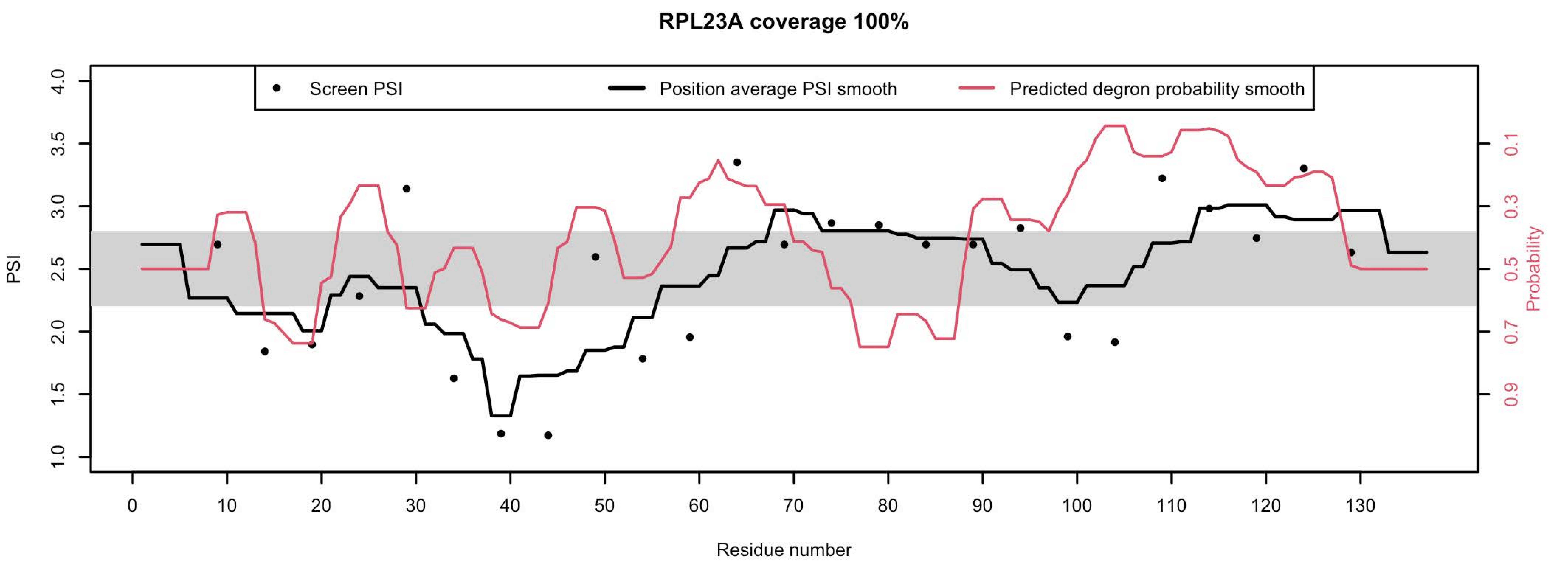

RPL23A coverage 100%
Screen PSI
Position average PSI smooth
Predicted degron probability smooth
PSI
1.0
1.5
2.0
2.5
3.0
3.5
4.0
Probability
0.1
0.3
0.5
0.7
0.9
0
10
20
30
40
50
60
70
80
90
100
110
120
130
Residue number

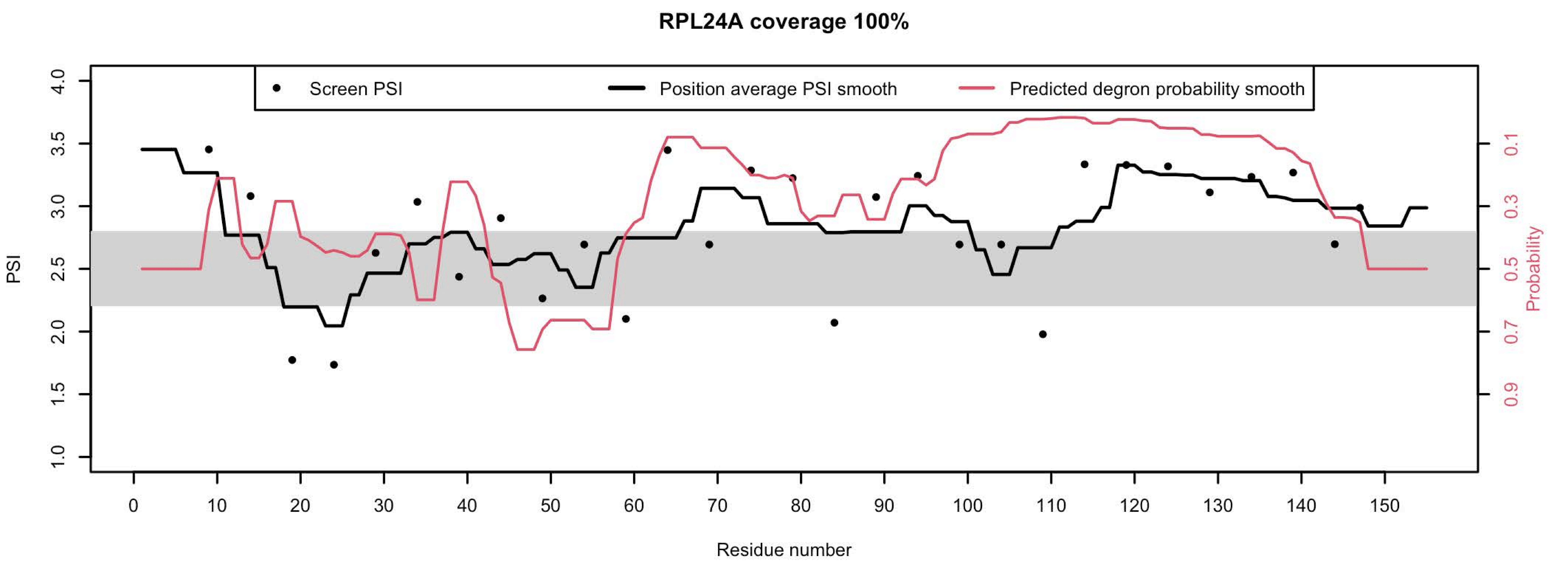
RPL24A coverage 100%
Screen PSI
Position average PSI smooth
Predicted degron probability smooth
PSI
1.0
1.5
2.0
2.5
3.0
3.5
4.0
Probability
0.1
0.3
0.5
0.7
0.9
Residue number
0
10
20
30
40
50
60
70
80
90
100
110
120
130
140
150

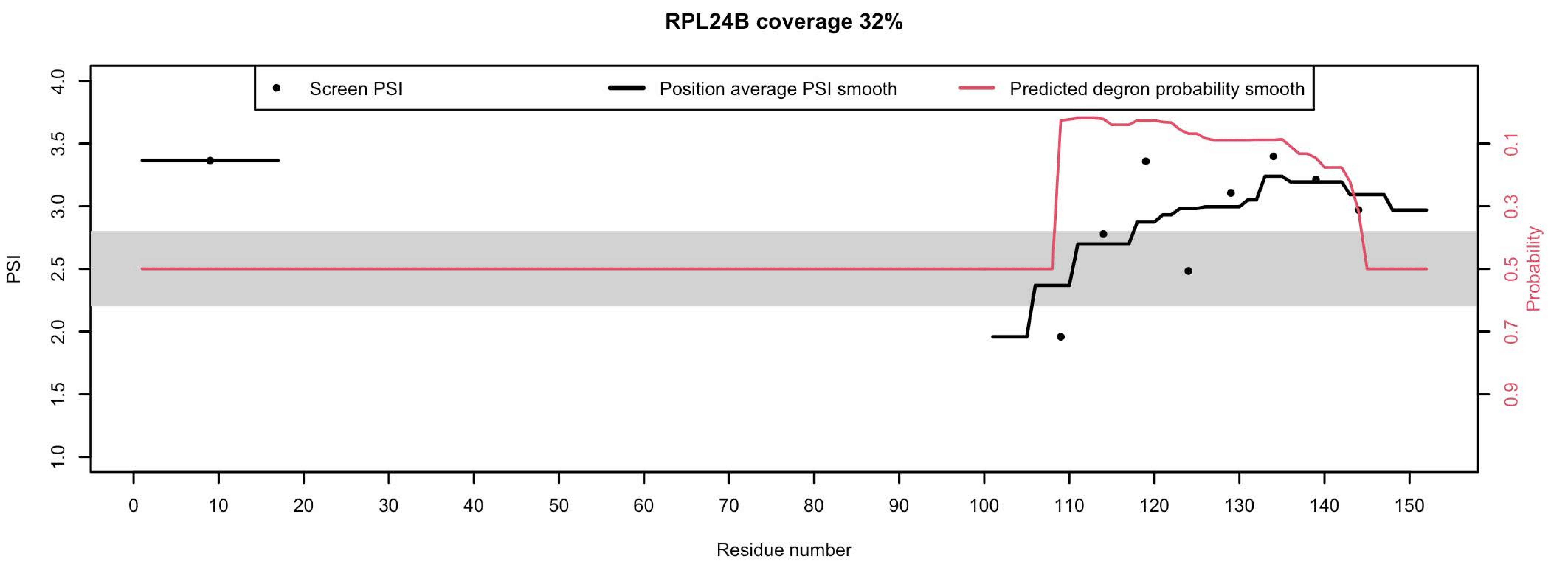
RPL24B coverage 32%
Screen PSI
Position average PSI smooth
Predicted degron probability smooth
PSI
1.0
1.5
2.0
2.5
3.0
3.5
4.0
Probability
0.1
0.3
0.5
0.7
0.9
Residue number
0
10
20
30
40
50
60
70
80
90
100
110
120
130
140
150

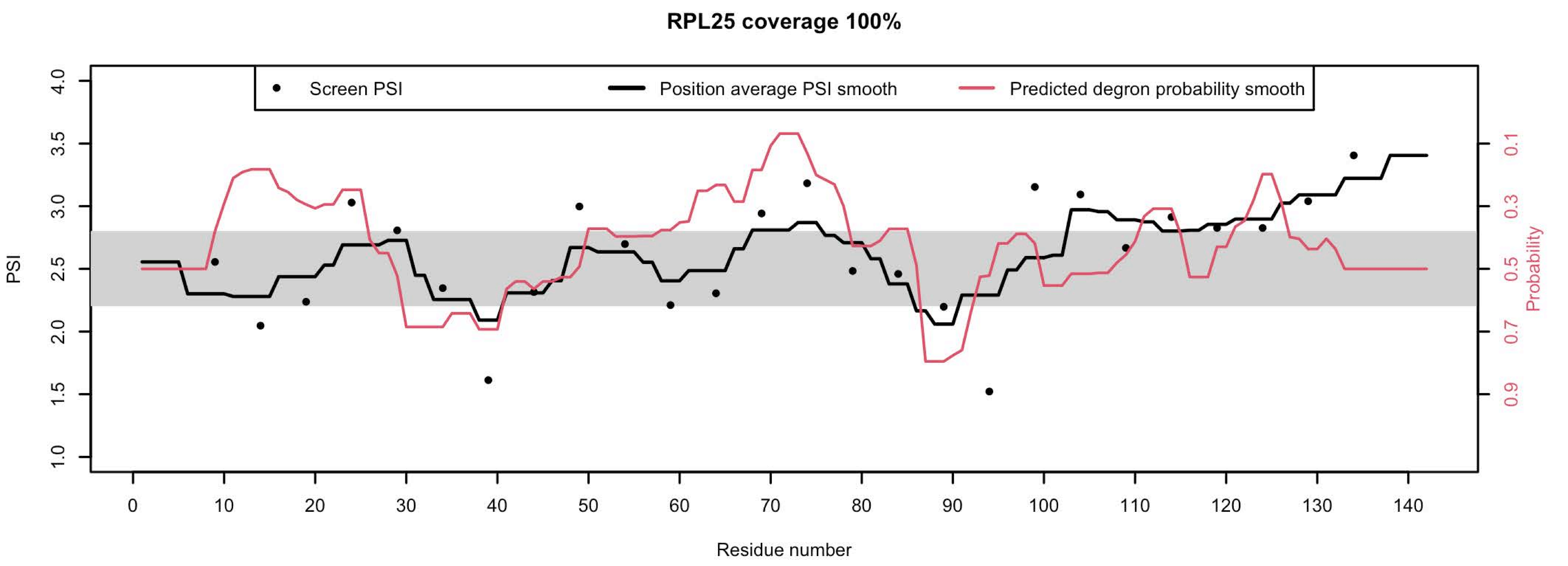

RPL25 coverage 100%
Screen PSI
Position average PSI smooth
Predicted degron probability smooth
PSI
Probability
Residue number
1.0
1.5
2.0
2.5
3.0
3.5
4.0
0.1
0.3
0.5
0.7
0.9
0
10
20
30
40
50
60
70
80
90
100
110
120
130
140

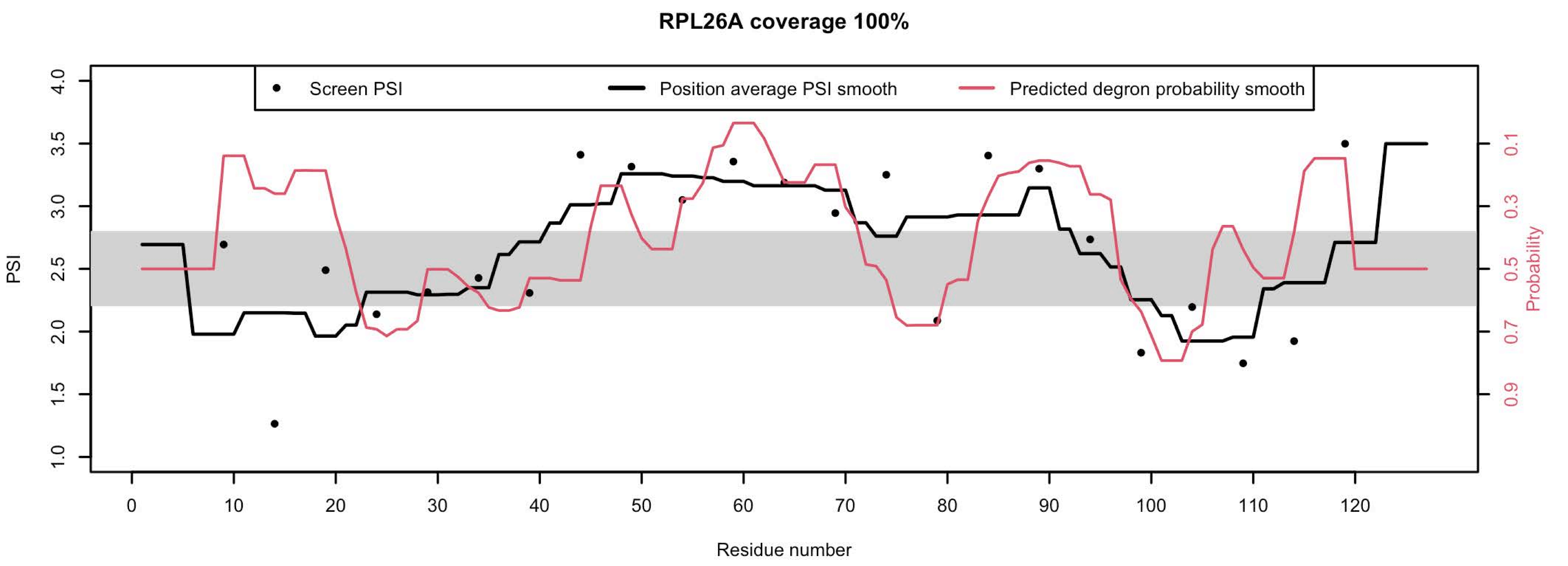

RPL26A coverage 100%
Screen PSI
Position average PSI smooth
Predicted degron probability smooth
PSI
1.0
1.5
2.0
2.5
3.0
3.5
4.0
Probability
0.1
0.3
0.5
0.7
0.9
0
10
20
30
40
50
60
70
80
90
100
110
120
Residue number

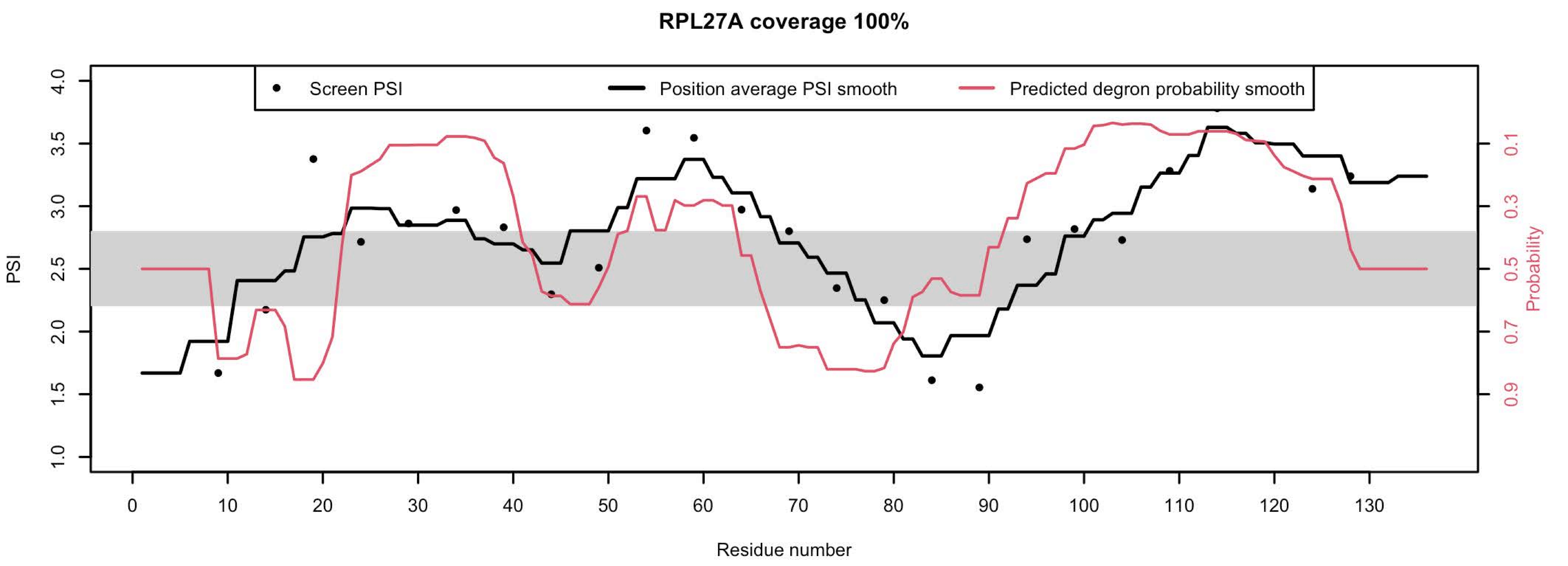

RPL27A coverage 100%
Screen PSI
Position average PSI smooth
Predicted degron probability smooth
PSI
1.0
1.5
2.0
2.5
3.0
3.5
4.0
Probability
0.1
0.3
0.5
0.7
0.9
0
10
20
30
40
50
60
70
80
90
100
110
120
130
Residue number

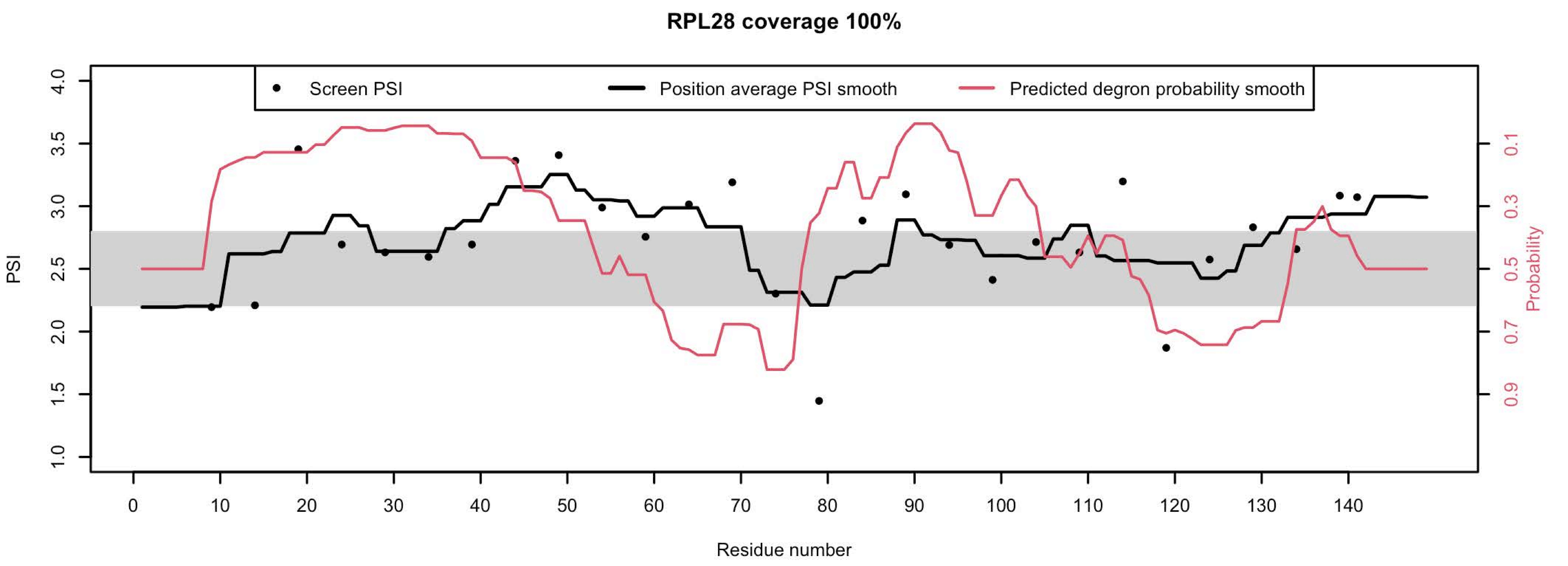

RPL28 coverage 100%
Screen PSI
Position average PSI smooth
Predicted degron probability smooth
PSI
1.0
1.5
2.0
2.5
3.0
3.5
4.0
Probability
0.1
0.3
0.5
0.7
0.9
0
10
20
30
40
50
60
70
80
90
100
110
120
130
140
Residue number

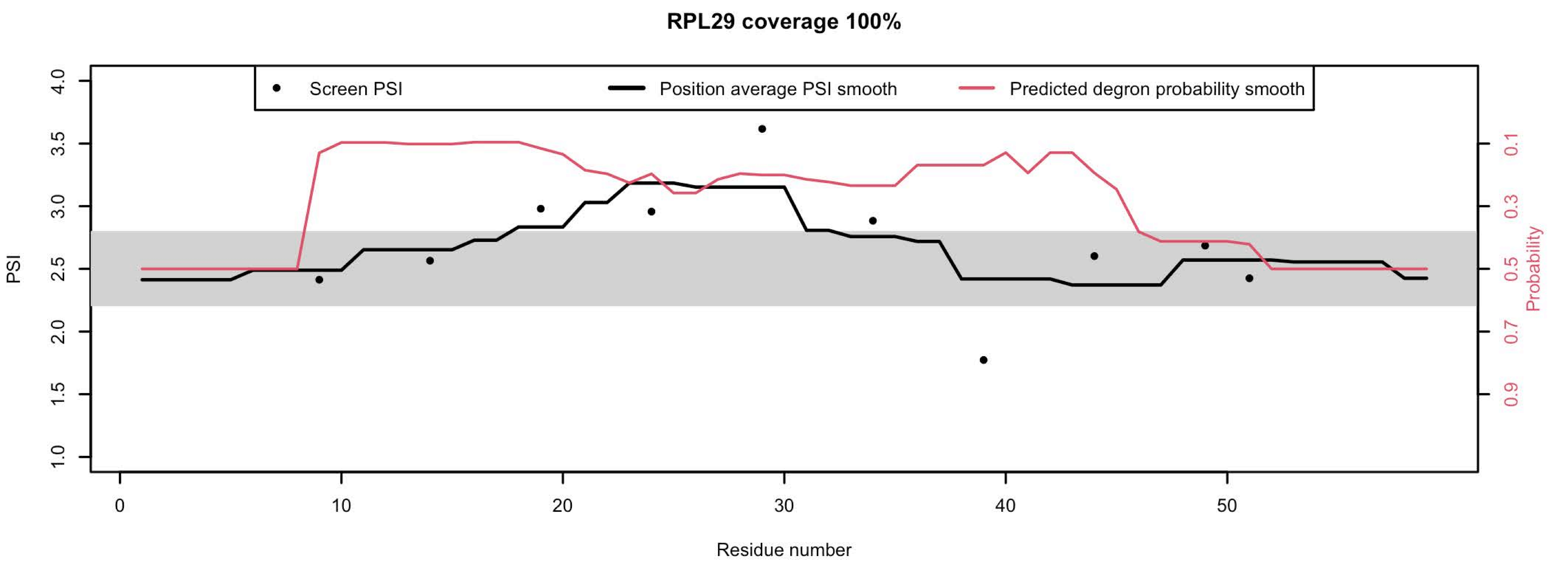
RPL29 coverage 100%
Screen PSI
Position average PSI smooth
Predicted degron probability smooth
PSI
Probability
Residue number
1.0
1.5
2.0
2.5
3.0
3.5
4.0
0.1
0.3
0.5
0.7
0.9
0
10
20
30
40
50

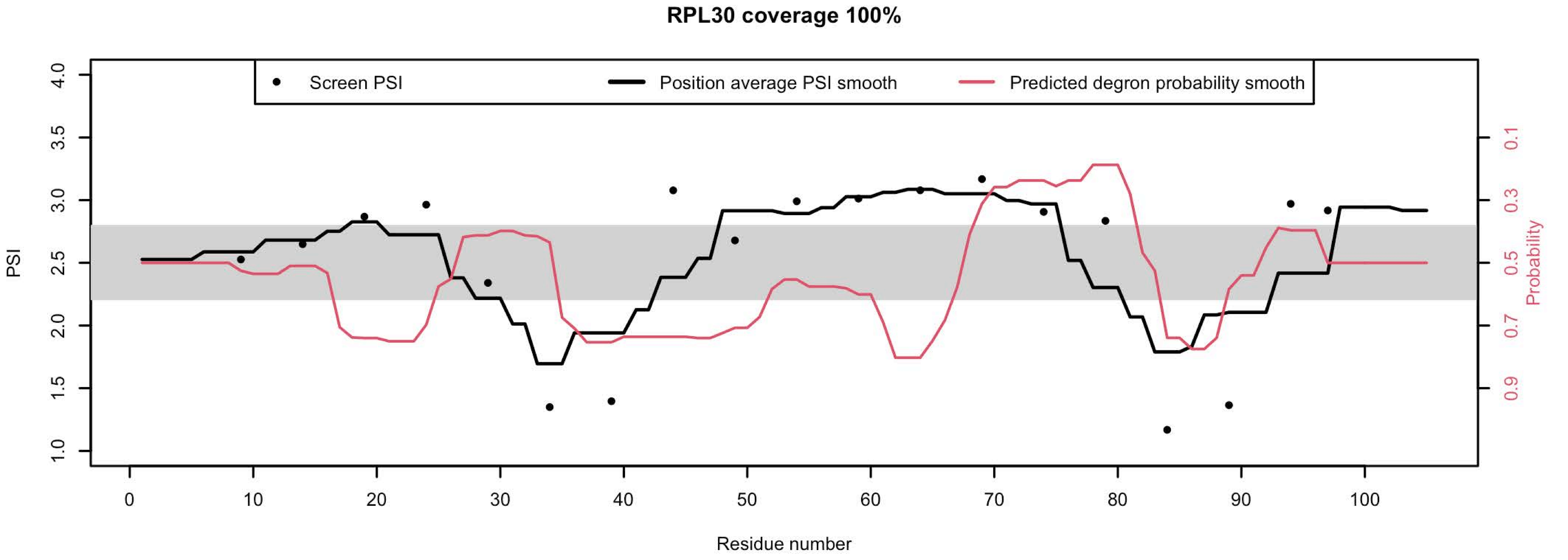

RPL30 coverage 100%
Screen PSI
Position average PSI smooth
Predicted degron probability smooth
PSI
1.0
1.5
2.0
2.5
3.0
3.5
4.0
Probability
0.9
0.7
0.5
0.3
0.1
0
10
20
30
40
50
60
70
80
90
100
Residue number

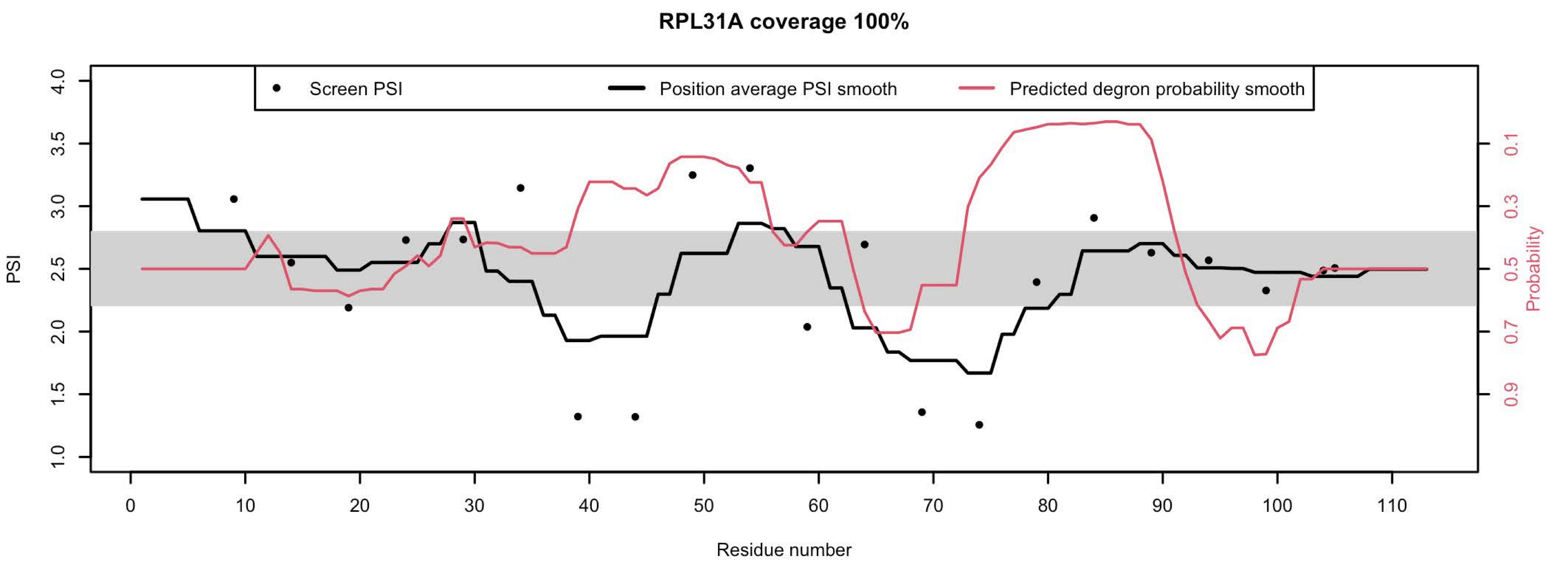
RPL31A coverage 100%
Screen PSI
Position average PSI smooth
Predicted degron probability smooth
PSI
Probability
Residue number
1.0
1.5
2.0
2.5
3.0
3.5
4.0
0.1
0.3
0.5
0.7
0.9
0
10
20
30
40
50
60
70
80
90
100
110

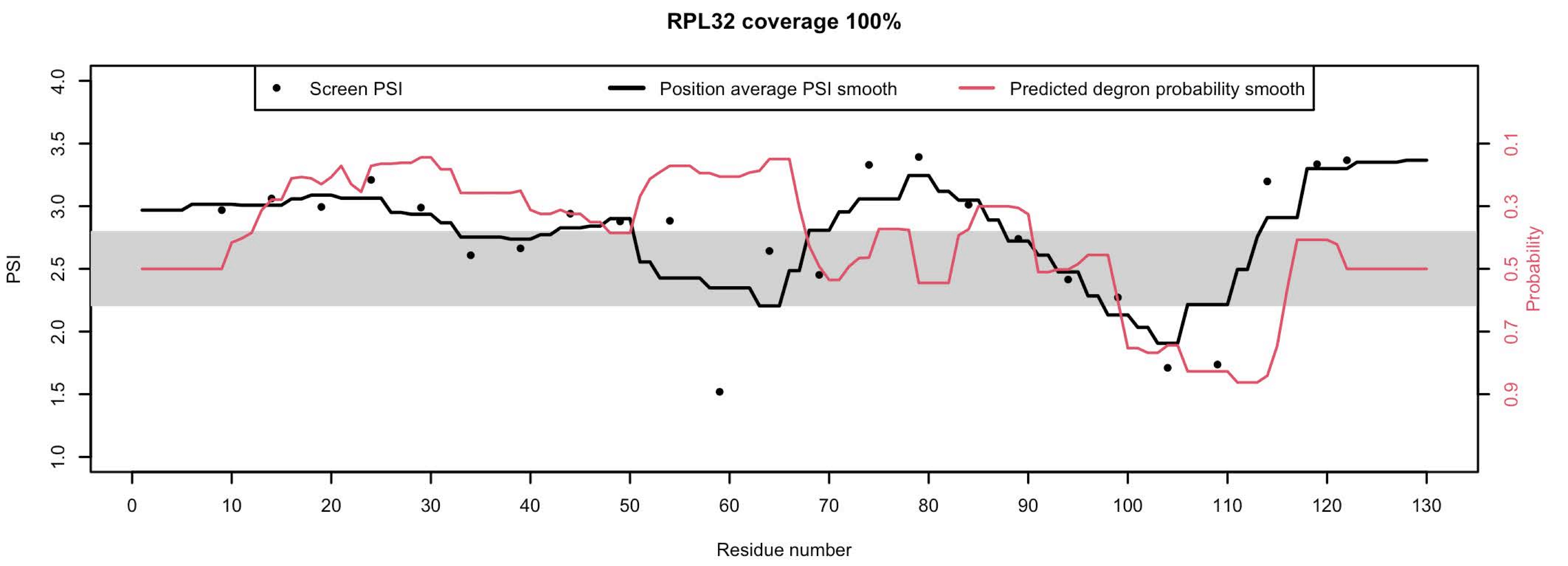
RPL32 coverage 100%
Screen PSI
Position average PSI smooth
Predicted degron probability smooth
PSI
1.0
1.5
2.0
2.5
3.0
3.5
4.0
Probability
0.1
0.3
0.5
0.7
0.9
0
10
20
30
40
50
60
70
80
90
100
110
120
130
Residue number

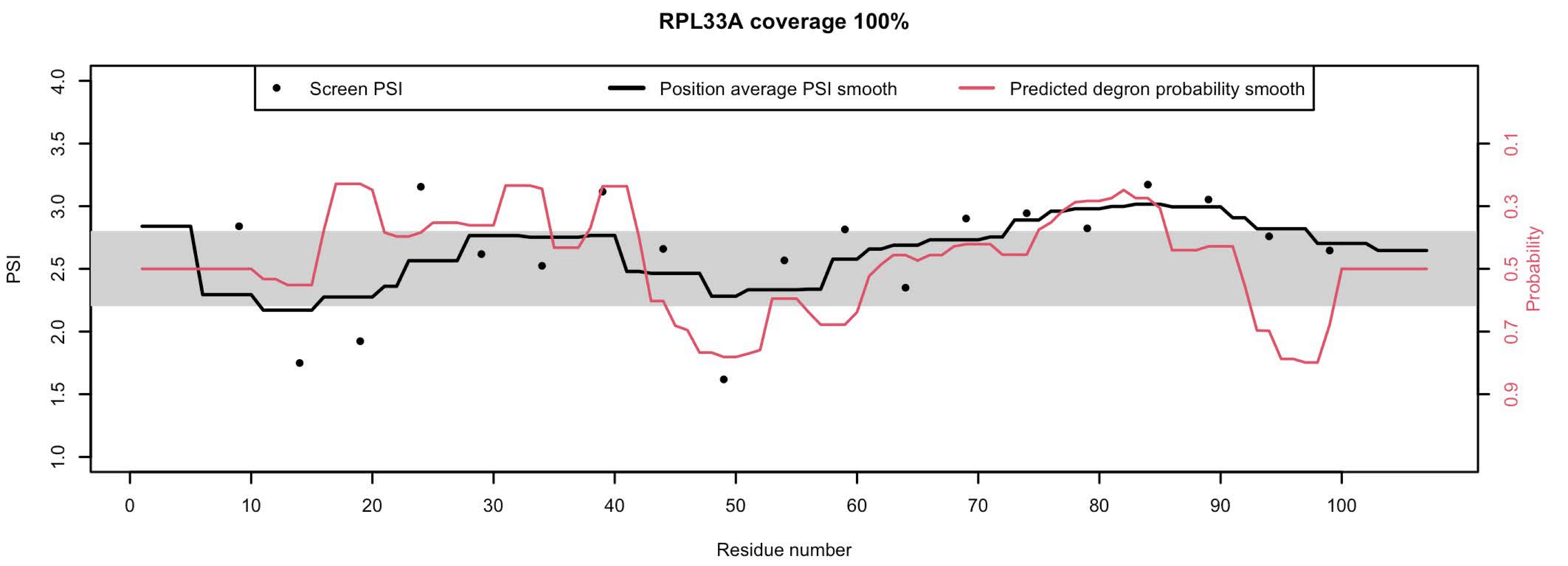

RPL33A coverage 100%
Screen PSI
Position average PSI smooth
Predicted degron probability smooth
PSI
4.0
3.5
3.0
2.5
2.0
1.5
1.0
Probability
0.1
0.3
0.5
0.7
0.9
0
10
20
30
40
50
60
70
80
90
100
Residue number

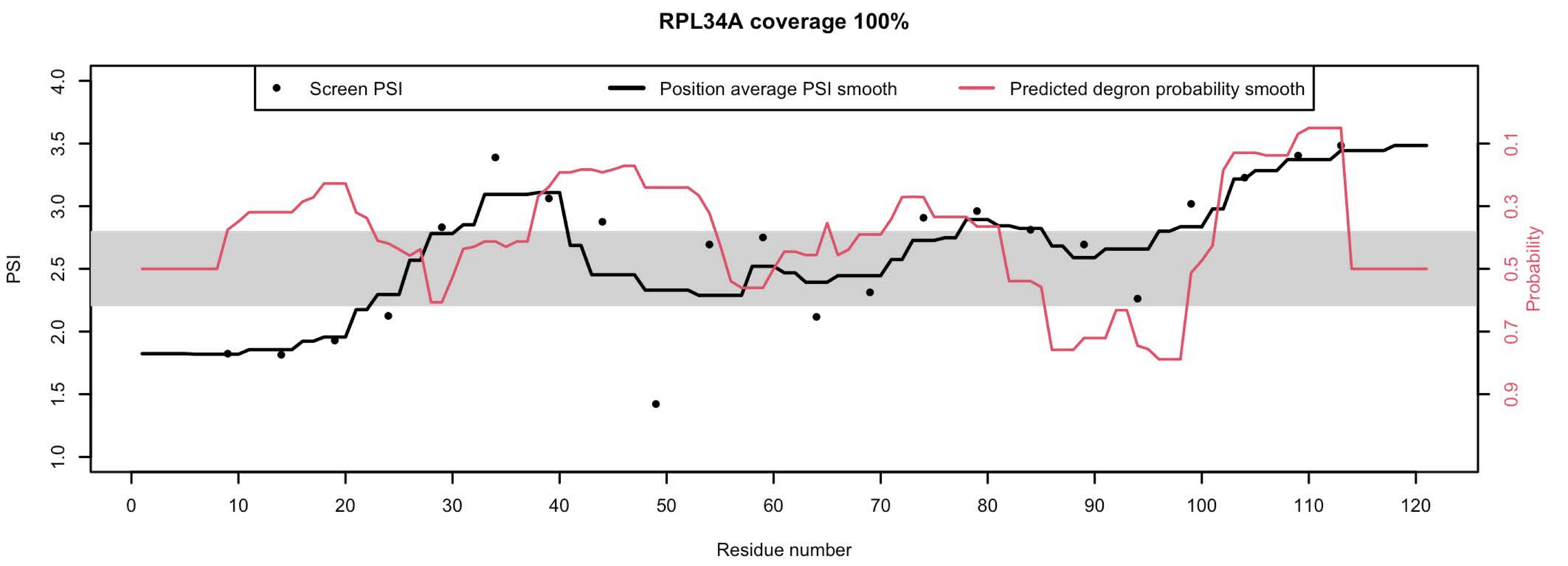

RPL34A coverage 100%
Screen PSI
Position average PSI smooth
Predicted degron probability smooth
PSI
Probability
Residue number
1.0
1.5
2.0
2.5
3.0
3.5
4.0
0.1
0.3
0.5
0.7
0.9
0
10
20
30
40
50
60
70
80
90
100
110
120

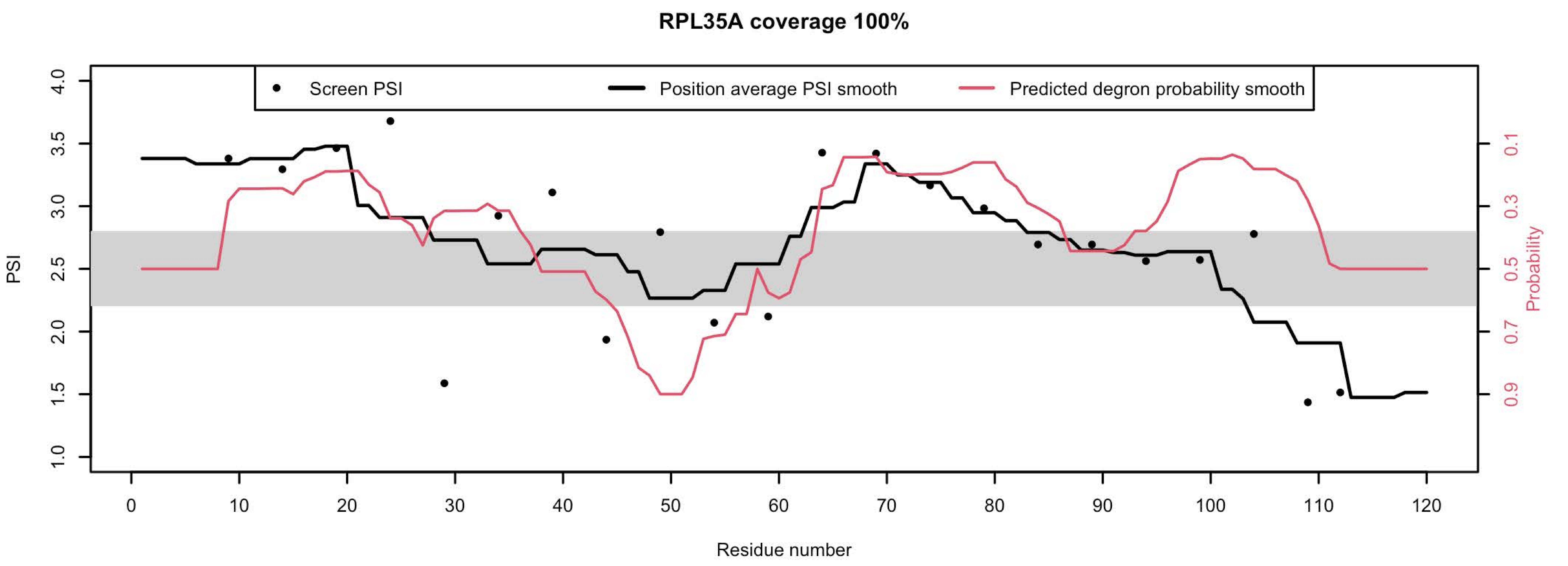

RPL35A coverage 100%
Screen PSI
Position average PSI smooth
Predicted degron probability smooth
PSI
1.0
1.5
2.0
2.5
3.0
3.5
4.0
Probability
0.1
0.3
0.5
0.7
0.9
Residue number
0
10
20
30
40
50
60
70
80
90
100
110
120

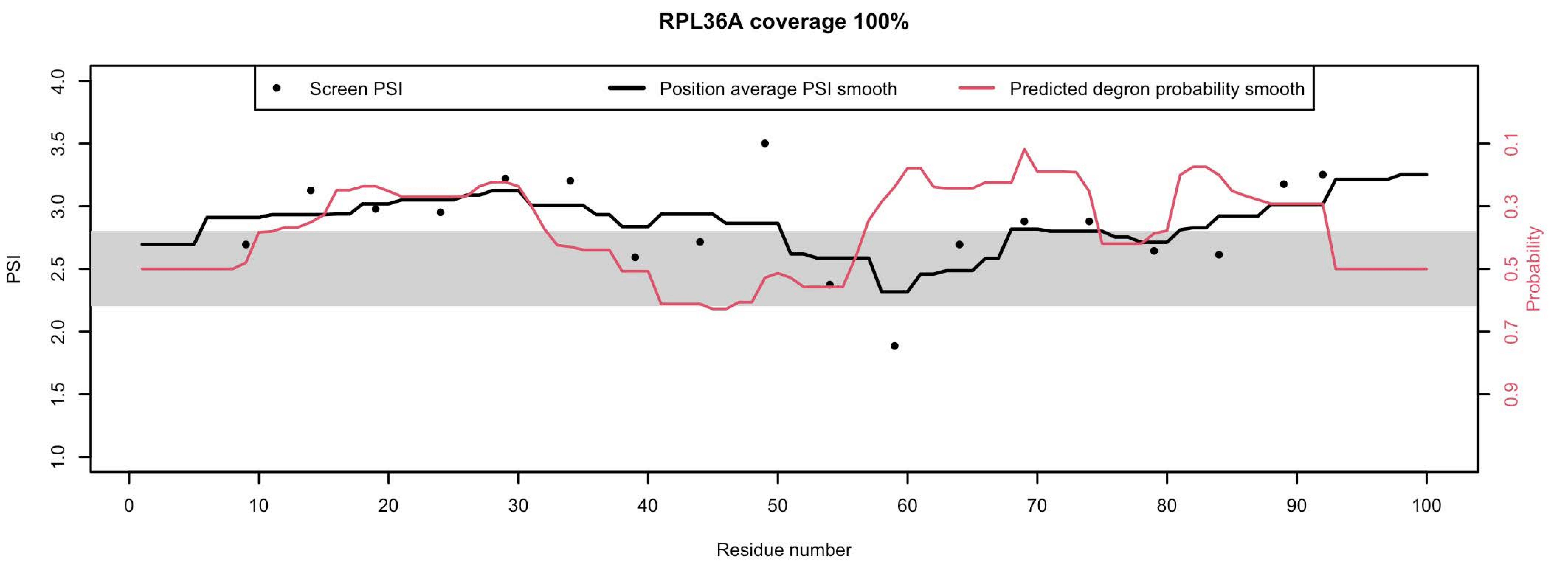

RPL36A coverage 100%
Screen PSI
Position average PSI smooth
Predicted degron probability smooth
PSI
1.0
1.5
2.0
2.5
3.0
3.5
4.0
Probability
0.1
0.3
0.5
0.7
0.9
Residue number
0
10
20
30
40
50
60
70
80
90
100

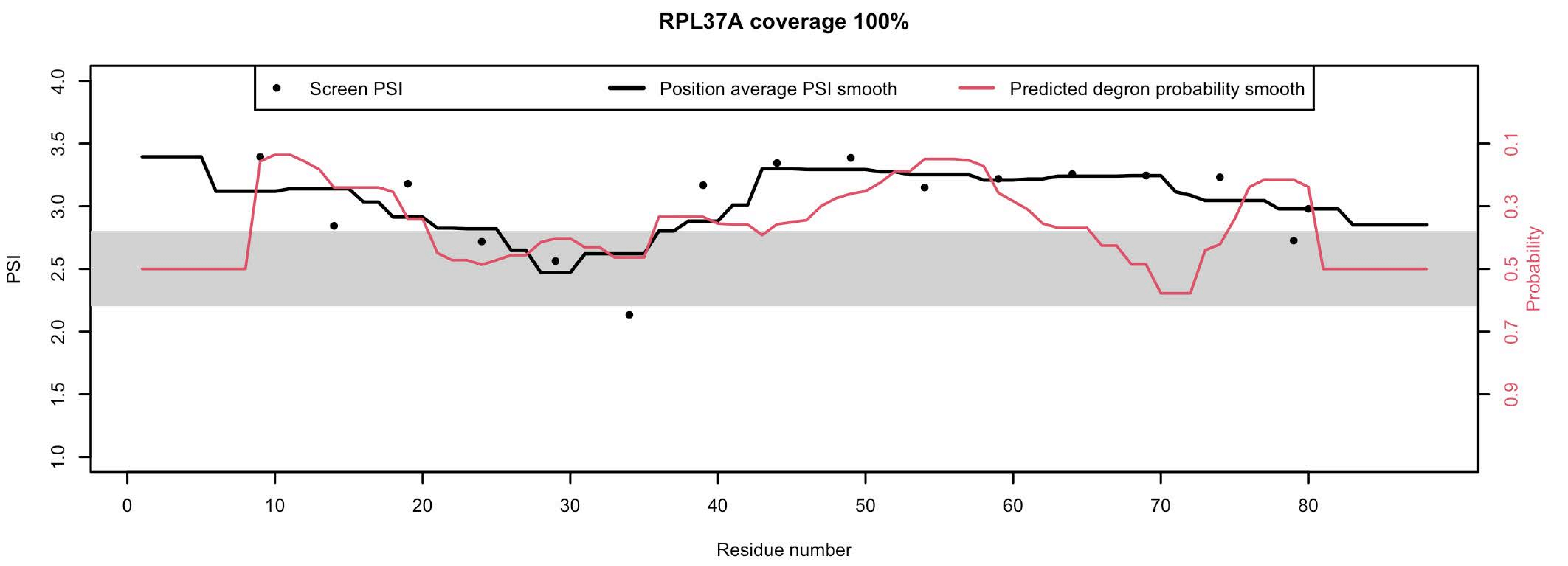
RPL37A coverage 100%
Screen PSI
Position average PSI smooth
Predicted degron probability smooth
PSI
Probability
Residue number
1.0
1.5
2.0
2.5
3.0
3.5
4.0
0.1
0.3
0.5
0.7
0.9
0
10
20
30
40
50
60
70
80

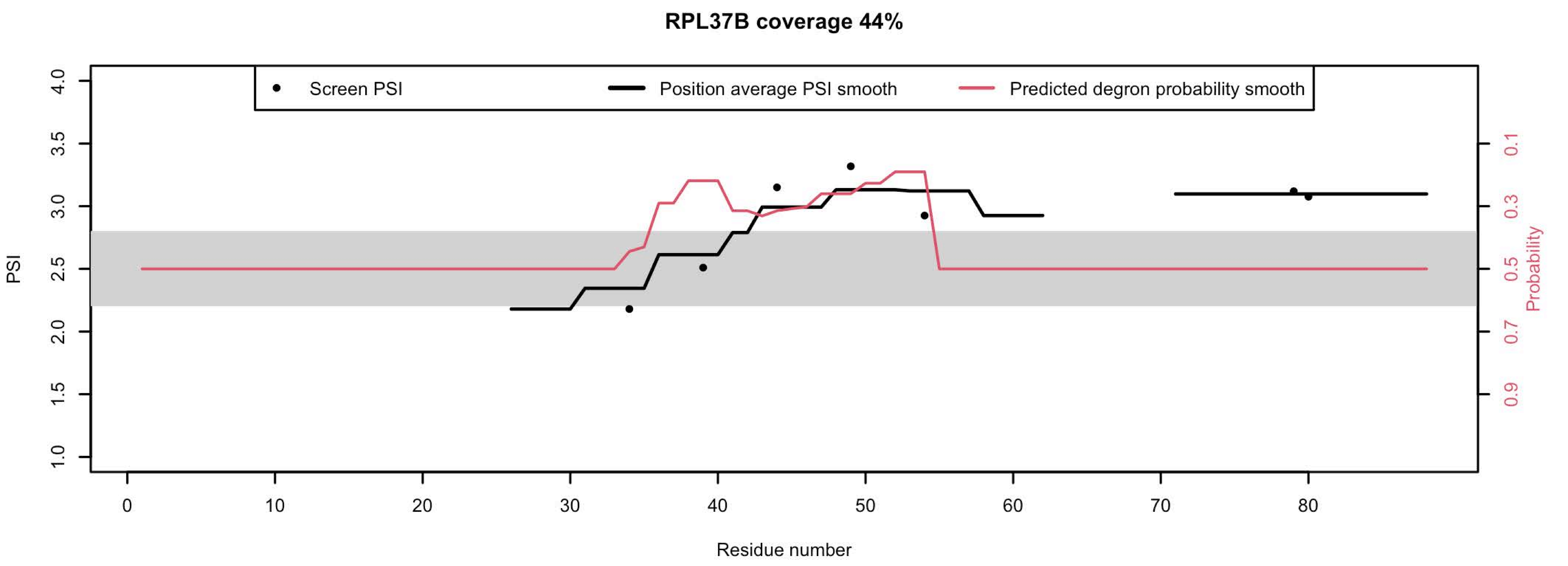
RPL37B coverage 44%
Screen PSI
Position average PSI smooth
Predicted degron probability smooth
PSI
4.0
3.5
3.0
2.5
2.0
1.5
1.0
Probability
0.1
0.3
0.5
0.7
0.9
0
10
20
30
40
50
60
70
80
Residue number

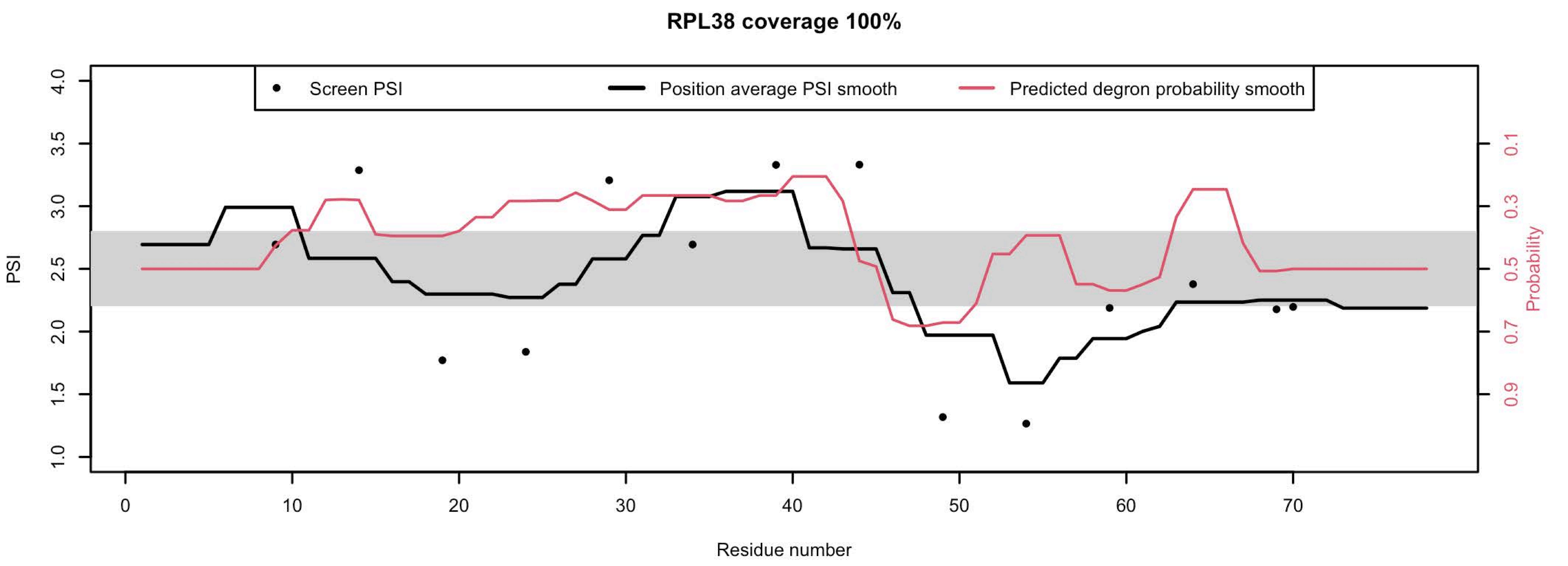

RPL38 coverage 100%
Screen PSI
Position average PSI smooth
Predicted degron probability smooth
PSI
1.0
1.5
2.0
2.5
3.0
3.5
4.0
Probability
0.1
0.3
0.5
0.7
0.9
0
10
20
30
40
50
60
70
Residue number

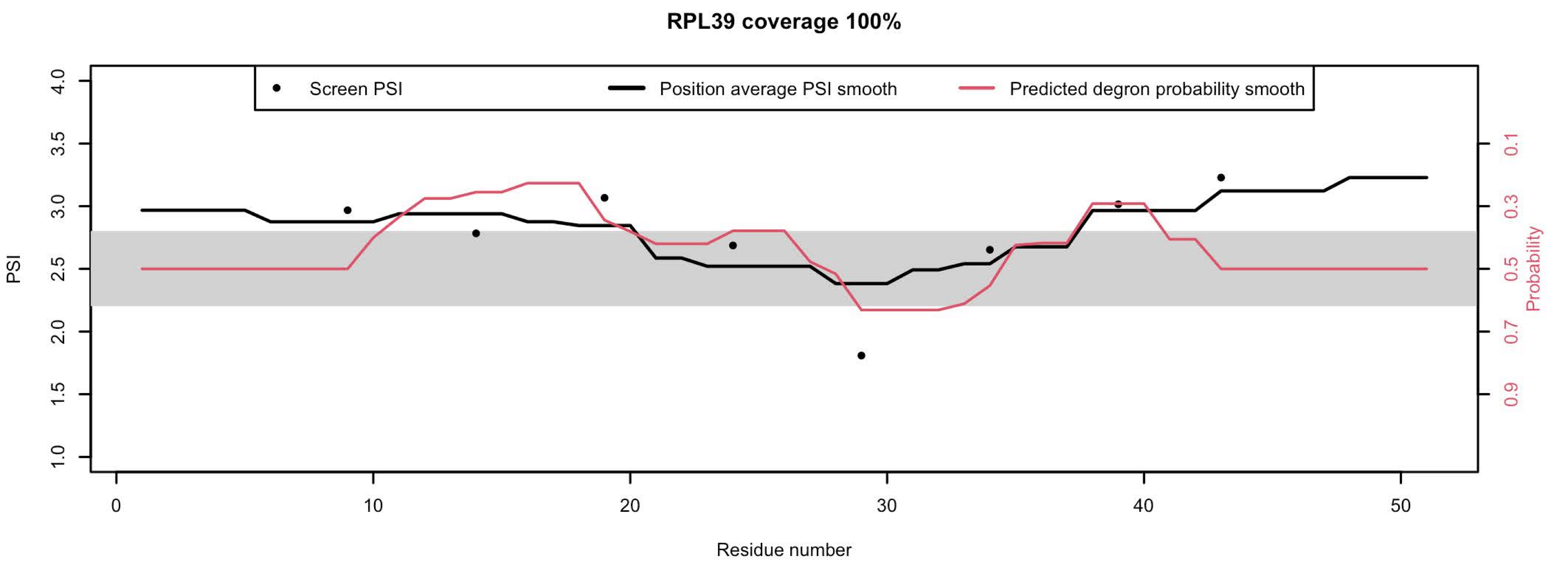

RPL39 coverage 100%
Screen PSI
Position average PSI smooth
Predicted degron probability smooth
PSI
1.0
1.5
2.0
2.5
3.0
3.5
4.0
0.1
0.3
0.5
0.7
0.9
Probability
0
10
20
30
40
50
Residue number

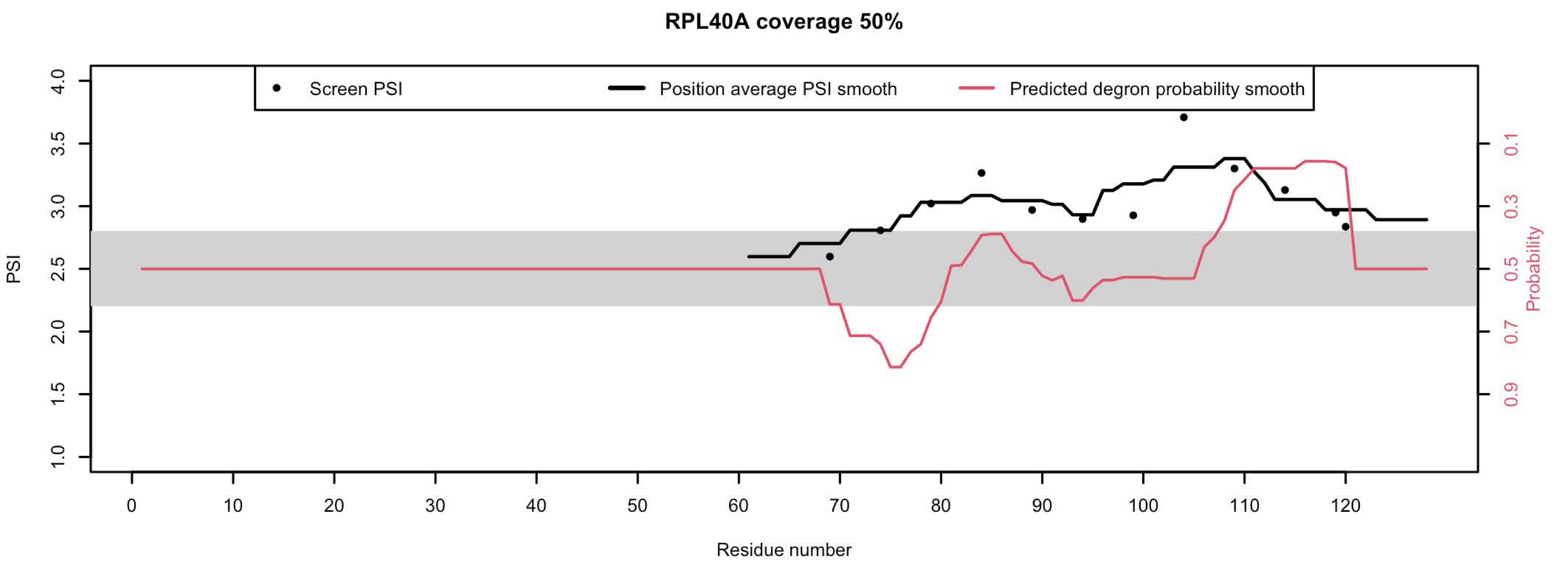

RPL40A coverage 50%
Screen PSI
Position average PSI smooth
Predicted degron probability smooth
PSI
4.0
3.5
3.0
2.5
2.0
1.5
1.0
0.1
0.3
0.5
0.7
0.9
Probability
0
10
20
30
40
50
60
70
80
90
100
110
120
Residue number

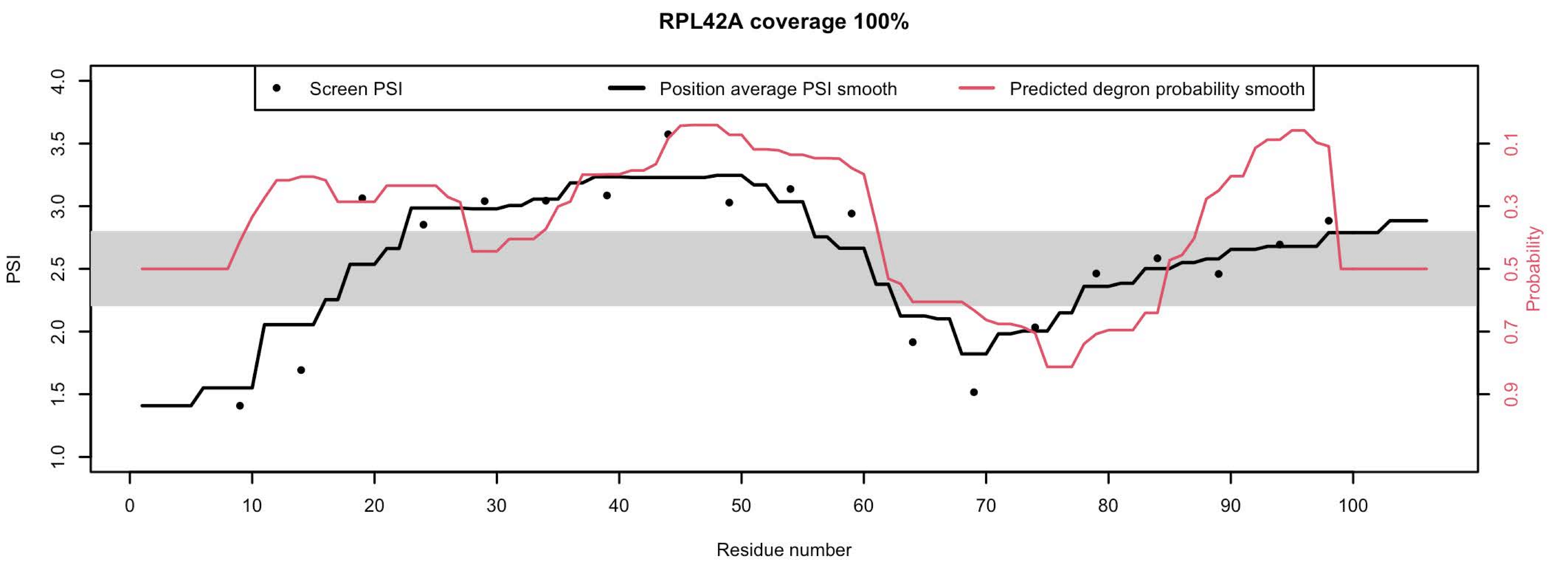

RPL42A coverage 100%
Screen PSI
Position average PSI smooth
Predicted degron probability smooth
PSI
1.0
1.5
2.0
2.5
3.0
3.5
4.0
Probability
0.1
0.3
0.5
0.7
0.9
0
10
20
30
40
50
60
70
80
90
100
Residue number

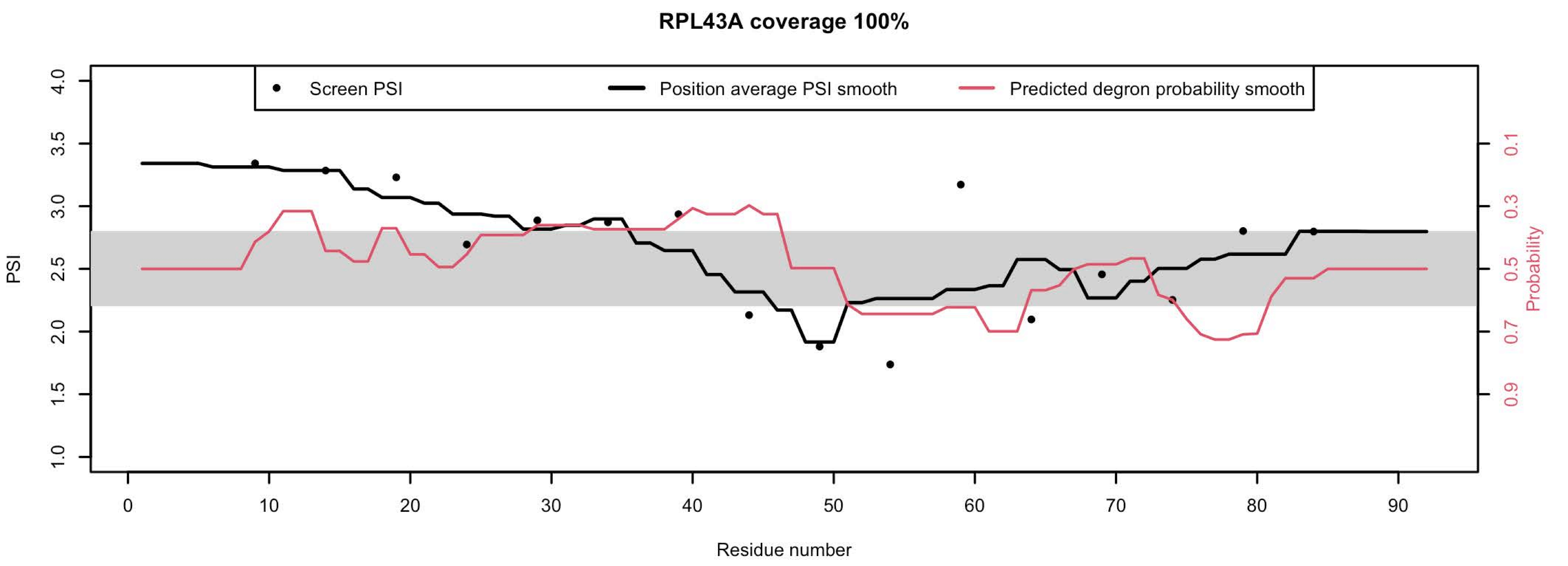
RPL43A coverage 100%
Screen PSI
Position average PSI smooth
Predicted degron probability smooth
PSI
Probability
Residue number
4.0
3.5
3.0
2.5
2.0
1.5
1.0
0.1
0.3
0.5
0.7
0.9
0
10
20
30
40
50
60
70
80
90

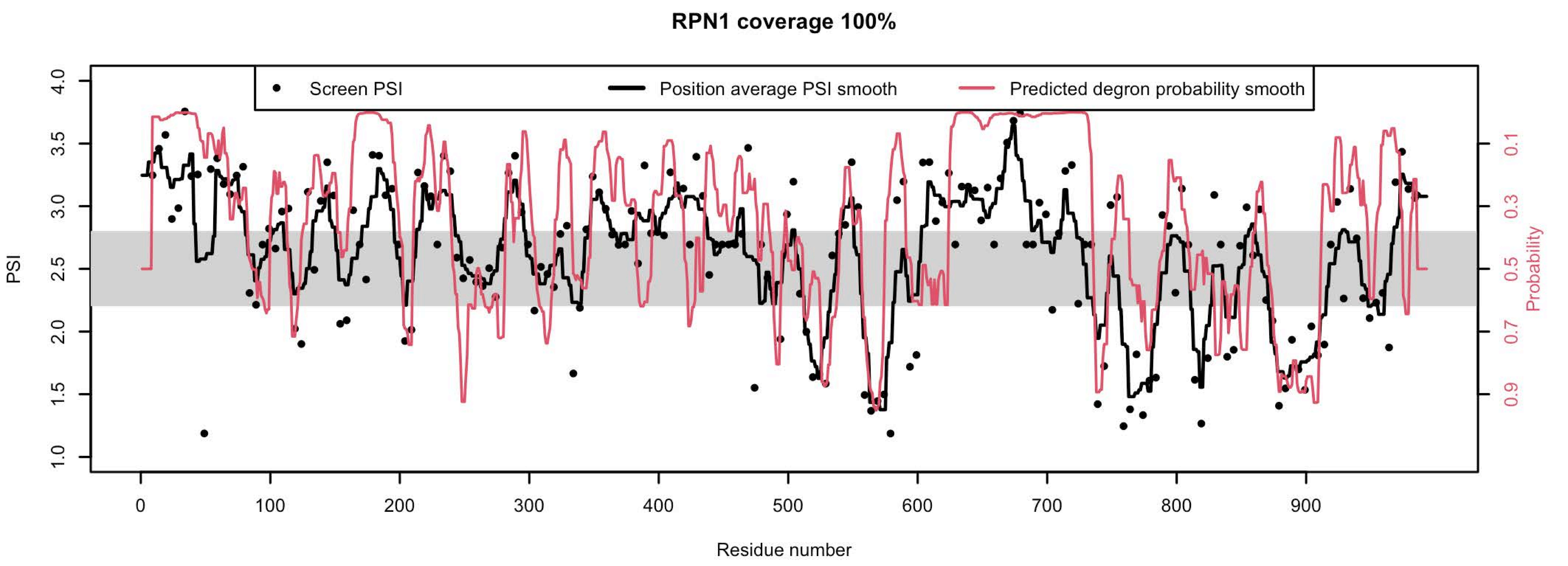
RPN1 coverage 100%
Screen PSI
Position average PSI smooth
Predicted degron probability smooth
PSI
1.0
1.5
2.0
2.5
3.0
3.5
4.0
Probability
0.1
0.3
0.5
0.7
0.9
0
100
200
300
400
500
600
700
800
900
Residue number

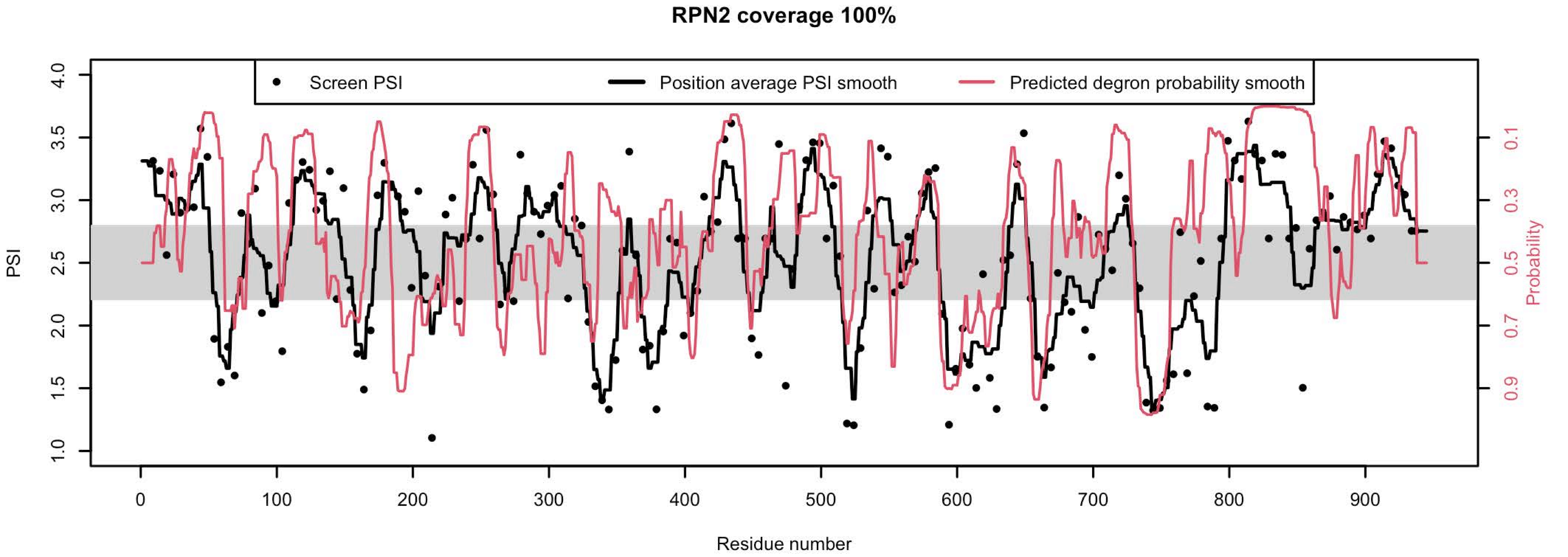

RPN2 coverage 100%
Screen PSI
Position average PSI smooth
Predicted degron probability smooth
PSI
1.0
1.5
2.0
2.5
3.0
3.5
4.0
Probability
0.1
0.3
0.5
0.7
0.9
Residue number
0
100
200
300
400
500
600
700
800
900

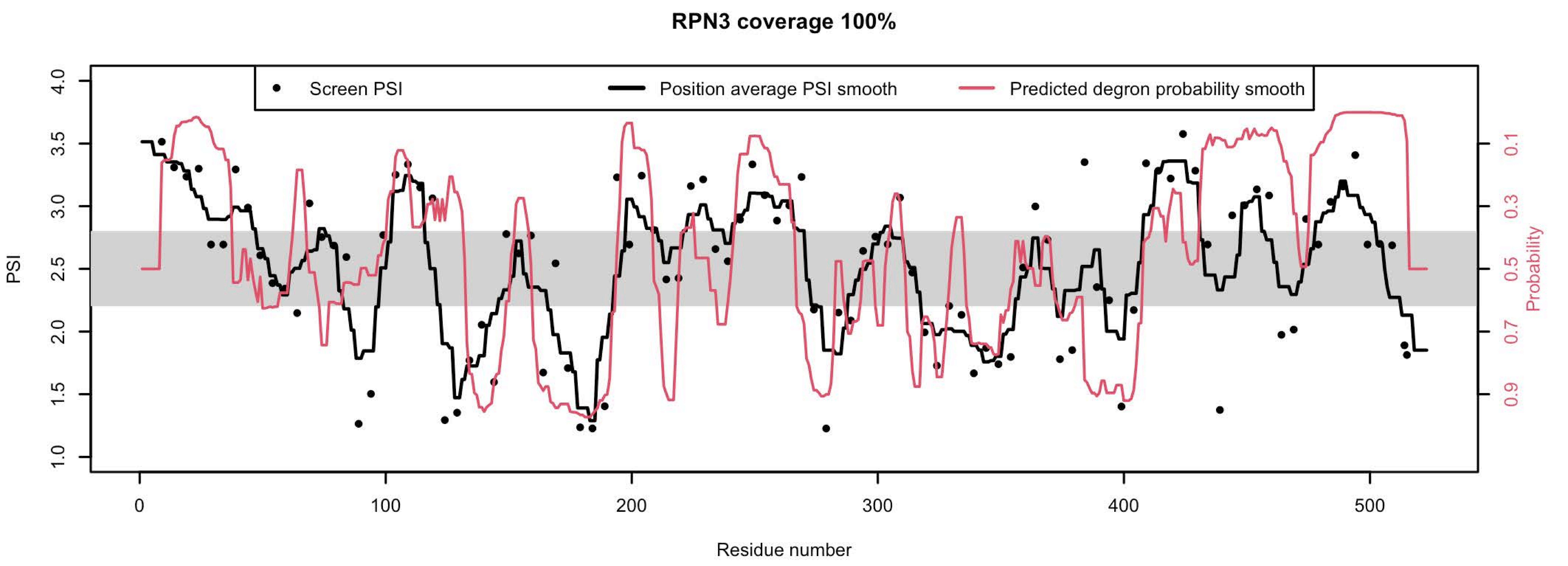

RPN3 coverage 100%
Screen PSI
Position average PSI smooth
Predicted degron probability smooth
PSI
1.0
1.5
2.0
2.5
3.0
3.5
4.0
Probability
0.1
0.3
0.5
0.7
0.9
0
100
200
300
400
500
Residue number

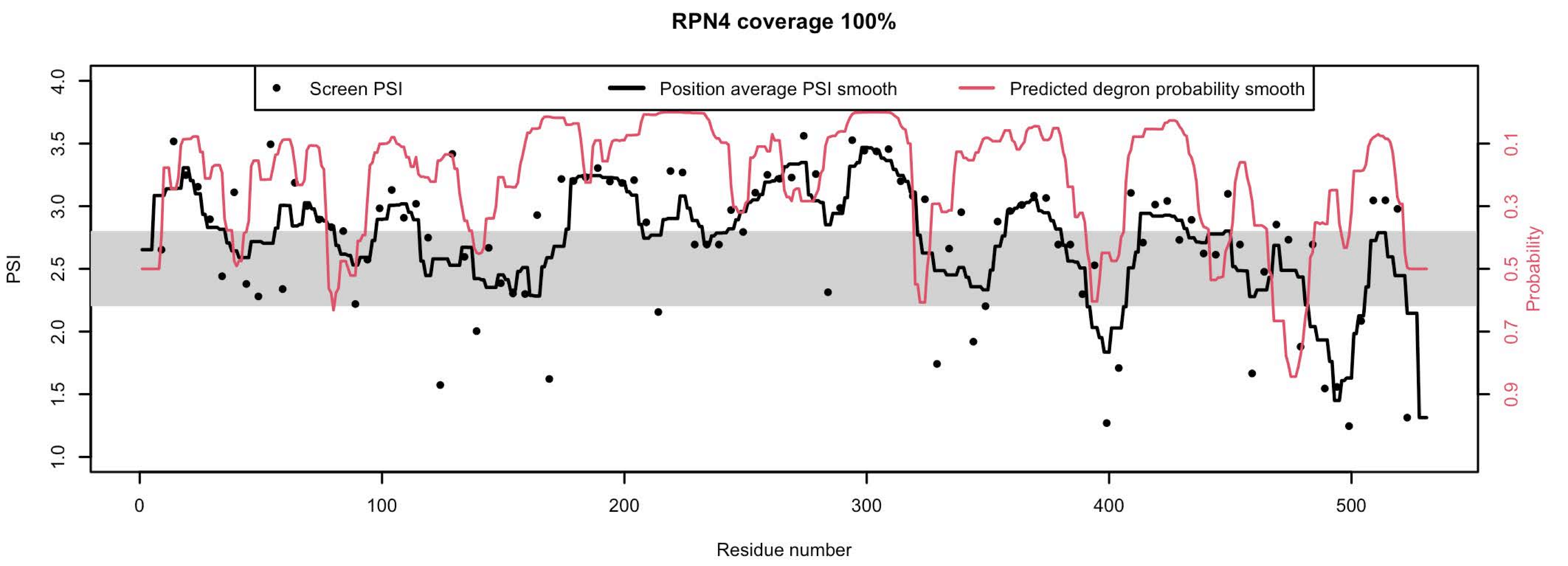

RPN4 coverage 100%
Screen PSI
Position average PSI smooth
Predicted degron probability smooth
PSI
Probability
Residue number
1.0
1.5
2.0
2.5
3.0
3.5
4.0
0.1
0.3
0.5
0.7
0.9
0
100
200
300
400
500

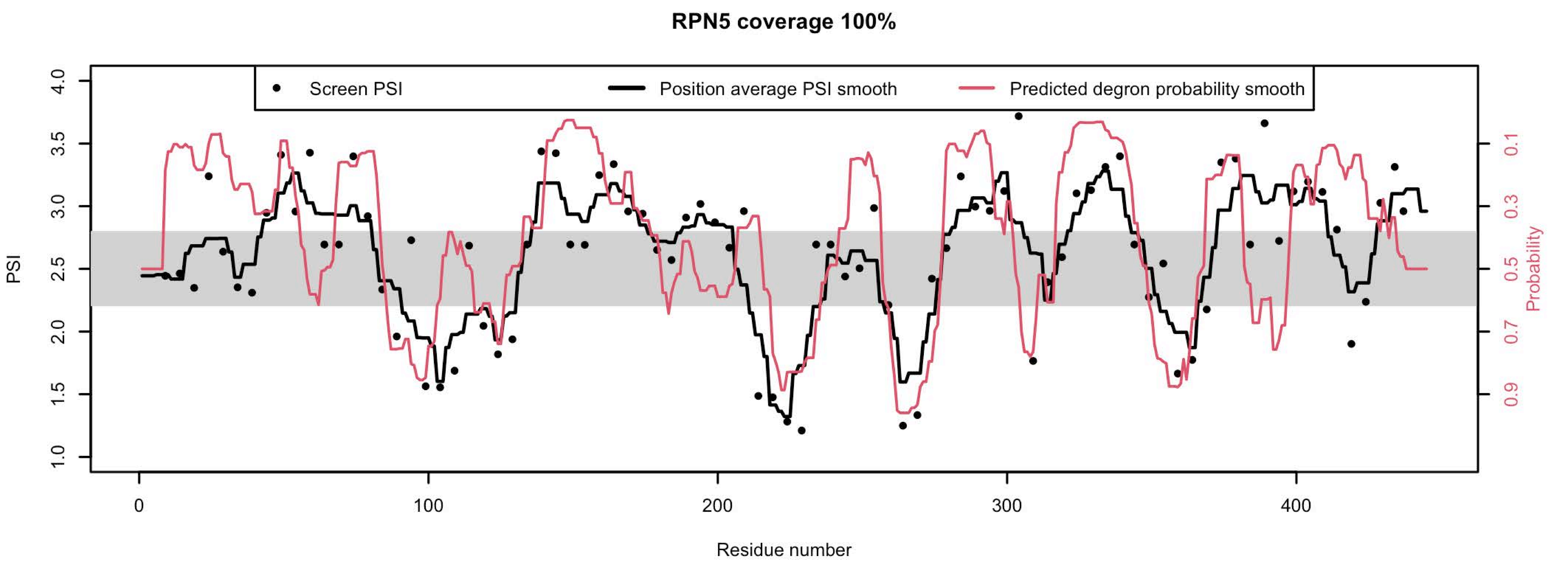
RPN5 coverage 100%
Screen PSI
Position average PSI smooth
Predicted degron probability smooth
PSI
Probability
Residue number
1.0
1.5
2.0
2.5
3.0
3.5
4.0
0.1
0.3
0.5
0.7
0.9
0
100
200
300
400

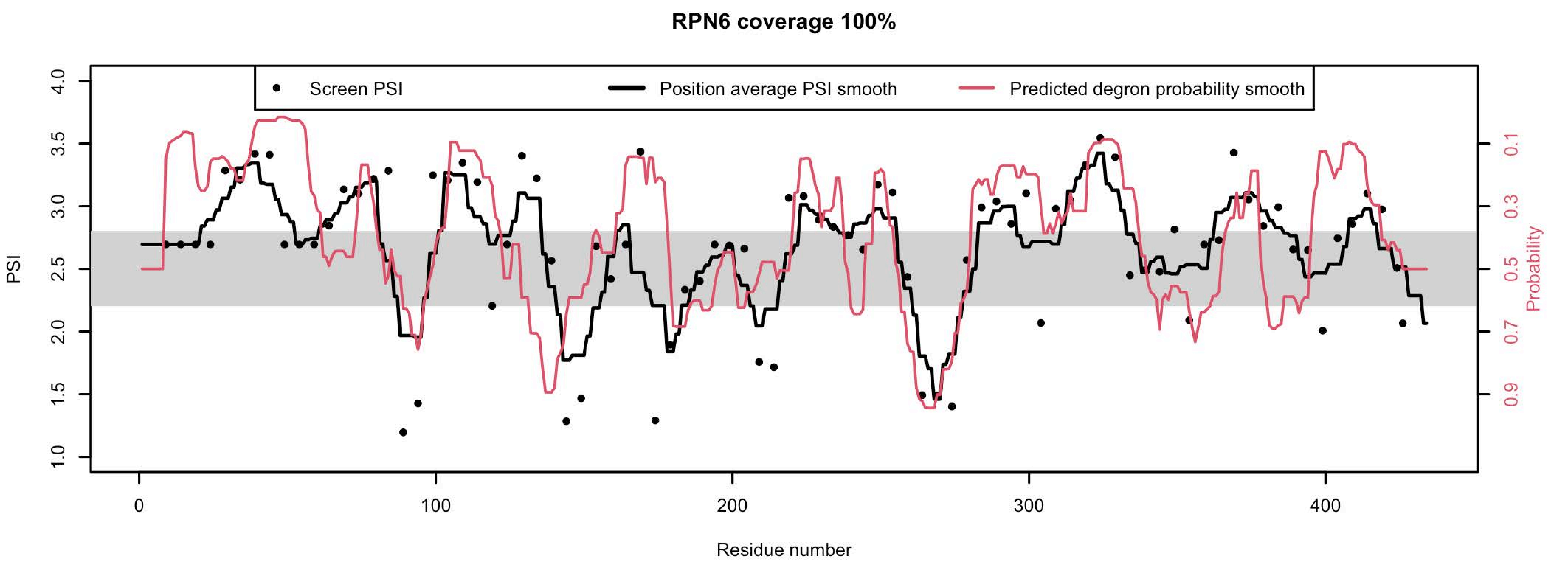

RPN6 coverage 100%
Screen PSI
Position average PSI smooth
Predicted degron probability smooth
PSI
1.0
1.5
2.0
2.5
3.0
3.5
4.0
Probability
0.1
0.3
0.5
0.7
0.9
Residue number
0
100
200
300
400

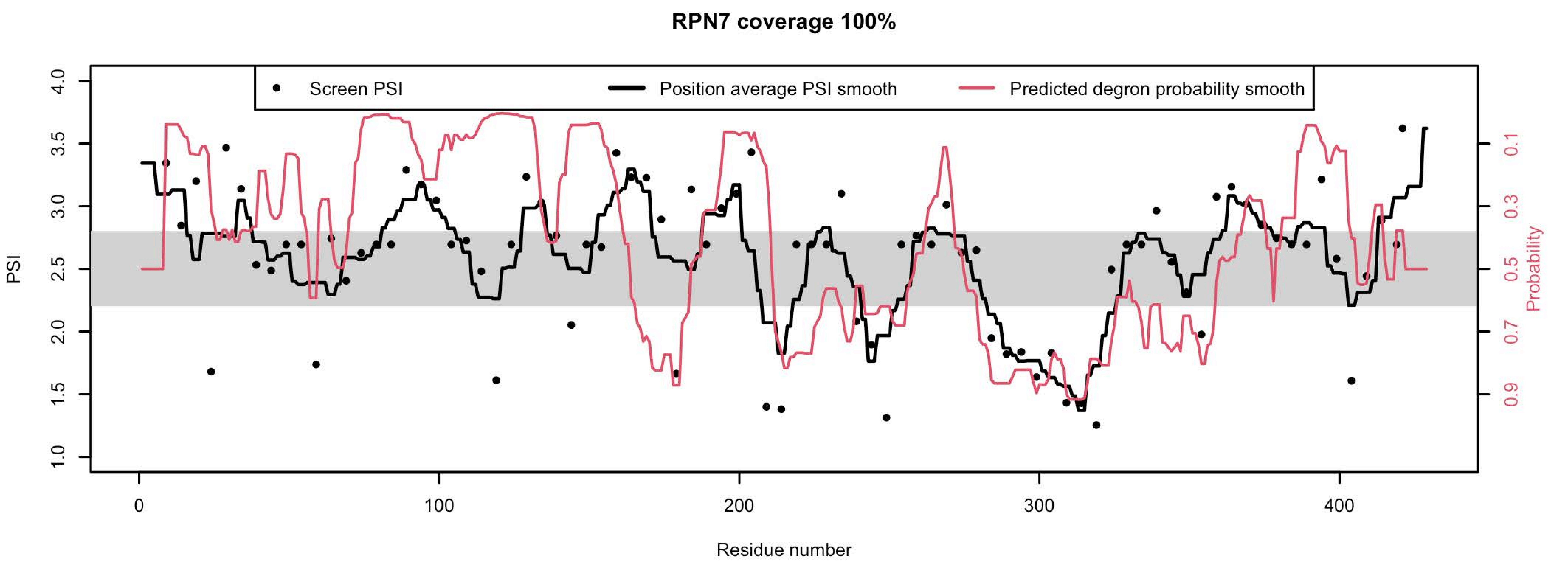
RPN7 coverage 100%
Screen PSI
Position average PSI smooth
Predicted degron probability smooth
PSI
Probability
Residue number
1.0
1.5
2.0
2.5
3.0
3.5
4.0
0.1
0.3
0.5
0.7
0.9
0
100
200
300
400

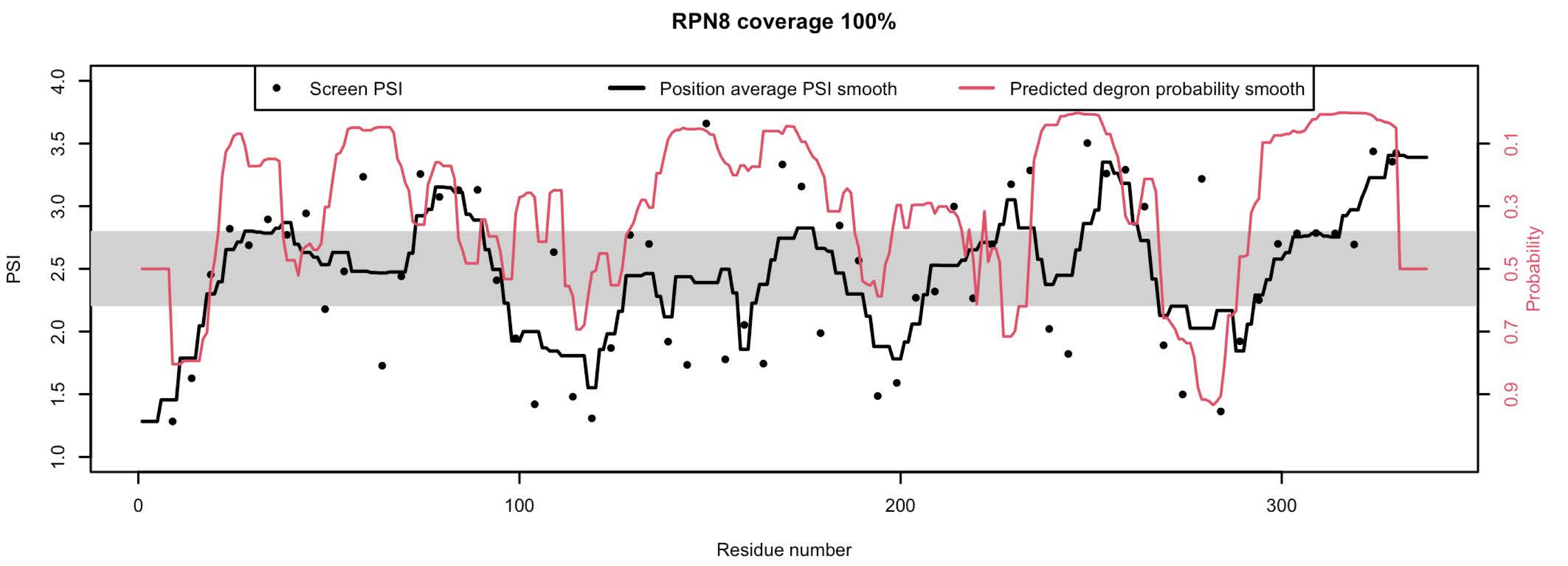

RPN8 coverage 100%
Screen PSI
Position average PSI smooth
Predicted degron probability smooth
PSI
1.0
1.5
2.0
2.5
3.0
3.5
4.0
Probability
0.1
0.3
0.5
0.7
0.9
0
100
200
300
Residue number

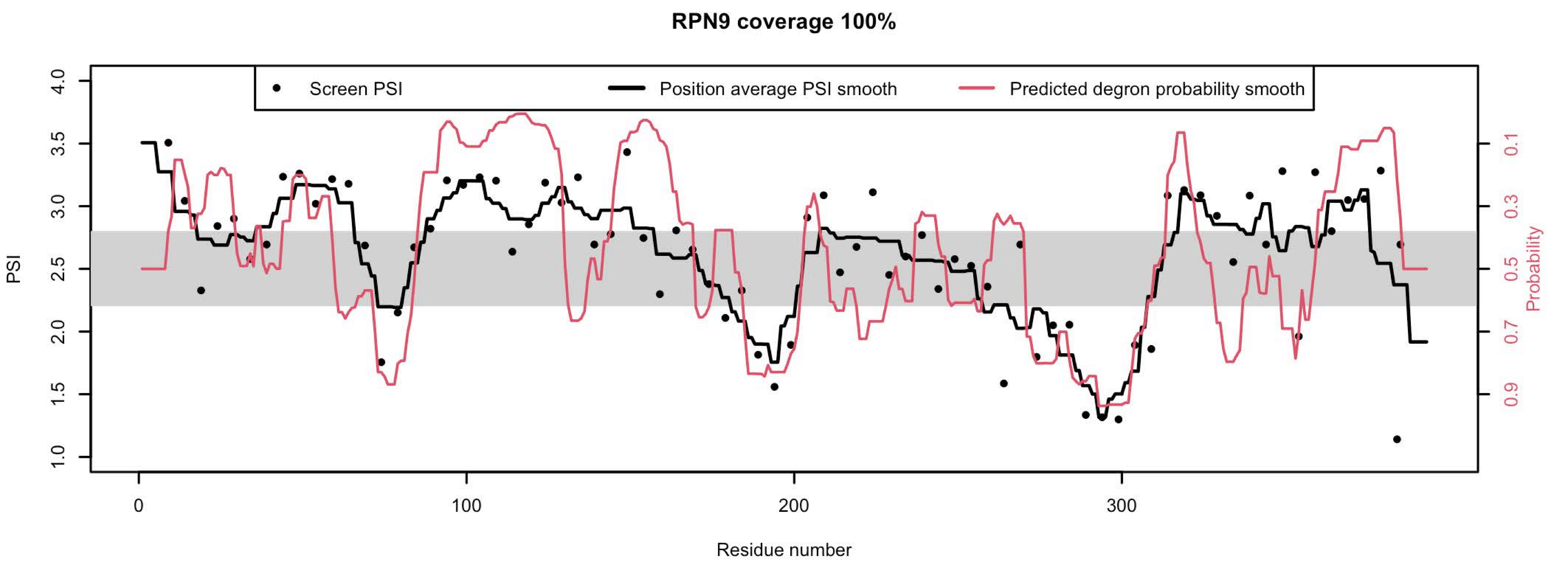

RPN9 coverage 100%
Screen PSI
Position average PSI smooth
Predicted degron probability smooth
PSI
4.0
3.5
3.0
2.5
2.0
1.5
1.0
0.1
0.3
0.5
0.7
0.9
Probability
0
100
200
300
Residue number

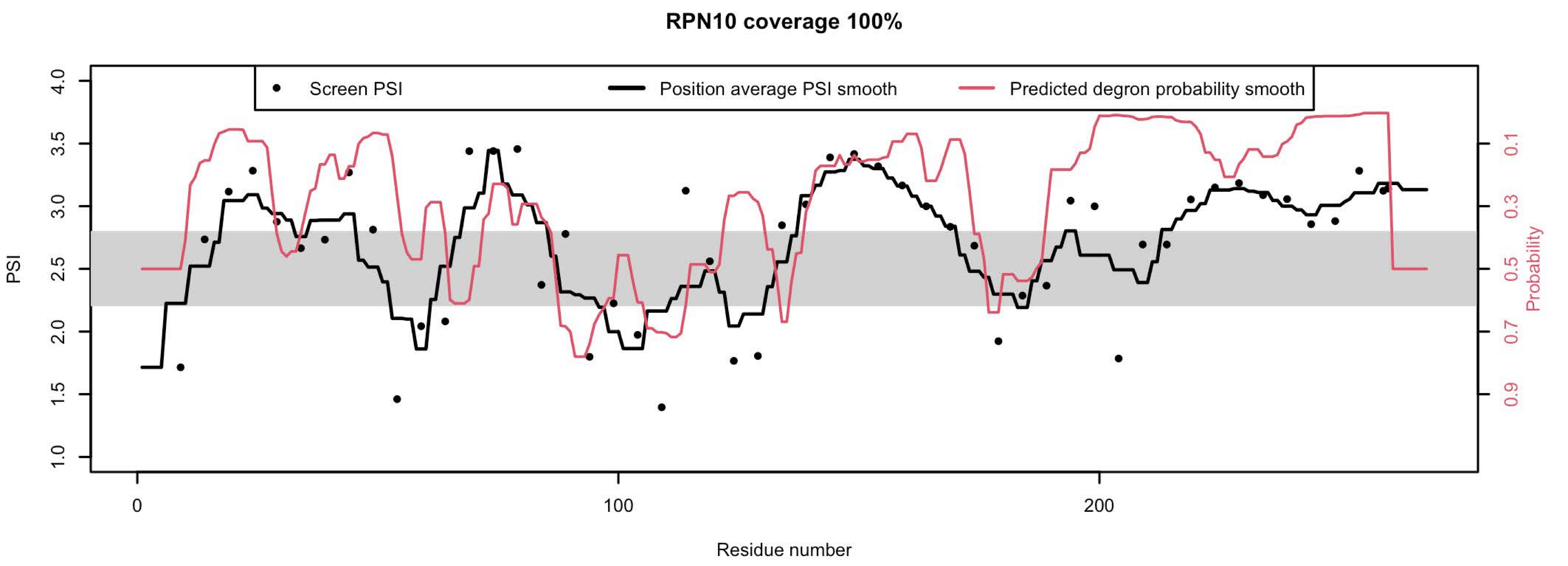

RPN10 coverage 100%
Screen PSI
Position average PSI smooth
Predicted degron probability smooth
PSI
1.0
1.5
2.0
2.5
3.0
3.5
4.0
Probability
0.1
0.3
0.5
0.7
0.9
0
100
200
Residue number

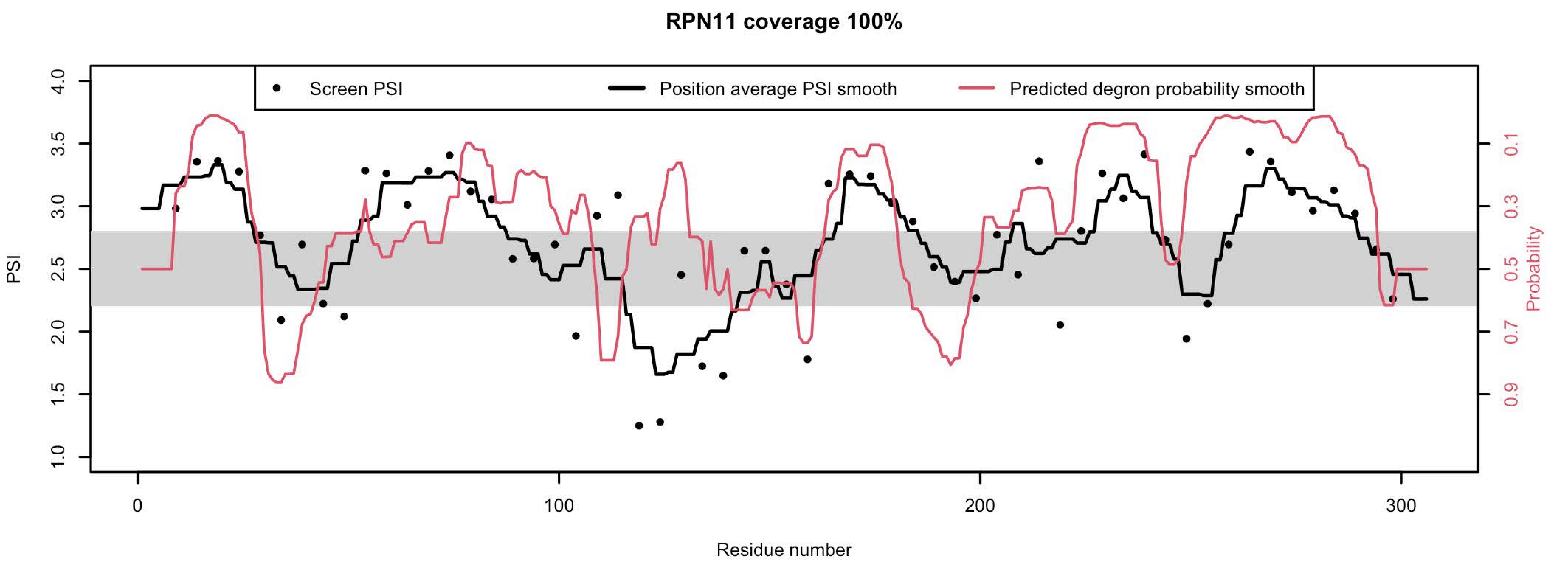

RPN11 coverage 100%
Screen PSI
Position average PSI smooth
Predicted degron probability smooth
PSI
1.0
1.5
2.0
2.5
3.0
3.5
4.0
Probability
0.1
0.3
0.5
0.7
0.9
0
100
200
300
Residue number

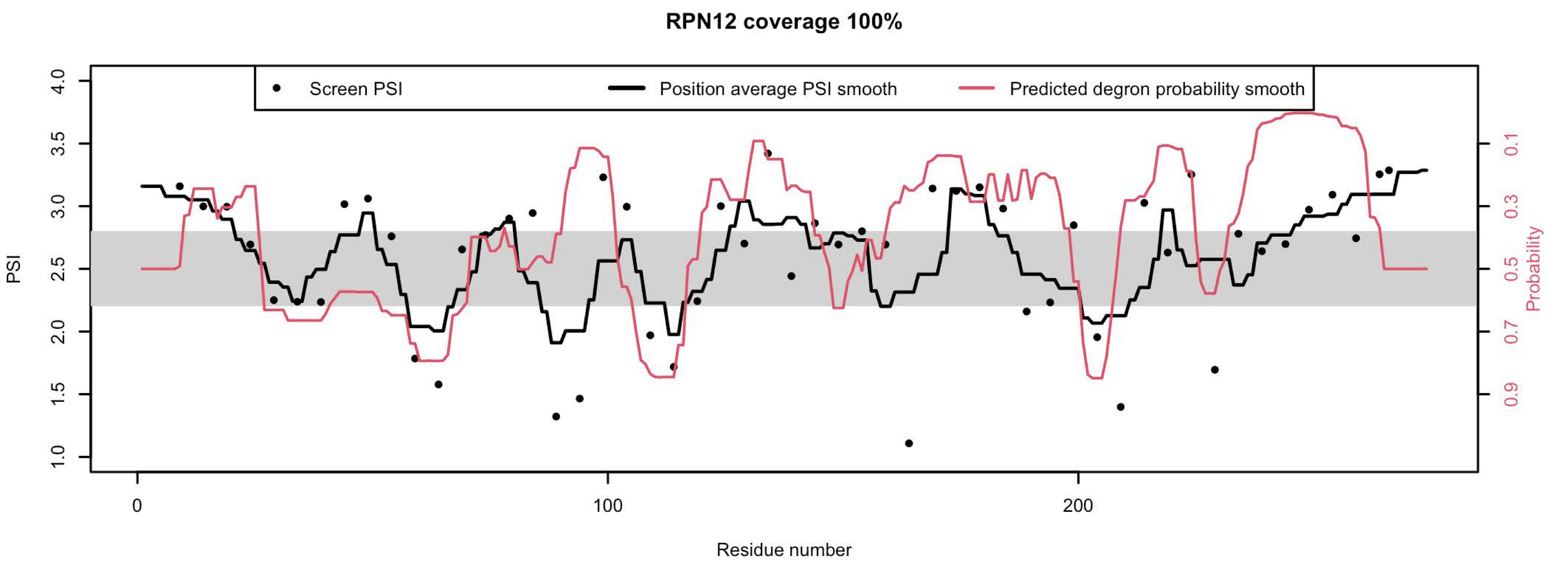

RPN12 coverage 100%
Screen PSI
Position average PSI smooth
Predicted degron probability smooth
PSI
Probability
Residue number
1.0
1.5
2.0
2.5
3.0
3.5
4.0
0.1
0.3
0.5
0.7
0.9
0
100
200

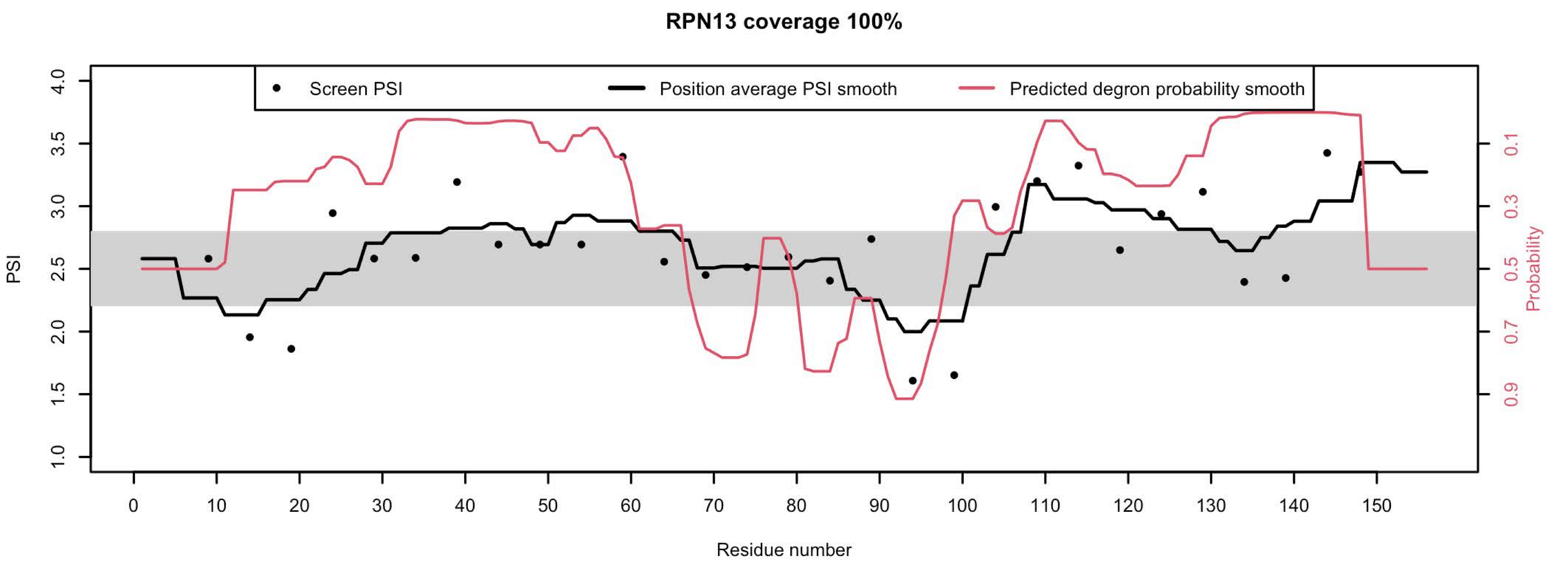
RPN13 coverage 100%
Screen PSI
Position average PSI smooth
Predicted degron probability smooth
PSI
Probability
Residue number
1.0
1.5
2.0
2.5
3.0
3.5
4.0
0.1
0.3
0.5
0.7
0.9
0
10
20
30
40
50
60
70
80
90
100
110
120
130
140
150

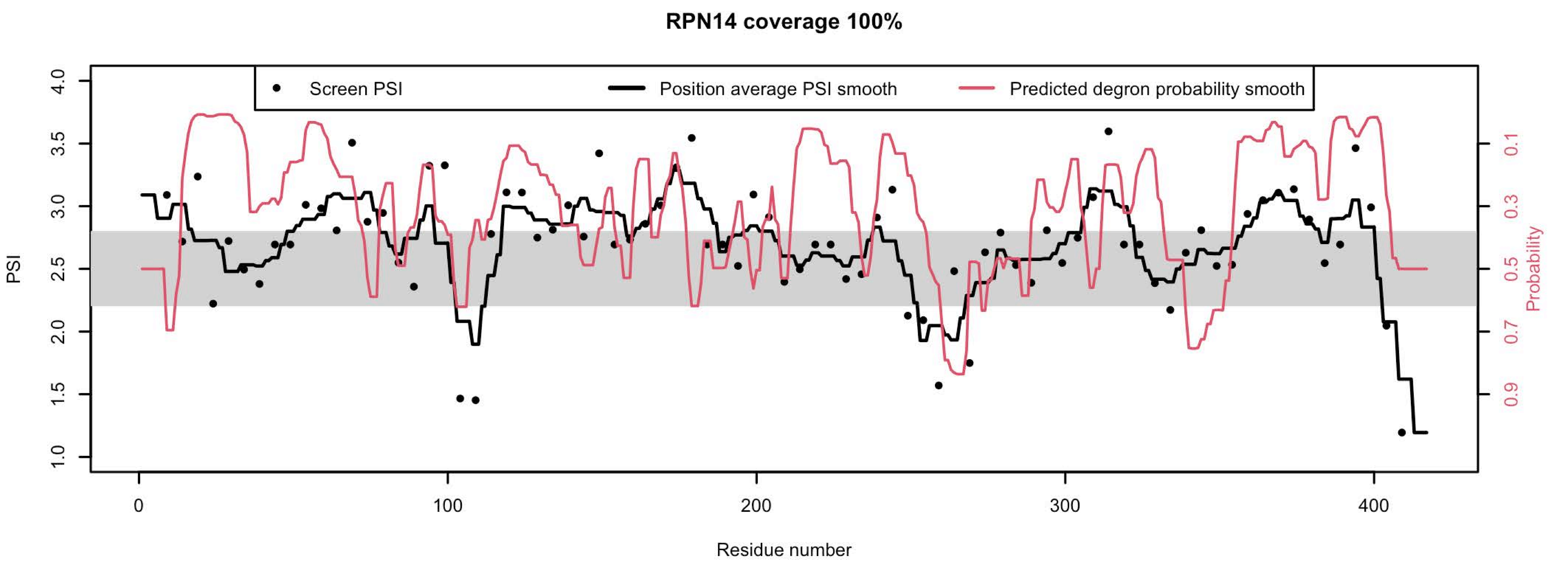
RPN14 coverage 100%
Screen PSI
Position average PSI smooth
Predicted degron probability smooth
PSI
1.0
1.5
2.0
2.5
3.0
3.5
4.0
Probability
0.1
0.3
0.5
0.7
0.9
0
100
200
300
400
Residue number

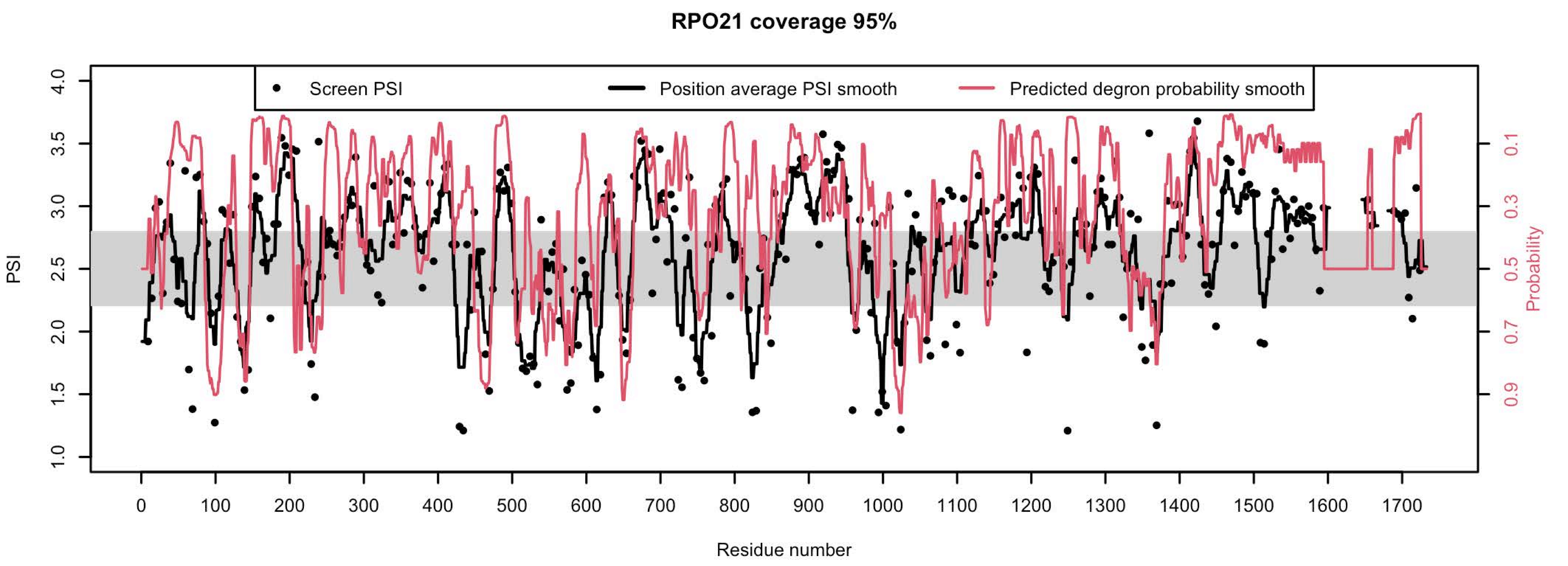

RPO21 coverage 95%
Screen PSI
Position average PSI smooth
Predicted degron probability smooth
PSI
1.0
1.5
2.0
2.5
3.0
3.5
4.0
Probability
0.1
0.3
0.5
0.7
0.9
Residue number
0
100
200
300
400
500
600
700
800
900
1000
1100
1200
1300
1400
1500
1600
1700

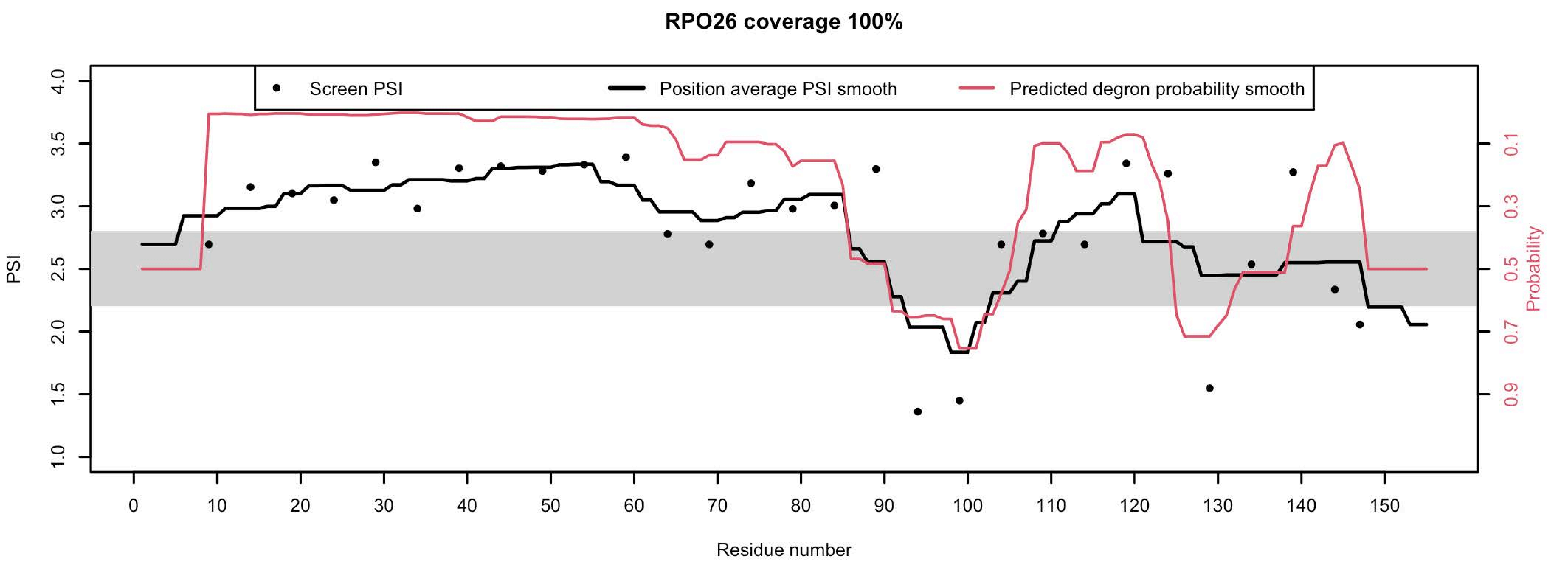

RPO26 coverage 100%
Screen PSI
Position average PSI smooth
Predicted degron probability smooth
PSI
Probability
Residue number
1.0
1.5
2.0
2.5
3.0
3.5
4.0
0.1
0.3
0.5
0.7
0.9
0
10
20
30
40
50
60
70
80
90
100
110
120
130
140
150

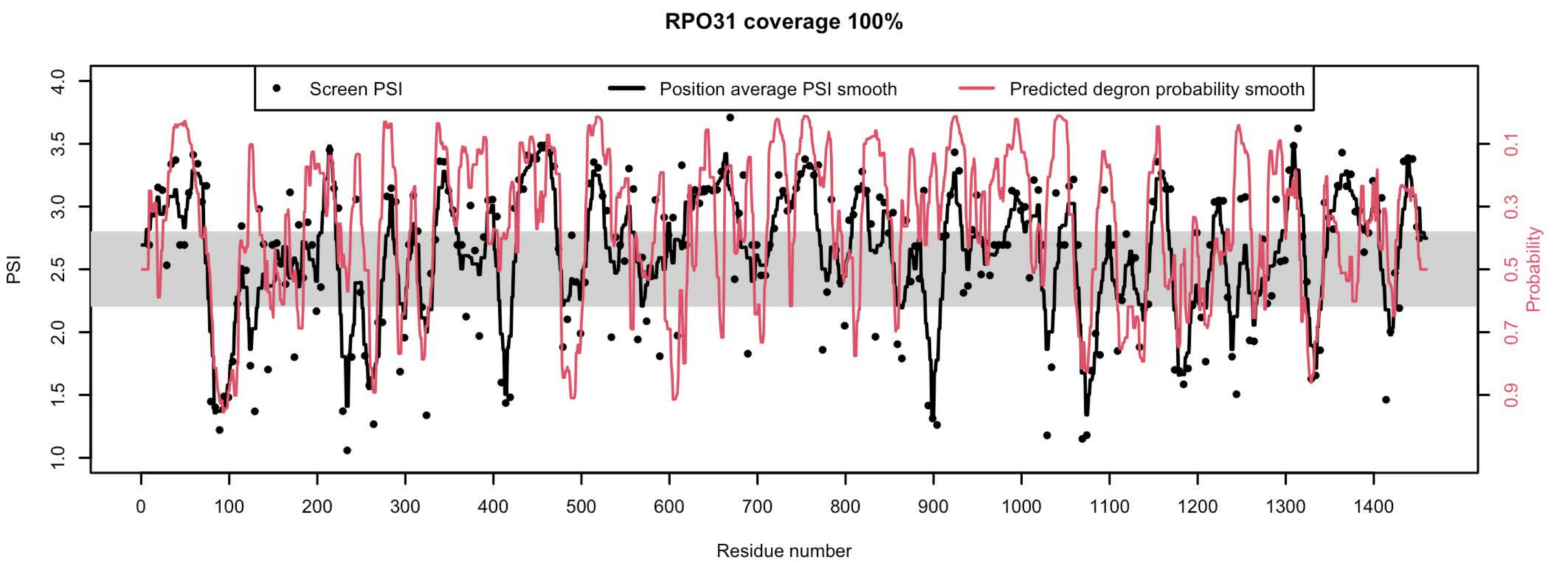
RPO31 coverage 100%
Screen PSI
Position average PSI smooth
Predicted degron probability smooth
PSI
Probability
Residue number
1.0
1.5
2.0
2.5
3.0
3.5
4.0
0.1
0.3
0.5
0.7
0.9
0
100
200
300
400
500
600
700
800
900
1000
1100
1200
1300
1400

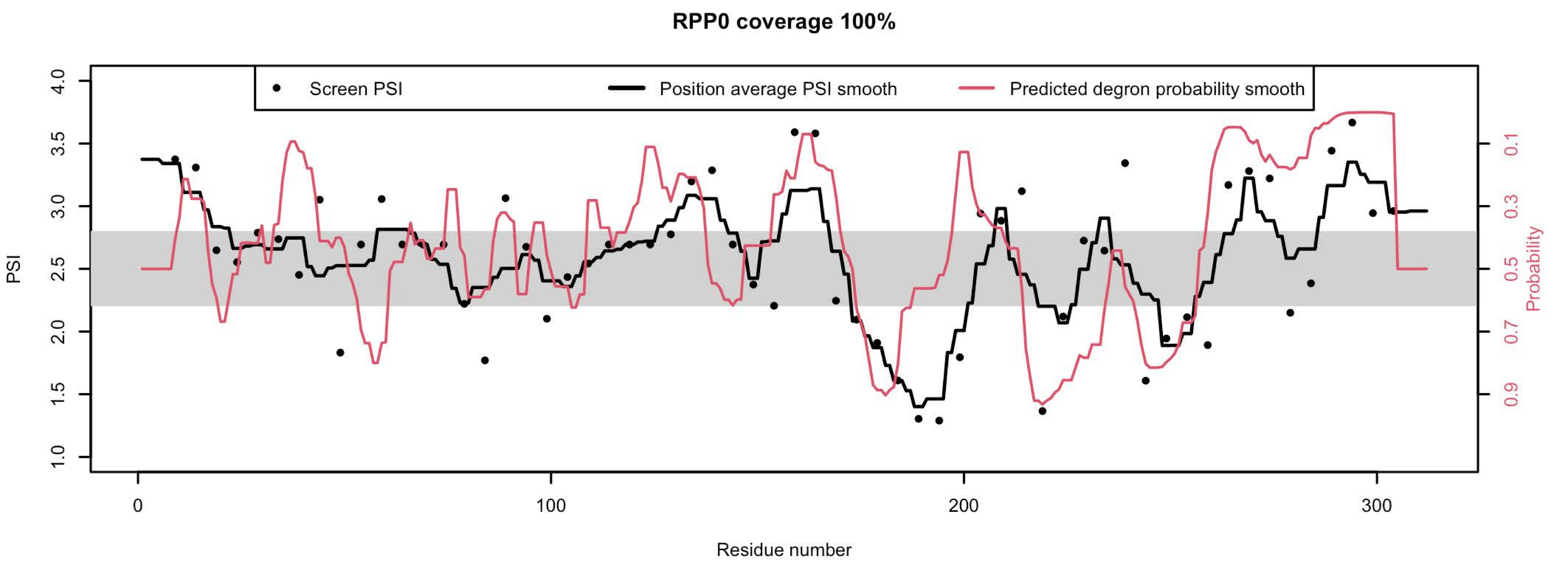

RPP0 coverage 100%
Screen PSI
Position average PSI smooth
Predicted degron probability smooth
PSI
Probability
Residue number
1.0
1.5
2.0
2.5
3.0
3.5
4.0
0.1
0.3
0.5
0.7
0.9
0
100
200
300

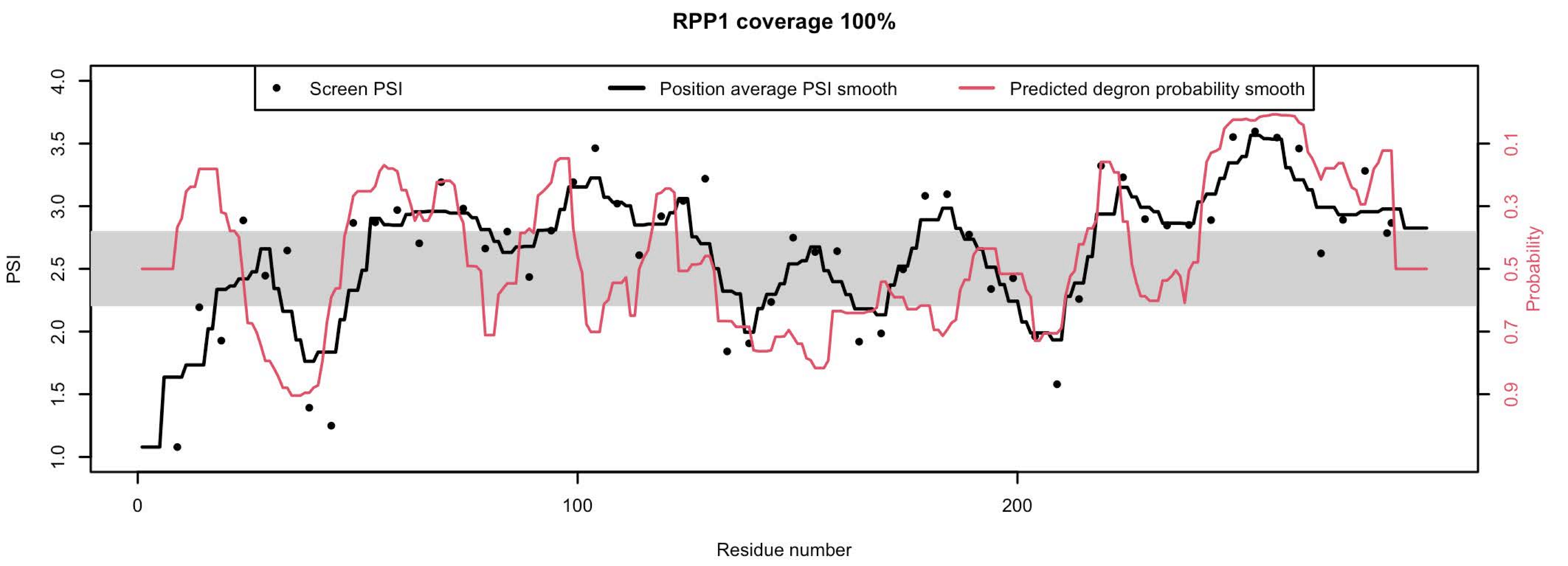
RPP1 coverage 100%
Screen PSI
Position average PSI smooth
Predicted degron probability smooth
PSI
1.0
1.5
2.0
2.5
3.0
3.5
4.0
Probability
0.1
0.3
0.5
0.7
0.9
0
100
200
Residue number

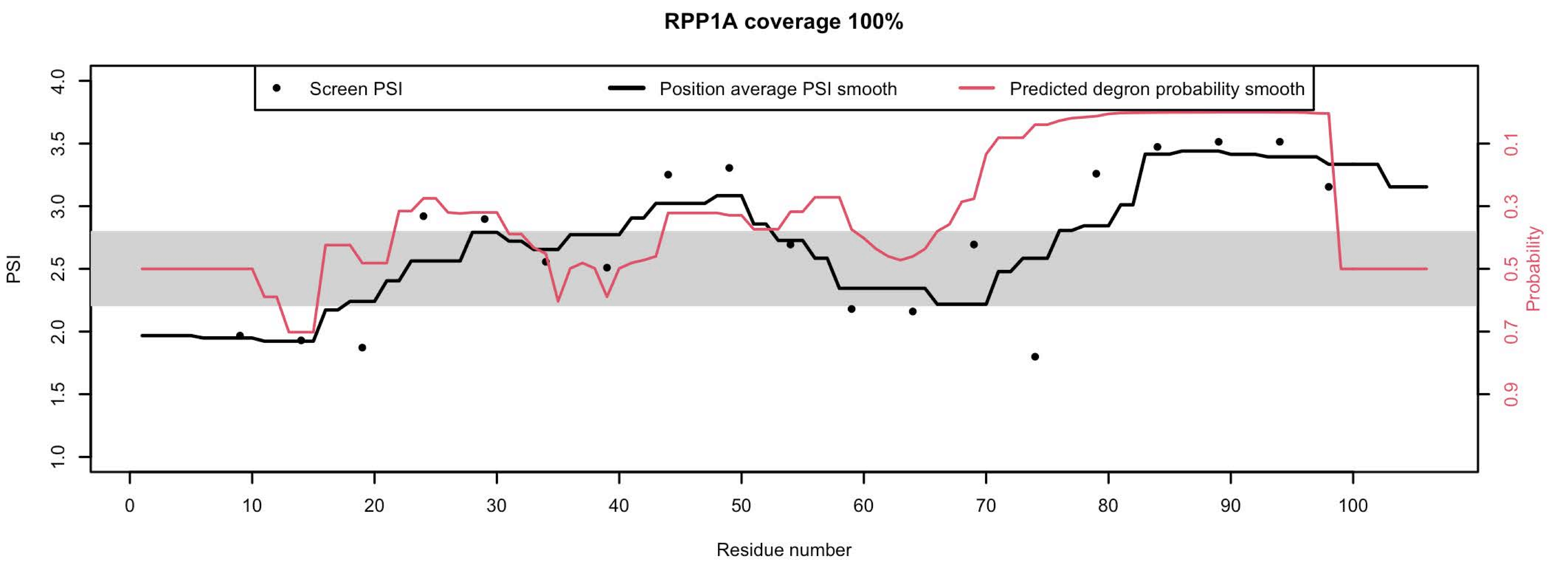

RPP1A coverage 100%
Screen PSI
Position average PSI smooth
Predicted degron probability smooth
PSI
1.0
1.5
2.0
2.5
3.0
3.5
4.0
Probability
0.1
0.3
0.5
0.7
0.9
0
10
20
30
40
50
60
70
80
90
100
Residue number

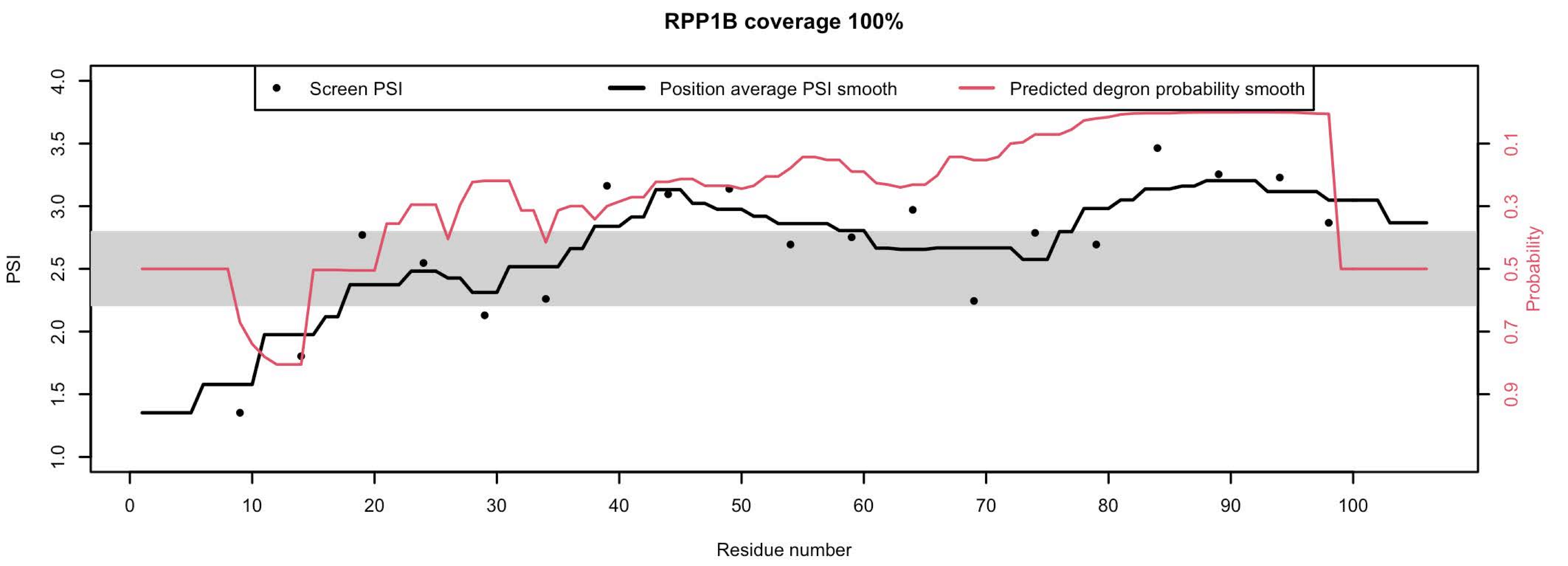
RPP1B coverage 100%
Screen PSI
Position average PSI smooth
Predicted degron probability smooth
PSI
Probability
Residue number
1.0
1.5
2.0
2.5
3.0
3.5
4.0
0.1
0.3
0.5
0.7
0.9
0
10
20
30
40
50
60
70
80
90
100

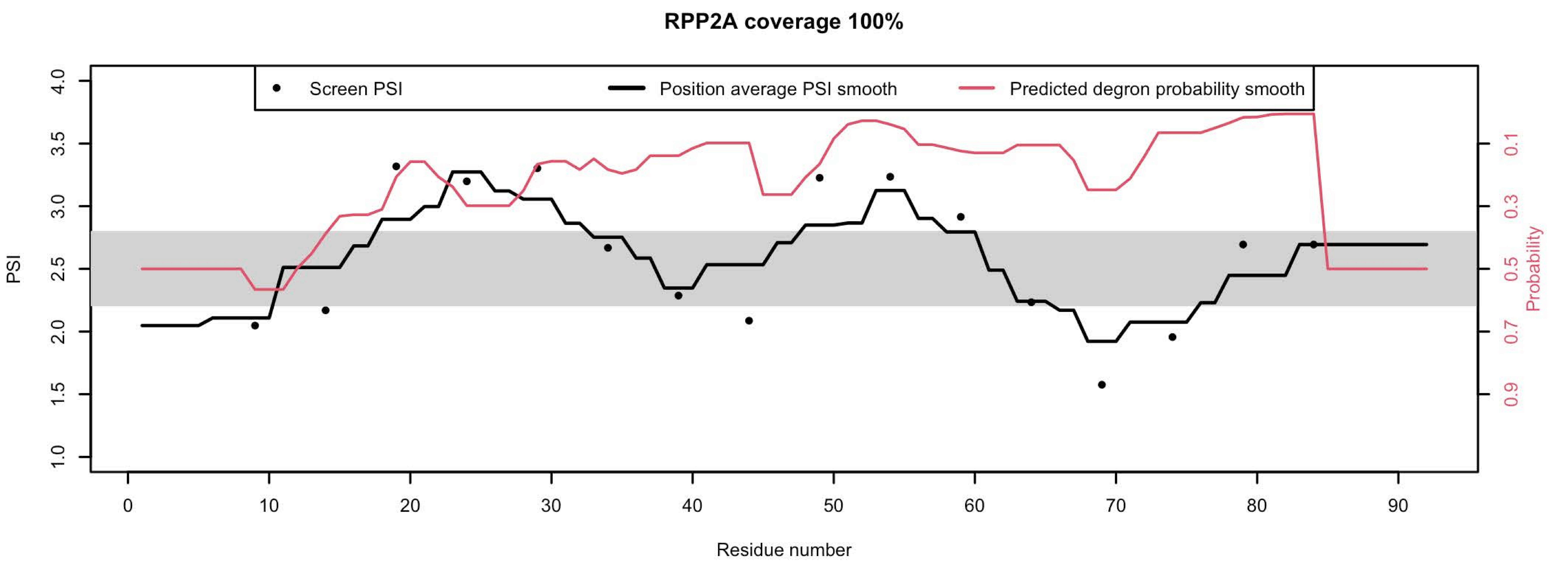

RPP2A coverage 100%
Screen PSI
Position average PSI smooth
Predicted degron probability smooth
PSI
1.0
1.5
2.0
2.5
3.0
3.5
4.0
Probability
0.1
0.3
0.5
0.7
0.9
Residue number
0
10
20
30
40
50
60
70
80
90

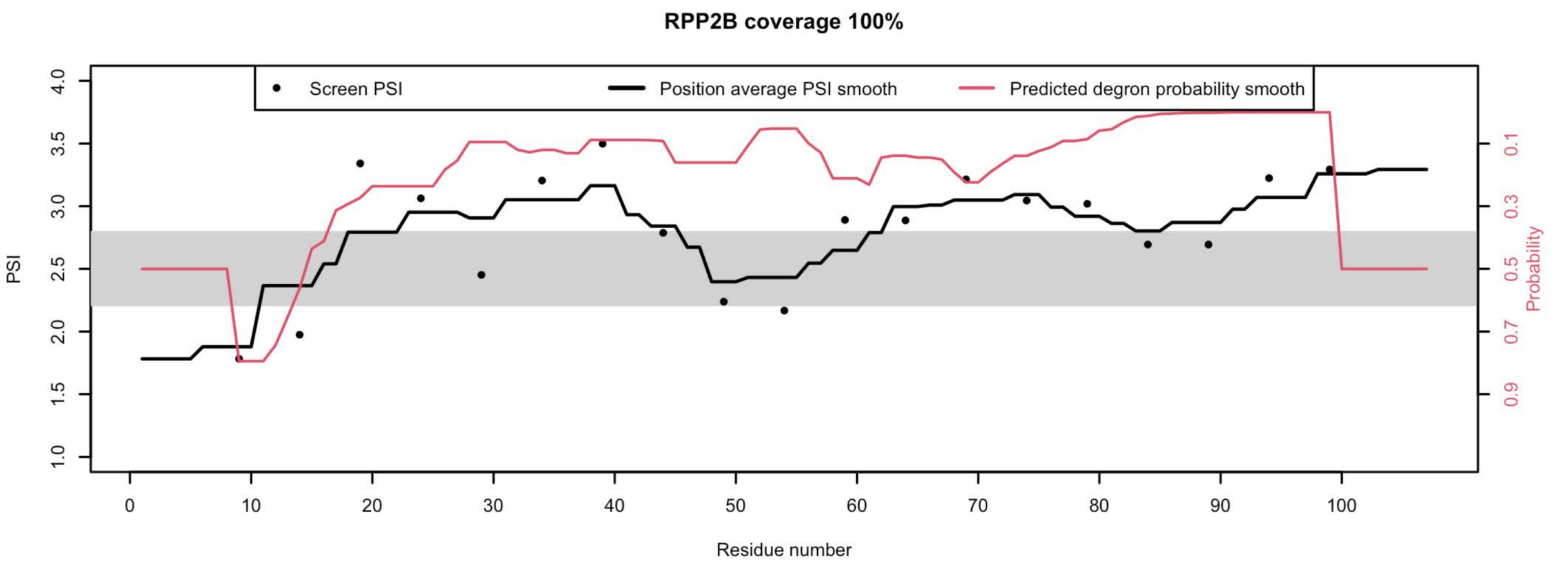

RPP2B coverage 100%
Screen PSI
Position average PSI smooth
Predicted degron probability smooth
PSI
1.0
1.5
2.0
2.5
3.0
3.5
4.0
Probability
0.1
0.3
0.5
0.7
0.9
Residue number
0
10
20
30
40
50
60
70
80
90
100

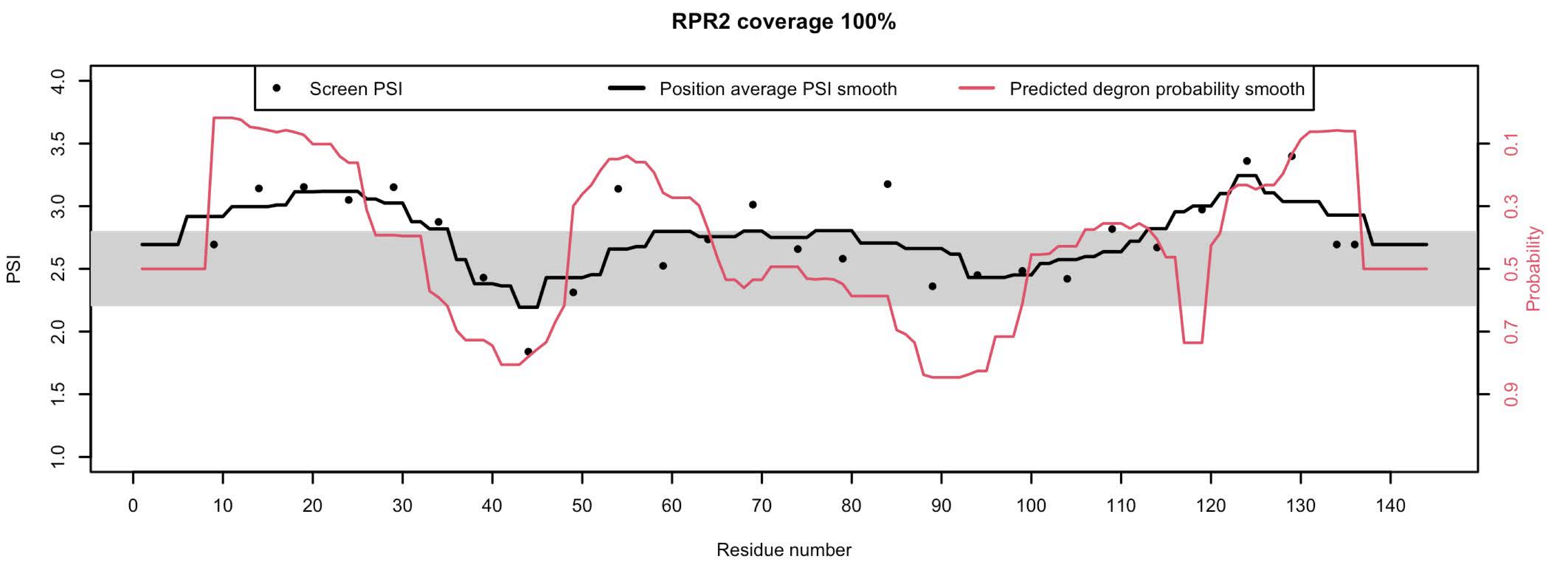

RPR2 coverage 100%
Screen PSI
Position average PSI smooth
Predicted degron probability smooth
PSI
4.0
3.5
3.0
2.5
2.0
1.5
1.0
Probability
0.1
0.3
0.5
0.7
0.9
0
10
20
30
40
50
60
70
80
90
100
110
120
130
140
Residue number

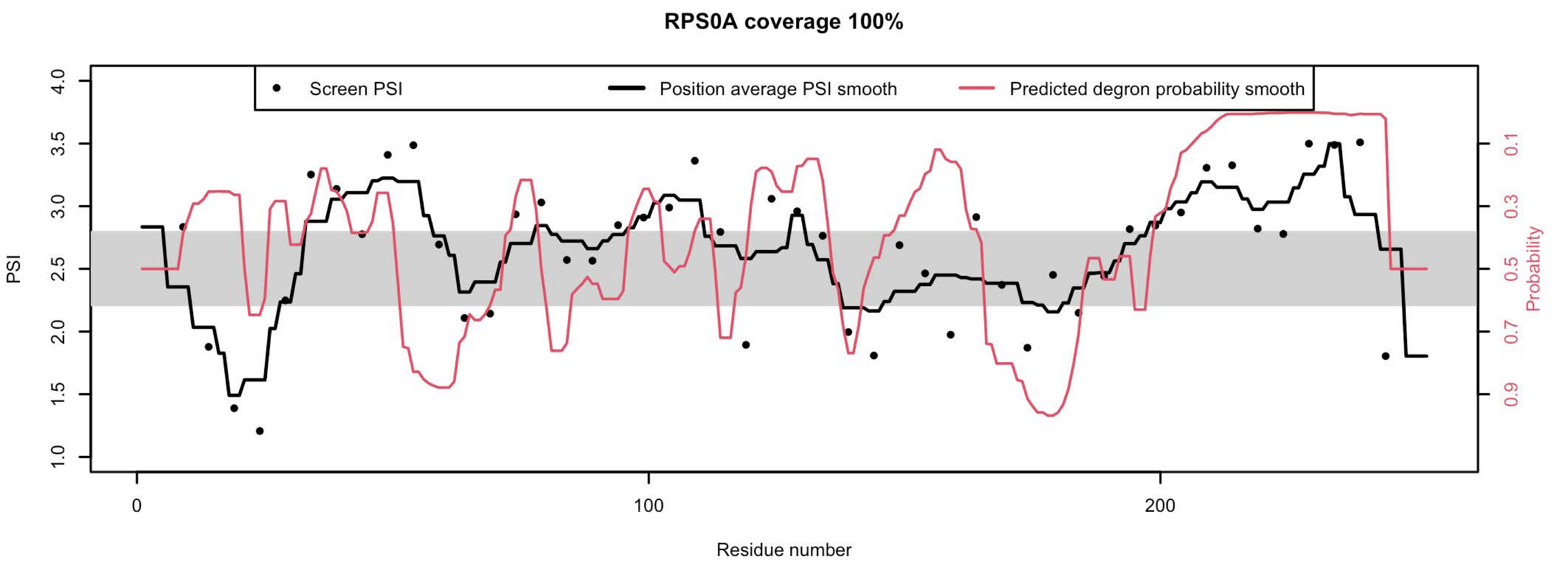

RPS0A coverage 100%
Screen PSI
Position average PSI smooth
Predicted degron probability smooth
PSI
1.0
1.5
2.0
2.5
3.0
3.5
4.0
Probability
0.1
0.3
0.5
0.7
0.9
Residue number
0
100
200

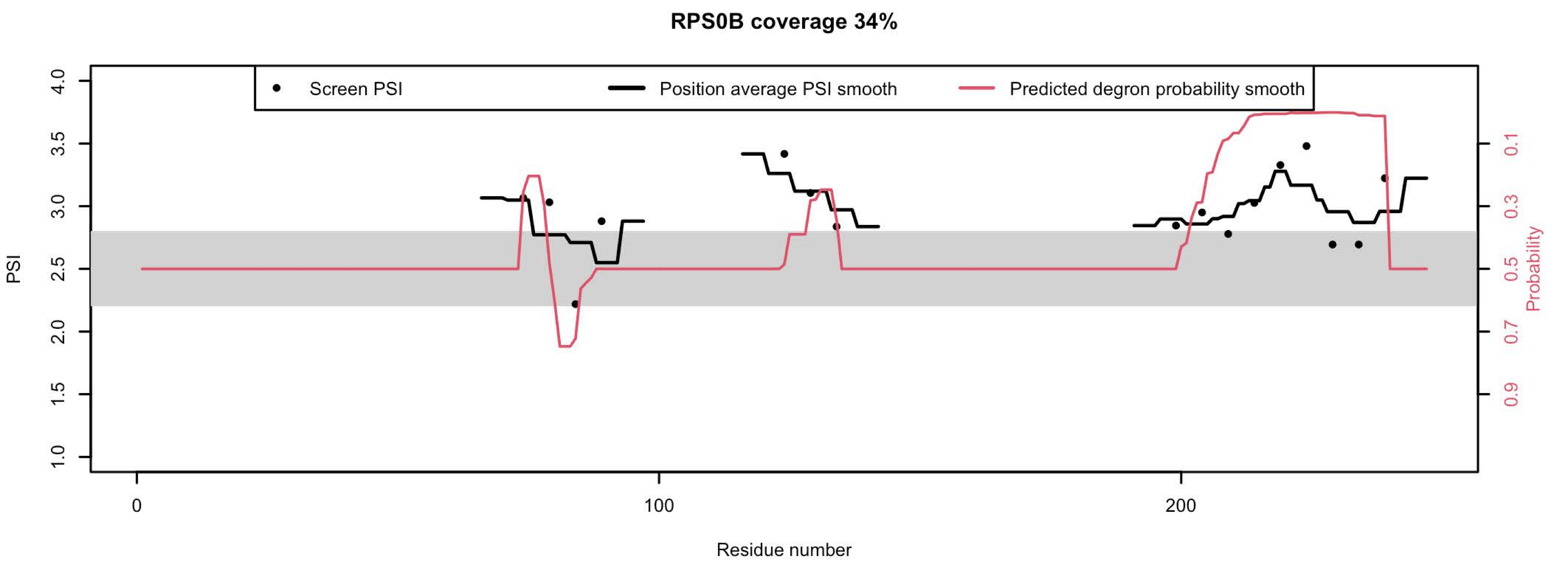

RPS0B coverage 34%
Screen PSI
Position average PSI smooth
Predicted degron probability smooth
PSI
1.0
1.5
2.0
2.5
3.0
3.5
4.0
Probability
0.1
0.3
0.5
0.7
0.9
Residue number
0
100
200

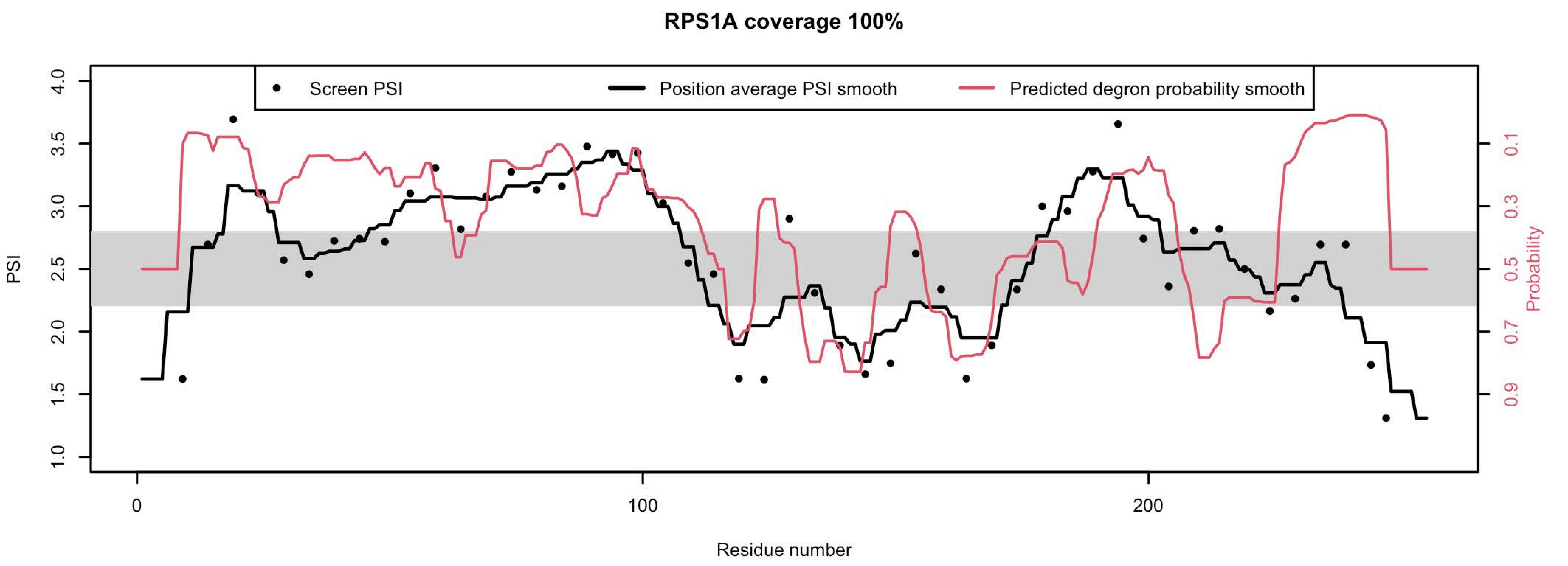

RPS1A coverage 100%
Screen PSI
Position average PSI smooth
Predicted degron probability smooth
PSI
Probability
Residue number
1.0
1.5
2.0
2.5
3.0
3.5
4.0
0.1
0.3
0.5
0.7
0.9
0
100
200

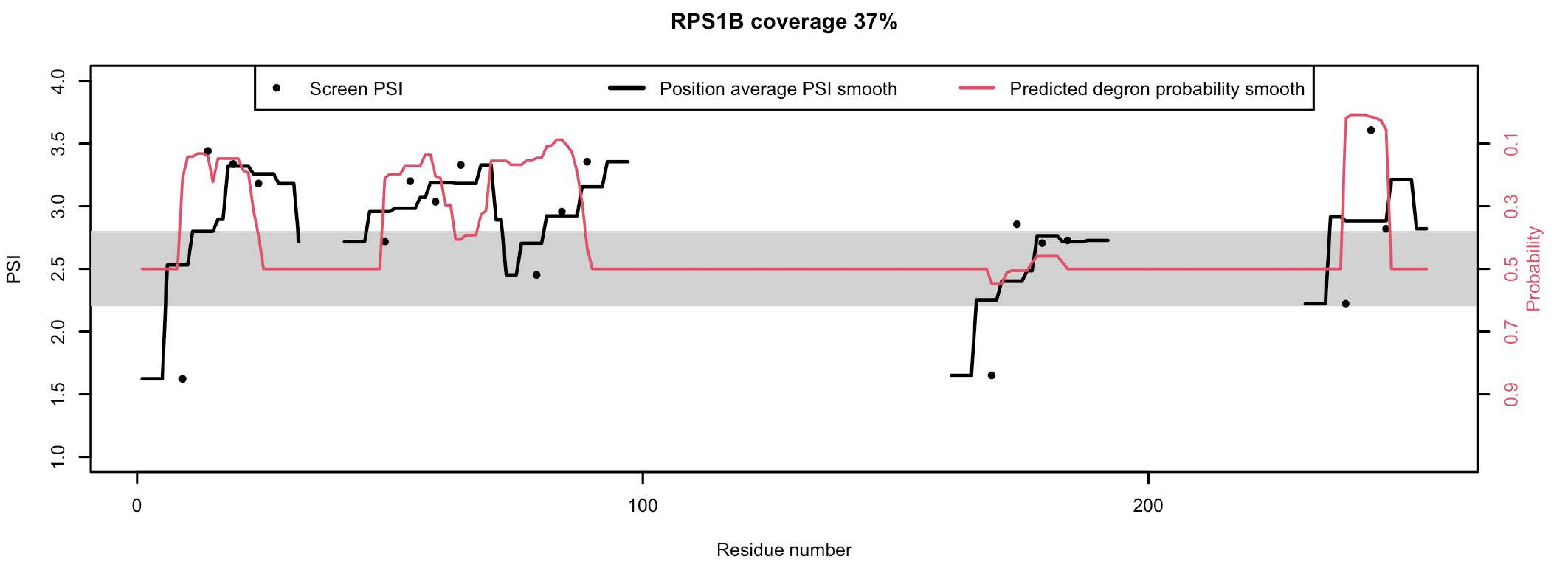

RPS1B coverage 37%
Screen PSI
Position average PSI smooth
Predicted degron probability smooth
PSI
4.0
3.5
3.0
2.5
2.0
1.5
1.0
Probability
0.1
0.3
0.5
0.7
0.9
0
100
200
Residue number

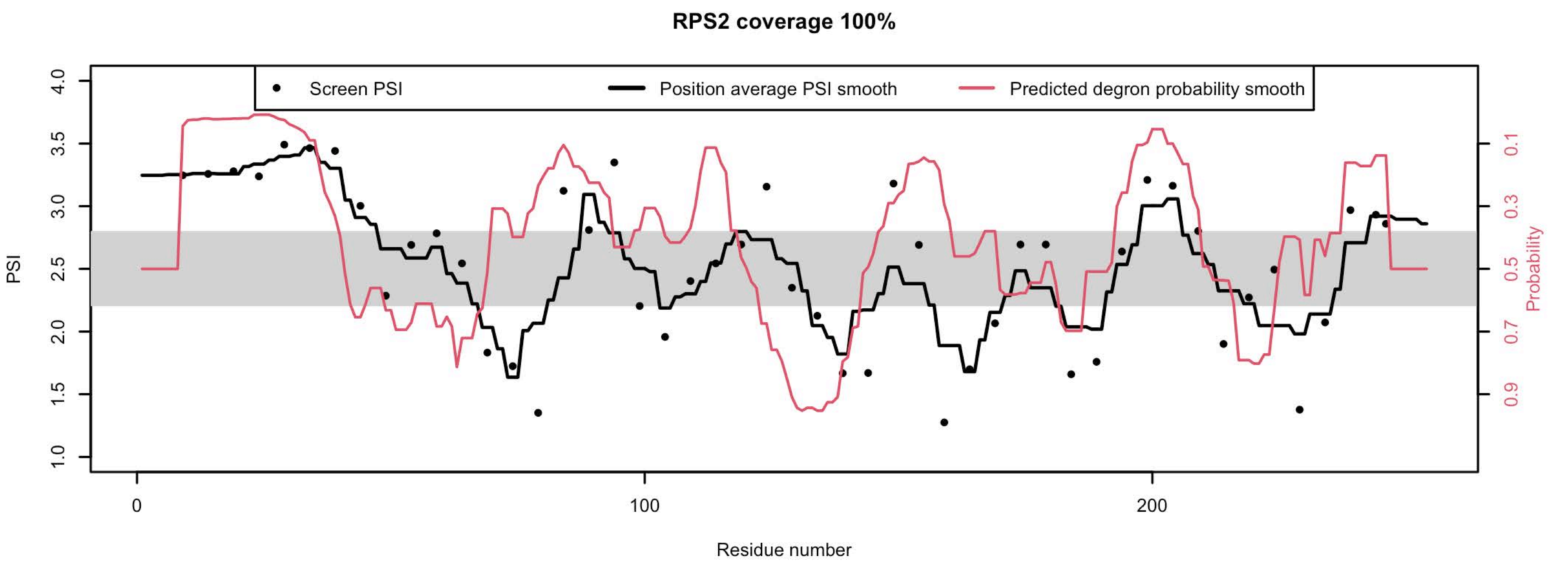

RPS2 coverage 100%
Screen PSI
Position average PSI smooth
Predicted degron probability smooth
PSI
1.0
1.5
2.0
2.5
3.0
3.5
4.0
Probability
0.1
0.3
0.5
0.7
0.9
0
100
200
Residue number

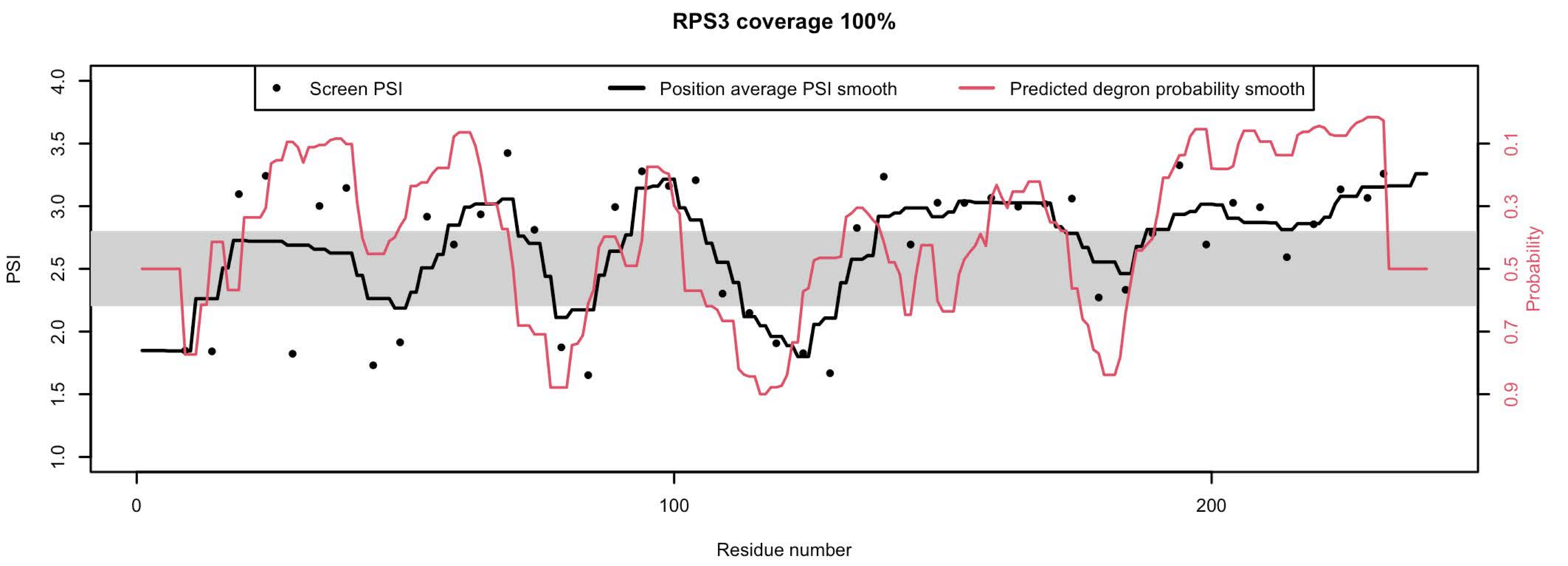

RPS3 coverage 100%
Screen PSI
Position average PSI smooth
Predicted degron probability smooth
PSI
1.0
1.5
2.0
2.5
3.0
3.5
4.0
Probability
0.1
0.3
0.5
0.7
0.9
0
100
200
Residue number

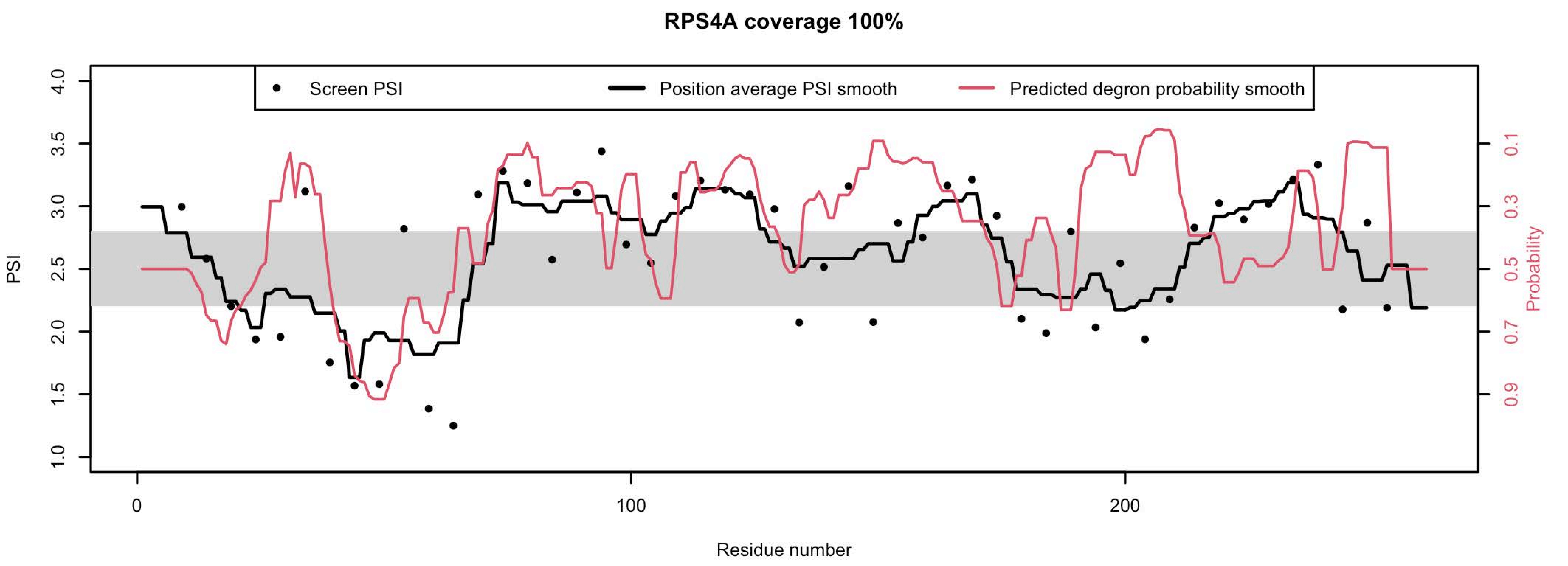

RPS4A coverage 100%
Screen PSI
Position average PSI smooth
Predicted degron probability smooth
PSI
1.0
1.5
2.0
2.5
3.0
3.5
4.0
Probability
0.1
0.3
0.5
0.7
0.9
0
100
200
Residue number

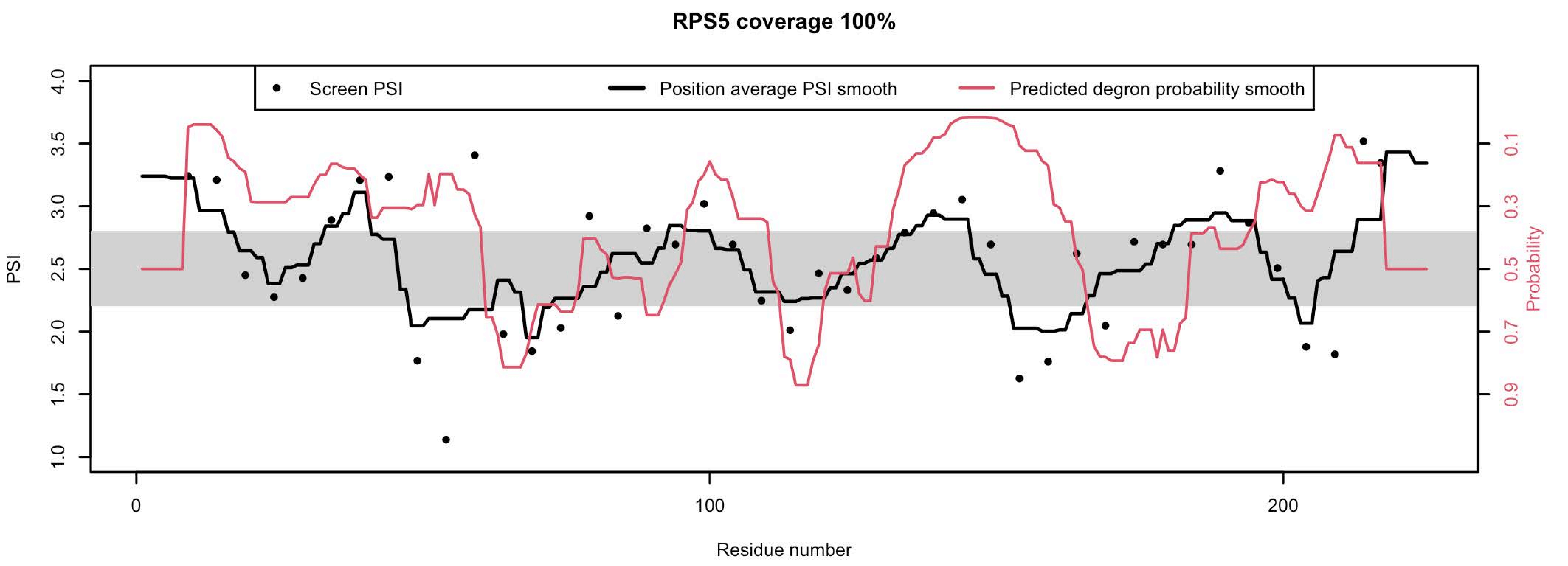

RPS5 coverage 100%
Screen PSI
Position average PSI smooth
Predicted degron probability smooth
PSI
Probability
Residue number
1.0
1.5
2.0
2.5
3.0
3.5
4.0
0.1
0.3
0.5
0.7
0.9
0
100
200

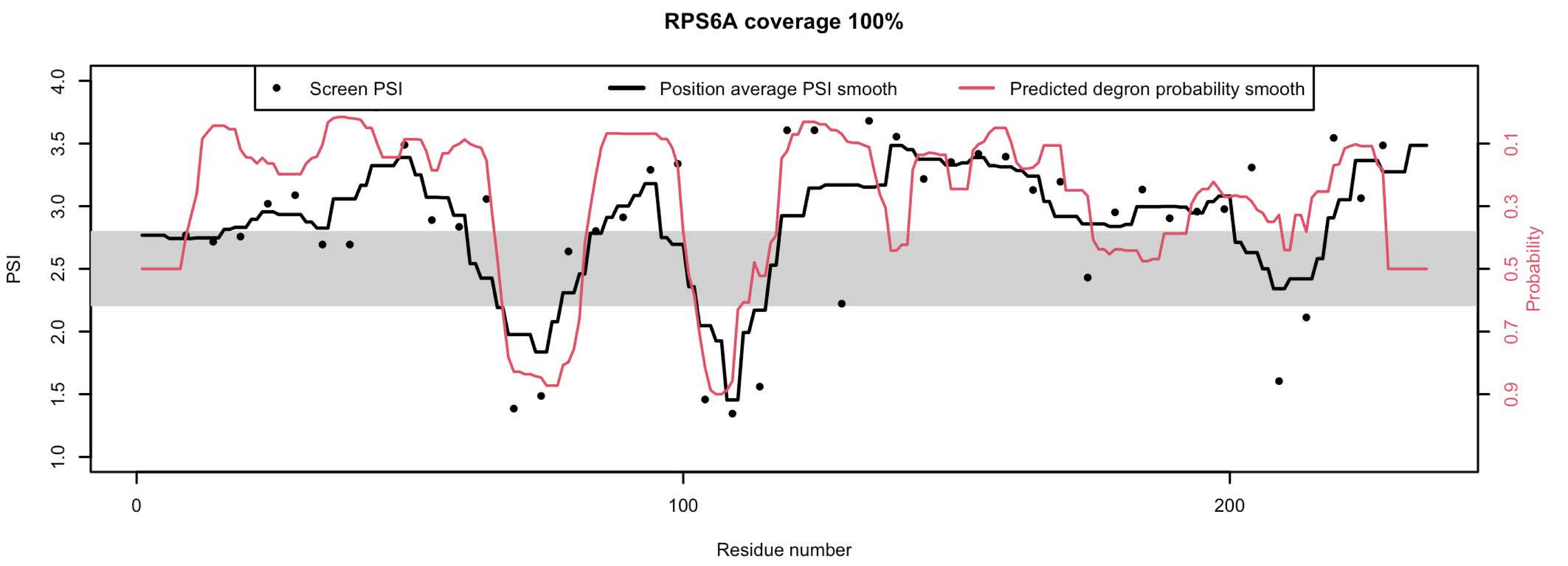
RPS6A coverage 100%
Screen PSI
Position average PSI smooth
Predicted degron probability smooth
PSI
Probability
Residue number
1.0
1.5
2.0
2.5
3.0
3.5
4.0
0.1
0.3
0.5
0.7
0.9
0
100
200

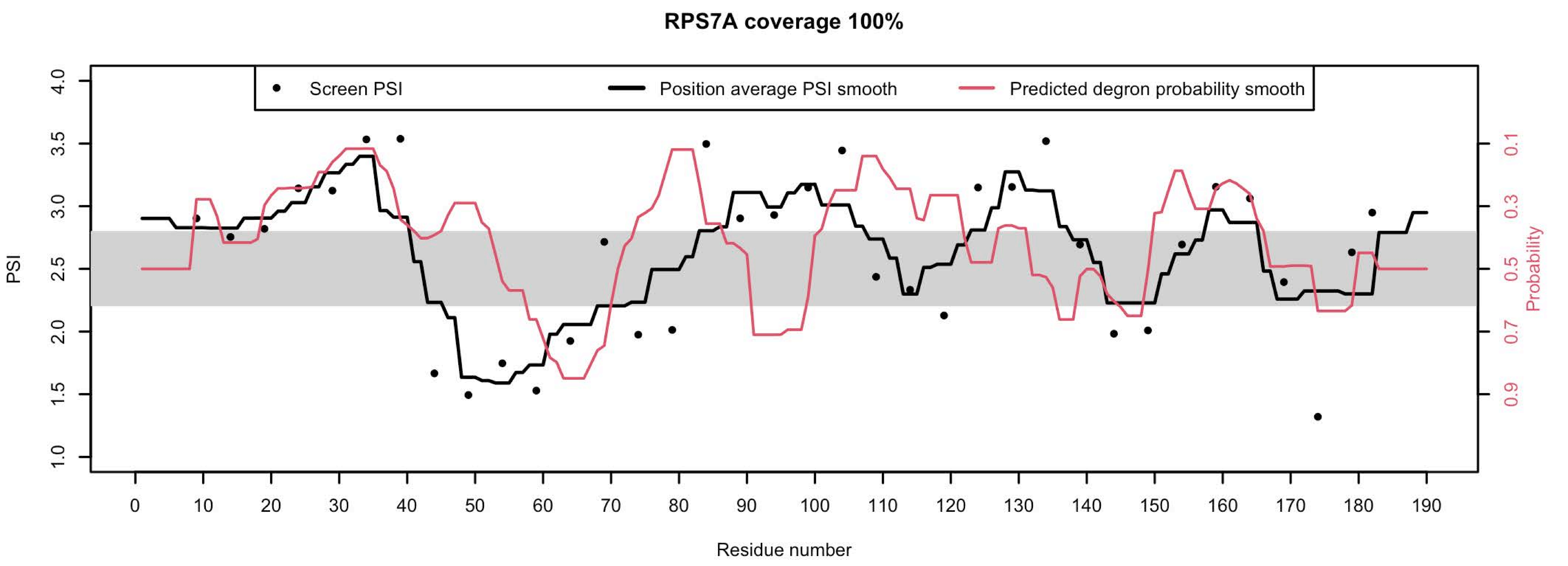

RPS7A coverage 100%
Screen PSI
Position average PSI smooth
Predicted degron probability smooth
PSI
Probability
Residue number
1.0
1.5
2.0
2.5
3.0
3.5
4.0
0.1
0.3
0.5
0.7
0.9
0 10 20 30 40 50 60 70 80 90 100 110 120 130 140 150 160 170 180 190

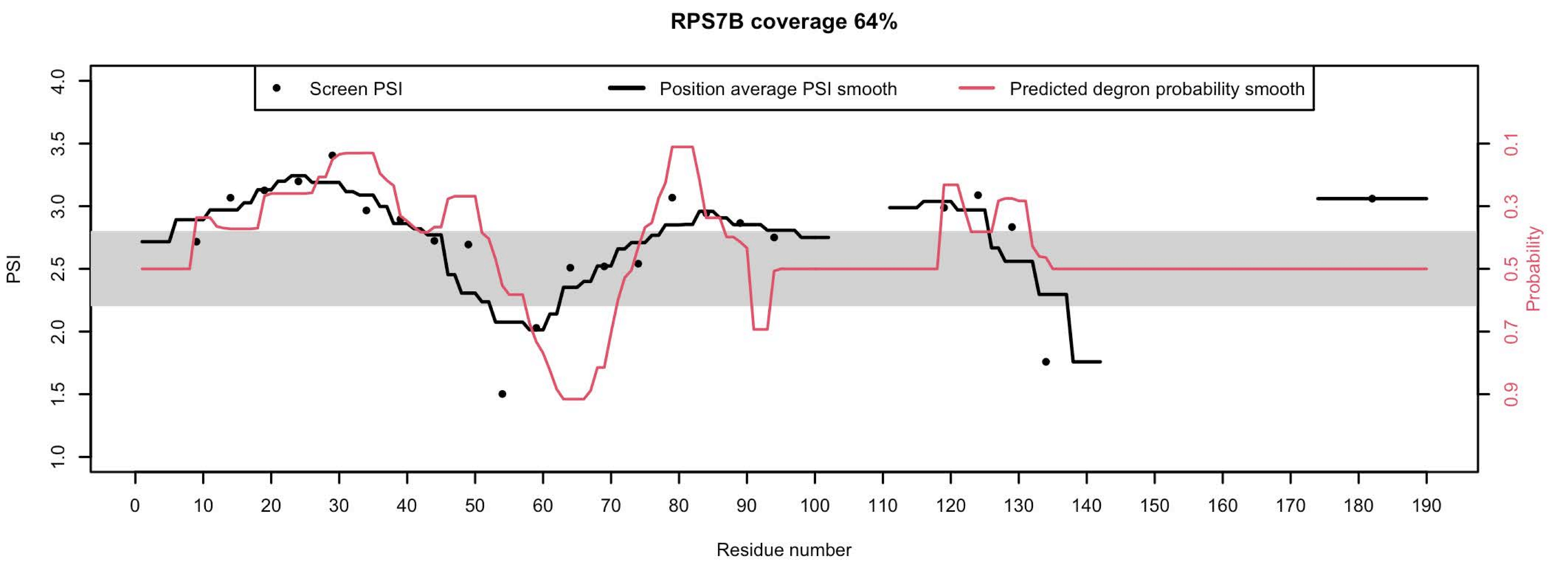
RPS7B coverage 64%
Screen PSI
Position average PSI smooth
Predicted degron probability smooth
PSI
Probability
Residue number
1.0
1.5
2.0
2.5
3.0
3.5
4.0
0.1
0.3
0.5
0.7
0.9
0
10
20
30
40
50
60
70
80
90
100
110
120
130
140
150
160
170
180
190

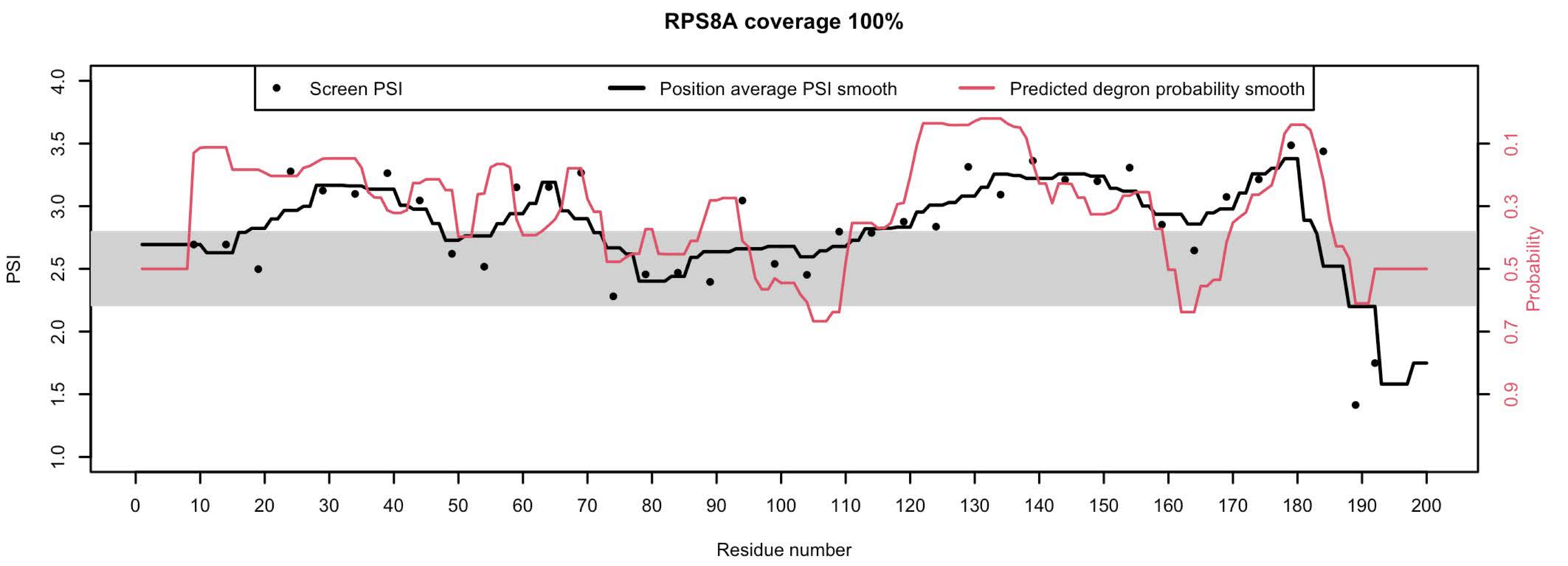

RPS8A coverage 100%
Screen PSI
Position average PSI smooth
Predicted degron probability smooth
PSI
1.0
1.5
2.0
2.5
3.0
3.5
4.0
Probability
0.1
0.3
0.5
0.7
0.9
0
10
20
30
40
50
60
70
80
90
100
110
120
130
140
150
160
170
180
190
200
Residue number

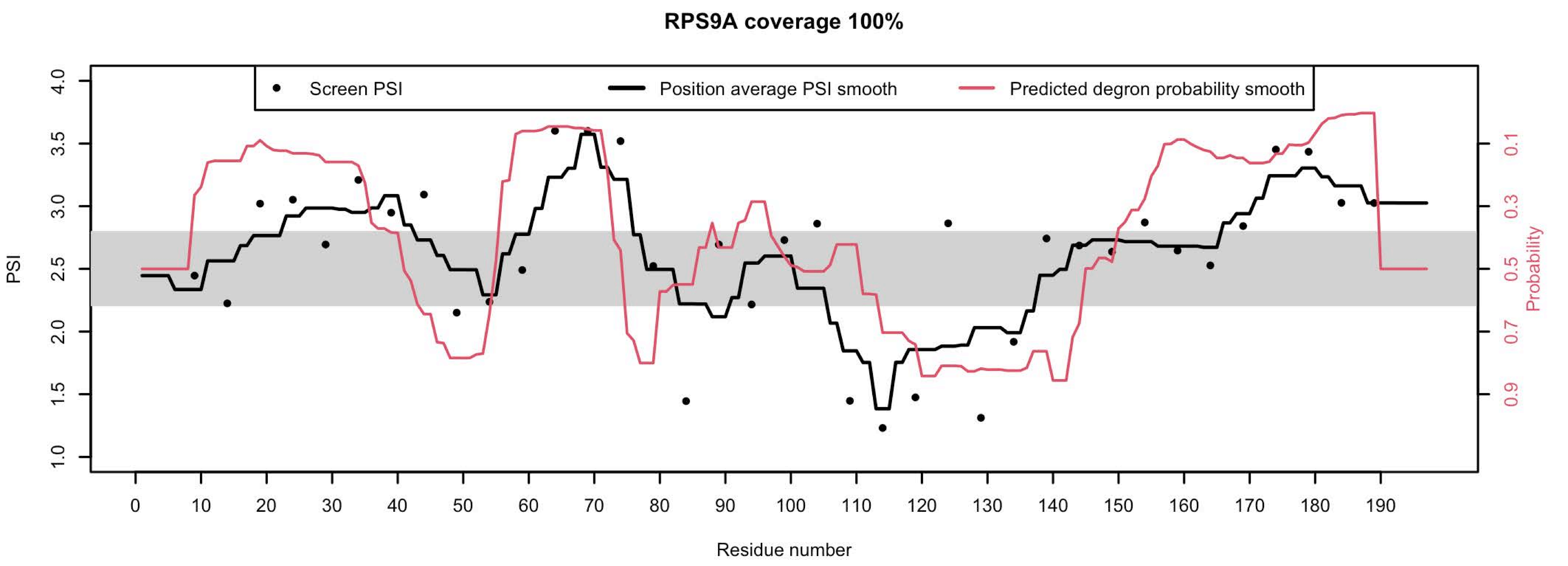
RPS9A coverage 100%
Screen PSI
Position average PSI smooth
Predicted degron probability smooth
PSI
Probability
Residue number
1.0
1.5
2.0
2.5
3.0
3.5
4.0
0.1
0.3
0.5
0.7
0.9
0 10 20 30 40 50 60 70 80 90 100 110 120 130 140 150 160 170 180 190

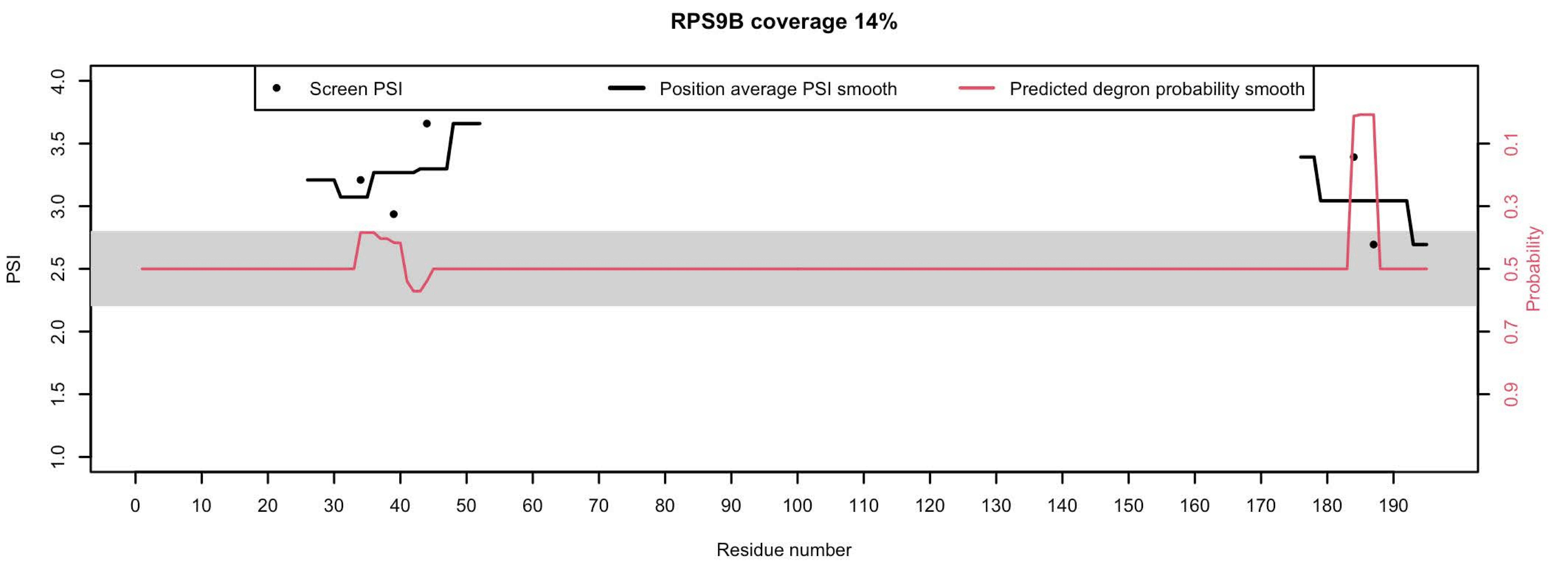
RPS9B coverage 14%
Screen PSI
Position average PSI smooth
Predicted degron probability smooth
PSI
Probability
Residue number
1.0
1.5
2.0
2.5
3.0
3.5
4.0
0.1
0.3
0.5
0.7
0.9
0
10
20
30
40
50
60
70
80
90
100
110
120
130
140
150
160
170
180
190

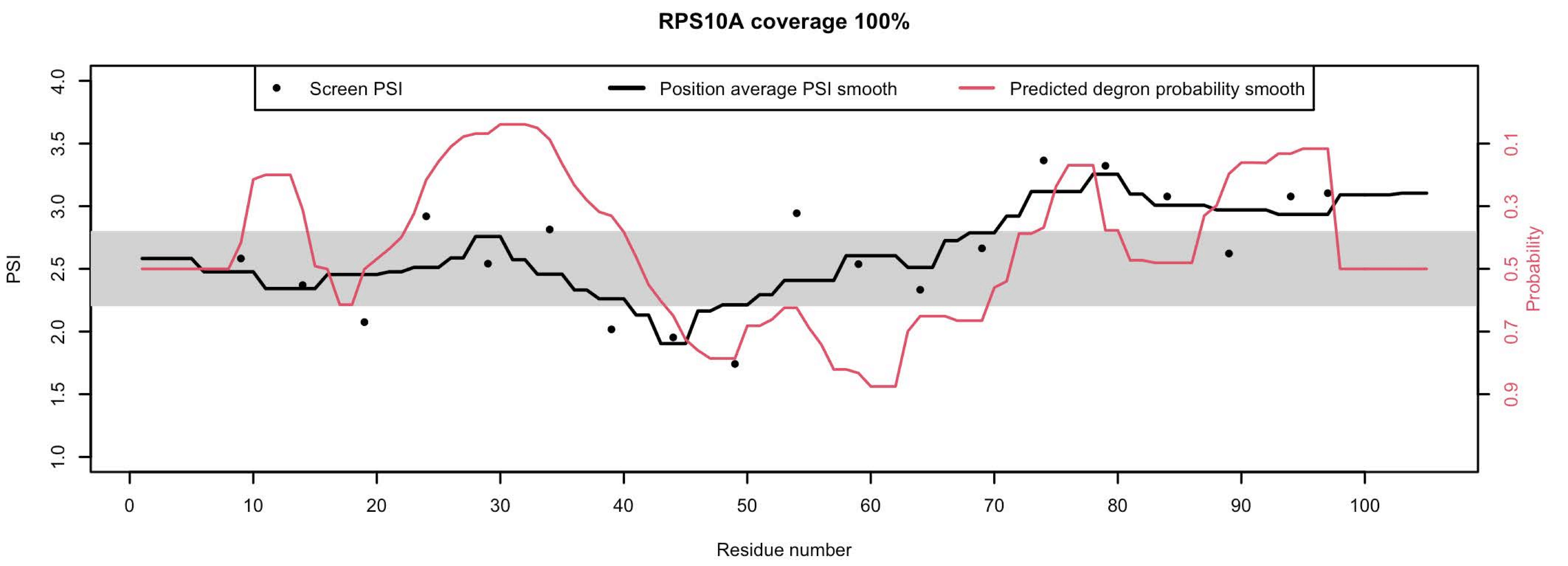

RPS10A coverage 100%
Screen PSI
Position average PSI smooth
Predicted degron probability smooth
PSI
1.0
1.5
2.0
2.5
3.0
3.5
4.0
Probability
0.1
0.3
0.5
0.7
0.9
0
10
20
30
40
50
60
70
80
90
100
Residue number

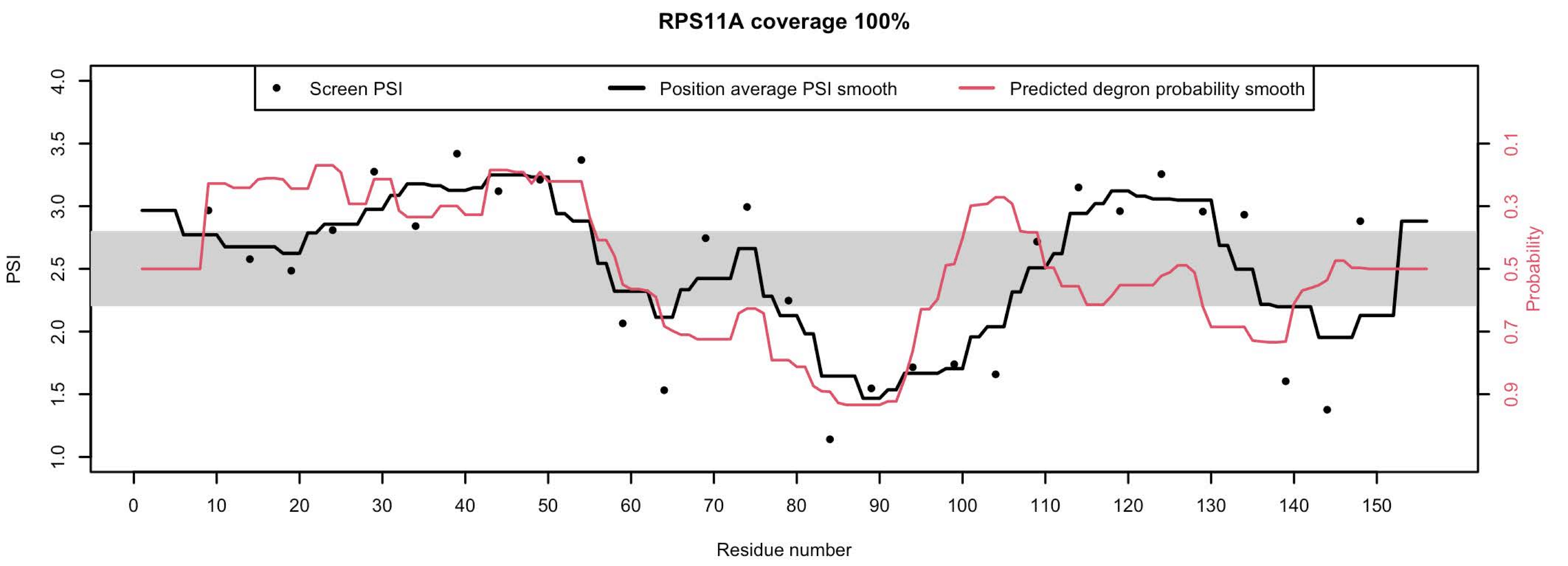

RPS11A coverage 100%
Screen PSI
Position average PSI smooth
Predicted degron probability smooth
PSI
Probability
Residue number
1.0
1.5
2.0
2.5
3.0
3.5
4.0
0.1
0.3
0.5
0.7
0.9
0
10
20
30
40
50
60
70
80
90
100
110
120
130
140
150

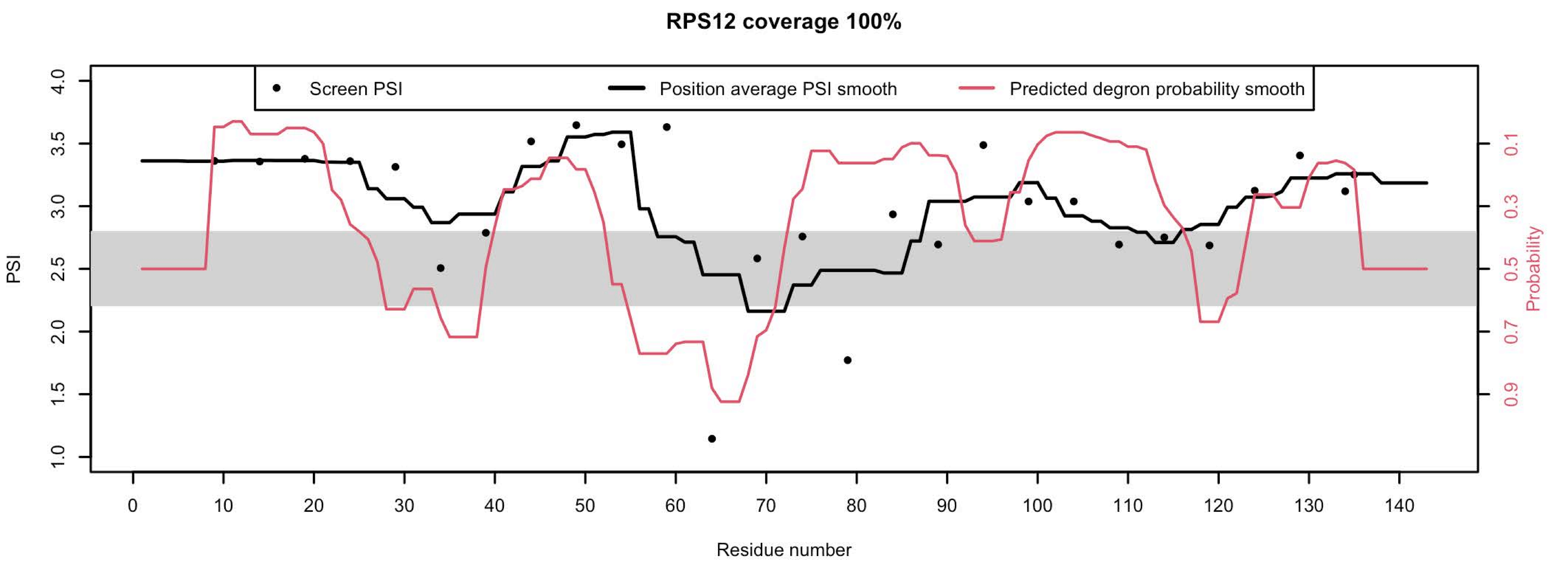
RPS12 coverage 100%
Screen PSI
Position average PSI smooth
Predicted degron probability smooth
PSI
4.0
3.5
3.0
2.5
2.0
1.5
1.0
Probability
0.1
0.3
0.5
0.7
0.9
0
10
20
30
40
50
60
70
80
90
100
110
120
130
140
Residue number

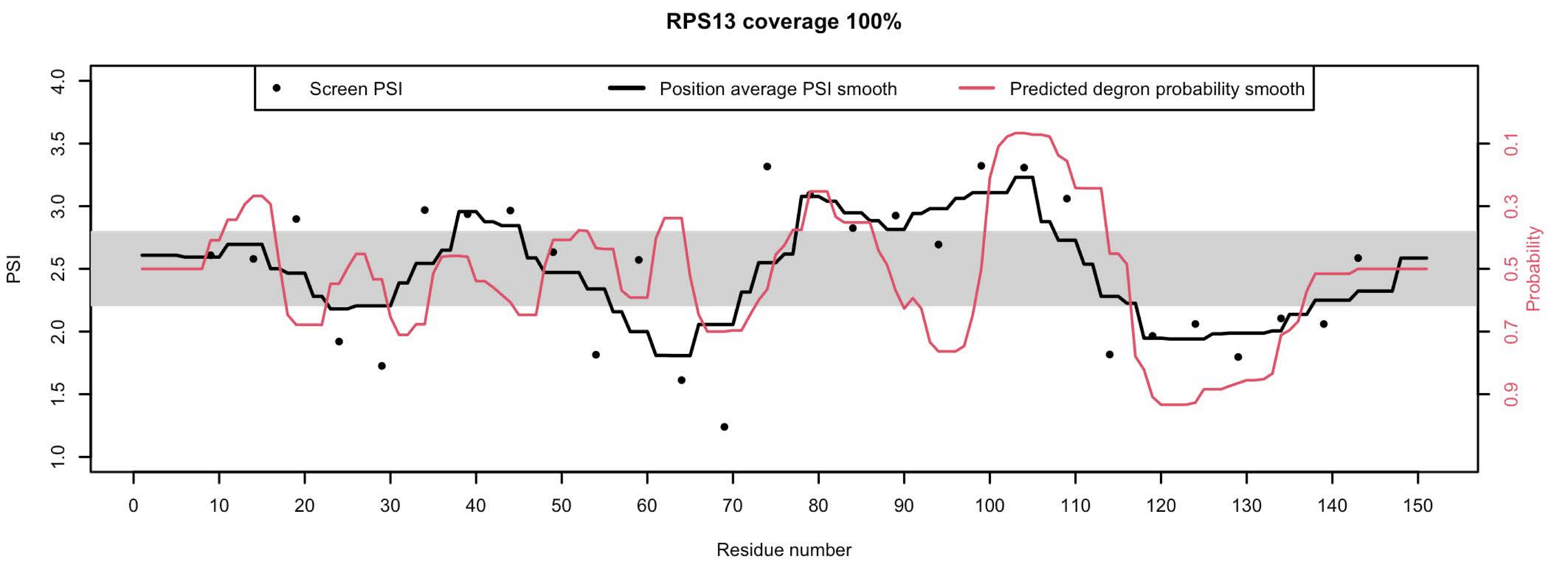

RPS13 coverage 100%
Screen PSI
Position average PSI smooth
Predicted degron probability smooth
PSI
1.0
1.5
2.0
2.5
3.0
3.5
4.0
Probability
0.1
0.3
0.5
0.7
0.9
Residue number
0
10
20
30
40
50
60
70
80
90
100
110
120
130
140
150

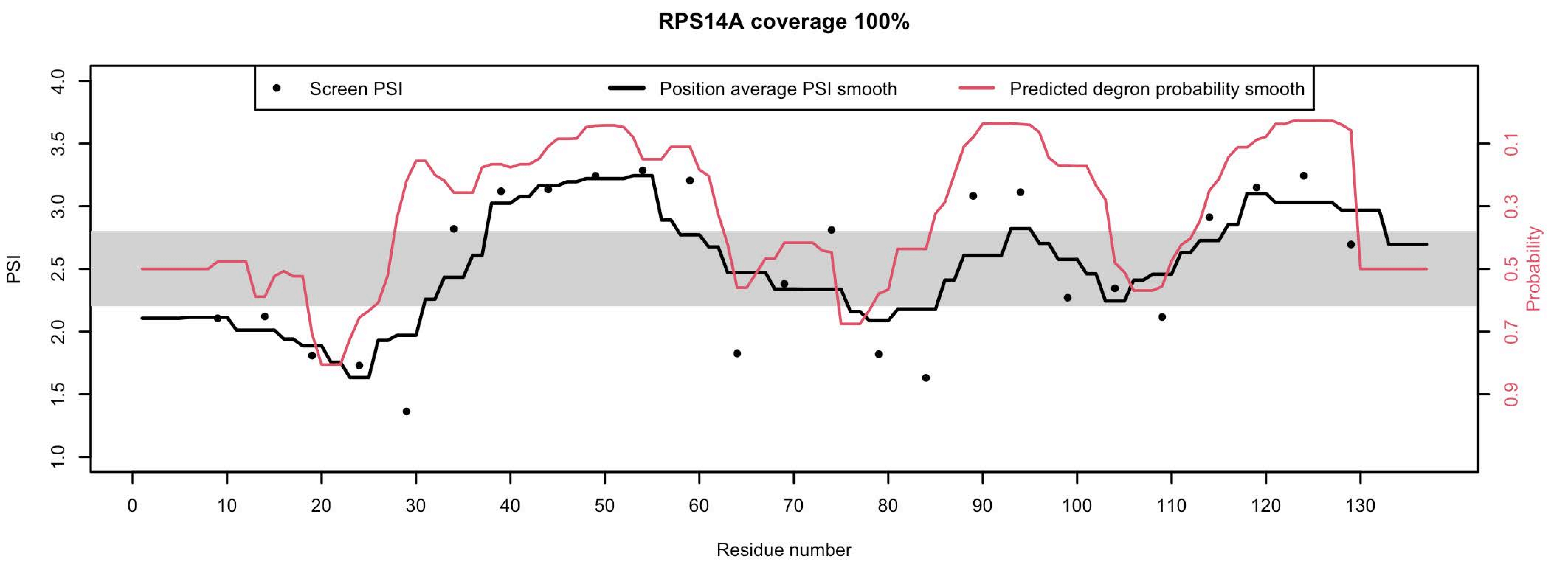

RPS14A coverage 100%
Screen PSI
Position average PSI smooth
Predicted degron probability smooth
PSI
Probability
Residue number
1.0
1.5
2.0
2.5
3.0
3.5
4.0
0.1
0.3
0.5
0.7
0.9
0
10
20
30
40
50
60
70
80
90
100
110
120
130

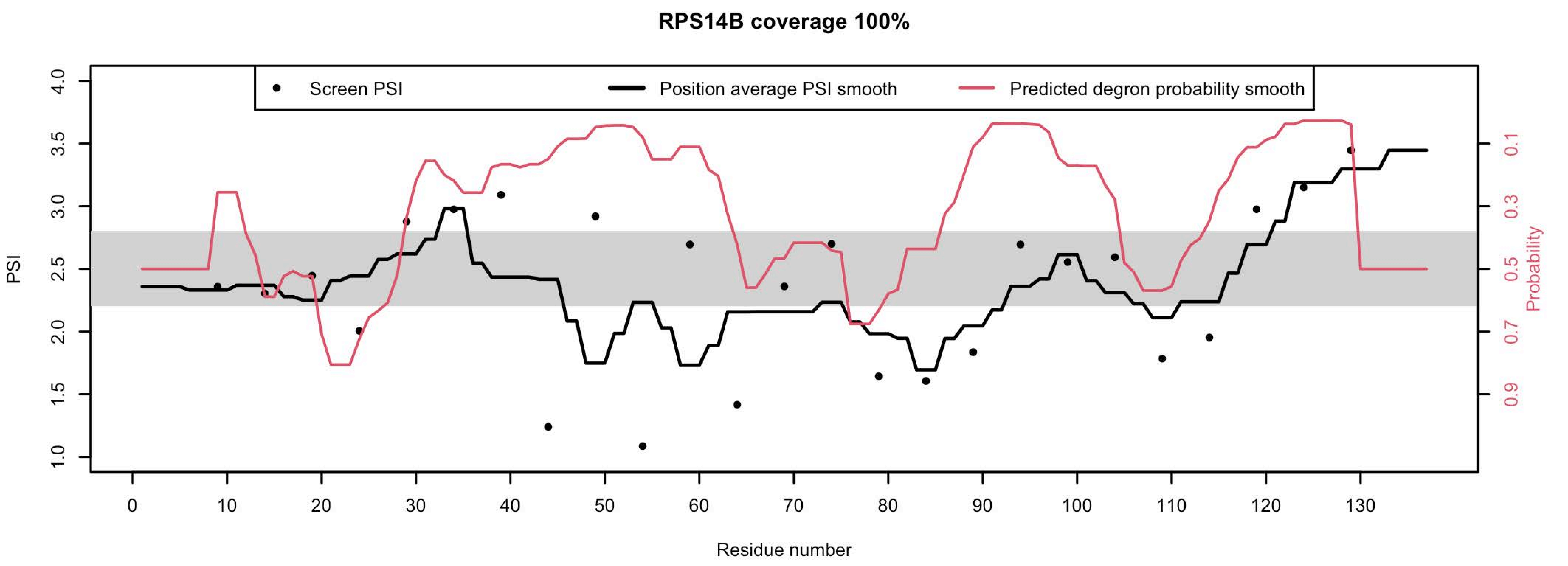

RPS14B coverage 100%
Screen PSI
Position average PSI smooth
Predicted degron probability smooth
PSI
1.0
1.5
2.0
2.5
3.0
3.5
4.0
Probability
0.1
0.3
0.5
0.7
0.9
0
10
20
30
40
50
60
70
80
90
100
110
120
130
Residue number

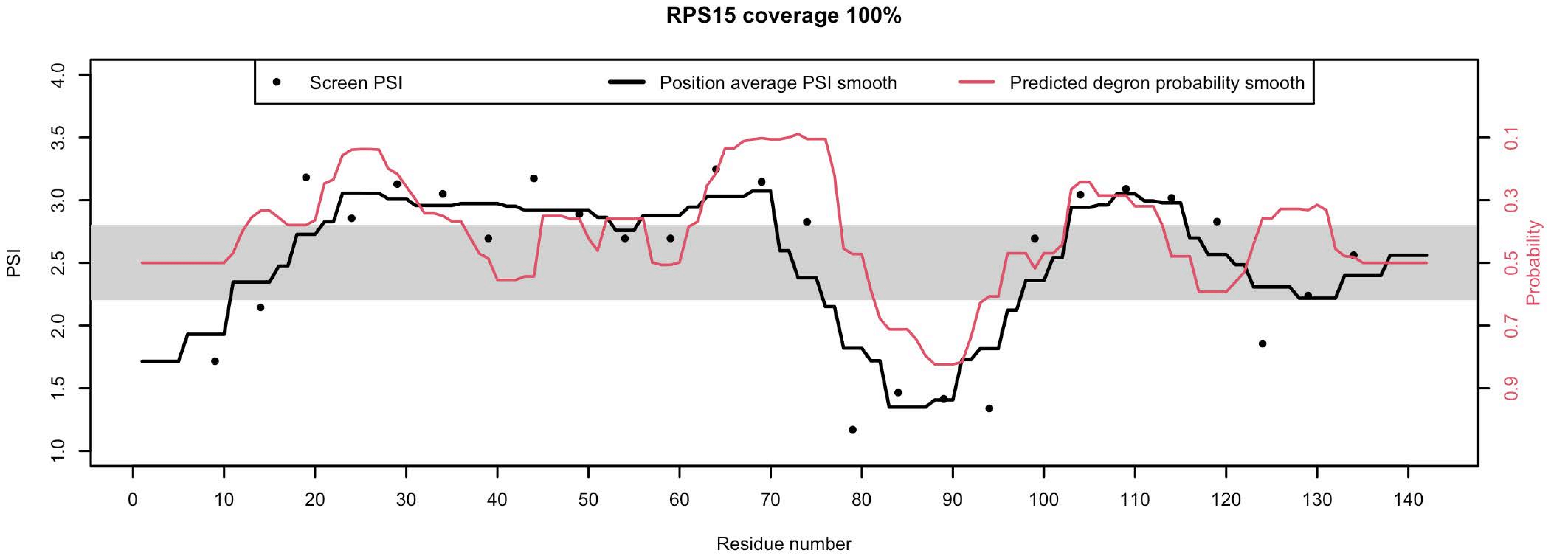

RPS15 coverage 100%
Screen PSI
Position average PSI smooth
Predicted degron probability smooth
PSI
Probability
Residue number
1.0
1.5
2.0
2.5
3.0
3.5
4.0
0.1
0.3
0.5
0.7
0.9
0
10
20
30
40
50
60
70
80
90
100
110
120
130
140

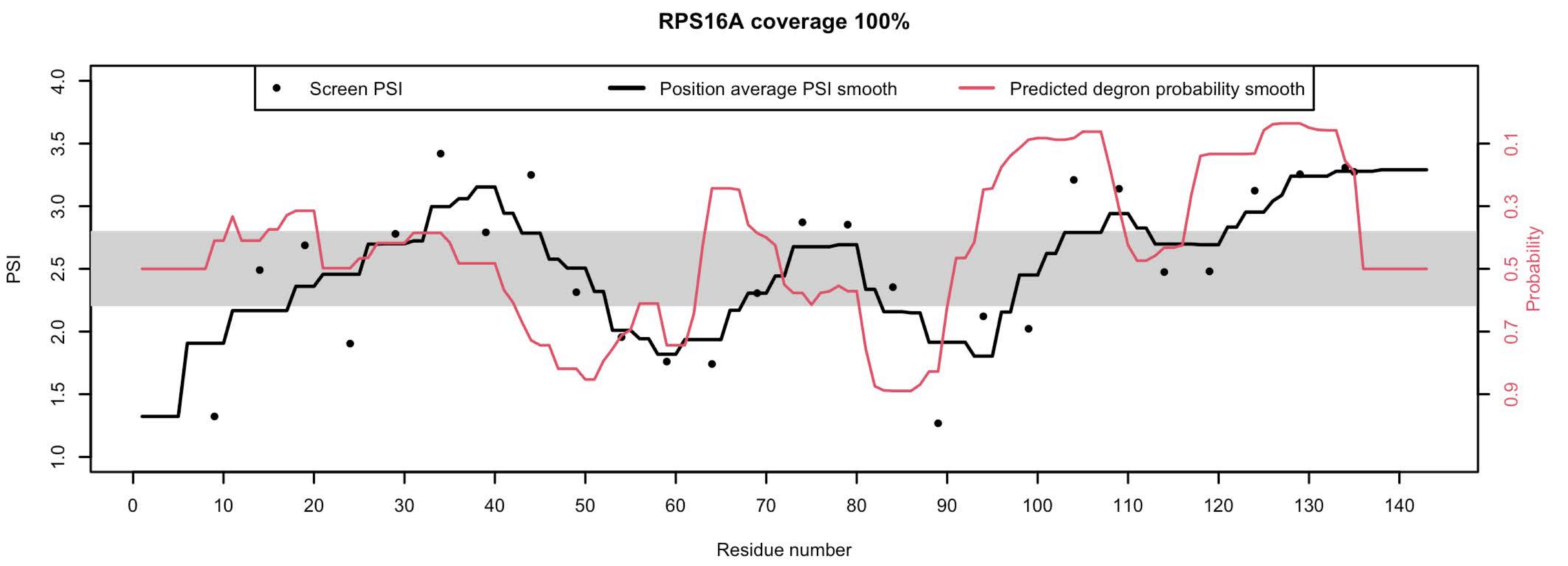

RPS16A coverage 100%
Screen PSI
Position average PSI smooth
Predicted degron probability smooth
PSI
Probability
Residue number
1.0
1.5
2.0
2.5
3.0
3.5
4.0
0.1
0.3
0.5
0.7
0.9
0
10
20
30
40
50
60
70
80
90
100
110
120
130
140

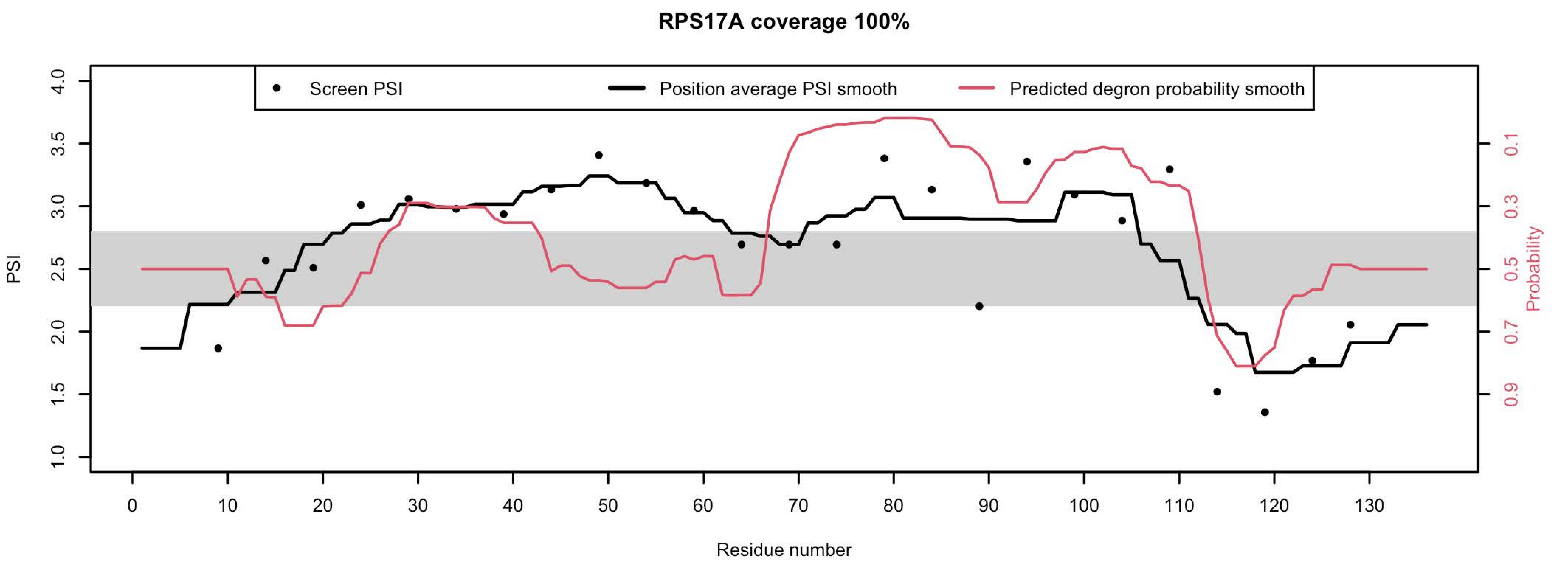

RPS17A coverage 100%
Screen PSI
Position average PSI smooth
Predicted degron probability smooth
PSI
1.0
1.5
2.0
2.5
3.0
3.5
4.0
Probability
0.1
0.3
0.5
0.7
0.9
Residue number
0
10
20
30
40
50
60
70
80
90
100
110
120
130

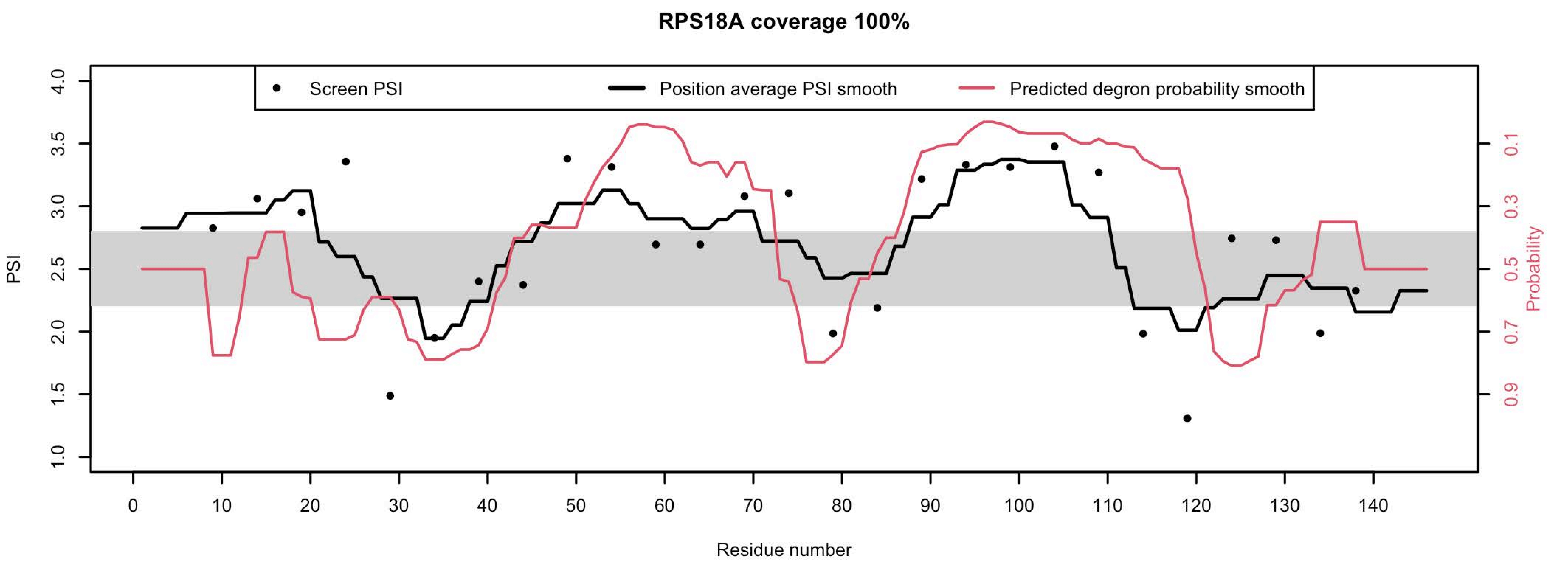
RPS18A coverage 100%
Screen PSI
Position average PSI smooth
Predicted degron probability smooth
PSI
Probability
Residue number
1.0
1.5
2.0
2.5
3.0
3.5
4.0
0.1
0.3
0.5
0.7
0.9
0
10
20
30
40
50
60
70
80
90
100
110
120
130
140

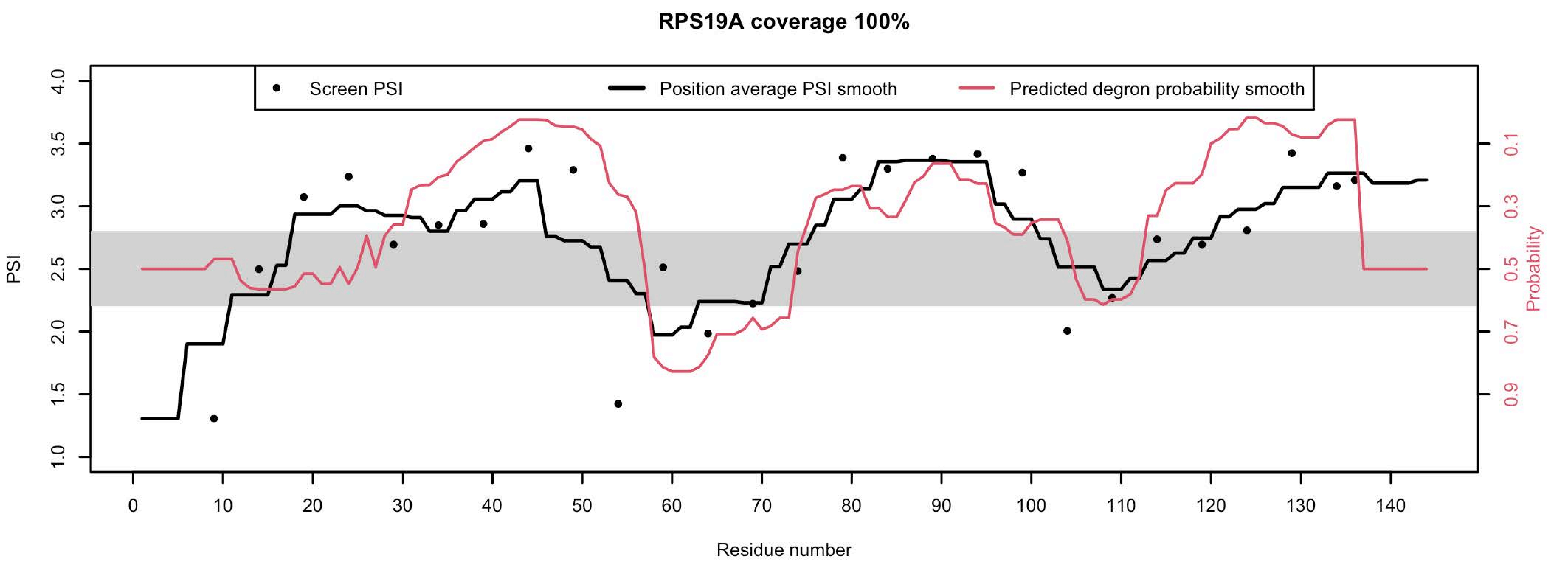
RPS19A coverage 100%
Screen PSI
Position average PSI smooth
Predicted degron probability smooth
PSI
Probability
Residue number
1.0
1.5
2.0
2.5
3.0
3.5
4.0
0.1
0.3
0.5
0.7
0.9
0
10
20
30
40
50
60
70
80
90
100
110
120
130
140

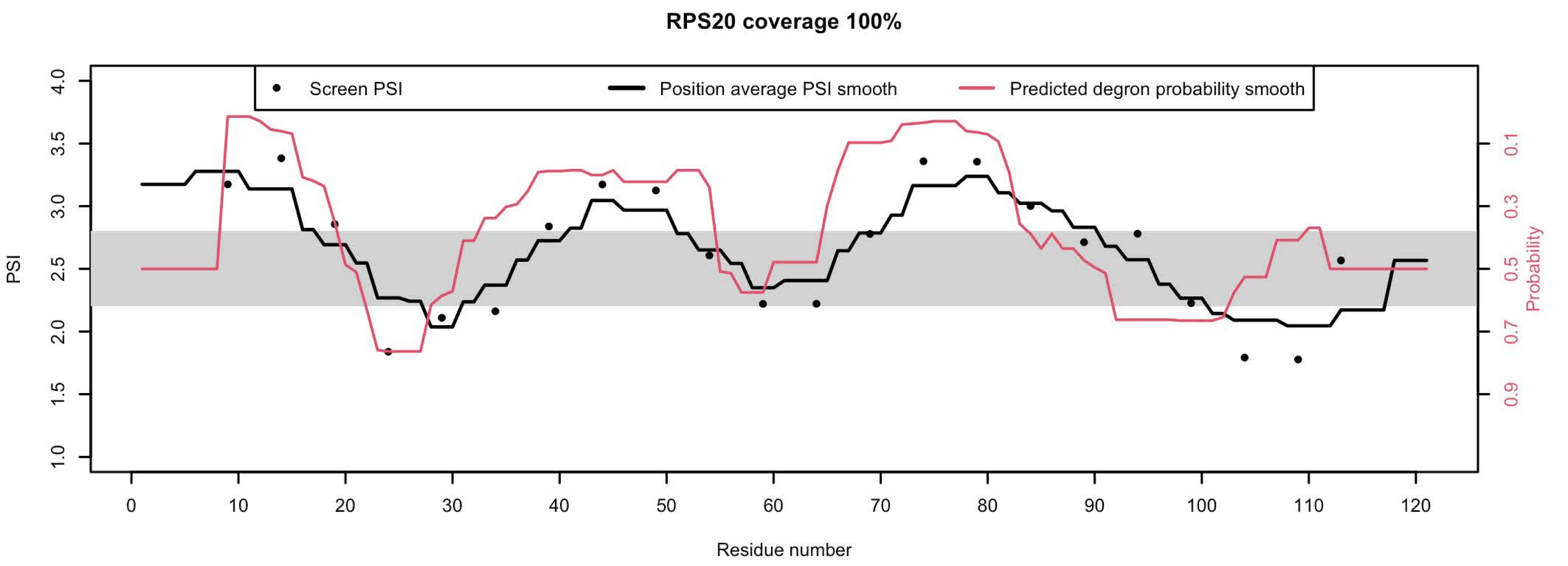

RPS20 coverage 100%
Screen PSI
Position average PSI smooth
Predicted degron probability smooth
PSI
Probability
Residue number
1.0
1.5
2.0
2.5
3.0
3.5
4.0
0.1
0.3
0.5
0.7
0.9
0
10
20
30
40
50
60
70
80
90
100
110
120

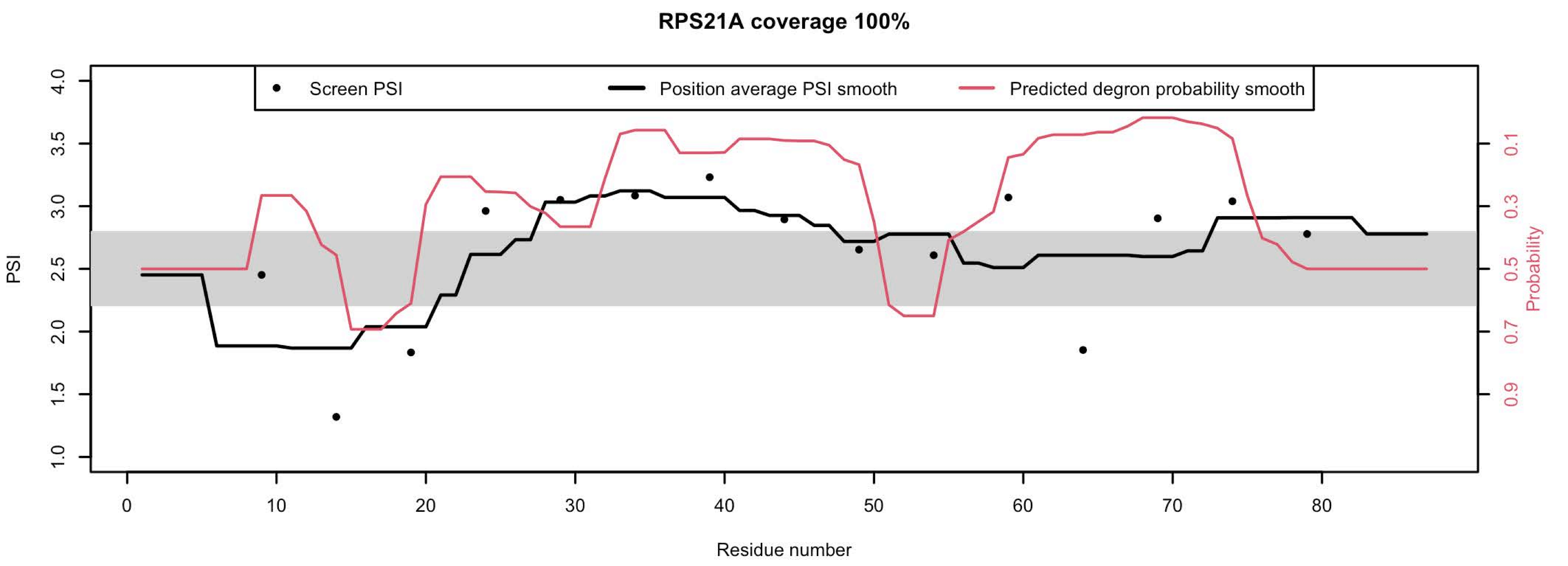
RPS21A coverage 100%
Screen PSI
Position average PSI smooth
Predicted degron probability smooth
PSI
Probability
Residue number
1.0
1.5
2.0
2.5
3.0
3.5
4.0
0.1
0.3
0.5
0.7
0.9
0
10
20
30
40
50
60
70
80

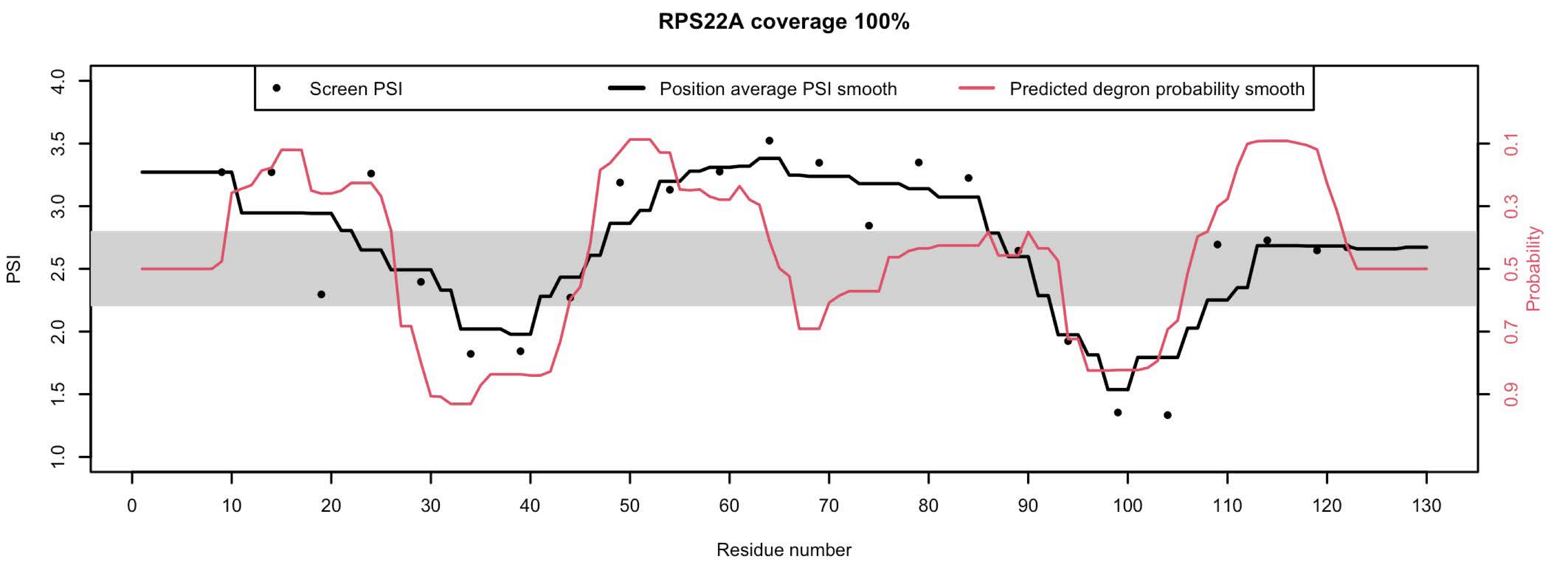
RPS22A coverage 100%
Screen PSI
Position average PSI smooth
Predicted degron probability smooth
PSI
1.0
1.5
2.0
2.5
3.0
3.5
4.0
Probability
0.1
0.3
0.5
0.7
0.9
0
10
20
30
40
50
60
70
80
90
100
110
120
130
Residue number

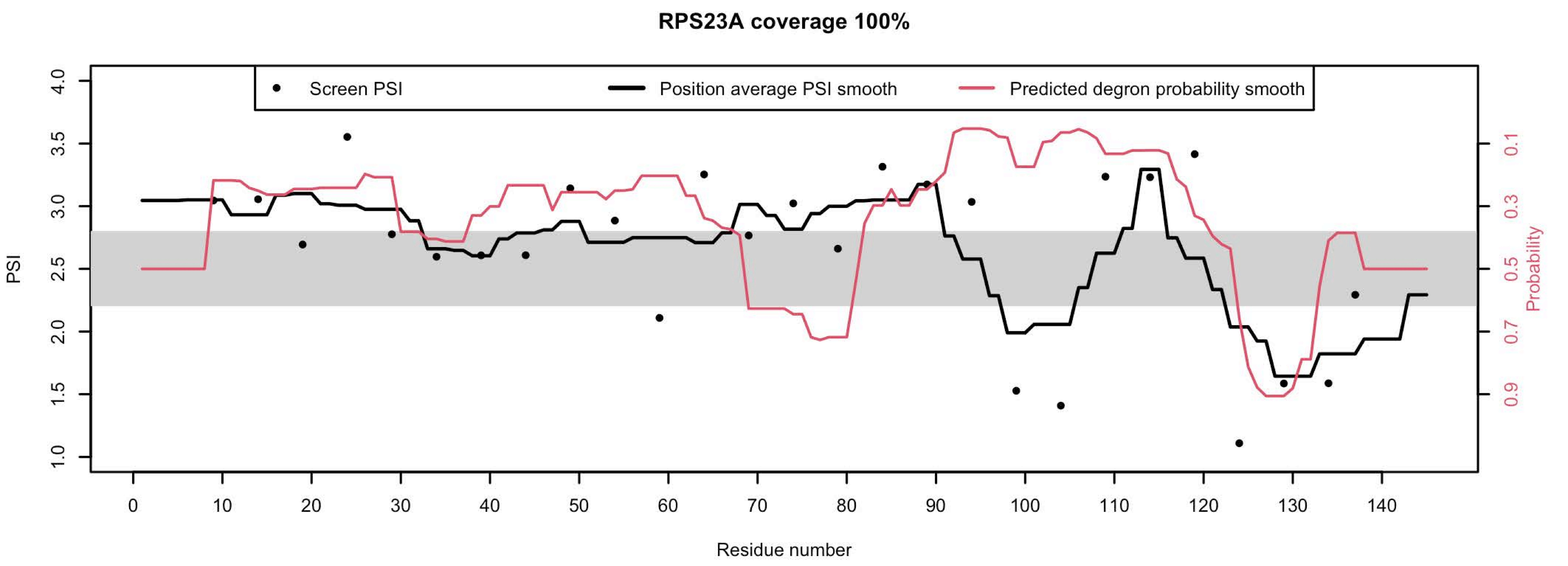

RPS23A coverage 100%
Screen PSI
Position average PSI smooth
Predicted degron probability smooth
PSI
4.0
3.5
3.0
2.5
2.0
1.5
1.0
Probability
0.1
0.3
0.5
0.7
0.9
0
10
20
30
40
50
60
70
80
90
100
110
120
130
140
Residue number

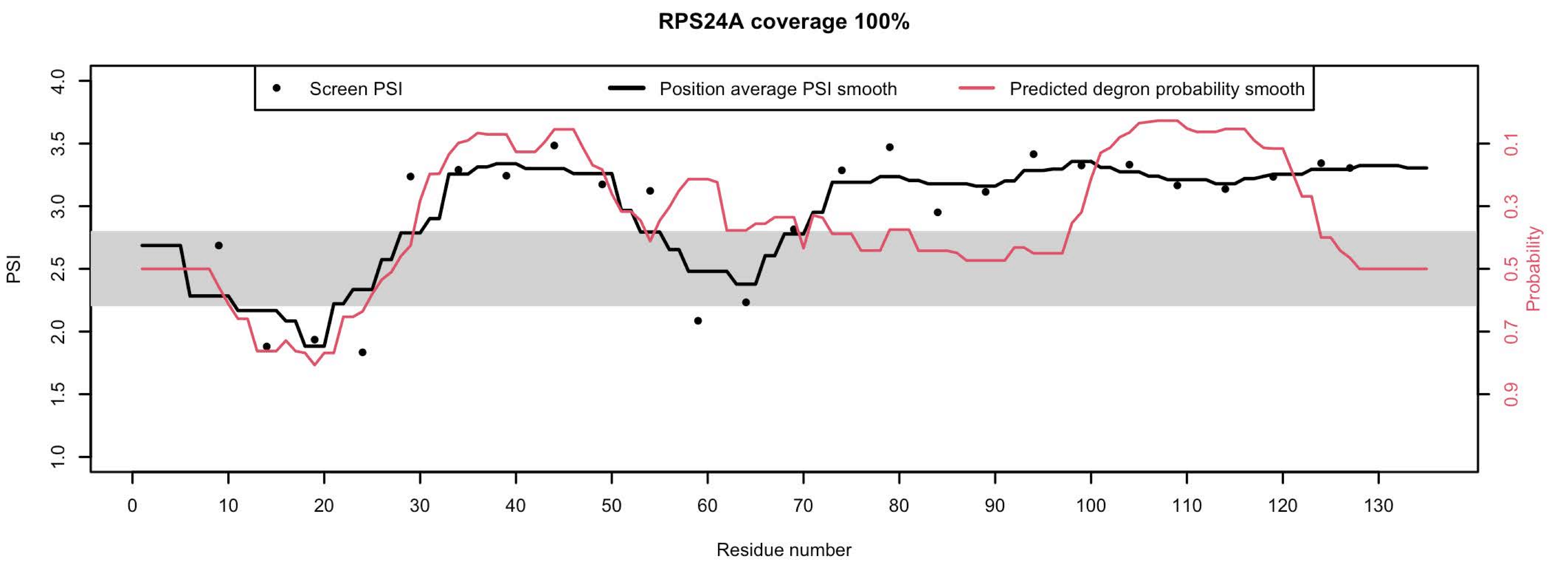

RPS24A coverage 100%
Screen PSI
Position average PSI smooth
Predicted degron probability smooth
PSI
1.0
1.5
2.0
2.5
3.0
3.5
4.0
Probability
0.1
0.3
0.5
0.7
0.9
0
10
20
30
40
50
60
70
80
90
100
110
120
130
Residue number

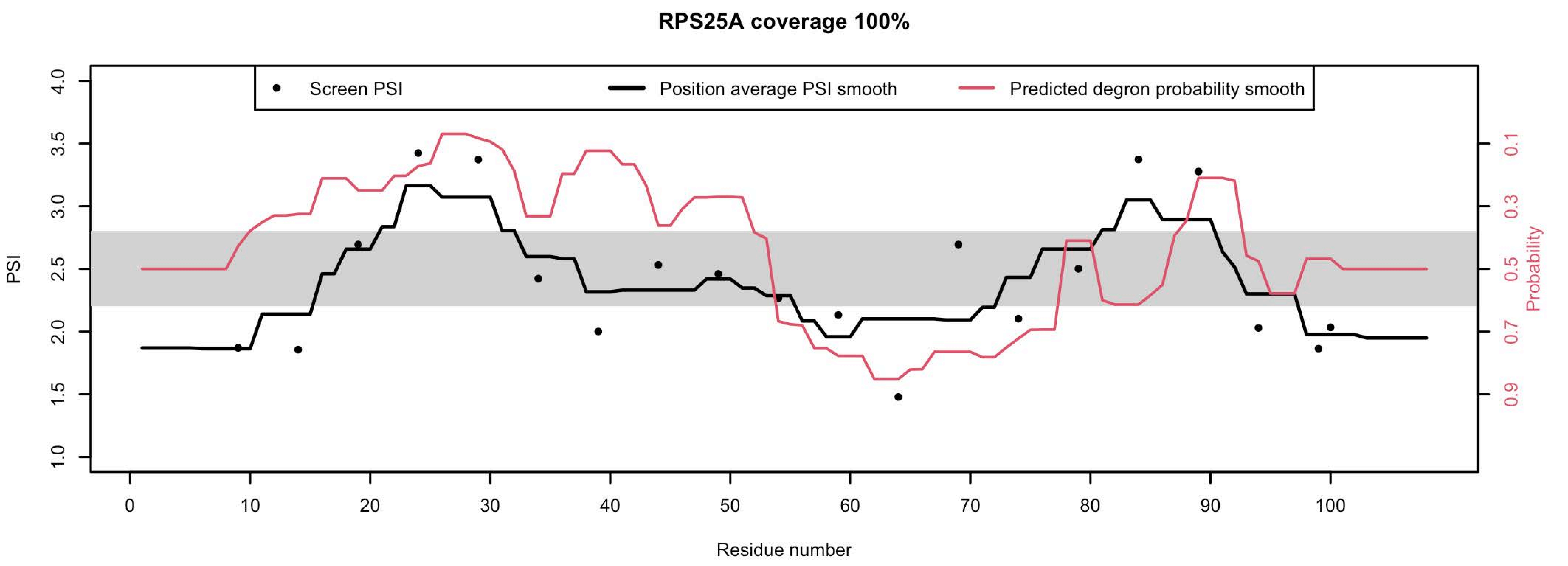

RPS25A coverage 100%
Screen PSI
Position average PSI smooth
Predicted degron probability smooth
PSI
Probability
Residue number
1.0
1.5
2.0
2.5
3.0
3.5
4.0
0.1
0.3
0.5
0.7
0.9
0
10
20
30
40
50
60
70
80
90
100

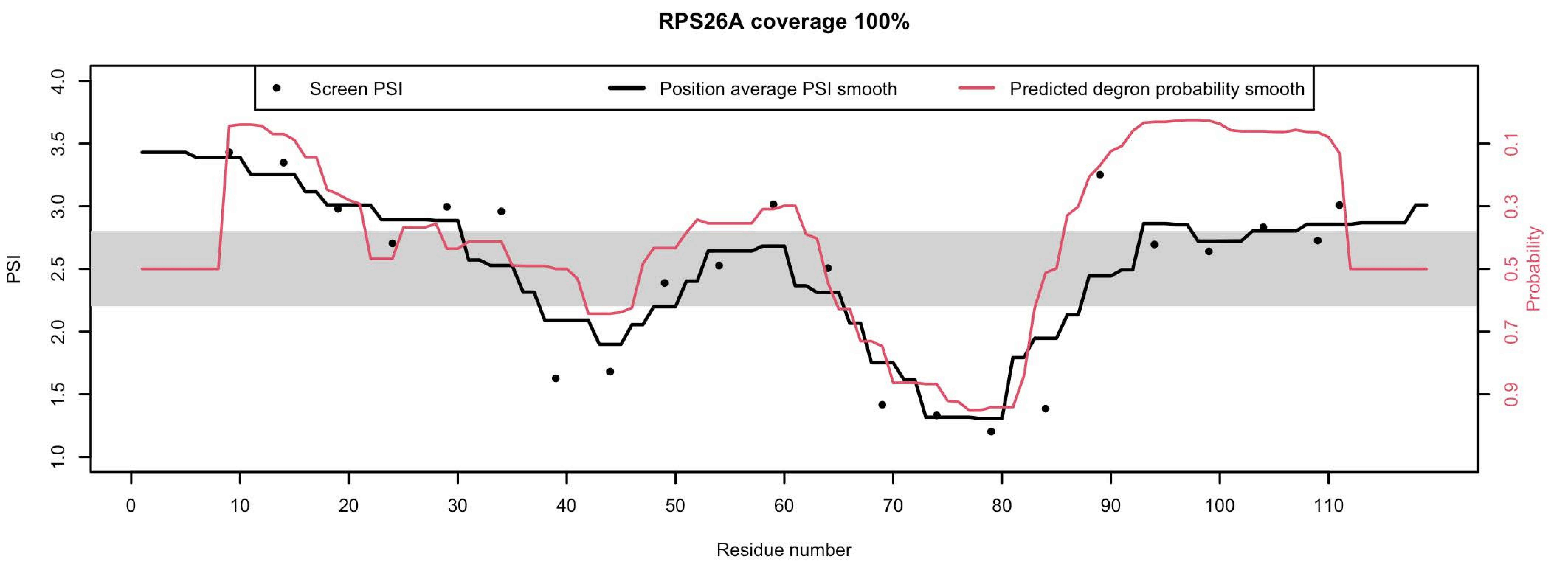

RPS26A coverage 100%
Screen PSI
Position average PSI smooth
Predicted degron probability smooth
PSI
Probability
Residue number
1.0
1.5
2.0
2.5
3.0
3.5
4.0
0.1
0.3
0.5
0.7
0.9
0
10
20
30
40
50
60
70
80
90
100
110

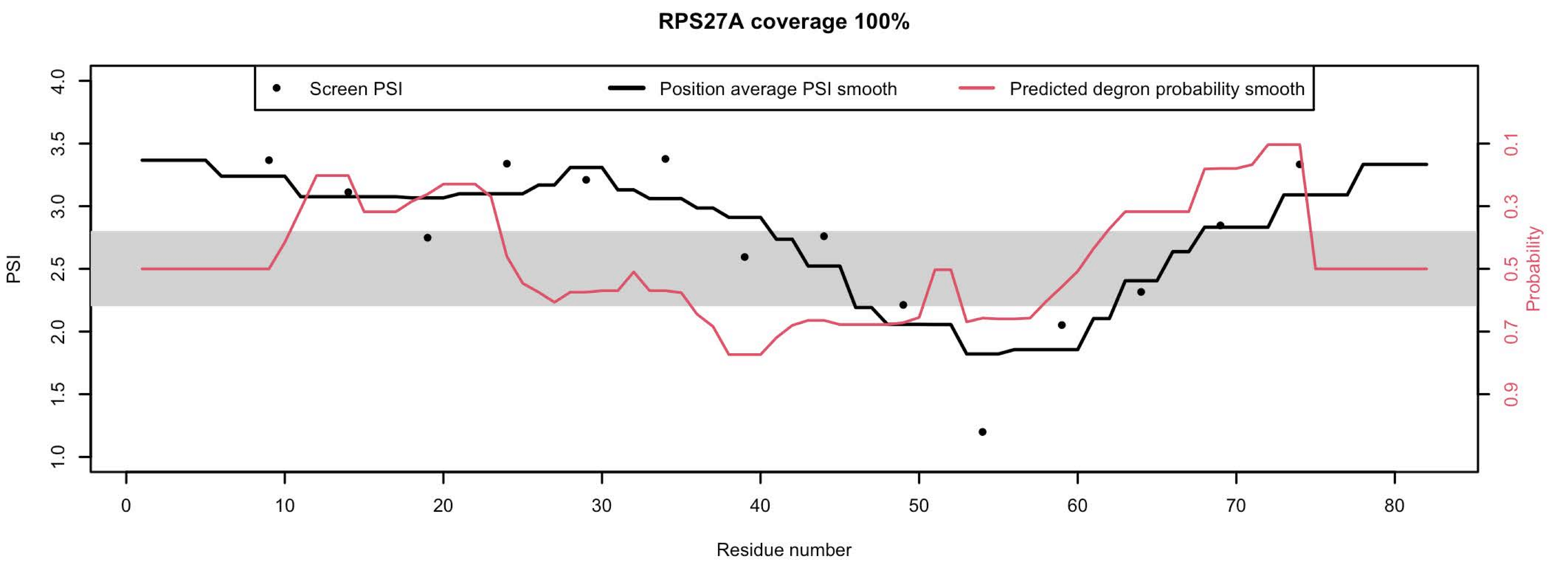
RPS27A coverage 100%
Screen PSI
Position average PSI smooth
Predicted degron probability smooth
PSI
1.0
1.5
2.0
2.5
3.0
3.5
4.0
Probability
0.1
0.3
0.5
0.7
0.9
Residue number
0
10
20
30
40
50
60
70
80

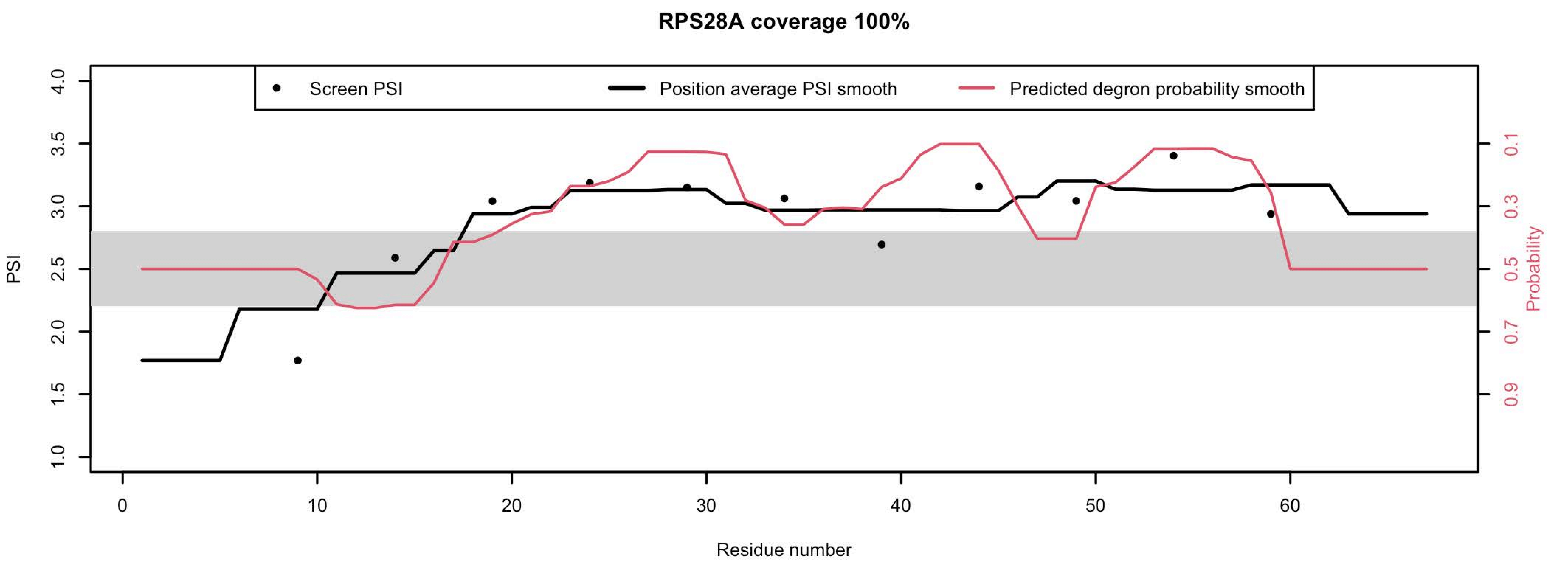

RPS28A coverage 100%
Screen PSI
Position average PSI smooth
Predicted degron probability smooth
PSI
1.0
1.5
2.0
2.5
3.0
3.5
4.0
Probability
0.1
0.3
0.5
0.7
0.9
Residue number
0
10
20
30
40
50
60

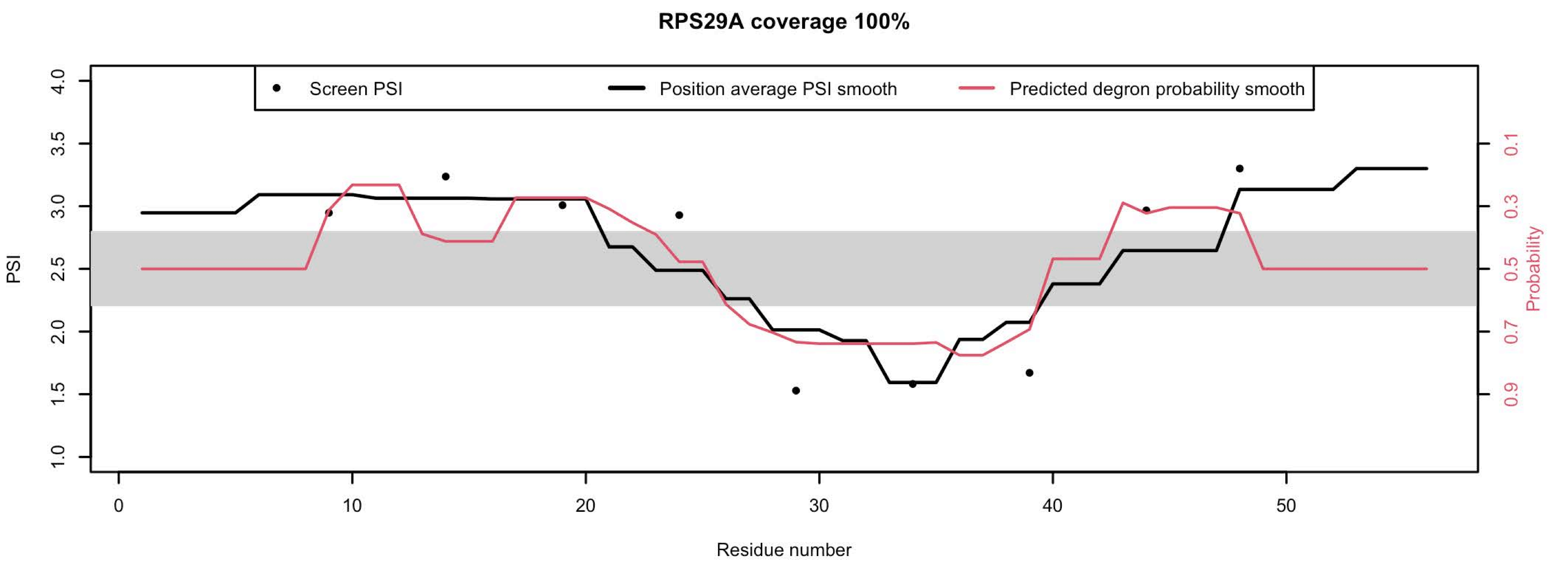

RPS29A coverage 100%
Screen PSI
Position average PSI smooth
Predicted degron probability smooth
PSI
1.0
1.5
2.0
2.5
3.0
3.5
4.0
Probability
0.1
0.3
0.5
0.7
0.9
Residue number
0
10
20
30
40
50

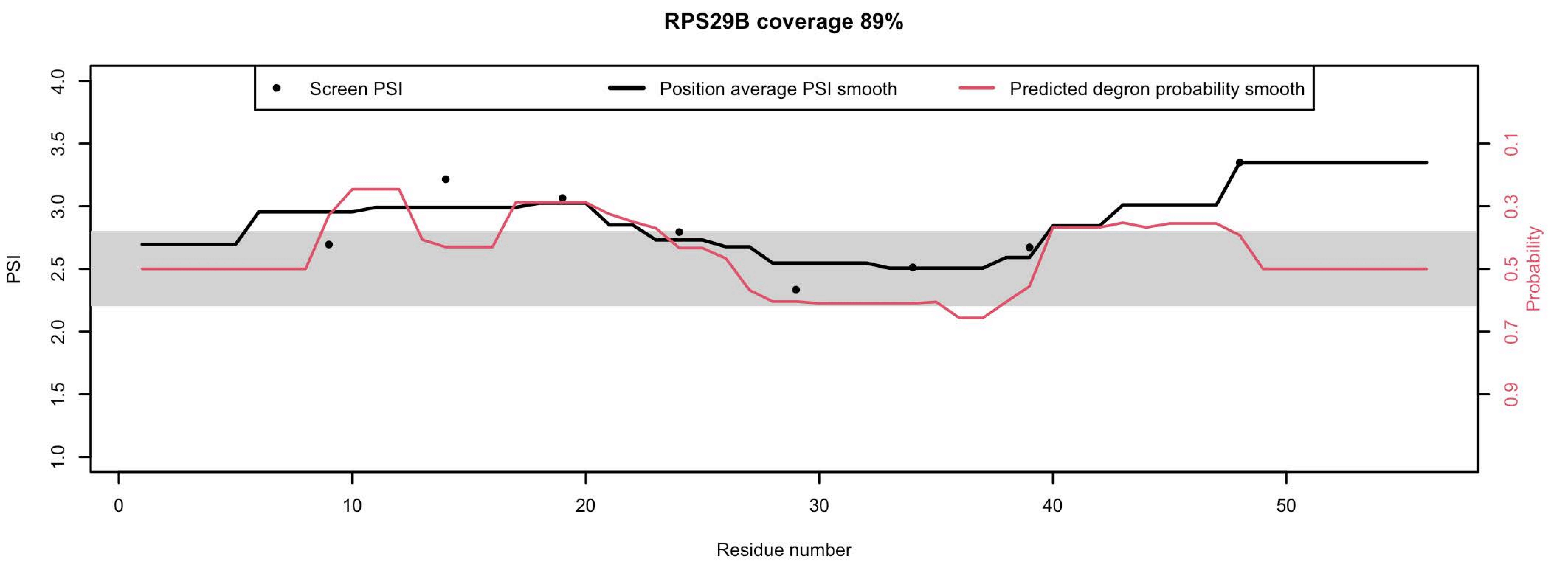

RPS29B coverage 89%
Screen PSI
Position average PSI smooth
Predicted degron probability smooth
PSI
4.0
3.5
3.0
2.5
2.0
1.5
1.0
Probability
0.1
0.3
0.5
0.7
0.9
0
10
20
30
40
50
Residue number

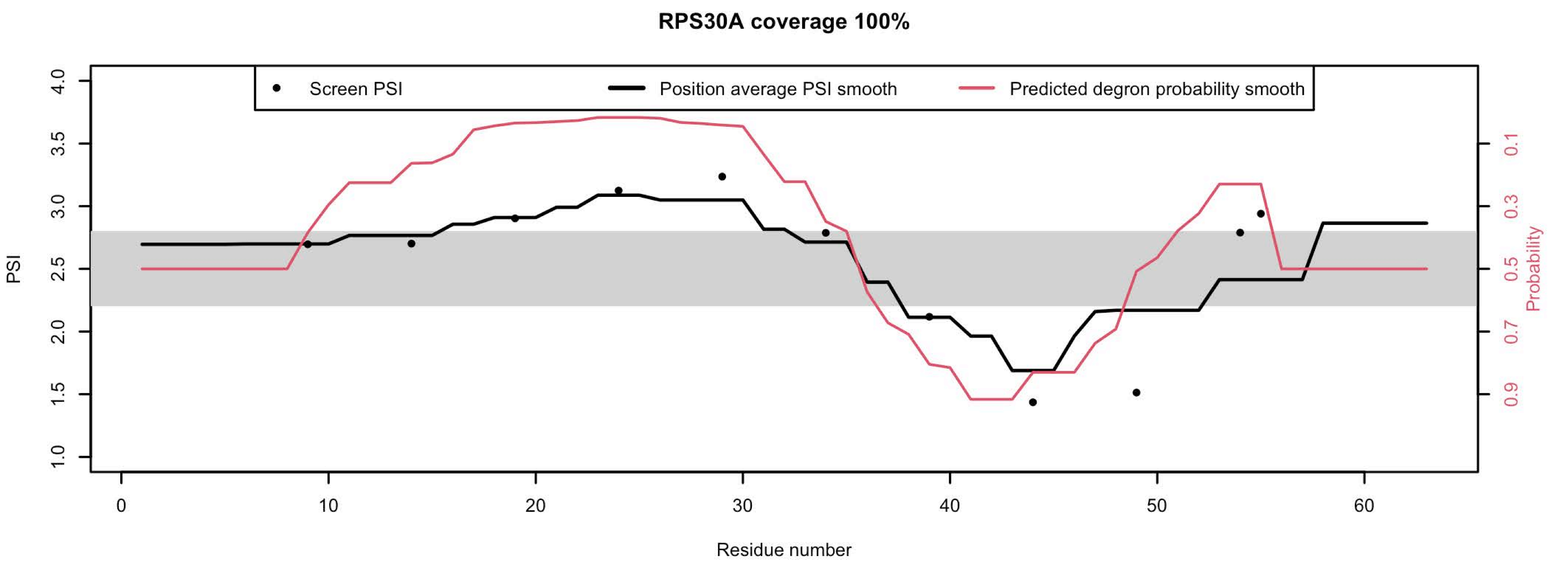

RPS30A coverage 100%
Screen PSI
Position average PSI smooth
Predicted degron probability smooth
PSI
1.0
1.5
2.0
2.5
3.0
3.5
4.0
Probability
0.1
0.3
0.5
0.7
0.9
Residue number
0
10
20
30
40
50
60

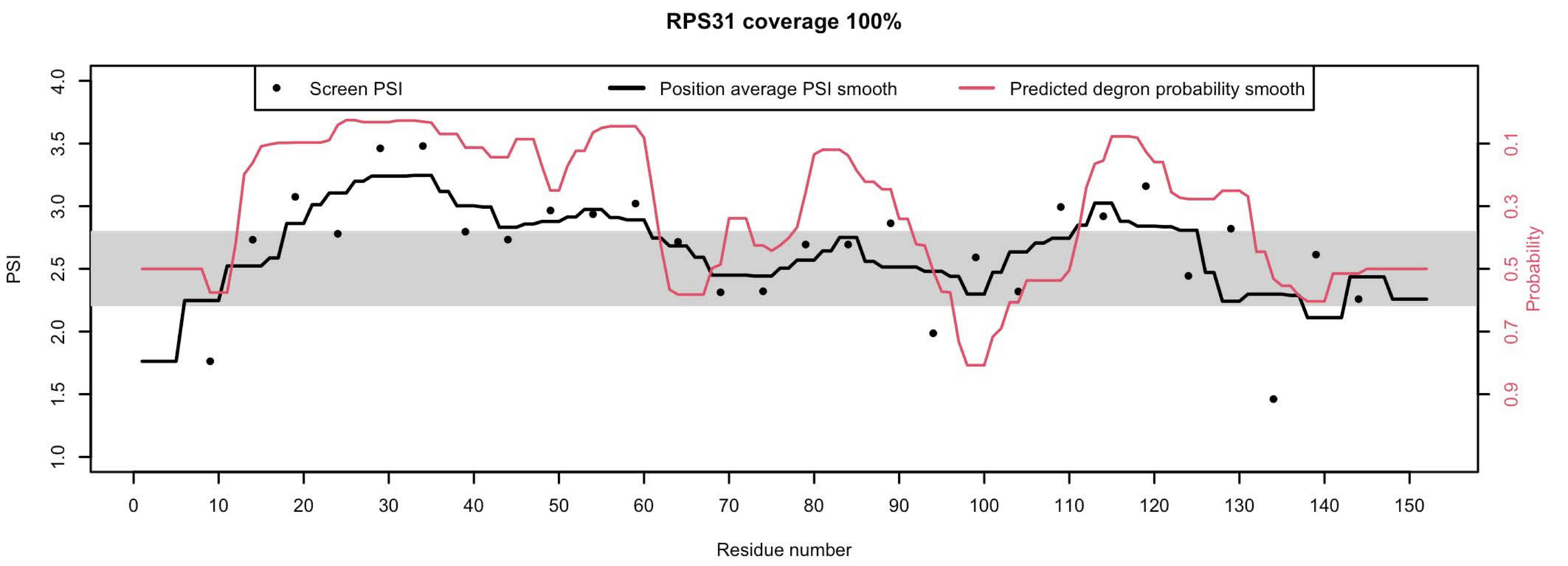
RPS31 coverage 100%
Screen PSI
Position average PSI smooth
Predicted degron probability smooth
PSI
Probability
Residue number
1.0
1.5
2.0
2.5
3.0
3.5
4.0
0.1
0.3
0.5
0.7
0.9
0
10
20
30
40
50
60
70
80
90
100
110
120
130
140
150

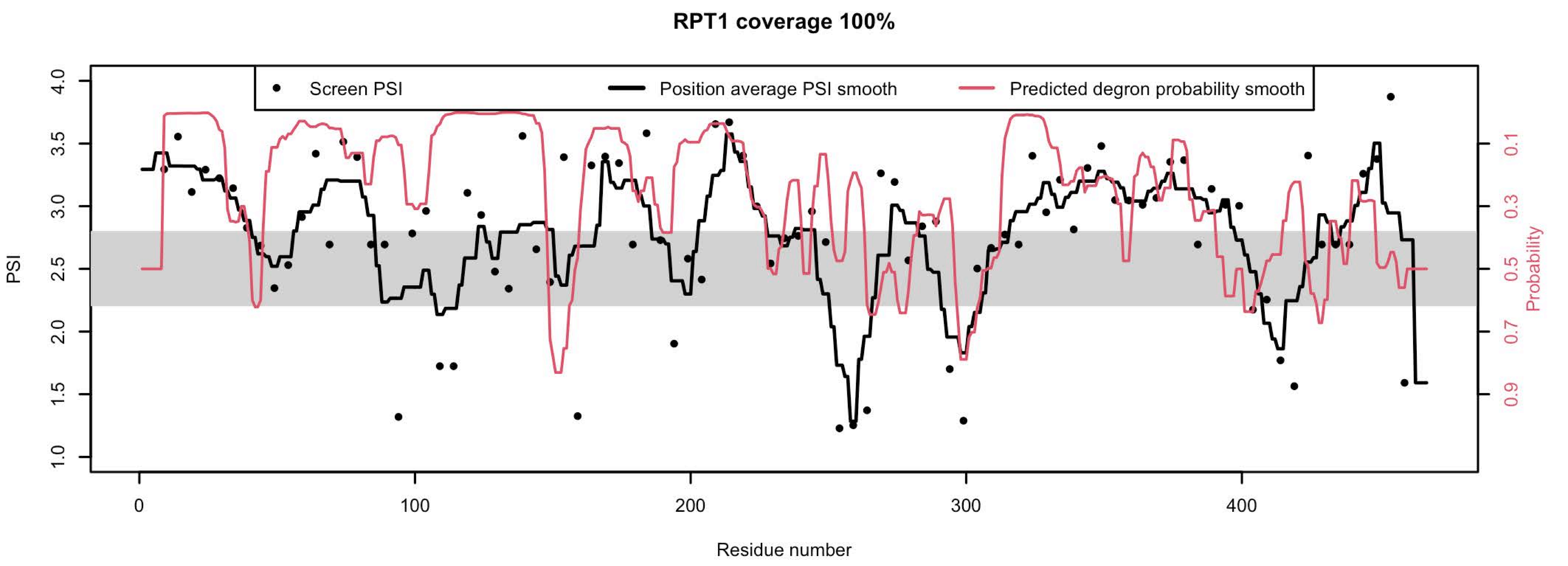
RPT1 coverage 100%
Screen PSI
Position average PSI smooth
Predicted degron probability smooth
PSI
1.0
1.5
2.0
2.5
3.0
3.5
4.0
0
100
200
300
400
Residue number
Probability
0.1
0.3
0.5
0.7
0.9

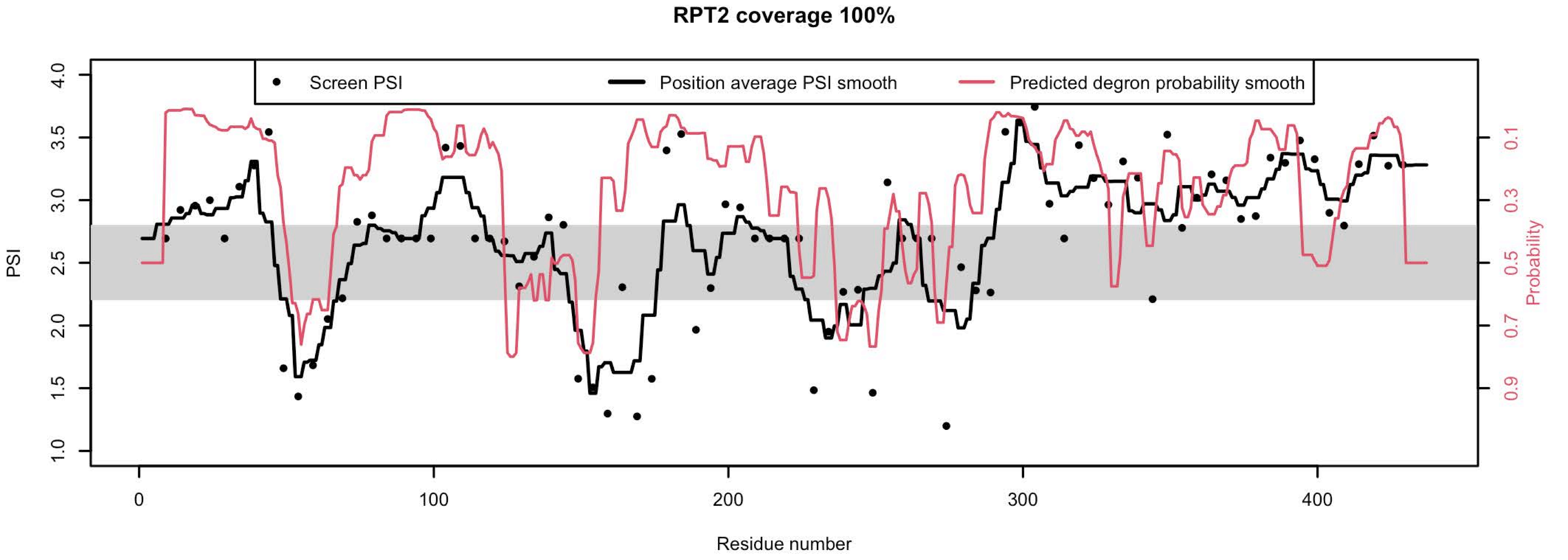

RPT2 coverage 100%
Screen PSI
Position average PSI smooth
Predicted degron probability smooth
PSI
1.0
1.5
2.0
2.5
3.0
3.5
4.0
Probability
0.1
0.3
0.5
0.7
0.9
0
100
200
300
400
Residue number

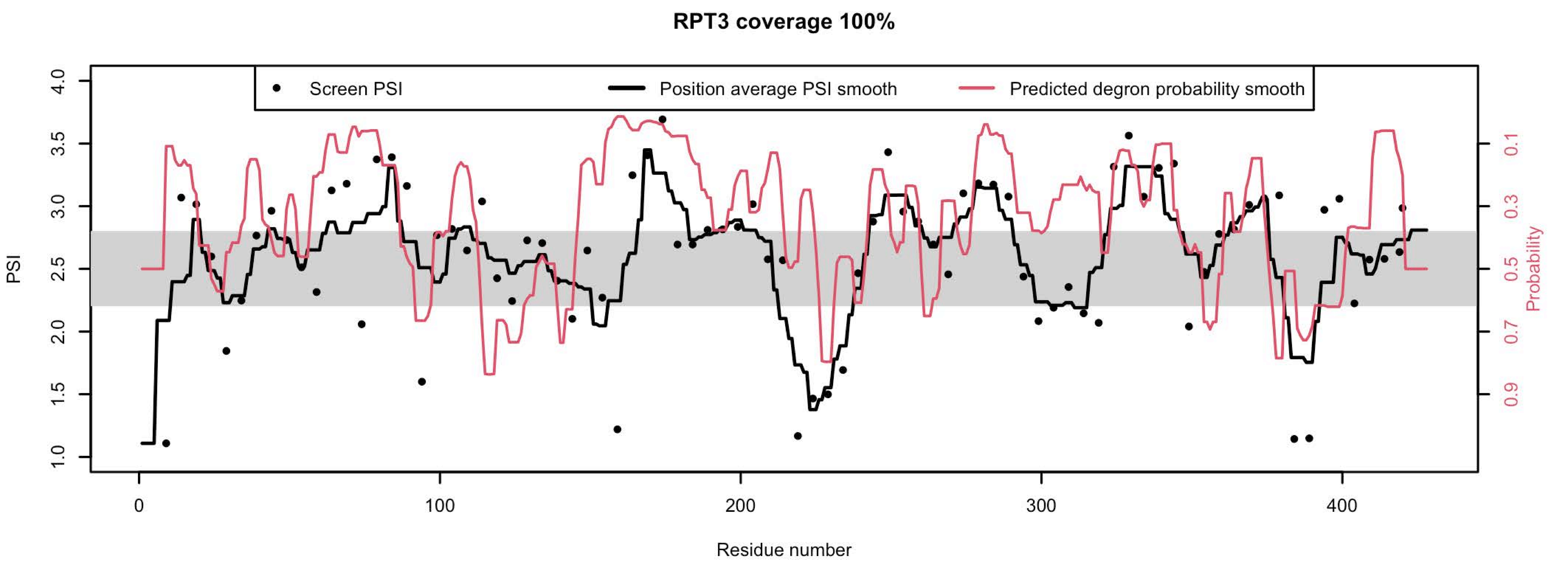
RPT3 coverage 100%
Screen PSI
Position average PSI smooth
Predicted degron probability smooth
PSI
1.0
1.5
2.0
2.5
3.0
3.5
4.0
Probability
0.1
0.3
0.5
0.7
0.9
0
100
200
300
400
Residue number

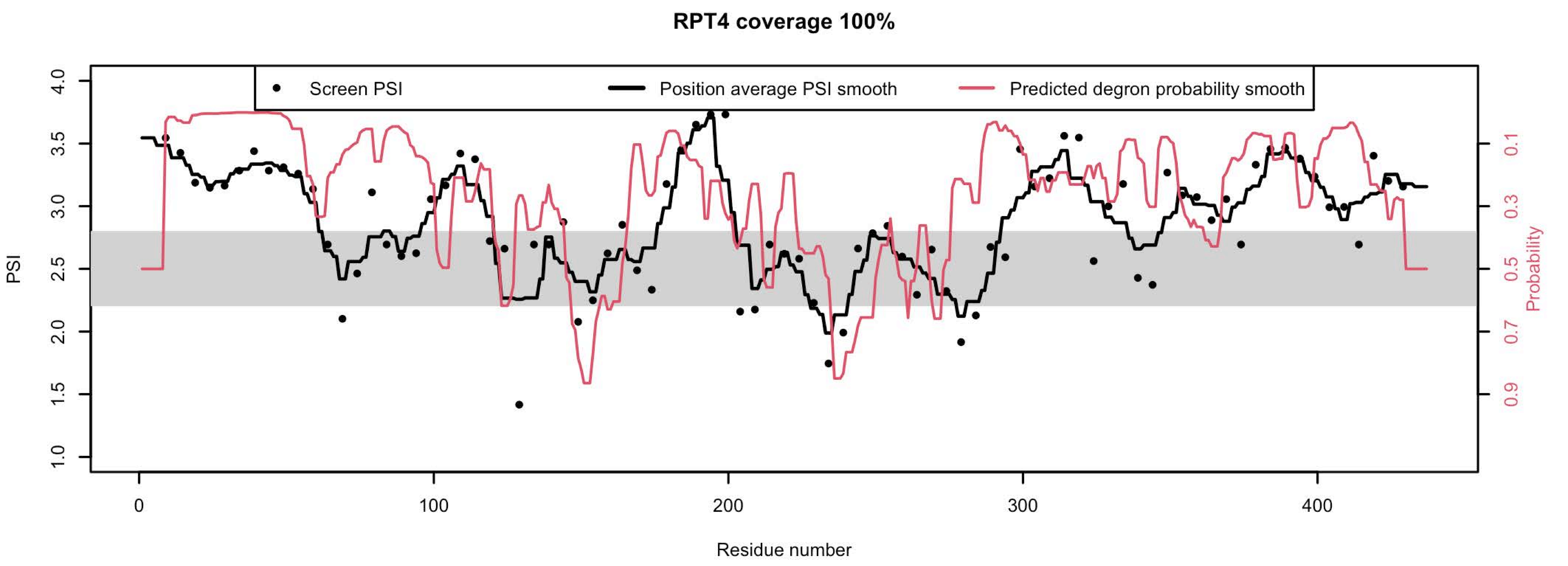

RPT4 coverage 100%
Screen PSI
Position average PSI smooth
Predicted degron probability smooth
PSI
1.0
1.5
2.0
2.5
3.0
3.5
4.0
Probability
0.1
0.3
0.5
0.7
0.9
0
100
200
300
400
Residue number

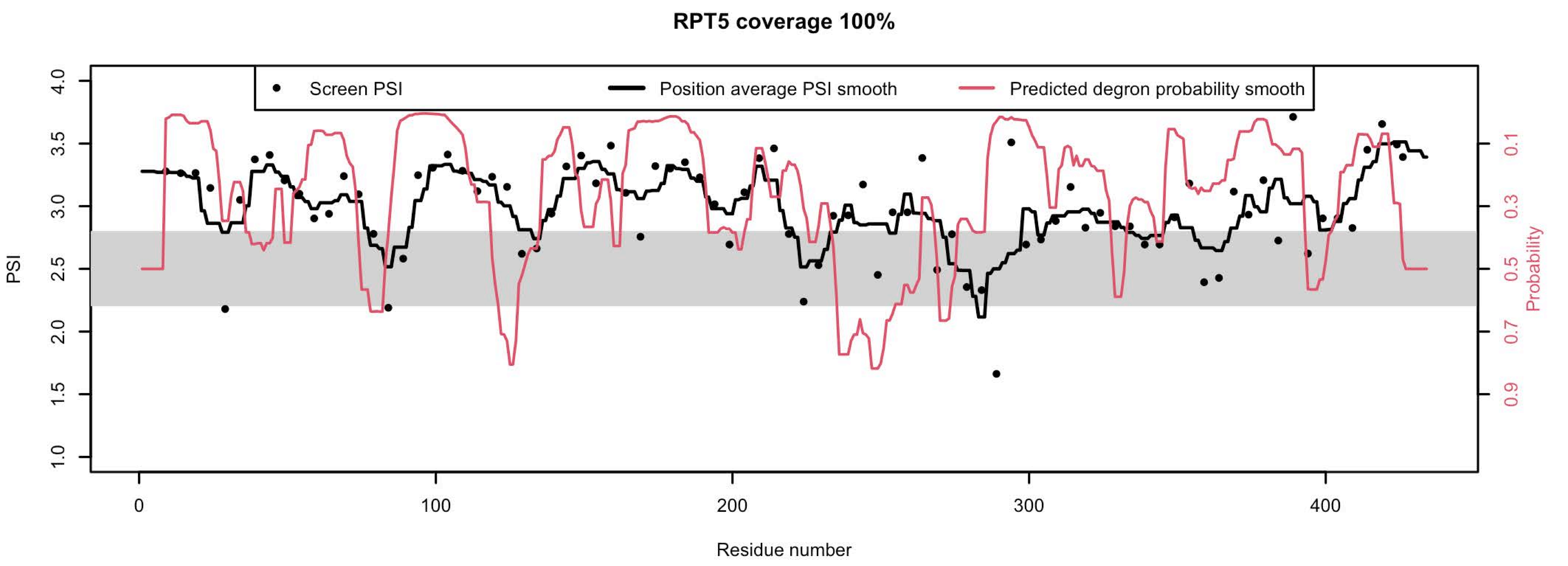
RPT5 coverage 100%
Screen PSI
Position average PSI smooth
Predicted degron probability smooth
PSI
1.0
1.5
2.0
2.5
3.0
3.5
4.0
Probability
0.1
0.3
0.5
0.7
0.9
Residue number
0
100
200
300
400

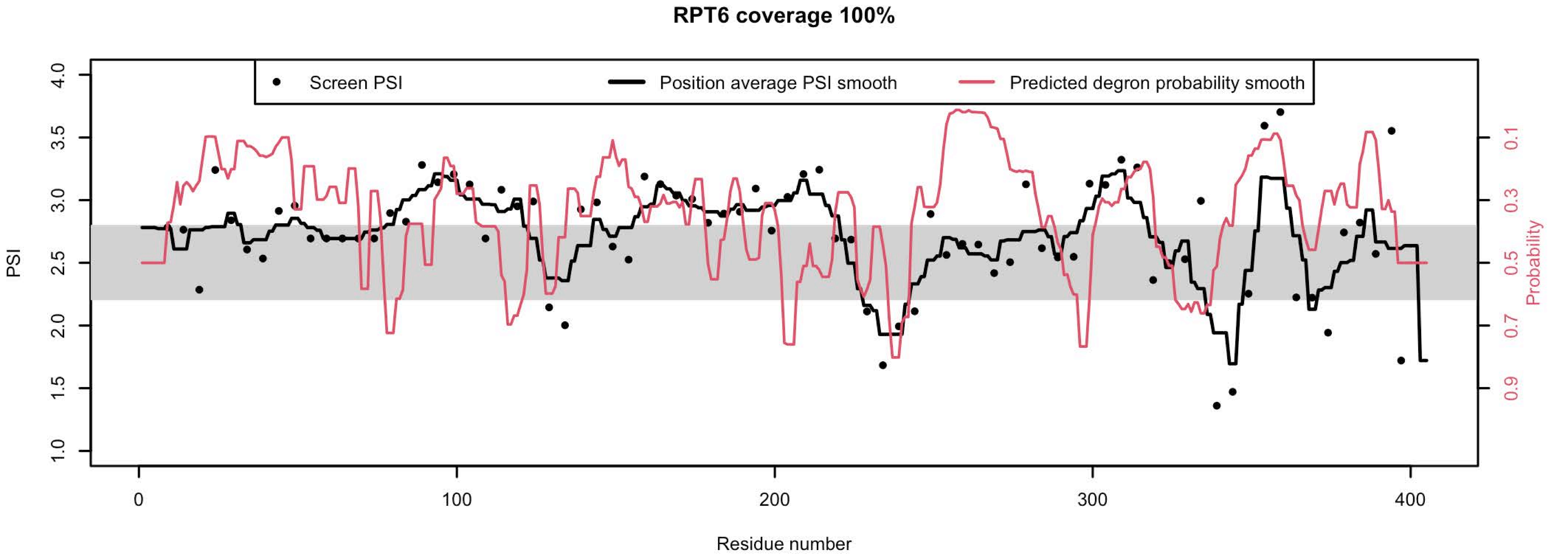
RPT6 coverage 100%
Screen PSI
Position average PSI smooth
Predicted degron probability smooth
PSI
1.0
1.5
2.0
2.5
3.0
3.5
4.0
Probability
0.1
0.3
0.5
0.7
0.9
0
100
200
300
400
Residue number

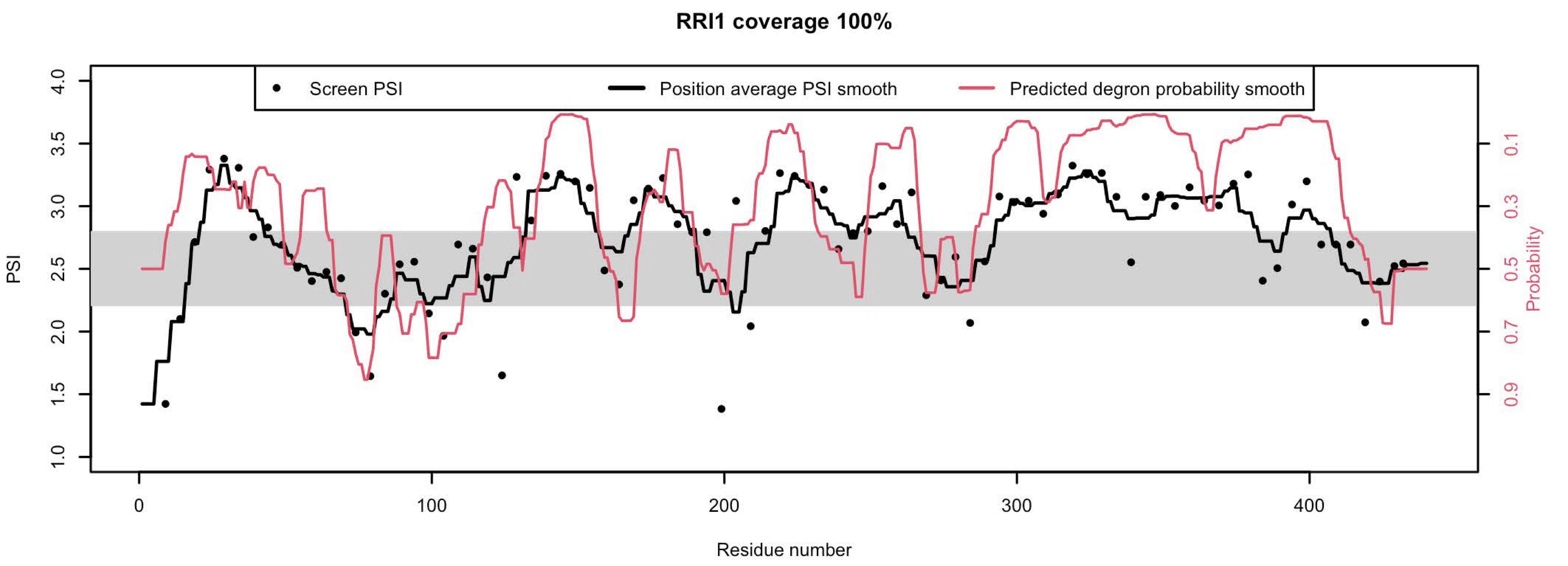
RRI1 coverage 100%
Screen PSI
Position average PSI smooth
Predicted degron probability smooth
PSI
1.0
1.5
2.0
2.5
3.0
3.5
4.0
Probability
0.1
0.3
0.5
0.7
0.9
0
100
200
300
400
Residue number

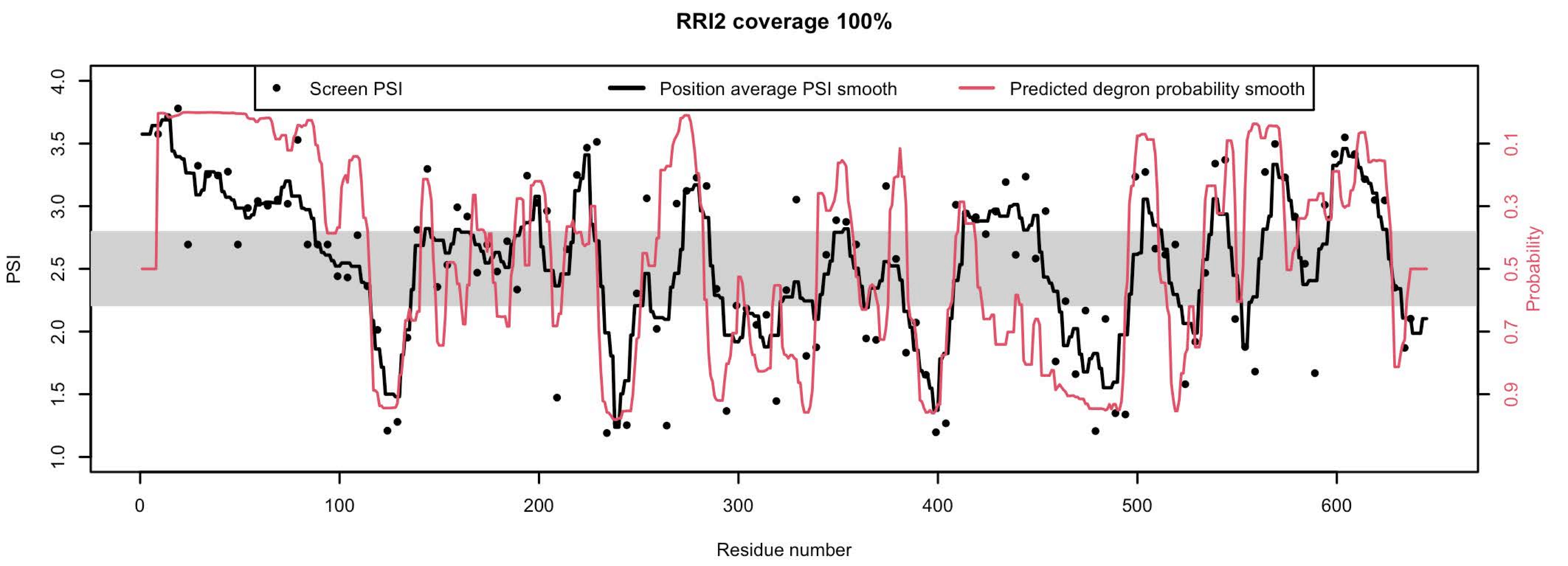

RRI2 coverage 100%
Screen PSI
Position average PSI smooth
Predicted degron probability smooth
PSI
Probability
Residue number
1.0
1.5
2.0
2.5
3.0
3.5
4.0
0.1
0.3
0.5
0.7
0.9
0
100
200
300
400
500
600

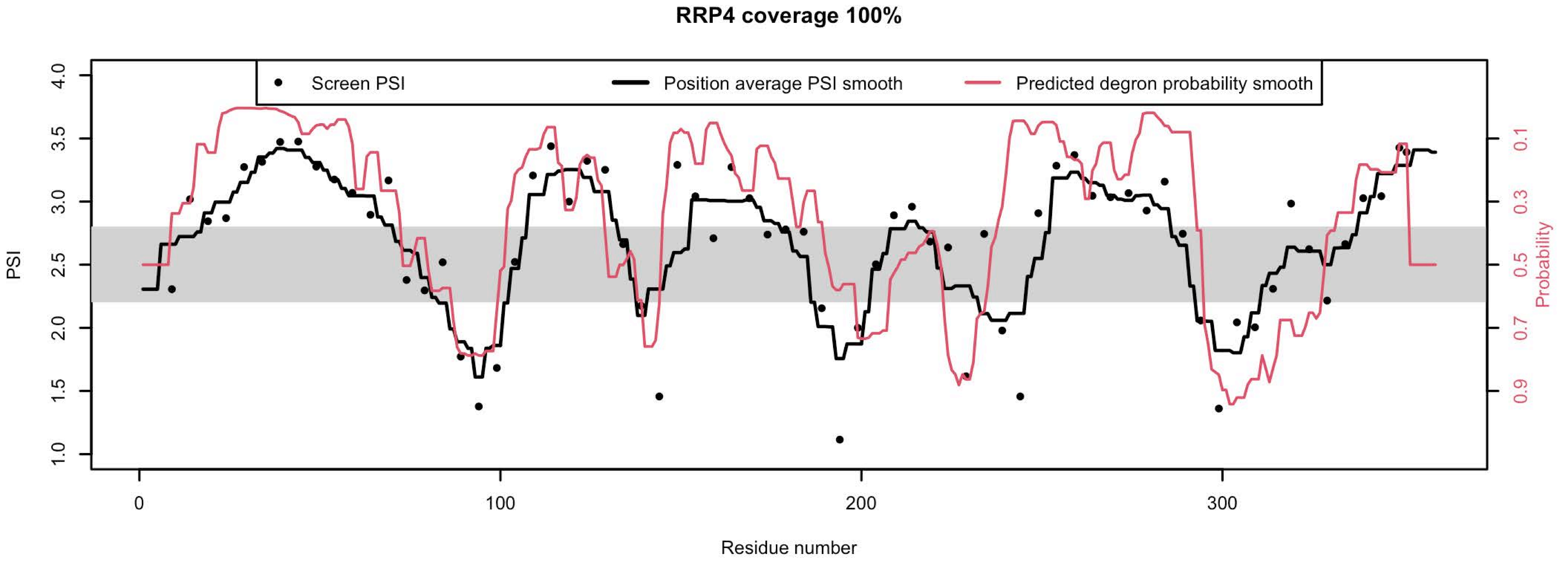

RRP4 coverage 100%
Screen PSI
Position average PSI smooth
Predicted degron probability smooth
PSI
Probability
Residue number
1.0
1.5
2.0
2.5
3.0
3.5
4.0
0.1
0.3
0.5
0.7
0.9
0
100
200
300

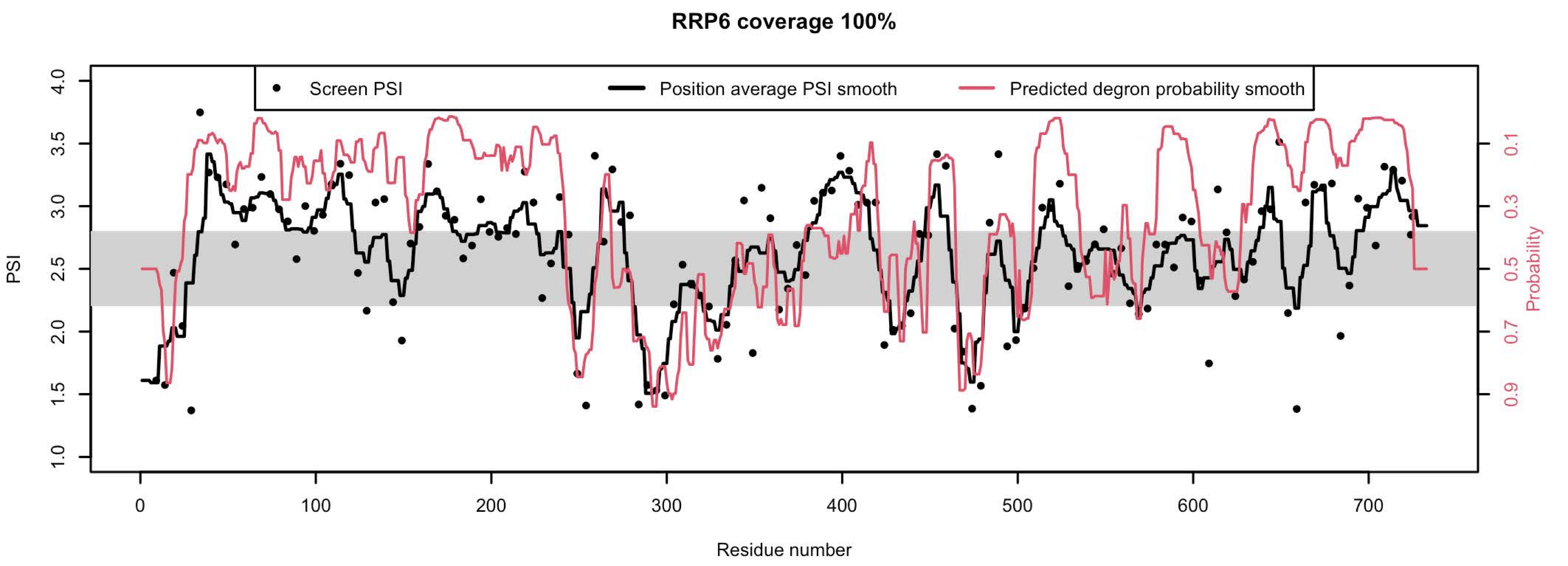

RRP6 coverage 100%
Screen PSI
Position average PSI smooth
Predicted degron probability smooth
PSI
1.0
1.5
2.0
2.5
3.0
3.5
4.0
Probability
0.1
0.3
0.5
0.7
0.9
0
100
200
300
400
500
600
700
Residue number

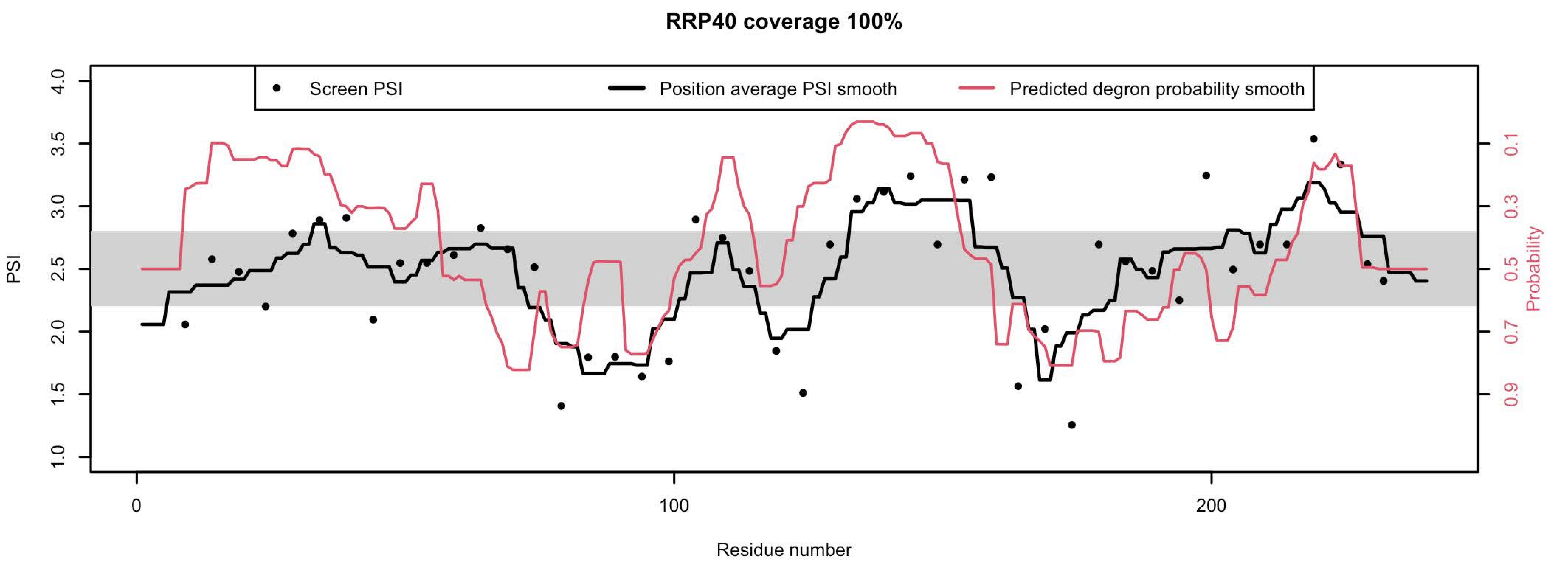
RRP40 coverage 100%
Screen PSI
Position average PSI smooth
Predicted degron probability smooth
PSI
1.0
1.5
2.0
2.5
3.0
3.5
4.0
Probability
0.1
0.3
0.5
0.7
0.9
0
100
200
Residue number

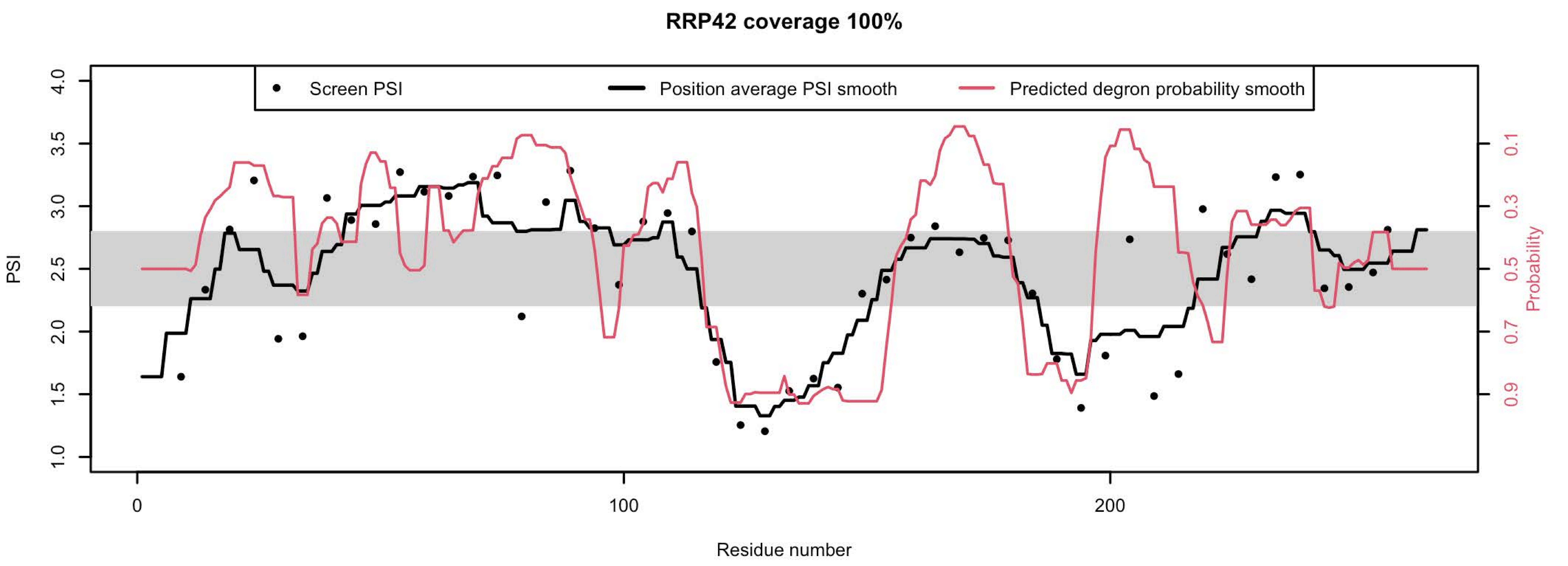

RRP42 coverage 100%
Screen PSI
Position average PSI smooth
Predicted degron probability smooth
PSI
Probability
Residue number
4.0
3.5
3.0
2.5
2.0
1.5
1.0
0.1
0.3
0.5
0.7
0.9
0
100
200

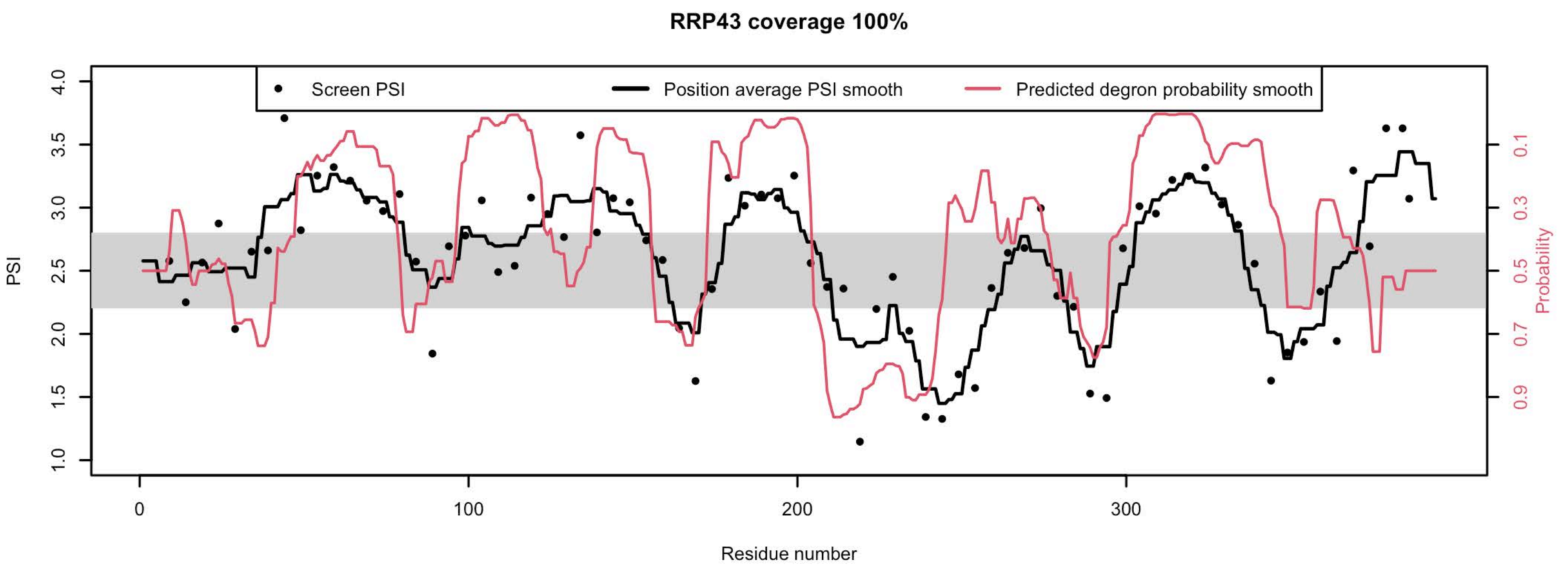

RRP43 coverage 100%
Screen PSI
Position average PSI smooth
Predicted degron probability smooth
PSI
1.0
1.5
2.0
2.5
3.0
3.5
4.0
Probability
0.1
0.3
0.5
0.7
0.9
0
100
200
300
Residue number

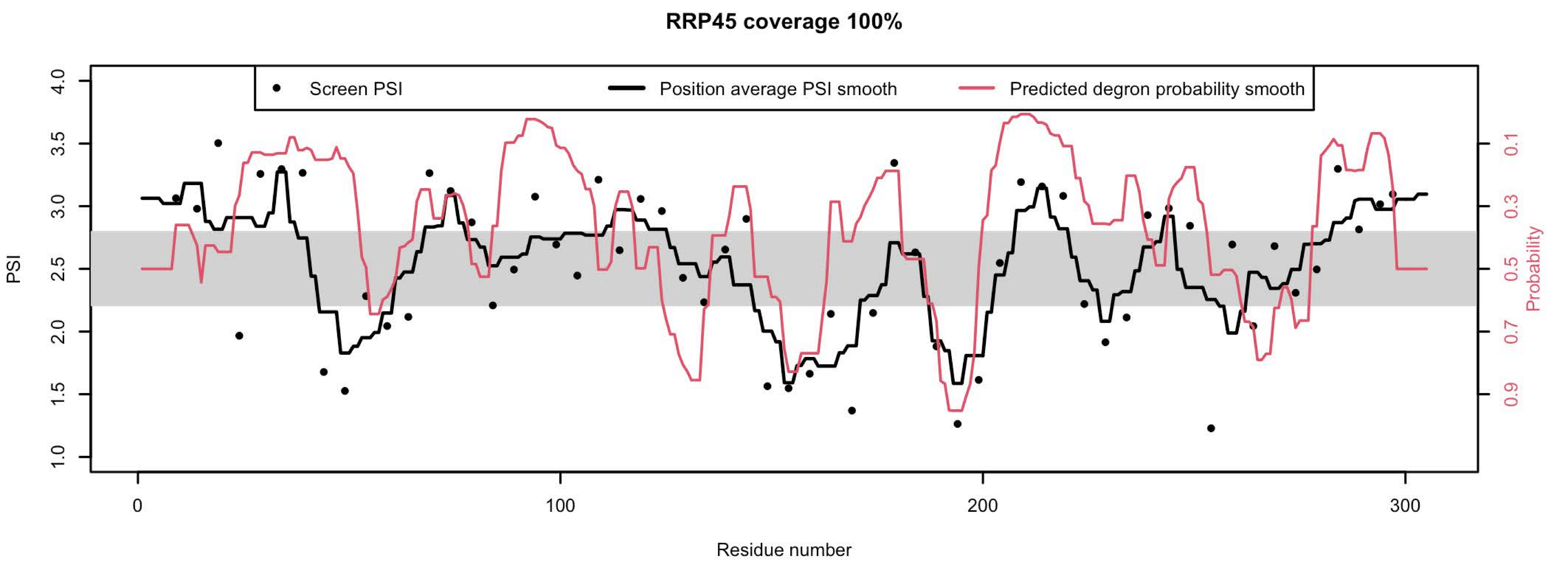

RRP45 coverage 100%
Screen PSI
Position average PSI smooth
Predicted degron probability smooth
PSI
1.0
1.5
2.0
2.5
3.0
3.5
4.0
Probability
0.1
0.3
0.5
0.7
0.9
0
100
200
300
Residue number

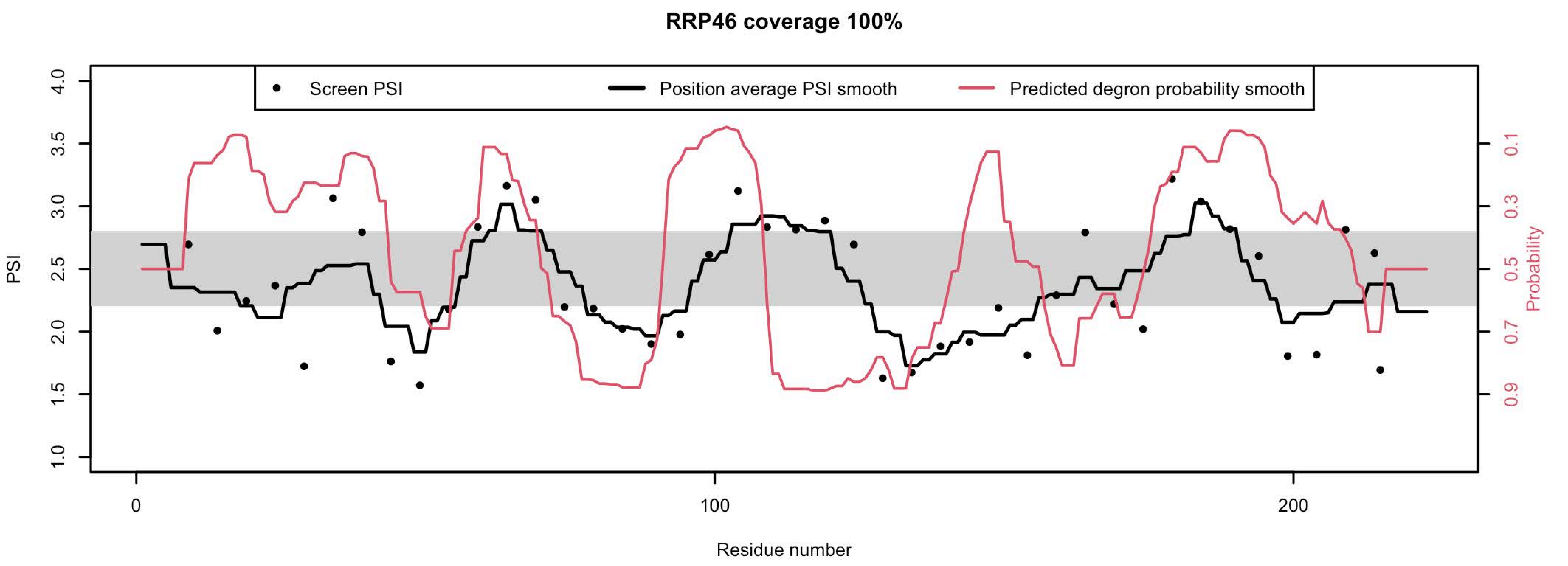

RRP46 coverage 100%
Screen PSI
Position average PSI smooth
Predicted degron probability smooth
PSI
4.0
3.5
3.0
2.5
2.0
1.5
1.0
Probability
0.1
0.3
0.5
0.7
0.9
0
100
200
Residue number

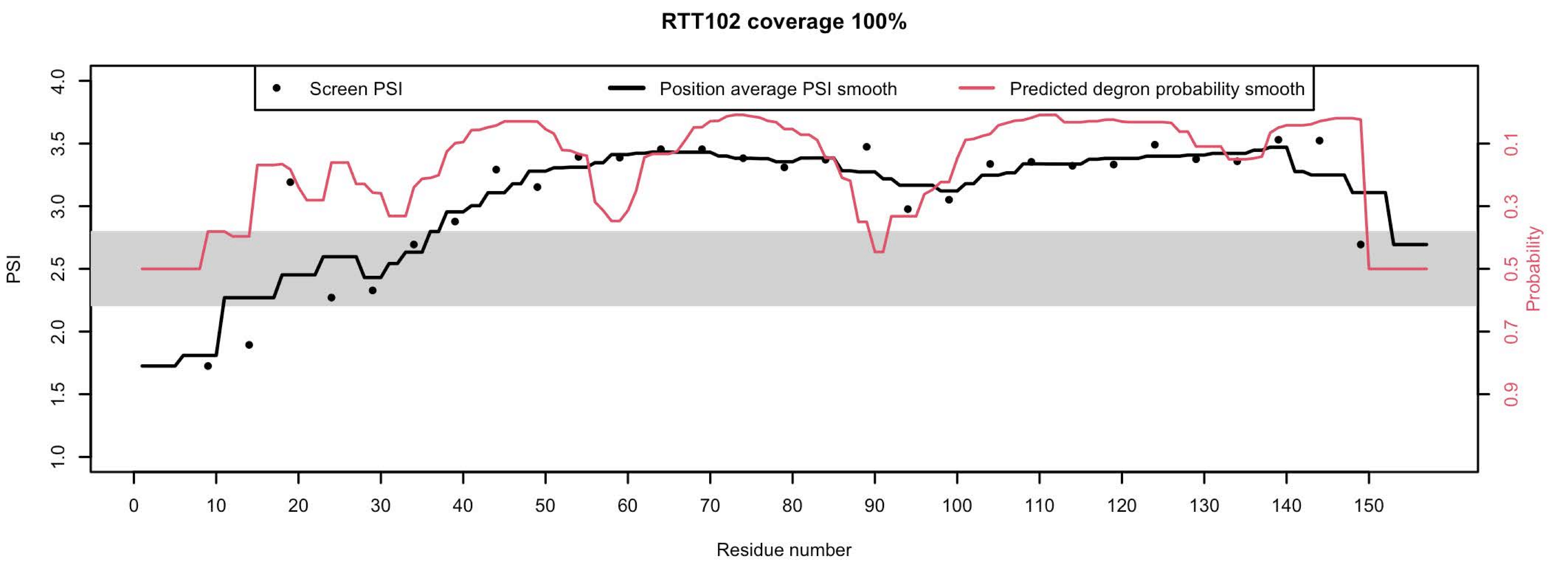
RTT102 coverage 100%
Screen PSI
Position average PSI smooth
Predicted degron probability smooth
PSI
1.0
1.5
2.0
2.5
3.0
3.5
4.0
Probability
0.1
0.3
0.5
0.7
0.9
0
10
20
30
40
50
60
70
80
90
100
110
120
130
140
150
Residue number

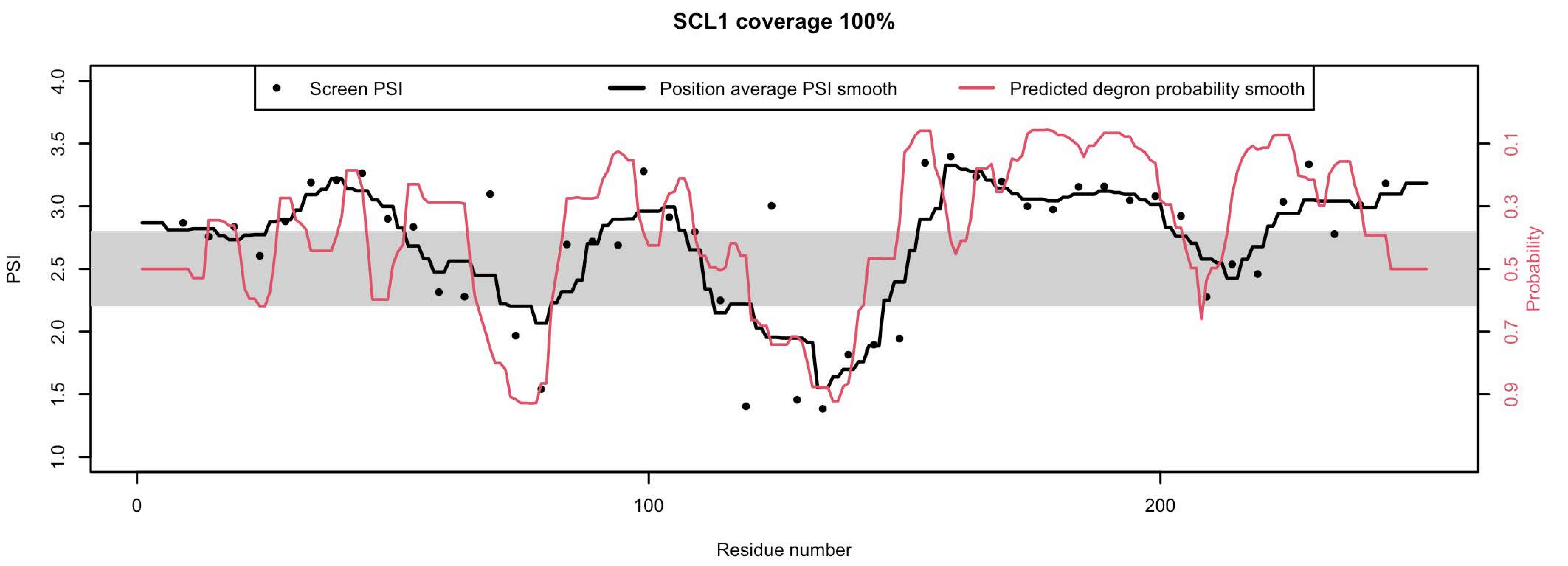
SCL1 coverage 100%
Screen PSI
Position average PSI smooth
Predicted degron probability smooth
PSI
4.0
3.5
3.0
2.5
2.0
1.5
1.0
Probability
0.1
0.3
0.5
0.7
0.9
0
100
200
Residue number

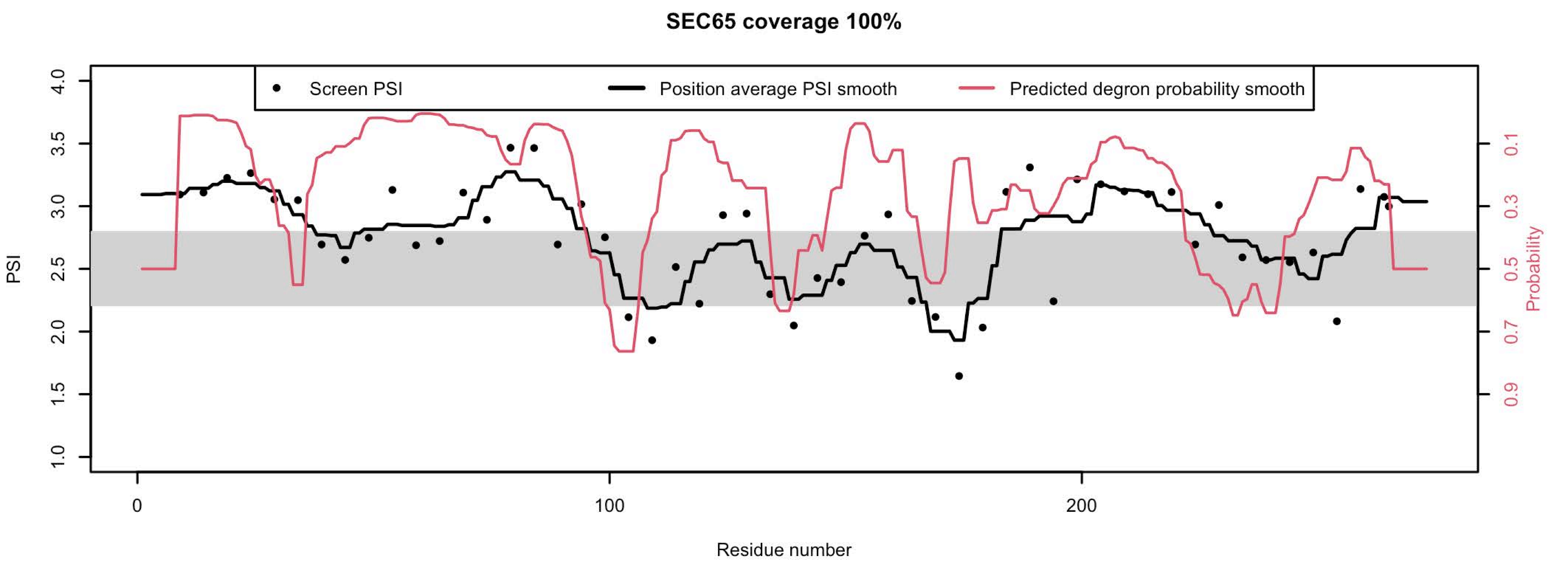
SEC65 coverage 100%
Screen PSI
Position average PSI smooth
Predicted degron probability smooth
PSI
1.0
1.5
2.0
2.5
3.0
3.5
4.0
Probability
0.1
0.3
0.5
0.7
0.9
0
100
200
Residue number

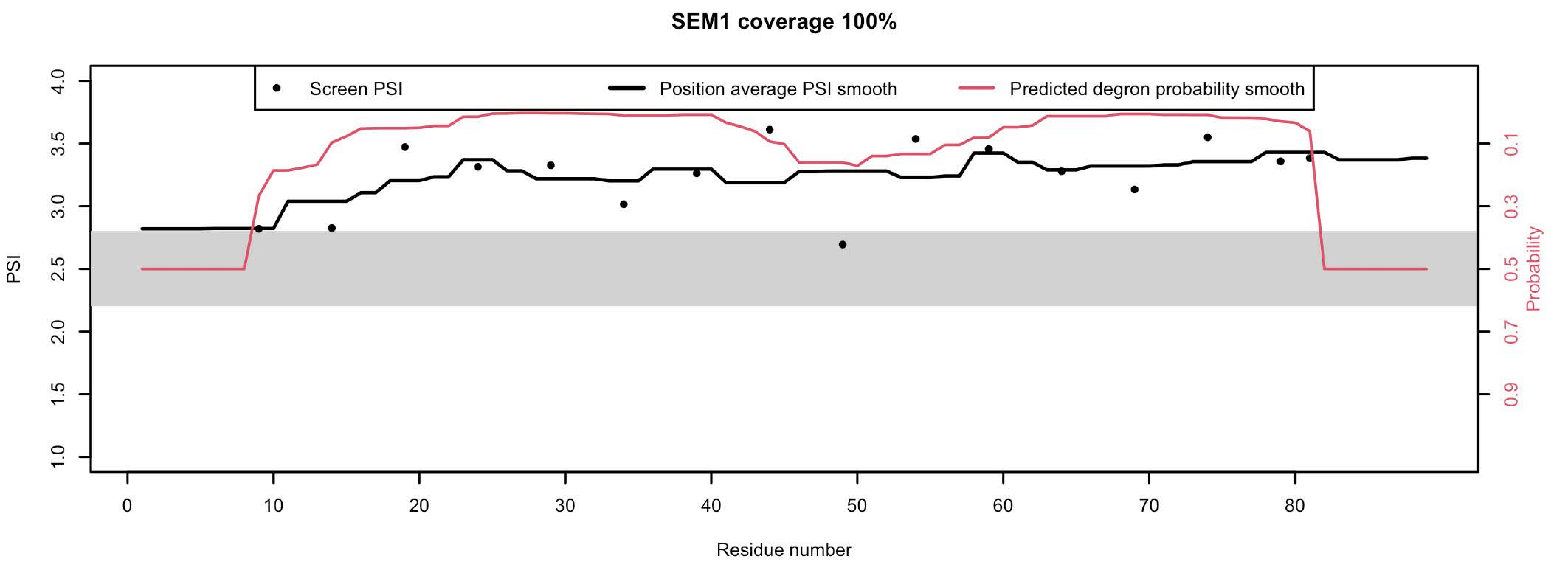
SEM1 coverage 100%
Screen PSI
Position average PSI smooth
Predicted degron probability smooth
PSI
Probability
Residue number
1.0
1.5
2.0
2.5
3.0
3.5
4.0
0.1
0.3
0.5
0.7
0.9
0
10
20
30
40
50
60
70
80

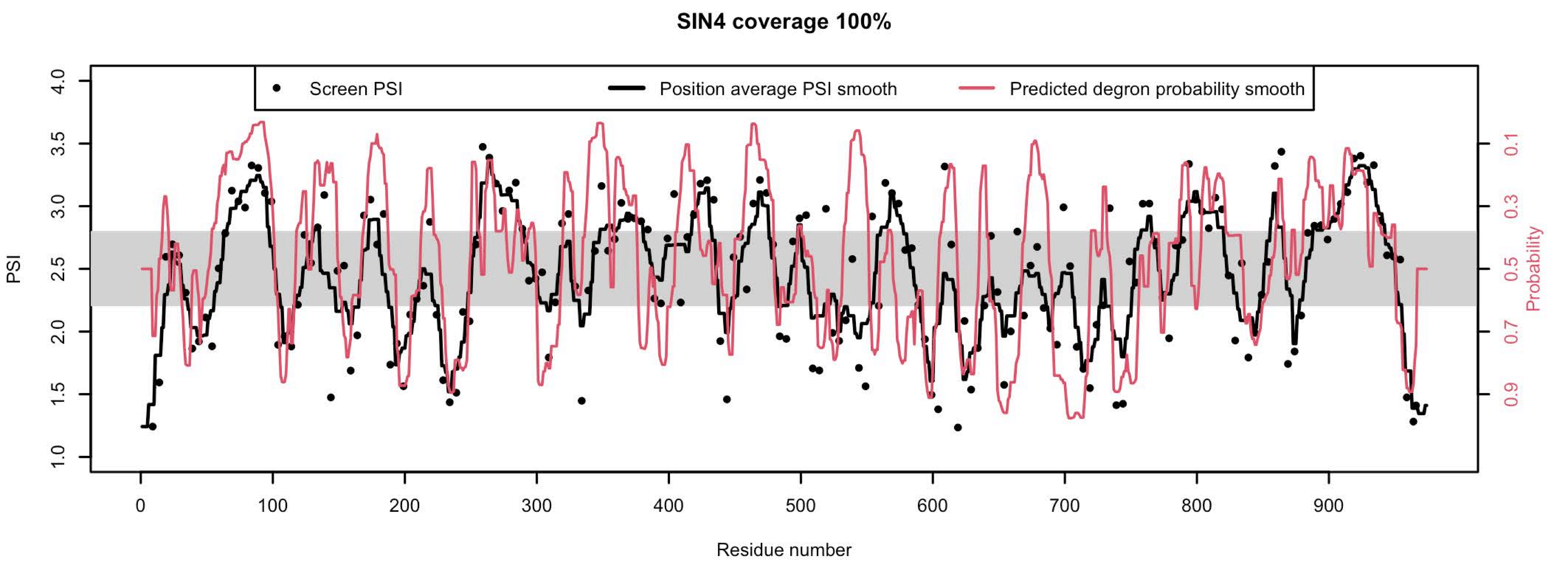

SIN4 coverage 100%
Screen PSI
Position average PSI smooth
Predicted degron probability smooth
PSI
1.0
1.5
2.0
2.5
3.0
3.5
4.0
Probability
0.1
0.3
0.5
0.7
0.9
Residue number
0
100
200
300
400
500
600
700
800
900

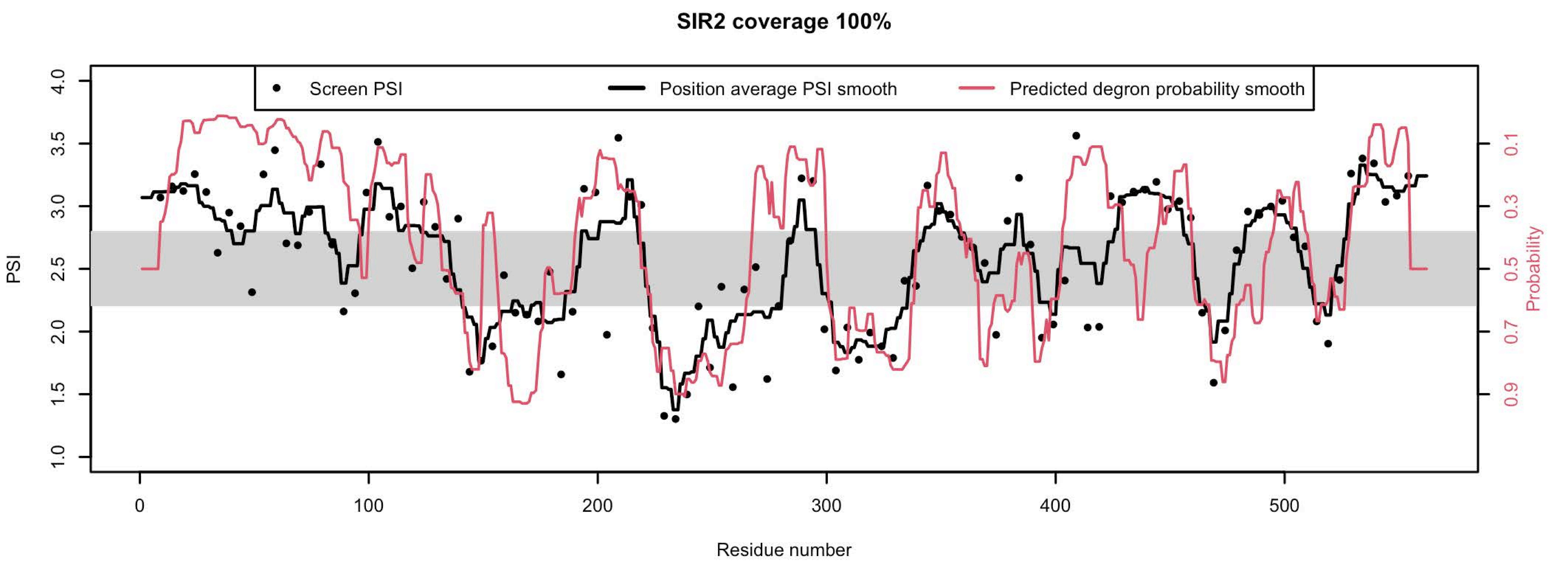
SIR2 coverage 100%
Screen PSI
Position average PSI smooth
Predicted degron probability smooth
PSI
4.0
3.5
3.0
2.5
2.0
1.5
1.0
Probability
0.1
0.3
0.5
0.7
0.9
0
100
200
300
400
500
Residue number

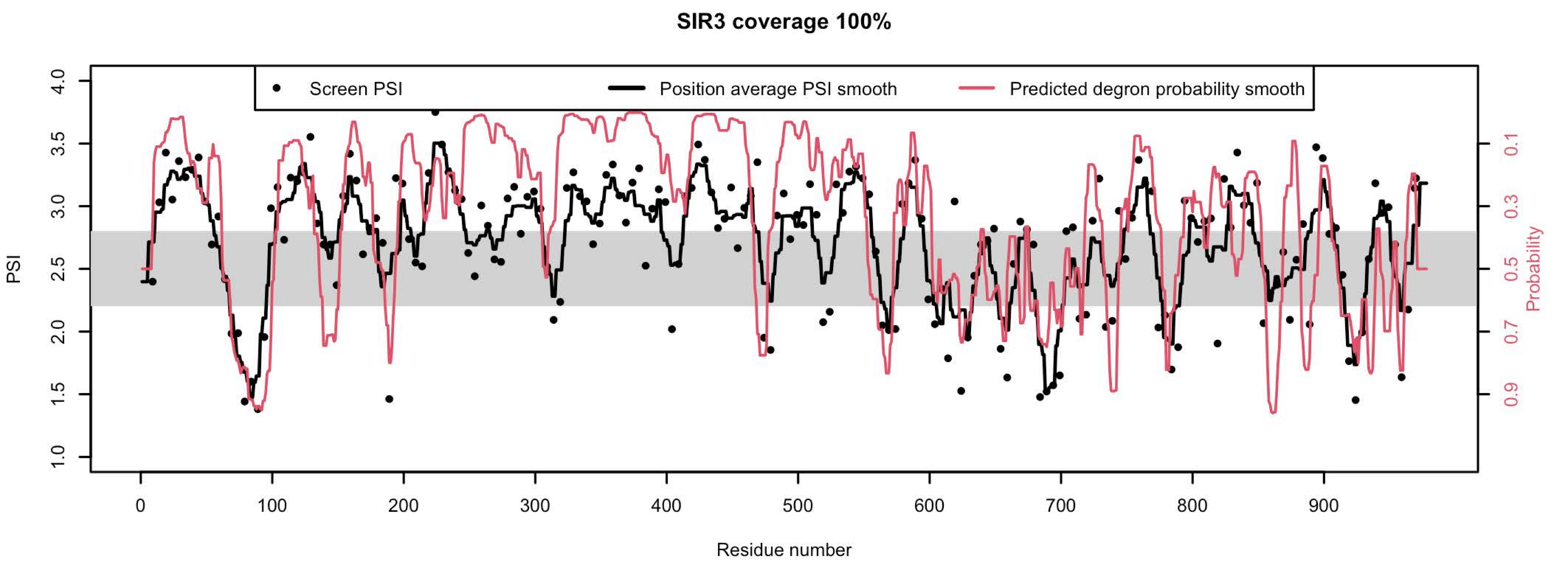

SIR3 coverage 100%
Screen PSI
Position average PSI smooth
Predicted degron probability smooth
PSI
1.0
1.5
2.0
2.5
3.0
3.5
4.0
Probability
0.1
0.3
0.5
0.7
0.9
Residue number
0
100
200
300
400
500
600
700
800
900

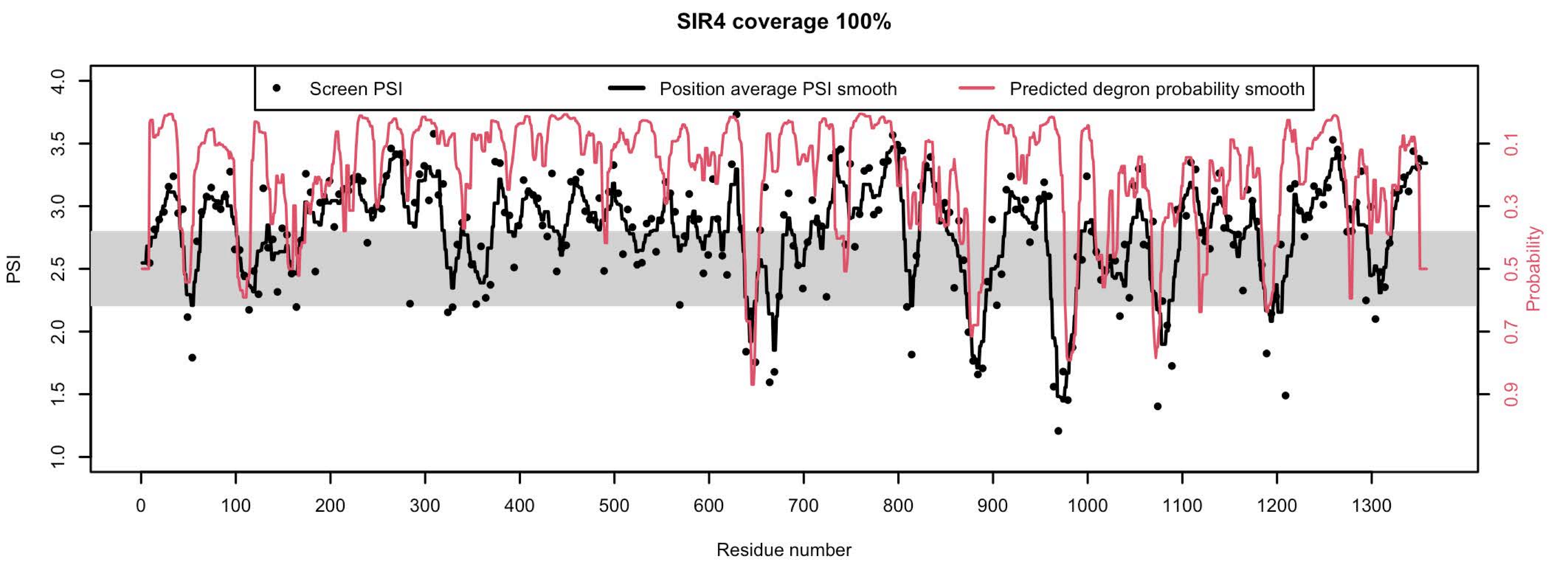
SIR4 coverage 100%
Screen PSI
Position average PSI smooth
Predicted degron probability smooth
PSI
Probability
Residue number
1.0
1.5
2.0
2.5
3.0
3.5
4.0
0.1
0.3
0.5
0.7
0.9
0
100
200
300
400
500
600
700
800
900
1000
1100
1200
1300

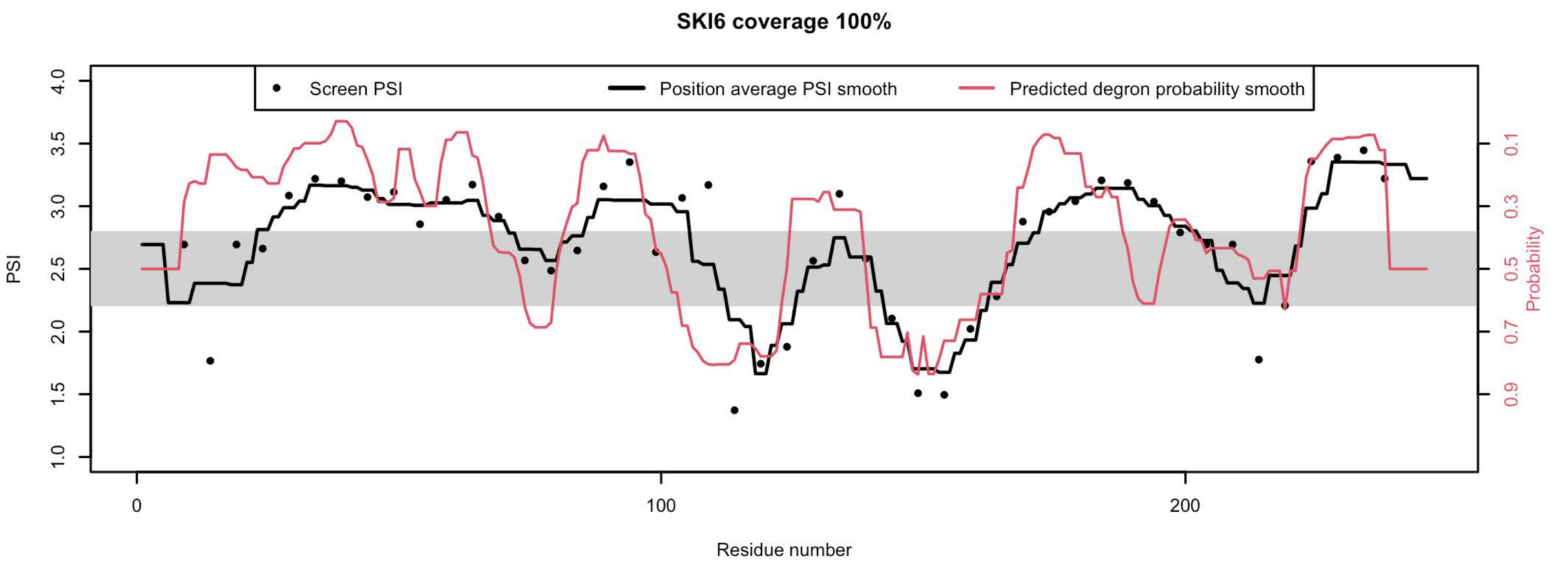
SKI6 coverage 100%
Screen PSI
Position average PSI smooth
Predicted degron probability smooth
PSI
Probability
Residue number
4.0
3.5
3.0
2.5
2.0
1.5
1.0
0.1
0.3
0.5
0.7
0.9
0
100
200

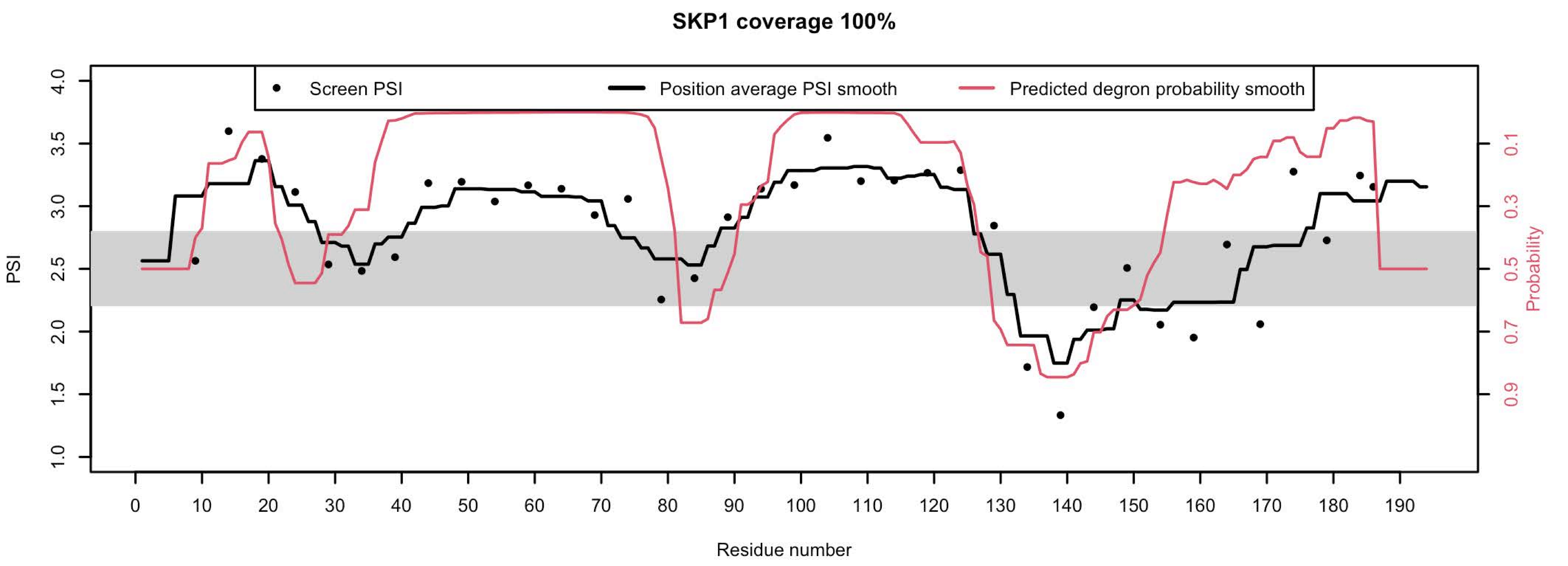

SKP1 coverage 100%
Screen PSI
Position average PSI smooth
Predicted degron probability smooth
PSI
1.0
1.5
2.0
2.5
3.0
3.5
4.0
Probability
0.1
0.3
0.5
0.7
0.9
Residue number
0
10
20
30
40
50
60
70
80
90
100
110
120
130
140
150
160
170
180
190

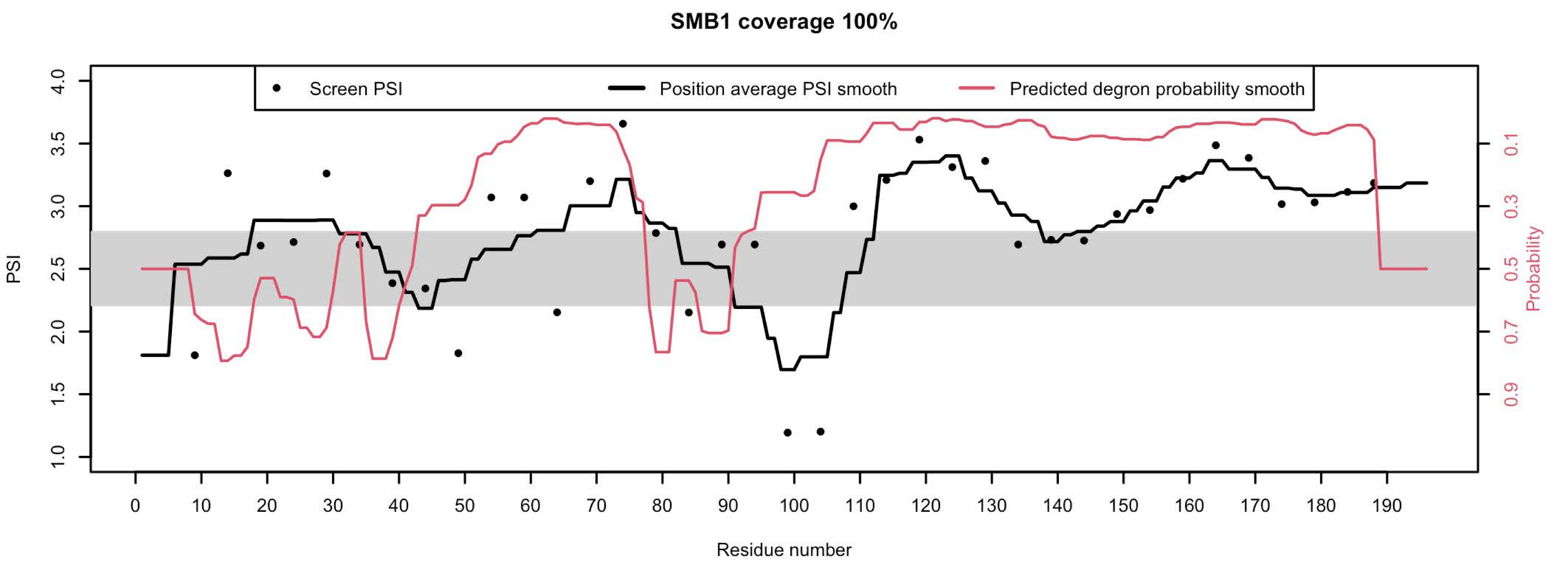
SMB1 coverage 100%
Screen PSI
Position average PSI smooth
Predicted degron probability smooth
PSI
1.0
1.5
2.0
2.5
3.0
3.5
4.0
Probability
0.1
0.3
0.5
0.7
0.9
0
10
20
30
40
50
60
70
80
90
100
110
120
130
140
150
160
170
180
190
Residue number

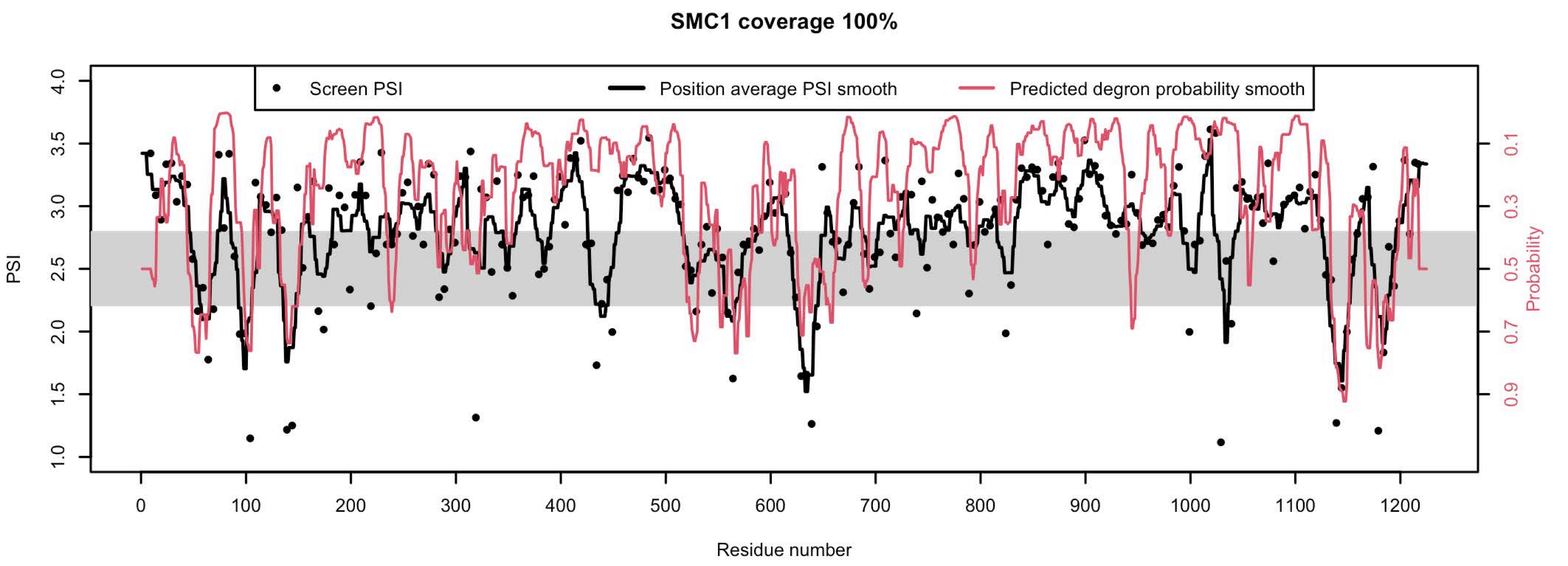
SMC1 coverage 100%
Screen PSI
Position average PSI smooth
Predicted degron probability smooth
PSI
Probability
Residue number
1.0
1.5
2.0
2.5
3.0
3.5
4.0
0.1
0.3
0.5
0.7
0.9
0
100
200
300
400
500
600
700
800
900
1000
1100
1200

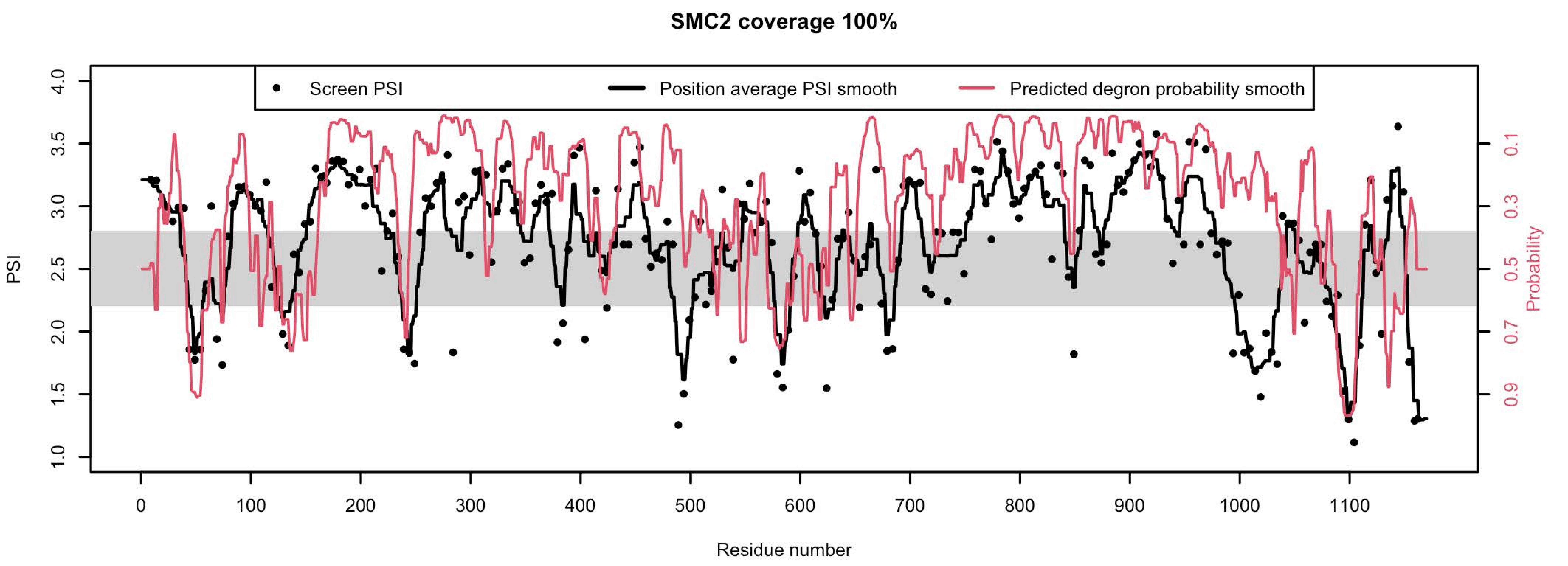

SMC2 coverage 100%
Screen PSI
Position average PSI smooth
Predicted degron probability smooth
PSI
Probability
Residue number
1.0
1.5
2.0
2.5
3.0
3.5
4.0
0.1
0.3
0.5
0.7
0.9
0
100
200
300
400
500
600
700
800
900
1000
1100

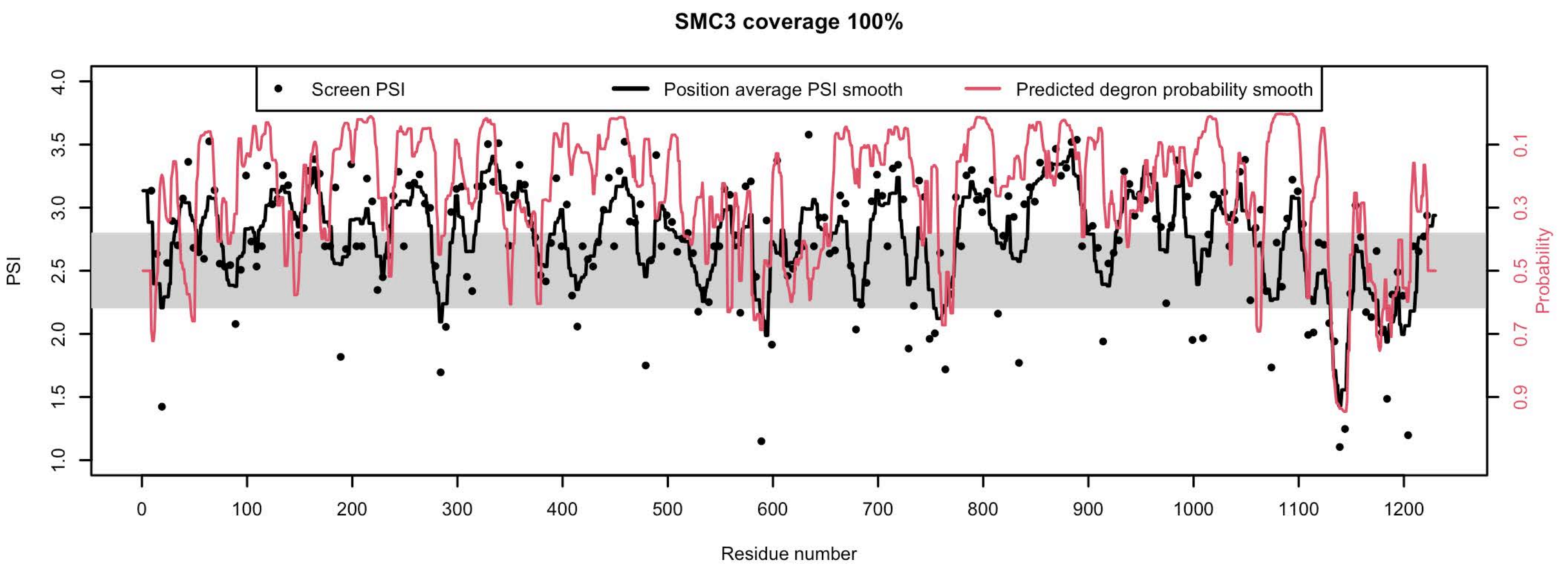

SMC3 coverage 100%
Screen PSI
Position average PSI smooth
Predicted degron probability smooth
PSI
Probability
Residue number
4.0
3.5
3.0
2.5
2.0
1.5
1.0
0.1
0.3
0.5
0.7
0.9
0
100
200
300
400
500
600
700
800
900
1000
1100
1200

SMC4 coverage 100%

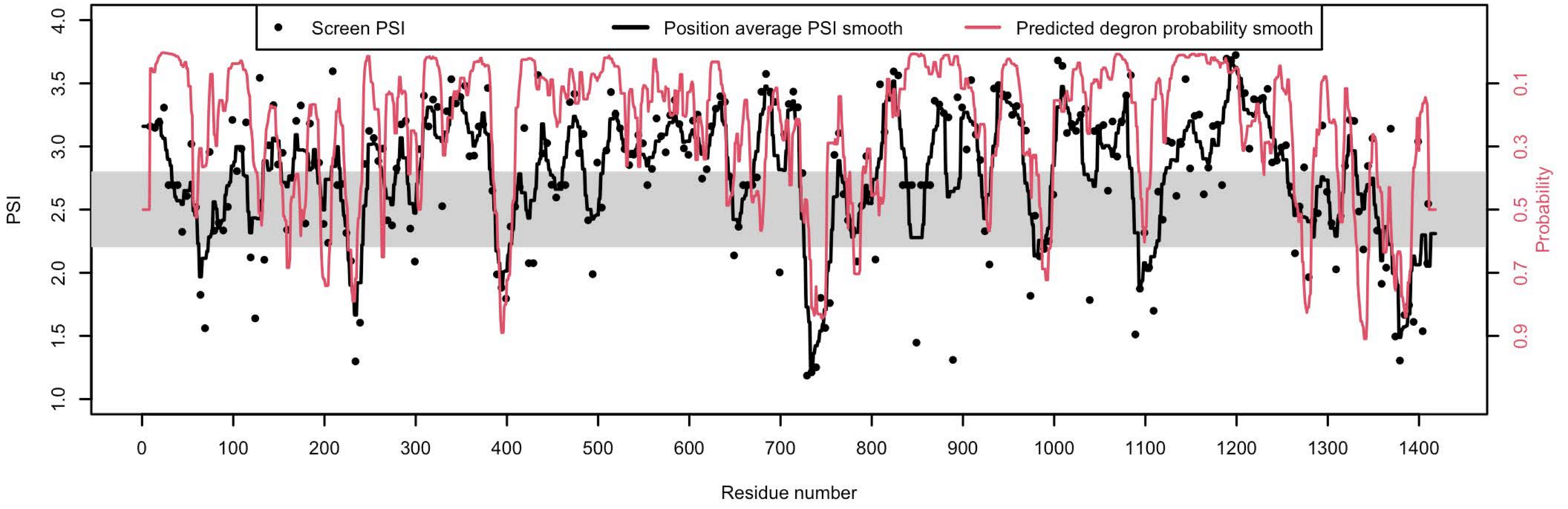

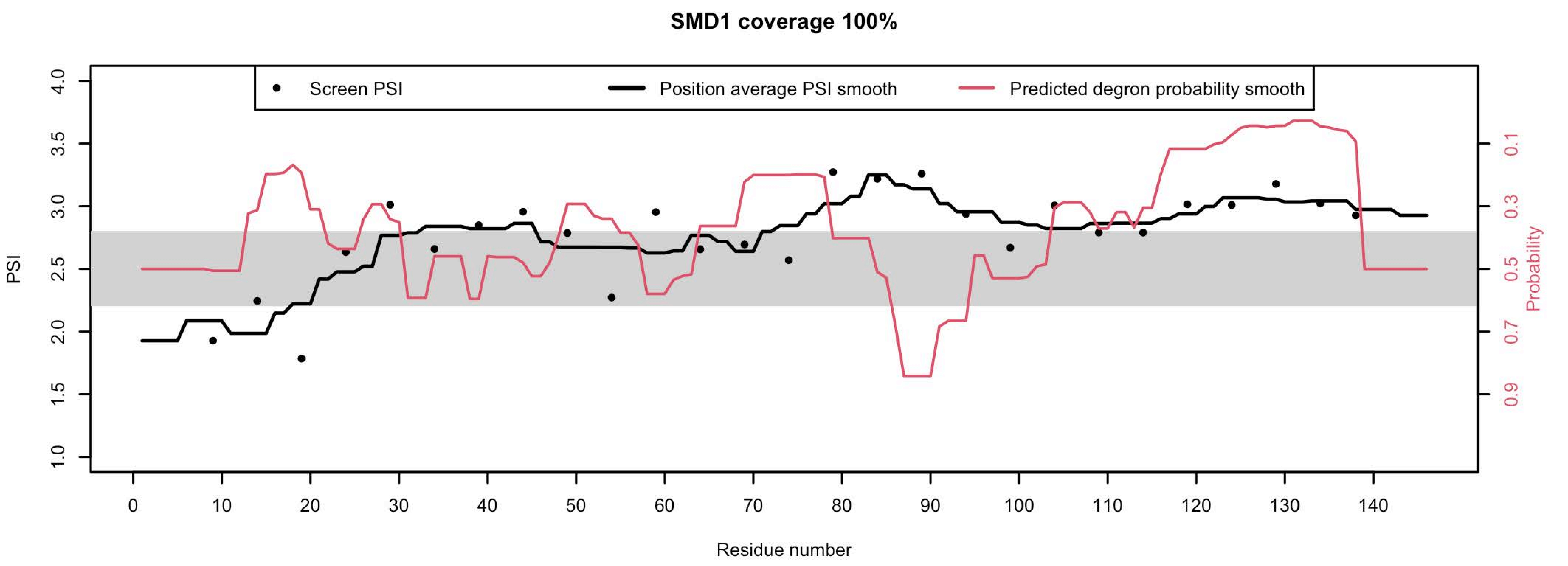
SMD1 coverage 100%
Screen PSI
Position average PSI smooth
Predicted degron probability smooth
PSI
1.0
1.5
2.0
2.5
3.0
3.5
4.0
Probability
0.1
0.3
0.5
0.7
0.9
Residue number
0
10
20
30
40
50
60
70
80
90
100
110
120
130
140

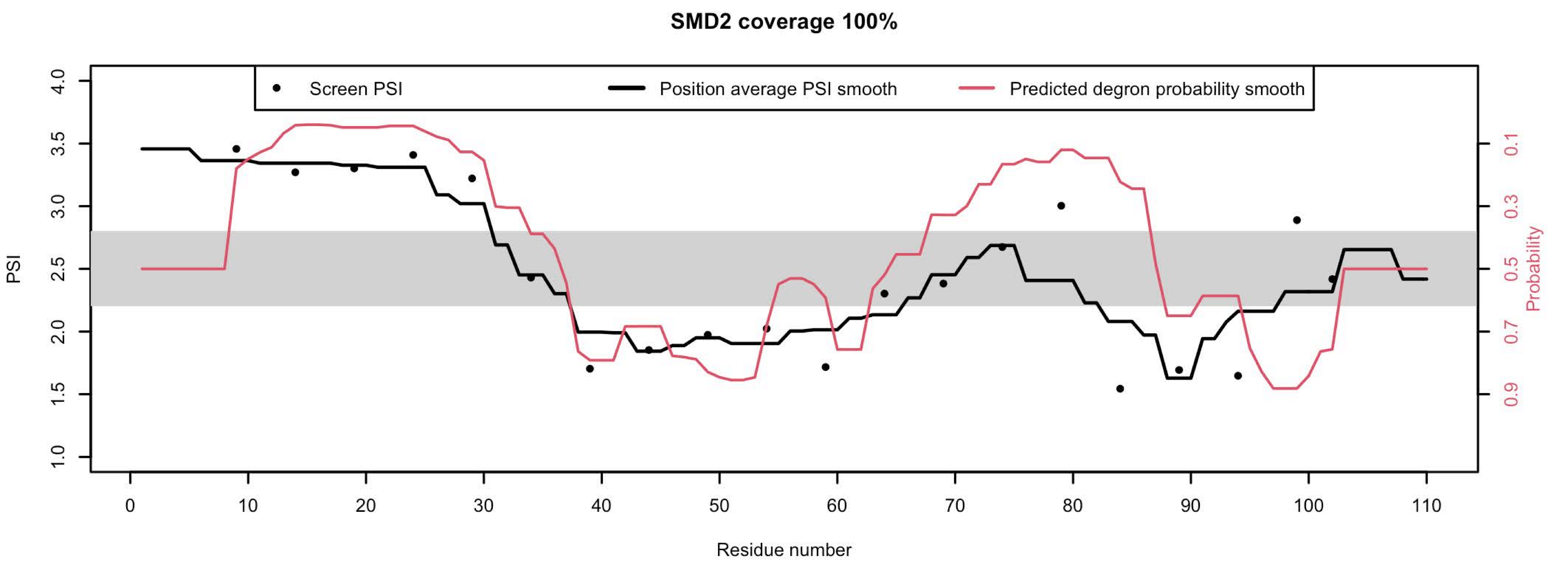
SMD2 coverage 100%
Screen PSI
Position average PSI smooth
Predicted degron probability smooth
PSI
1.0
1.5
2.0
2.5
3.0
3.5
4.0
Probability
0.1
0.3
0.5
0.7
0.9
Residue number
0
10
20
30
40
50
60
70
80
90
100
110

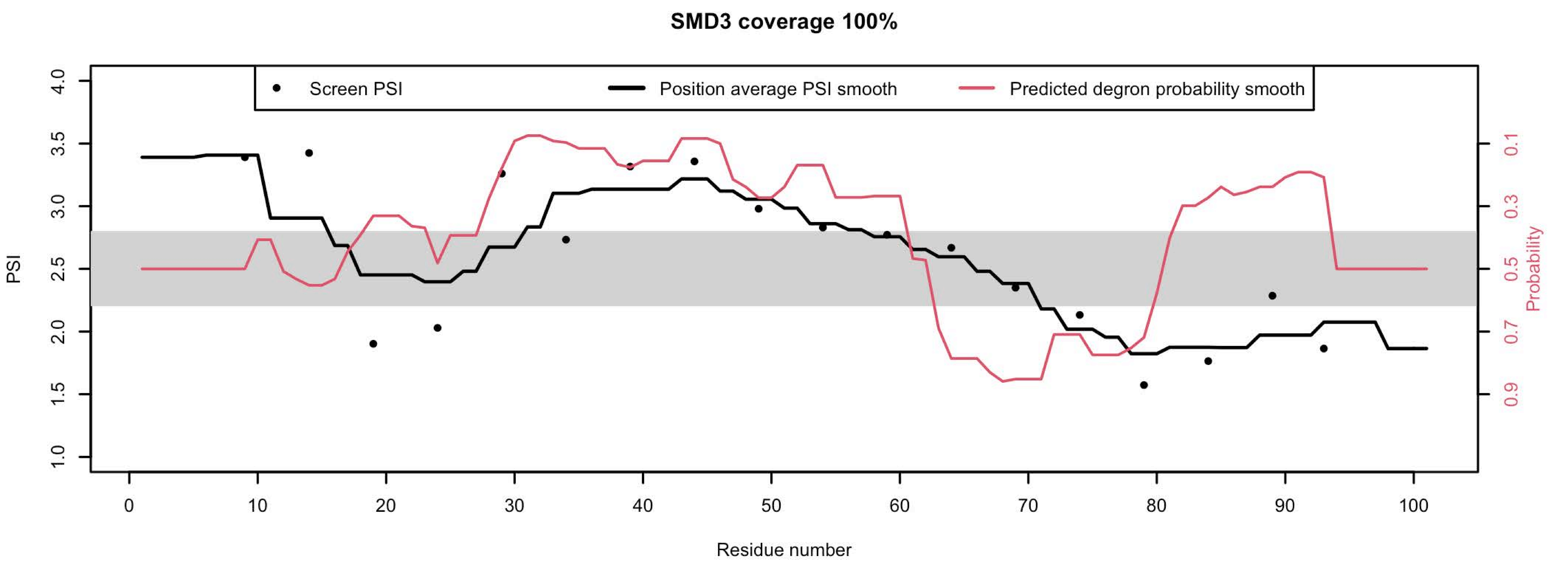
SMD3 coverage 100%
Screen PSI
Position average PSI smooth
Predicted degron probability smooth
PSI
1.0
1.5
2.0
2.5
3.0
3.5
4.0
Probability
0.1
0.3
0.5
0.7
0.9
0
10
20
30
40
50
60
70
80
90
100
Residue number

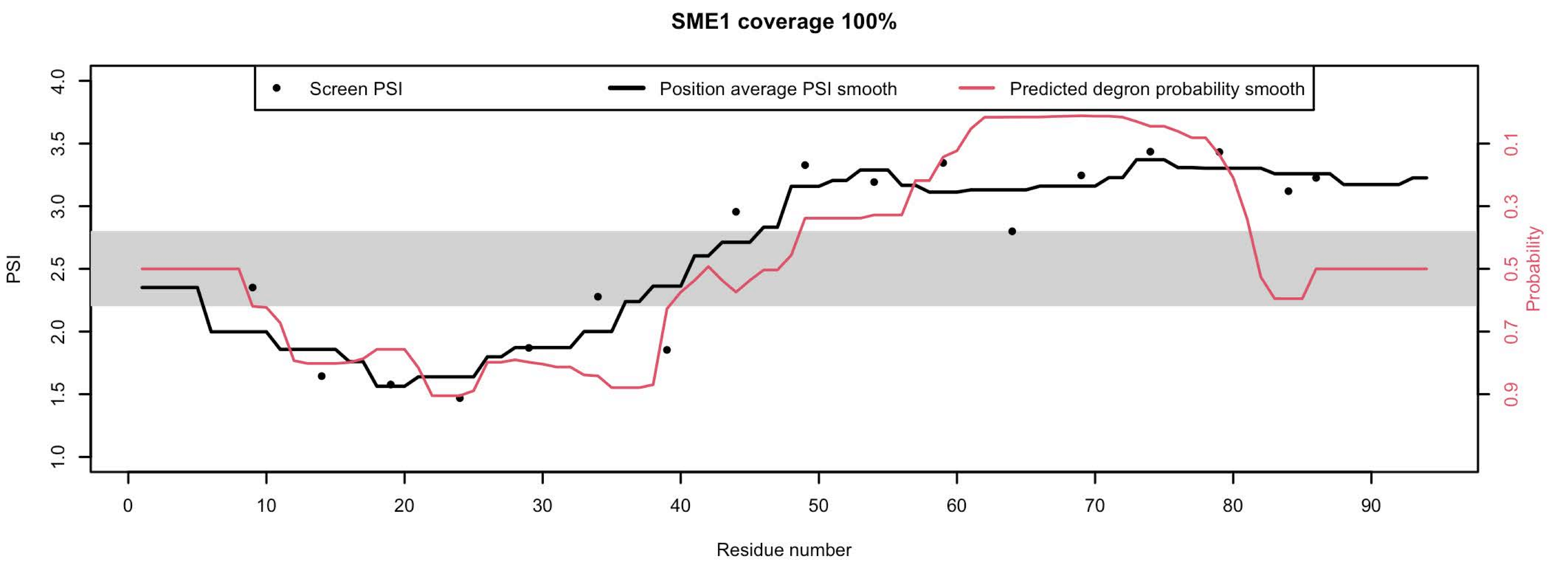
SME1 coverage 100%
Screen PSI
Position average PSI smooth
Predicted degron probability smooth
PSI
Probability
Residue number
1.0
1.5
2.0
2.5
3.0
3.5
4.0
0.1
0.3
0.5
0.7
0.9
0
10
20
30
40
50
60
70
80
90

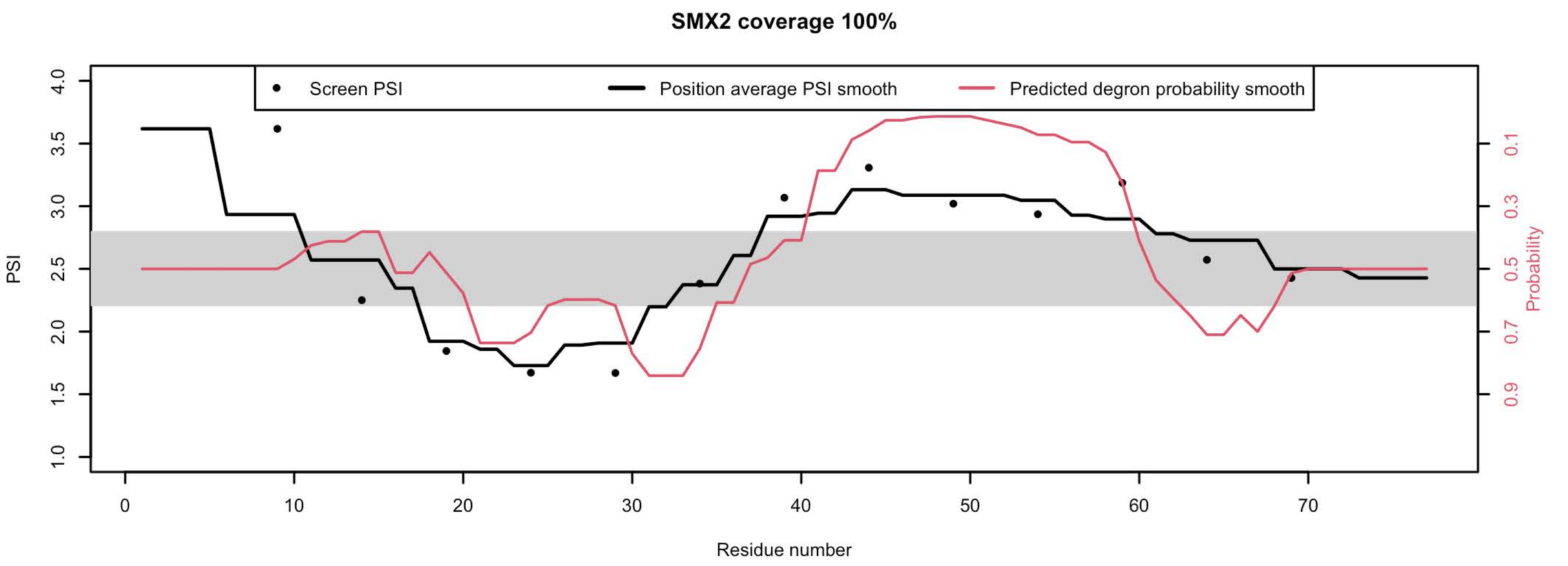

SMX2 coverage 100%
Screen PSI
Position average PSI smooth
Predicted degron probability smooth
PSI
1.0
1.5
2.0
2.5
3.0
3.5
4.0
Probability
0.1
0.3
0.5
0.7
0.9
Residue number
0
10
20
30
40
50
60
70

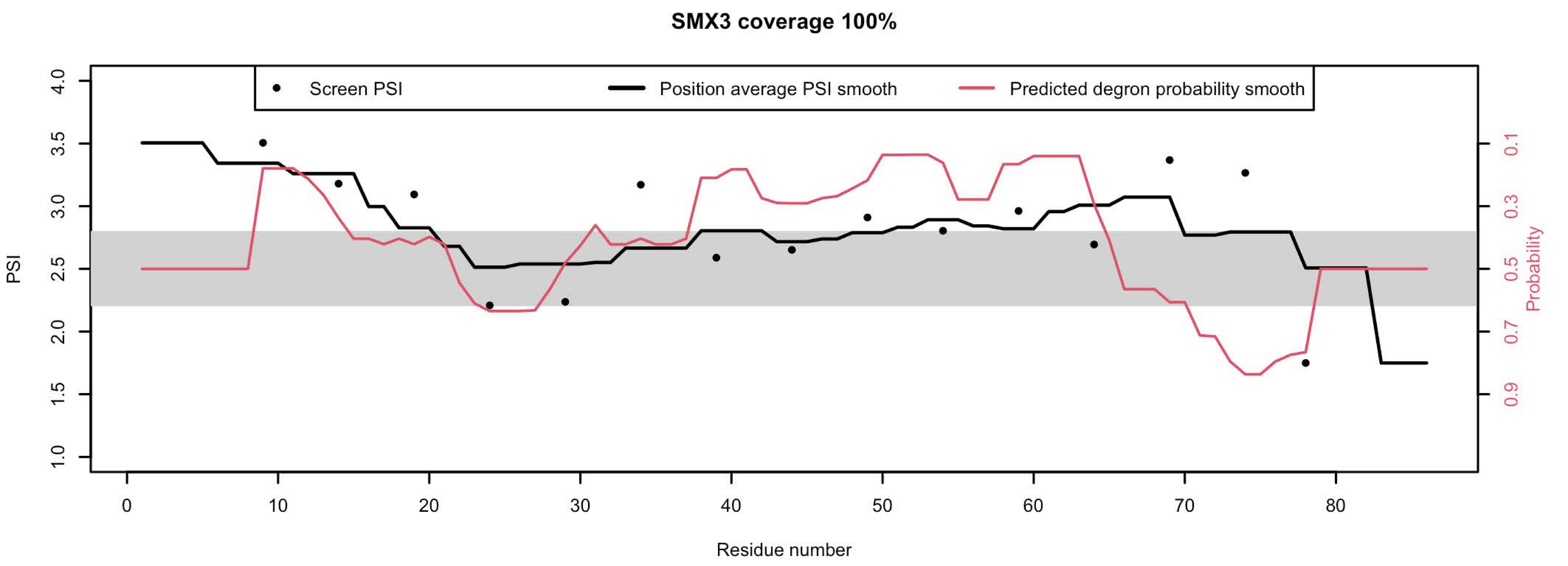

SMX3 coverage 100%
Screen PSI
Position average PSI smooth
Predicted degron probability smooth
PSI
Probability
Residue number
1.0
1.5
2.0
2.5
3.0
3.5
4.0
0.1
0.3
0.5
0.7
0.9
0
10
20
30
40
50
60
70
80

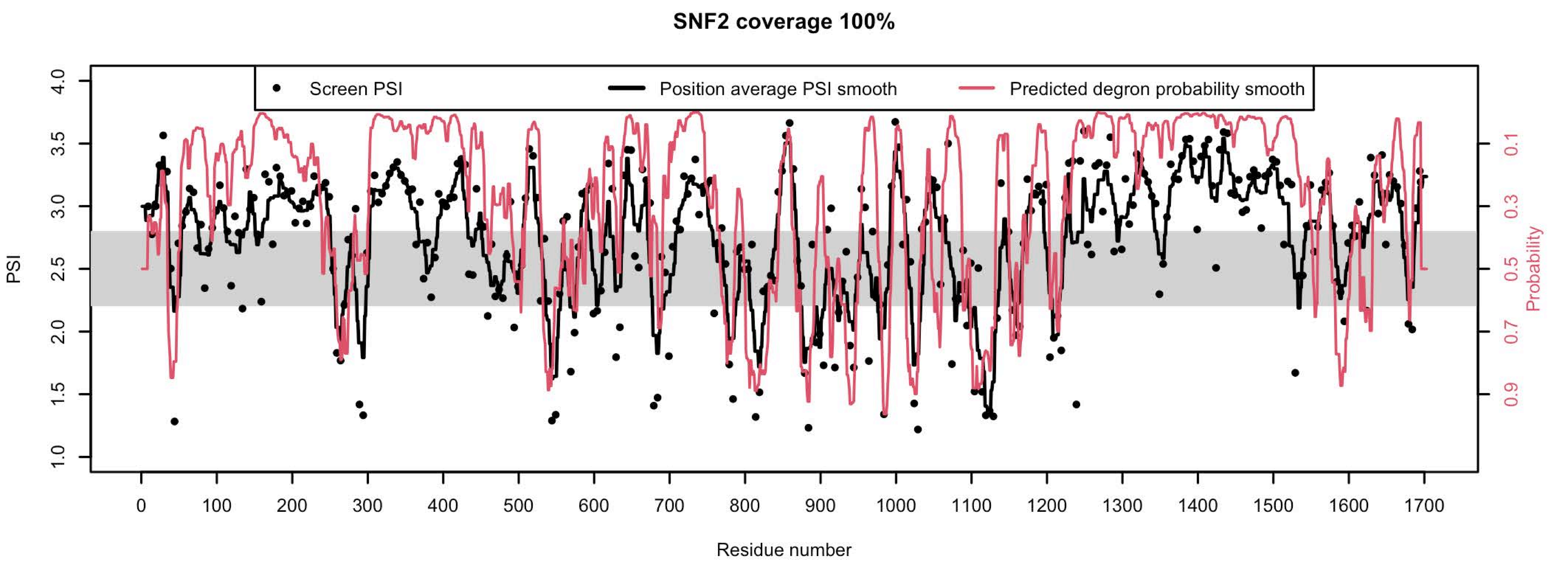

SNF2 coverage 100%
Screen PSI
Position average PSI smooth
Predicted degron probability smooth
PSI
Probability
Residue number
1.0
1.5
2.0
2.5
3.0
3.5
4.0
0.1
0.3
0.5
0.7
0.9
0
100
200
300
400
500
600
700
800
900
1000
1100
1200
1300
1400
1500
1600
1700

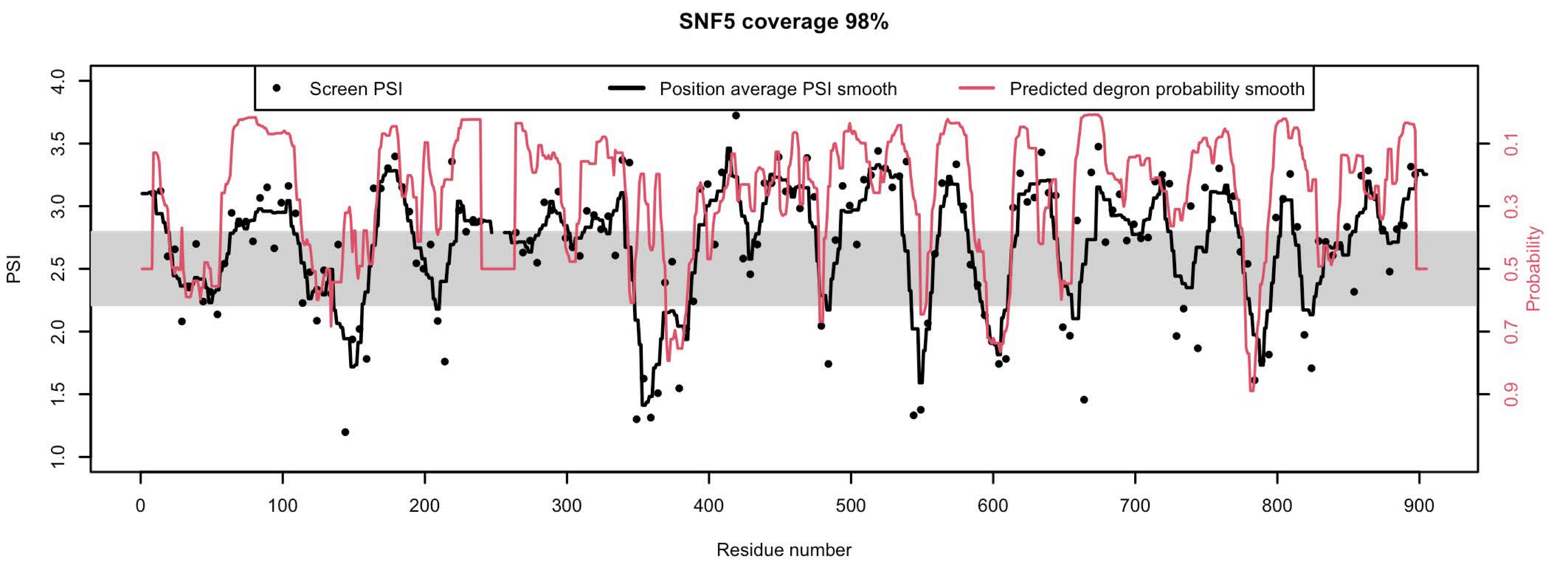

SNF5 coverage 98%
Screen PSI
Position average PSI smooth
Predicted degron probability smooth
PSI
Probability
Residue number
1.0
1.5
2.0
2.5
3.0
3.5
4.0
0.1
0.3
0.5
0.7
0.9
0
100
200
300
400
500
600
700
800
900

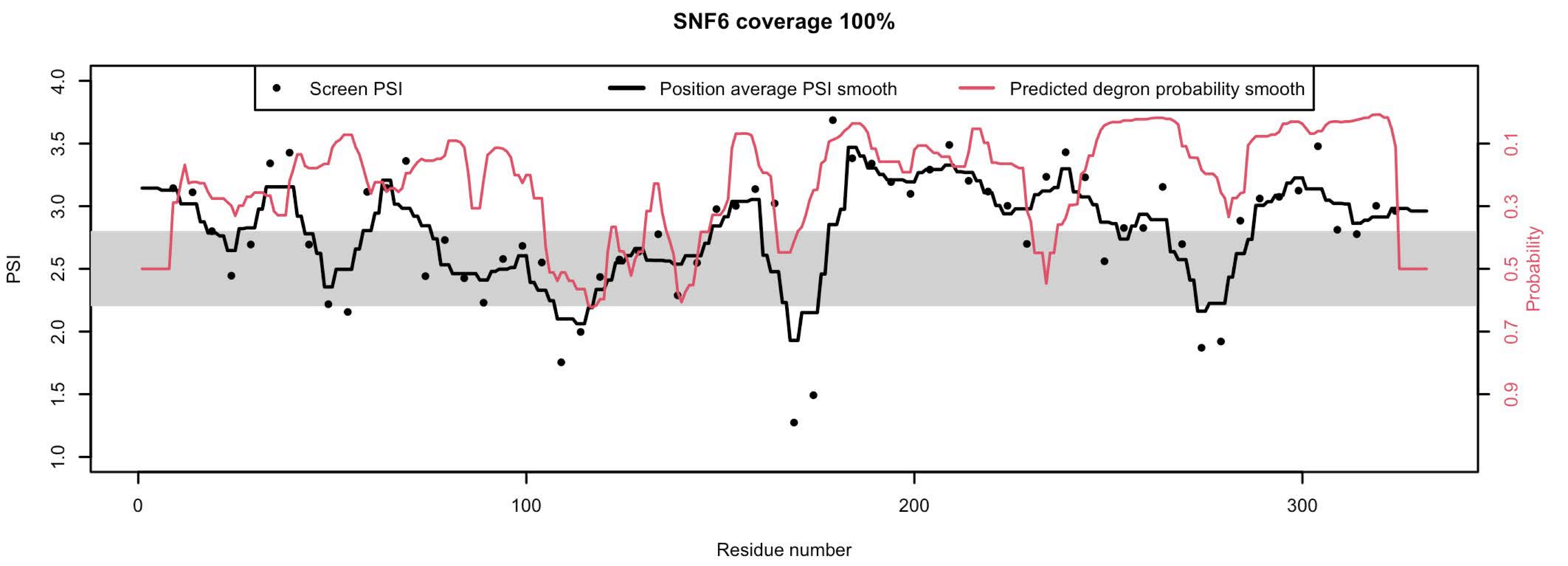

SNF6 coverage 100%
Screen PSI
Position average PSI smooth
Predicted degron probability smooth
PSI
Probability
Residue number
4.0
3.5
3.0
2.5
2.0
1.5
1.0
0.1
0.3
0.5
0.7
0.9
0
100
200
300

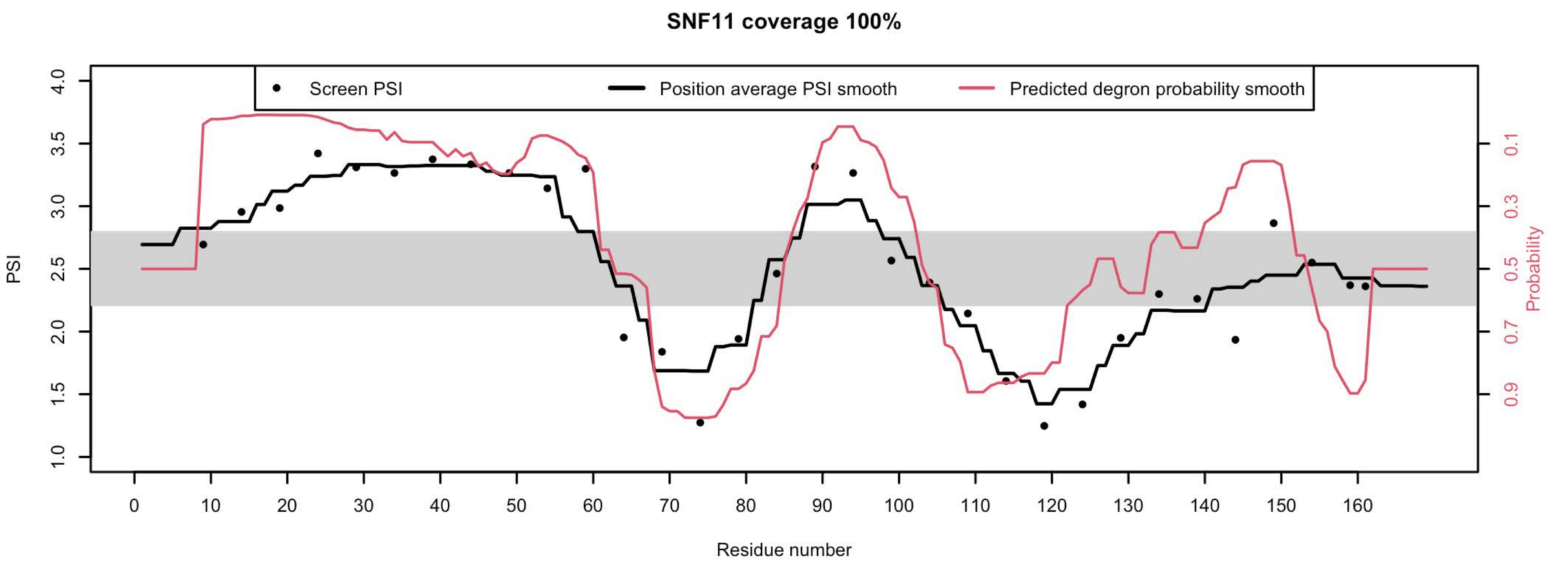
SNF11 coverage 100%
Screen PSI
Position average PSI smooth
Predicted degron probability smooth
PSI
1.0
1.5
2.0
2.5
3.0
3.5
4.0
Probability
0.1
0.3
0.5
0.7
0.9
Residue number
0
10
20
30
40
50
60
70
80
90
100
110
120
130
140
150
160

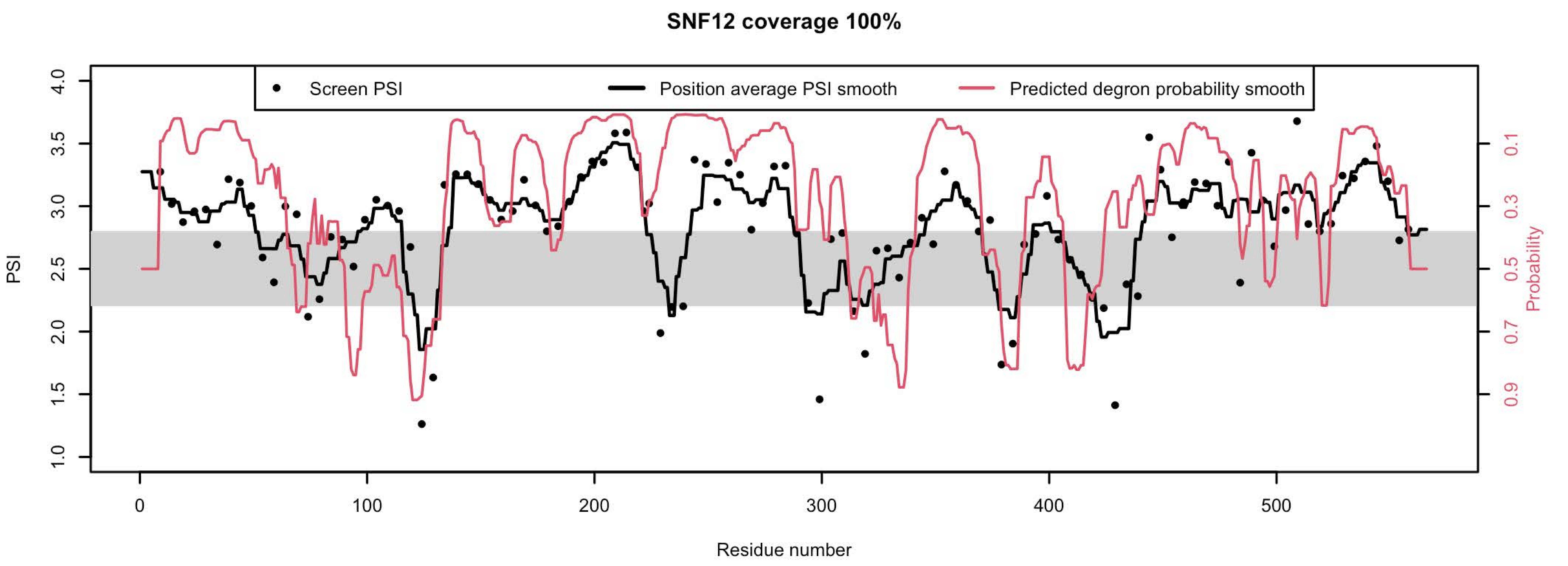

SNF12 coverage 100%
Screen PSI
Position average PSI smooth
Predicted degron probability smooth
PSI
Probability
Residue number
1.0
1.5
2.0
2.5
3.0
3.5
4.0
0.1
0.3
0.5
0.7
0.9
0
100
200
300
400
500

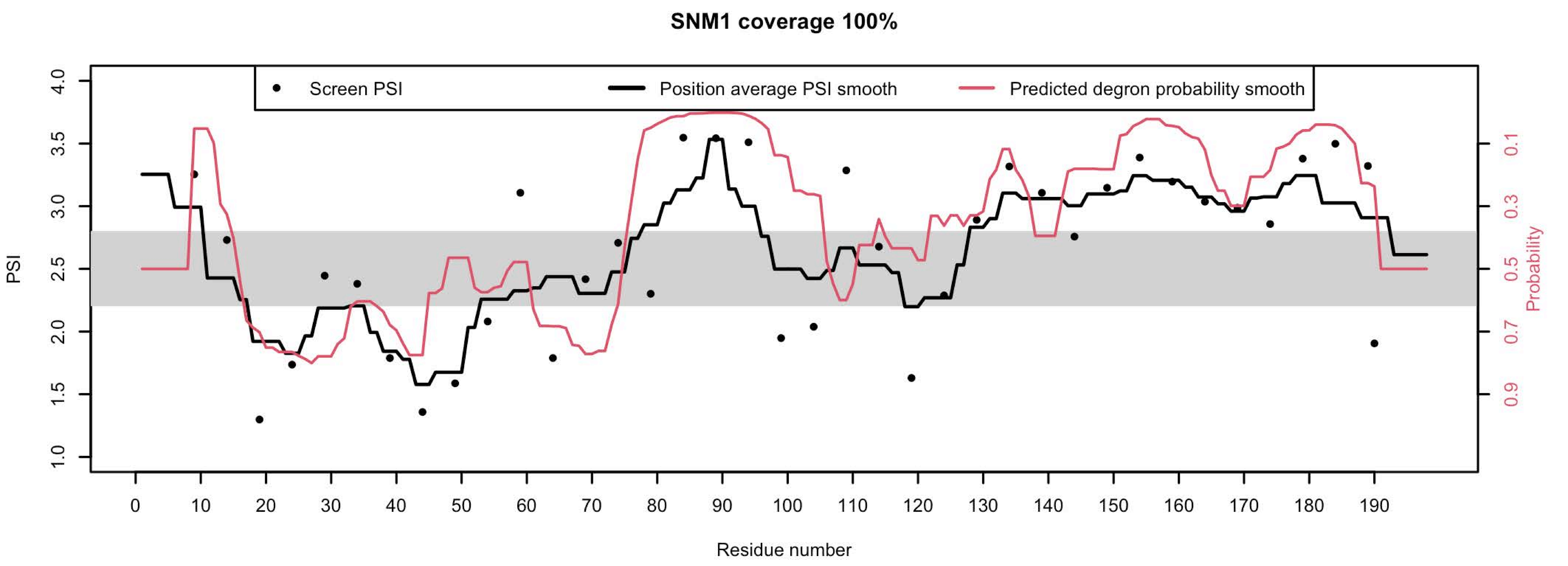
SNM1 coverage 100%
Screen PSI
Position average PSI smooth
Predicted degron probability smooth
PSI
1.0
1.5
2.0
2.5
3.0
3.5
4.0
Probability
0.1
0.3
0.5
0.7
0.9
Residue number
0
10
20
30
40
50
60
70
80
90
100
110
120
130
140
150
160
170
180
190

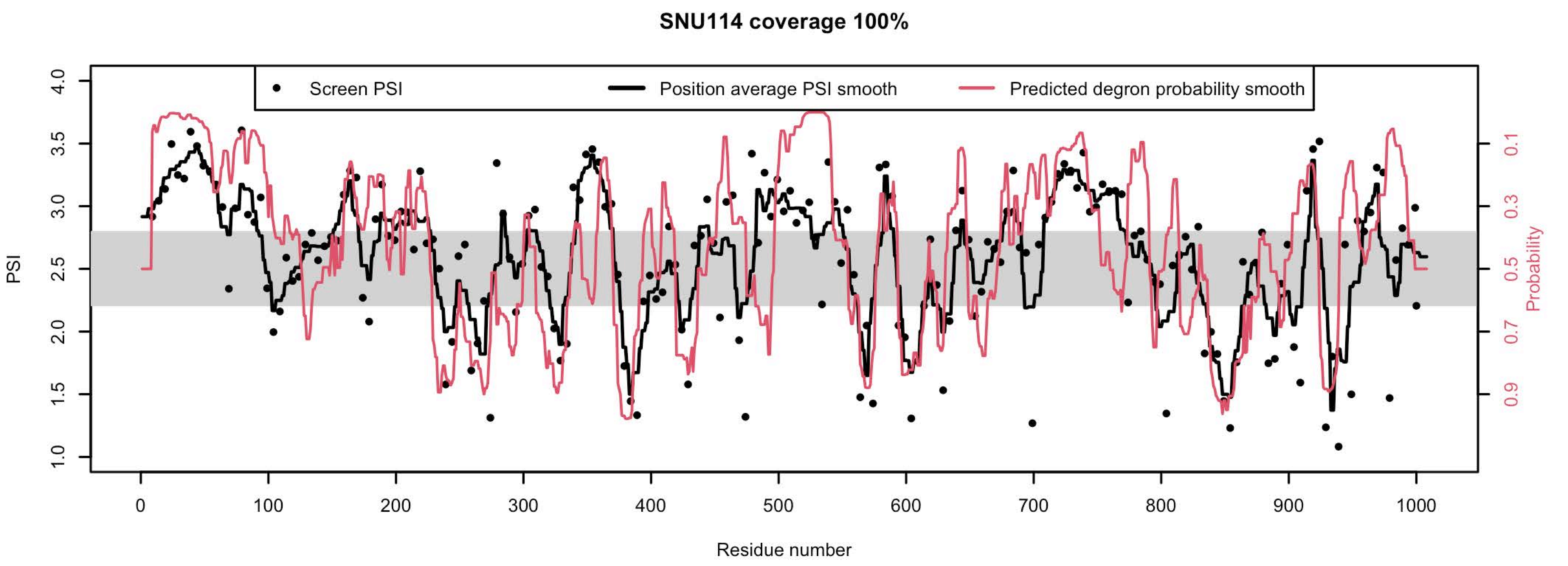
SNU114 coverage 100%
Screen PSI
Position average PSI smooth
Predicted degron probability smooth
PSI
4.0
3.5
3.0
2.5
2.0
1.5
1.0
Probability
0.1
0.3
0.5
0.7
0.9
0
100
200
300
400
500
600
700
800
900
1000
Residue number

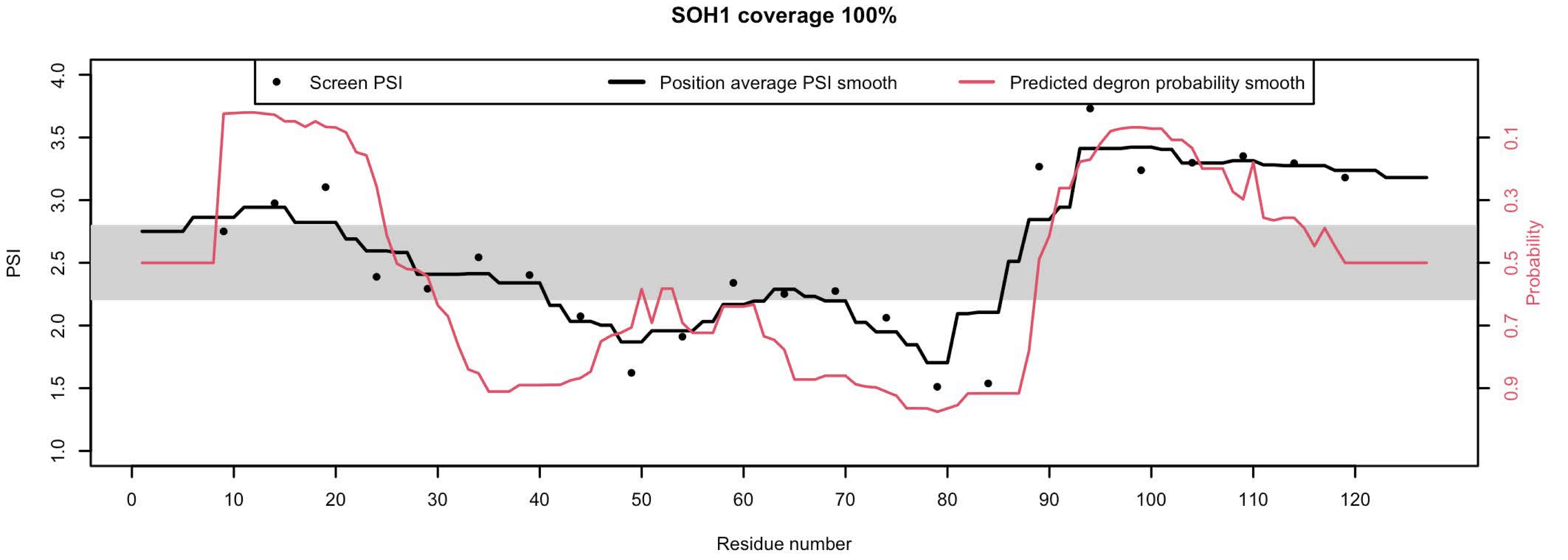

SOH1 coverage 100%
Screen PSI
Position average PSI smooth
Predicted degron probability smooth
PSI
1.0
1.5
2.0
2.5
3.0
3.5
4.0
Probability
0.1
0.3
0.5
0.7
0.9
0
10
20
30
40
50
60
70
80
90
100
110
120
Residue number

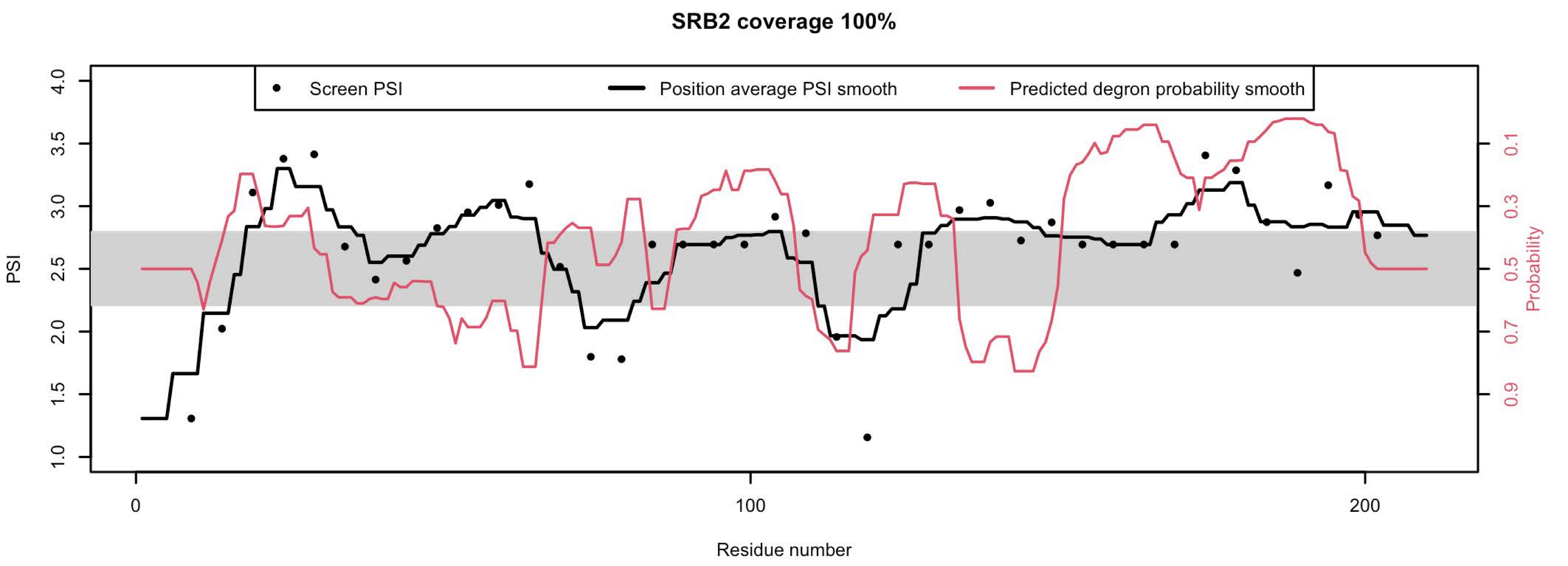

SRB2 coverage 100%
Screen PSI
Position average PSI smooth
Predicted degron probability smooth
PSI
1.0
1.5
2.0
2.5
3.0
3.5
4.0
Probability
0.1
0.3
0.5
0.7
0.9
0
100
200
Residue number

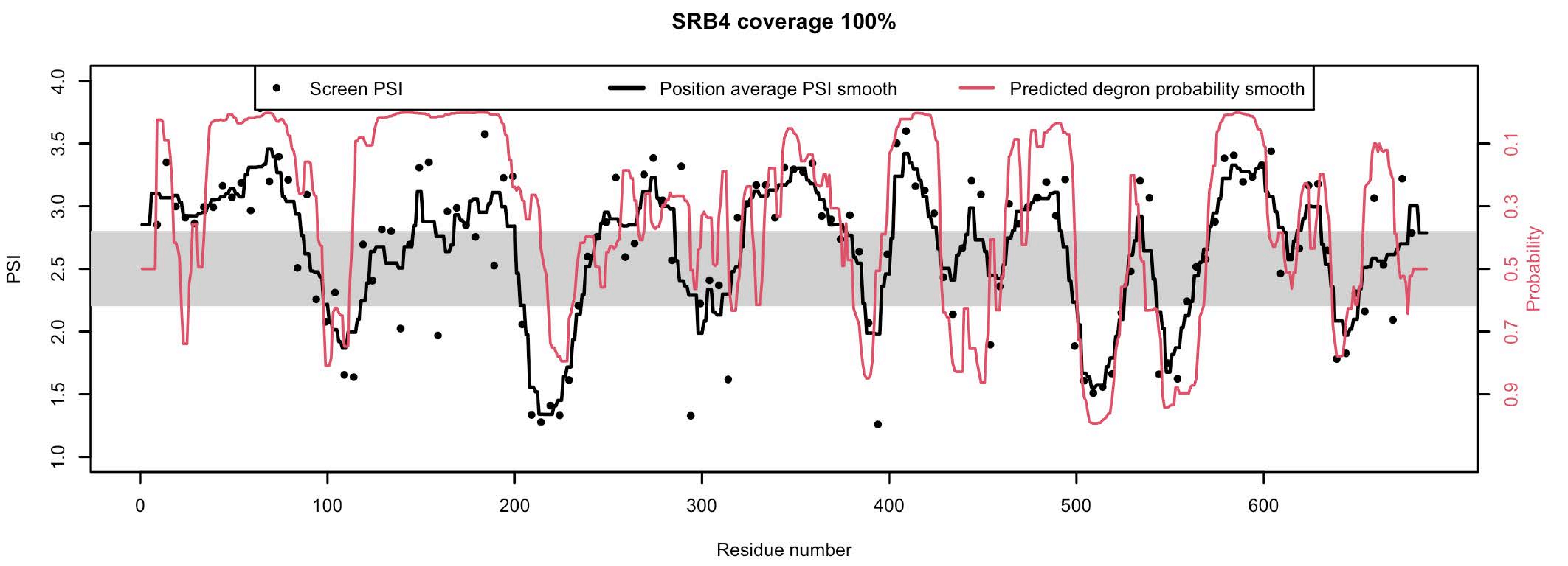

SRB4 coverage 100%
Screen PSI
Position average PSI smooth
Predicted degron probability smooth
PSI
Probability
Residue number
1.0
1.5
2.0
2.5
3.0
3.5
4.0
0.1
0.3
0.5
0.7
0.9
0
100
200
300
400
500
600

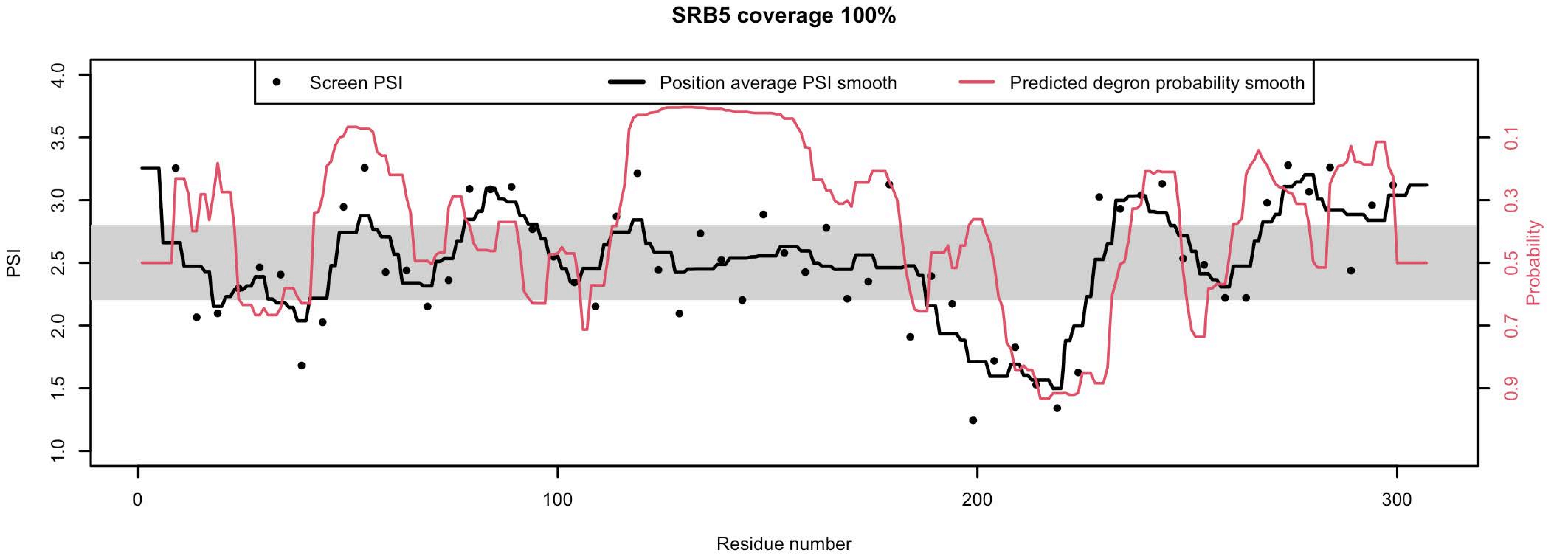

SRB5 coverage 100%
Screen PSI
Position average PSI smooth
Predicted degron probability smooth
PSI
1.0
1.5
2.0
2.5
3.0
3.5
4.0
0
100
200
300
Residue number
Probability
0.1
0.3
0.5
0.7
0.9

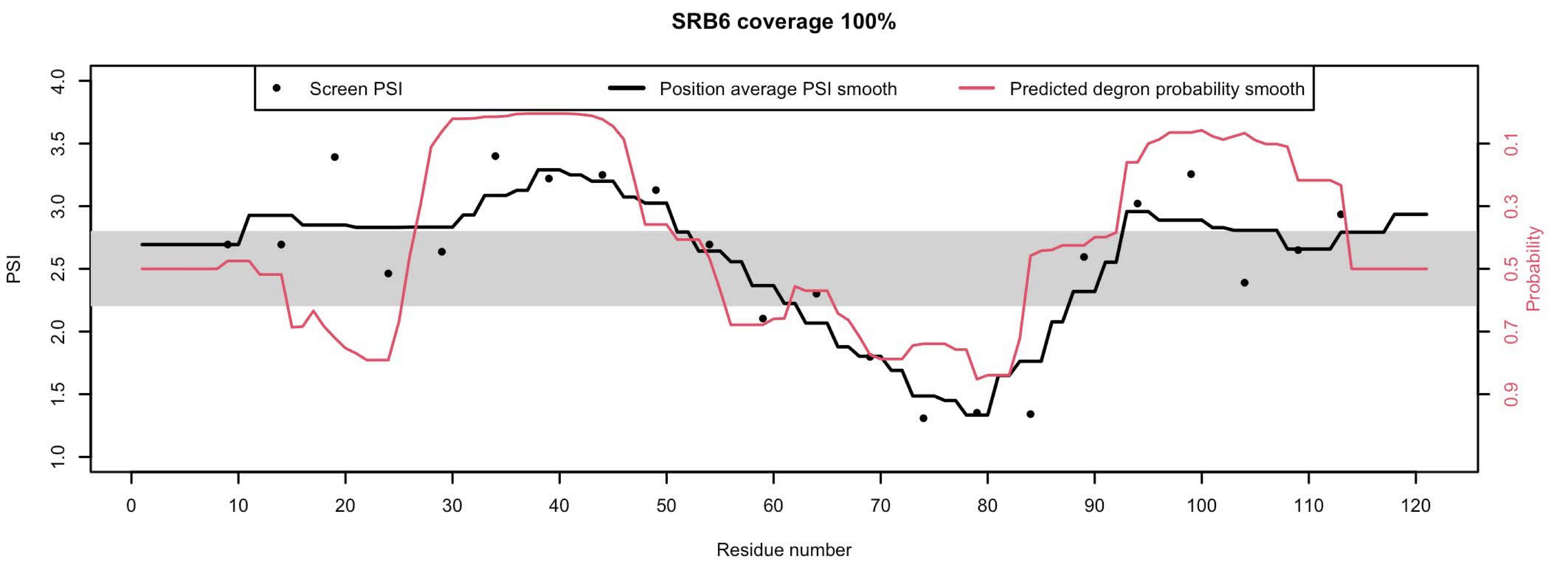
SRB6 coverage 100%
Screen PSI
Position average PSI smooth
Predicted degron probability smooth
PSI
1.0
1.5
2.0
2.5
3.0
3.5
4.0
Probability
0.1
0.3
0.5
0.7
0.9
Residue number
0
10
20
30
40
50
60
70
80
90
100
110
120

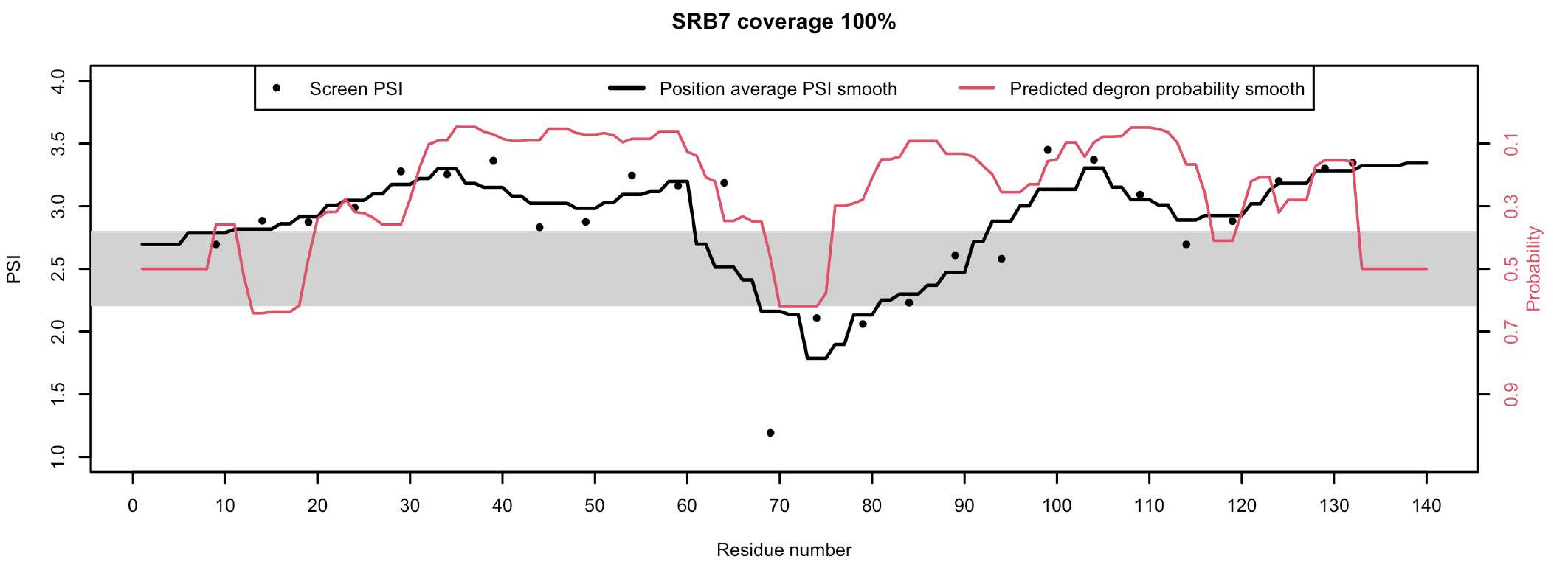

SRB7 coverage 100%
Screen PSI
Position average PSI smooth
Predicted degron probability smooth
PSI
Probability
Residue number
1.0
1.5
2.0
2.5
3.0
3.5
4.0
0.1
0.3
0.5
0.7
0.9
0
10
20
30
40
50
60
70
80
90
100
110
120
130
140

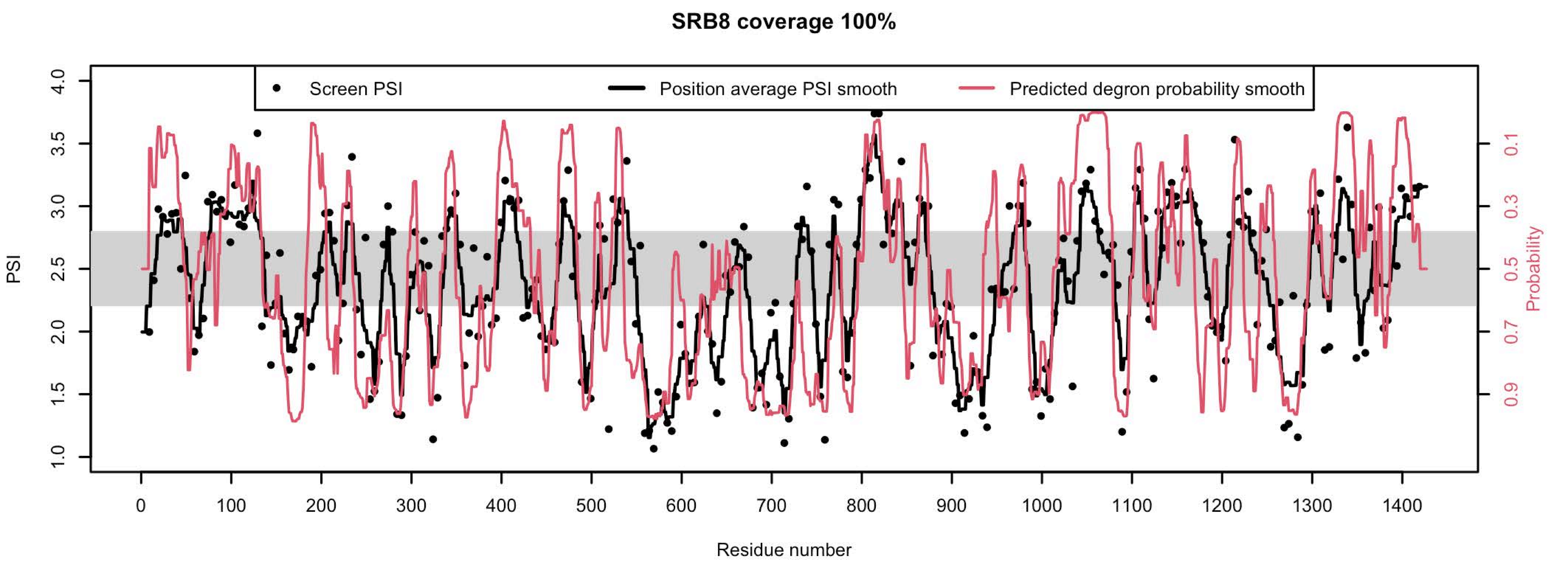

SRB8 coverage 100%
Screen PSI
Position average PSI smooth
Predicted degron probability smooth
PSI
1.0
1.5
2.0
2.5
3.0
3.5
4.0
Probability
0.1
0.3
0.5
0.7
0.9
Residue number
0
100
200
300
400
500
600
700
800
900
1000
1100
1200
1300
1400

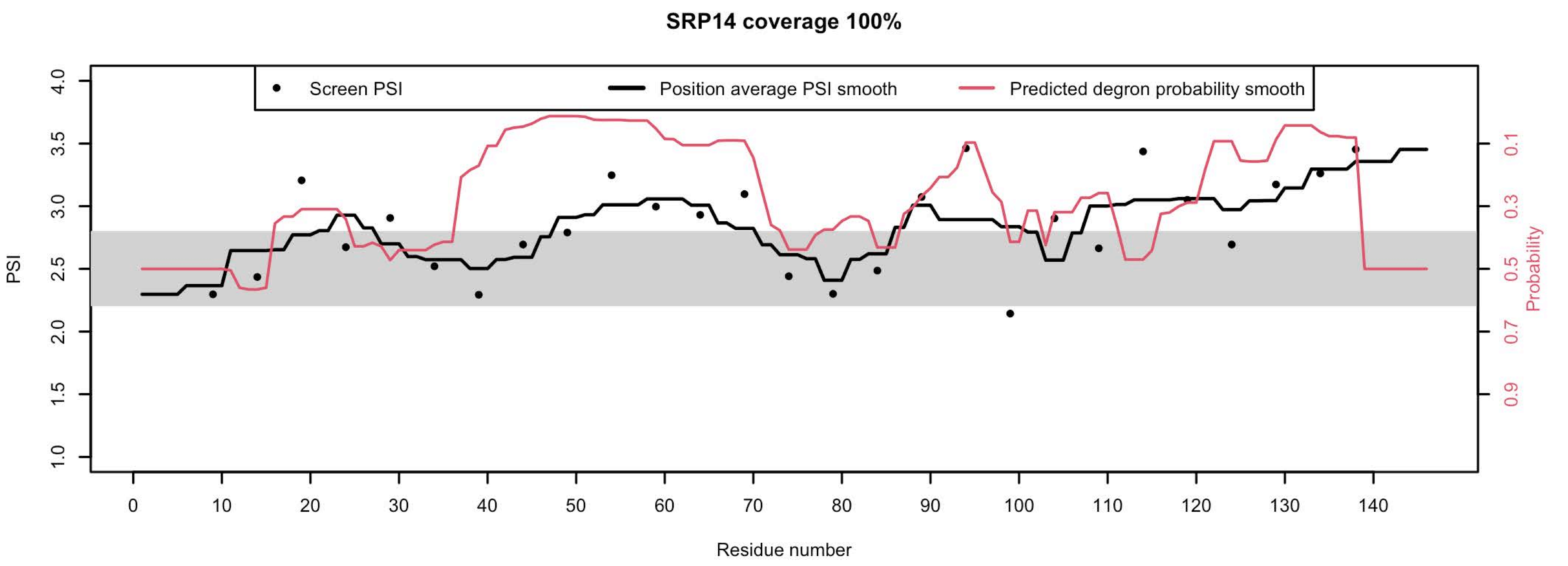

SRP14 coverage 100%
Screen PSI
Position average PSI smooth
Predicted degron probability smooth
PSI
1.0
1.5
2.0
2.5
3.0
3.5
4.0
Probability
0.1
0.3
0.5
0.7
0.9
Residue number
0
10
20
30
40
50
60
70
80
90
100
110
120
130
140

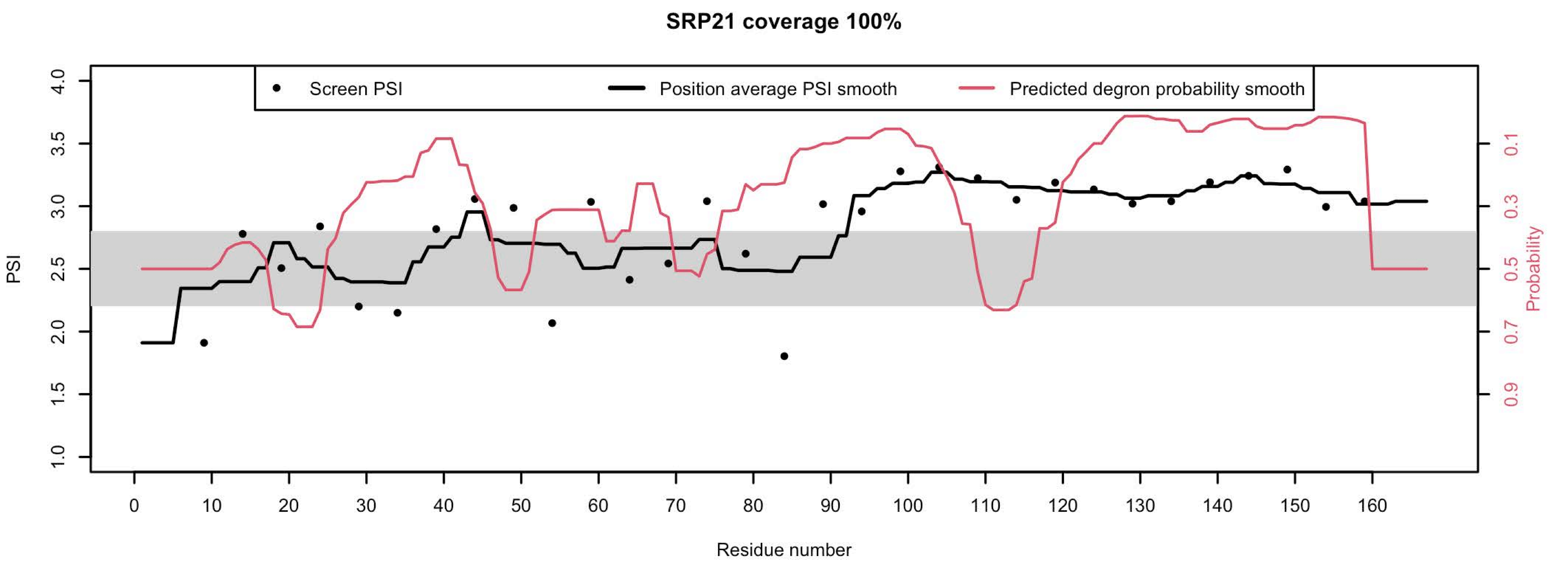

SRP21 coverage 100%
Screen PSI
Position average PSI smooth
Predicted degron probability smooth
PSI
1.0
1.5
2.0
2.5
3.0
3.5
4.0
Probability
0.1
0.3
0.5
0.7
0.9
0
10
20
30
40
50
60
70
80
90
100
110
120
130
140
150
160
Residue number

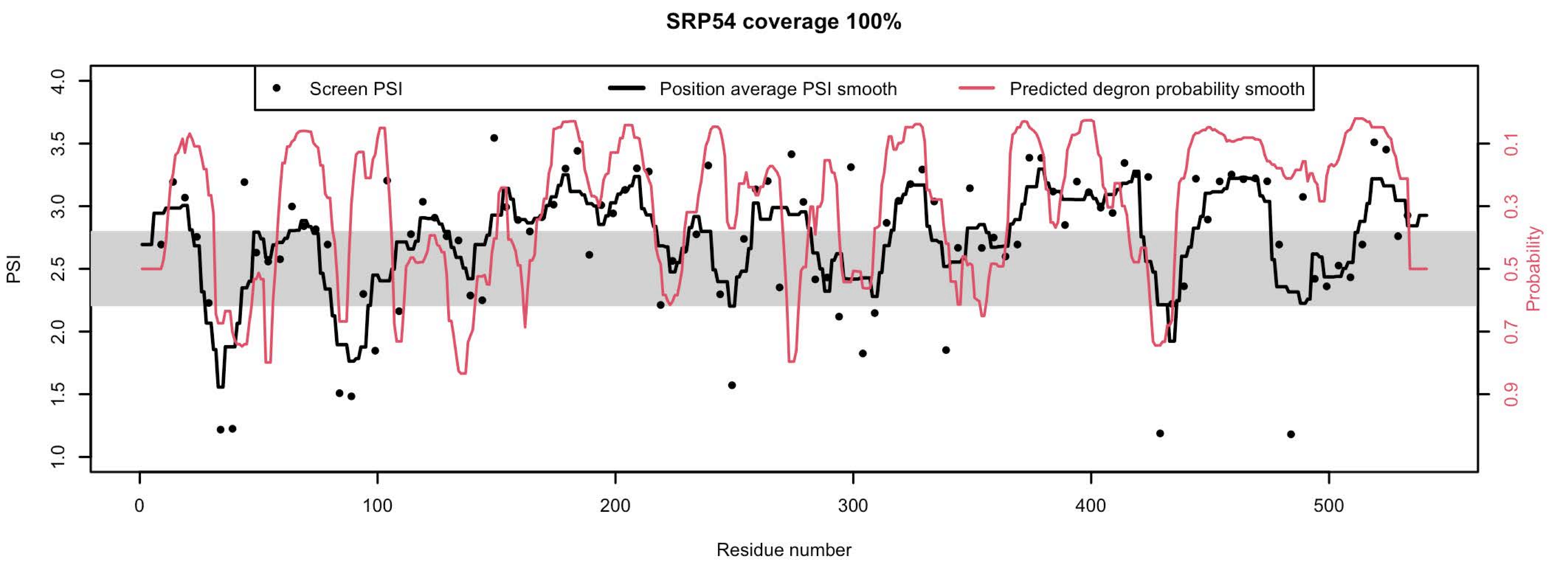

SRP54 coverage 100%
Screen PSI
Position average PSI smooth
Predicted degron probability smooth
PSI
Probability
Residue number
1.0
1.5
2.0
2.5
3.0
3.5
4.0
0.1
0.3
0.5
0.7
0.9
0
100
200
300
400
500

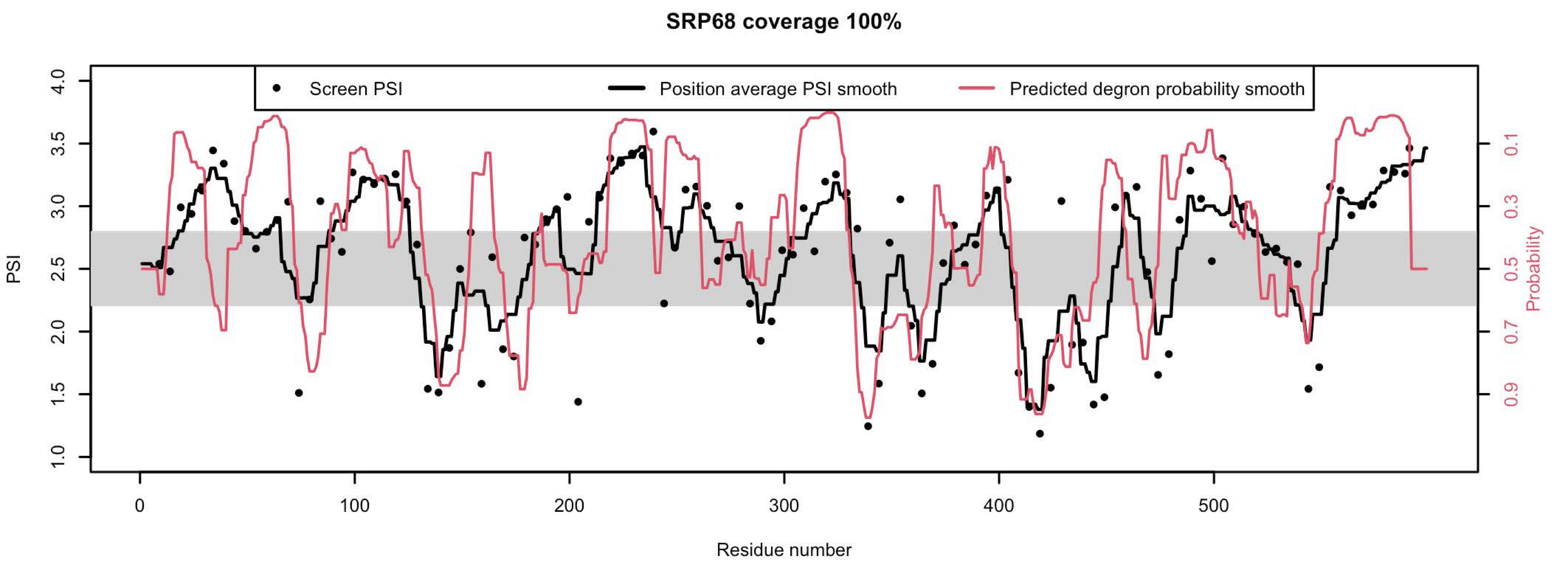

SRP68 coverage 100%
Screen PSI
Position average PSI smooth
Predicted degron probability smooth
PSI
1.0
1.5
2.0
2.5
3.0
3.5
4.0
0
100
200
300
400
500
Residue number
Probability
0.1
0.3
0.5
0.7
0.9

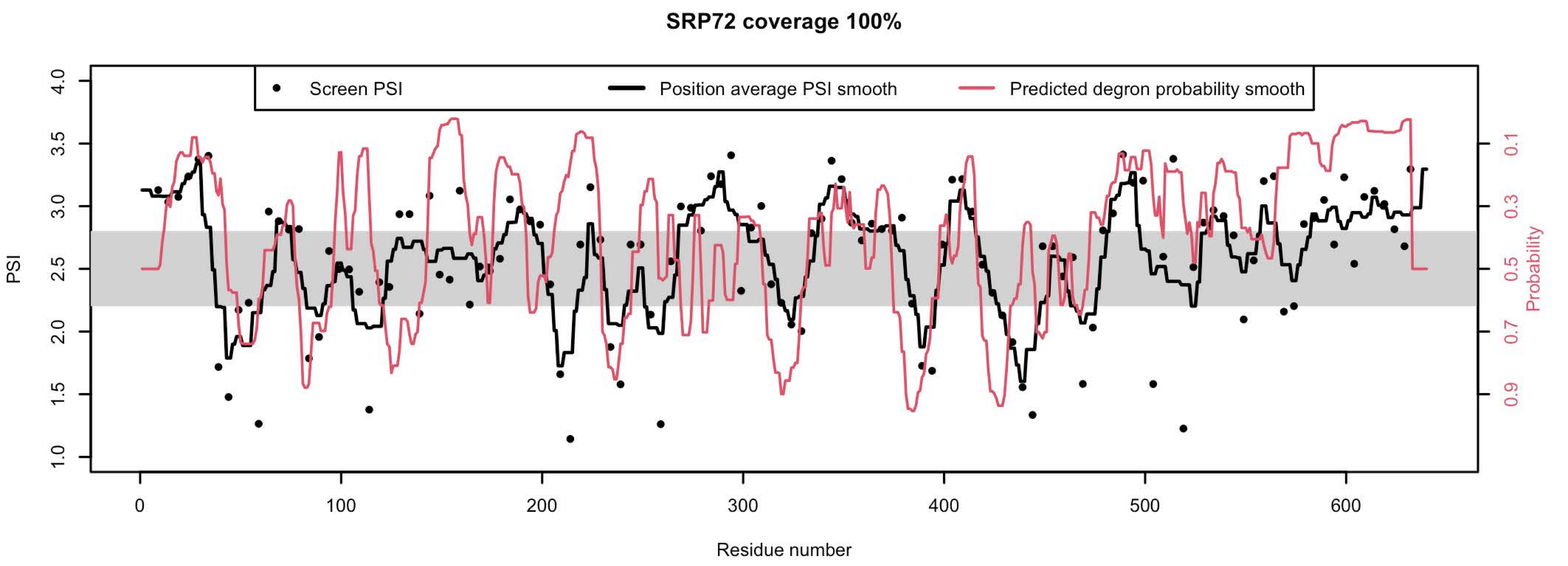

SRP72 coverage 100%
Screen PSI
Position average PSI smooth
Predicted degron probability smooth
PSI
Probability
Residue number
1.0
1.5
2.0
2.5
3.0
3.5
4.0
0.1
0.3
0.5
0.7
0.9
0
100
200
300
400
500
600

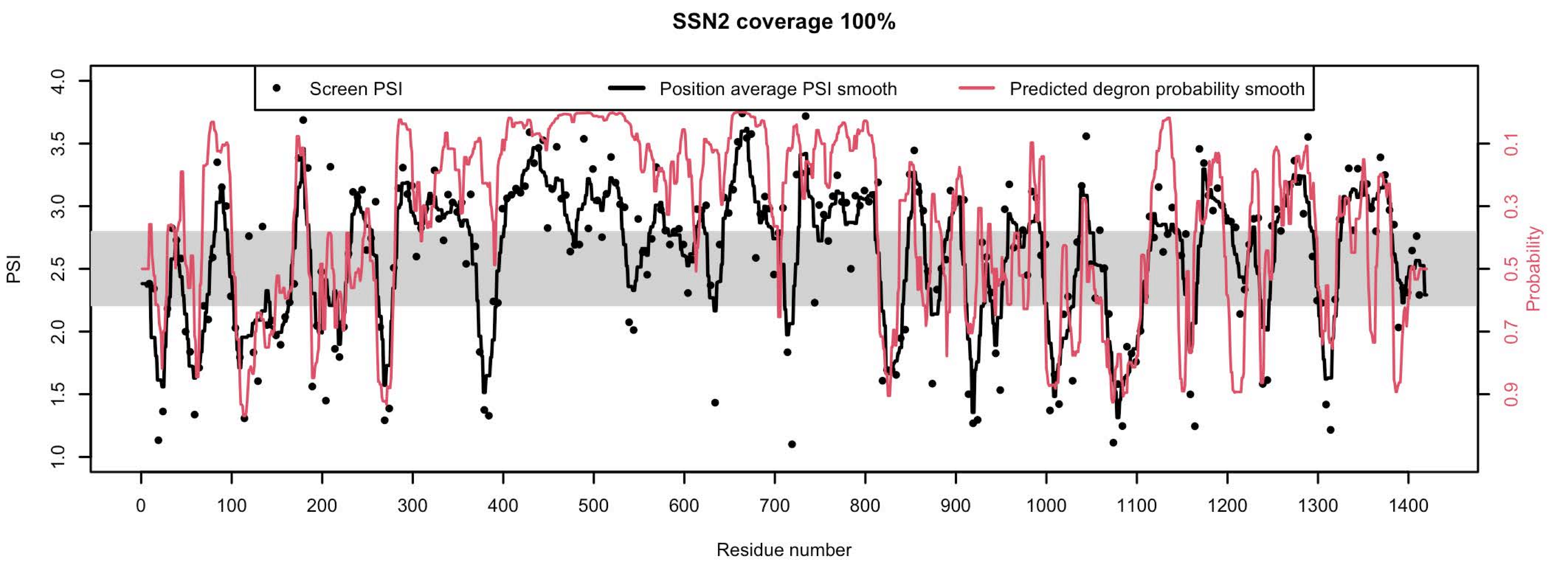

SSN2 coverage 100%
Screen PSI
Position average PSI smooth
Predicted degron probability smooth
PSI
1.0
1.5
2.0
2.5
3.0
3.5
4.0
Probability
0.1
0.3
0.5
0.7
0.9
0
100
200
300
400
500
600
700
800
900
1000
1100
1200
1300
1400
Residue number

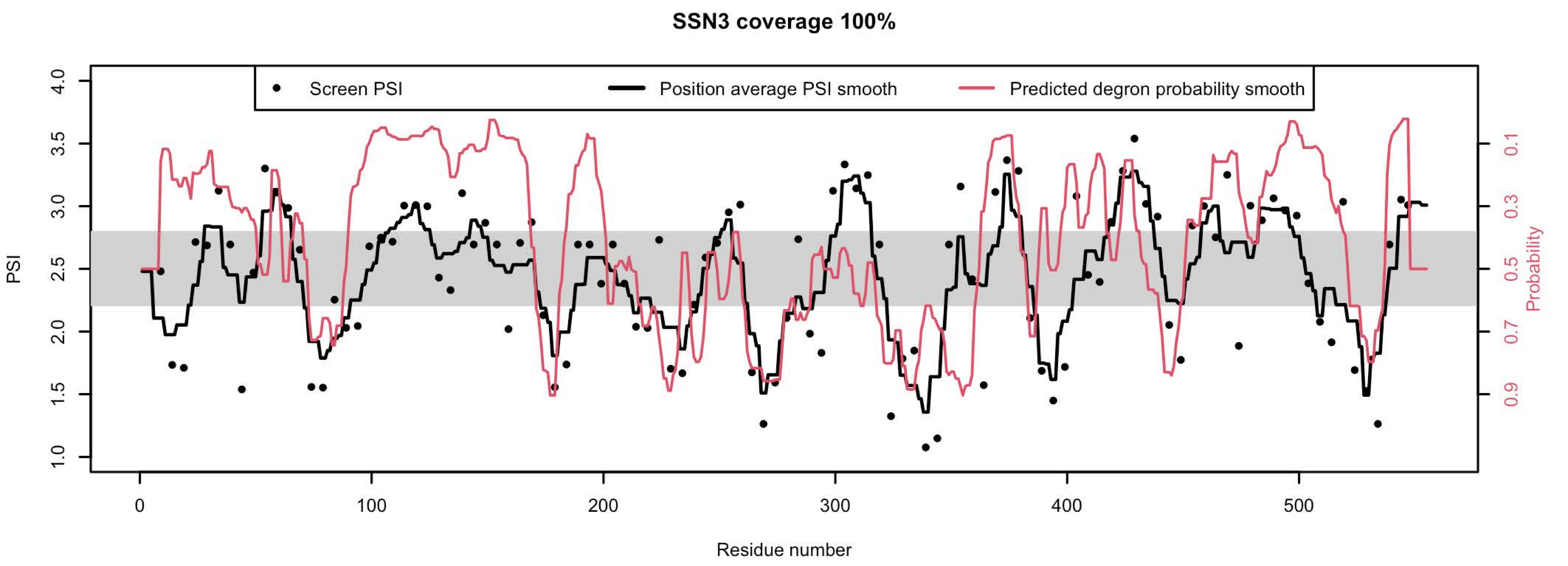

SSN3 coverage 100%
Screen PSI
Position average PSI smooth
Predicted degron probability smooth
PSI
1.0
1.5
2.0
2.5
3.0
3.5
4.0
Probability
0.1
0.3
0.5
0.7
0.9
0
100
200
300
400
500
Residue number

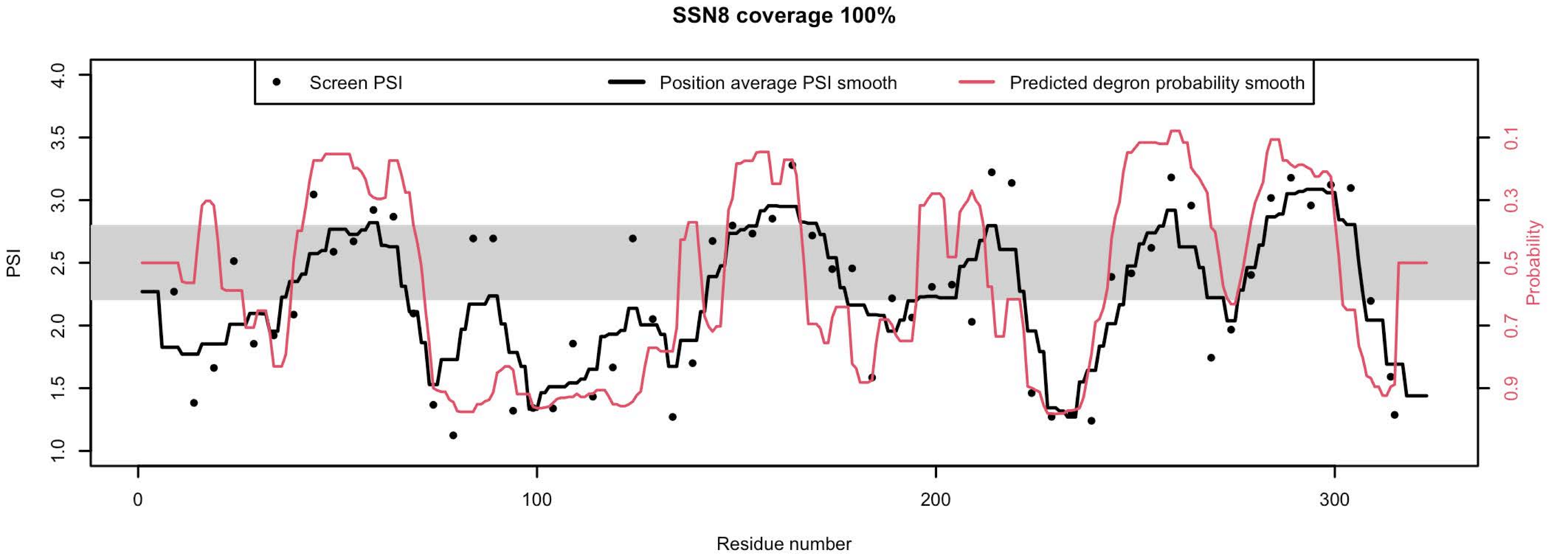

SSN8 coverage 100%
Screen PSI
Position average PSI smooth
Predicted degron probability smooth
PSI
1.0
1.5
2.0
2.5
3.0
3.5
4.0
Probability
0.1
0.3
0.5
0.7
0.9
0
100
200
300
Residue number

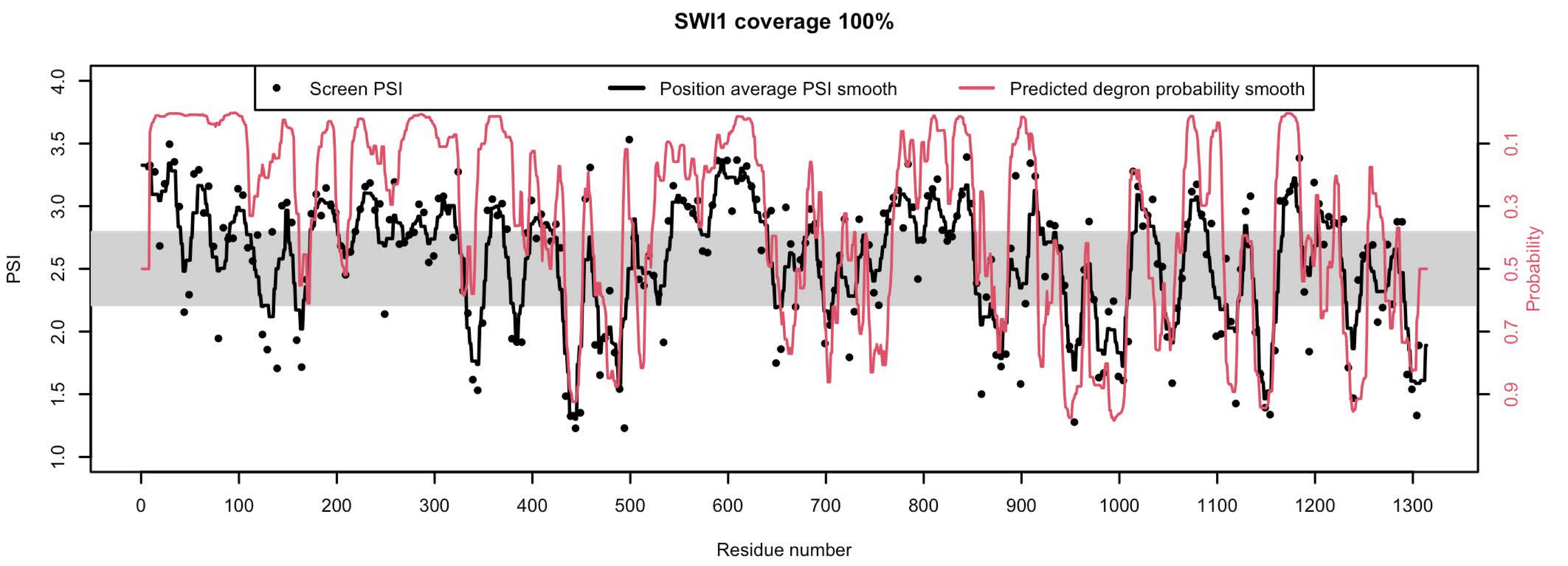
SWI1 coverage 100%
Screen PSI
Position average PSI smooth
Predicted degron probability smooth
PSI
1.0
1.5
2.0
2.5
3.0
3.5
4.0
Probability
0.1
0.3
0.5
0.7
0.9
0
100
200
300
400
500
600
700
800
900
1000
1100
1200
1300
Residue number

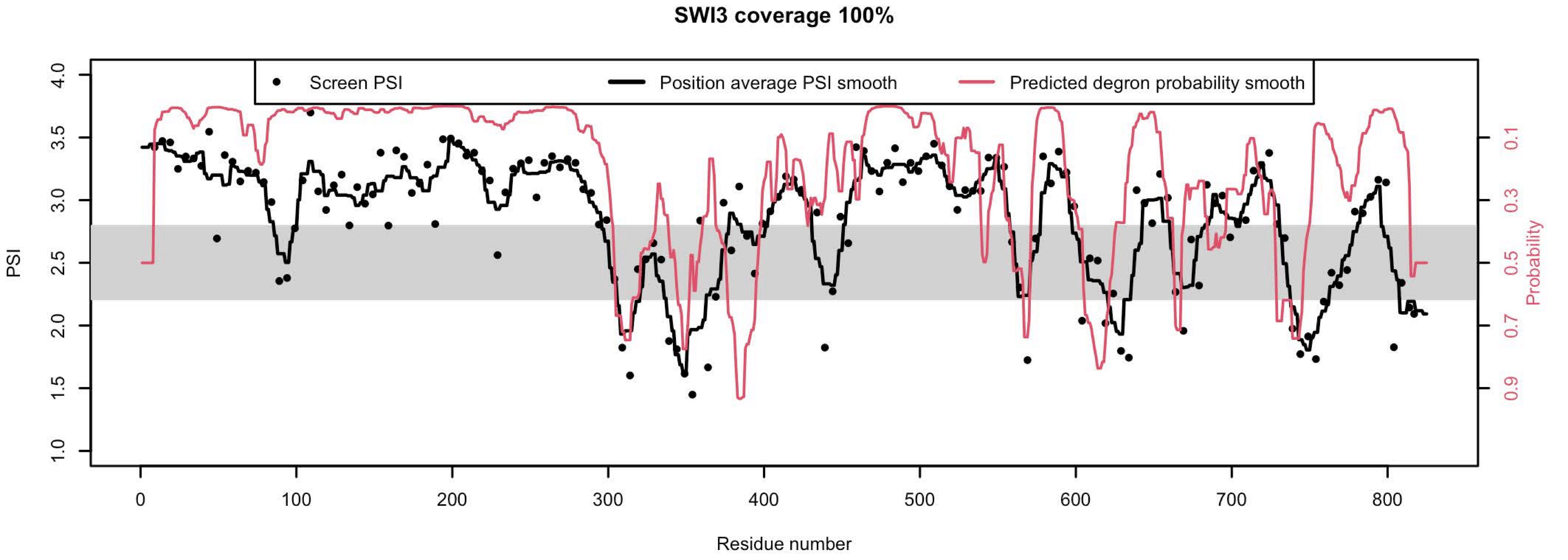

SWI3 coverage 100%
Screen PSI
Position average PSI smooth
Predicted degron probability smooth
PSI
Probability
Residue number
4.0
3.5
3.0
2.5
2.0
1.5
1.0
0.1
0.3
0.5
0.7
0.9
0
100
200
300
400
500
600
700
800

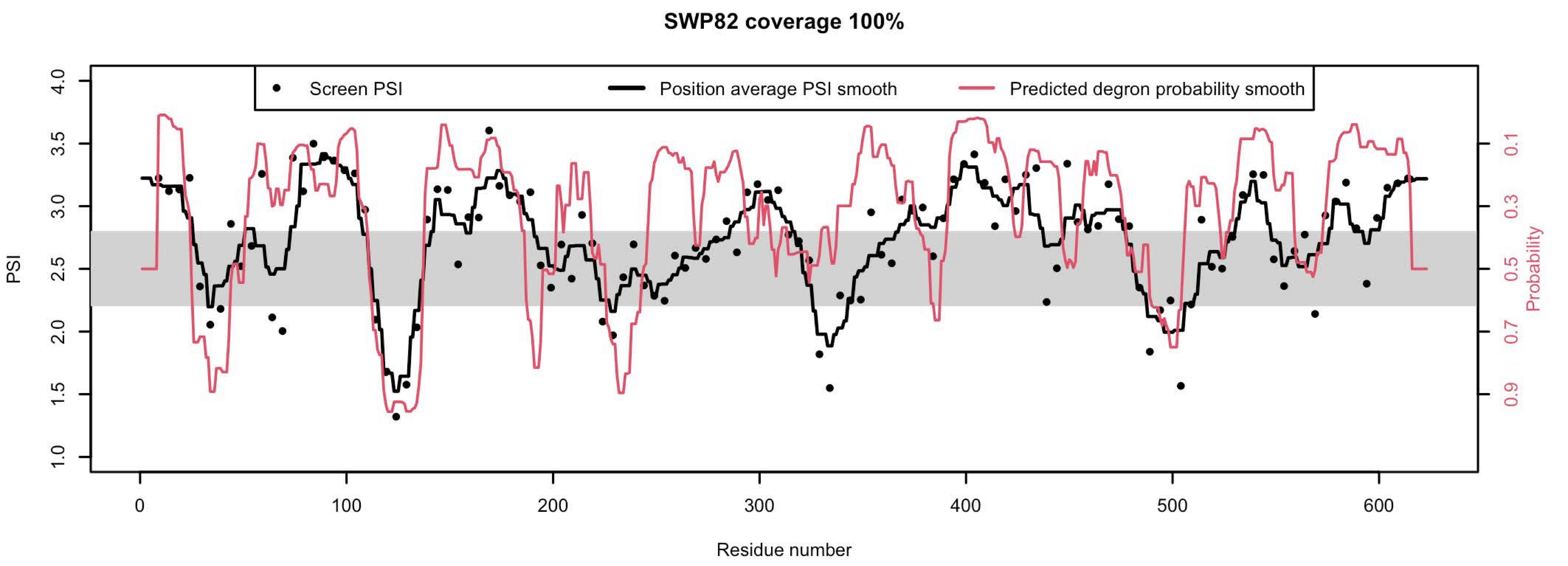

SWP82 coverage 100%
Screen PSI
Position average PSI smooth
Predicted degron probability smooth
PSI
Probability
Residue number
1.0
1.5
2.0
2.5
3.0
3.5
4.0
0.1
0.3
0.5
0.7
0.9
0
100
200
300
400
500
600

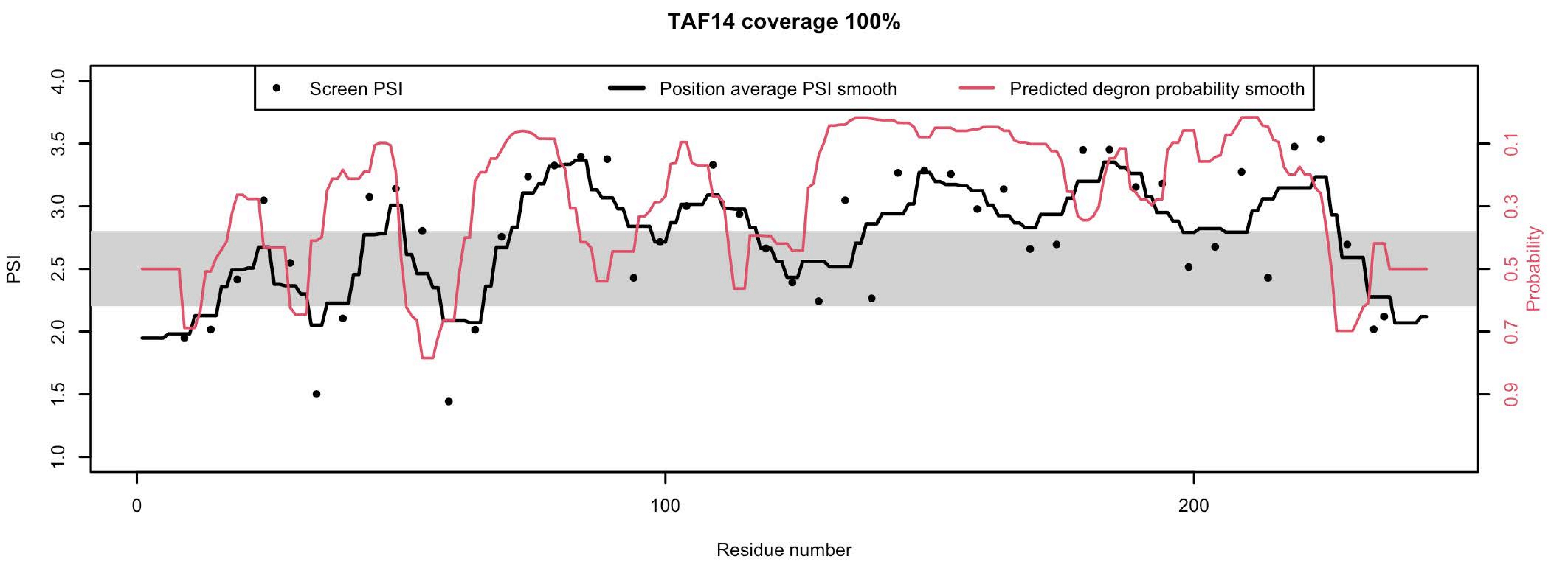
TAF14 coverage 100%
Screen PSI
Position average PSI smooth
Predicted degron probability smooth
PSI
1.0
1.5
2.0
2.5
3.0
3.5
4.0
Probability
0.9
0.7
0.5
0.3
0.1
0
100
200
Residue number

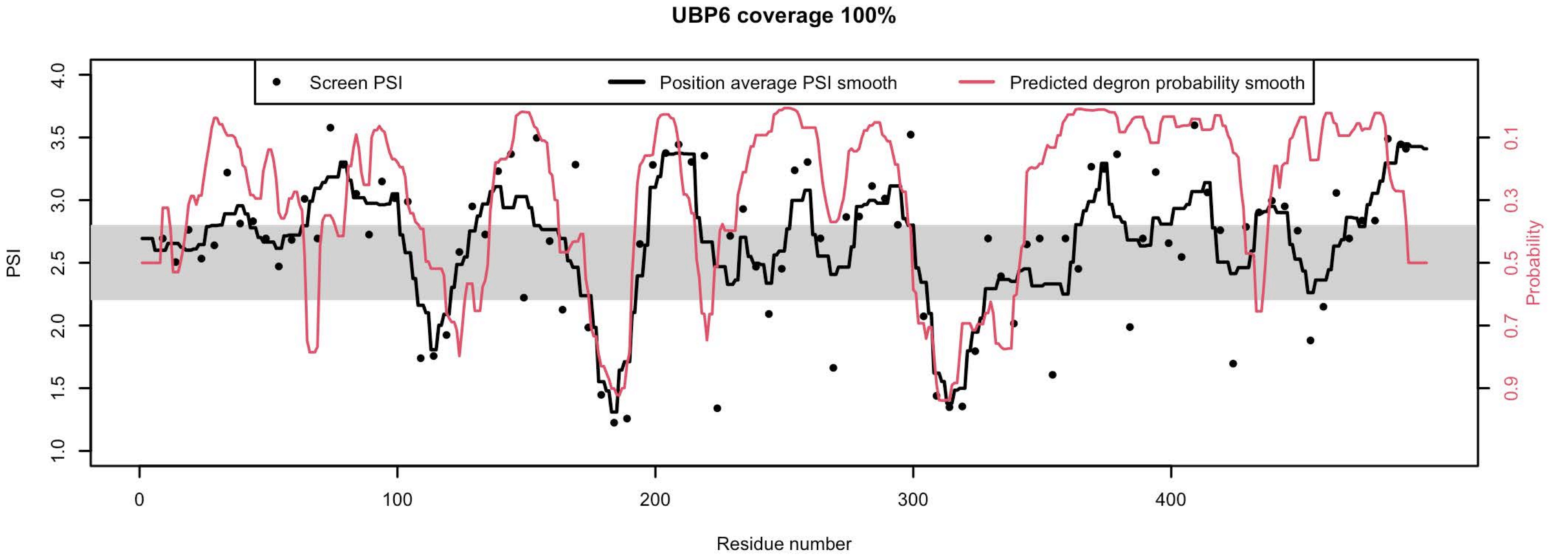

UBP6 coverage 100%
Screen PSI
Position average PSI smooth
Predicted degron probability smooth
PSI
Probability
Residue number
1.0
1.5
2.0
2.5
3.0
3.5
4.0
0.1
0.3
0.5
0.7
0.9
0
100
200
300
400

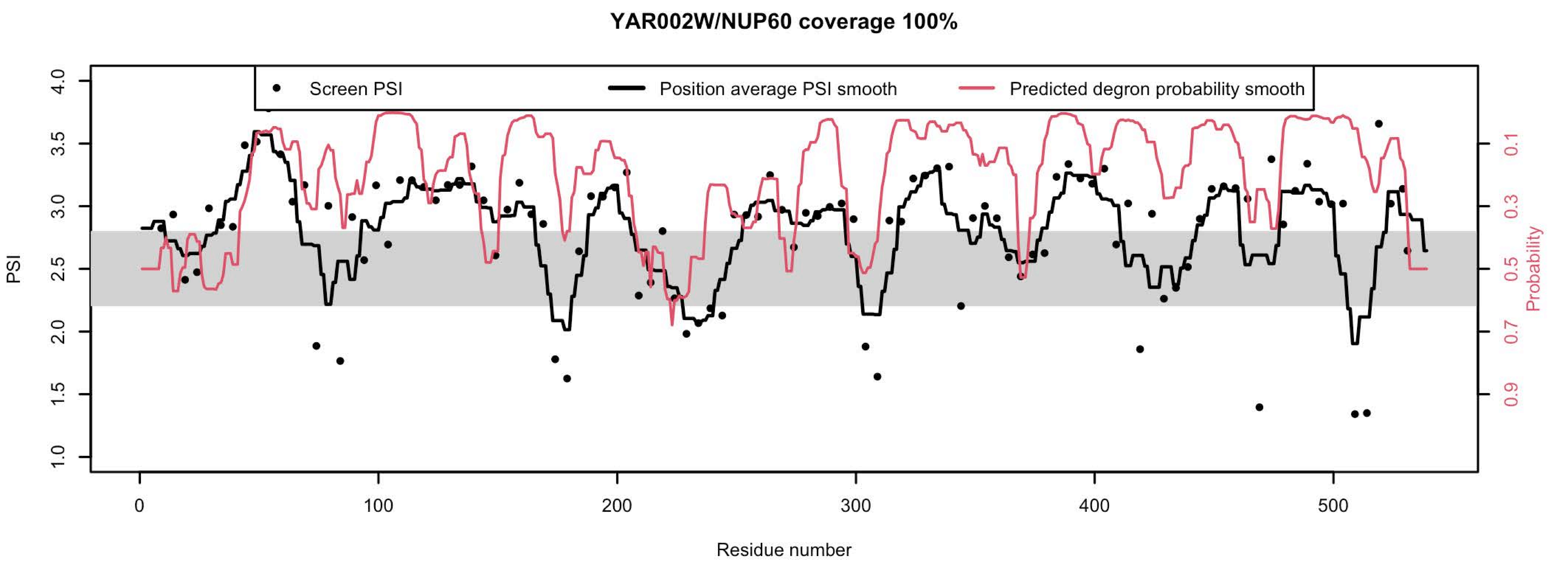
YAR002W/NUP60 coverage 100%
Screen PSI
Position average PSI smooth
Predicted degron probability smooth
PSI
Probability
Residue number
1.0
1.5
2.0
2.5
3.0
3.5
4.0
0.1
0.3
0.5
0.7
0.9
0
100
200
300
400
500

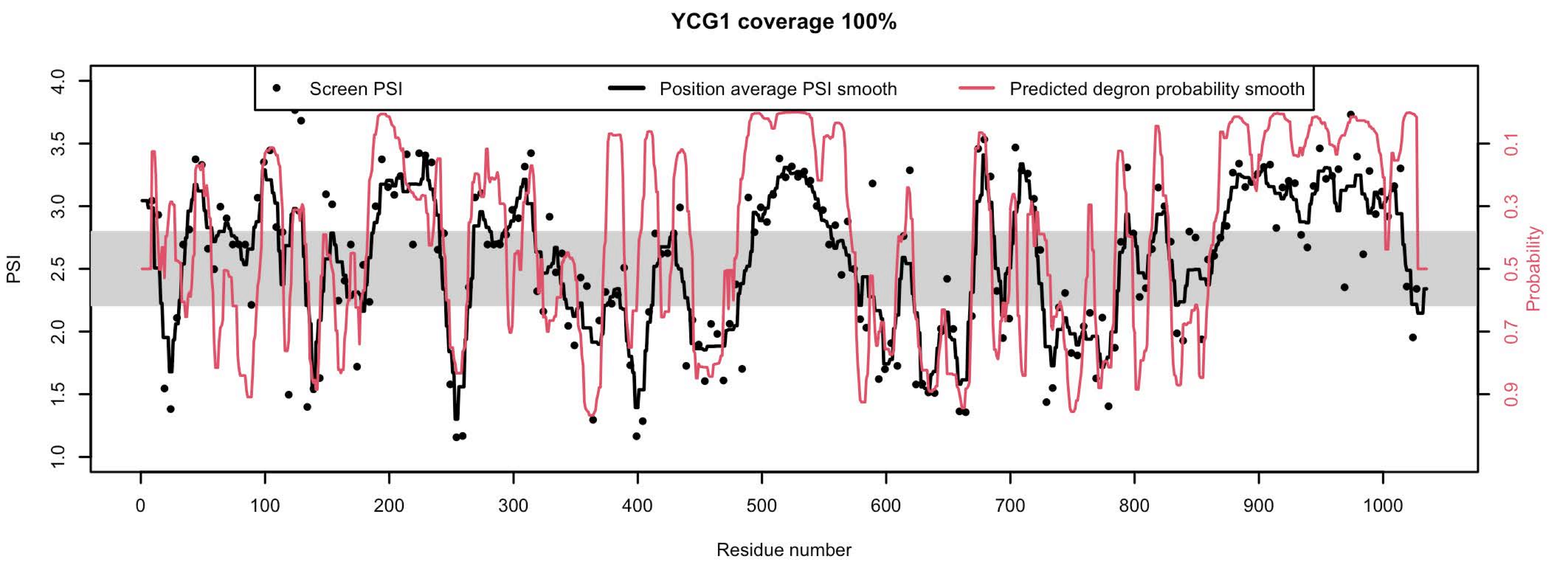
YCG1 coverage 100%
Screen PSI
Position average PSI smooth
Predicted degron probability smooth
PSI
Probability
Residue number
1.0
1.5
2.0
2.5
3.0
3.5
4.0
0.1
0.3
0.5
0.7
0.9
0
100
200
300
400
500
600
700
800
900
1000

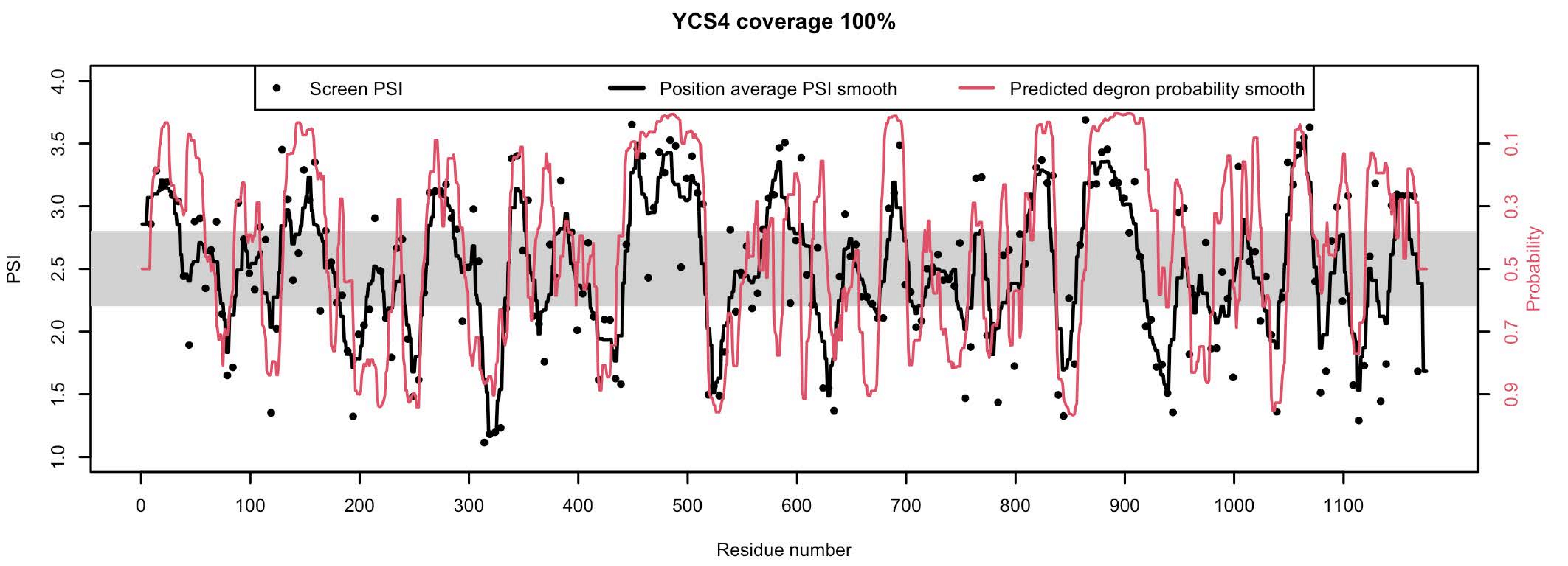

YCS4 coverage 100%
Screen PSI
Position average PSI smooth
Predicted degron probability smooth
PSI
Probability
Residue number
1.0
1.5
2.0
2.5
3.0
3.5
4.0
0.1
0.3
0.5
0.7
0.9
0
100
200
300
400
500
600
700
800
900
1000
1100

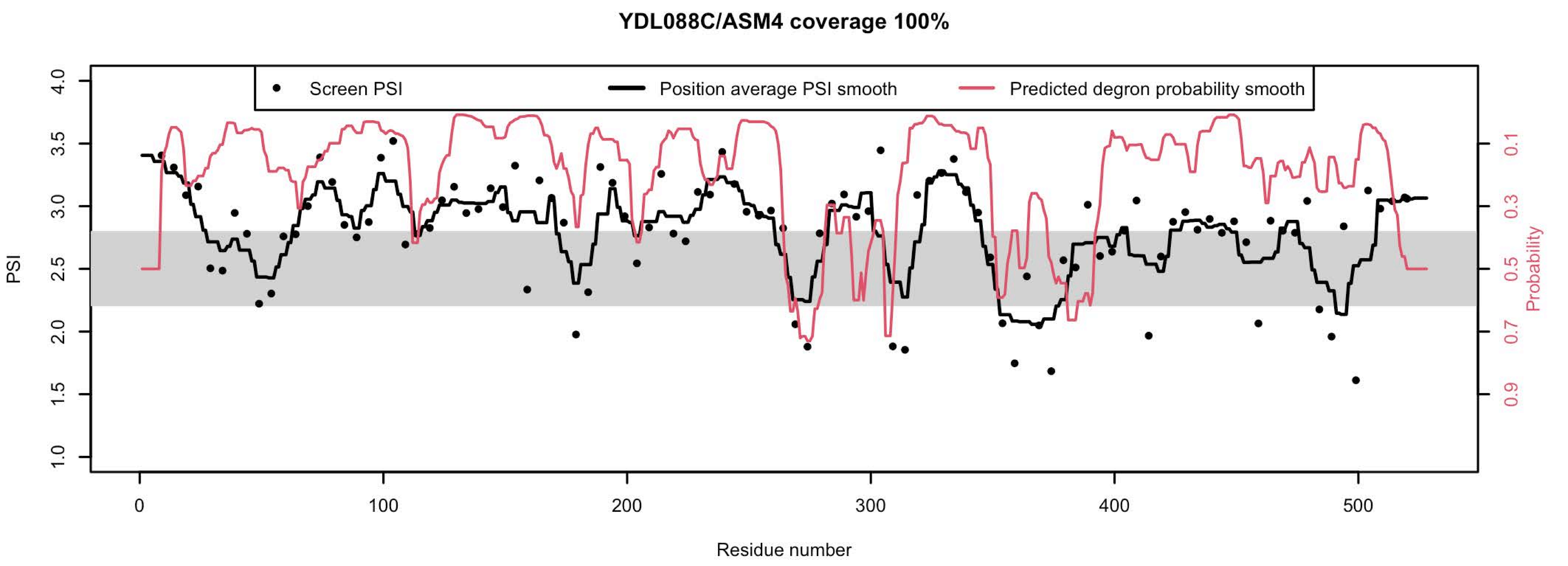
YDL088C/ASM4 coverage 100%
Screen PSI
Position average PSI smooth
Predicted degron probability smooth
PSI
Probability
Residue number
1.0
1.5
2.0
2.5
3.0
3.5
4.0
0.1
0.3
0.5
0.7
0.9
0
100
200
300
400
500

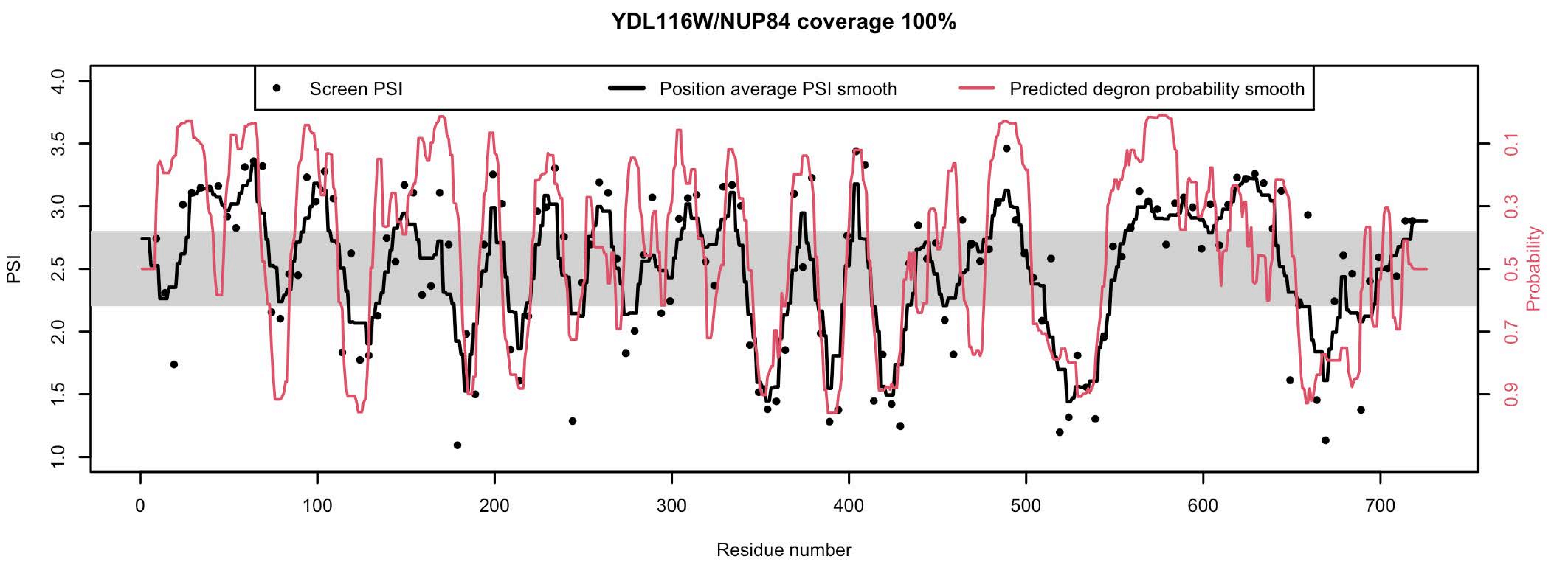
YDL116W/NUP84 coverage 100%
Screen PSI
Position average PSI smooth
Predicted degron probability smooth
PSI
1.0
1.5
2.0
2.5
3.0
3.5
4.0
Probability
0.1
0.3
0.5
0.7
0.9
Residue number
0
100
200
300
400
500
600
700

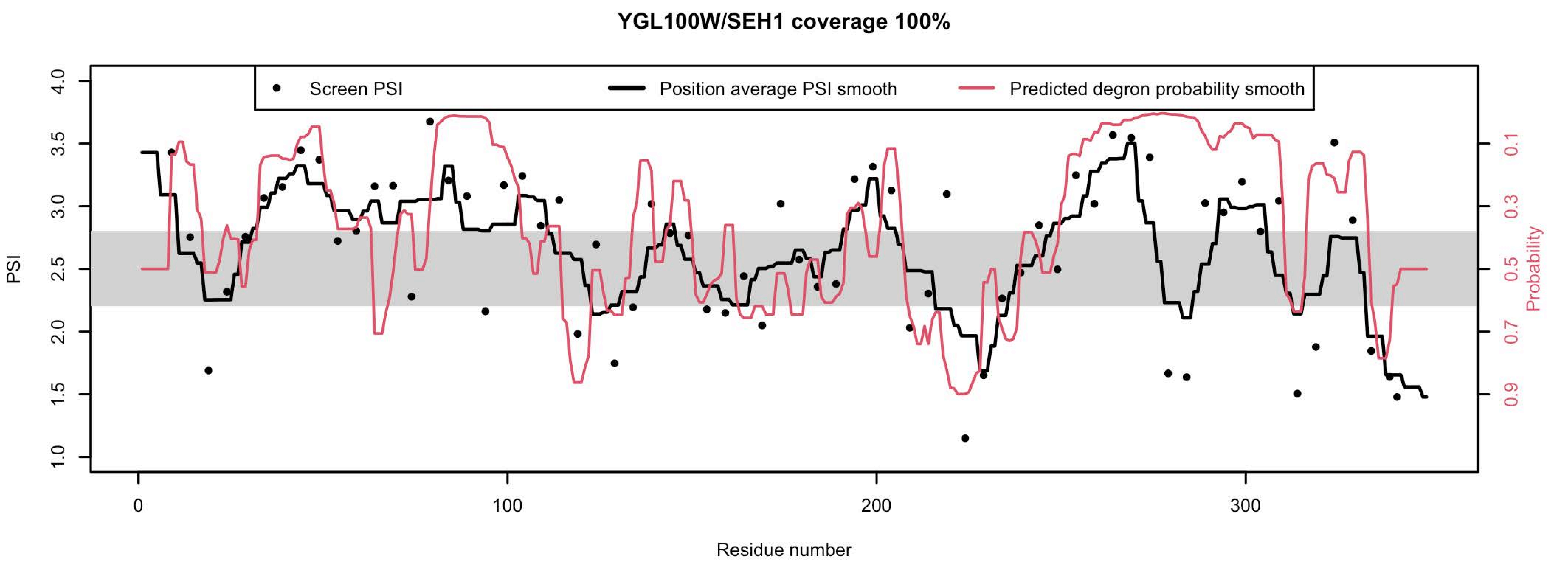
YGL100W/SEH1 coverage 100%
Screen PSI
Position average PSI smooth
Predicted degron probability smooth
PSI
4.0
3.5
3.0
2.5
2.0
1.5
1.0
Probability
0.1
0.3
0.5
0.7
0.9
0
100
200
300
Residue number

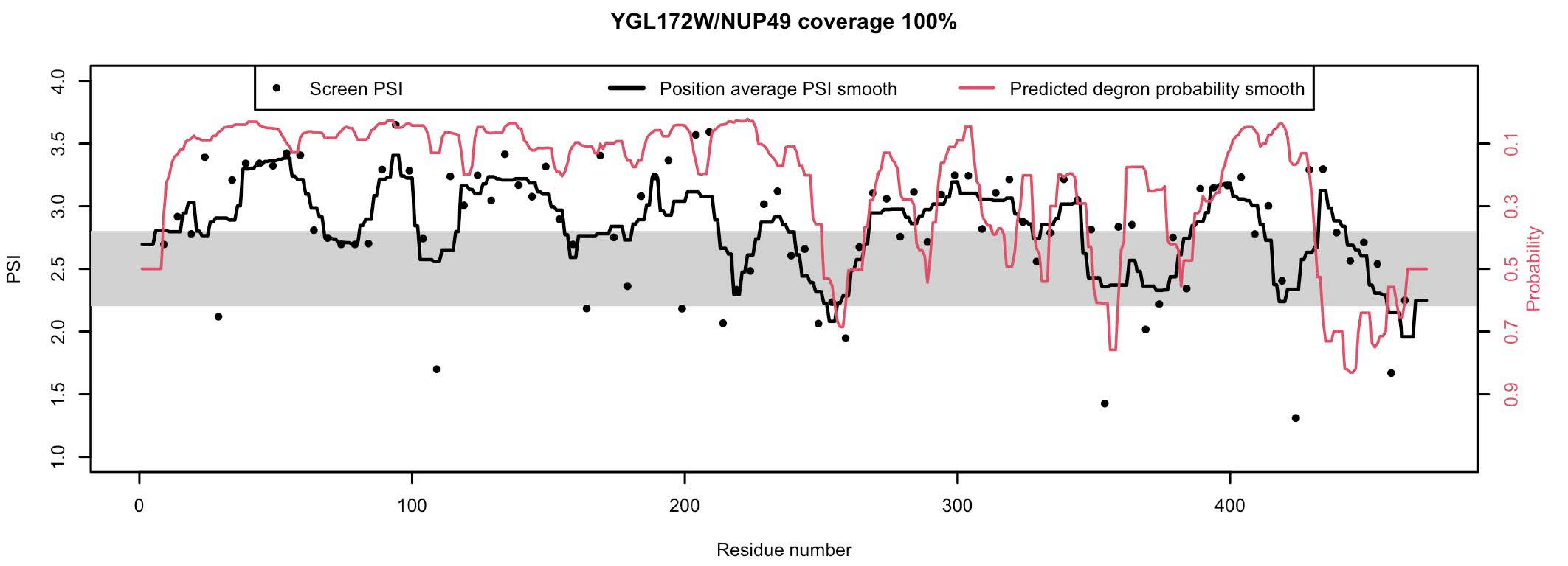
YGL172W/NUP49 coverage 100%
Screen PSI
Position average PSI smooth
Predicted degron probability smooth
PSI
Probability
Residue number
1.0
1.5
2.0
2.5
3.0
3.5
4.0
0.1
0.3
0.5
0.7
0.9
0
100
200
300
400

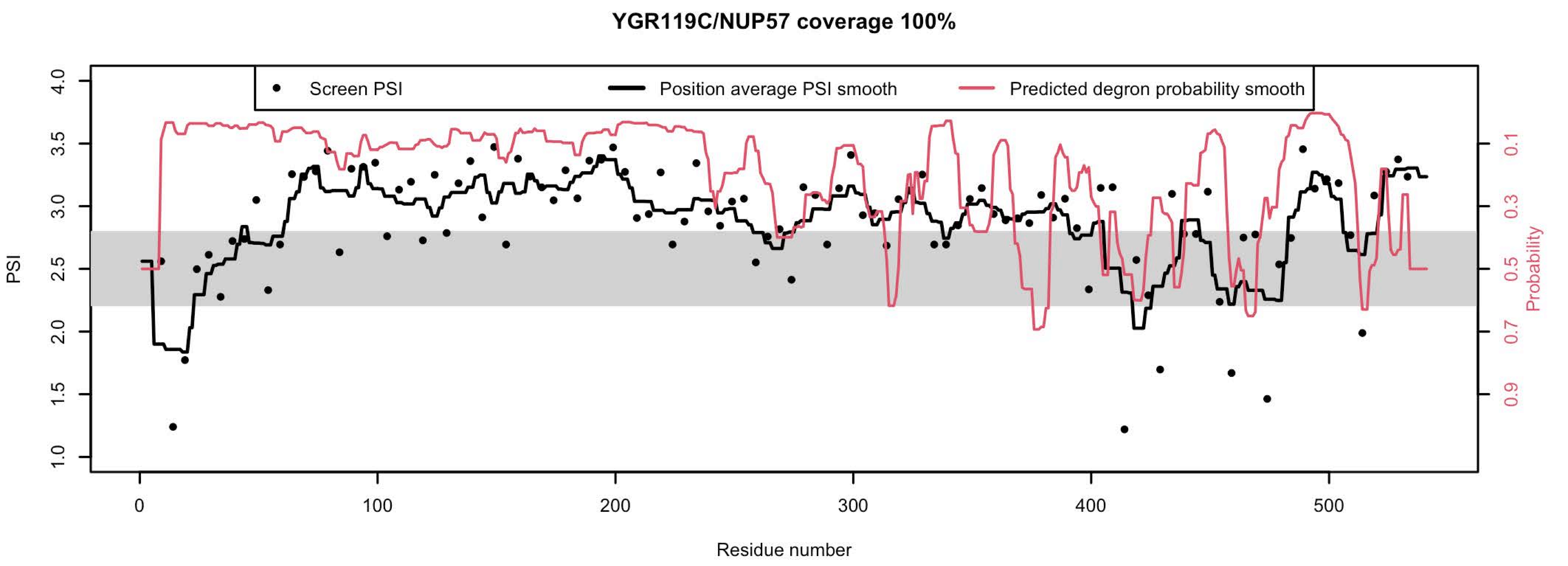
YGR119C/NUP57 coverage 100%
Screen PSI
Position average PSI smooth
Predicted degron probability smooth
PSI
Probability
Residue number
1.0
1.5
2.0
2.5
3.0
3.5
4.0
0.1
0.3
0.5
0.7
0.9
0
100
200
300
400
500

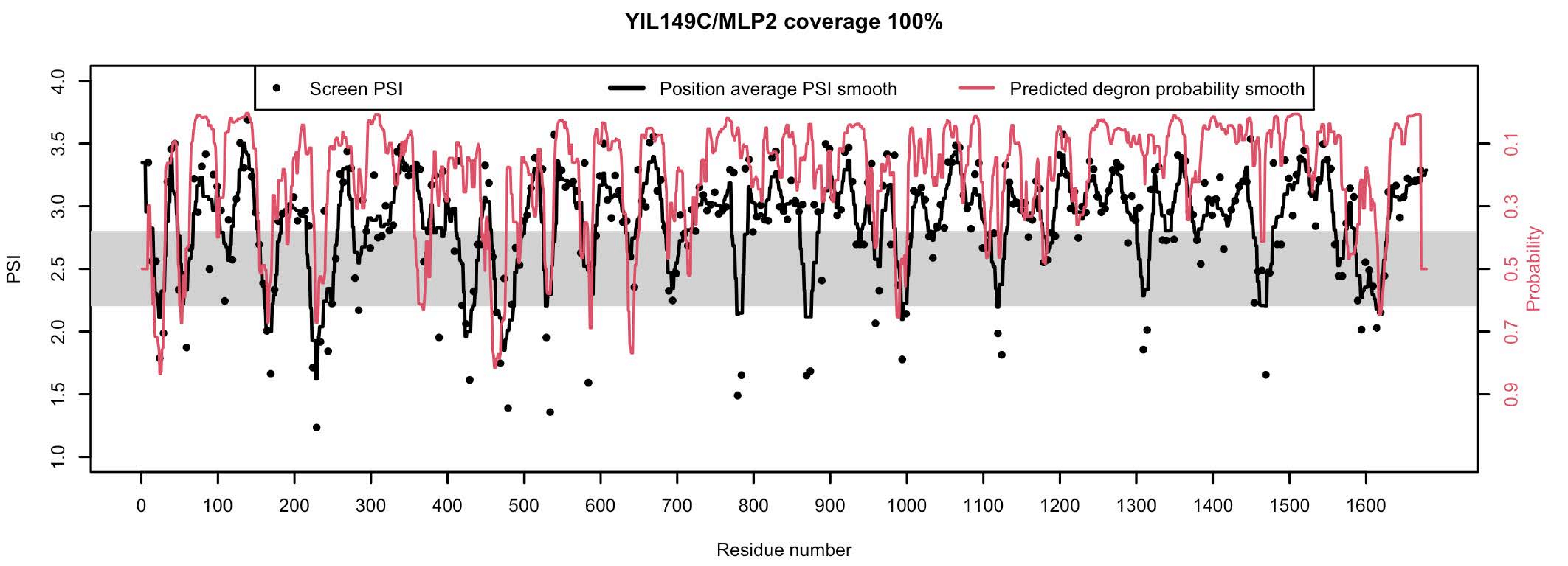

YIL149C/MLP2 coverage 100%
Screen PSI
Position average PSI smooth
Predicted degron probability smooth
PSI
Probability
Residue number
1.0
1.5
2.0
2.5
3.0
3.5
4.0
0.1
0.3
0.5
0.7
0.9
0
100
200
300
400
500
600
700
800
900
1000
1100
1200
1300
1400
1500
1600

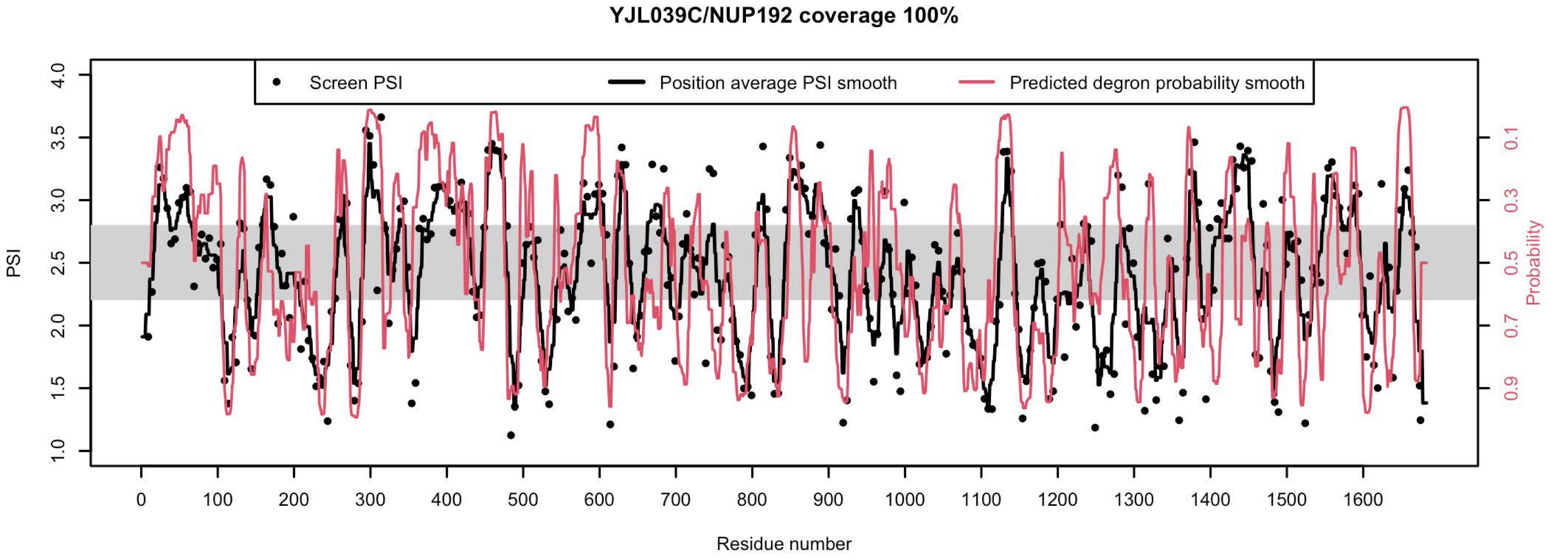

YJL039C/NUP192 coverage 100%
Screen PSI
Position average PSI smooth
Predicted degron probability smooth
PSI
1.0
1.5
2.0
2.5
3.0
3.5
4.0
Probability
0.1
0.3
0.5
0.7
0.9
Residue number
0
100
200
300
400
500
600
700
800
900
1000
1100
1200
1300
1400
1500
1600

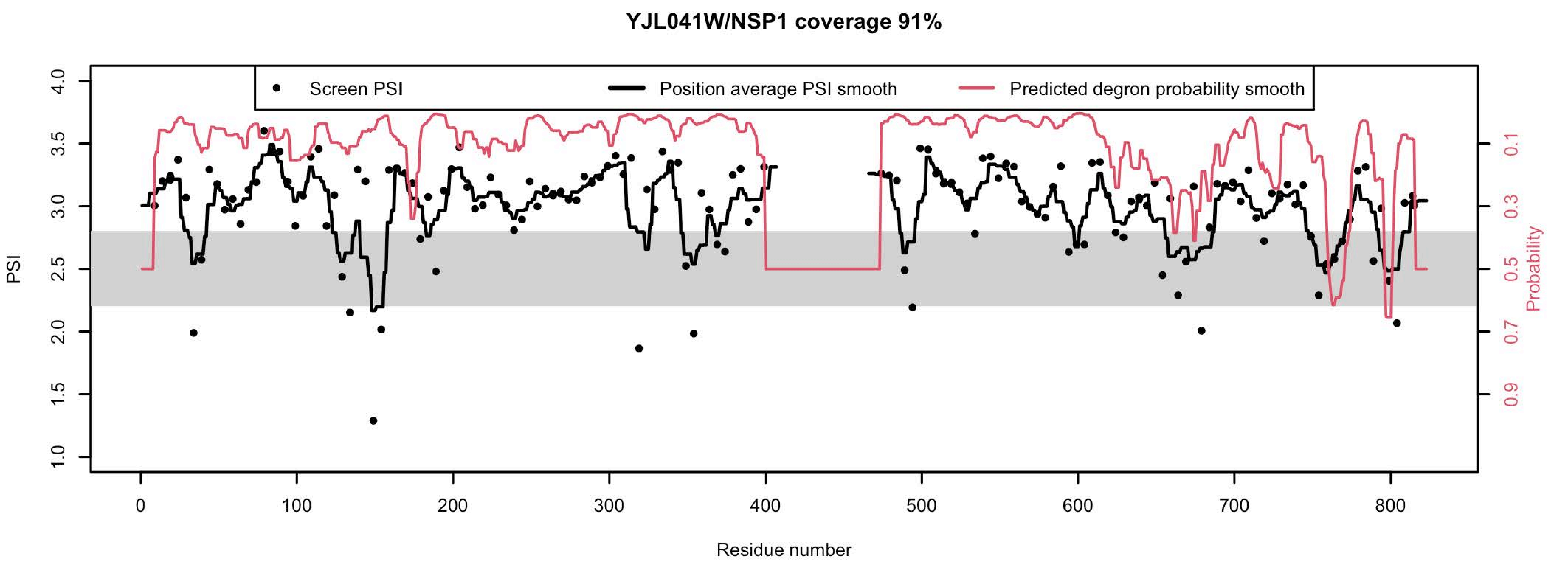

YJL041W/NSP1 coverage 91%
Screen PSI
Position average PSI smooth
Predicted degron probability smooth
PSI
Probability
Residue number
1.0
1.5
2.0
2.5
3.0
3.5
4.0
0.1
0.3
0.5
0.7
0.9
0
100
200
300
400
500
600
700
800

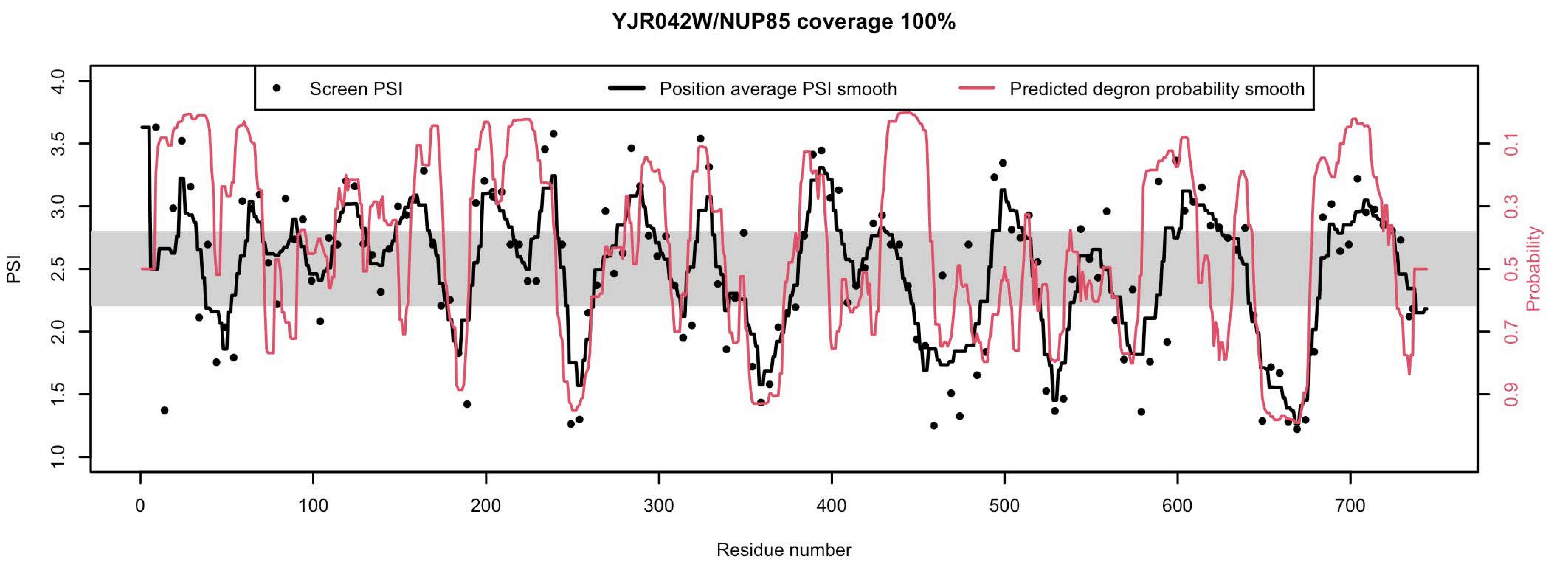

YJR042W/NUP85 coverage 100%
Screen PSI
Position average PSI smooth
Predicted degron probability smooth
PSI
Probability
Residue number
1.0
1.5
2.0
2.5
3.0
3.5
4.0
0.1
0.3
0.5
0.7
0.9
0
100
200
300
400
500
600
700

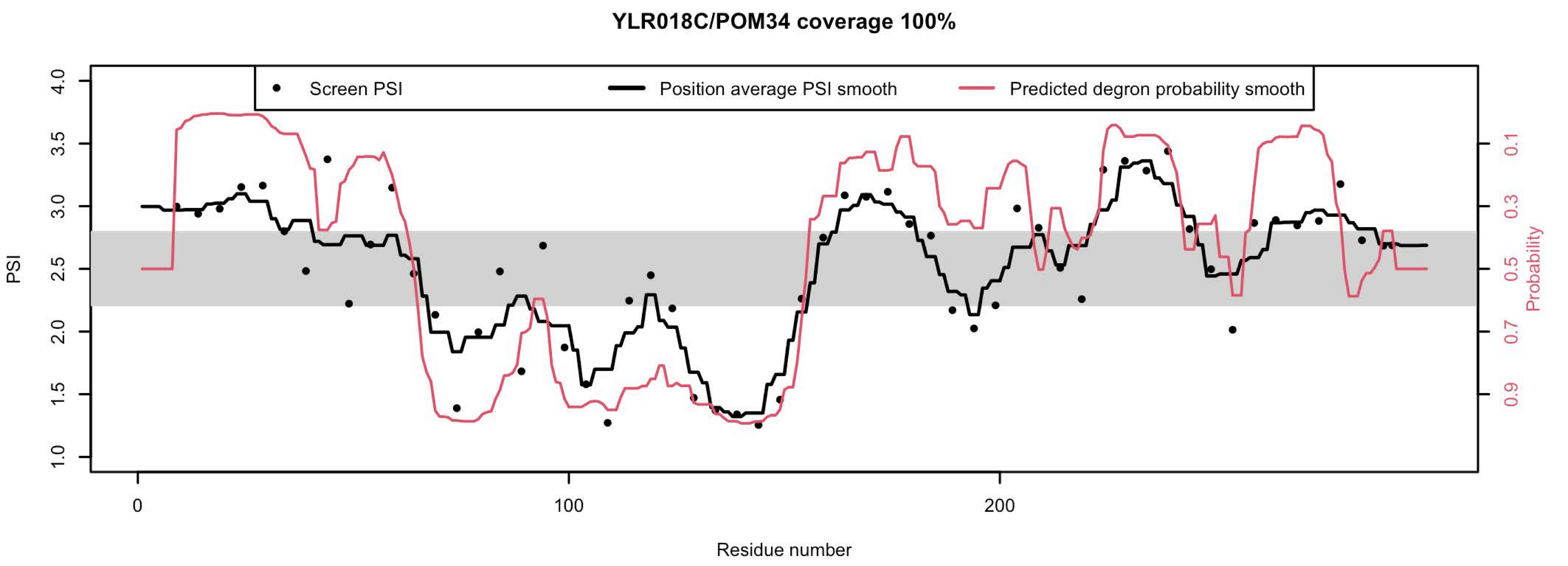

YLR018C/POM34 coverage 100%
Screen PSI
Position average PSI smooth
Predicted degron probability smooth
PSI
1.0
1.5
2.0
2.5
3.0
3.5
4.0
Probability
0.9
0.7
0.5
0.3
0.1
0
100
200
Residue number

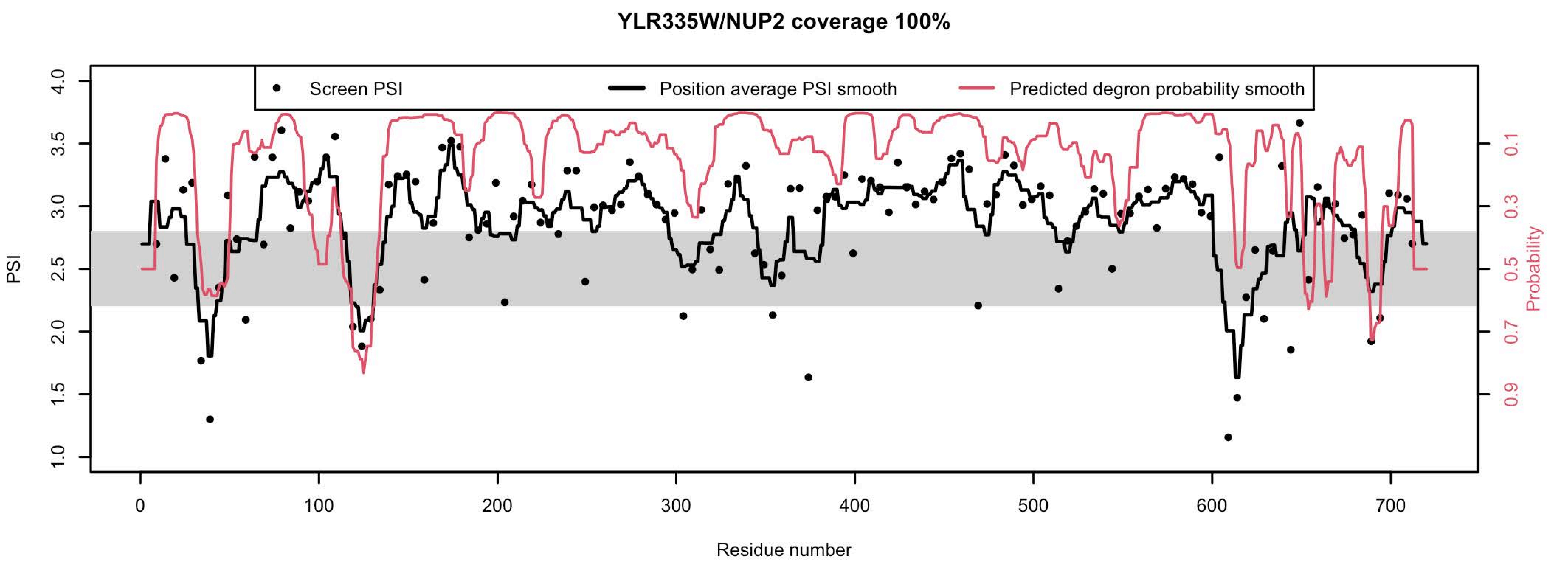

YLR335W/NUP2 coverage 100%
Screen PSI
Position average PSI smooth
Predicted degron probability smooth
PSI
Probability
Residue number
1.0
1.5
2.0
2.5
3.0
3.5
4.0
0.1
0.3
0.5
0.7
0.9
0
100
200
300
400
500
600
700

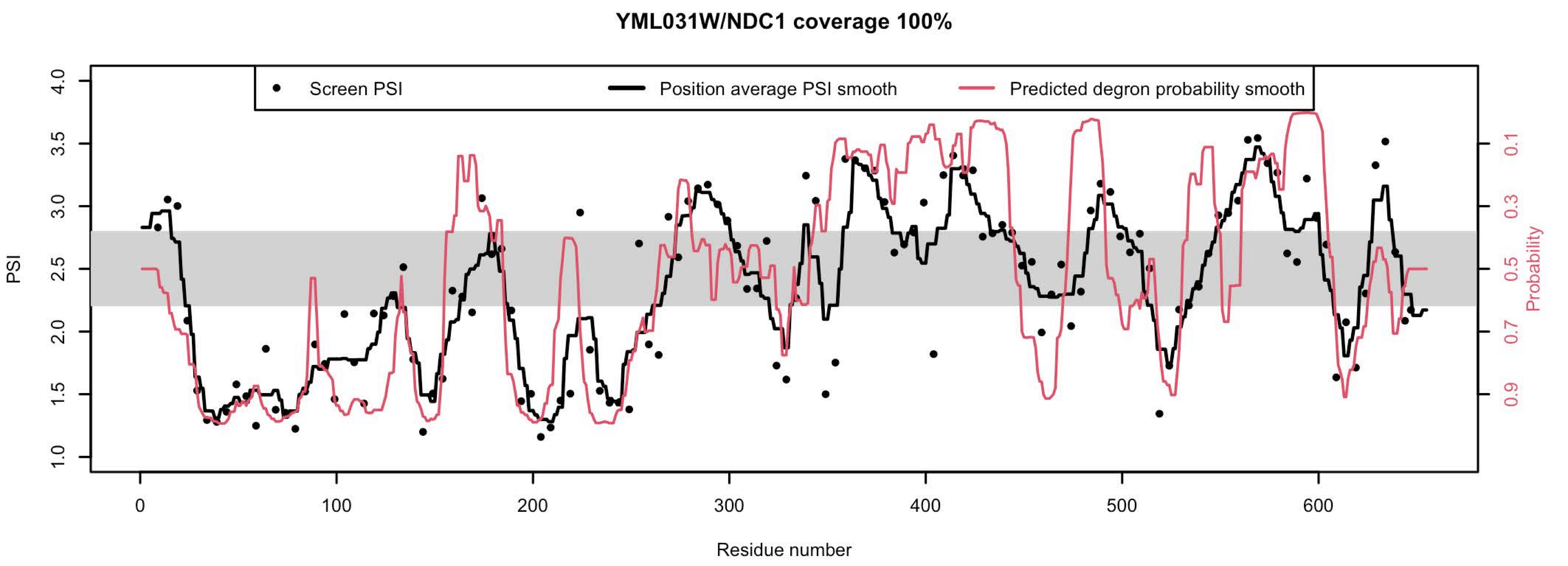

YML031W/NDC1 coverage 100%
Screen PSI
Position average PSI smooth
Predicted degron probability smooth
PSI
Probability
Residue number
1.0
1.5
2.0
2.5
3.0
3.5
4.0
0.1
0.3
0.5
0.7
0.9
0
100
200
300
400
500
600

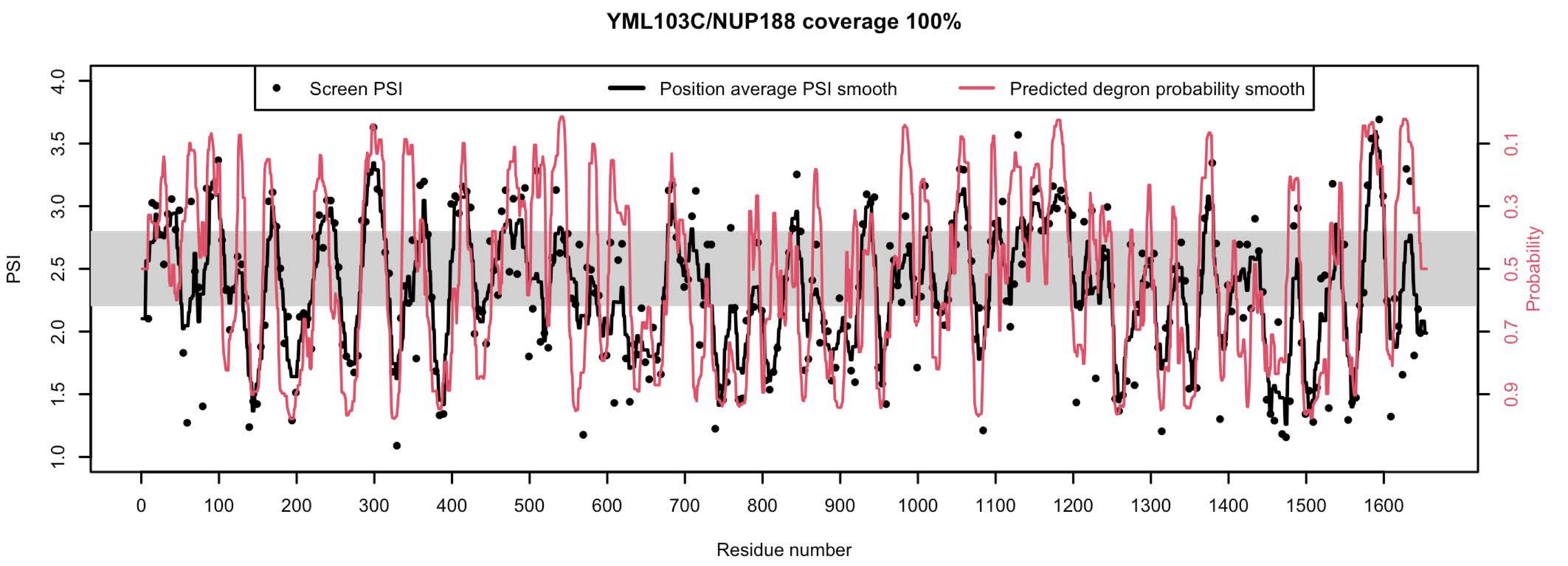

YML103C/NUP188 coverage 100%
Screen PSI
Position average PSI smooth
Predicted degron probability smooth
PSI
Probability
Residue number
1.0
1.5
2.0
2.5
3.0
3.5
4.0
0.1
0.3
0.5
0.7
0.9
0
100
200
300
400
500
600
700
800
900
1000
1100
1200
1300
1400
1500
1600

YMR129W/POM152 coverage 100%

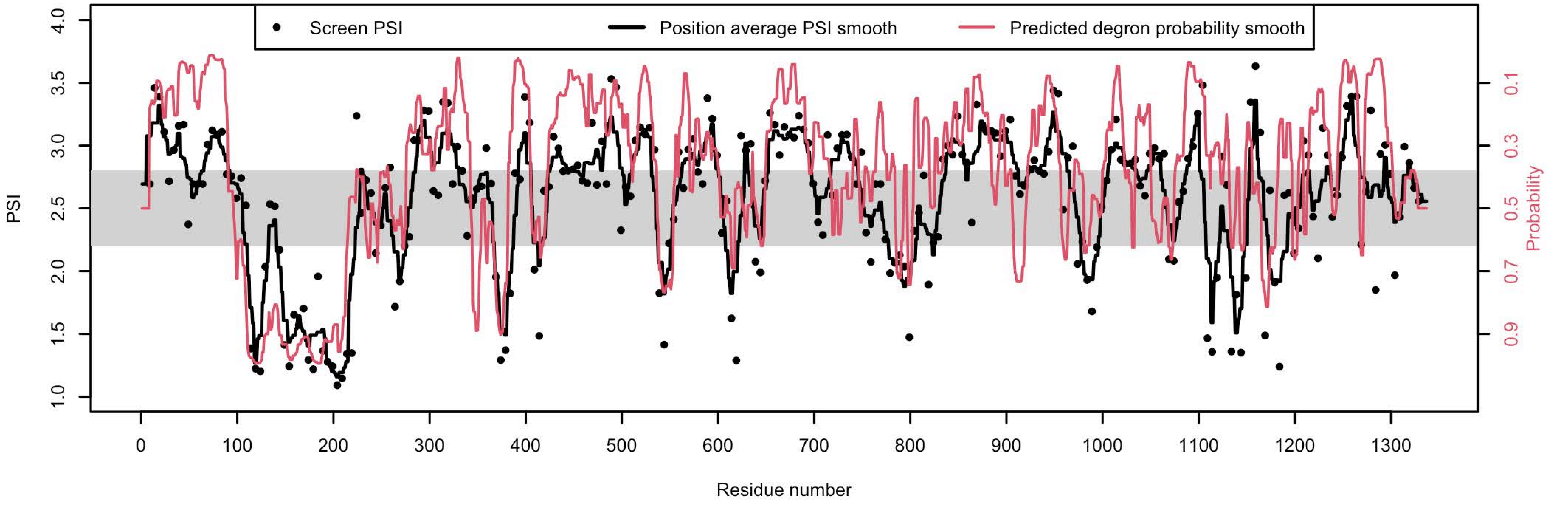

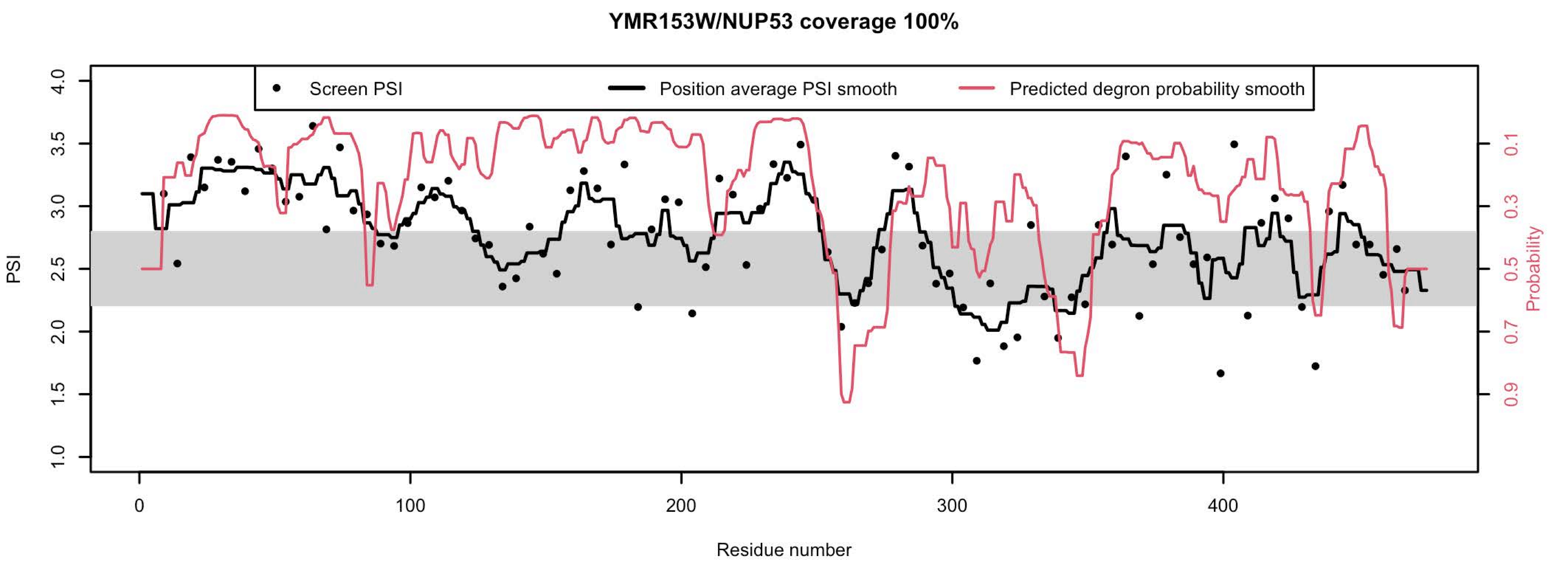

YMR153W/NUP53 coverage 100%
Screen PSI
Position average PSI smooth
Predicted degron probability smooth
PSI
1.0
1.5
2.0
2.5
3.0
3.5
4.0
Probability
0.1
0.3
0.5
0.7
0.9
0
100
200
300
400
Residue number

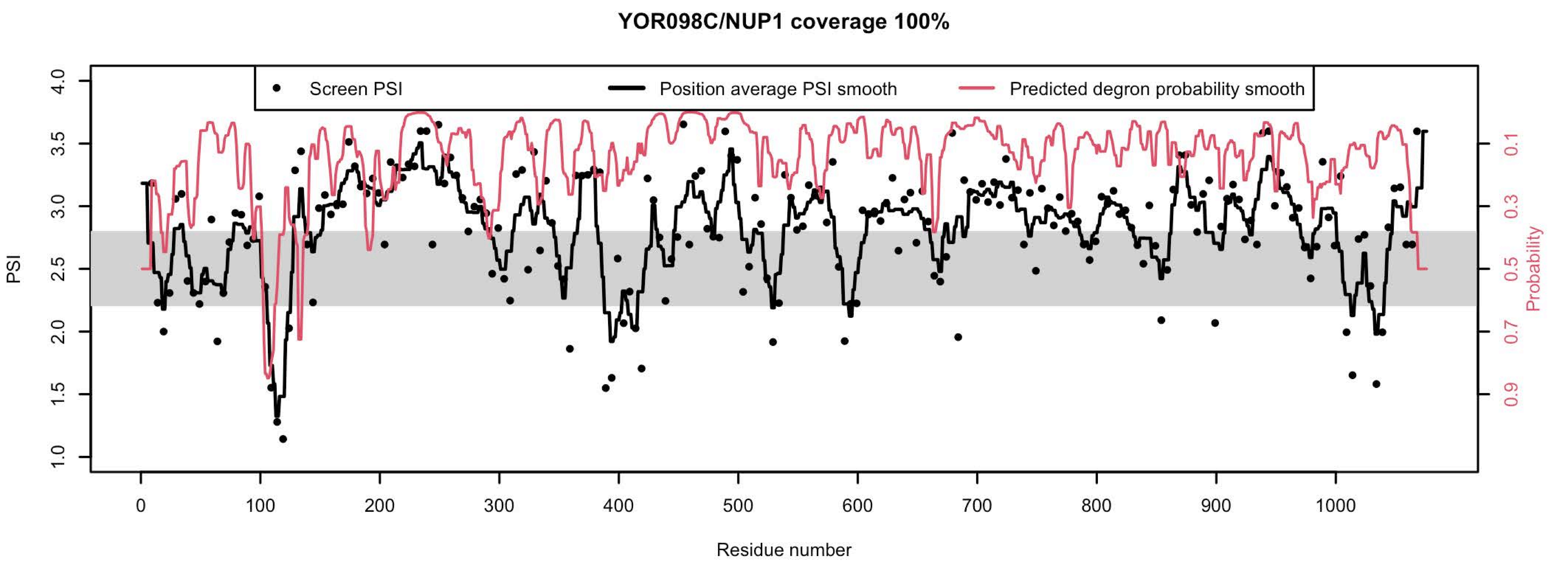
YOR098C/NUP1 coverage 100%
Screen PSI
Position average PSI smooth
Predicted degron probability smooth
PSI
Probability
Residue number
1.0
1.5
2.0
2.5
3.0
3.5
4.0
0.1
0.3
0.5
0.7
0.9
0
100
200
300
400
500
600
700
800
900
1000
